# Supplementary material for: Human acrocentric chromosome short arm de novo mutation and recombination
Source: bioRxiv. 2025 Dec 17:2025.12.16.694519. Preprint. [Version 1] doi: 10.64898/2025.12.16.694519 (PMC12724521; doi:10.64898/2025.12.16.694519)
Supplement: Supplement 2 [file media-2.pdf]

Data S2

This file contains all ModDotPlots, NucFreq plots and centromere validation plots for 156 pq-scatigs from 16 samples

NA12877

chr13

NA12877_1_haplotype1-0000001_chr13

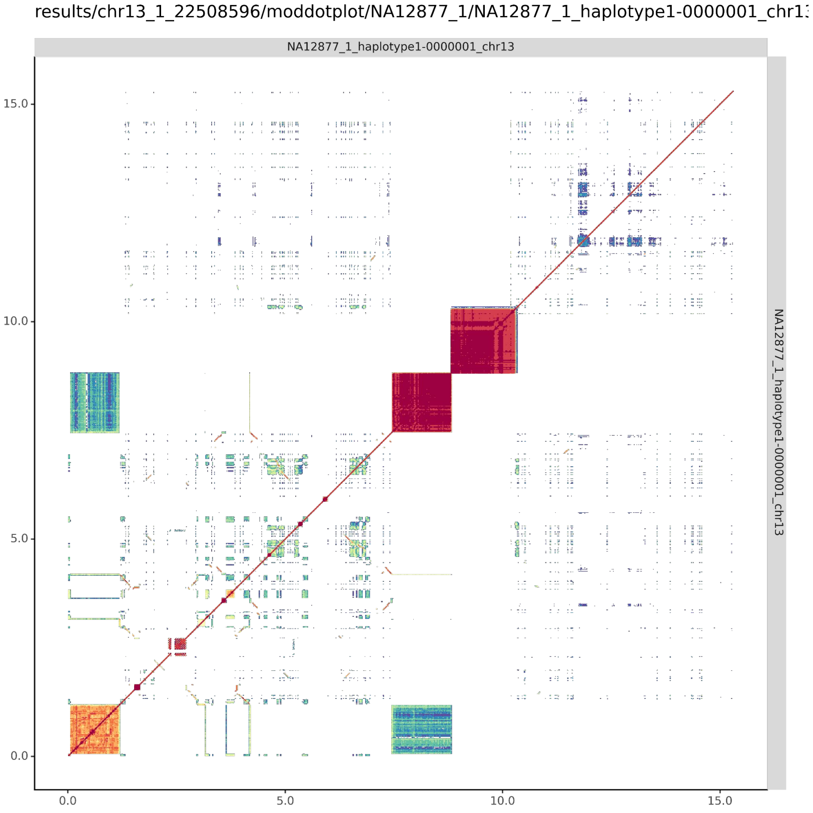

NA12877_2_haplotype2-0000046_chr13

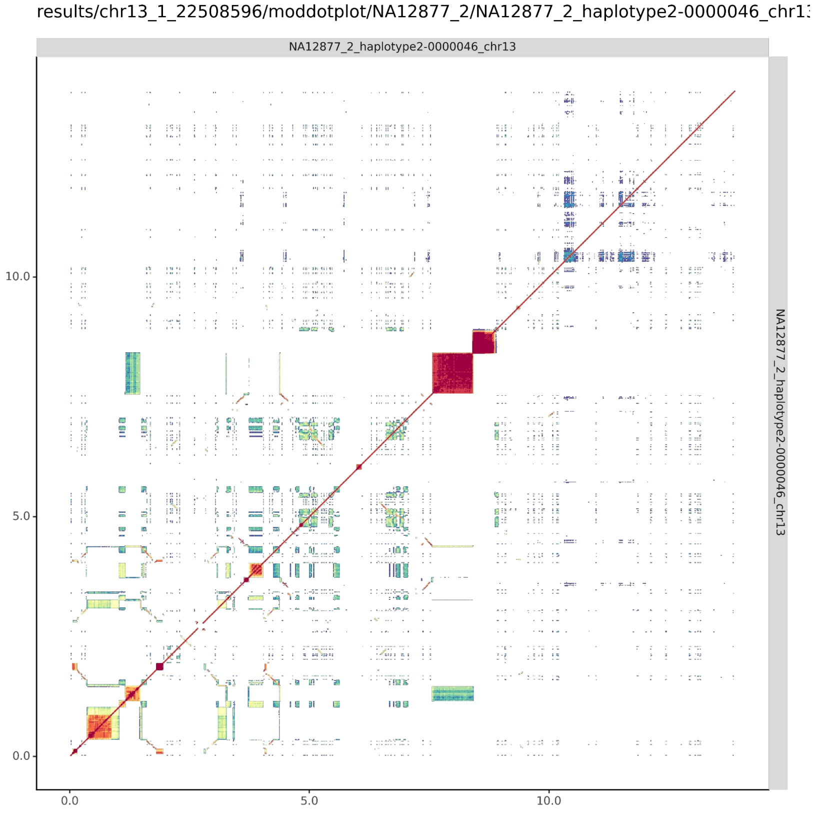

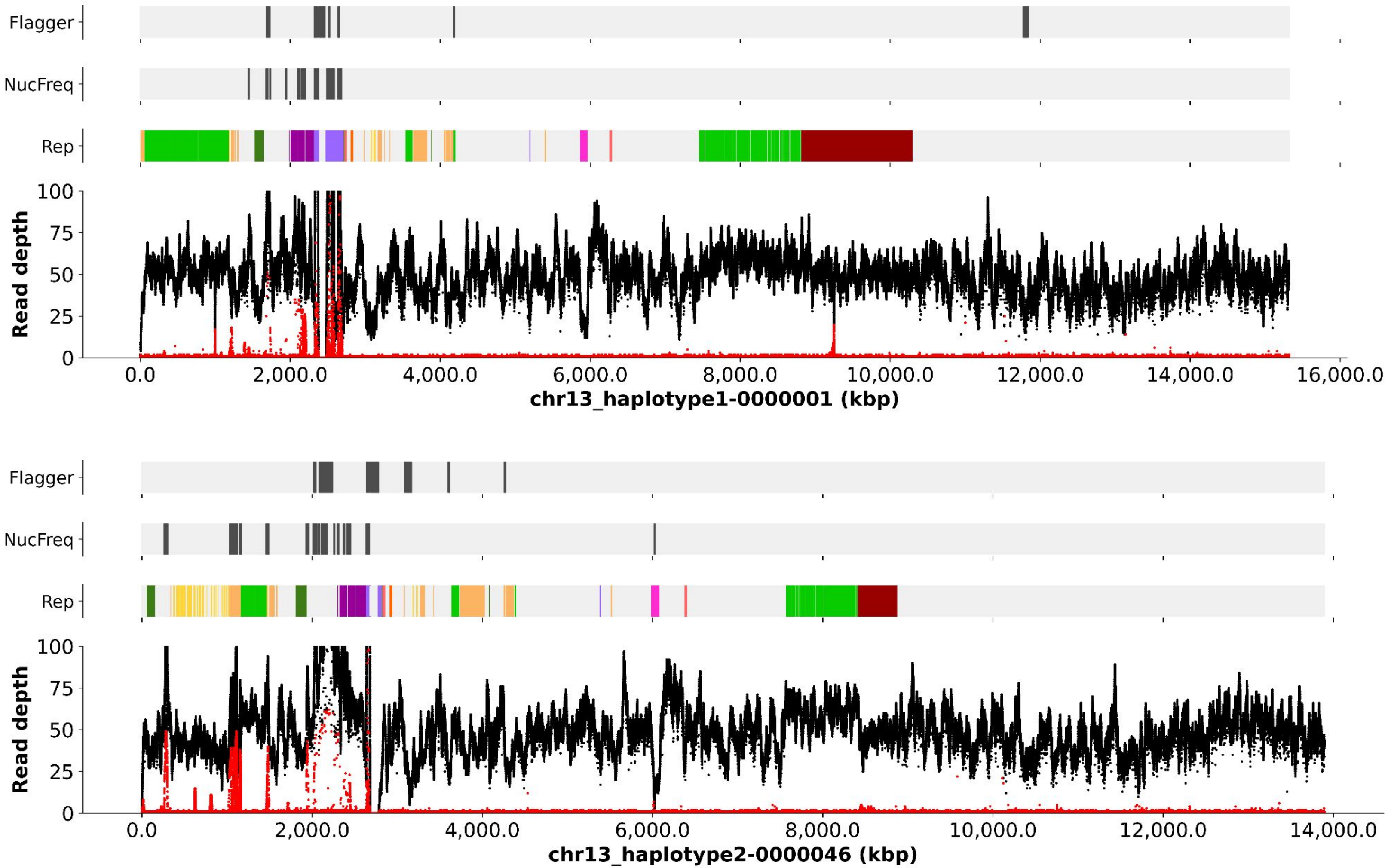

Flagger
NucFreq
Rep
Read depth
0
25
50
75
100
0.0
2,000.0
4,000.0
6,000.0
8,000.0
10,000.0
12,000.0
14,000.0
16,000.0
chr13_haplotype1-0000001 (kbp)
Flagger
NucFreq
Rep
Read depth
0
25
50
75
100
0.0
2,000.0
4,000.0
6,000.0
8,000.0
10,000.0
12,000.0
14,000.0
chr13_haplotype2-0000046 (kbp)

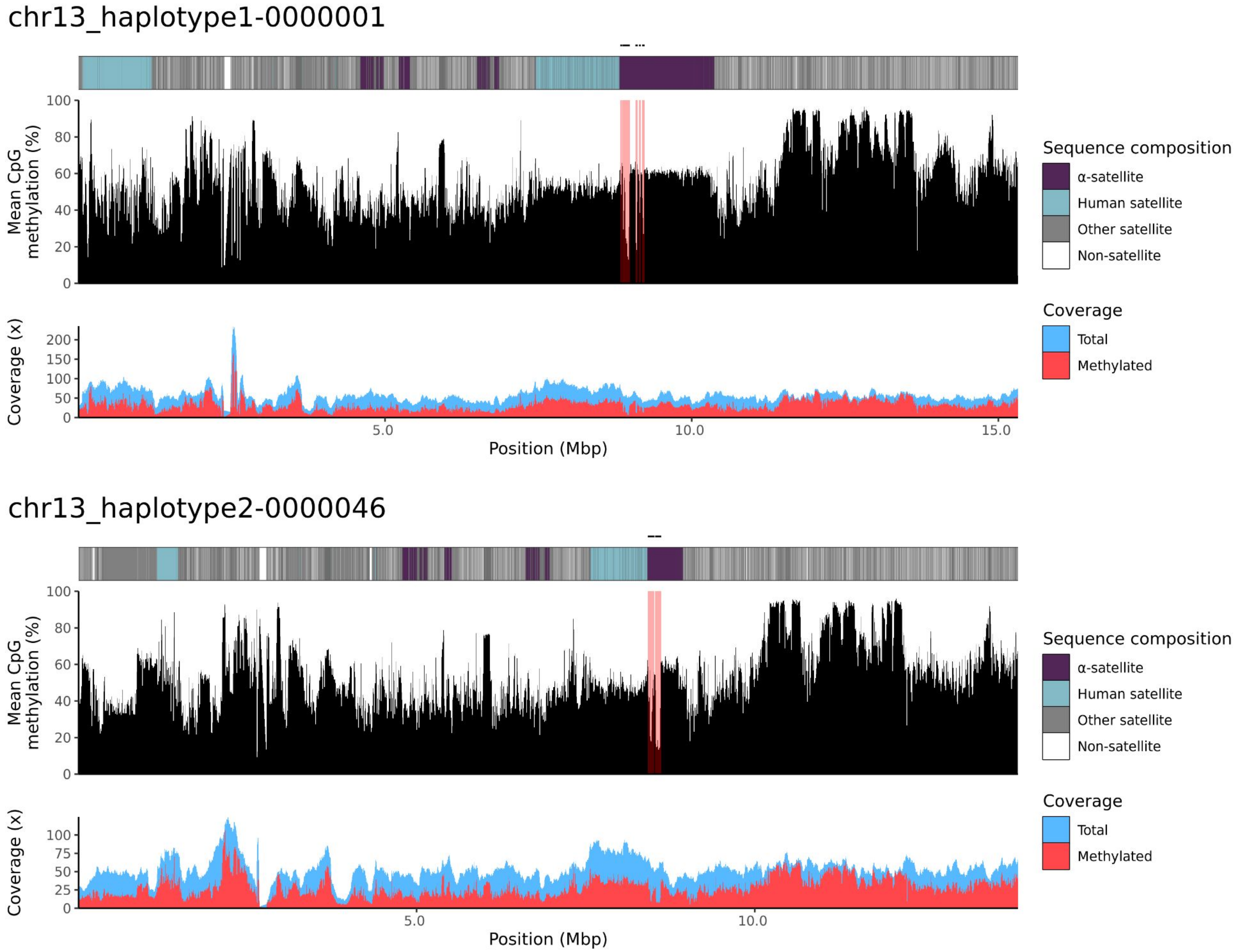
chr13_haplotype1-0000001
Mean CpG methylation (%)
100
80
60
40
20
0
Coverage (x)
200
150
100
50
0
5.0
10.0
15.0
Position (Mbp)
Sequence composition
α-satellite
Human satellite
Other satellite
Non-satellite
Coverage
Total
Methylated
chr13_haplotype2-0000046
Mean CpG methylation (%)
100
80
60
40
20
0
Coverage (x)
100
75
50
25
0
5.0
10.0
Position (Mbp)
Sequence composition
α-satellite
Human satellite
Other satellite
Non-satellite
Coverage
Total
Methylated

chr14

NA12877_1_haplotype1-0000013_chr14

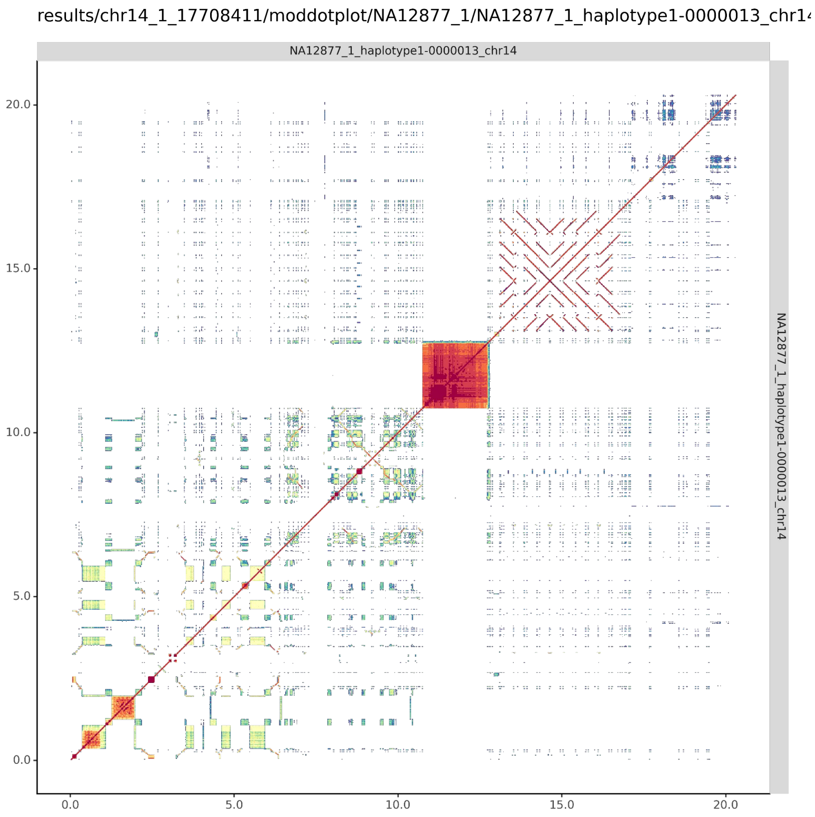

NA12877_2_haplotype2-0000056_chr14

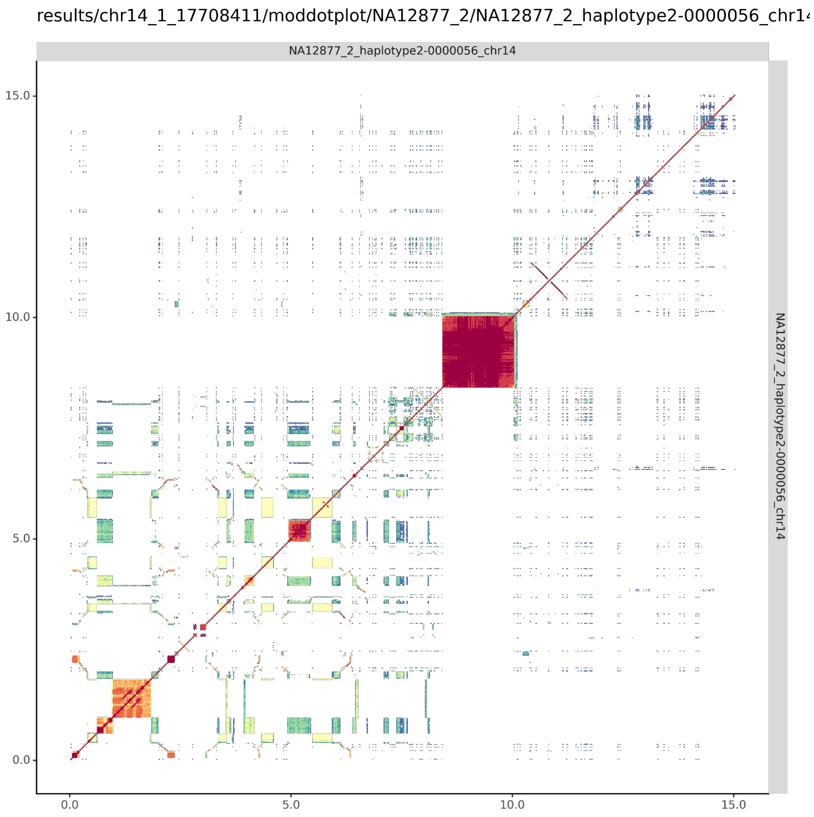

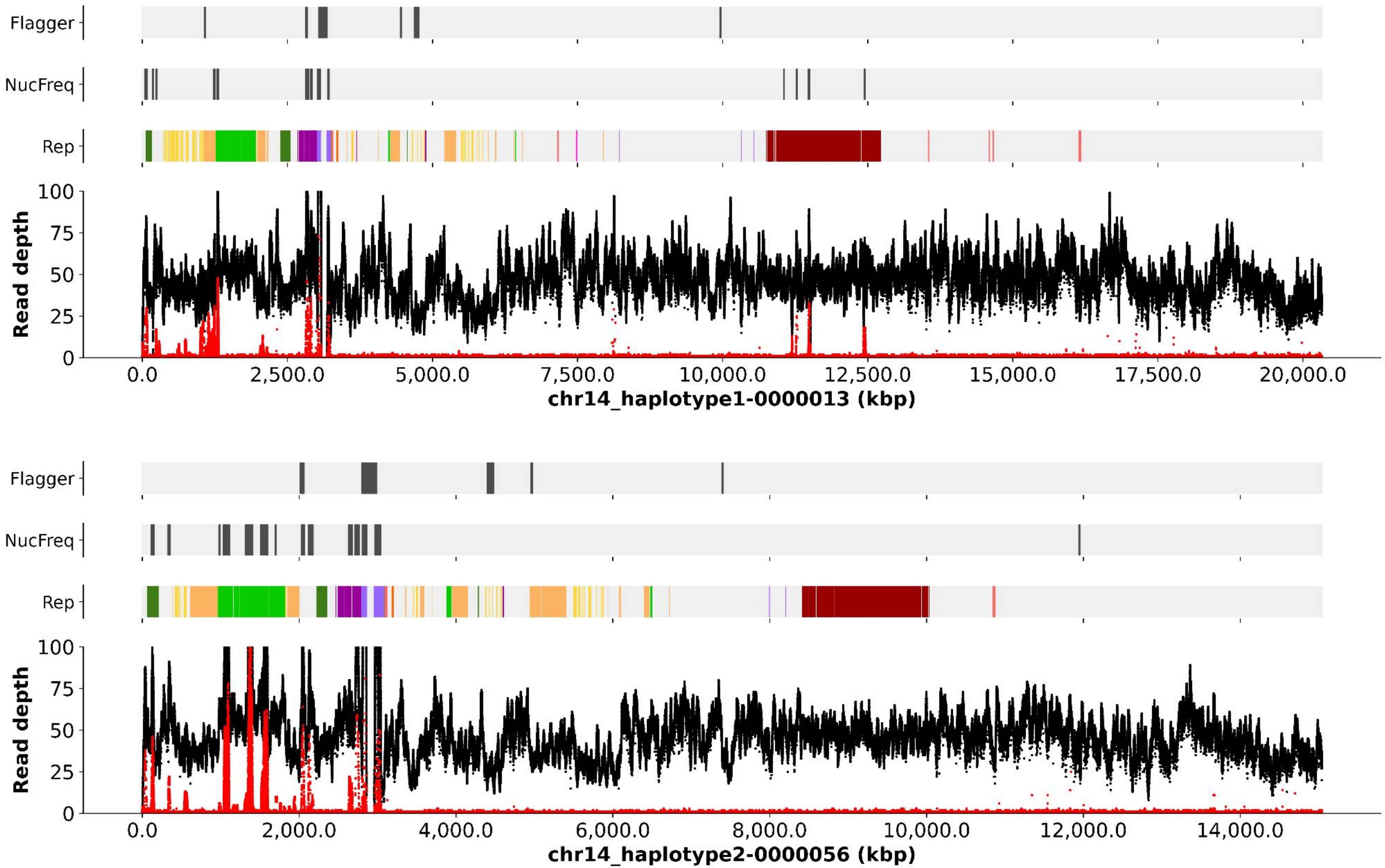

Flagger
NucFreq
Rep
Read depth
100
75
50
25
0
0.0
2,500.0
5,000.0
7,500.0
10,000.0
12,500.0
15,000.0
17,500.0
20,000.0
chr14_haplotype1-0000013 (kbp)
Flagger
NucFreq
Rep
Read depth
100
75
50
25
0
0.0
2,000.0
4,000.0
6,000.0
8,000.0
10,000.0
12,000.0
14,000.0
chr14_haplotype2-0000056 (kbp)

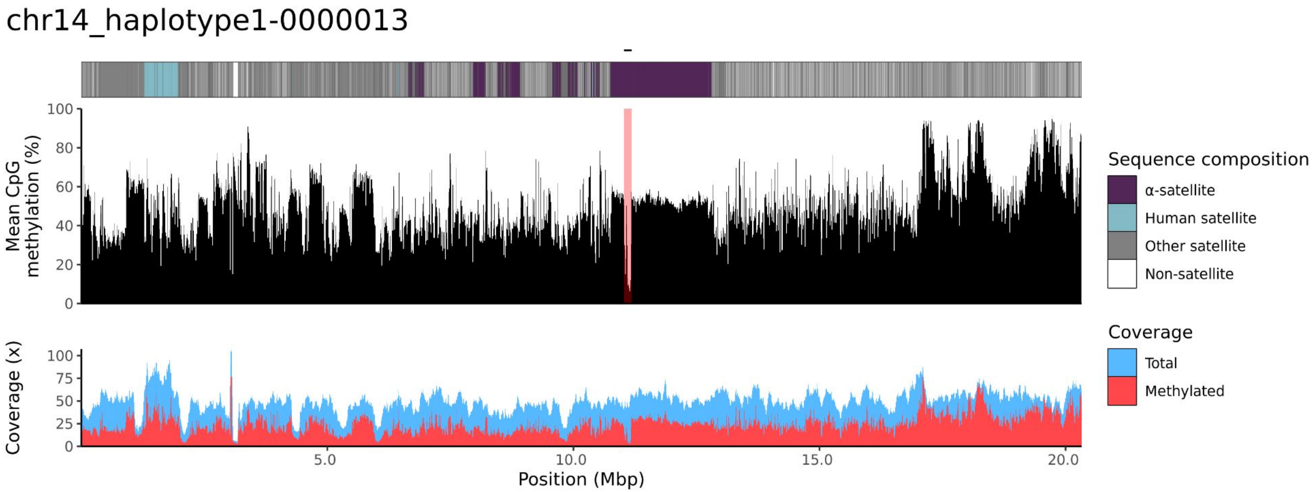
chr14_haplotype1-0000013
Mean CpG methylation (%)
Coverage (x)
Position (Mbp)
5.0
10.0
15.0
20.0
Sequence composition
α-satellite
Human satellite
Other satellite
Non-satellite
Coverage
Total
Methylated

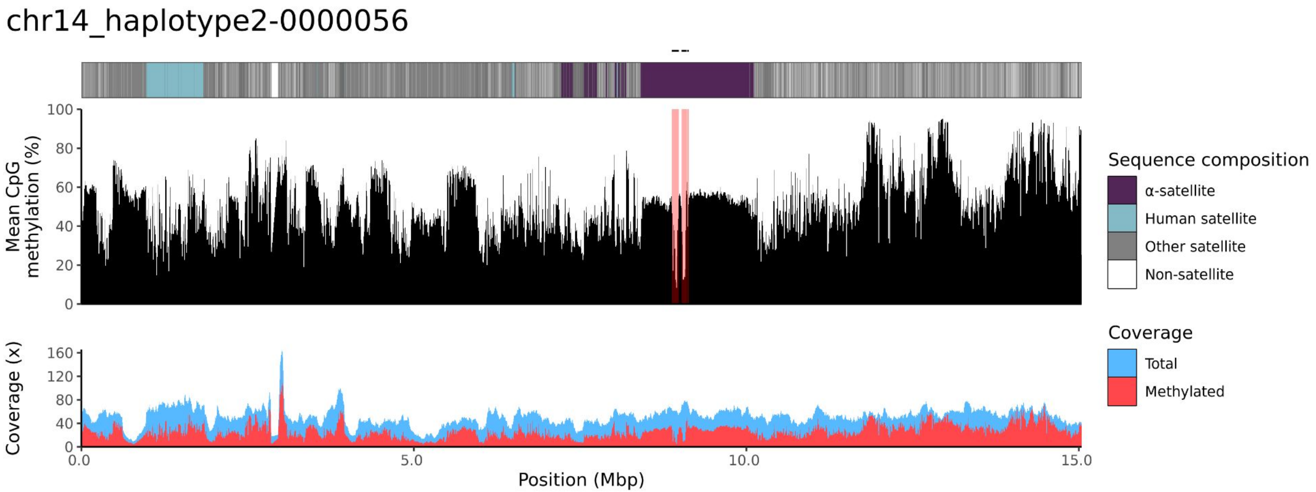
chr14_haplotype2-0000056
Mean CpG methylation (%)
Coverage (x)
Position (Mbp)
0.0
5.0
10.0
15.0
Sequence composition
α-satellite
Human satellite
Other satellite
Non-satellite
Coverage
Total
Methylated

chr15

NA12877_1_haplotype1-0000014_chr15

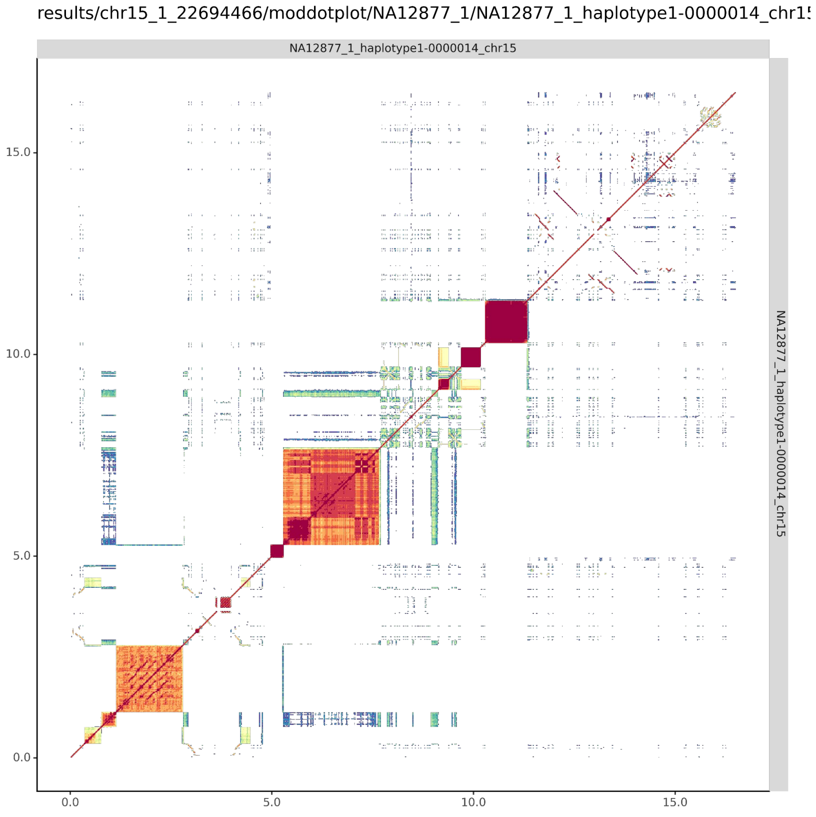

NA12877_2_haplotype2-0000057_chr15

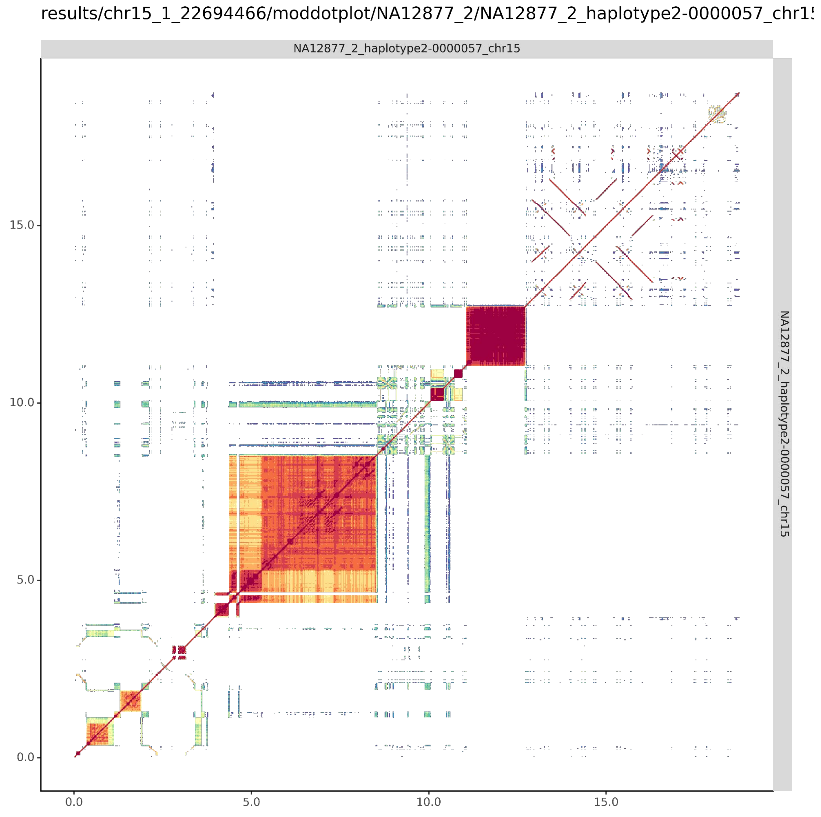

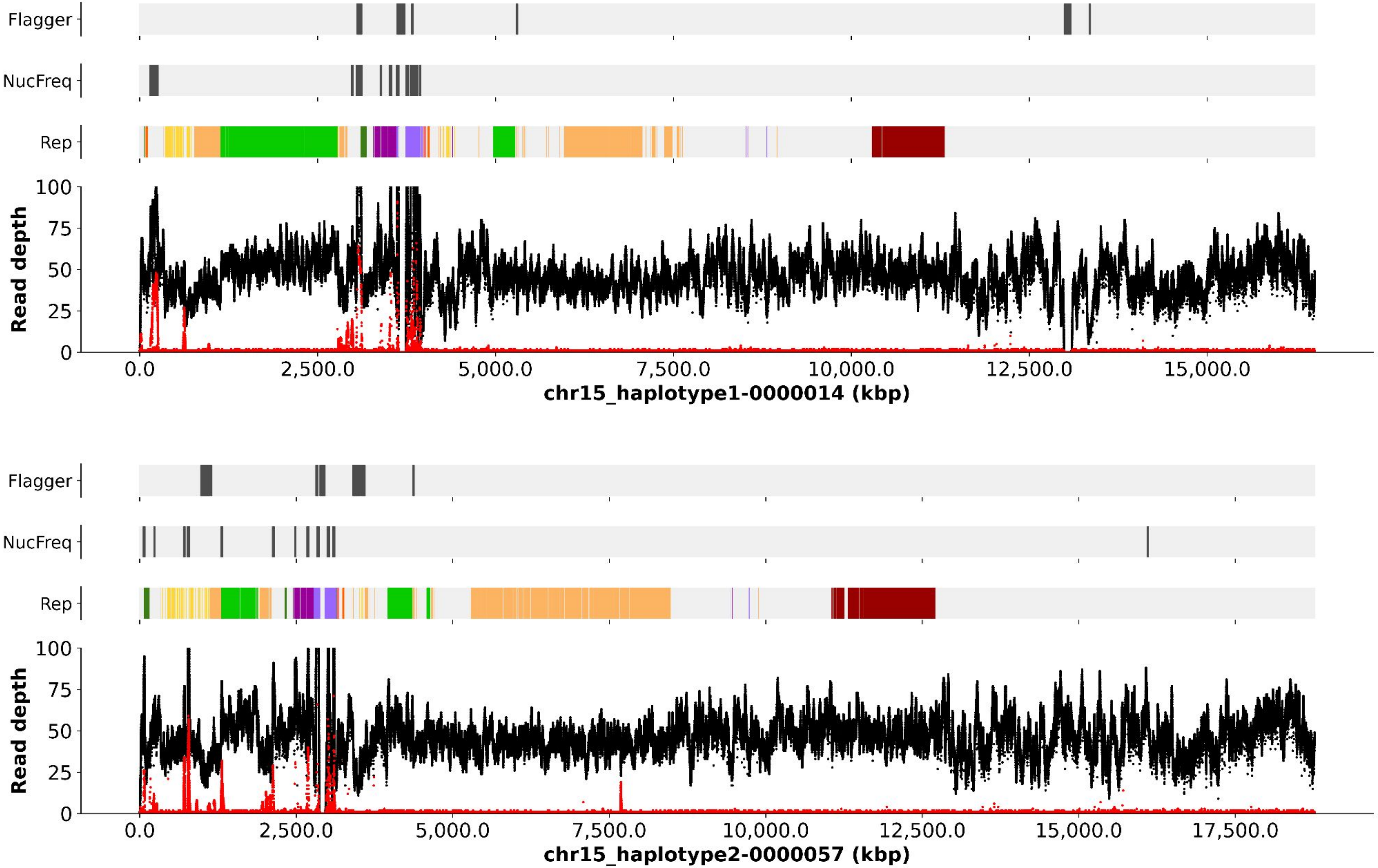
Flagger
NucFreq
Rep
Read depth
100
75
50
25
0
0.0
2,500.0
5,000.0
7,500.0
10,000.0
12,500.0
15,000.0
chr15_haplotype1-0000014 (kbp)
Flagger
NucFreq
Rep
Read depth
100
75
50
25
0
0.0
2,500.0
5,000.0
7,500.0
10,000.0
12,500.0
15,000.0
17,500.0
chr15_haplotype2-0000057 (kbp)

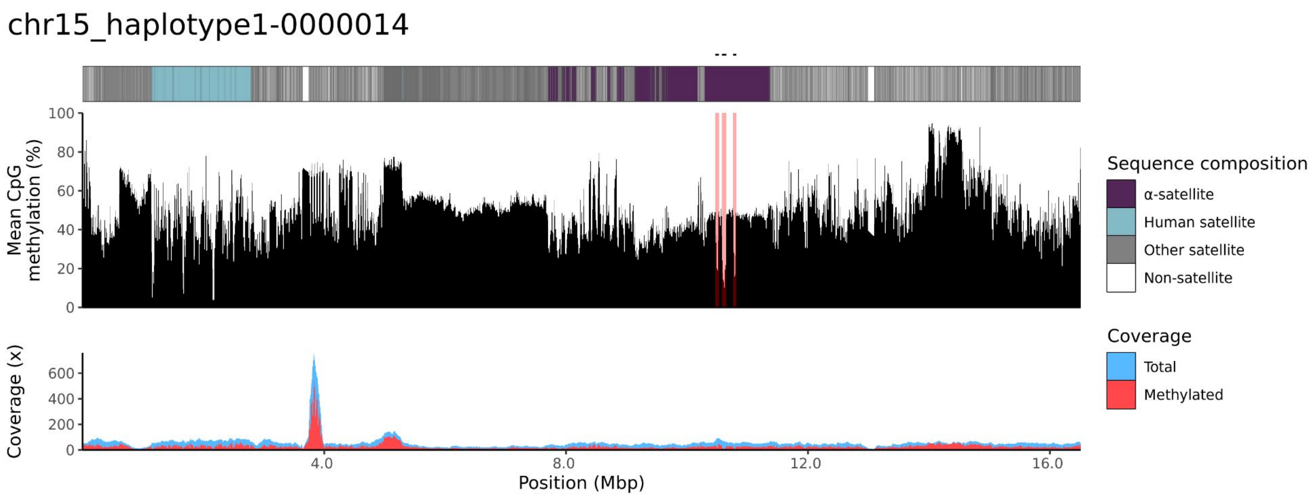

chr15_haplotype1-0000014
Mean CpG methylation (%)
100
80
60
40
20
0
Coverage (x)
600
400
200
0
4.0
8.0
12.0
16.0
Position (Mbp)
Sequence composition
α-satellite
Human satellite
Other satellite
Non-satellite
Coverage
Total
Methylated

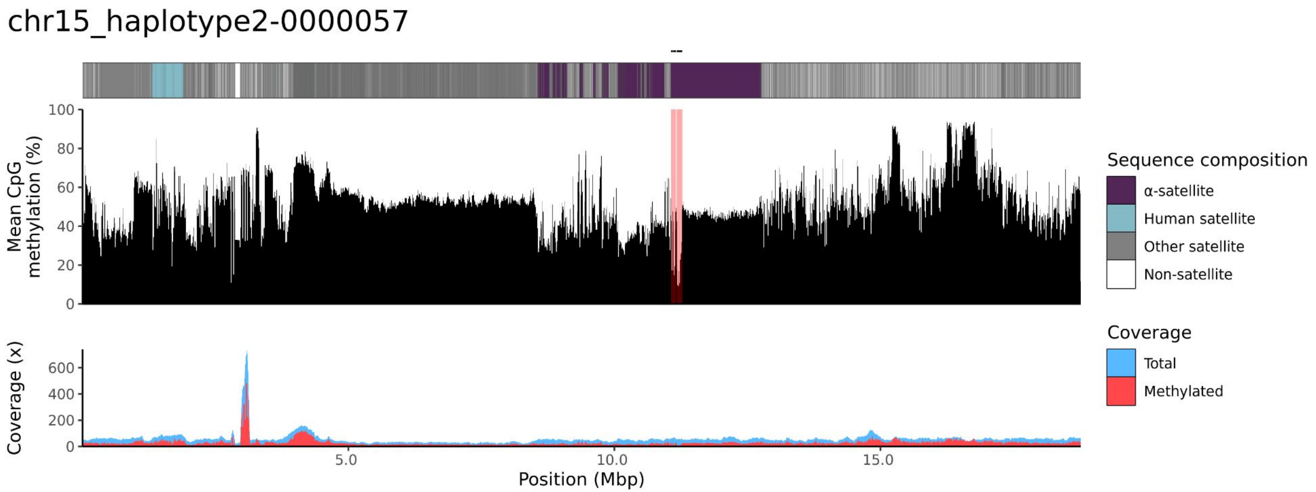

chr15_haplotype2-0000057
Mean CpG methylation (%)
100
80
60
40
20
0
Coverage (x)
600
400
200
0
5.0
10.0
15.0
Position (Mbp)
Sequence composition
α-satellite
Human satellite
Other satellite
Non-satellite
Coverage
Total
Methylated

chr21

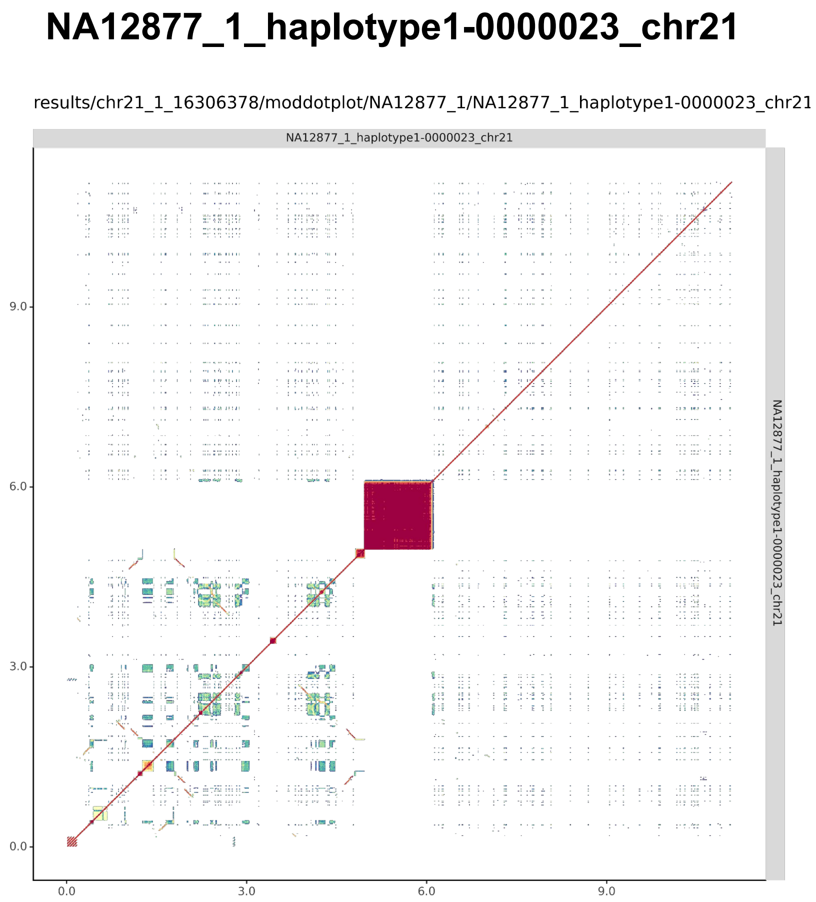
NA12877_1_haplotype1-0000023_chr21
results/chr21_1_16306378/moddotplot/NA12877_1/NA12877_1_haplotype1-0000023_chr21
NA12877_1_haplotype1-0000023_chr21
NA12877_1_haplotype1-0000023_chr21
0.0
3.0
6.0
9.0
0.0
3.0
6.0
9.0

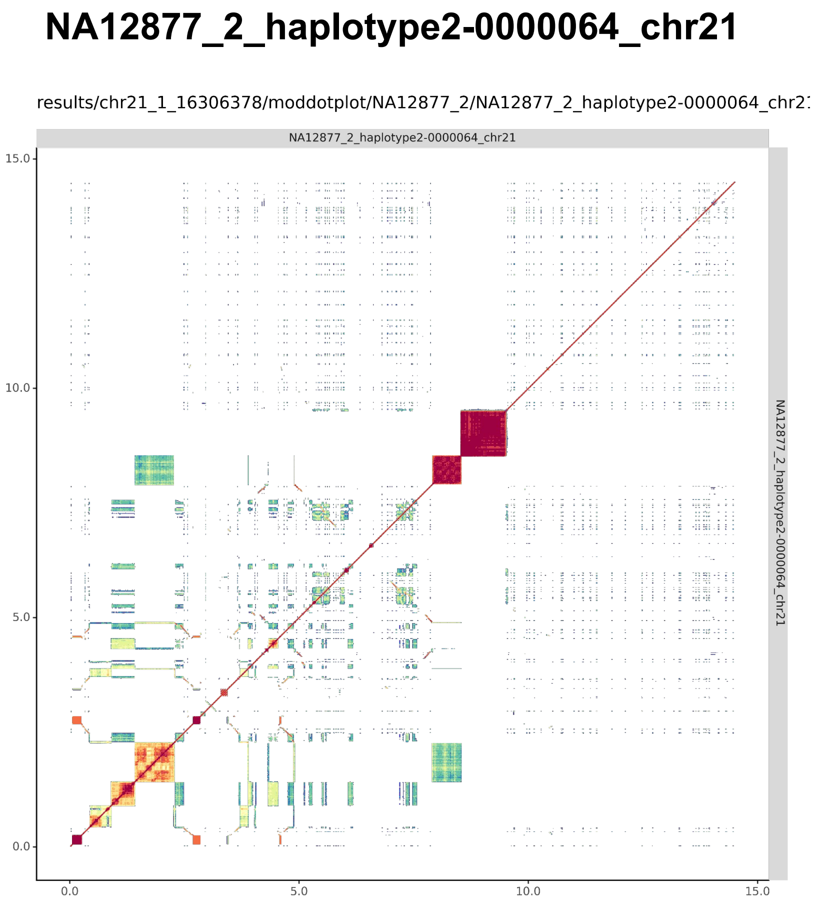
NA12877_2_haplotype2-0000064_chr21
results/chr21_1_16306378/moddotplot/NA12877_2/NA12877_2_haplotype2-0000064_chr2
NA12877_2_haplotype2-0000064_chr21
NA12877_2_haplotype2-0000064_chr21
0.0
5.0
10.0
15.0
0.0
5.0
10.0
15.0

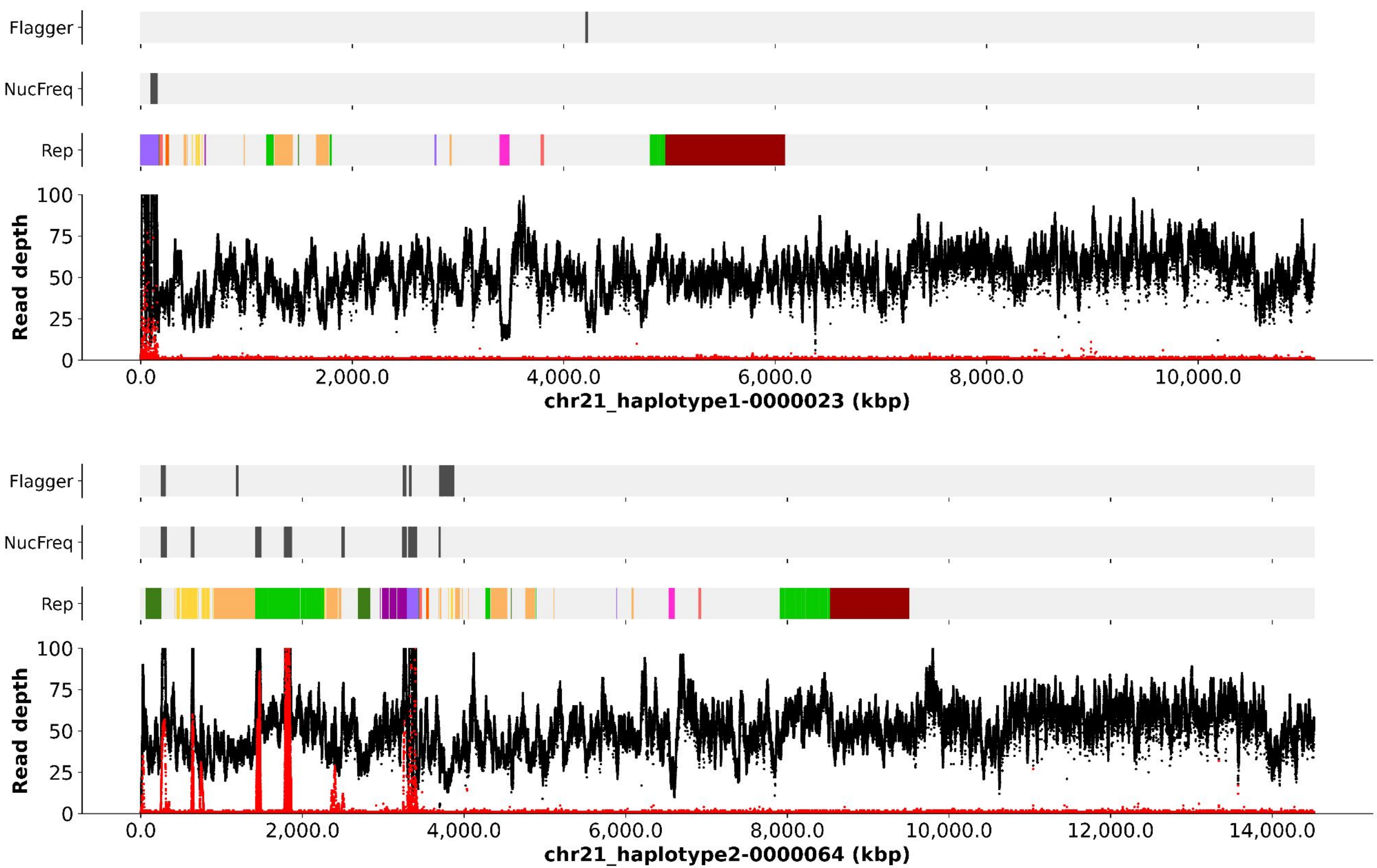
Flagger
NucFreq
Rep
Read depth
0
25
50
75
100
0.0
2,000.0
4,000.0
6,000.0
8,000.0
10,000.0
chr21_haplotype1-0000023 (kbp)
Flagger
NucFreq
Rep
Read depth
0
25
50
75
100
0.0
2,000.0
4,000.0
6,000.0
8,000.0
10,000.0
12,000.0
14,000.0
chr21_haplotype2-0000064 (kbp)

chr21_haplotype1-0000023

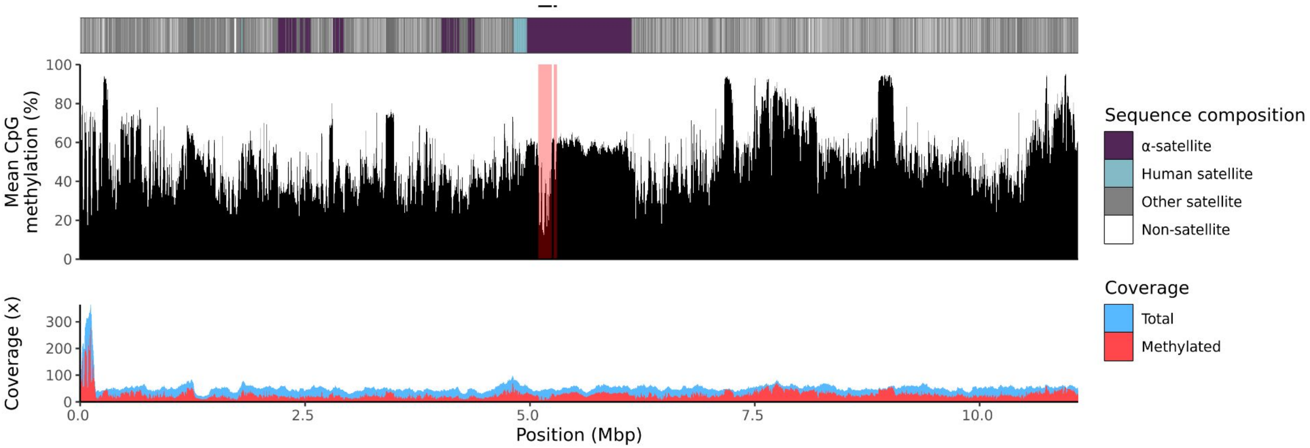

chr21_haplotype2-0000064

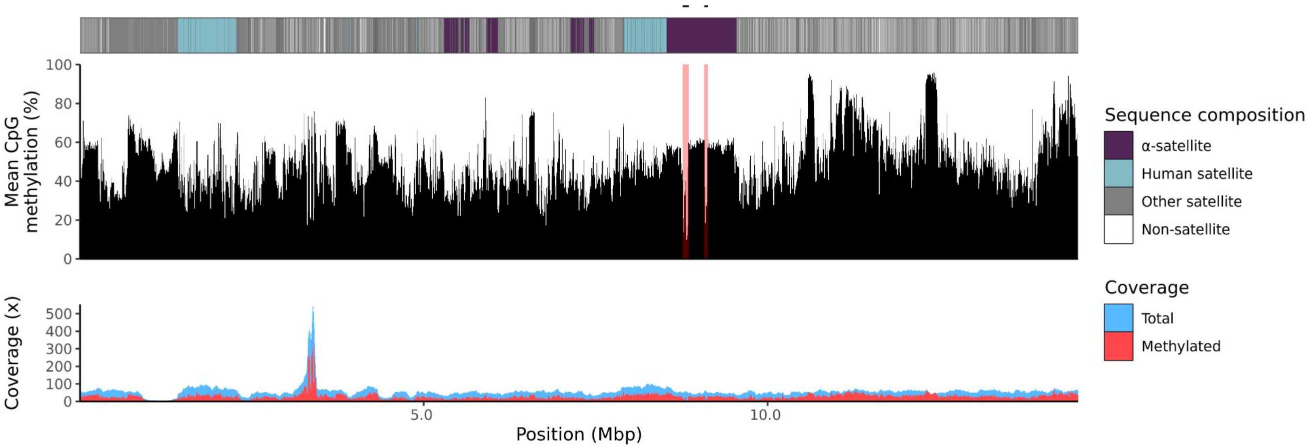

chr22

NA12877_1_haplotype1-0000020_chr22

results/chr22_1_20711065/moddotplot/NA12877_1/NA12877_1_haplotype1-0000020_chr22

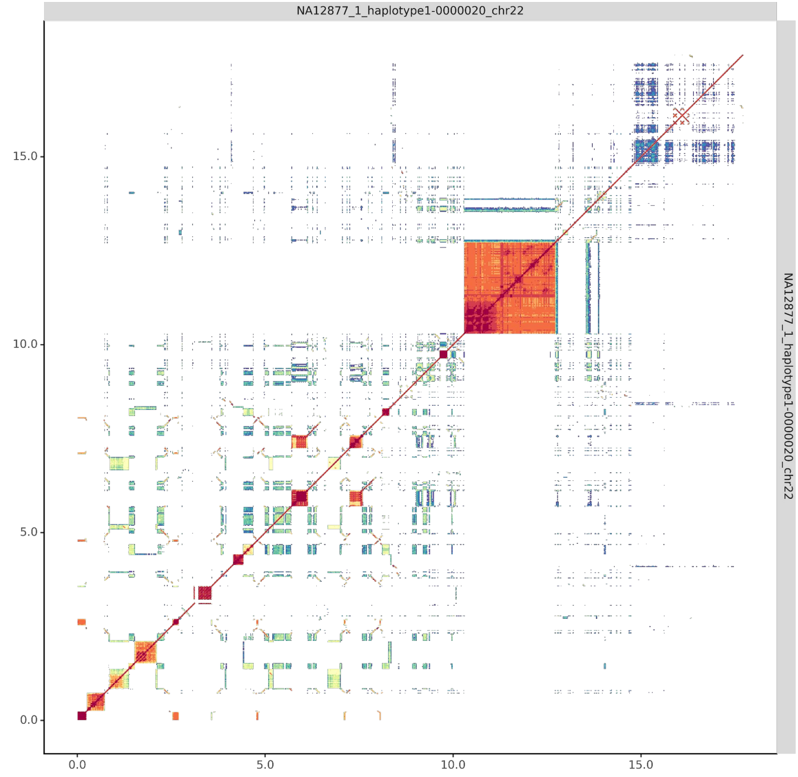

NA12877_2_haplotype2-0000070_chr22

results/chr22_1_20711065/moddotplot/NA12877_2/NA12877_2_haplotype2-0000070_chr22

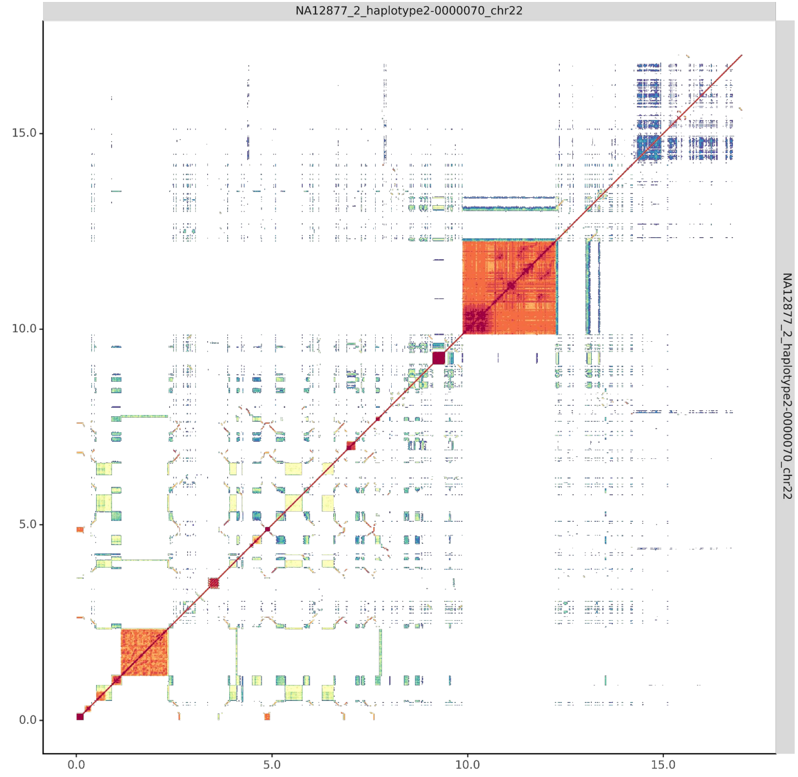

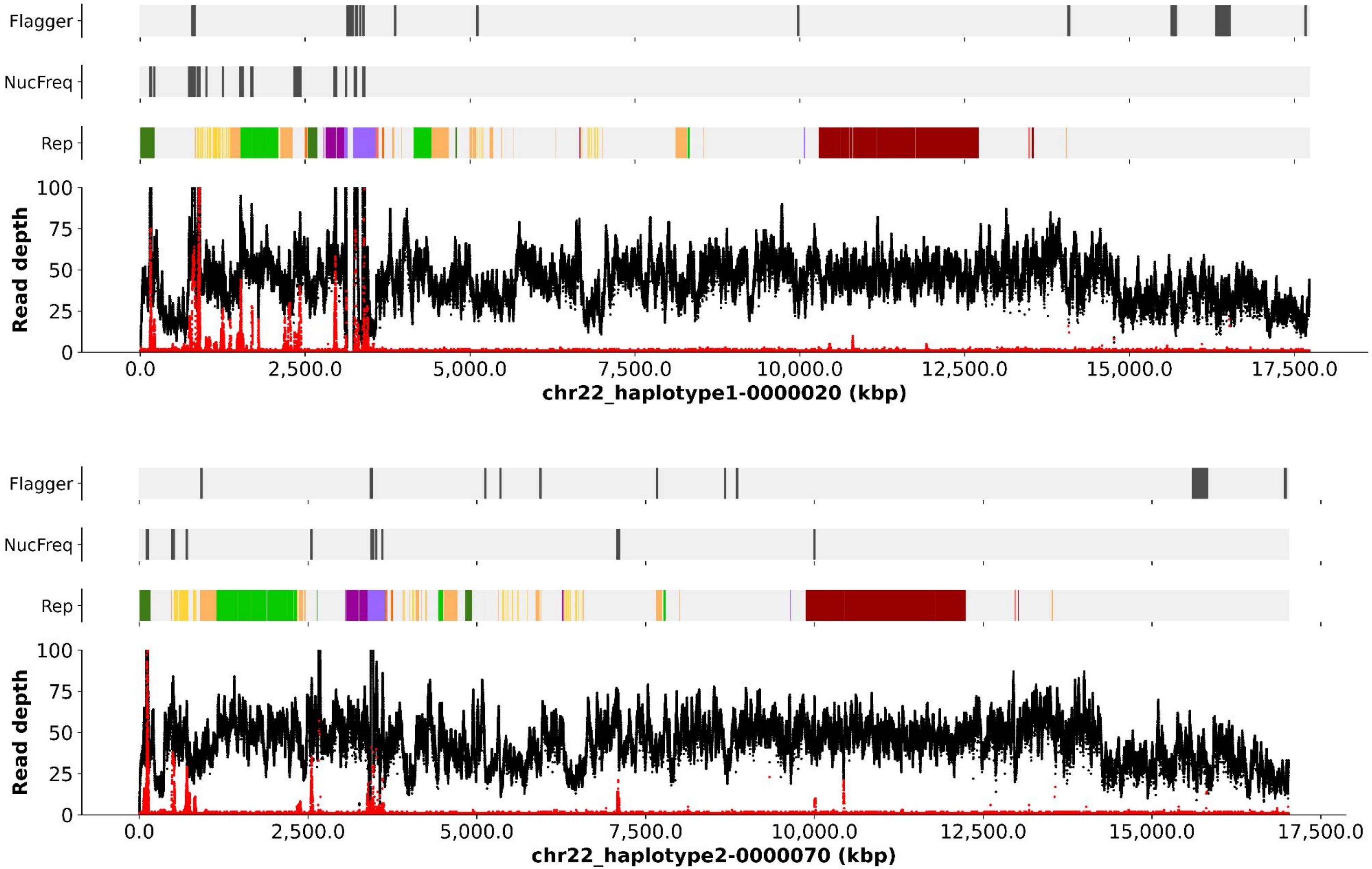

Flagger
NucFreq
Rep
Read depth
100
75
50
25
0
0.0
2,500.0
5,000.0
7,500.0
10,000.0
12,500.0
15,000.0
17,500.0
chr22_haplotype1-0000020 (kbp)
Flagger
NucFreq
Rep
Read depth
100
75
50
25
0
0.0
2,500.0
5,000.0
7,500.0
10,000.0
12,500.0
15,000.0
17,500.0
chr22_haplotype2-0000070 (kbp)

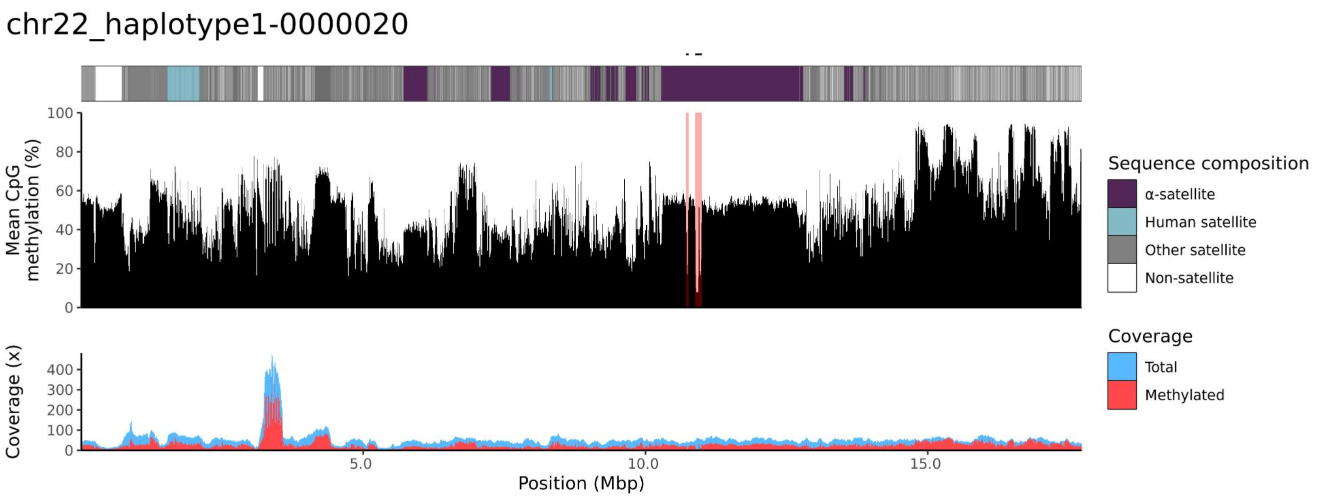
chr22_haplotype1-0000020
Sequence composition
α-satellite
Human satellite
Other satellite
Non-satellite
Coverage
Total
Methylated
Mean CpG methylation (%)
100
80
60
40
20
0
Coverage (x)
400
300
200
100
0
5.0
10.0
15.0
Position (Mbp)

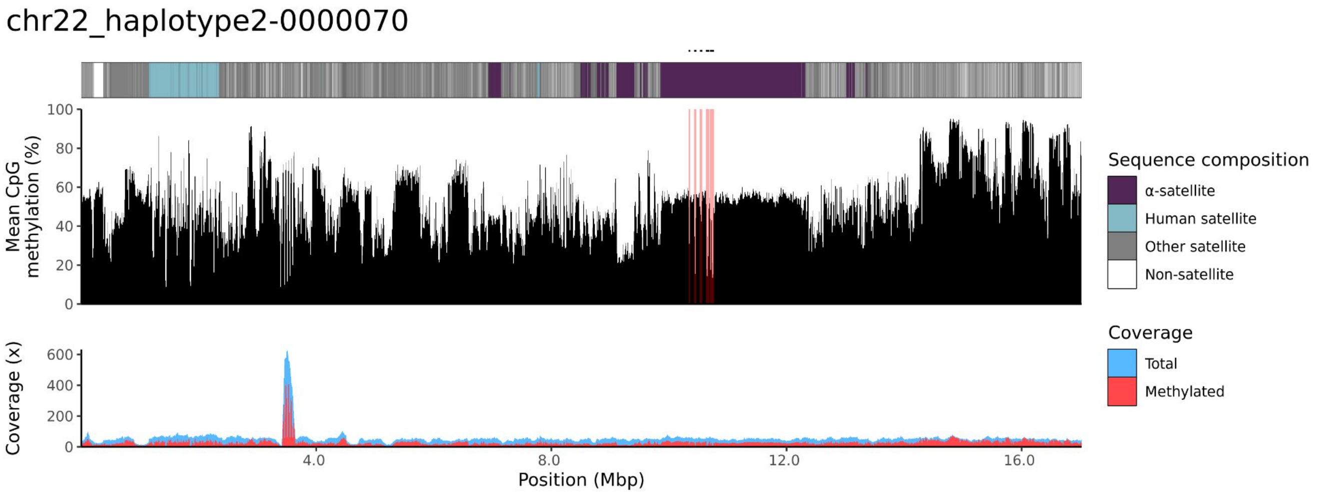
chr22_haplotype2-0000070
Sequence composition
α-satellite
Human satellite
Other satellite
Non-satellite
Coverage
Total
Methylated
Mean CpG methylation (%)
100
80
60
40
20
0
Coverage (x)
600
400
200
0
4.0
8.0
12.0
16.0
Position (Mbp)

NA12878

chr13

NA12878_1_haplotype1-0000005_chr13

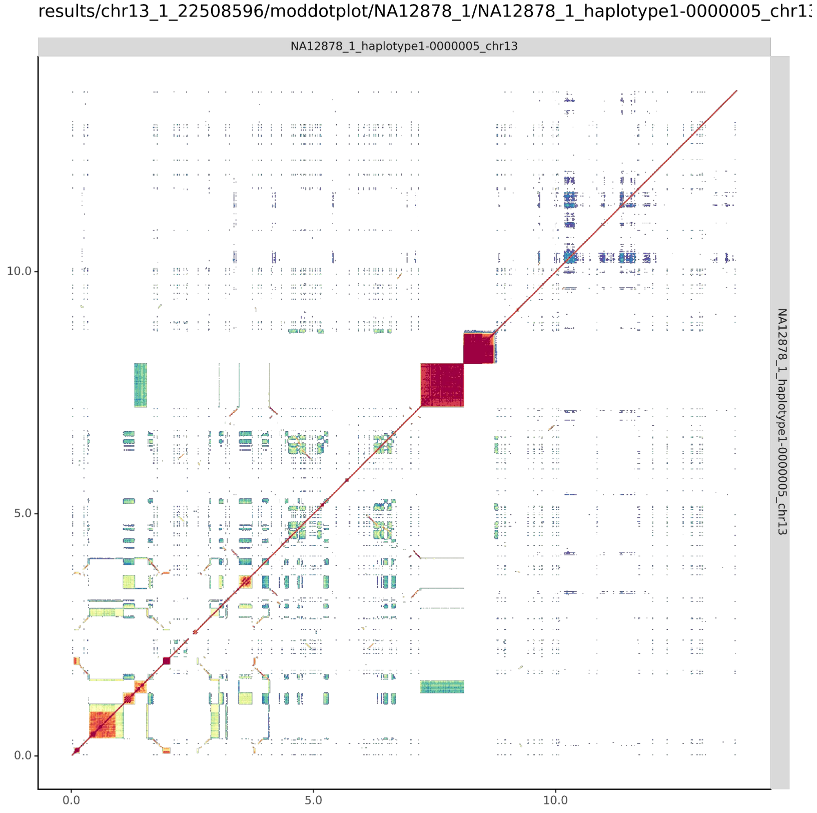

NA12878_2_haplotype2-0000030_chr13

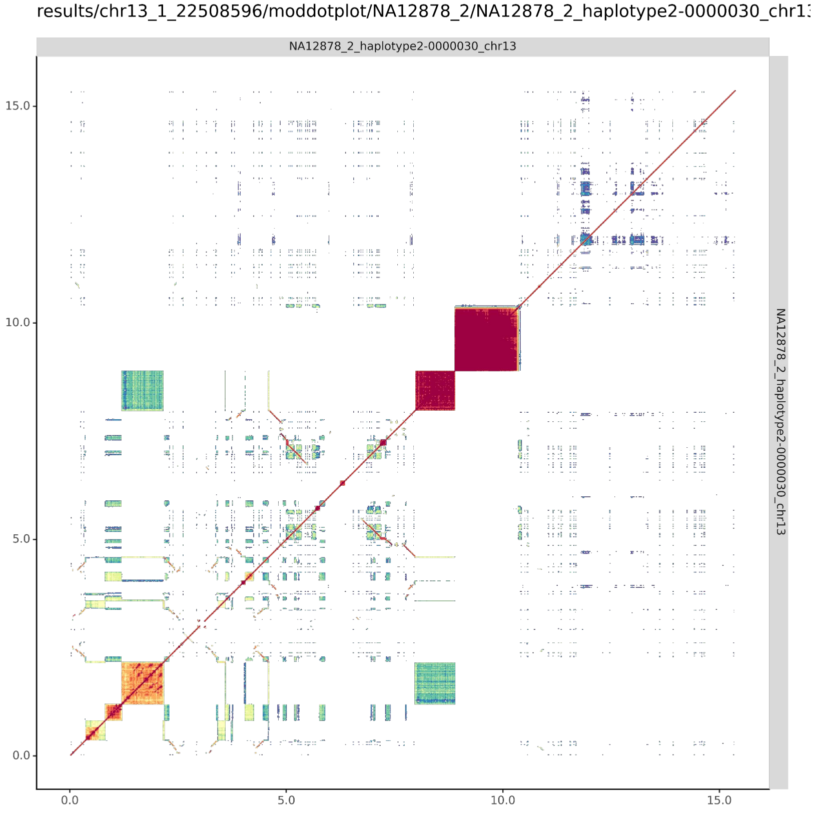

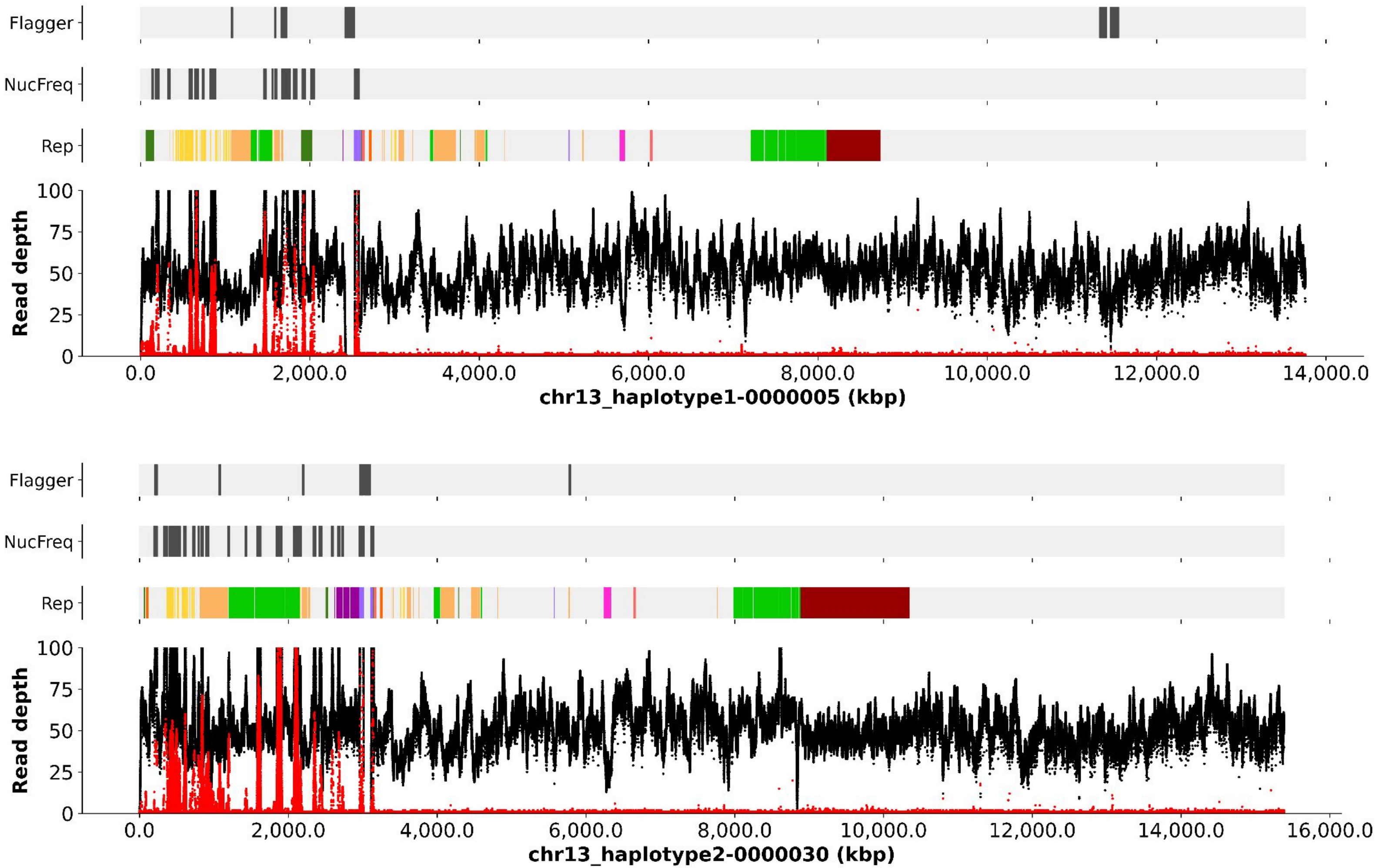

Flagger
NucFreq
Rep
Read depth
100
75
50
25
0
0.0
2,000.0
4,000.0
6,000.0
8,000.0
10,000.0
12,000.0
14,000.0
chr13_haplotype1-0000005 (kbp)
Flagger
NucFreq
Rep
Read depth
100
75
50
25
0
0.0
2,000.0
4,000.0
6,000.0
8,000.0
10,000.0
12,000.0
14,000.0
16,000.0
chr13_haplotype2-0000030 (kbp)

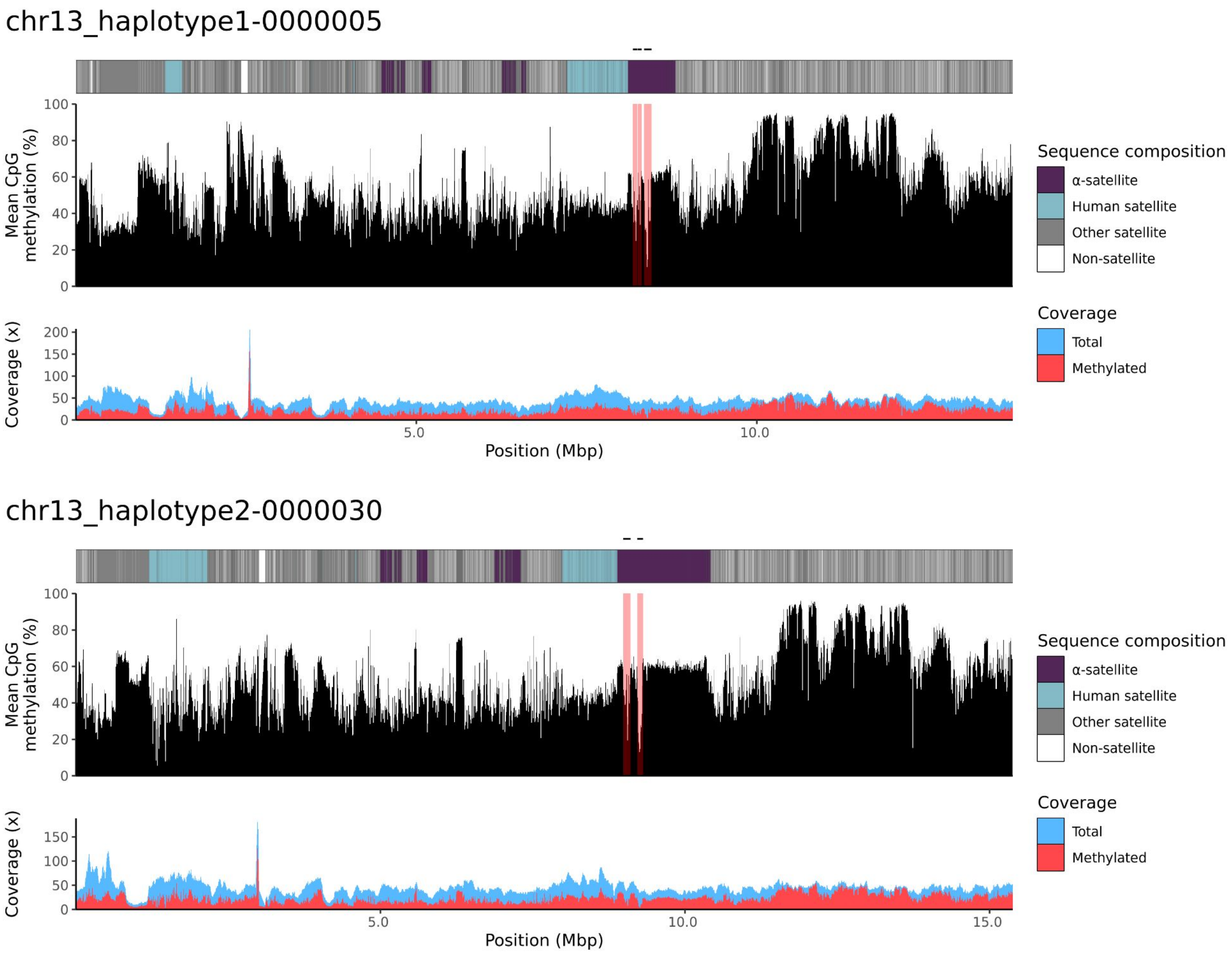

chr13_haplotype1-0000005
Mean CpG methylation (%)
Coverage (x)
Position (Mbp)
5.0
10.0
Sequence composition
α-satellite
Human satellite
Other satellite
Non-satellite
Coverage
Total
Methylated
chr13_haplotype2-0000030
Mean CpG methylation (%)
Coverage (x)
Position (Mbp)
5.0
10.0
15.0
Sequence composition
α-satellite
Human satellite
Other satellite
Non-satellite
Coverage
Total
Methylated

chr14

NA12878_1_haplotype1-0000013_chr14

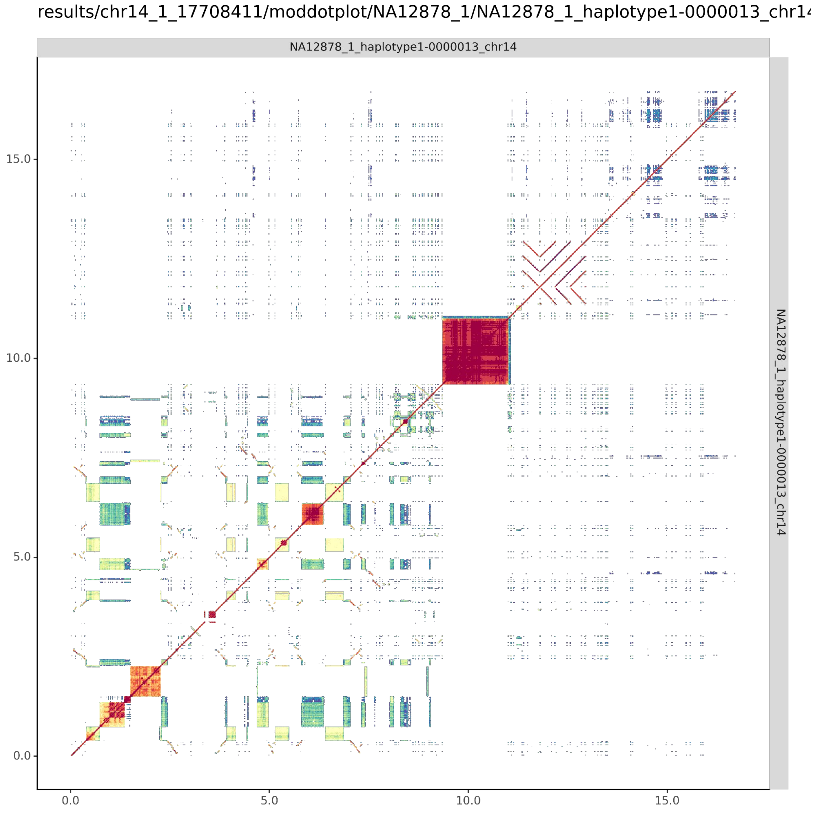

NA12878_2_haplotype2-0000041_chr14

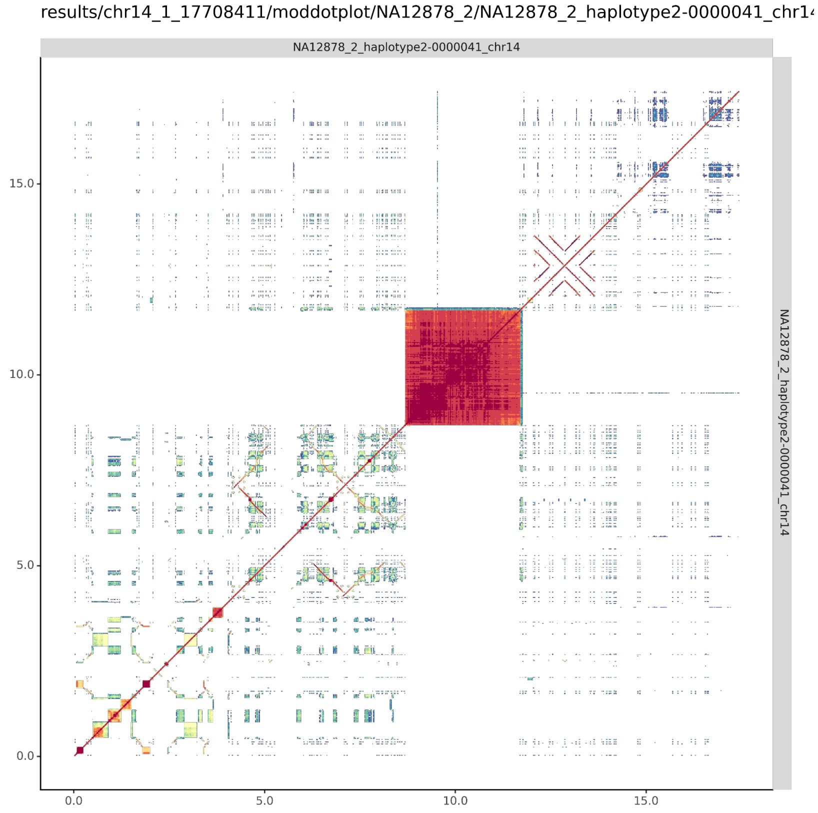

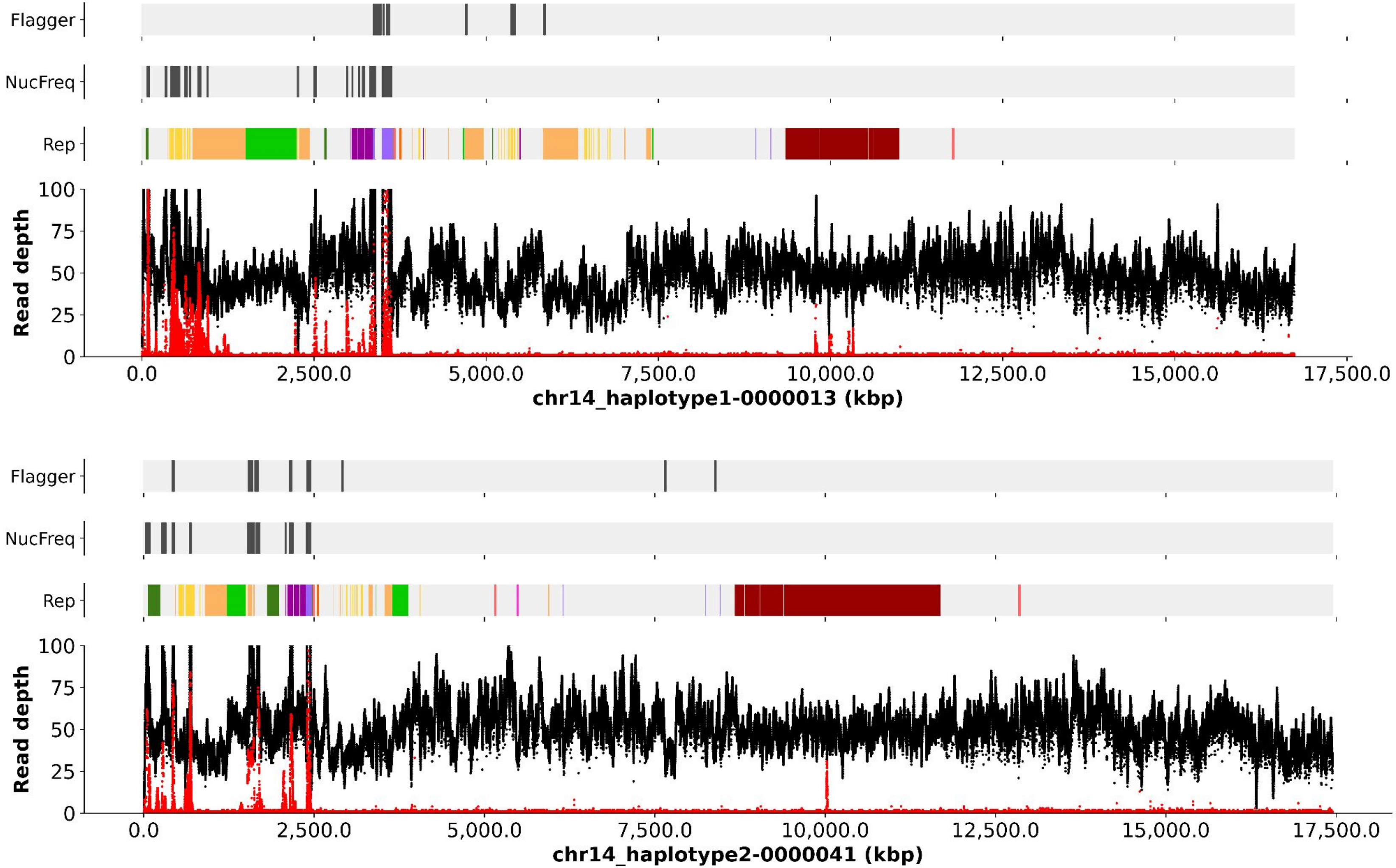
Flagger
NucFreq
Rep
Read depth
100
75
50
25
0
0.0
2,500.0
5,000.0
7,500.0
10,000.0
12,500.0
15,000.0
17,500.0
chr14_haplotype1-0000013 (kbp)
Flagger
NucFreq
Rep
Read depth
chr14_haplotype2-0000041 (kbp)

chr14_haplotype1-0000013

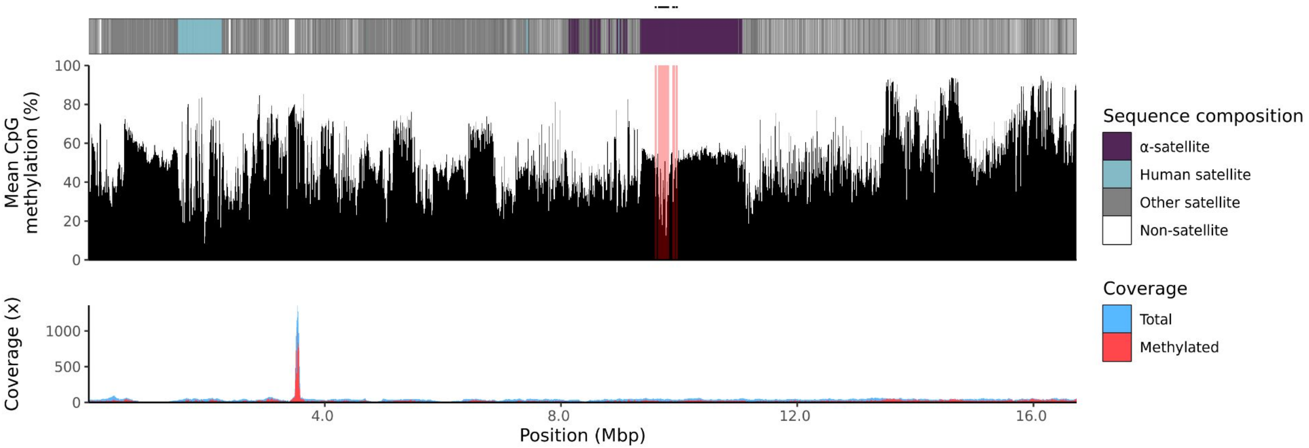

chr14_haplotype2-0000041

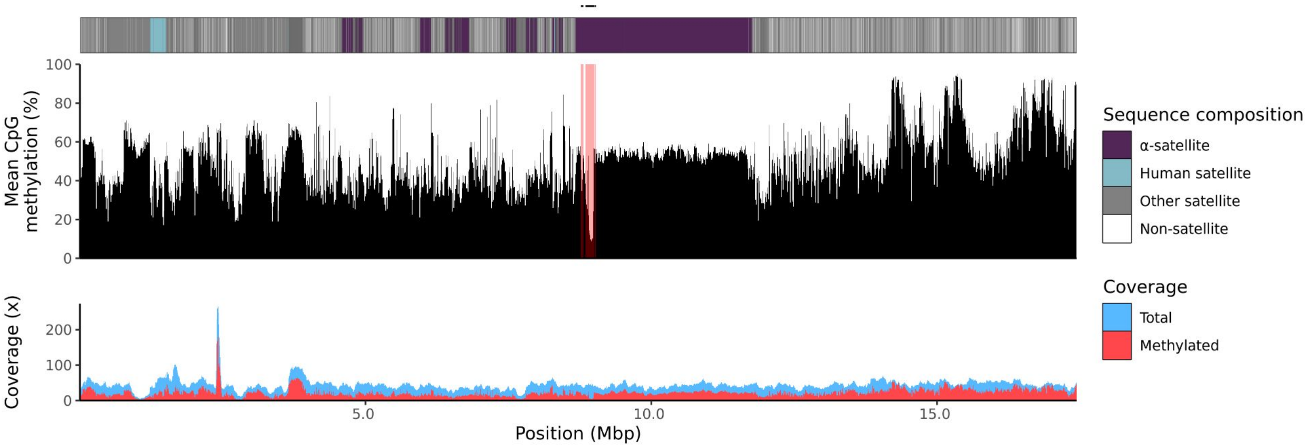

chr15

NA12878_1_haplotype1-0000007_chr15

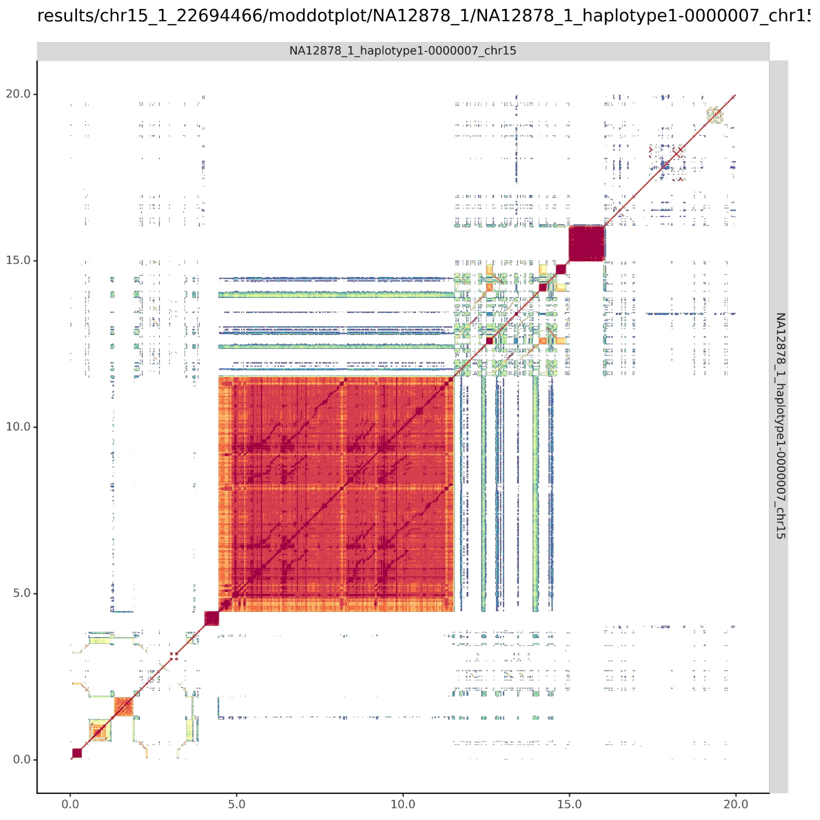

NA12878_2_haplotype2-0000044_chr15

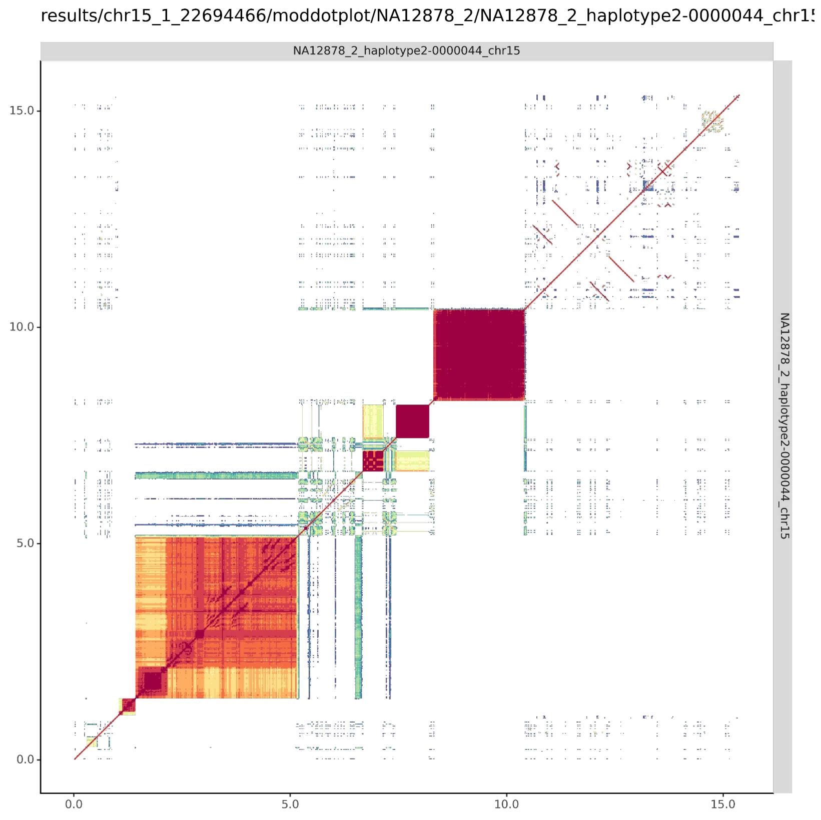

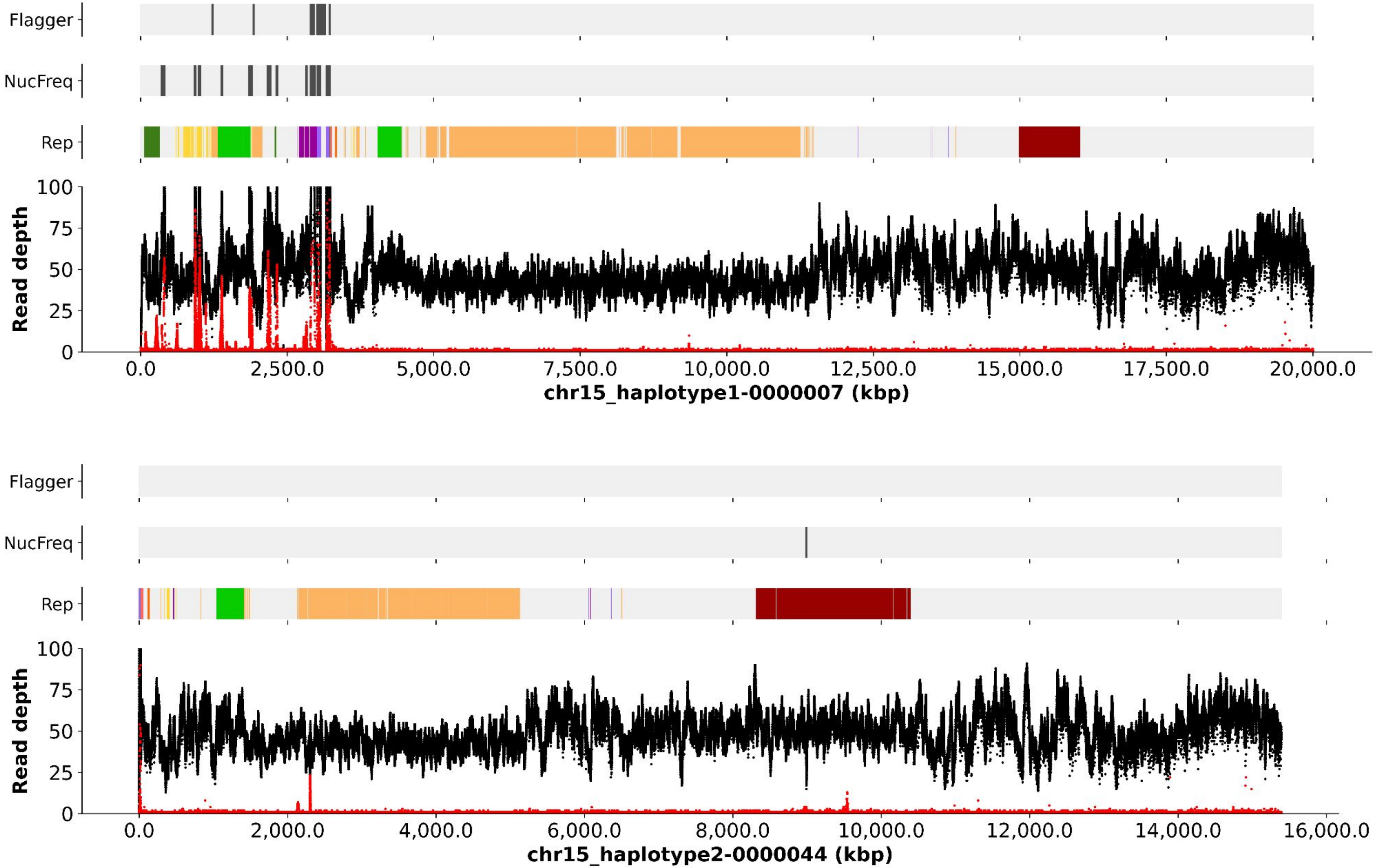

Flagger
NucFreq
Rep
Read depth
100
75
50
25
0
0.0
2,500.0
5,000.0
7,500.0
10,000.0
12,500.0
15,000.0
17,500.0
20,000.0
chr15_haplotype1-0000007 (kbp)
Flagger
NucFreq
Rep
Read depth
100
75
50
25
0
0.0
2,000.0
4,000.0
6,000.0
8,000.0
10,000.0
12,000.0
14,000.0
16,000.0
chr15_haplotype2-0000044 (kbp)

chr15_haplotype1-0000007

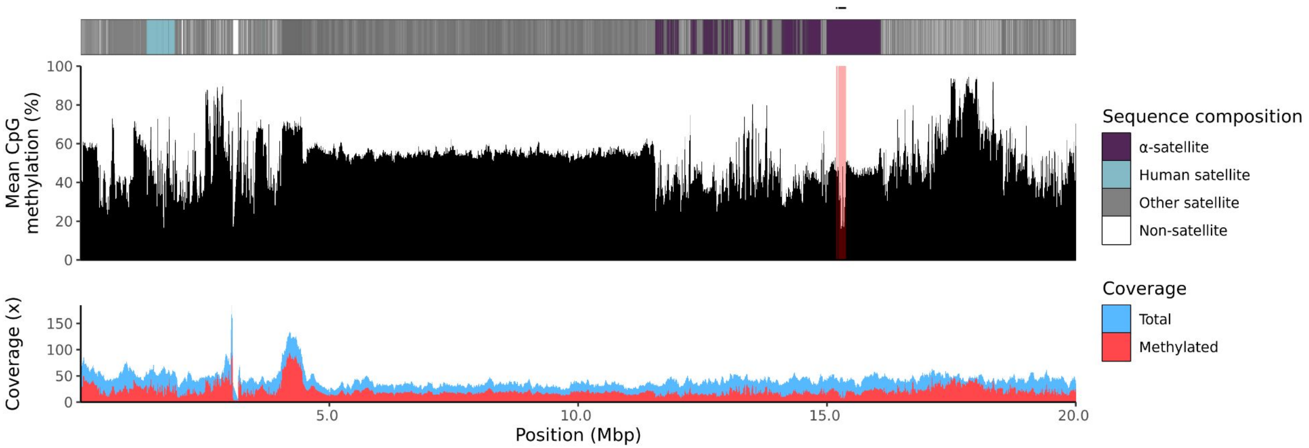

chr15_haplotype2-0000044

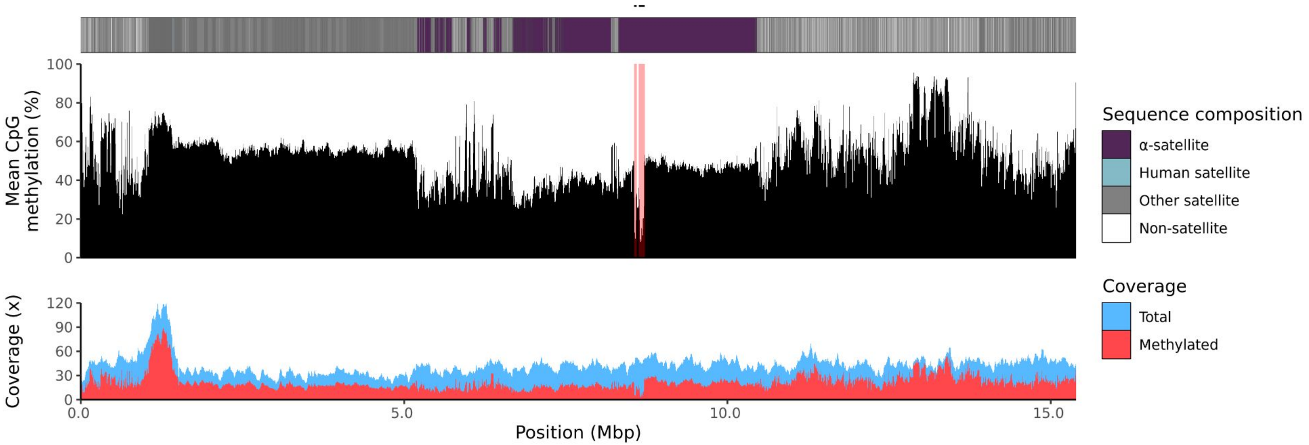

chr21

NA12878_1_haplotype1-0000009_chr21

results/chr21_1_16306378/moddotplot/NA12878_1/NA12878_1_haplotype1-0000009_chr2[illegible]

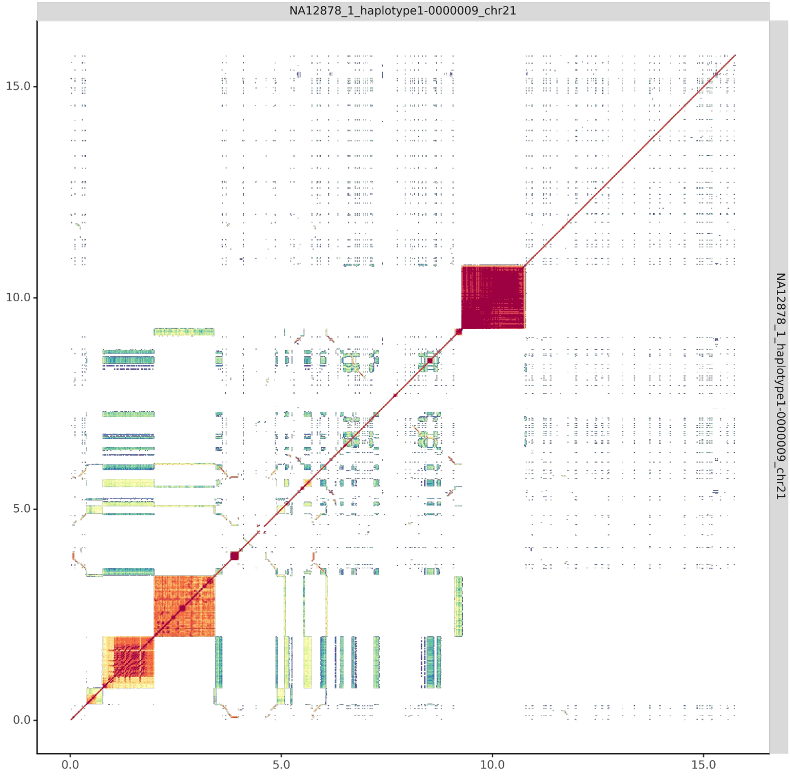

NA12878_2_haplotype2-0000032_chr21

results/chr21_1_16306378/moddotplot/NA12878_2/NA12878_2_haplotype2-0000032_chr2[illegible]

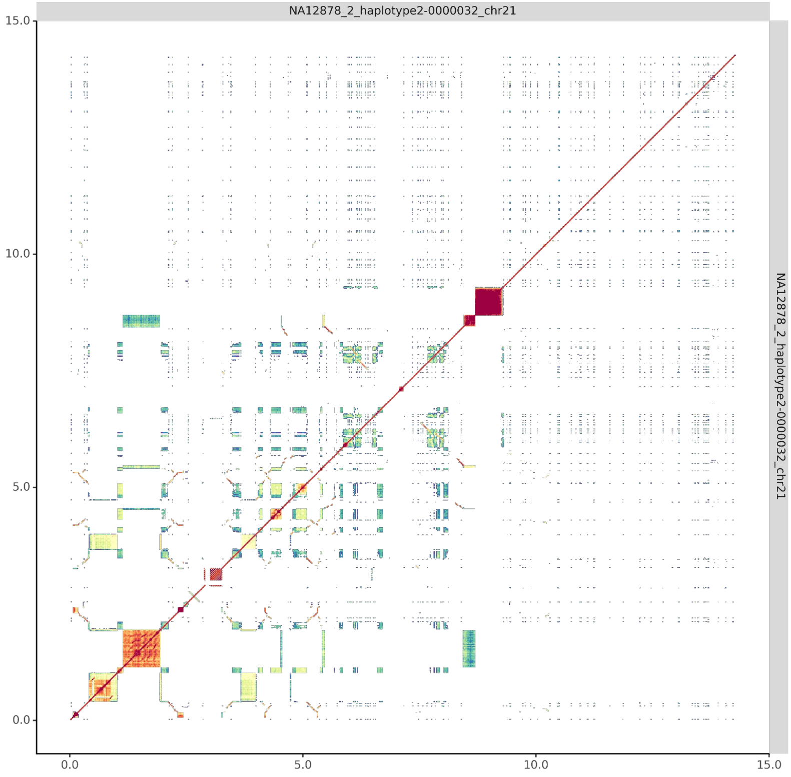

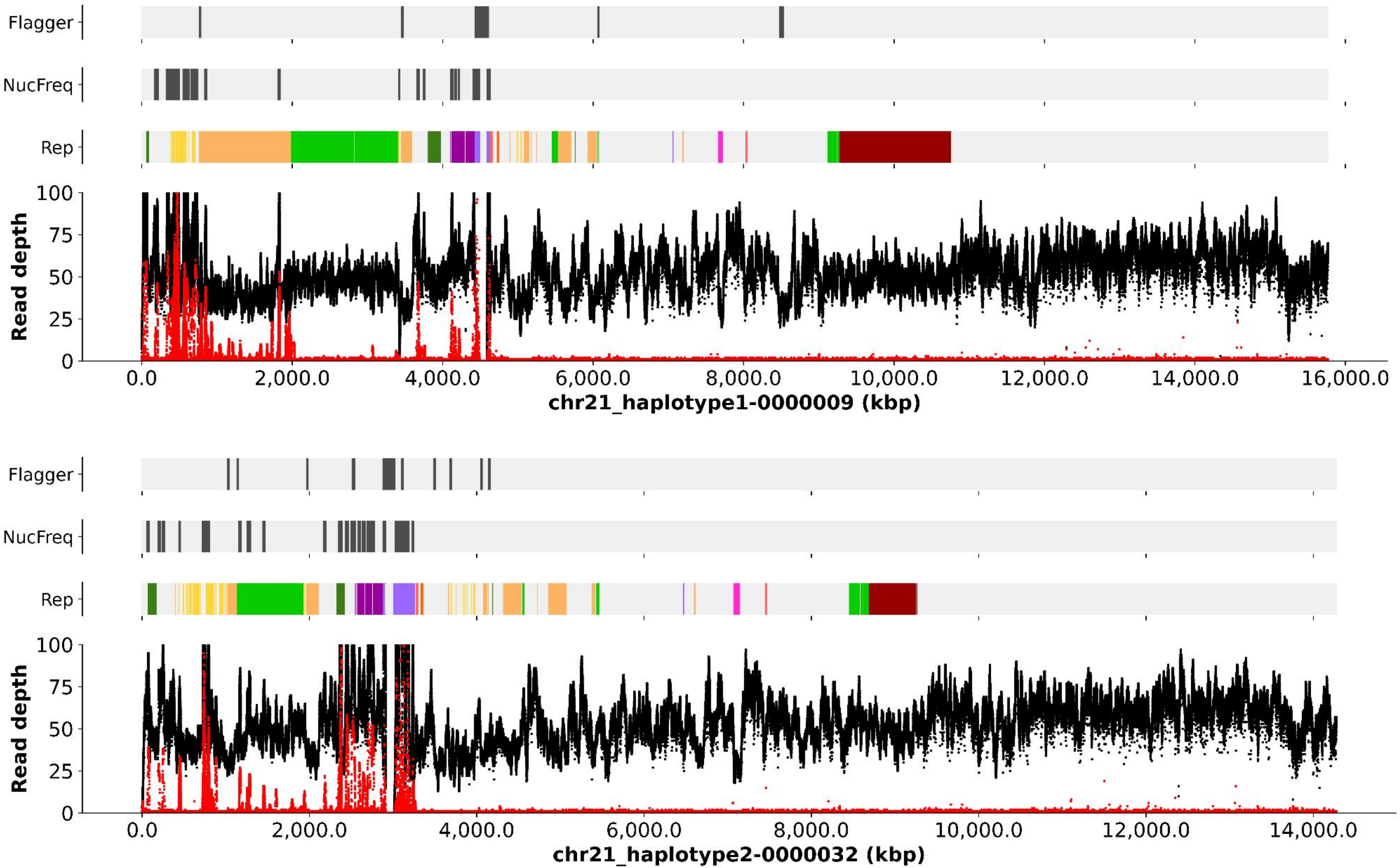

Flagger
NucFreq
Rep
Read depth
0
25
50
75
100
0.0
2,000.0
4,000.0
6,000.0
8,000.0
10,000.0
12,000.0
14,000.0
16,000.0
chr21_haplotype1-0000009 (kbp)
Flagger
NucFreq
Rep
Read depth
0
25
50
75
100
0.0
2,000.0
4,000.0
6,000.0
8,000.0
10,000.0
12,000.0
14,000.0
chr21_haplotype2-0000032 (kbp)

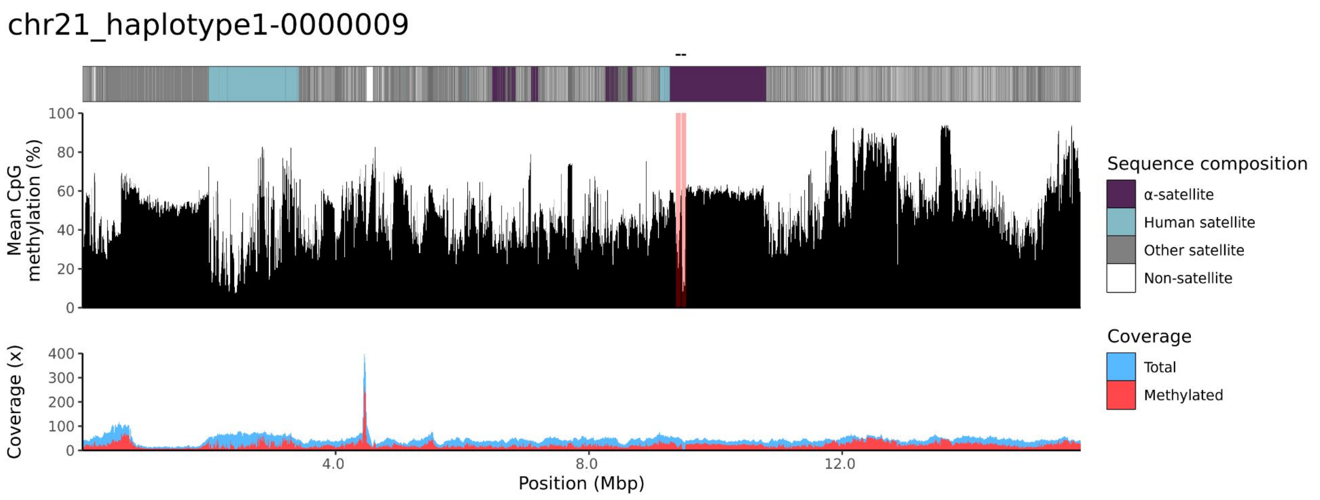
chr21_haplotype1-0000009
Mean CpG methylation (%)
100
80
60
40
20
0
Coverage (x)
400
300
200
100
0
4.0
8.0
12.0
Position (Mbp)
Sequence composition
α-satellite
Human satellite
Other satellite
Non-satellite
Coverage
Total
Methylated

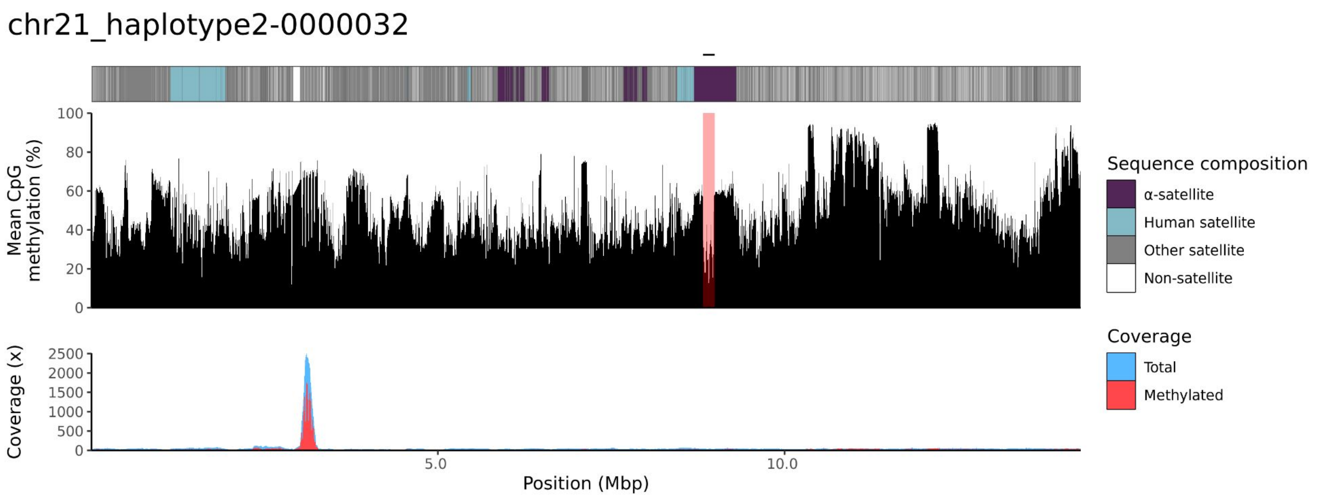
chr21_haplotype2-0000032
Mean CpG methylation (%)
100
80
60
40
20
0
Coverage (x)
2500
2000
1500
1000
500
0
5.0
10.0
Position (Mbp)
Sequence composition
α-satellite
Human satellite
Other satellite
Non-satellite
Coverage
Total
Methylated

chr22

NA12878_1_haplotype1-0000008_chr22

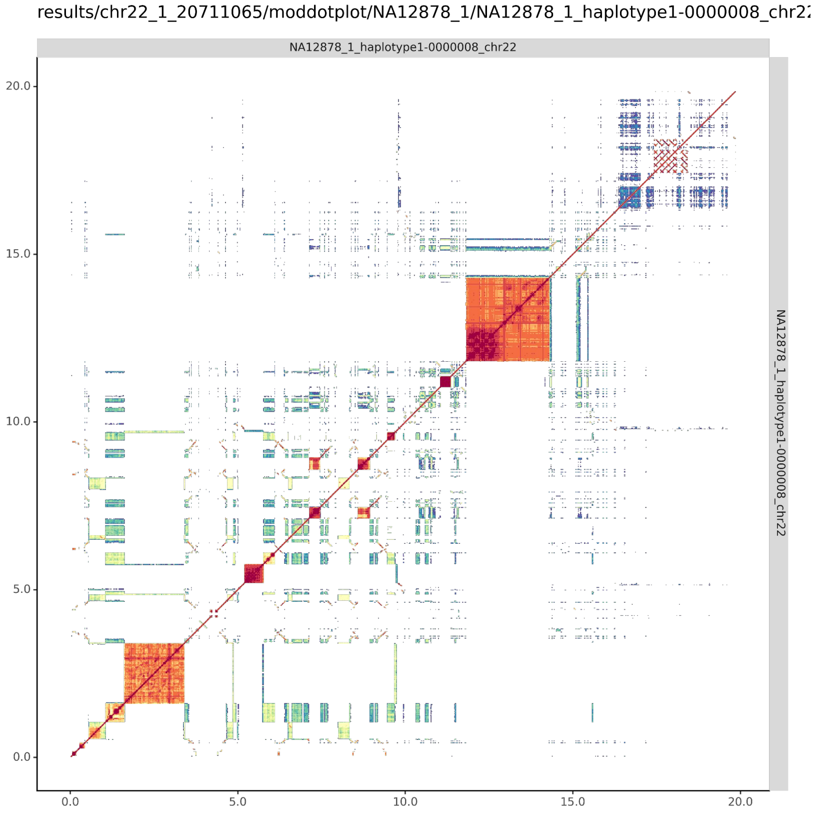

NA12878_2_haplotype2-0000037_chr22

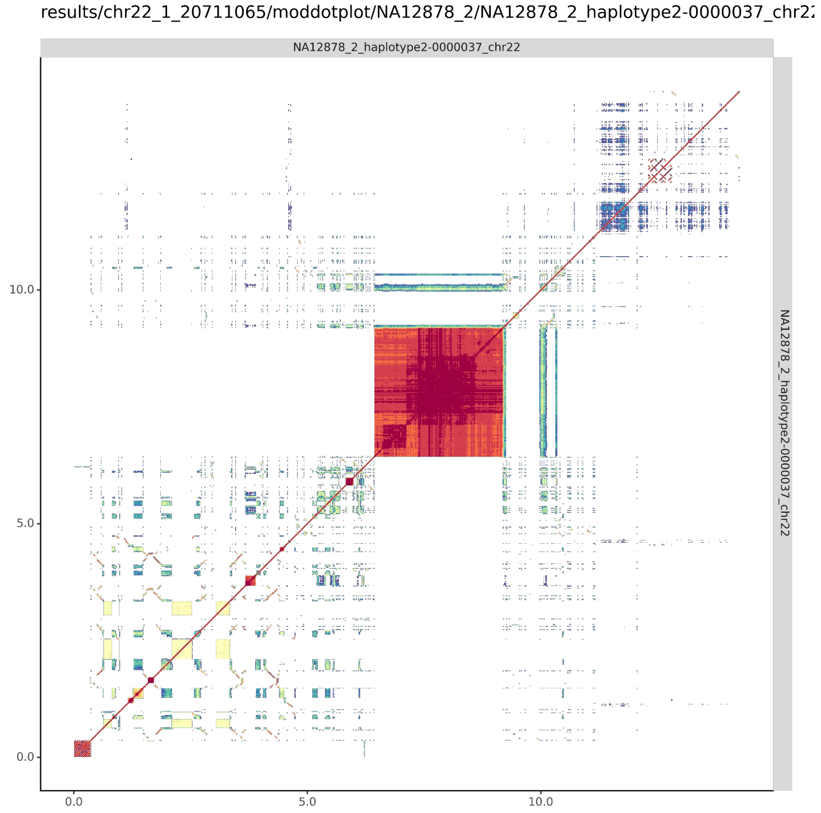

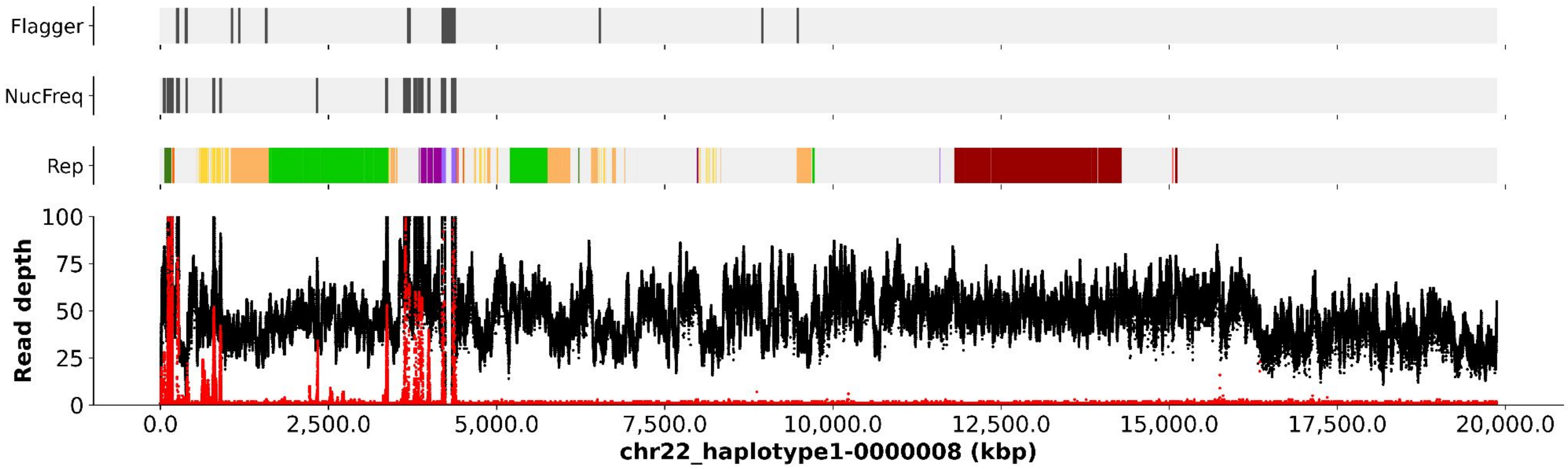
Flagger
NucFreq
Rep
Read depth
100
75
50
25
0
0.0
2,500.0
5,000.0
7,500.0
10,000.0
12,500.0
15,000.0
17,500.0
20,000.0
chr22_haplotype1-0000008 (kbp)

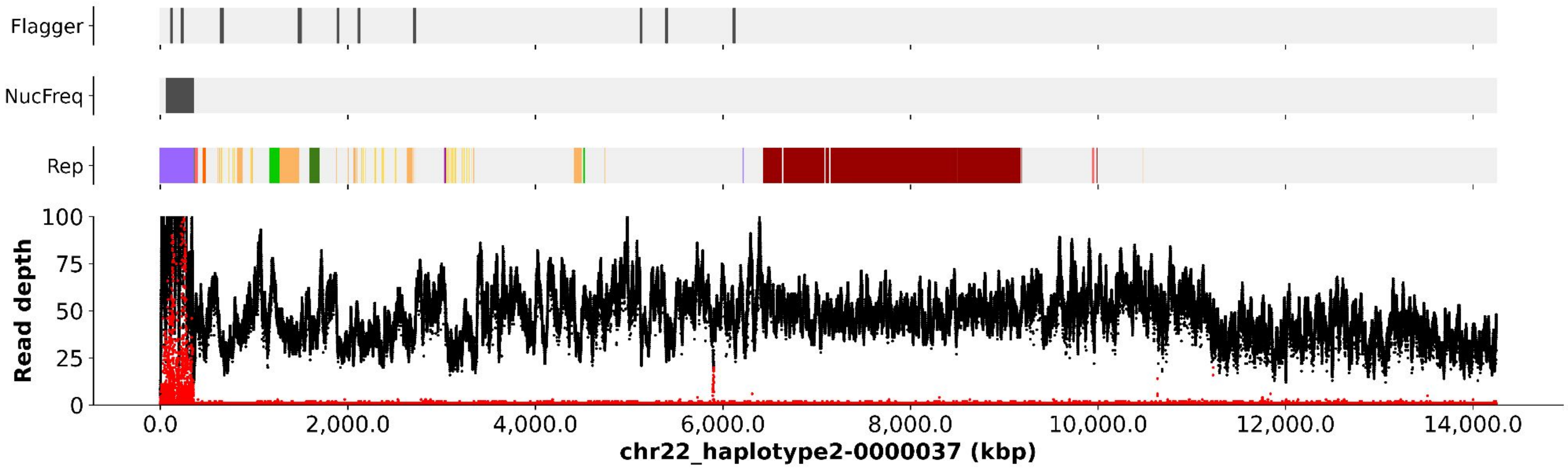
Flagger
NucFreq
Rep
Read depth
100
75
50
25
0
0.0
2,000.0
4,000.0
6,000.0
8,000.0
10,000.0
12,000.0
14,000.0
chr22_haplotype2-0000037 (kbp)

chr22_haplotype1-0000008

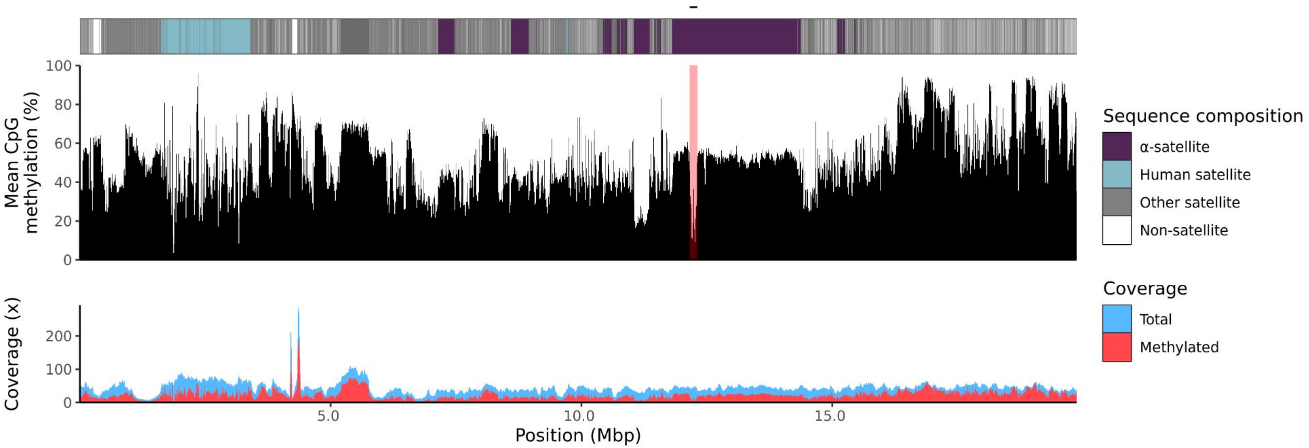

chr22_haplotype2-0000037

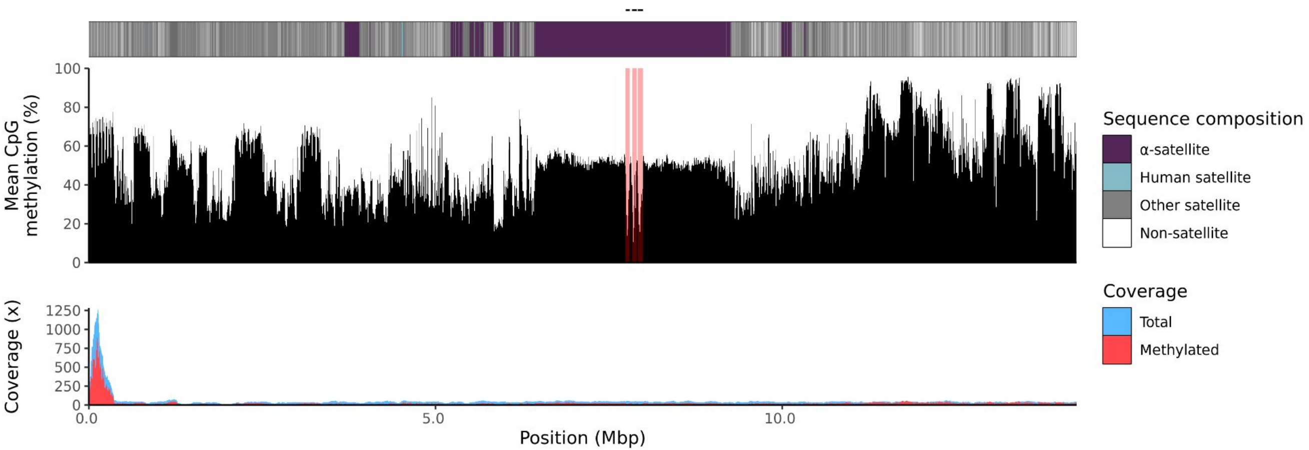

NA12879

chr13

NA12879_1_haplotype1-0000002_chr13

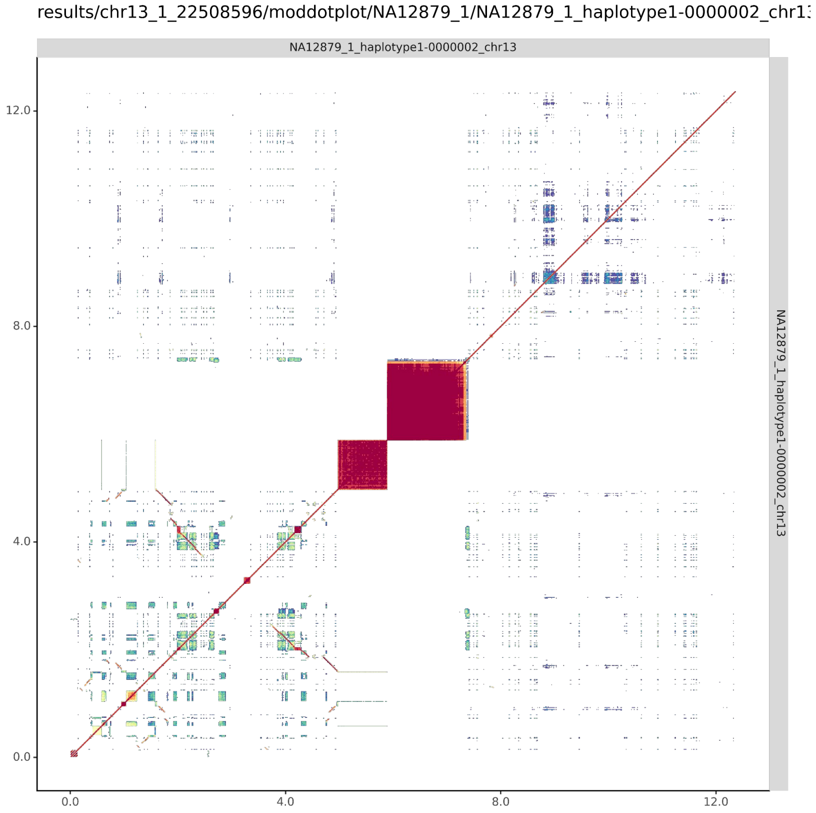

NA12879_2_haplotype2-0000092_chr13

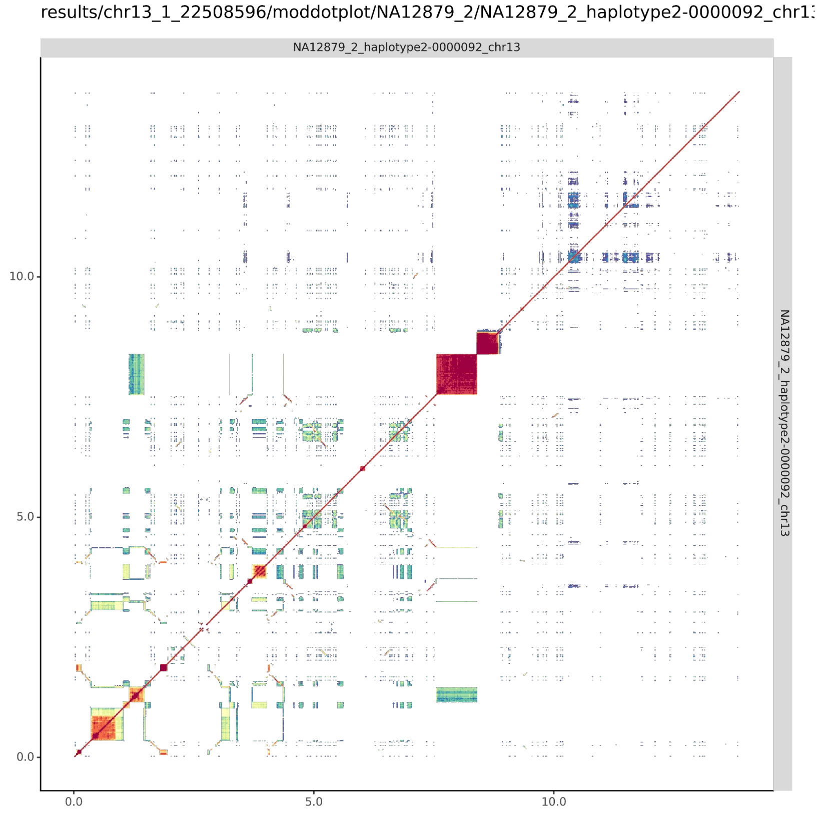

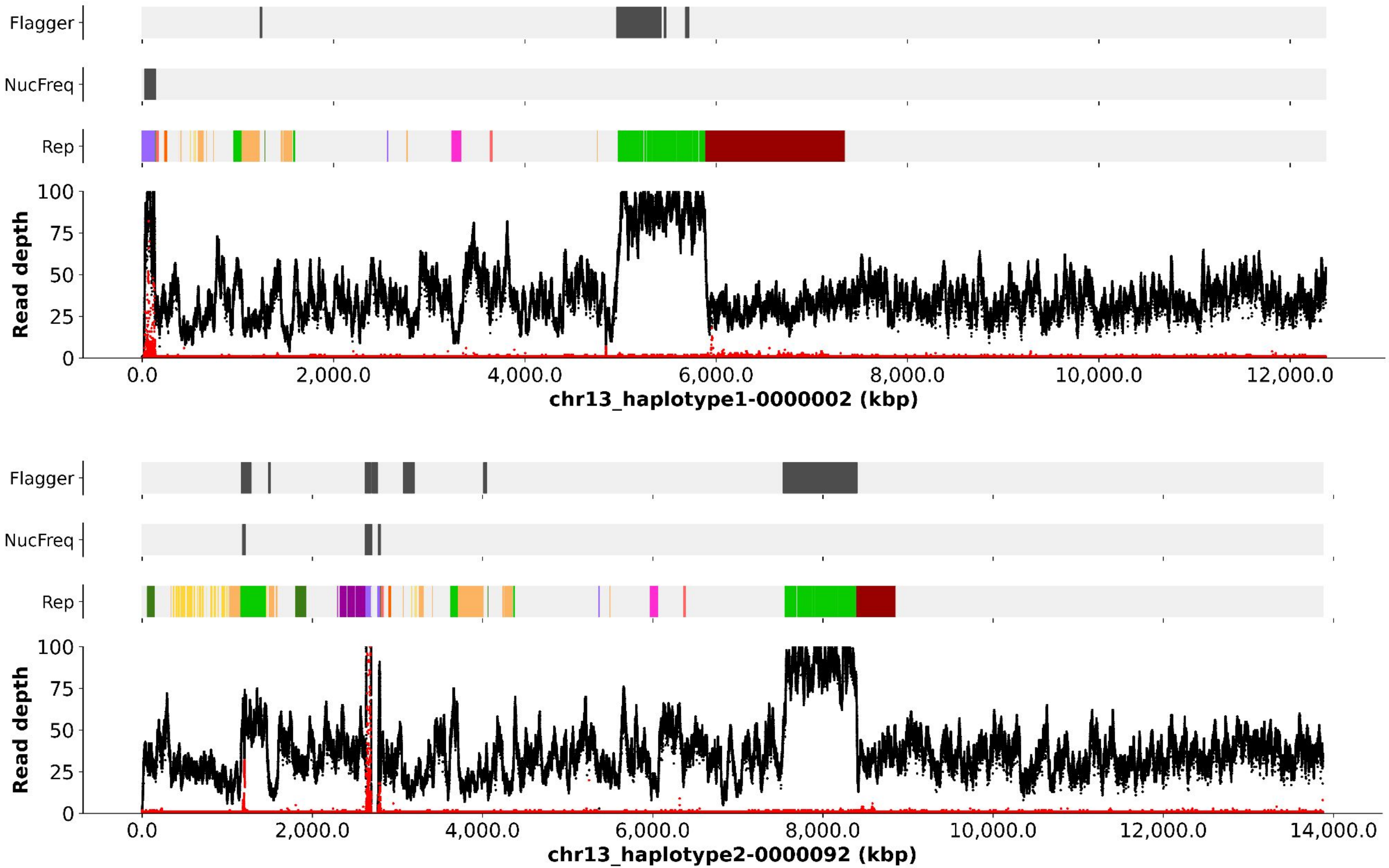

Flagger
NucFreq
Rep
Read depth
100
75
50
25
0
0.0
2,000.0
4,000.0
6,000.0
8,000.0
10,000.0
12,000.0
chr13_haplotype1-0000002 (kbp)
Flagger
NucFreq
Rep
Read depth
100
75
50
25
0
0.0
2,000.0
4,000.0
6,000.0
8,000.0
10,000.0
12,000.0
14,000.0
chr13_haplotype2-0000092 (kbp)

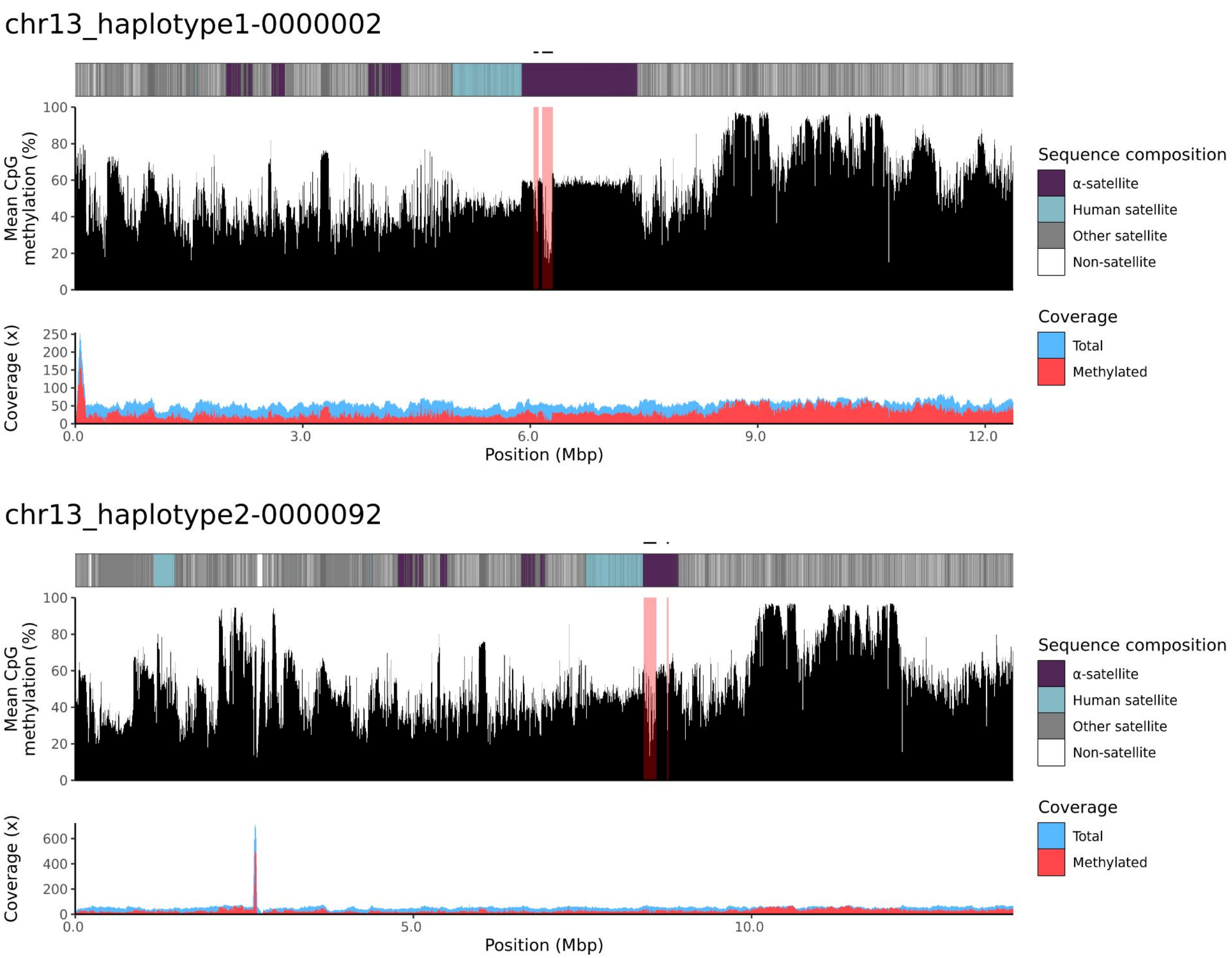
chr13_haplotype1-0000002
Mean CpG methylation (%)
Coverage (x)
Position (Mbp)
Sequence composition
α-satellite
Human satellite
Other satellite
Non-satellite
Coverage
Total
Methylated
chr13_haplotype2-0000092
Mean CpG methylation (%)
Coverage (x)
Position (Mbp)
Sequence composition
α-satellite
Human satellite
Other satellite
Non-satellite
Coverage
Total
Methylated

chr14

NA12879_1_haplotype1-0000015_chr14

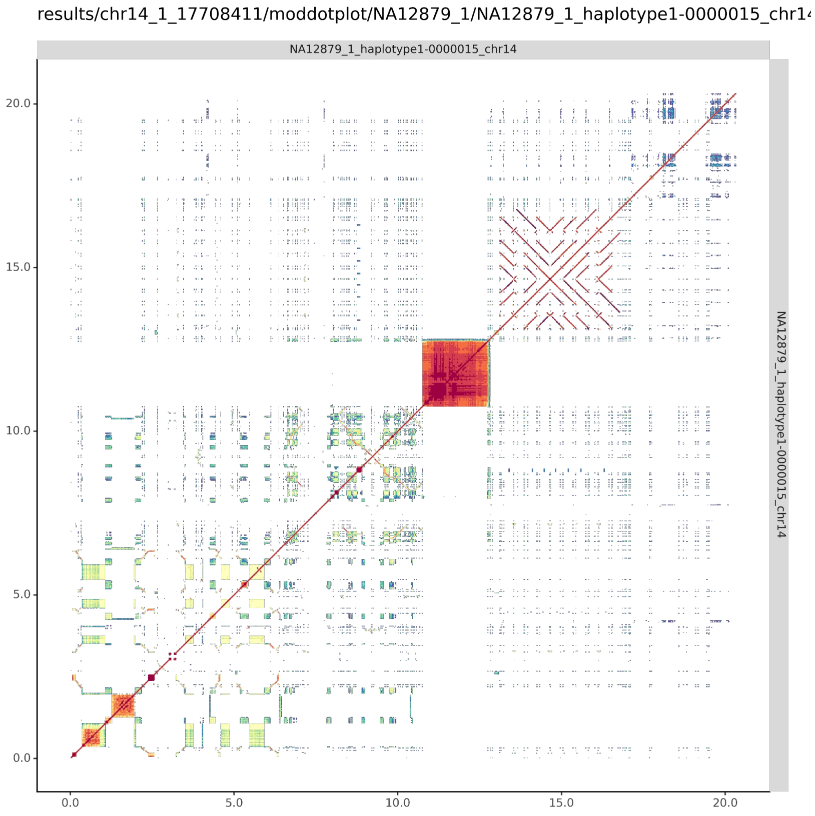

NA12879_2_haplotype2-0000083_chr14

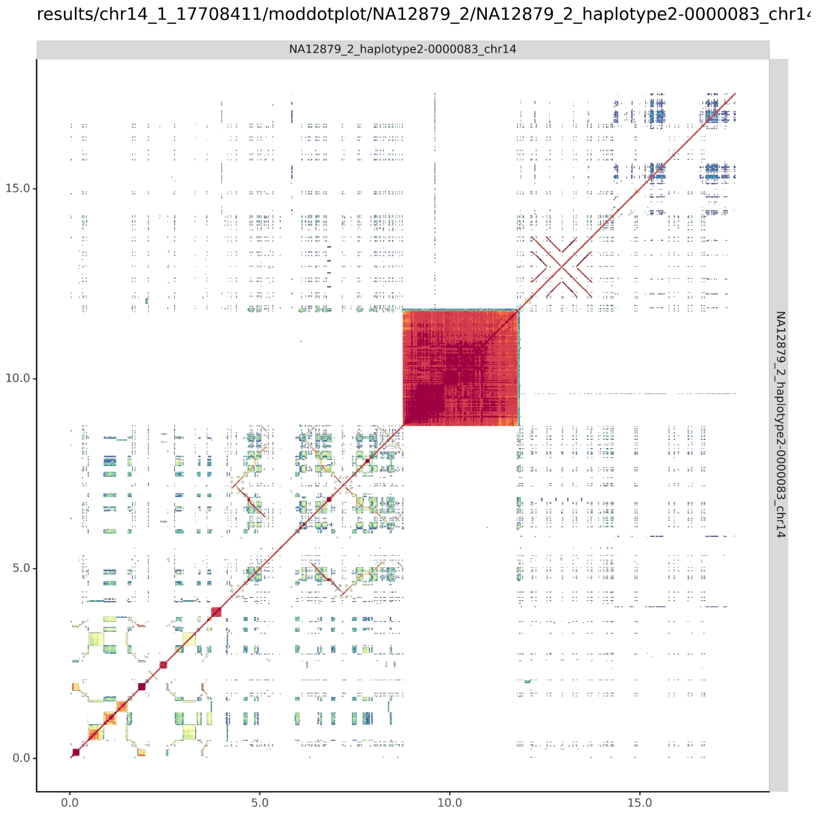

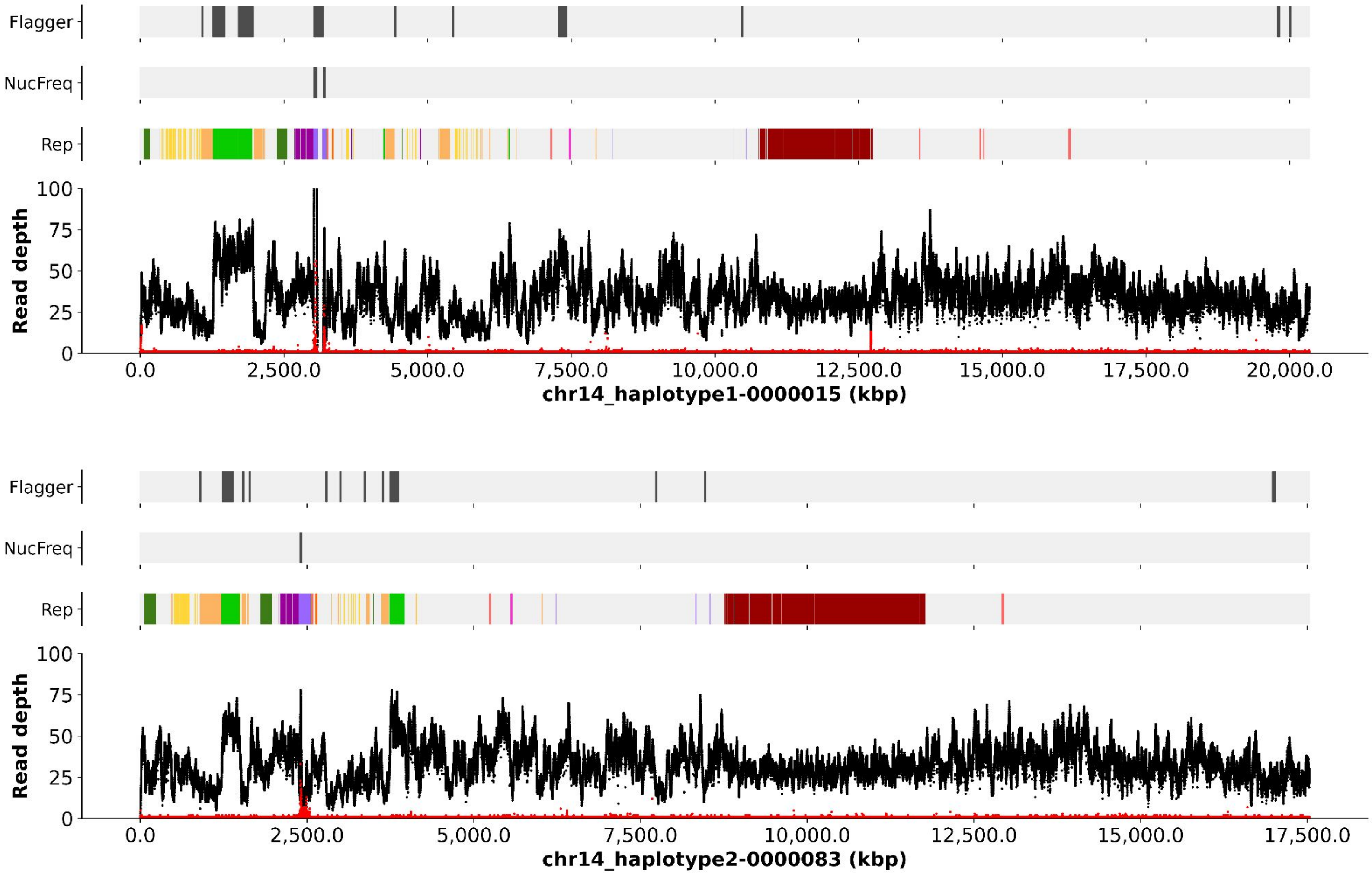
Flagger
NucFreq
Rep
Read depth
100
75
50
25
0
0.0
2,500.0
5,000.0
7,500.0
10,000.0
12,500.0
15,000.0
17,500.0
20,000.0
chr14_haplotype1-0000015 (kbp)
Flagger
NucFreq
Rep
Read depth
100
75
50
25
0
0.0
2,500.0
5,000.0
7,500.0
10,000.0
12,500.0
15,000.0
17,500.0
chr14_haplotype2-0000083 (kbp)

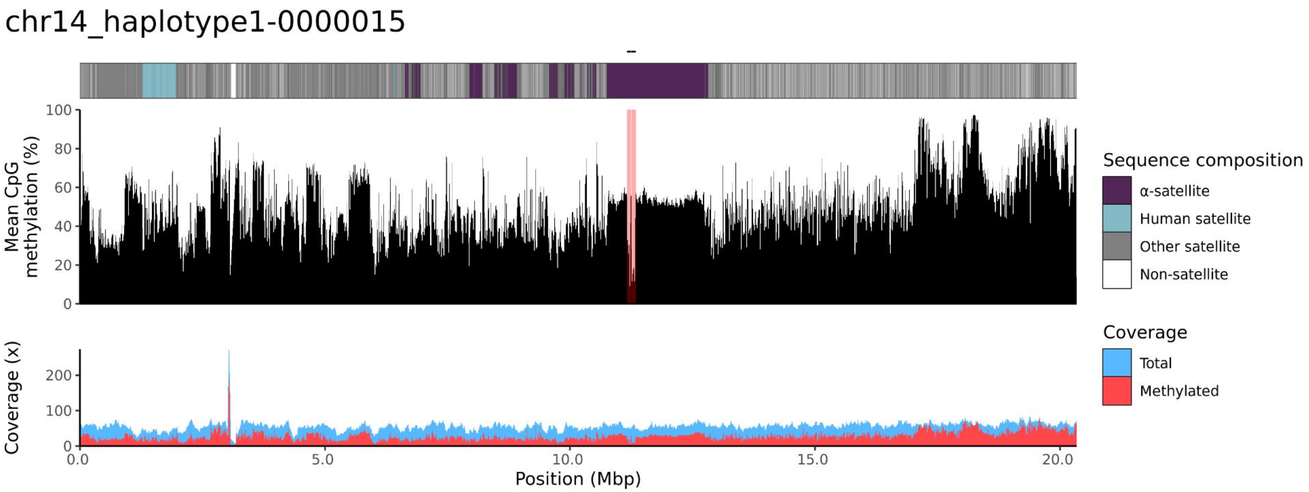
chr14_haplotype1-0000015
Mean CpG methylation (%)
0
20
40
60
80
100
Coverage (x)
0
100
200
0.0
5.0
10.0
15.0
20.0
Position (Mbp)
Sequence composition
α-satellite
Human satellite
Other satellite
Non-satellite
Coverage
Total
Methylated

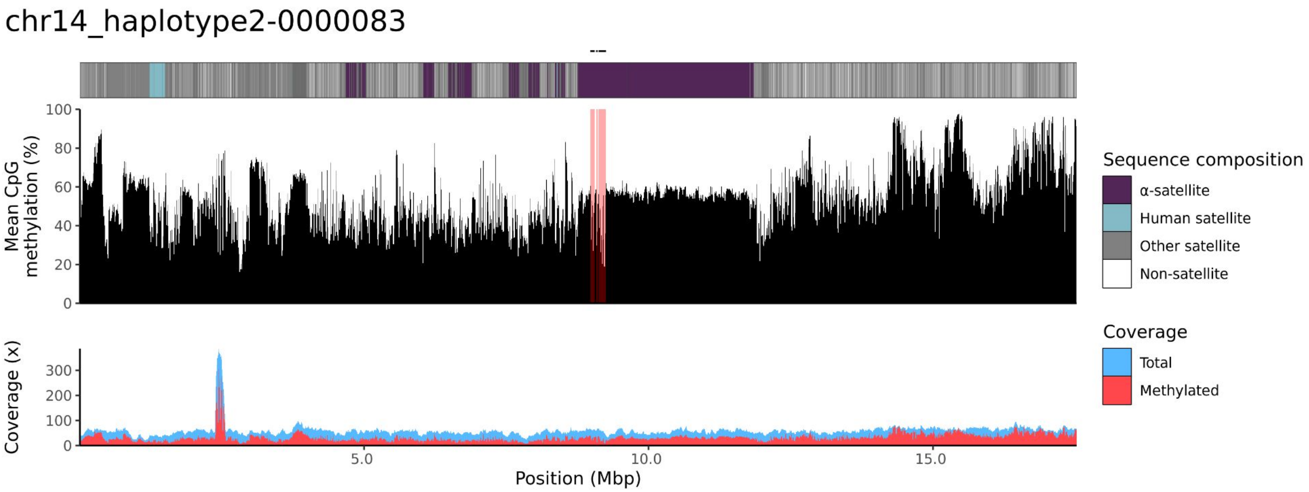
chr14_haplotype2-0000083
Mean CpG methylation (%)
0
20
40
60
80
100
Coverage (x)
0
100
200
300
5.0
10.0
15.0
Position (Mbp)
Sequence composition
α-satellite
Human satellite
Other satellite
Non-satellite
Coverage
Total
Methylated

chr15

NA12879_1_haplotype1-0000013_chr15

results/chr15_1_22694466/moddotplot/NA12879_1/NA12879_1_haplotype1-0000013_chr1!

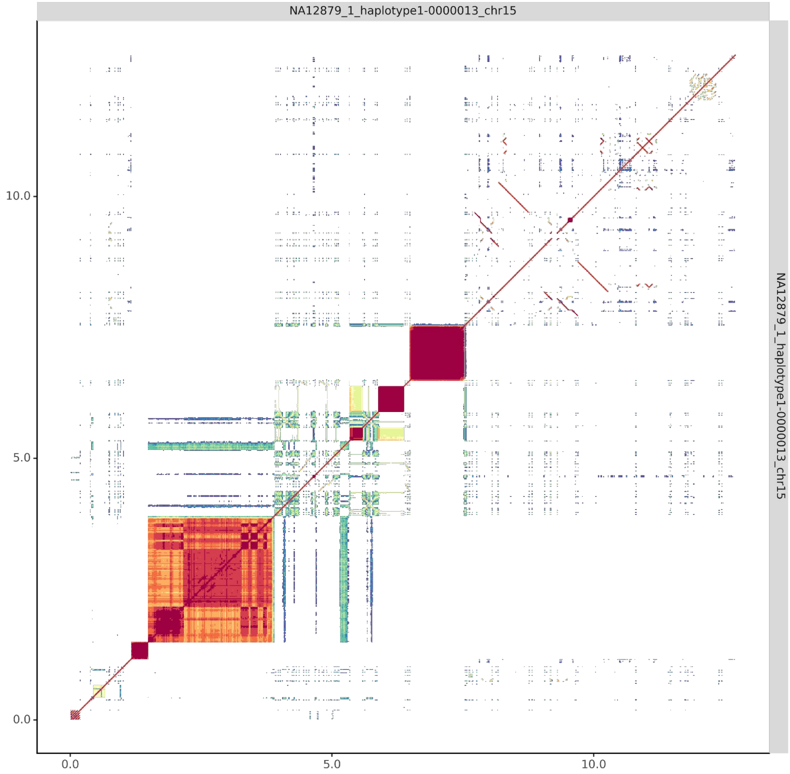

NA12879_2_haplotype2-0000075_chr15

results/chr15_1_22694466/moddotplot/NA12879_2/NA12879_2_haplotype2-0000075_chr1!

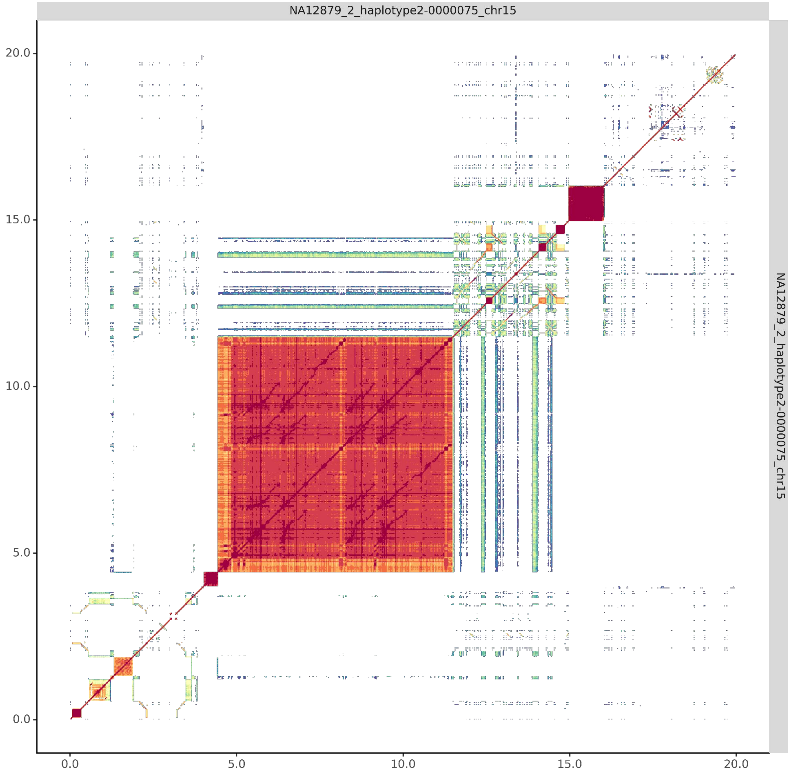

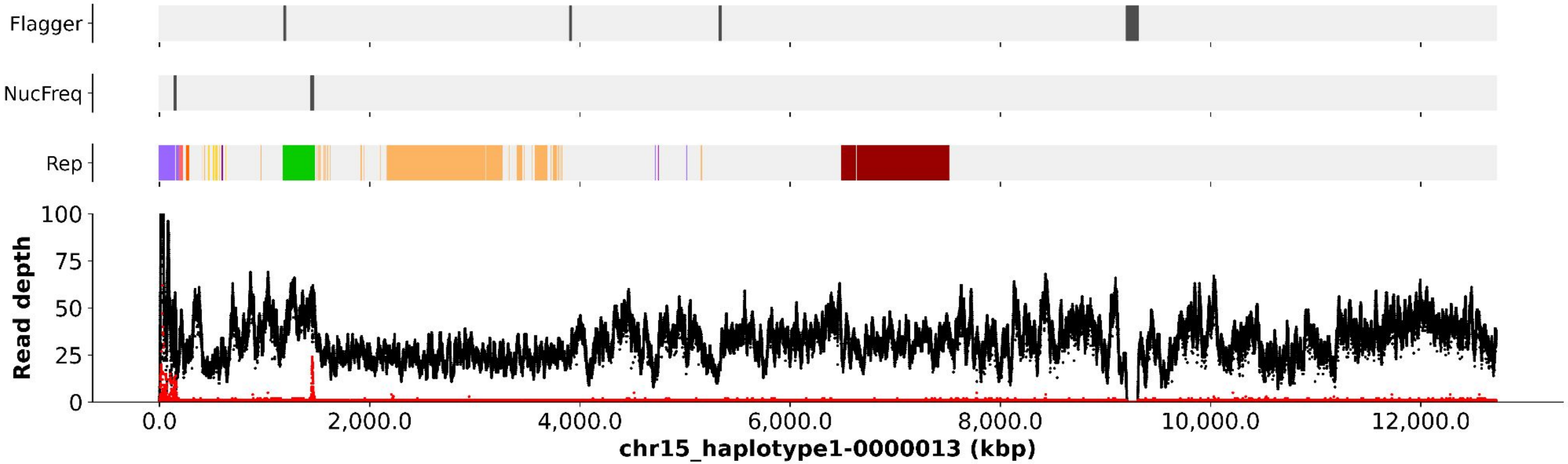

Flagger
NucFreq
Rep
Read depth
100
75
50
25
0
0.0
2,000.0
4,000.0
6,000.0
8,000.0
10,000.0
12,000.0
chr15_haplotype1-0000013 (kbp)

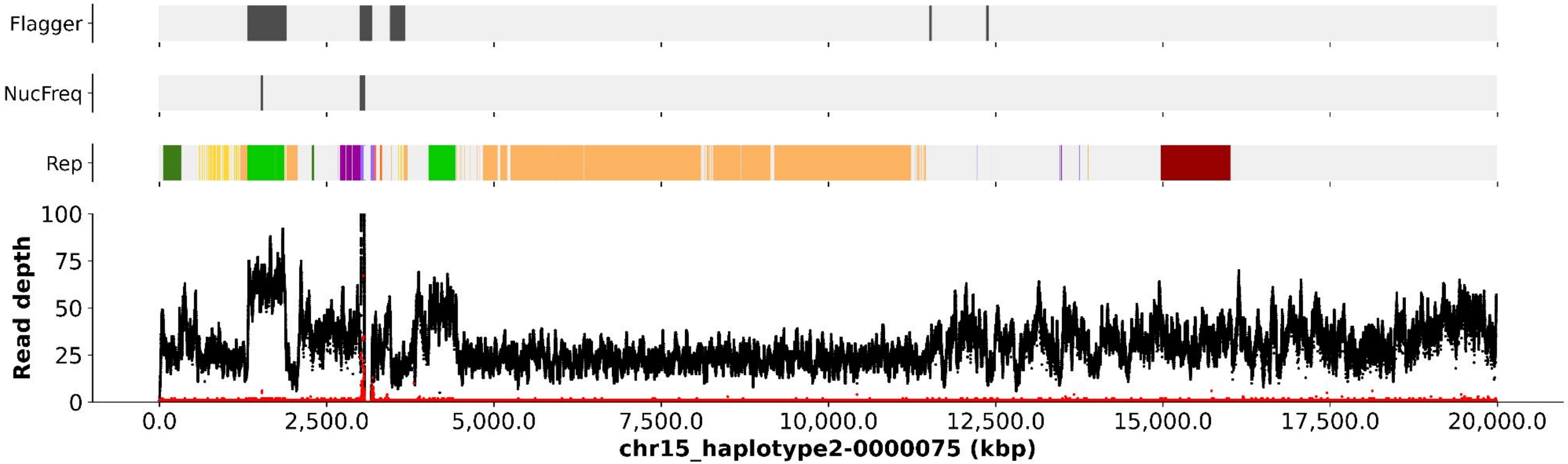

Flagger
NucFreq
Rep
Read depth
100
75
50
25
0
0.0
2,500.0
5,000.0
7,500.0
10,000.0
12,500.0
15,000.0
17,500.0
20,000.0
chr15_haplotype2-0000075 (kbp)

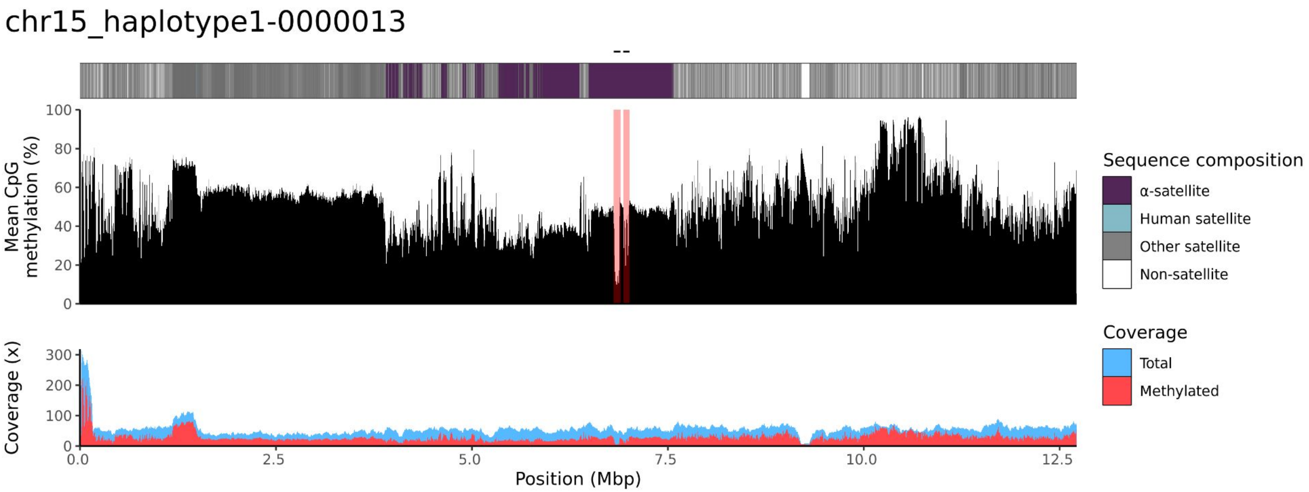
chr15_haplotype1-0000013
Mean CpG methylation (%)
100
80
60
40
20
0
Coverage (x)
300
200
100
0
0.0
2.5
5.0
7.5
10.0
12.5
Position (Mbp)
Sequence composition
α-satellite
Human satellite
Other satellite
Non-satellite
Coverage
Total
Methylated

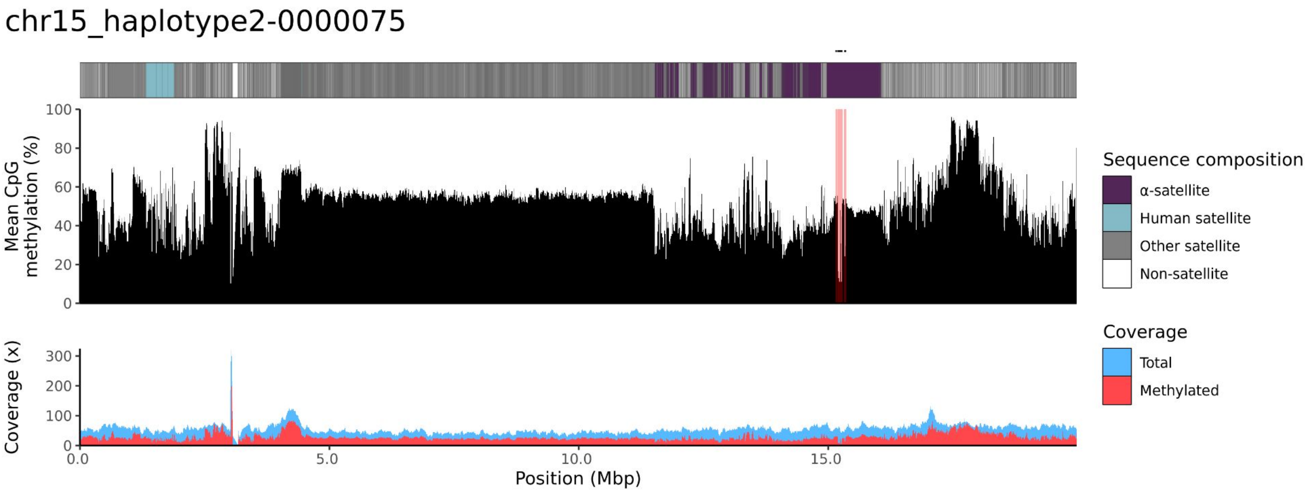
chr15_haplotype2-0000075
Mean CpG methylation (%)
100
80
60
40
20
0
Coverage (x)
300
200
100
0
0.0
5.0
10.0
15.0
Position (Mbp)
Sequence composition
α-satellite
Human satellite
Other satellite
Non-satellite
Coverage
Total
Methylated

chr21

NA12879_1_haplotype1-0000019_chr21

results/chr21_1_16306378/moddotplot/NA12879_1/NA12879_1_haplotype1-0000019_chr2:

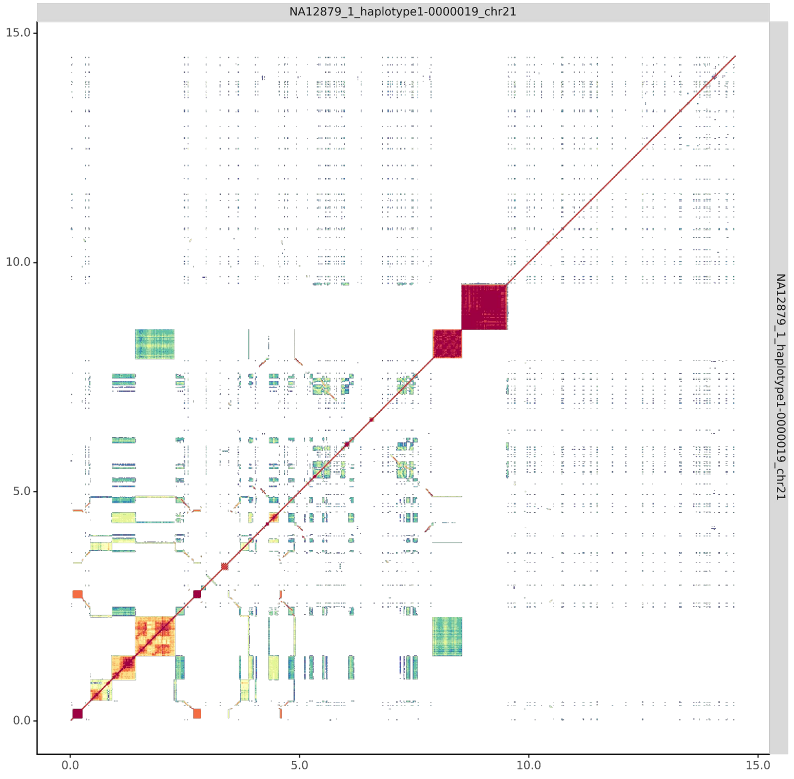

NA12879_2_haplotype2-0000087_chr21

results/chr21_1_16306378/moddotplot/NA12879_2/NA12879_2_haplotype2-0000087_chr2:

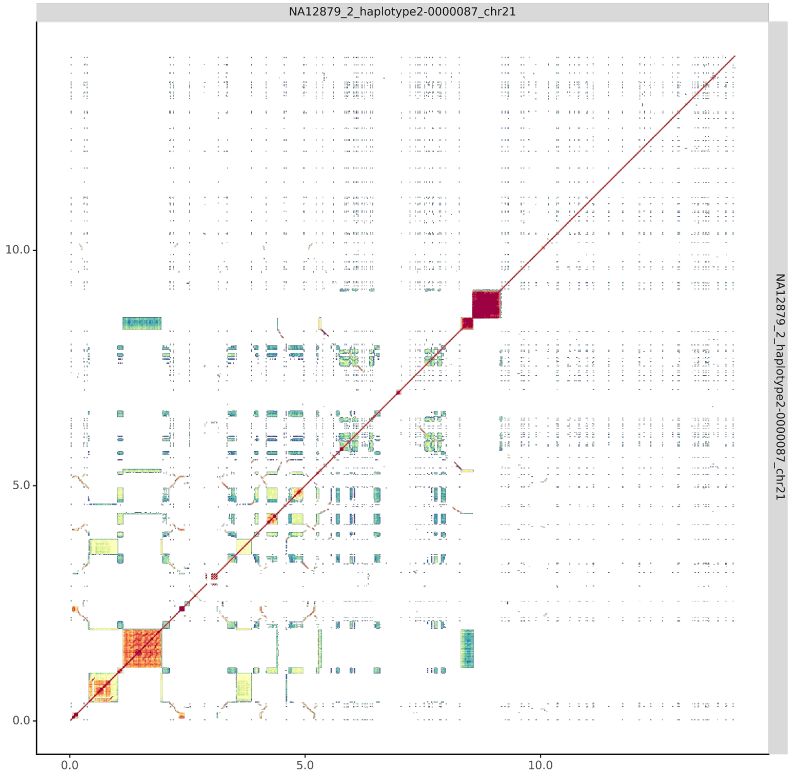

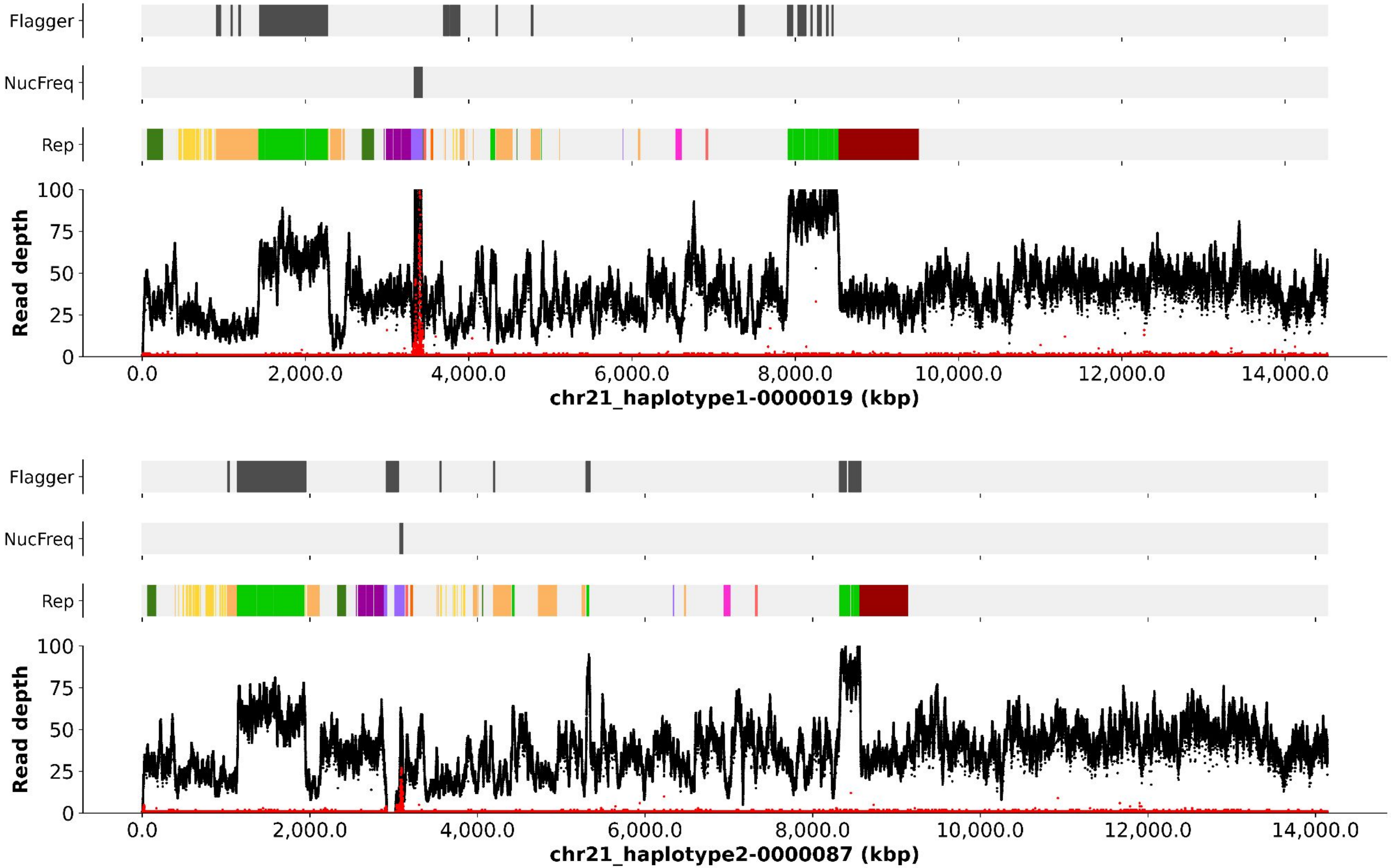

Flagger
NucFreq
Rep
Read depth
100
75
50
25
0
0.0
2,000.0
4,000.0
6,000.0
8,000.0
10,000.0
12,000.0
14,000.0
chr21_haplotype1-0000019 (kbp)
chr21_haplotype2-0000087 (kbp)

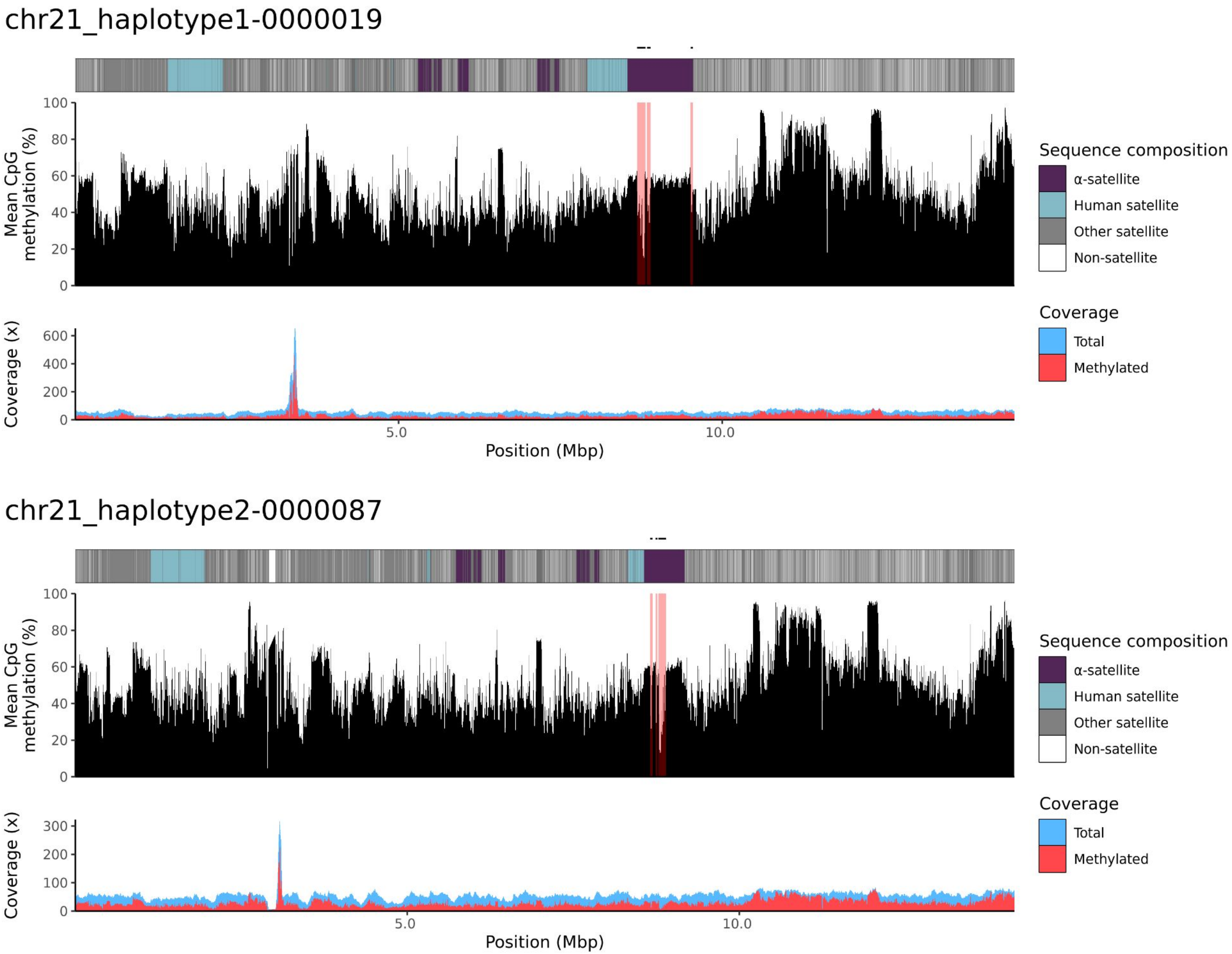
chr21_haplotype1-0000019
Mean CpG methylation (%)
100
80
60
40
20
0
Coverage (x)
600
400
200
0
5.0
10.0
Position (Mbp)
Sequence composition
α-satellite
Human satellite
Other satellite
Non-satellite
Coverage
Total
Methylated
chr21_haplotype2-0000087
Mean CpG methylation (%)
100
80
60
40
20
0
Coverage (x)
300
200
100
0
5.0
10.0
Position (Mbp)
Sequence composition
α-satellite
Human satellite
Other satellite
Non-satellite
Coverage
Total
Methylated

chr22

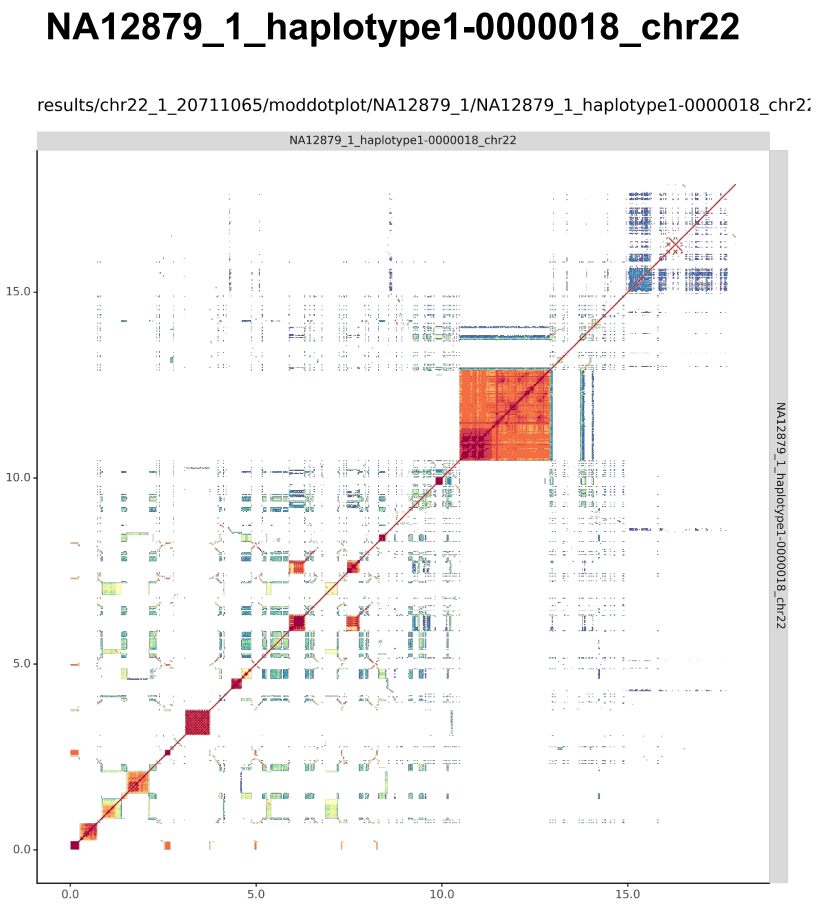

NA12879_1_haplotype1-0000018_chr22
results/chr22_1_20711065/moddotplot/NA12879_1/NA12879_1_haplotype1-0000018_chr22
NA12879_1_haplotype1-0000018_chr22
0.0
5.0
10.0
15.0
NA12879_1_haplotype1-0000018_chr22

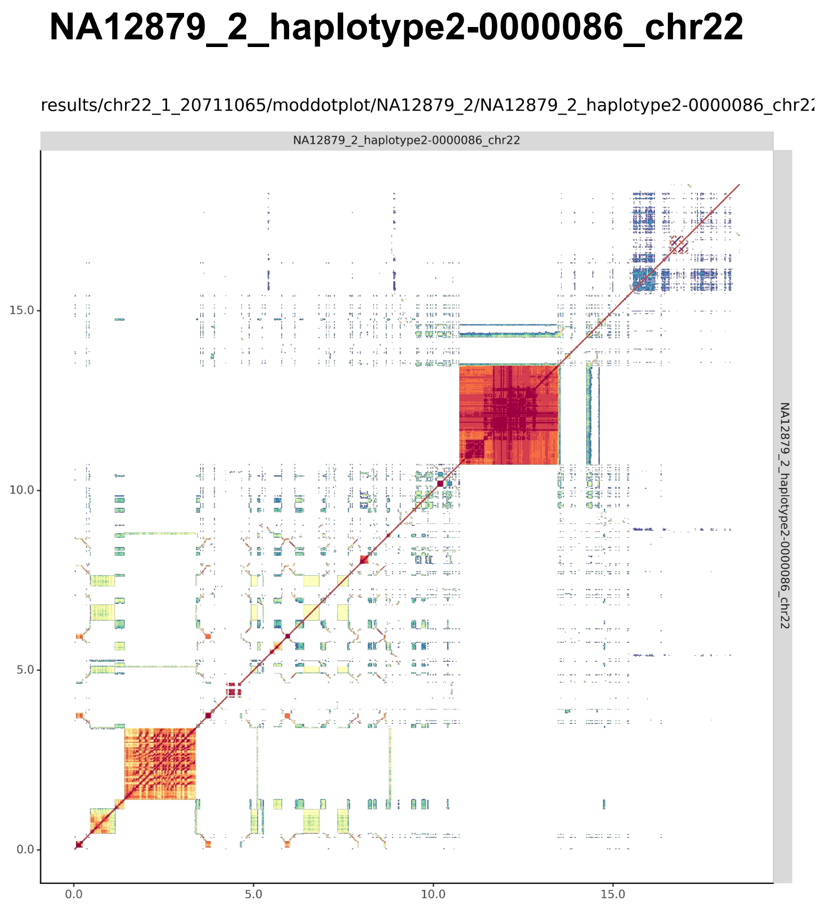

NA12879_2_haplotype2-0000086_chr22
results/chr22_1_20711065/moddotplot/NA12879_2/NA12879_2_haplotype2-0000086_chr22
NA12879_2_haplotype2-0000086_chr22
0.0
5.0
10.0
15.0
NA12879_2_haplotype2-0000086_chr22

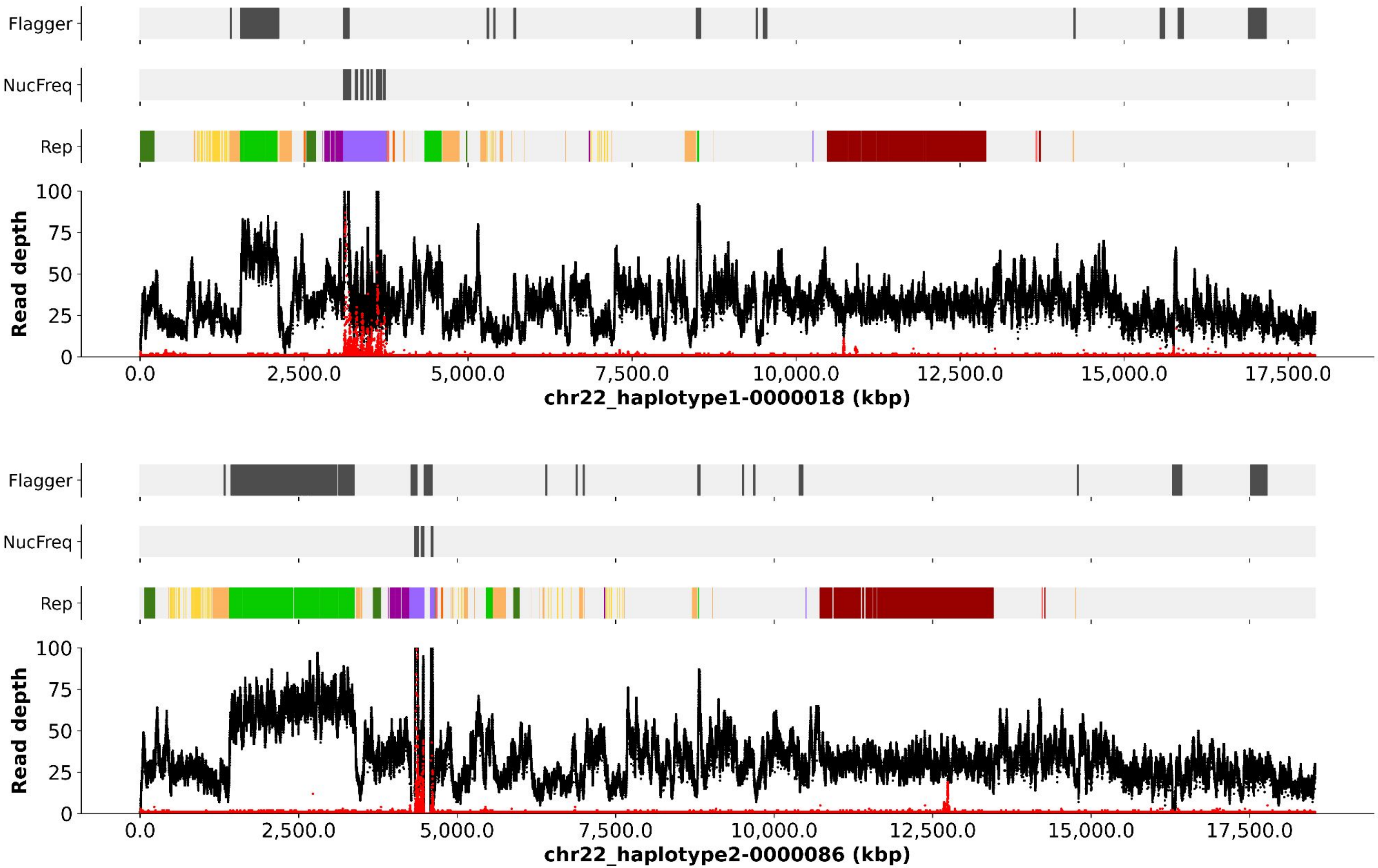
Flagger
NucFreq
Rep
Read depth
100
75
50
25
0
0.0
2,500.0
5,000.0
7,500.0
10,000.0
12,500.0
15,000.0
17,500.0
chr22_haplotype1-0000018 (kbp)
Flagger
NucFreq
Rep
Read depth
100
75
50
25
0
0.0
2,500.0
5,000.0
7,500.0
10,000.0
12,500.0
15,000.0
17,500.0
chr22_haplotype2-0000086 (kbp)

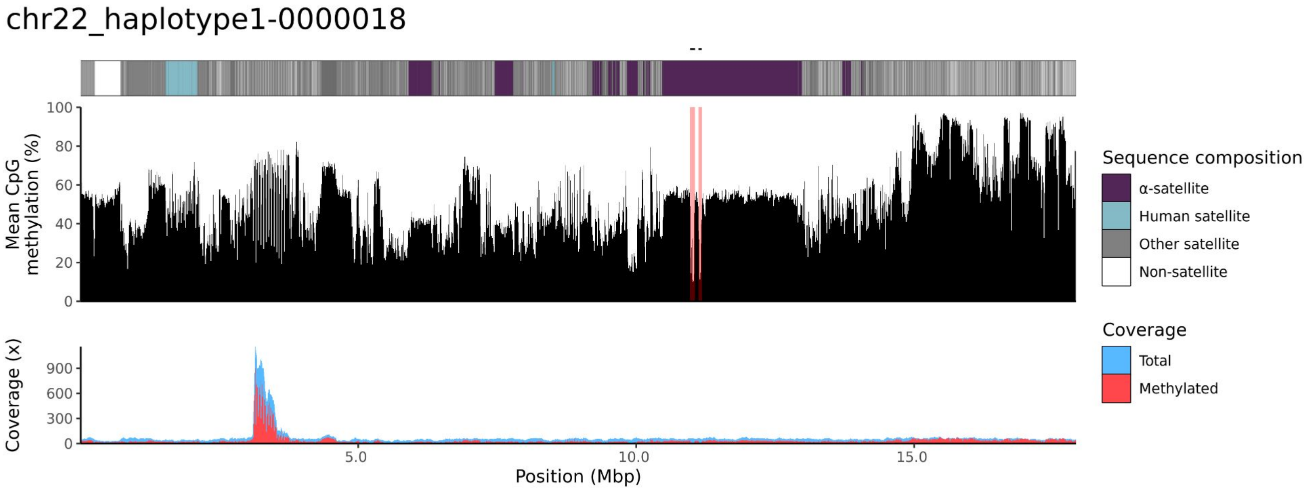

chr22_haplotype1-0000018
Mean CpG methylation (%)
100
80
60
40
20
0
Coverage (x)
900
600
300
0
5.0
10.0
15.0
Position (Mbp)
Sequence composition
α-satellite
Human satellite
Other satellite
Non-satellite
Coverage
Total
Methylated

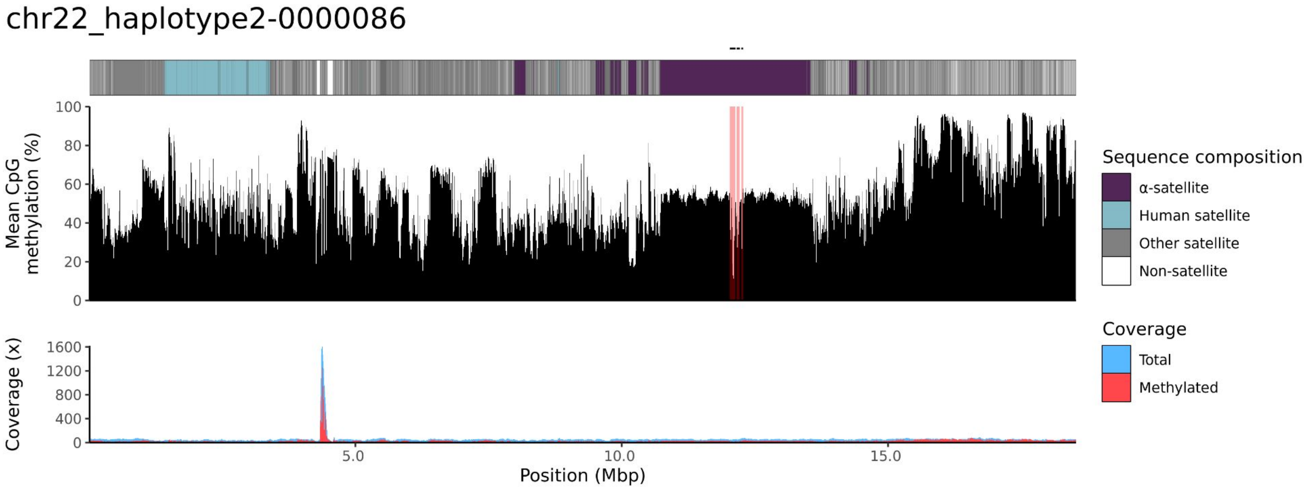

chr22_haplotype2-0000086
Mean CpG methylation (%)
100
80
60
40
20
0
Coverage (x)
1600
1200
800
400
0
5.0
10.0
15.0
Position (Mbp)
Sequence composition
α-satellite
Human satellite
Other satellite
Non-satellite
Coverage
Total
Methylated

NA12881

chr13

NA12881_1_haplotype1-0000010_chr13

NA12881_2_haplotype2-0000061_chr13

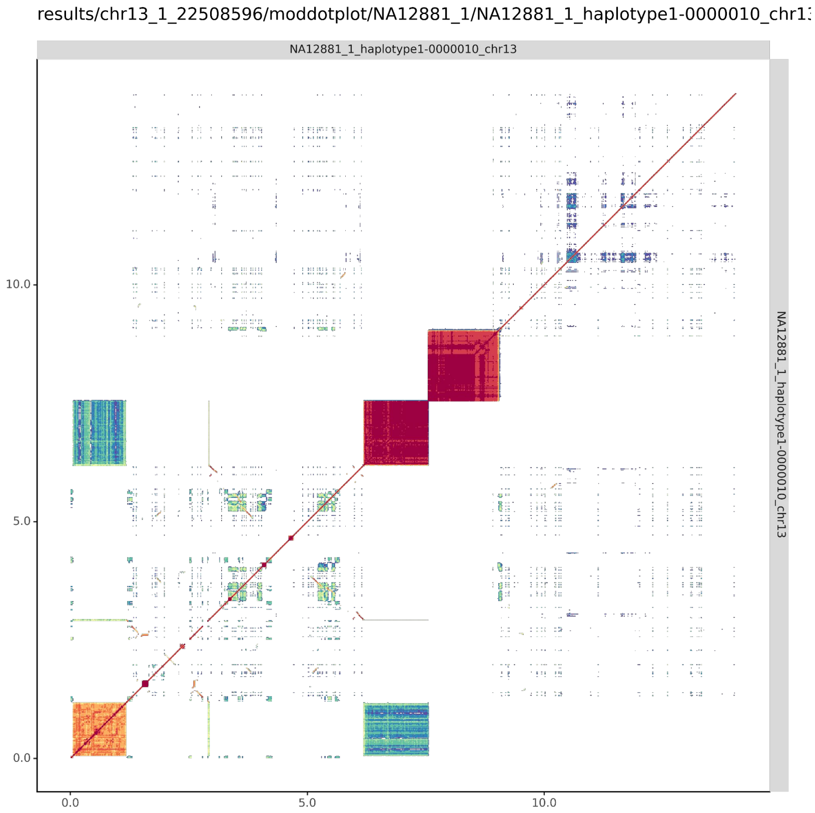

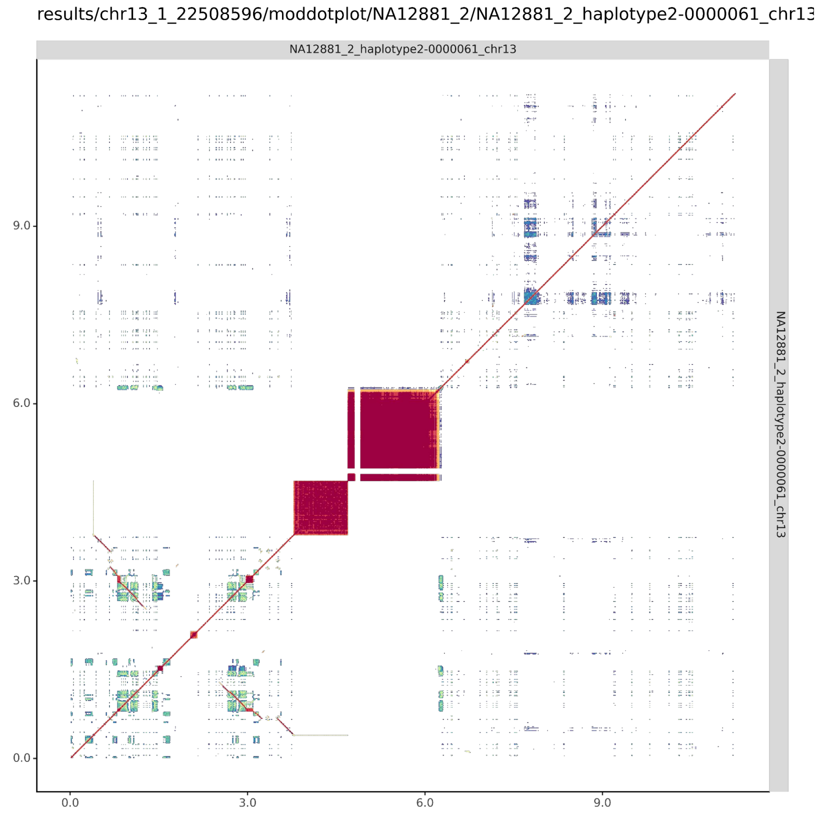

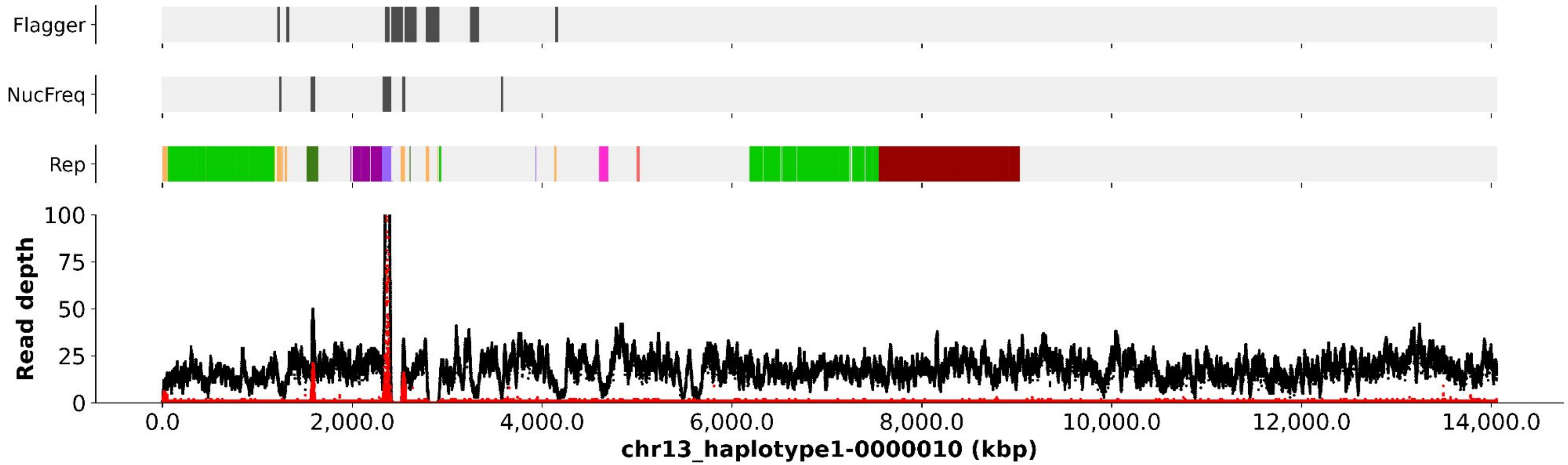

Flagger
NucFreq
Rep
Read depth
100
75
50
25
0
0.0
2,000.0
4,000.0
6,000.0
8,000.0
10,000.0
12,000.0
14,000.0
chr13_haplotype1-0000010 (kbp)

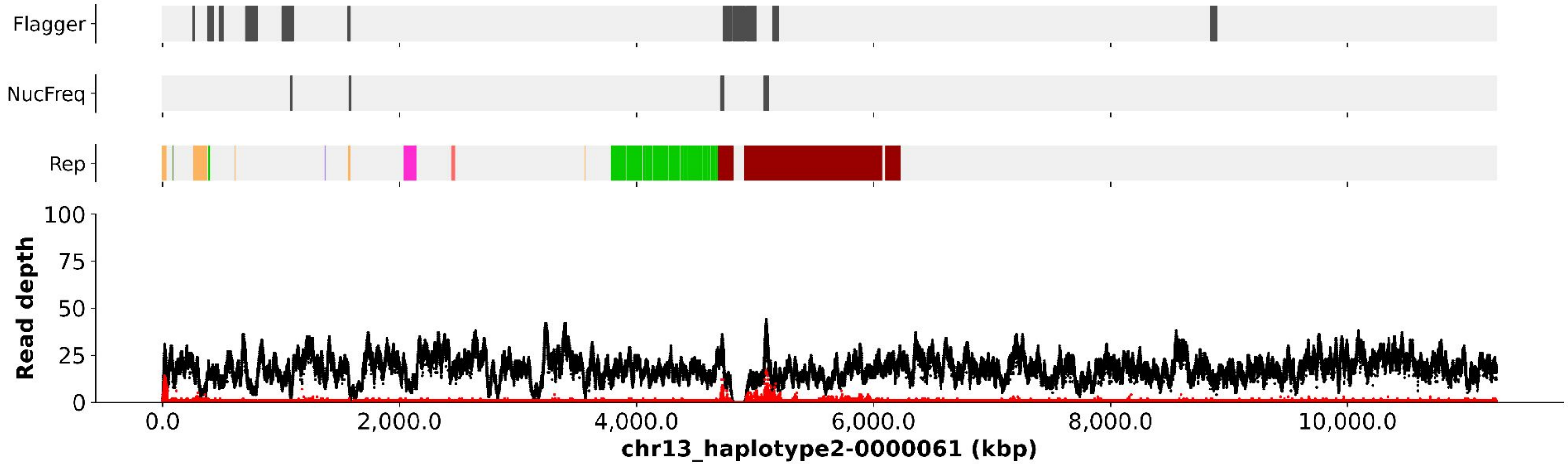

Flagger
NucFreq
Rep
Read depth
100
75
50
25
0
0.0
2,000.0
4,000.0
6,000.0
8,000.0
10,000.0
chr13_haplotype2-0000061 (kbp)

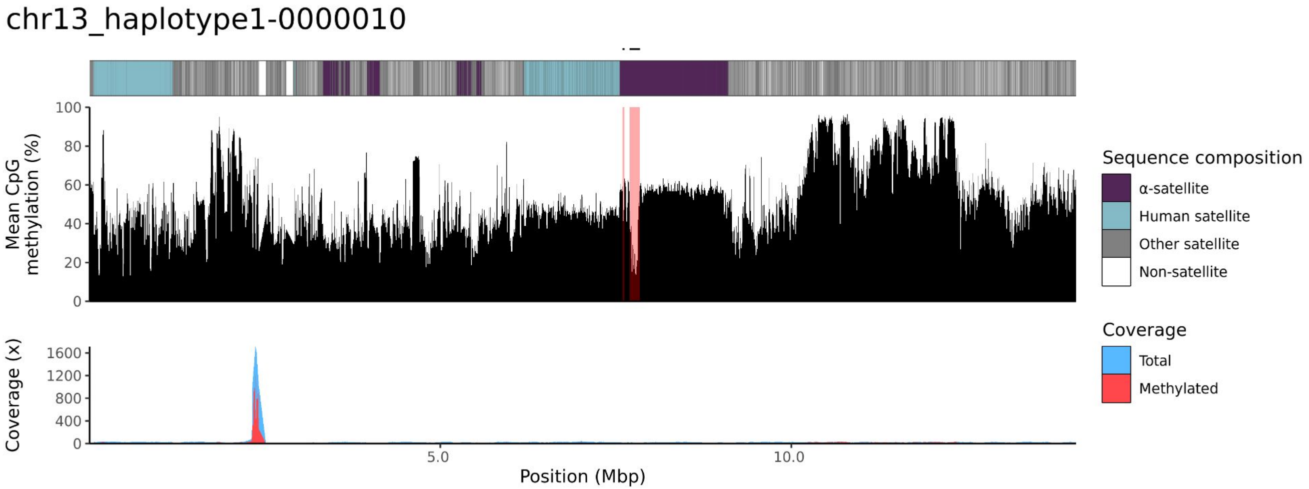
chr13_haplotype1-0000010
Mean CpG methylation (%)
0
20
40
60
80
100
Coverage (x)
0
400
800
1200
1600
5.0
10.0
Position (Mbp)
Sequence composition
α-satellite
Human satellite
Other satellite
Non-satellite
Coverage
Total
Methylated

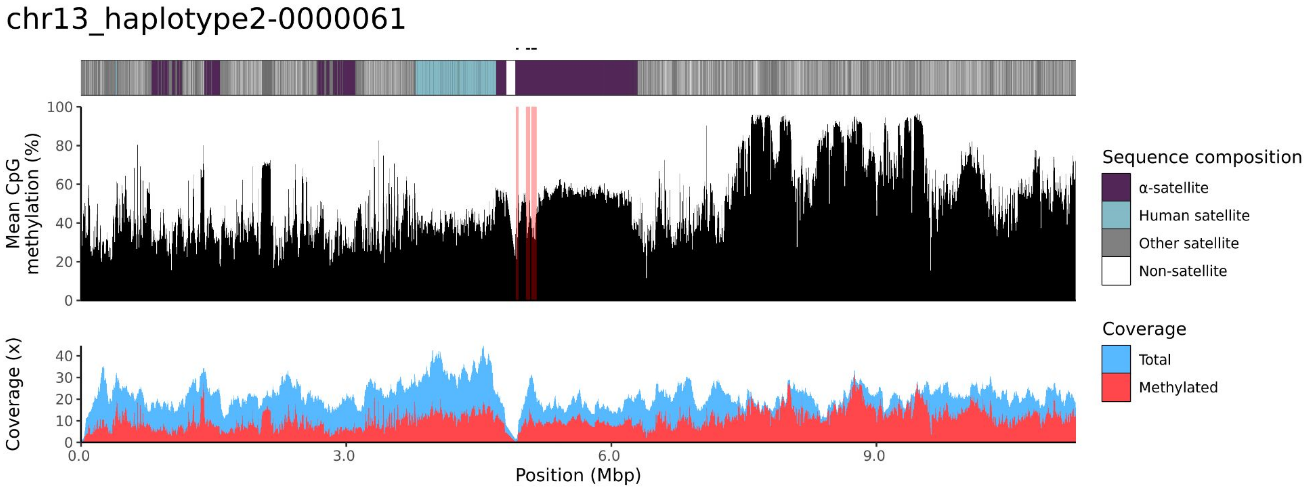
chr13_haplotype2-0000061
Mean CpG methylation (%)
0
20
40
60
80
100
Coverage (x)
0
10
20
30
40
0.0
3.0
6.0
9.0
Position (Mbp)
Sequence composition
α-satellite
Human satellite
Other satellite
Non-satellite
Coverage
Total
Methylated

chr14

NA12881_1_haplotype1-0000011_chr14

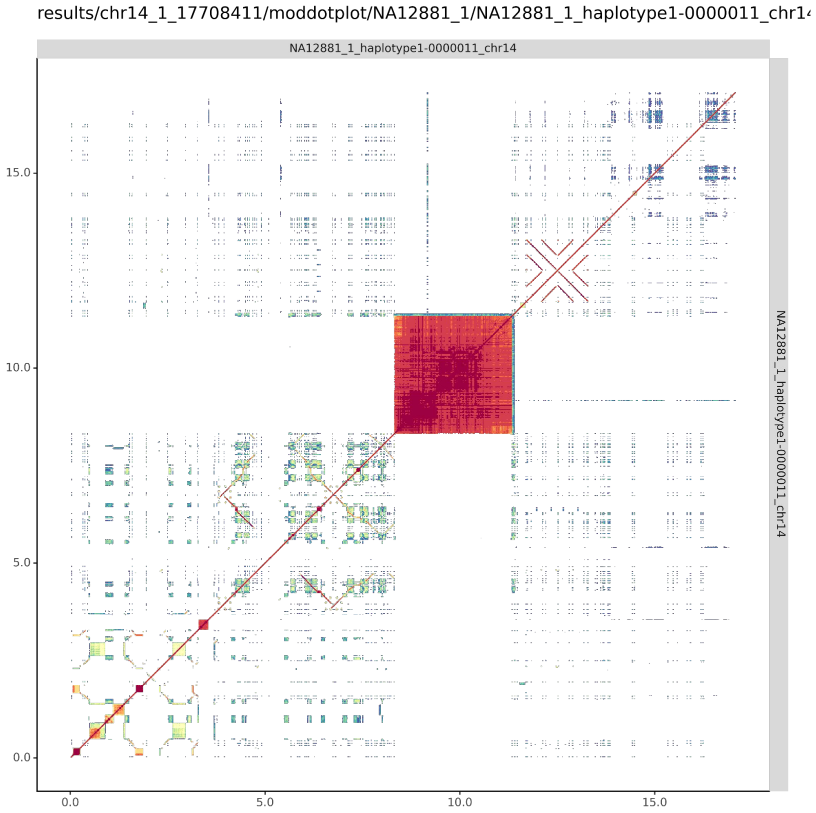

NA12881_2_haplotype2-0000062_chr14

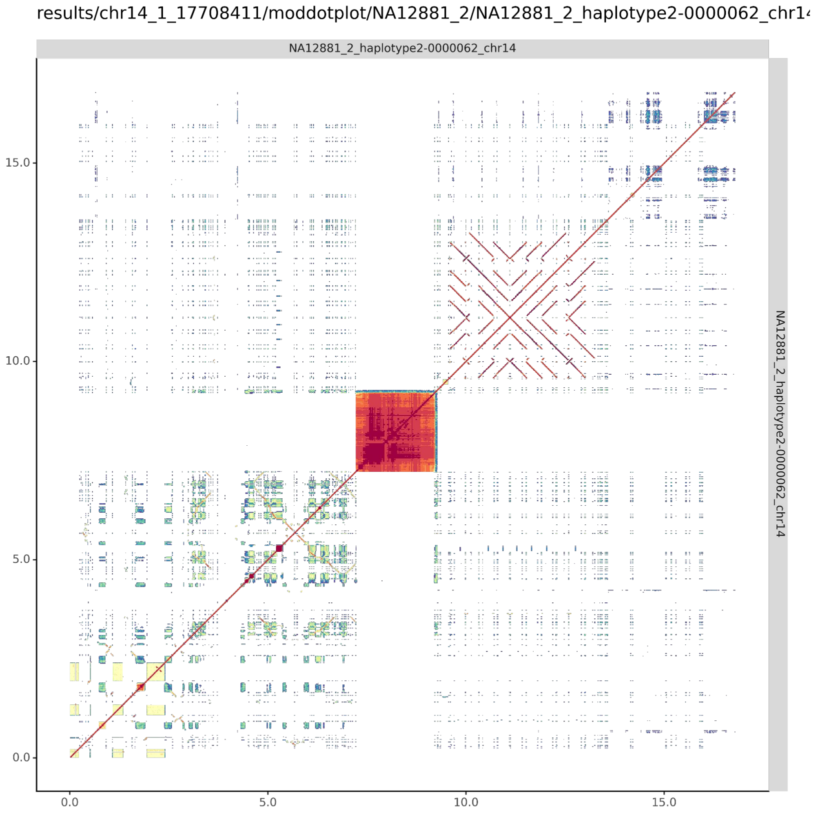

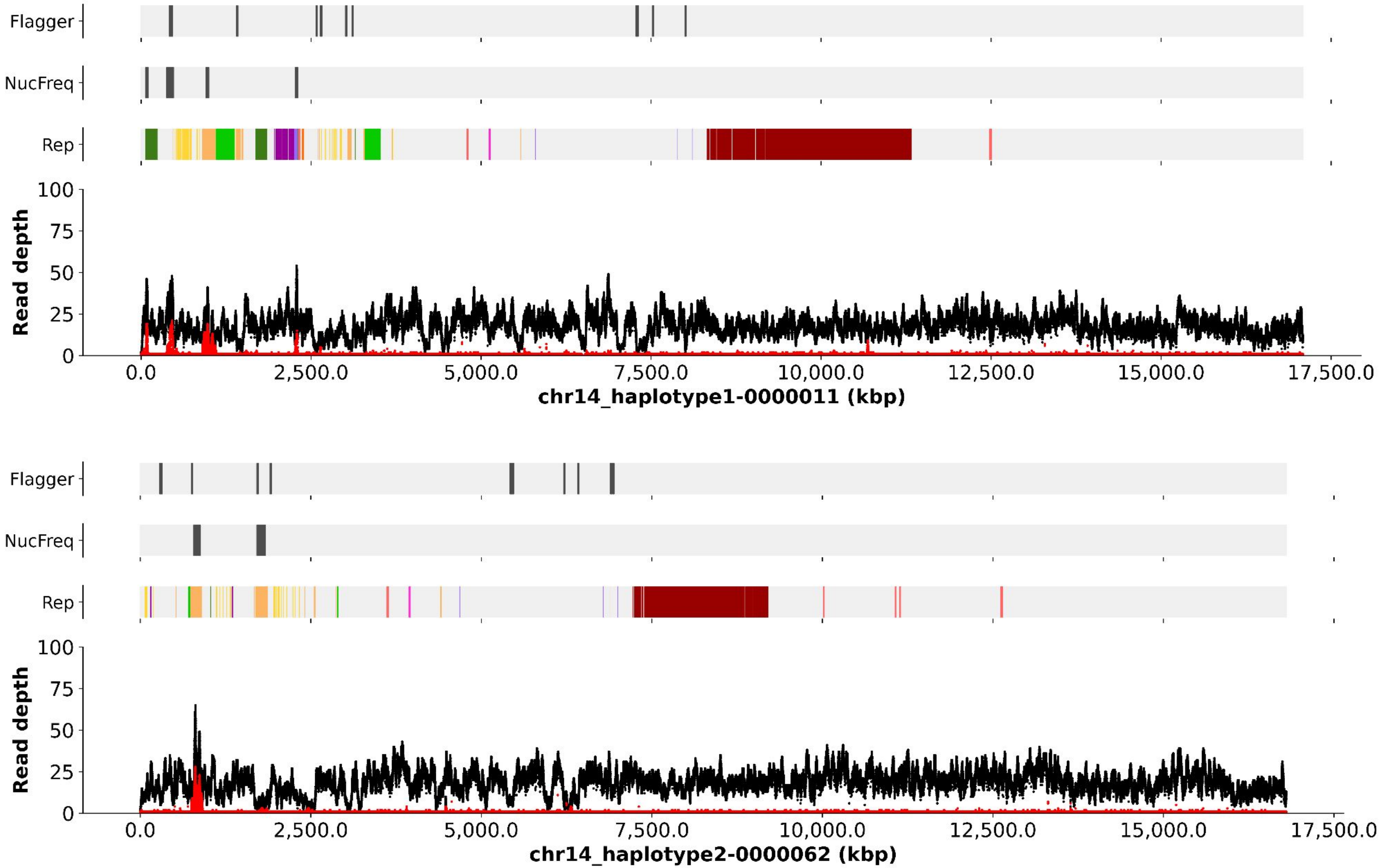

Flagger
NucFreq
Rep
Read depth
100
75
50
25
0
0.0
2,500.0
5,000.0
7,500.0
10,000.0
12,500.0
15,000.0
17,500.0
chr14_haplotype1-0000011 (kbp)
Flagger
NucFreq
Rep
Read depth
100
75
50
25
0
0.0
2,500.0
5,000.0
7,500.0
10,000.0
12,500.0
15,000.0
17,500.0
chr14_haplotype2-0000062 (kbp)

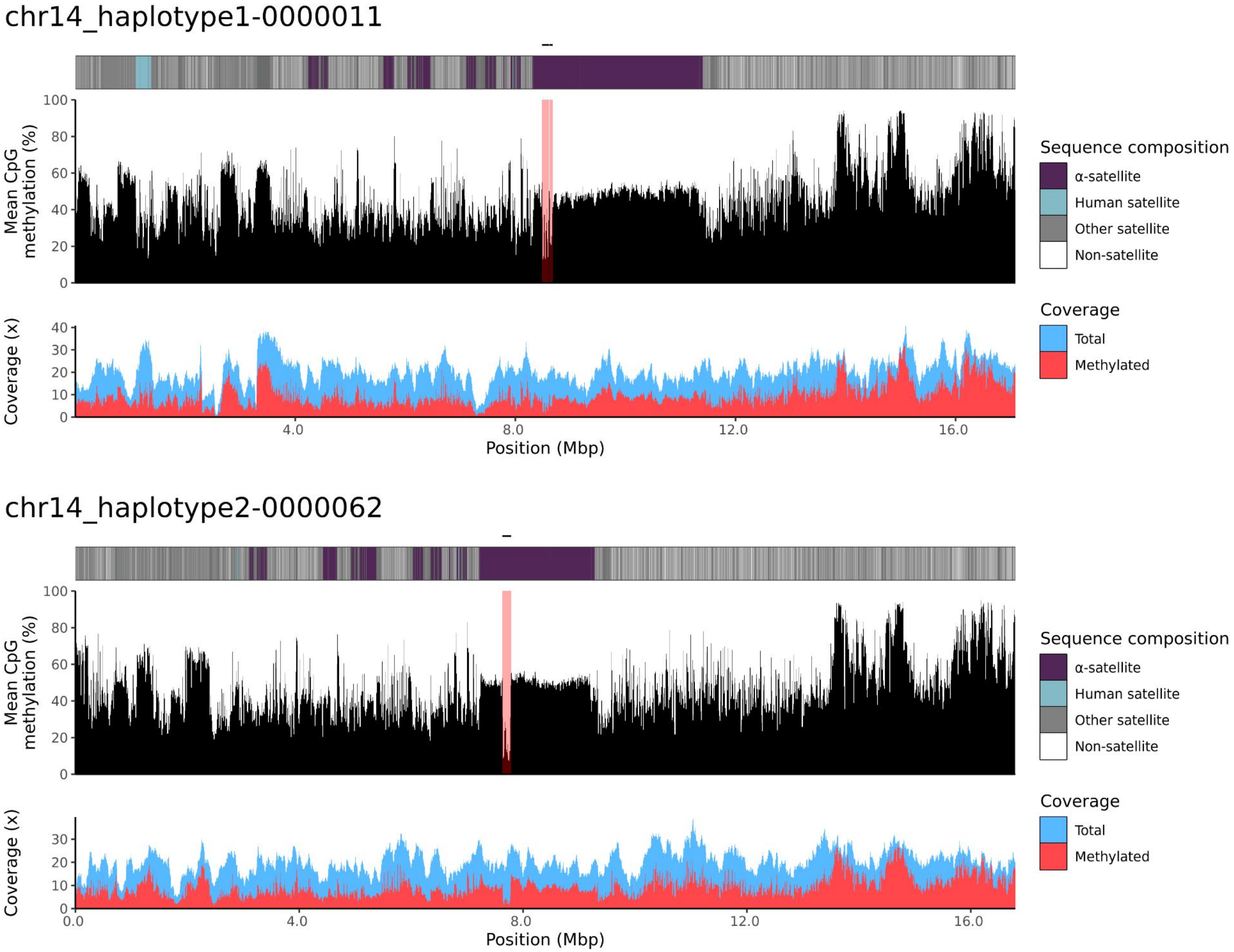
chr14_haplotype1-0000011
Sequence composition
α-satellite
Human satellite
Other satellite
Non-satellite
Coverage
Total
Methylated
Mean CpG methylation (%)
Coverage (x)
Position (Mbp)
chr14_haplotype2-0000062

chr15

NA12881_1_haplotype1-0000002_chr15

results/chr15_1_22694466/moddotplot/NA12881_1/NA12881_1_haplotype1-0000002_chr15

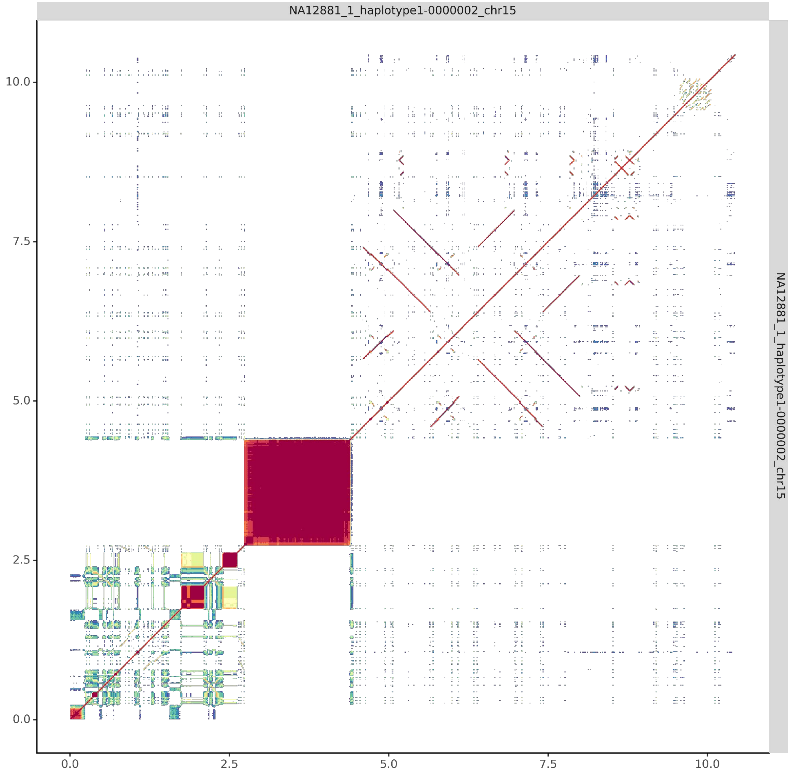

NA12881_2_haplotype2-0000055_chr15

results/chr15_1_22694466/moddotplot/NA12881_2/NA12881_2_haplotype2-0000055_chr15

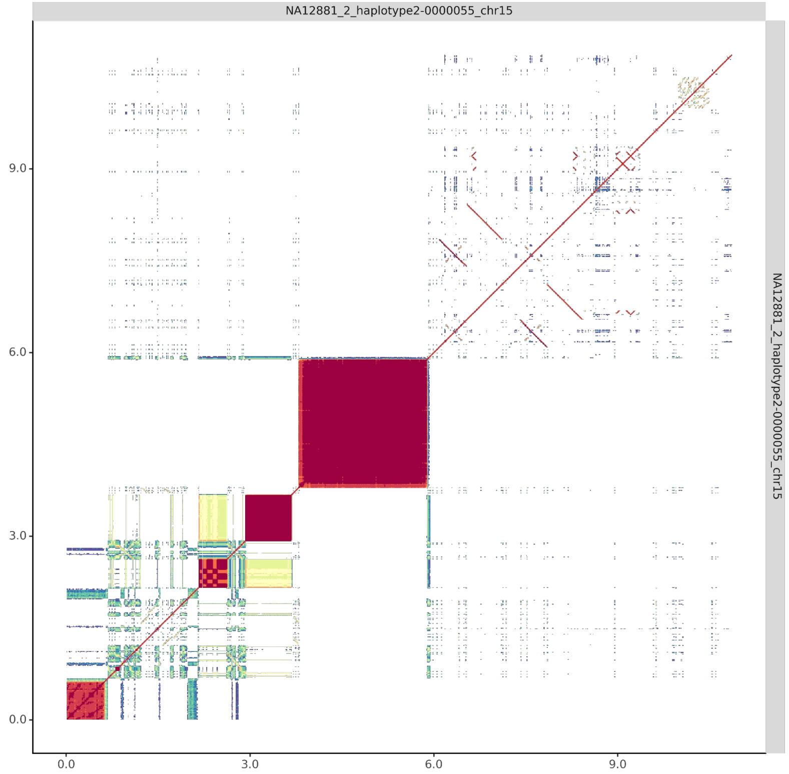

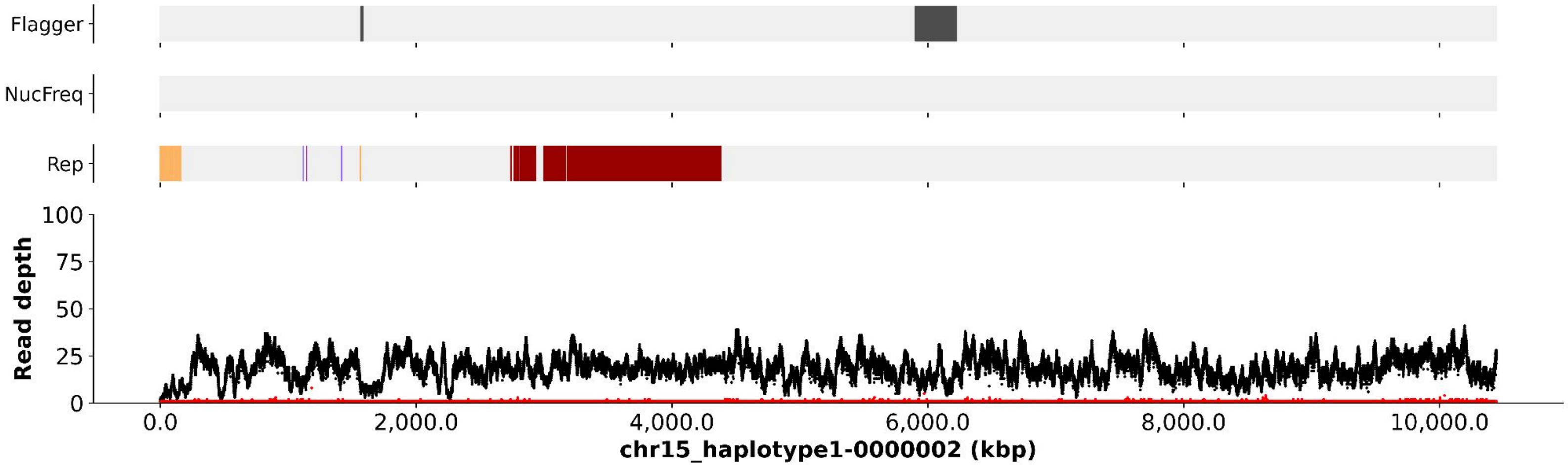

Flagger
NucFreq
Rep
Read depth
100
75
50
25
0
0.0
2,000.0
4,000.0
6,000.0
8,000.0
10,000.0
chr15_haplotype1-0000002 (kbp)

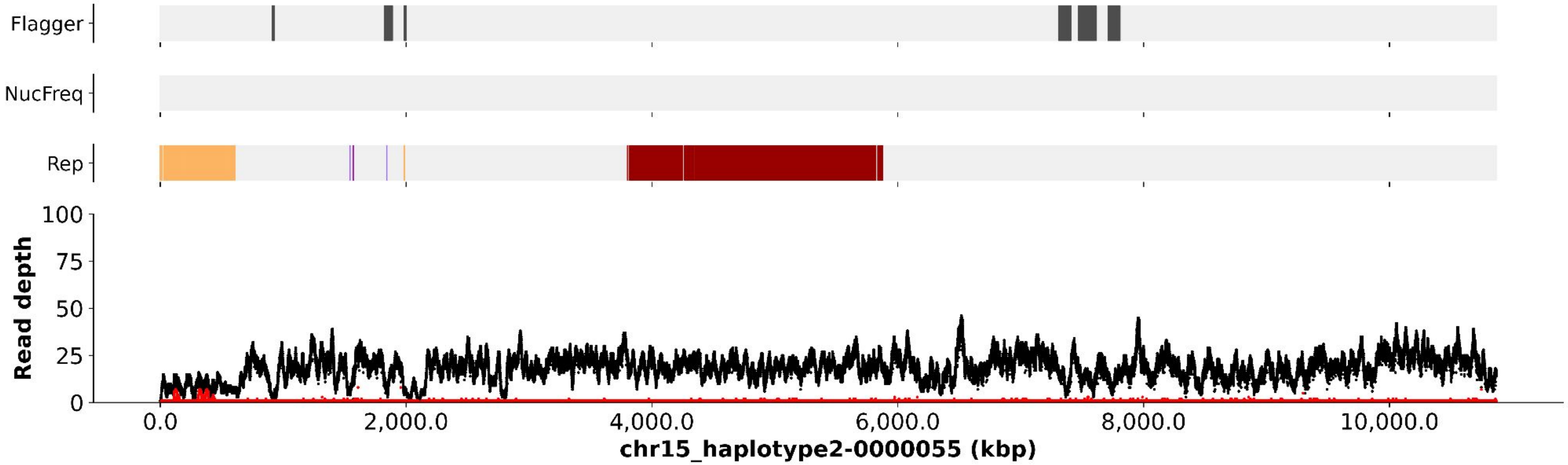

Flagger
NucFreq
Rep
Read depth
100
75
50
25
0
0.0
2,000.0
4,000.0
6,000.0
8,000.0
10,000.0
chr15_haplotype2-0000055 (kbp)

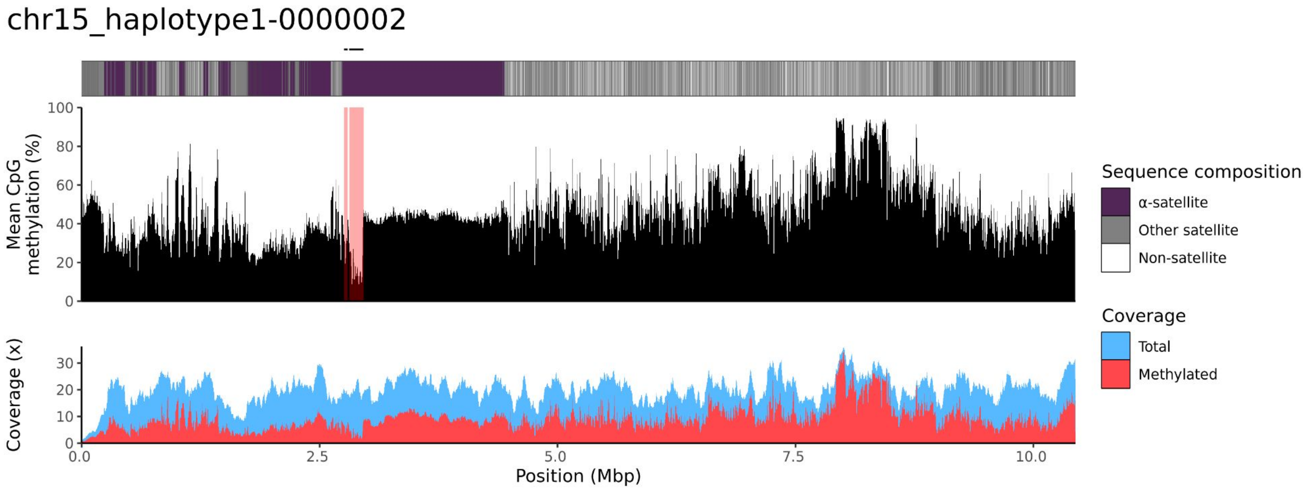
chr15_haplotype1-0000002
Mean CpG methylation (%)
100
80
60
40
20
0
Coverage (x)
30
20
10
0
0.0
2.5
5.0
7.5
10.0
Position (Mbp)
Sequence composition
α-satellite
Other satellite
Non-satellite
Coverage
Total
Methylated

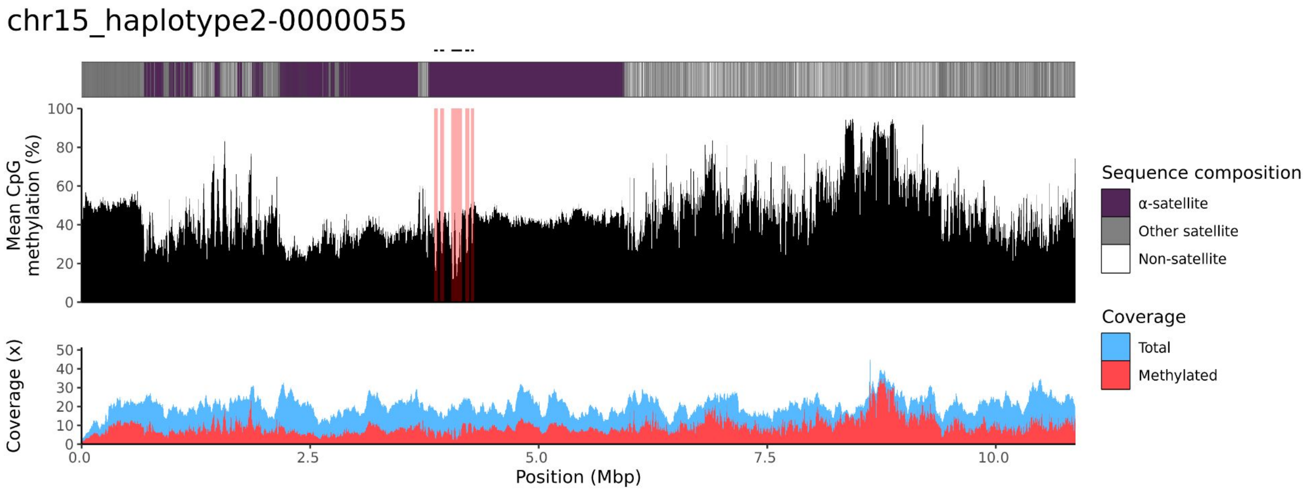
chr15_haplotype2-0000055
Mean CpG methylation (%)
100
80
60
40
20
0
Coverage (x)
50
40
30
20
10
0
0.0
2.5
5.0
7.5
10.0
Position (Mbp)
Sequence composition
α-satellite
Other satellite
Non-satellite
Coverage
Total
Methylated

chr21

NA12881_1_haplotype1-0000007_chr21

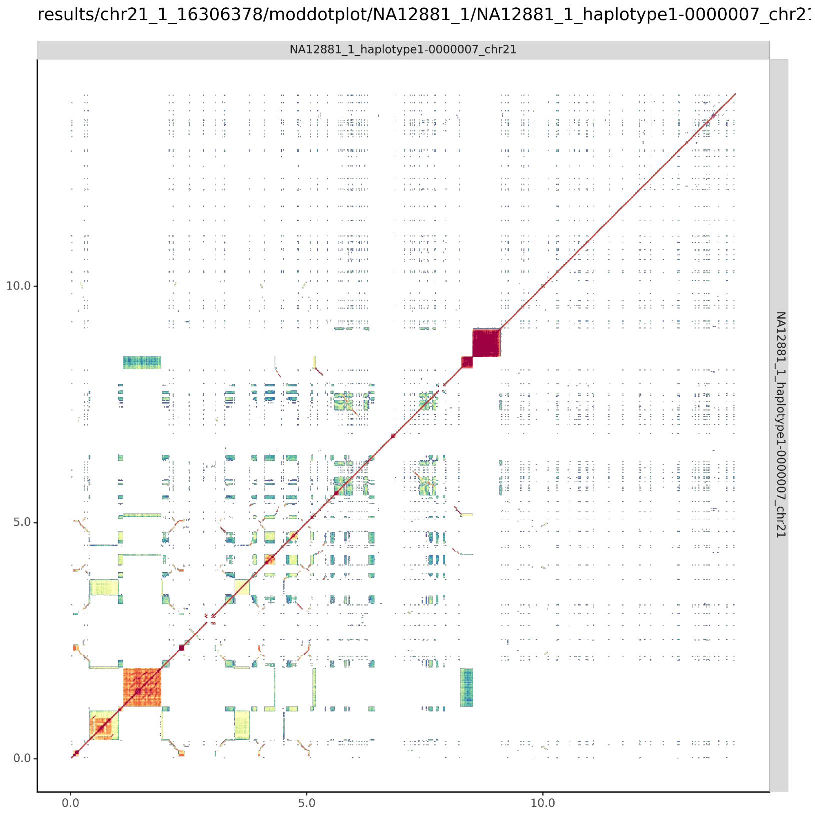

NA12881_2_haplotype2-0000058_chr21

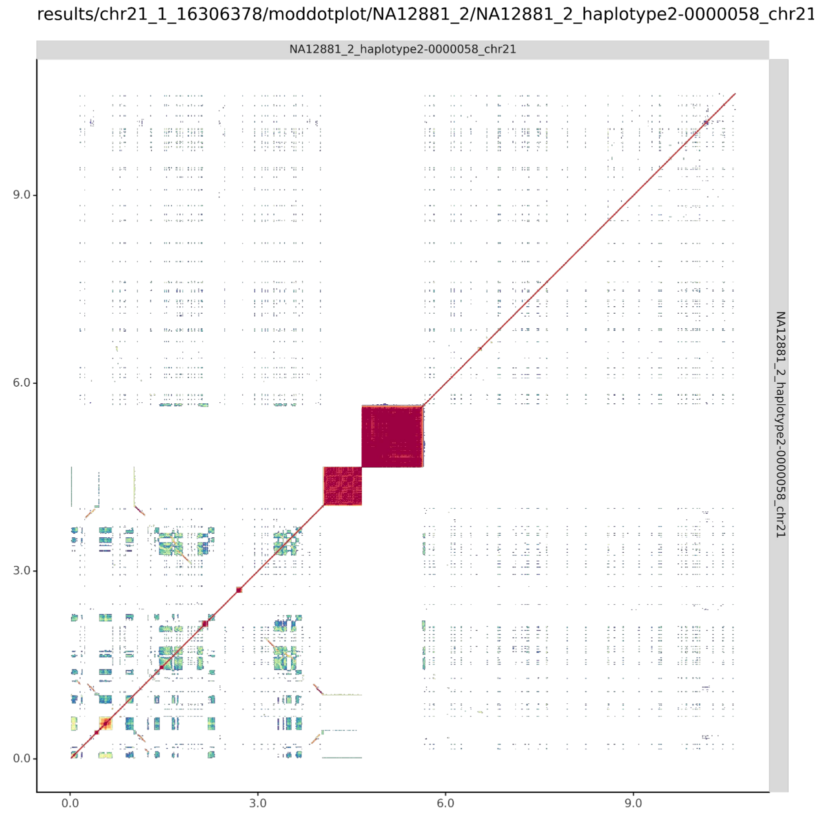

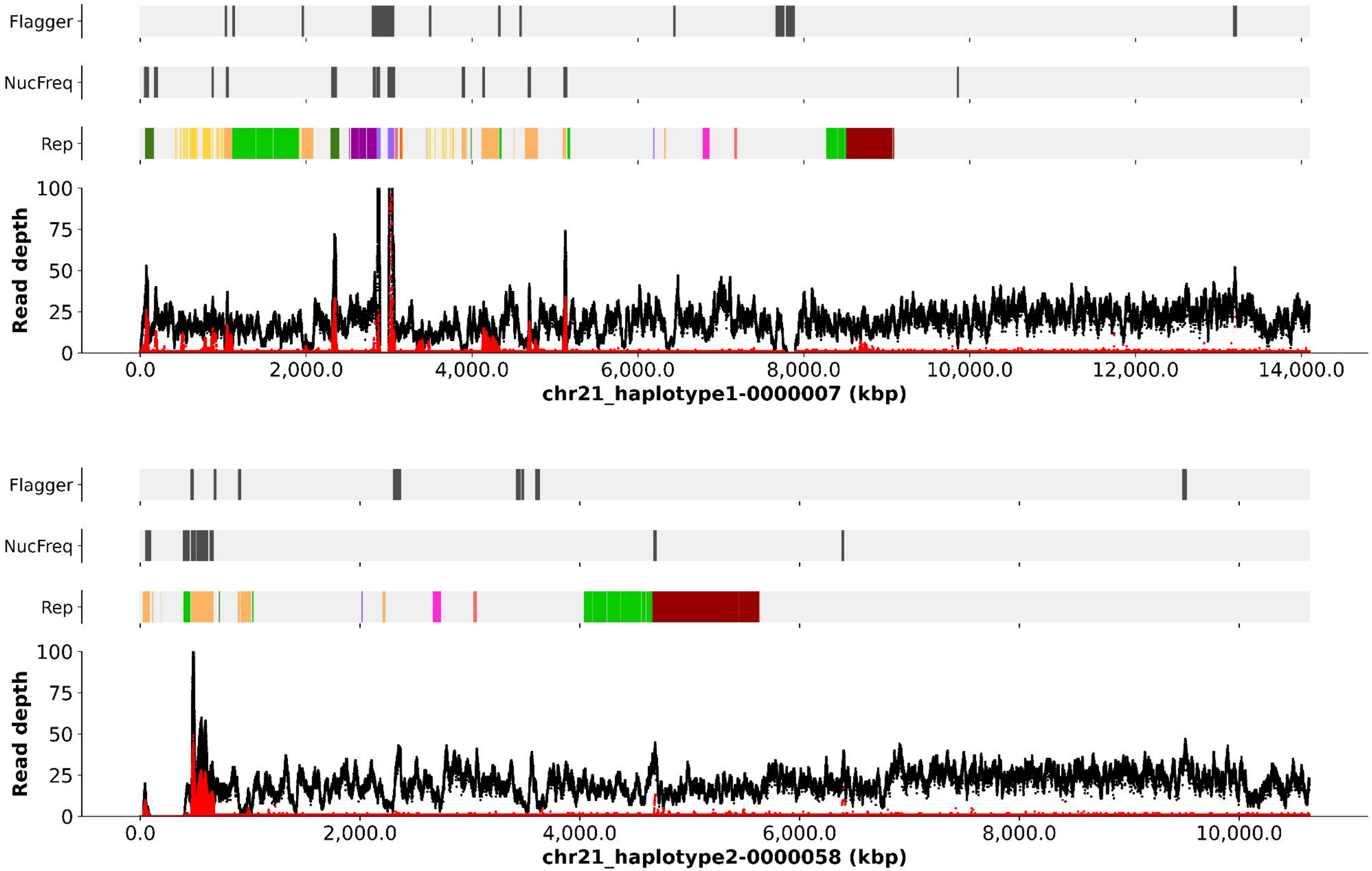

Flagger
NucFreq
Rep
Read depth
100
75
50
25
0
0.0
2,000.0
4,000.0
6,000.0
8,000.0
10,000.0
12,000.0
14,000.0
chr21_haplotype1-0000007 (kbp)
Flagger
NucFreq
Rep
Read depth
100
75
50
25
0
0.0
2,000.0
4,000.0
6,000.0
8,000.0
10,000.0
chr21_haplotype2-0000058 (kbp)

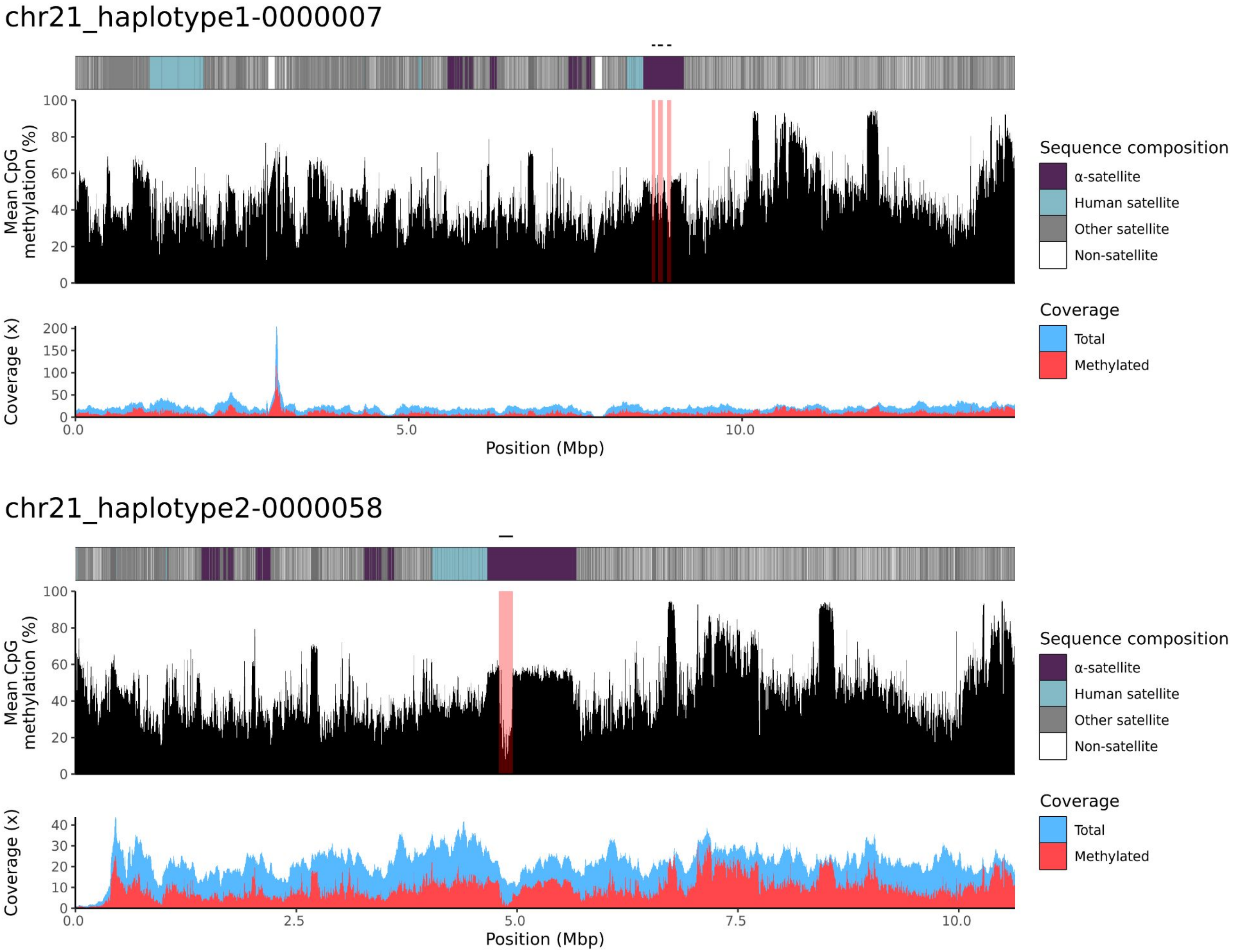
chr21_haplotype1-0000007
Mean CpG methylation (%)
Coverage (x)
Position (Mbp)
0.0
5.0
10.0
Sequence composition
α-satellite
Human satellite
Other satellite
Non-satellite
Coverage
Total
Methylated
chr21_haplotype2-0000058
Mean CpG methylation (%)
Coverage (x)
Position (Mbp)
0.0
2.5
5.0
7.5
10.0
Sequence composition
α-satellite
Human satellite
Other satellite
Non-satellite
Coverage
Total
Methylated

chr22

NA12881_1_haplotype1-0000024_chr22

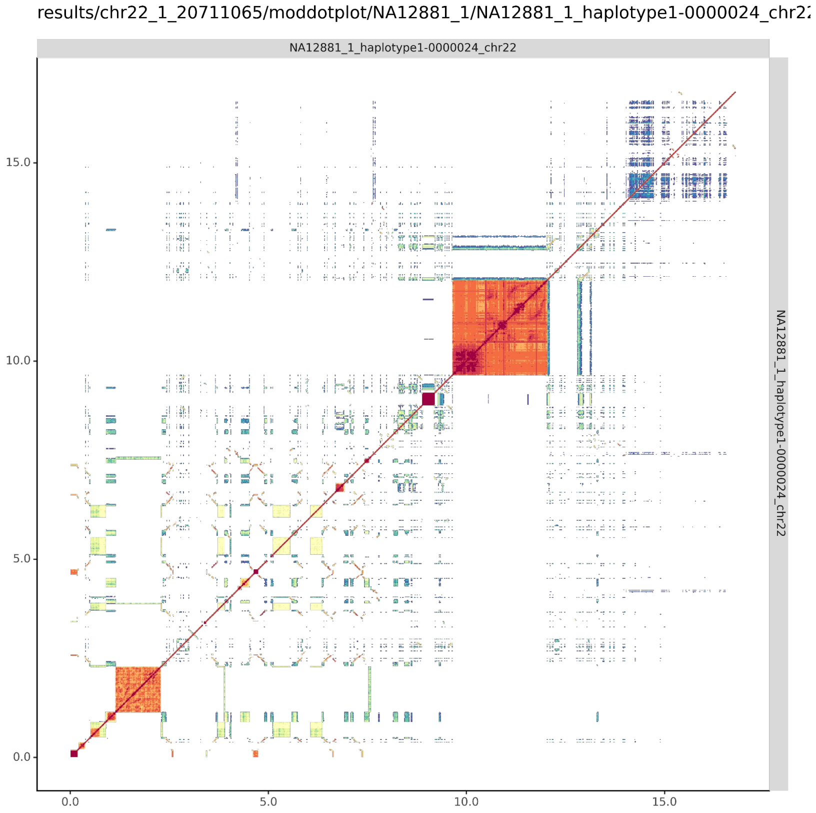

NA12881_2_haplotype2-0000077_chr22

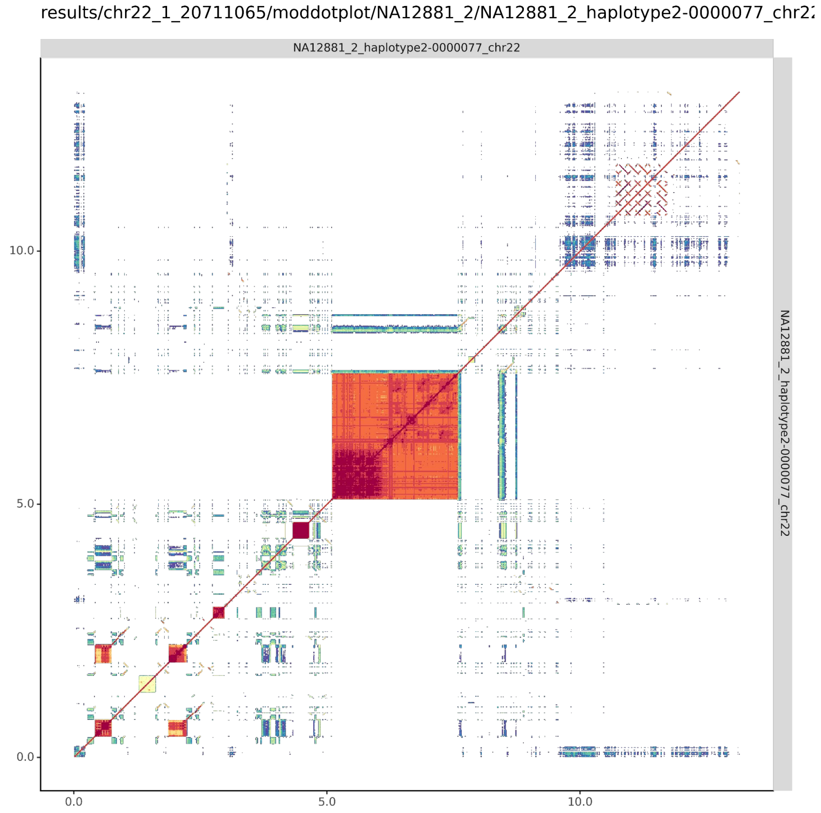

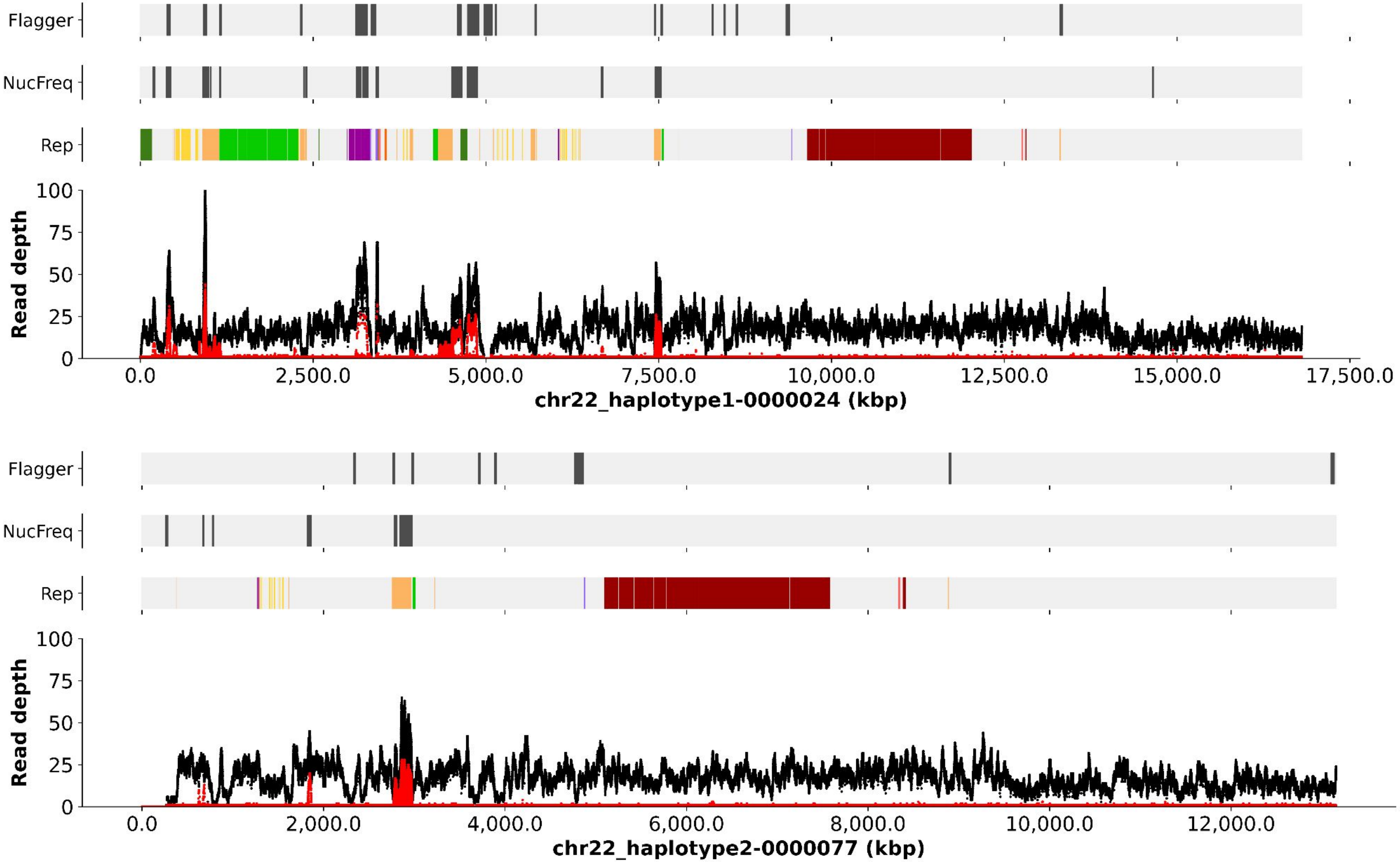

Flagger
NucFreq
Rep
Read depth
100
75
50
25
0
0.0
2,500.0
5,000.0
7,500.0
10,000.0
12,500.0
15,000.0
17,500.0
chr22_haplotype1-0000024 (kbp)
Flagger
NucFreq
Rep
Read depth
100
75
50
25
0
0.0
2,000.0
4,000.0
6,000.0
8,000.0
10,000.0
12,000.0
chr22_haplotype2-0000077 (kbp)

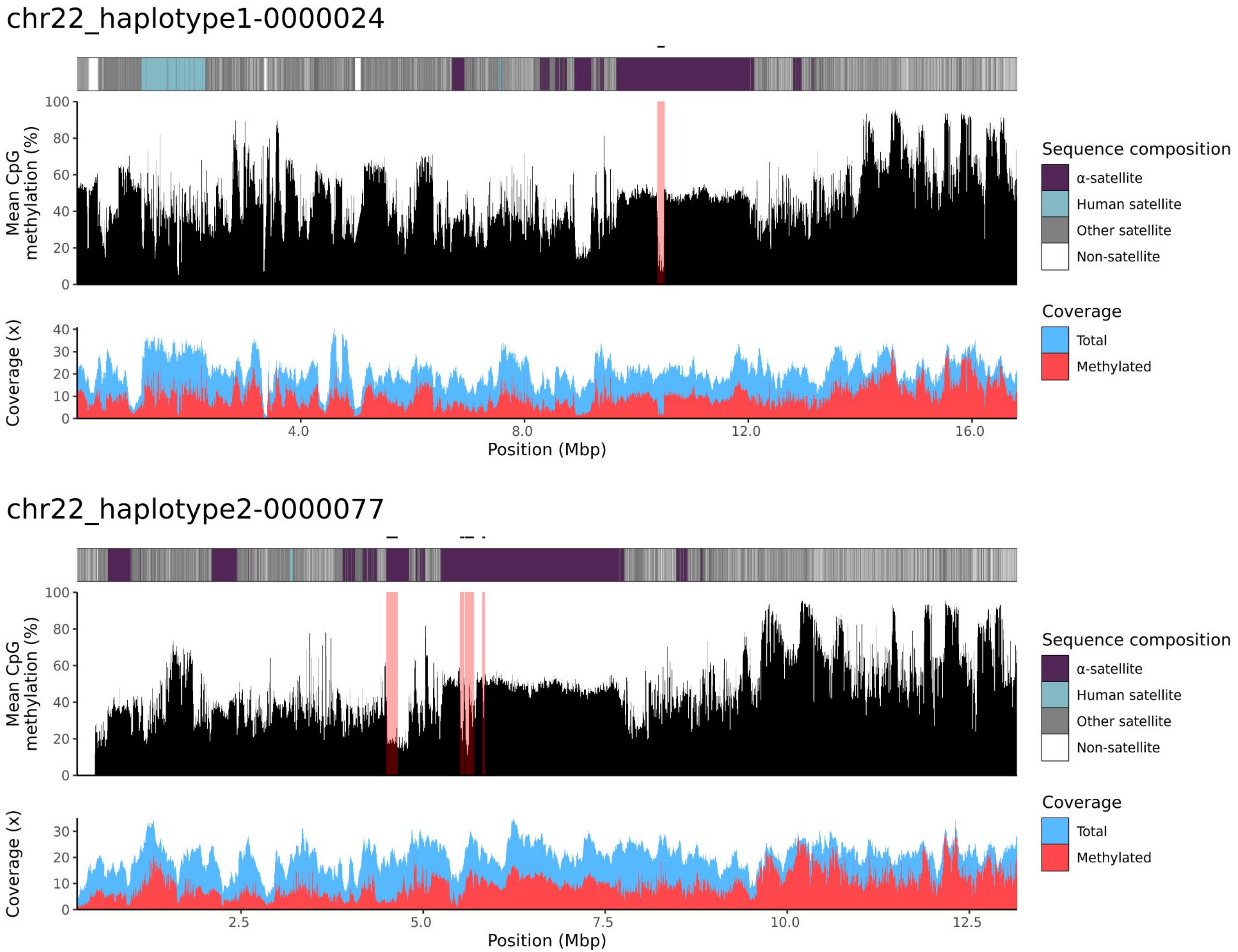
chr22_haplotype1-0000024
Mean CpG methylation (%)
Coverage (x)
Position (Mbp)
4.0
8.0
12.0
16.0
Sequence composition
α-satellite
Human satellite
Other satellite
Non-satellite
Coverage
Total
Methylated
chr22_haplotype2-0000077
Mean CpG methylation (%)
Coverage (x)
Position (Mbp)
2.5
5.0
7.5
10.0
12.5
Sequence composition
α-satellite
Human satellite
Other satellite
Non-satellite
Coverage
Total
Methylated

NA12882

chr13

NA12882_1_haplotype1-0000028_chr13

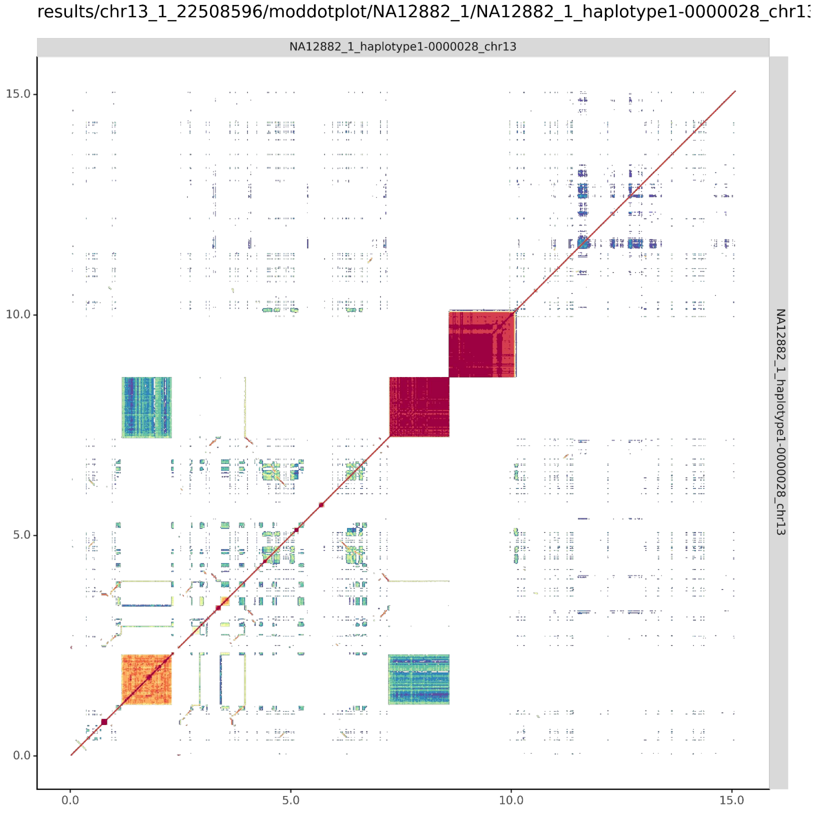

NA12882_2_haplotype2-0000122_chr13

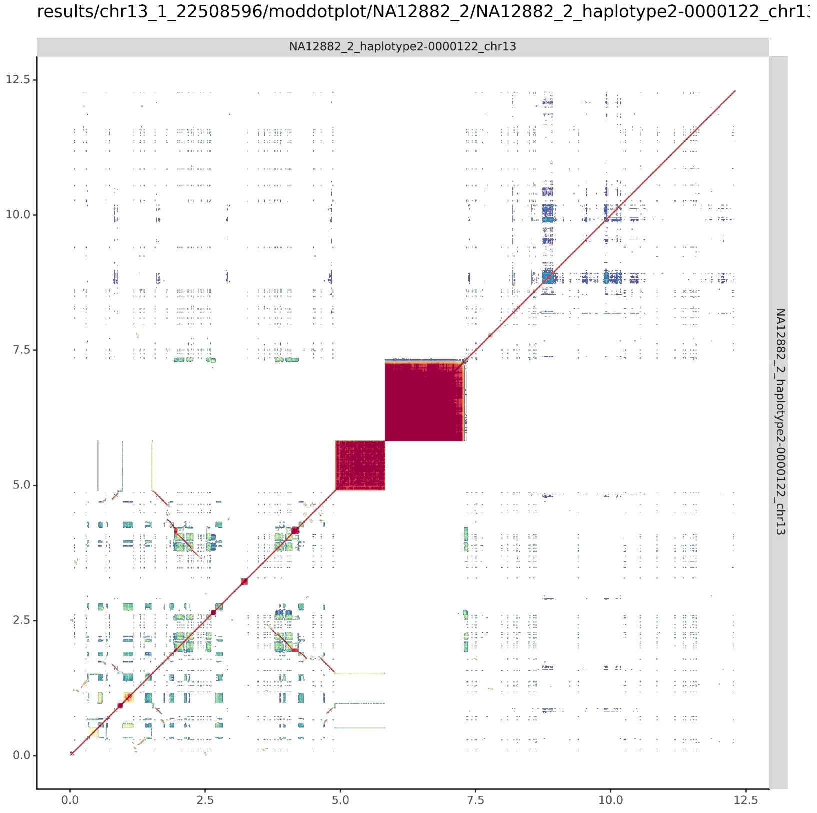

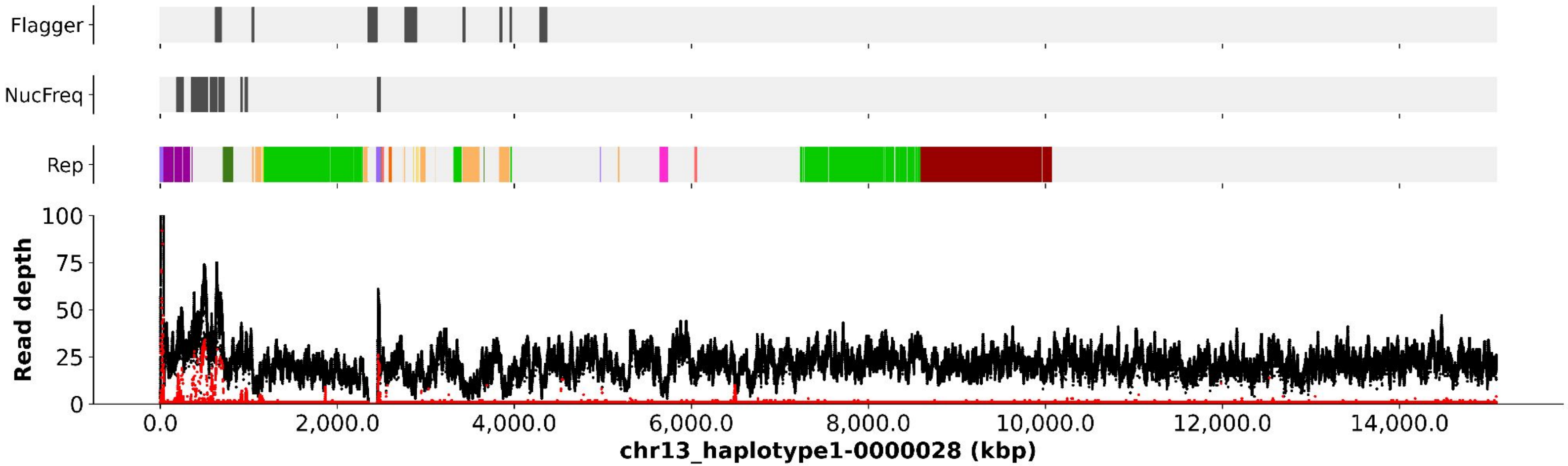

Flagger
NucFreq
Rep
Read depth
100
75
50
25
0
0.0
2,000.0
4,000.0
6,000.0
8,000.0
10,000.0
12,000.0
14,000.0
chr13_haplotype1-0000028 (kbp)

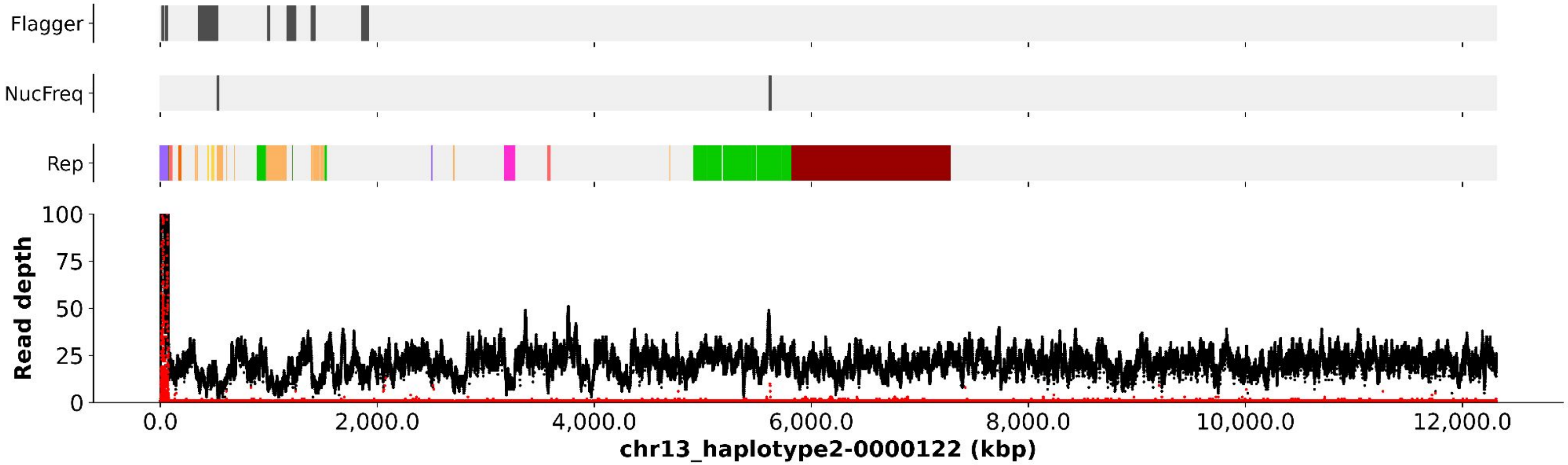

Flagger
NucFreq
Rep
Read depth
100
75
50
25
0
0.0
2,000.0
4,000.0
6,000.0
8,000.0
10,000.0
12,000.0
chr13_haplotype2-0000122 (kbp)

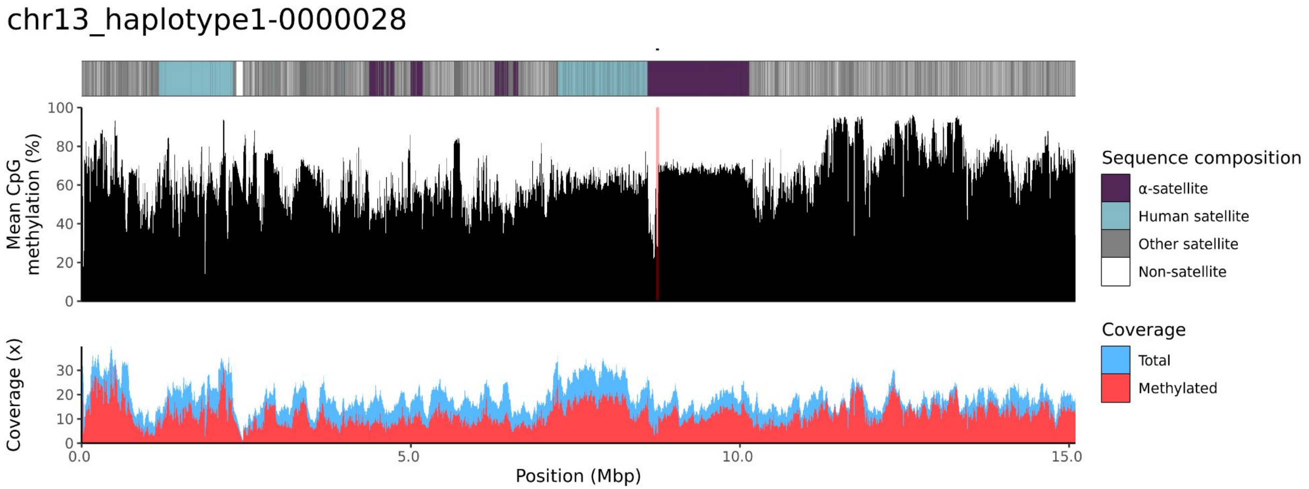
chr13_haplotype1-0000028
Mean CpG methylation (%)
100
80
60
40
20
0
Coverage (x)
30
20
10
0
0.0
5.0
10.0
15.0
Position (Mbp)
Sequence composition
α-satellite
Human satellite
Other satellite
Non-satellite
Coverage
Total
Methylated

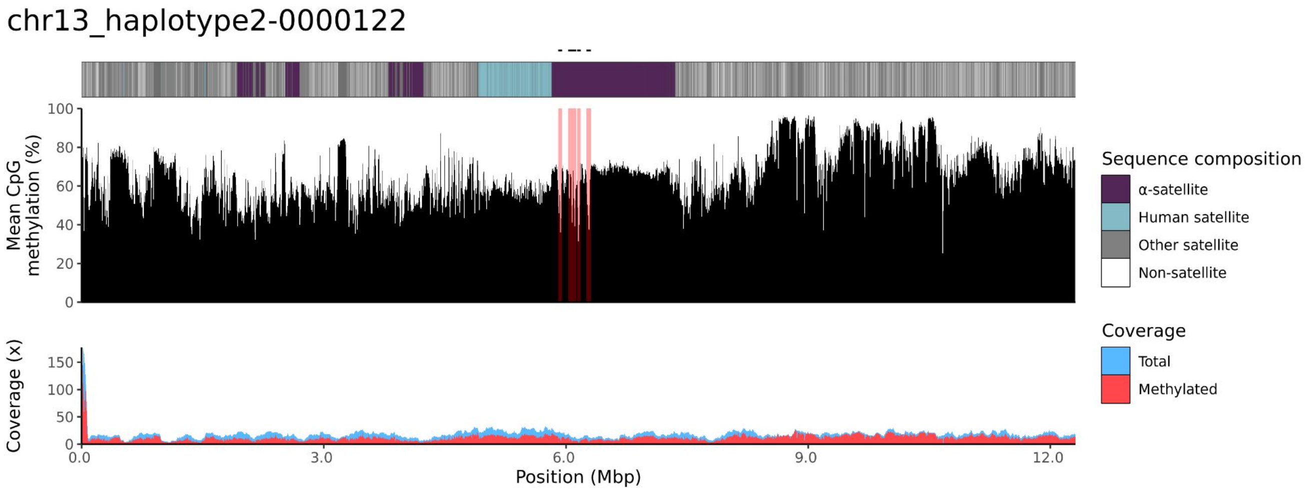
chr13_haplotype2-0000122
Mean CpG methylation (%)
100
80
60
40
20
0
Coverage (x)
150
100
50
0
0.0
3.0
6.0
9.0
12.0
Position (Mbp)
Sequence composition
α-satellite
Human satellite
Other satellite
Non-satellite
Coverage
Total
Methylated

chr14

NA12882_1_haplotype1-0000016_chr14

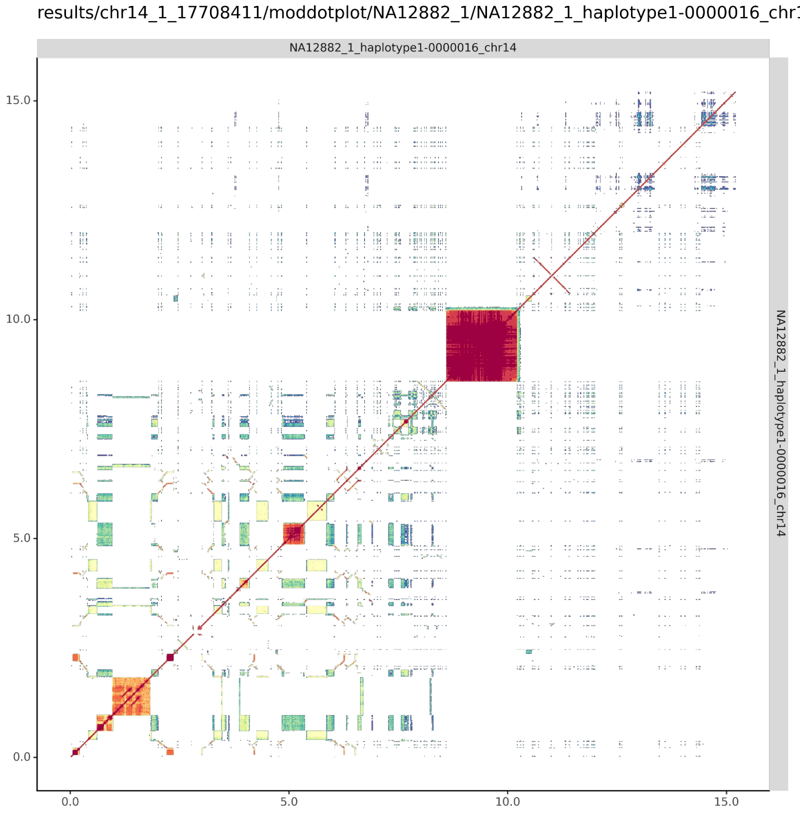

NA12882_2_haplotype2-0000112_chr14

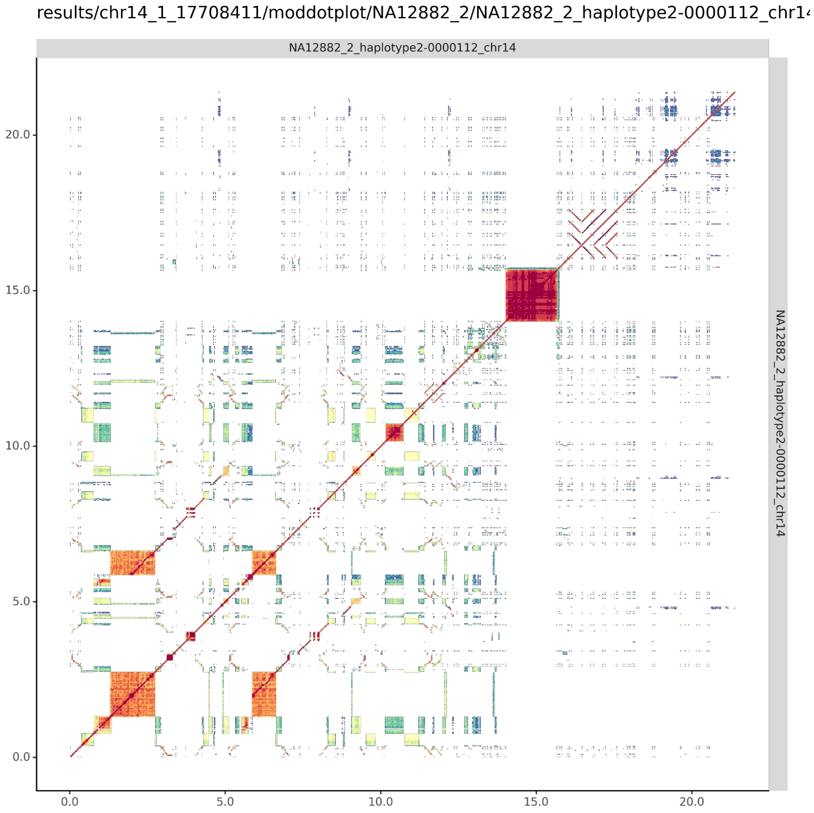

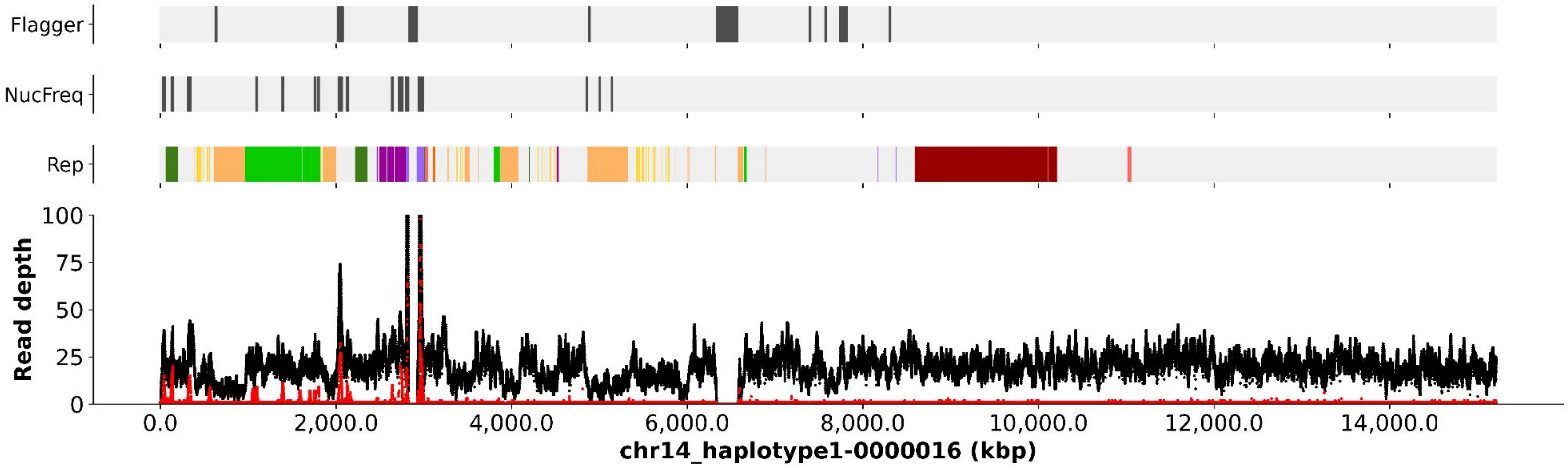

Flagger
NucFreq
Rep
Read depth
100
75
50
25
0
0.0
2,000.0
4,000.0
6,000.0
8,000.0
10,000.0
12,000.0
14,000.0
chr14_haplotype1-0000016 (kbp)

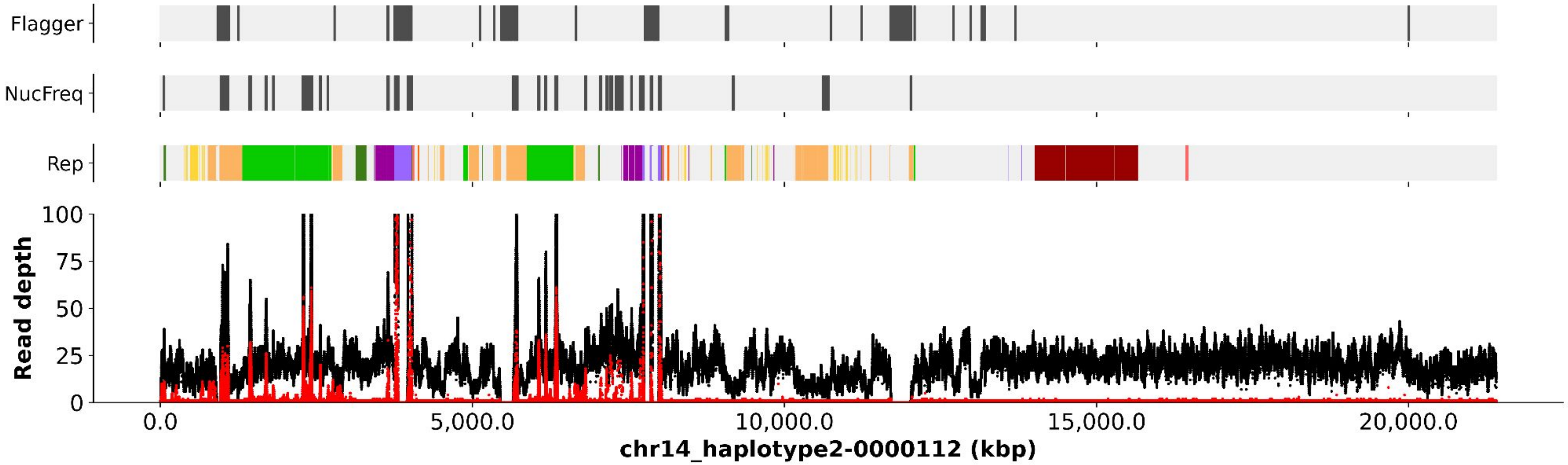

Flagger
NucFreq
Rep
Read depth
100
75
50
25
0
0.0
5,000.0
10,000.0
15,000.0
20,000.0
chr14_haplotype2-0000112 (kbp)

chr14_haplotype1-0000016

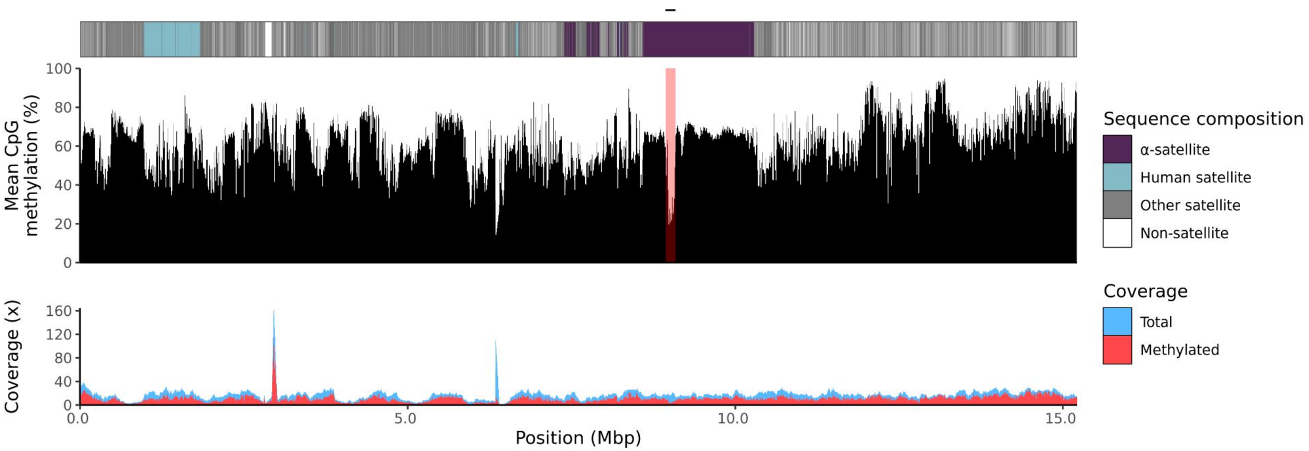

chr14_haplotype2-0000112

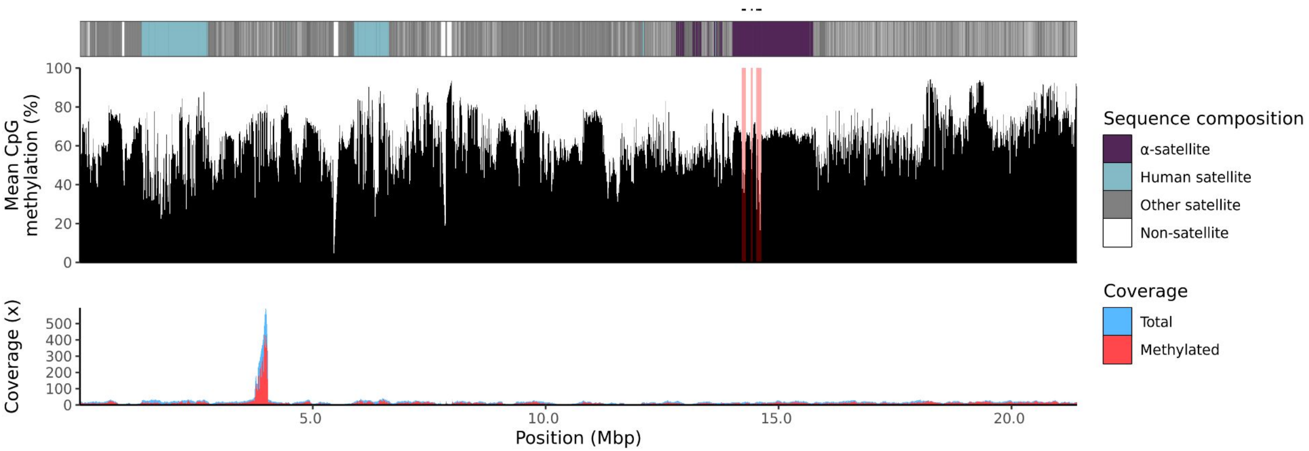

chr15

NA12882_1_haplotype1-0000019_chr15

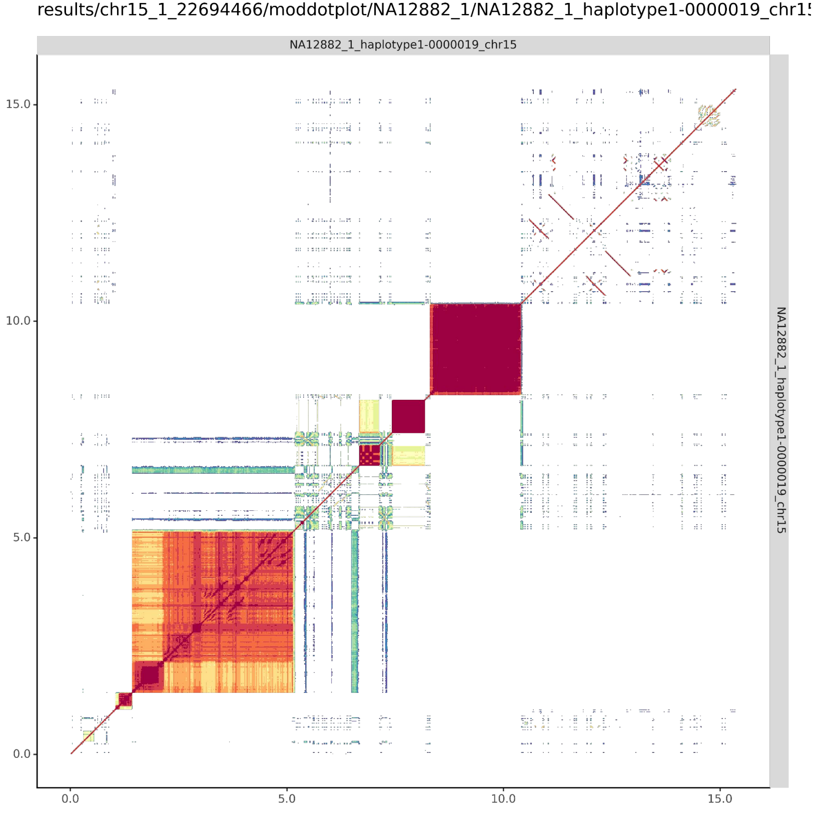

NA12882_2_haplotype2-0000116_chr15

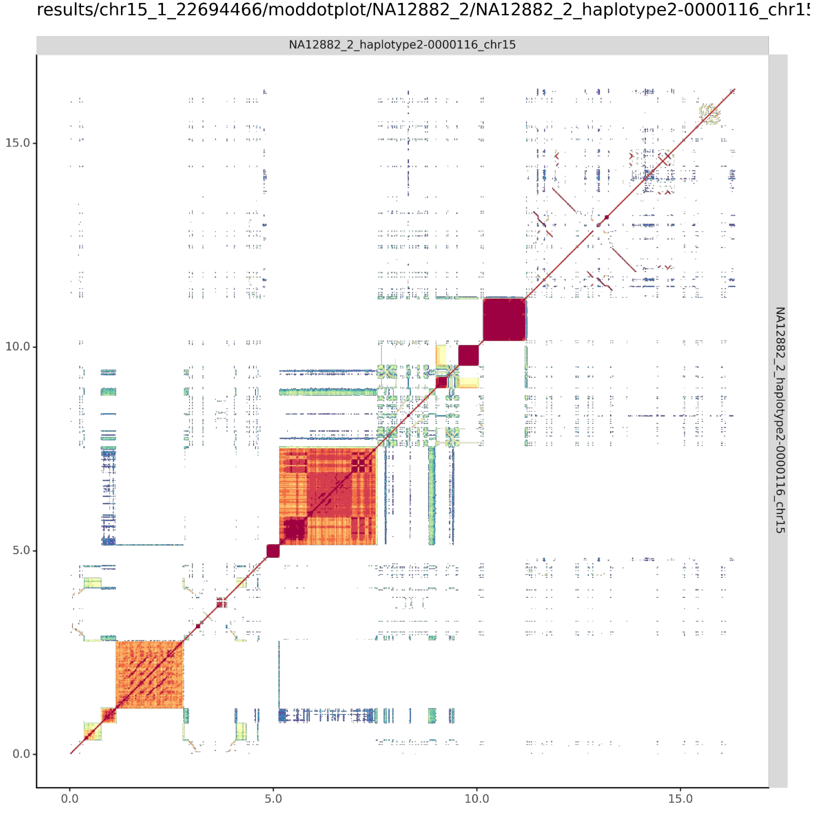

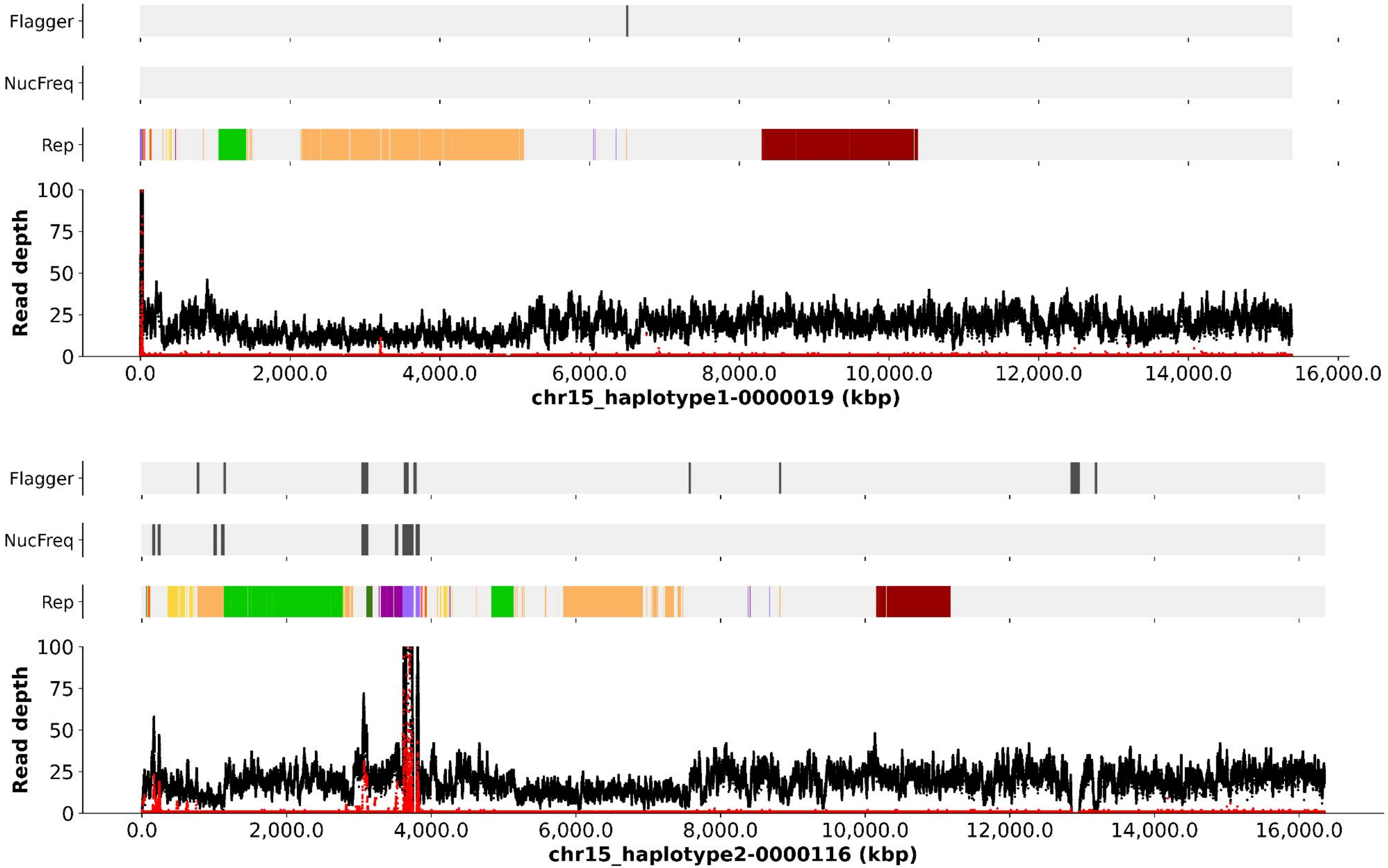
Flagger
NucFreq
Rep
Read depth
100
75
50
25
0
0.0
2,000.0
4,000.0
6,000.0
8,000.0
10,000.0
12,000.0
14,000.0
16,000.0
chr15_haplotype1-0000019 (kbp)
chr15_haplotype2-0000116 (kbp)

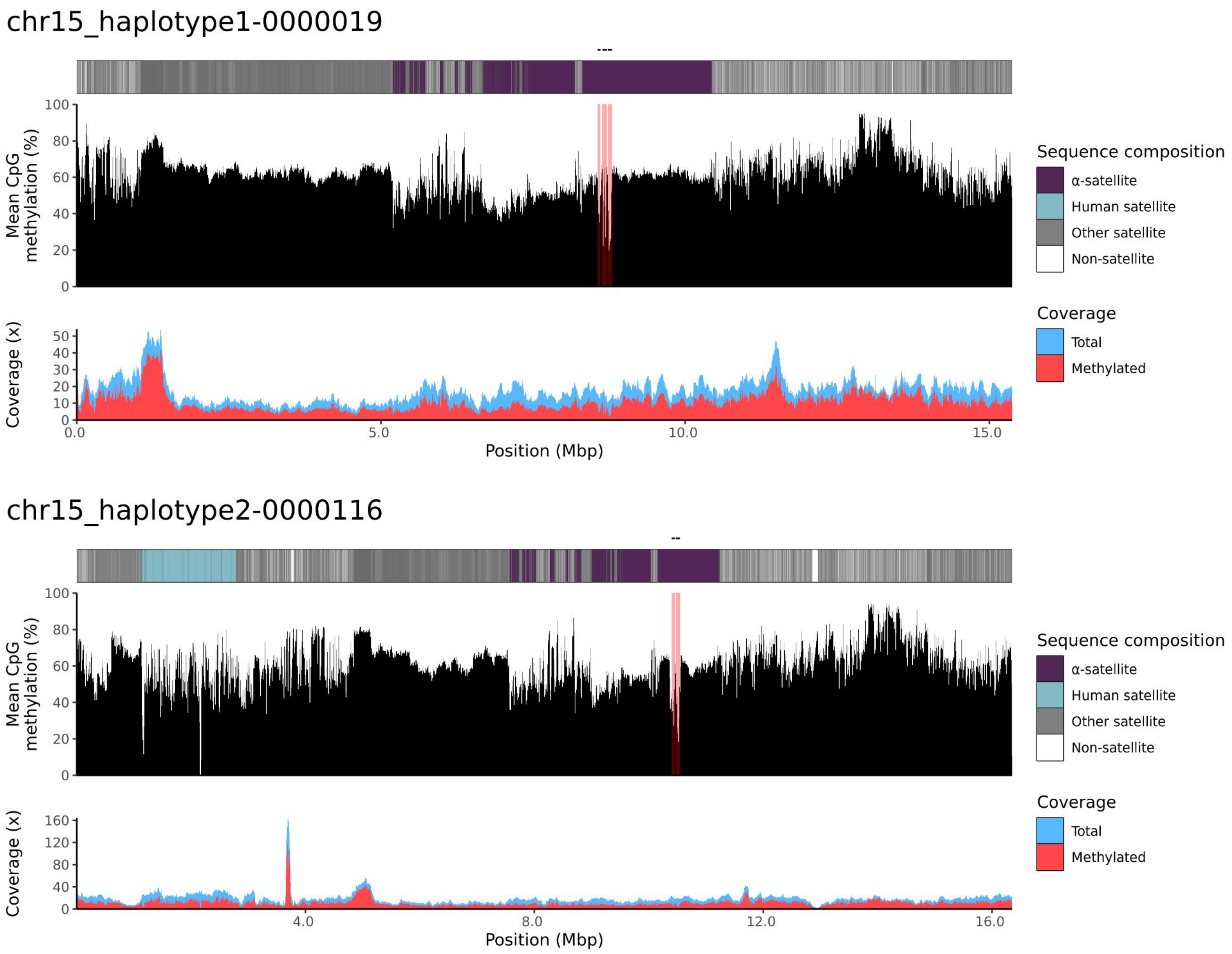

chr15_haplotype1-0000019
Mean CpG methylation (%)
Coverage (x)
Position (Mbp)
Sequence composition
α-satellite
Human satellite
Other satellite
Non-satellite
Coverage
Total
Methylated
chr15_haplotype2-0000116
Mean CpG methylation (%)
Coverage (x)
Position (Mbp)
Sequence composition
α-satellite
Human satellite
Other satellite
Non-satellite
Coverage
Total
Methylated

chr21

NA12882_1_haplotype1-0000010_chr21

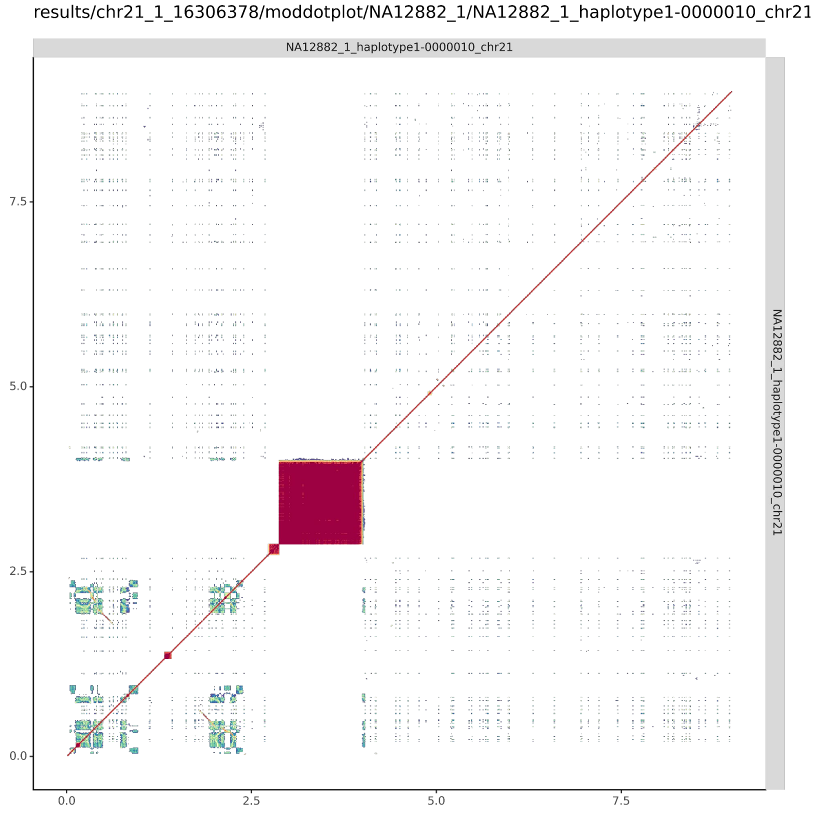

NA12882_2_haplotype2-0000107_chr21

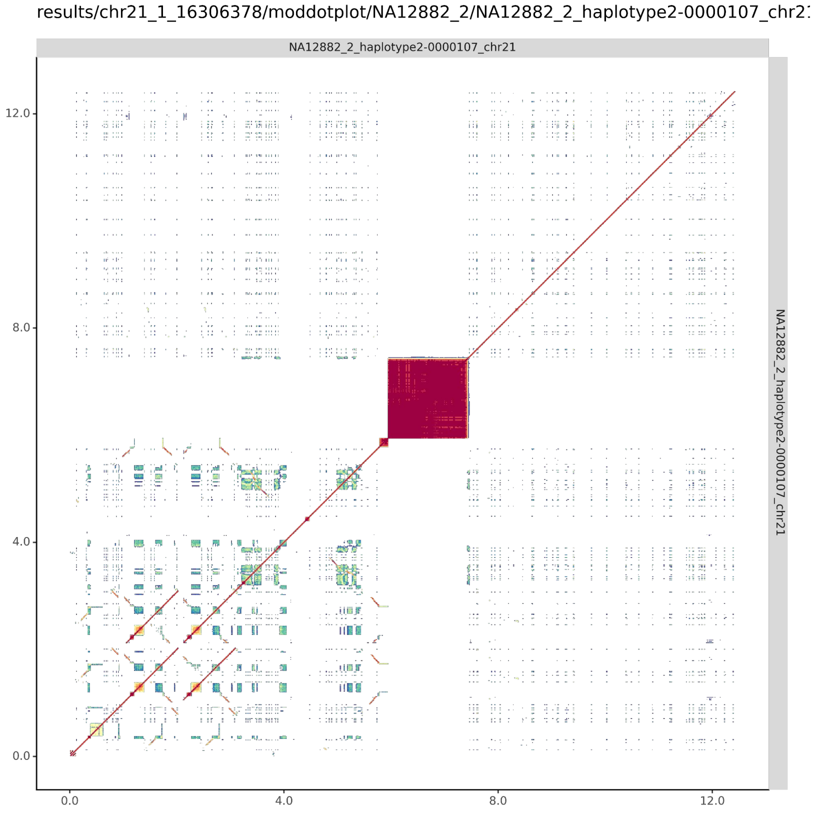

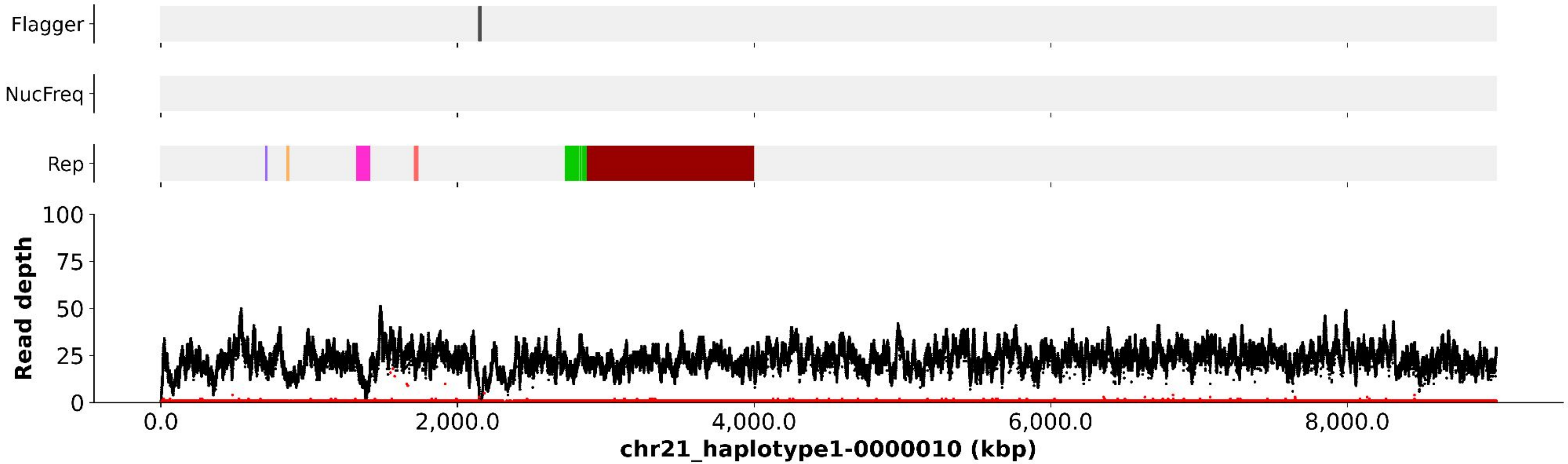
Flagger
NucFreq
Rep
Read depth
100
75
50
25
0
0.0
2,000.0
4,000.0
6,000.0
8,000.0
chr21_haplotype1-0000010 (kbp)

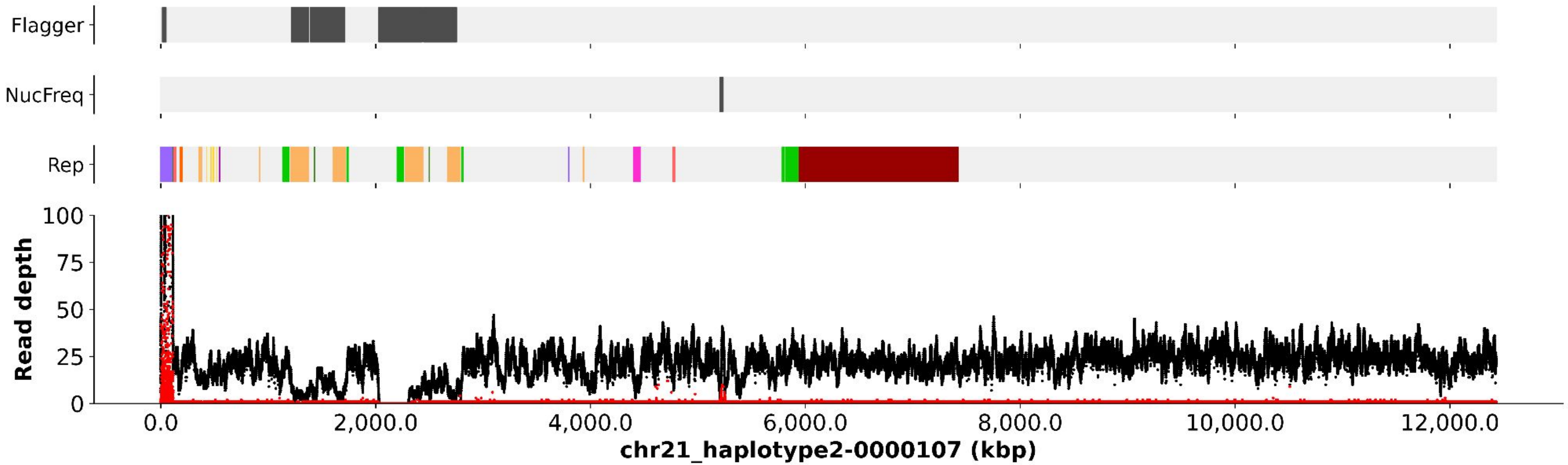
Flagger
NucFreq
Rep
Read depth
100
75
50
25
0
0.0
2,000.0
4,000.0
6,000.0
8,000.0
10,000.0
12,000.0
chr21_haplotype2-0000107 (kbp)

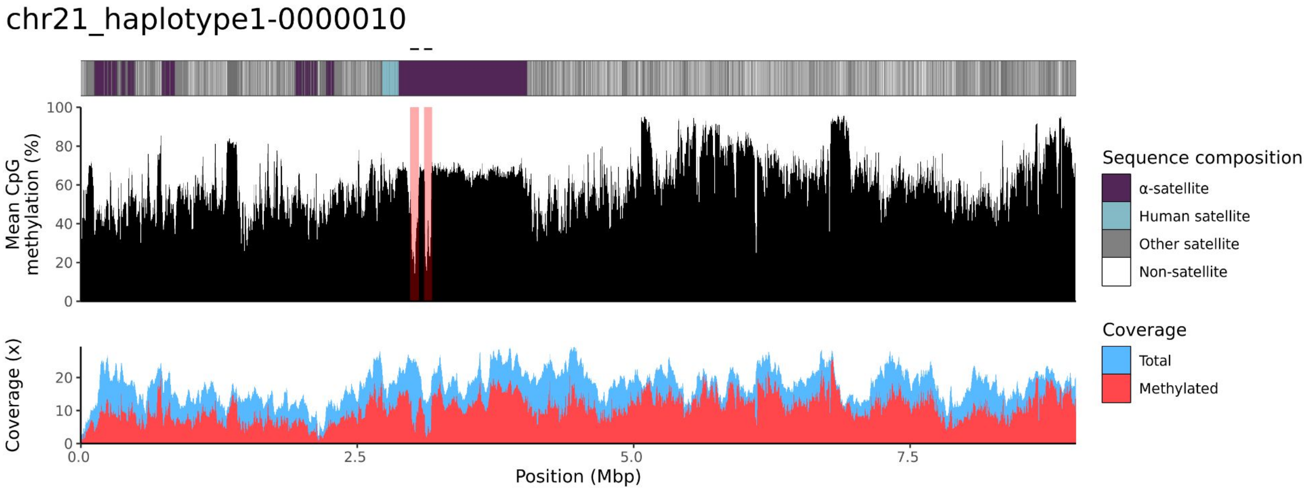
chr21_haplotype1-0000010
Mean CpG methylation (%)
Coverage (x)
Position (Mbp)
Sequence composition
α-satellite
Human satellite
Other satellite
Non-satellite
Coverage
Total
Methylated

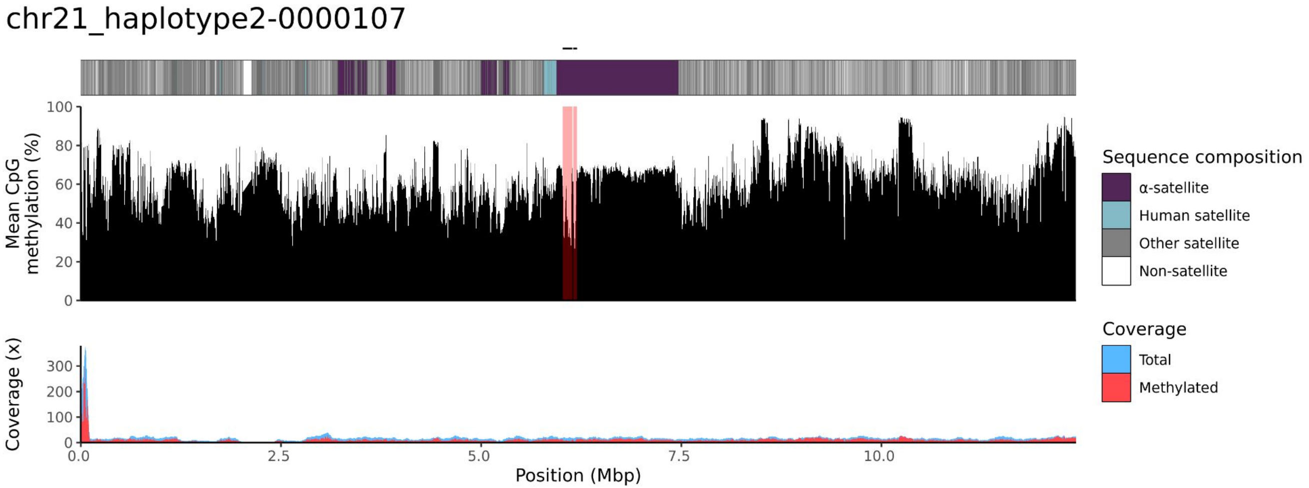
chr21_haplotype2-0000107
Mean CpG methylation (%)
Coverage (x)
Position (Mbp)
Sequence composition
α-satellite
Human satellite
Other satellite
Non-satellite
Coverage
Total
Methylated

chr22

NA12882_1_haplotype1-0000004_chr22

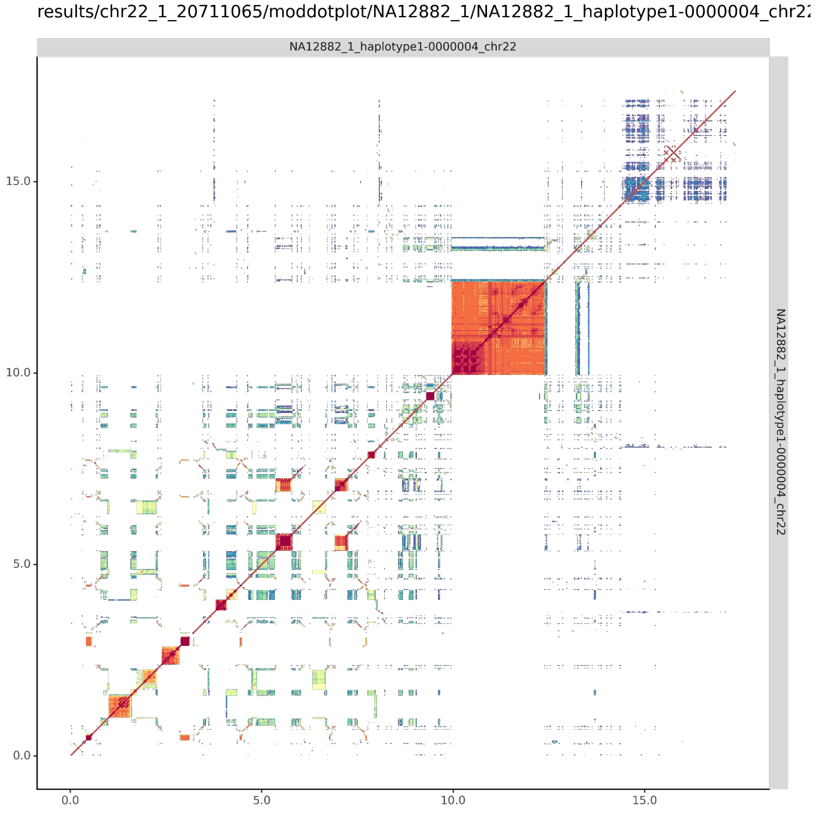

NA12882_2_haplotype2-0000099_chr22

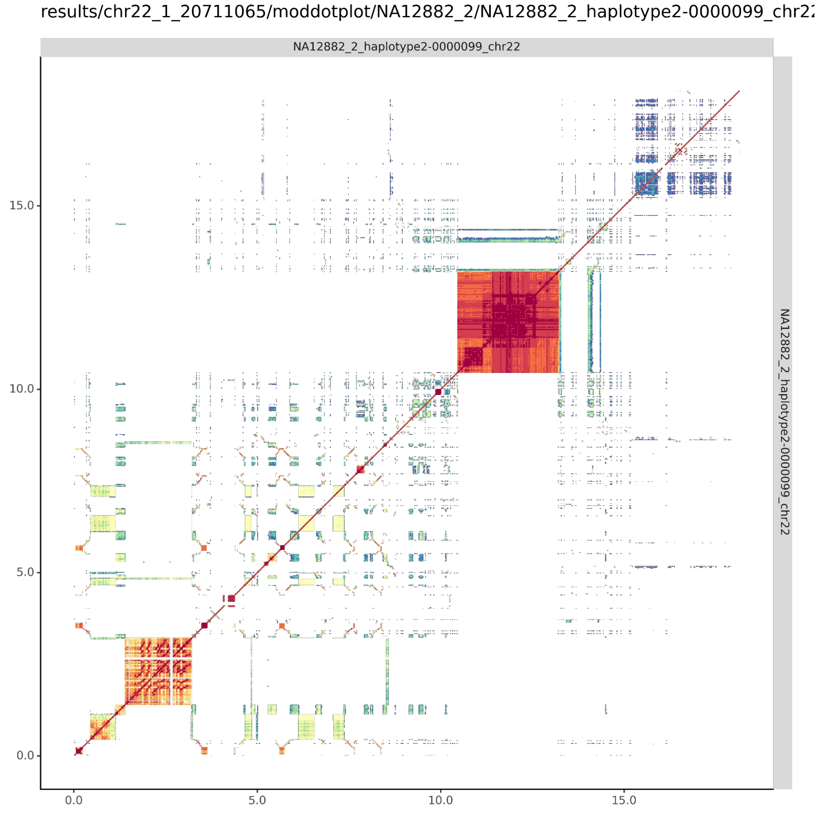

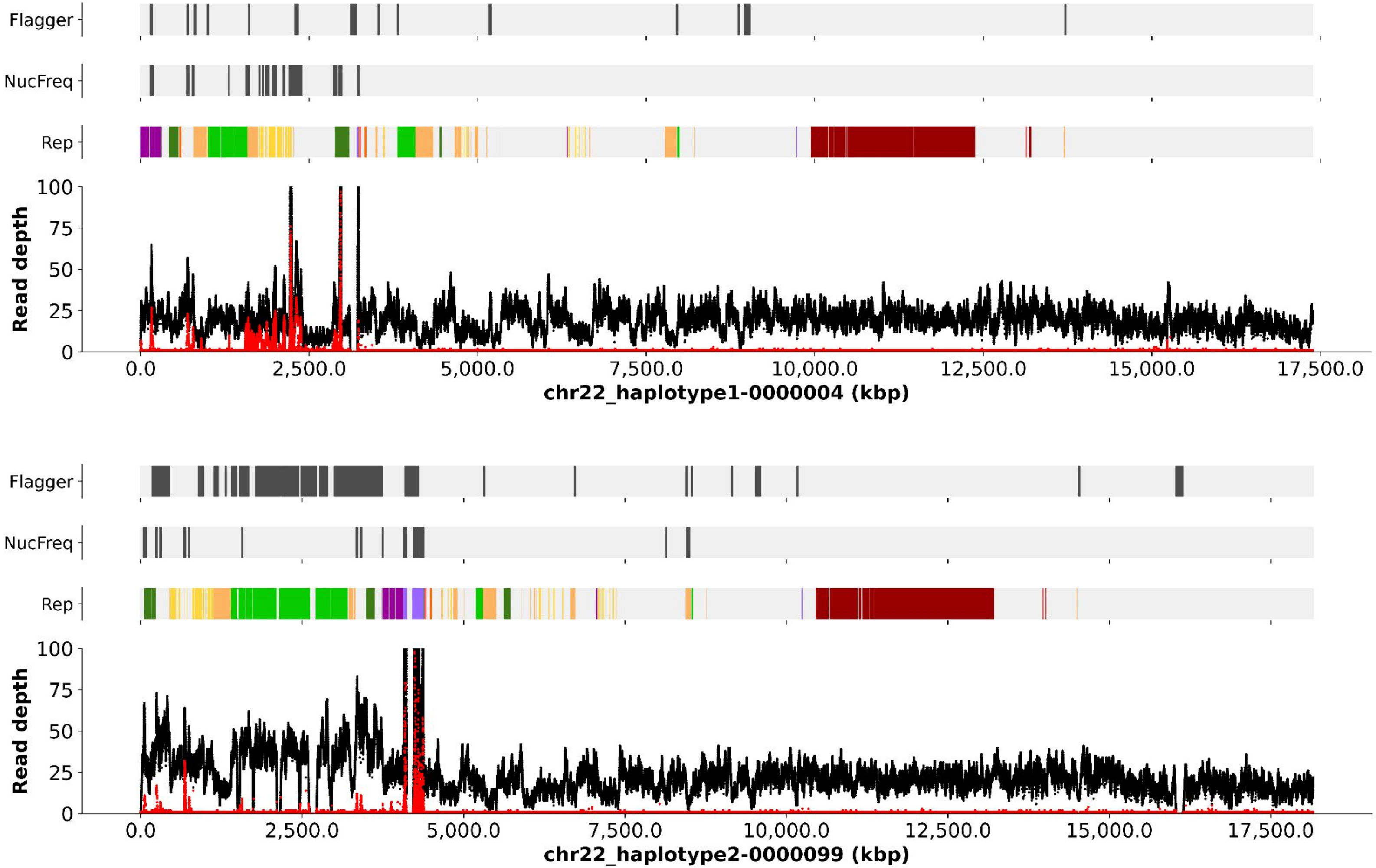
Flagger
NucFreq
Rep
Read depth
100
75
50
25
0
0.0
2,500.0
5,000.0
7,500.0
10,000.0
12,500.0
15,000.0
17,500.0
chr22_haplotype1-0000004 (kbp)
Flagger
NucFreq
Rep
Read depth
100
75
50
25
0
0.0
2,500.0
5,000.0
7,500.0
10,000.0
12,500.0
15,000.0
17,500.0
chr22_haplotype2-0000099 (kbp)

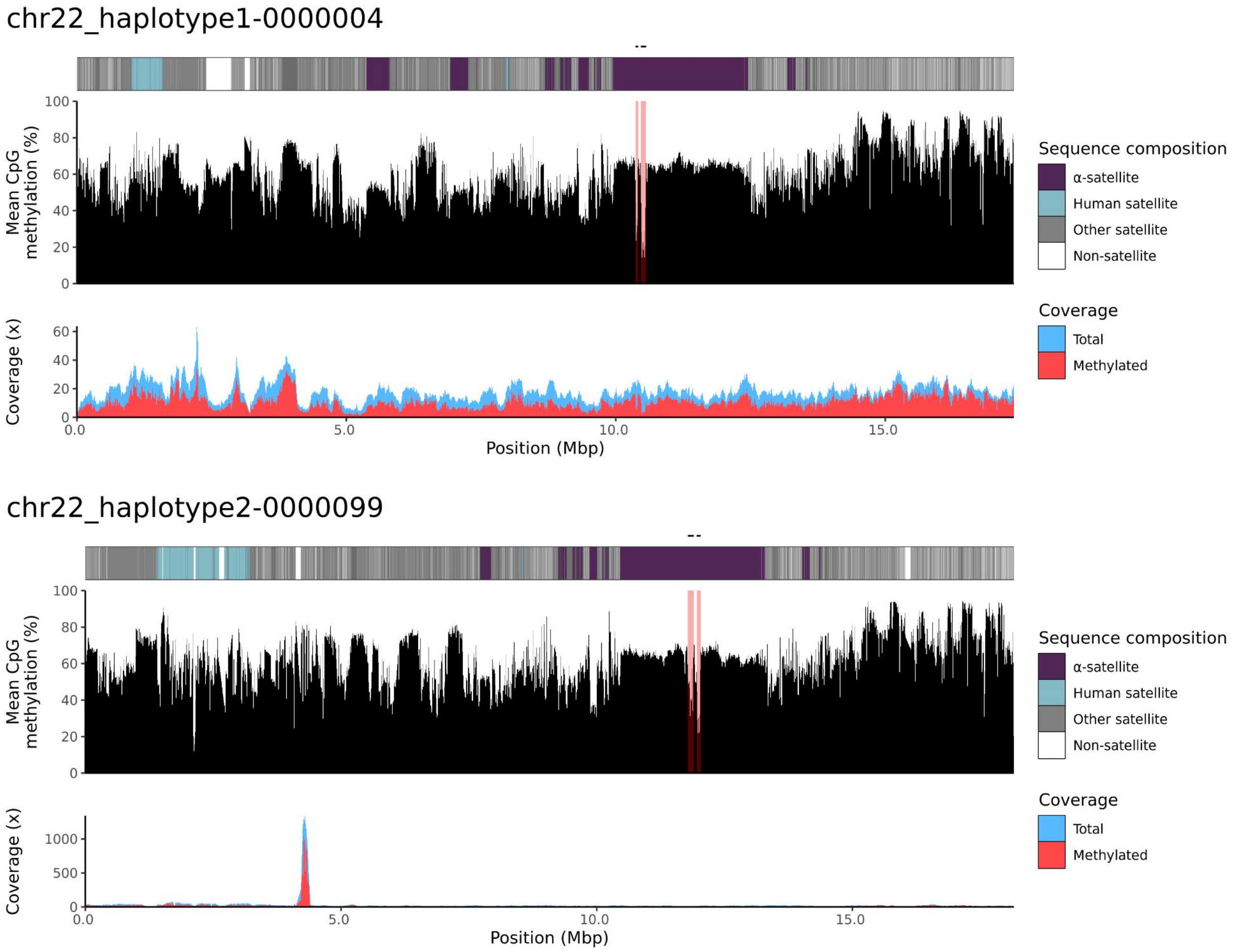

chr22_haplotype1-0000004
Mean CpG methylation (%)
Coverage (x)
Position (Mbp)
Sequence composition
α-satellite
Human satellite
Other satellite
Non-satellite
Coverage
Total
Methylated
chr22_haplotype2-0000099
Mean CpG methylation (%)
Coverage (x)
Position (Mbp)
Sequence composition
α-satellite
Human satellite
Other satellite
Non-satellite
Coverage
Total
Methylated

NA12883

chr13

NA12883_1_haplotype1-0000004_chr13

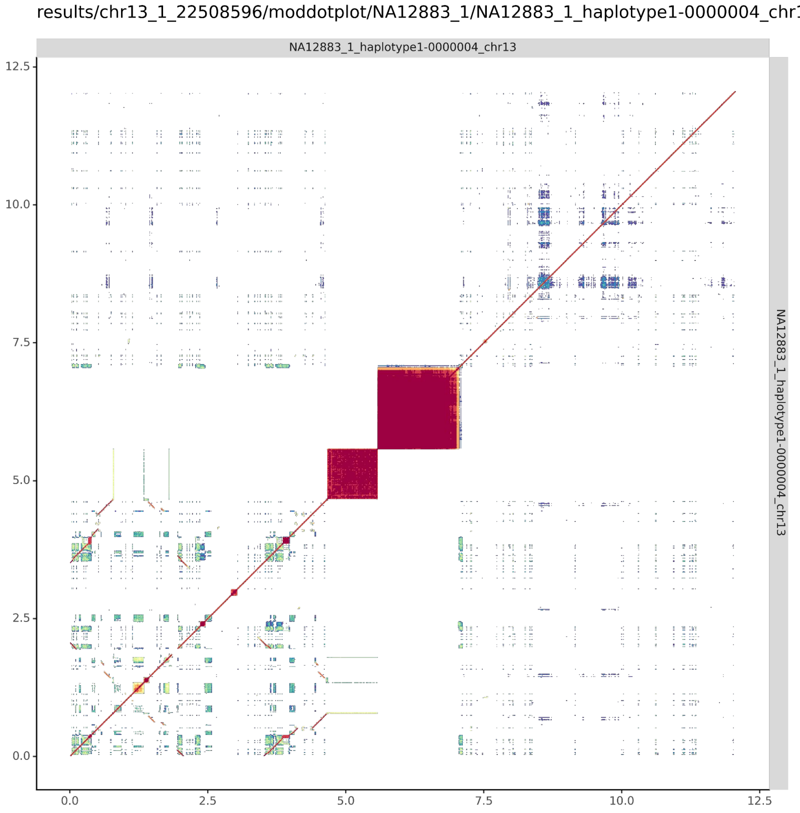

NA12883_2_haplotype2-0000078_chr13

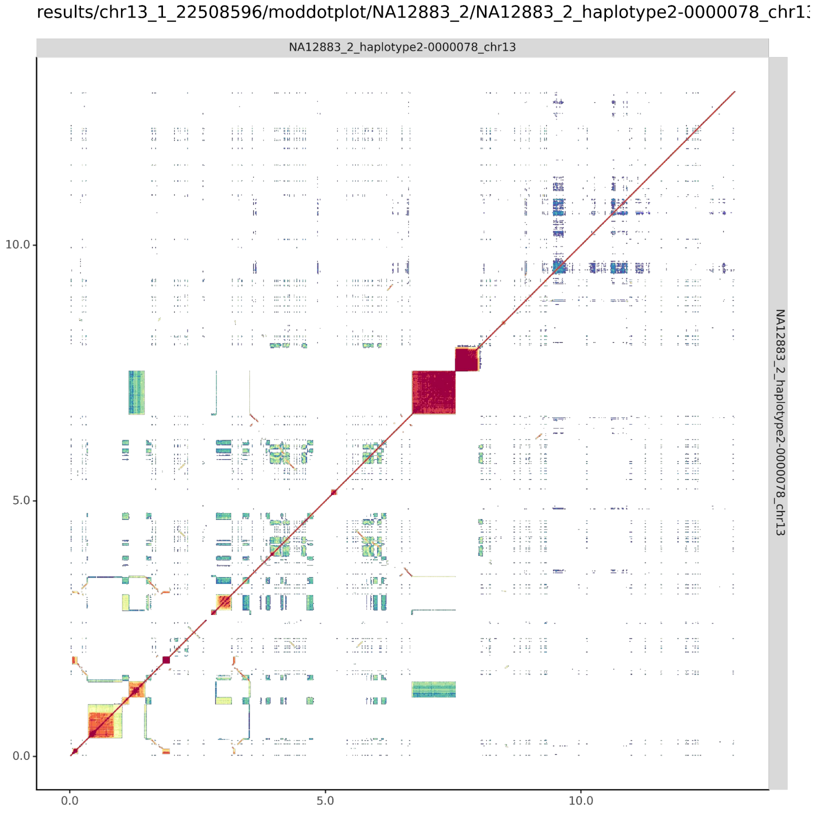

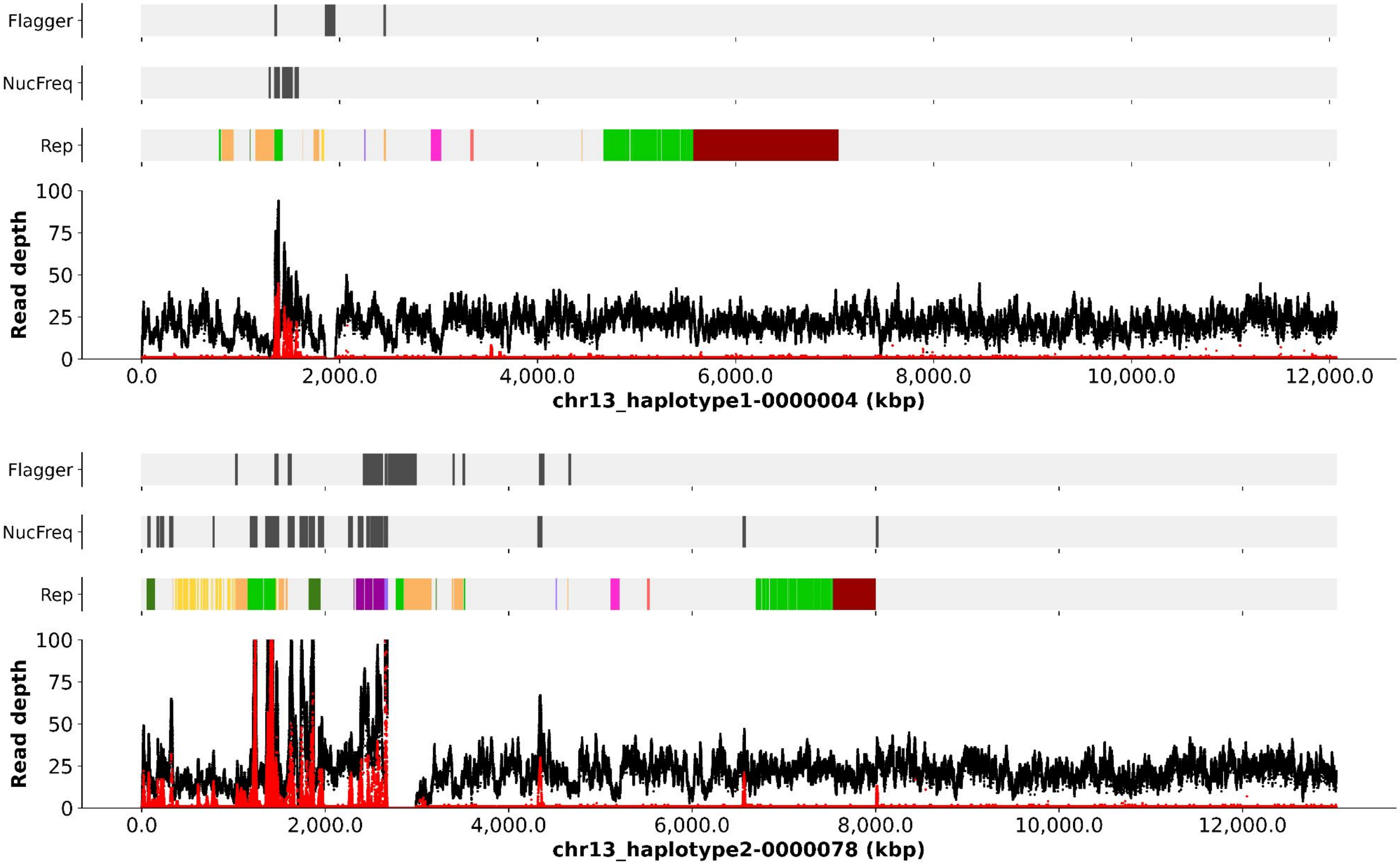

Flagger
NucFreq
Rep
Read depth
100
75
50
25
0
0.0
2,000.0
4,000.0
6,000.0
8,000.0
10,000.0
12,000.0
chr13_haplotype1-0000004 (kbp)
Flagger
NucFreq
Rep
Read depth
100
75
50
25
0
0.0
2,000.0
4,000.0
6,000.0
8,000.0
10,000.0
12,000.0
chr13_haplotype2-0000078 (kbp)

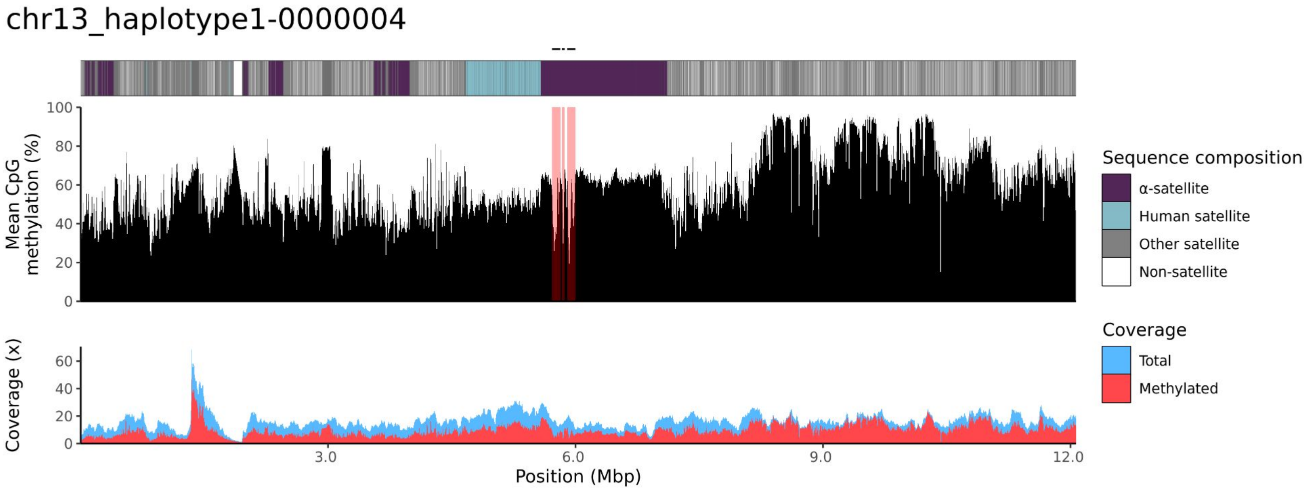
chr13_haplotype1-0000004
Mean CpG methylation (%)
100
80
60
40
20
0
Coverage (x)
60
40
20
0
3.0
6.0
9.0
12.0
Position (Mbp)
Sequence composition
α-satellite
Human satellite
Other satellite
Non-satellite
Coverage
Total
Methylated

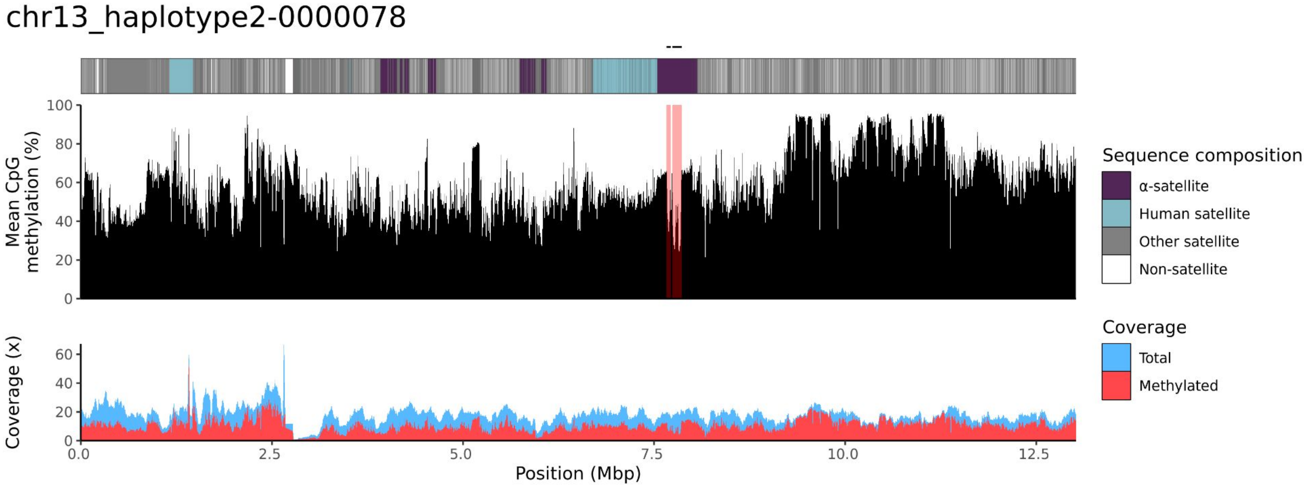
chr13_haplotype2-0000078
Mean CpG methylation (%)
100
80
60
40
20
0
Coverage (x)
60
40
20
0
0.0
2.5
5.0
7.5
10.0
12.5
Position (Mbp)
Sequence composition
α-satellite
Human satellite
Other satellite
Non-satellite
Coverage
Total
Methylated

chr14

NA12883_1_haplotype1-0000025_chr14

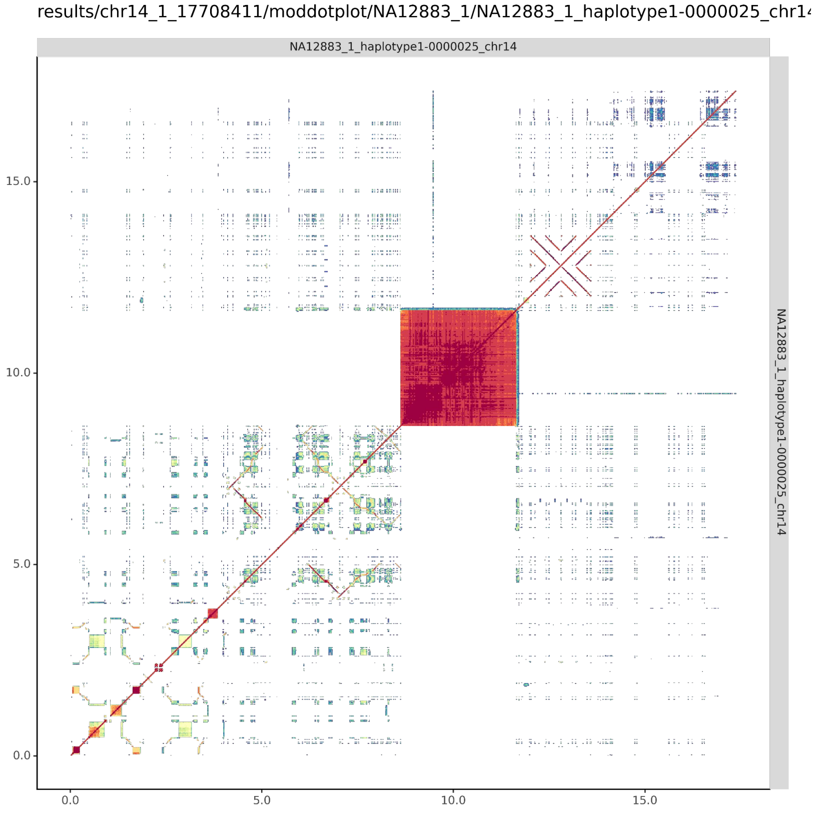

NA12883_2_haplotype2-0000084_chr14

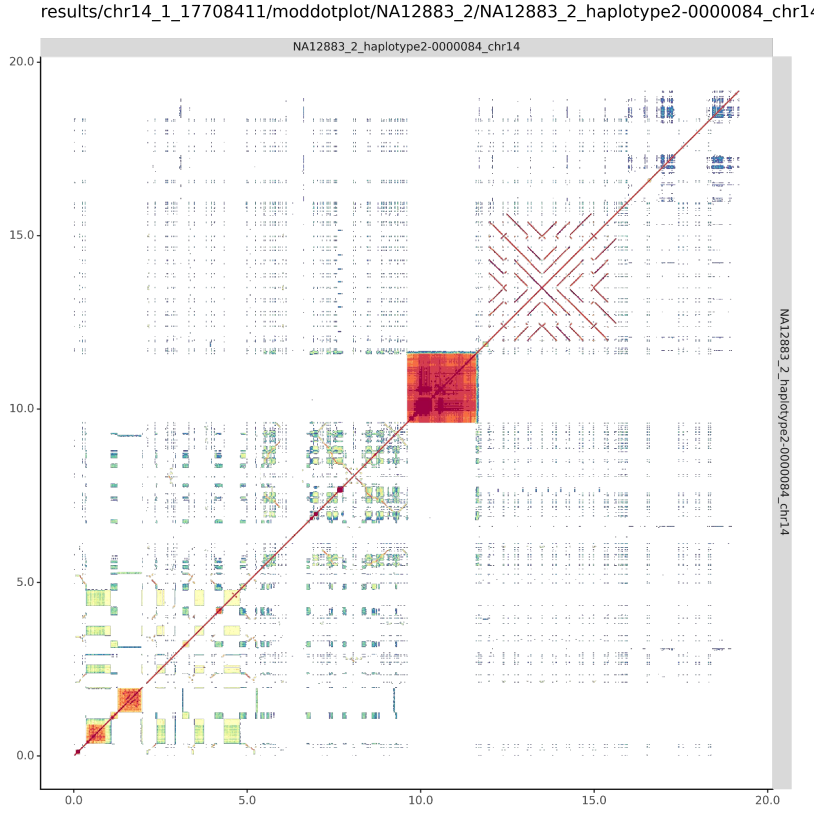

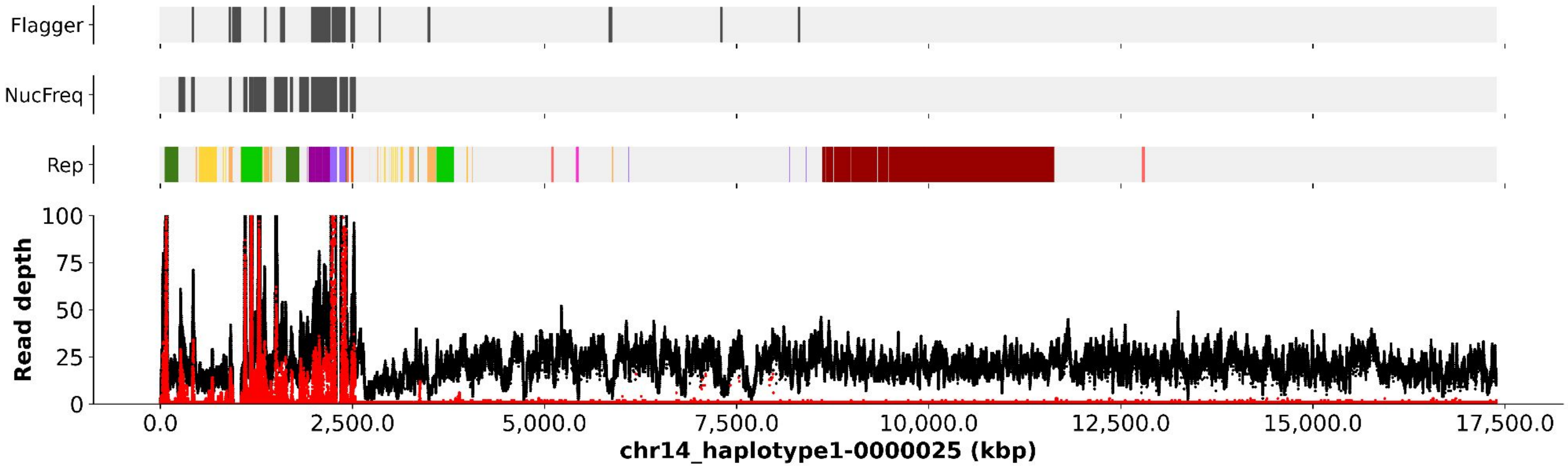

Flagger
NucFreq
Rep
Read depth
100
75
50
25
0
0.0
2,500.0
5,000.0
7,500.0
10,000.0
12,500.0
15,000.0
17,500.0
chr14_haplotype1-0000025 (kbp)

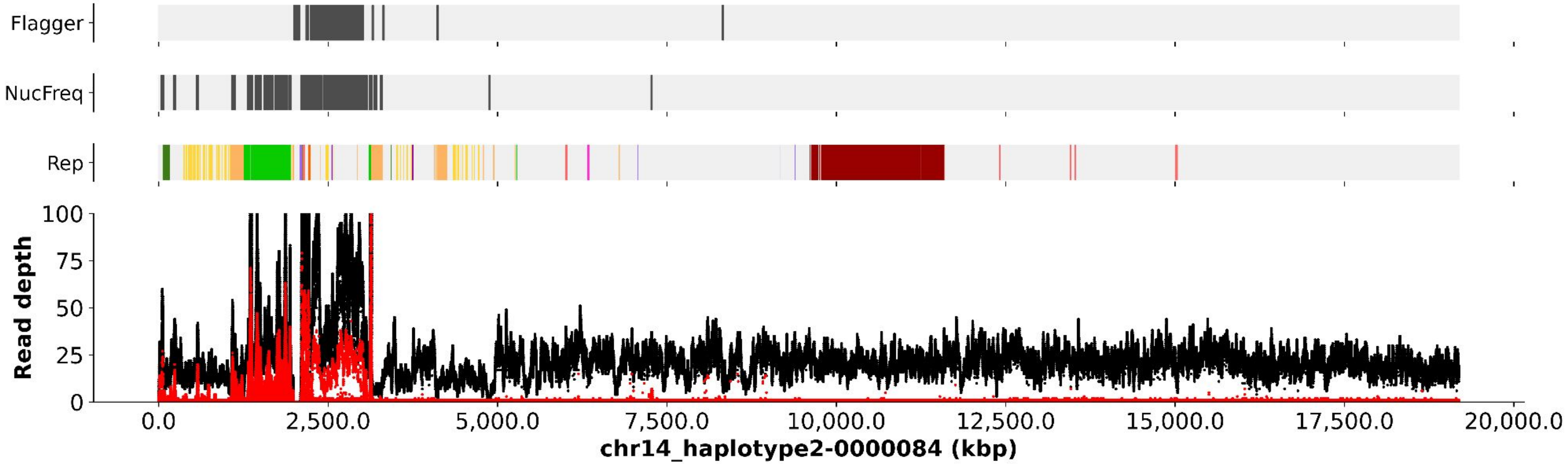

Flagger
NucFreq
Rep
Read depth
100
75
50
25
0
0.0
2,500.0
5,000.0
7,500.0
10,000.0
12,500.0
15,000.0
17,500.0
20,000.0
chr14_haplotype2-0000084 (kbp)

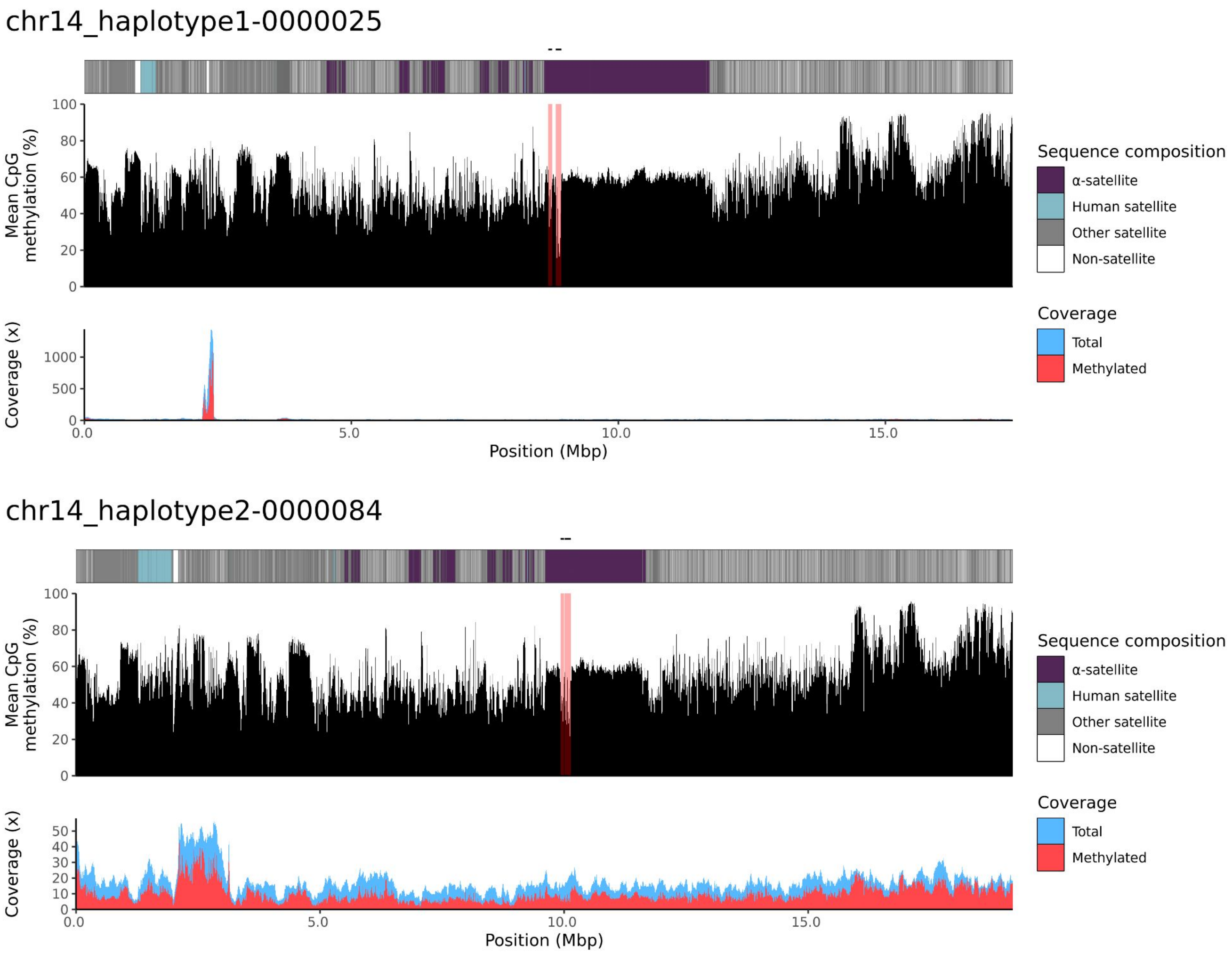
chr14_haplotype1-0000025
Mean CpG methylation (%)
100
80
60
40
20
0
Coverage (x)
1000
500
0
0.0
5.0
10.0
15.0
Position (Mbp)
Sequence composition
α-satellite
Human satellite
Other satellite
Non-satellite
Coverage
Total
Methylated
chr14_haplotype2-0000084
Mean CpG methylation (%)
100
80
60
40
20
0
Coverage (x)
50
40
30
20
10
0
0.0
5.0
10.0
15.0
Position (Mbp)
Sequence composition
α-satellite
Human satellite
Other satellite
Non-satellite
Coverage
Total
Methylated

chr15

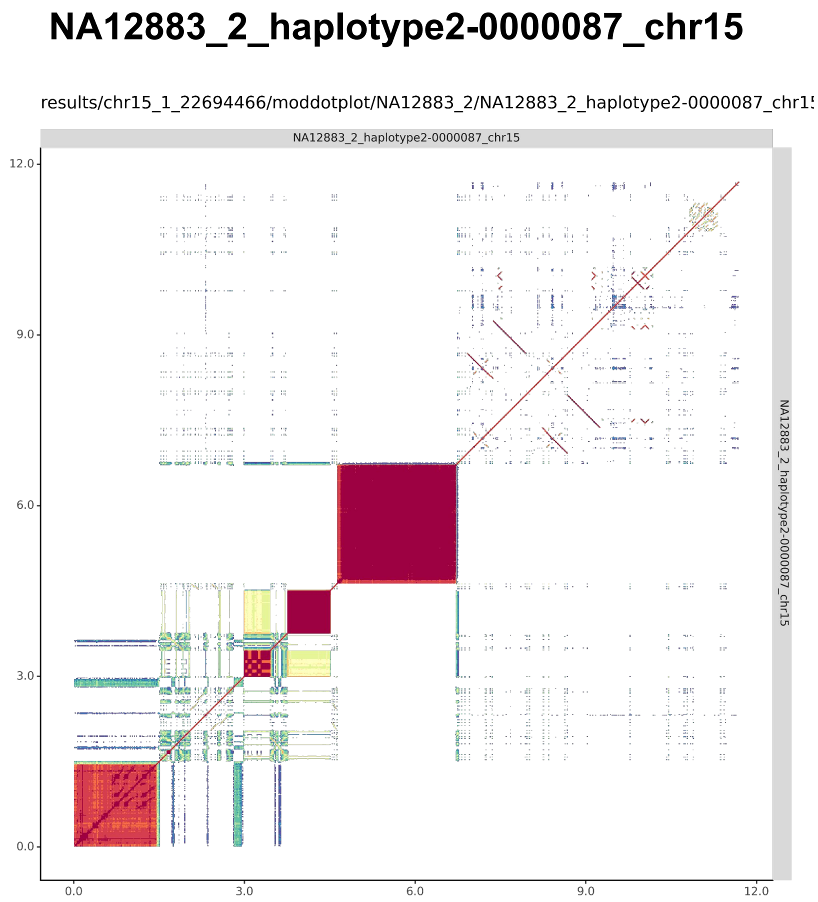

NA12883_2_haplotype2-0000087_chr15
results/chr15_1_22694466/moddotplot/NA12883_2/NA12883_2_haplotype2-0000087_chr15
NA12883_2_haplotype2-0000087_chr15
12.0
9.0
6.0
3.0
0.0
0.0
3.0
6.0
9.0
12.0
NA12883_2_haplotype2-0000087_chr15

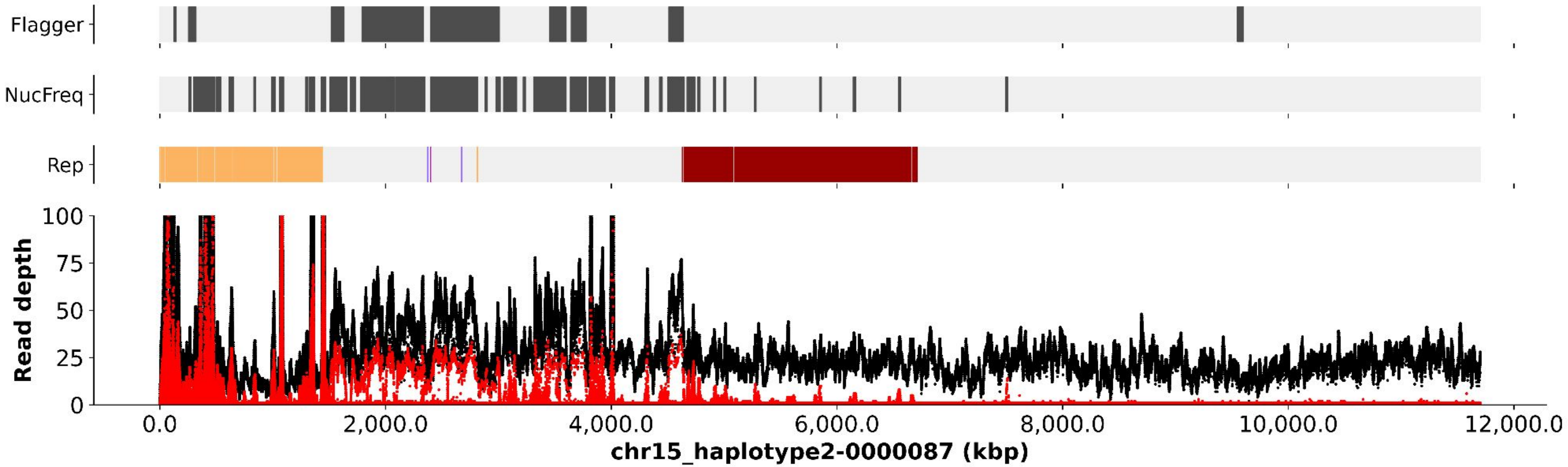

Flagger
NucFreq
Rep
Read depth
100
75
50
25
0
0.0
2,000.0
4,000.0
6,000.0
8,000.0
10,000.0
12,000.0
chr15_haplotype2-0000087 (kbp)

chr15_haplotype2-0000087

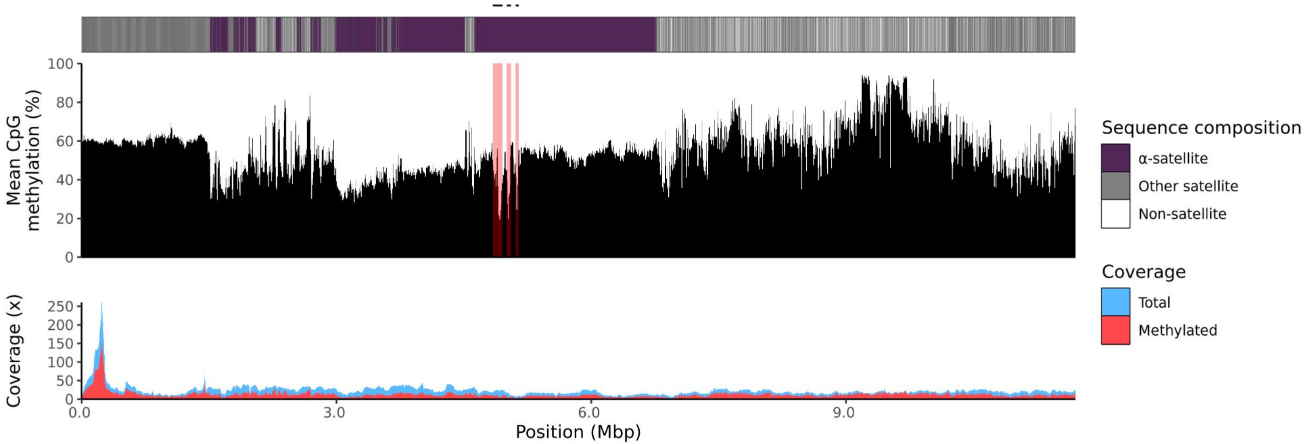

chr21

NA12883_1_haplotype1-0000019_chr21

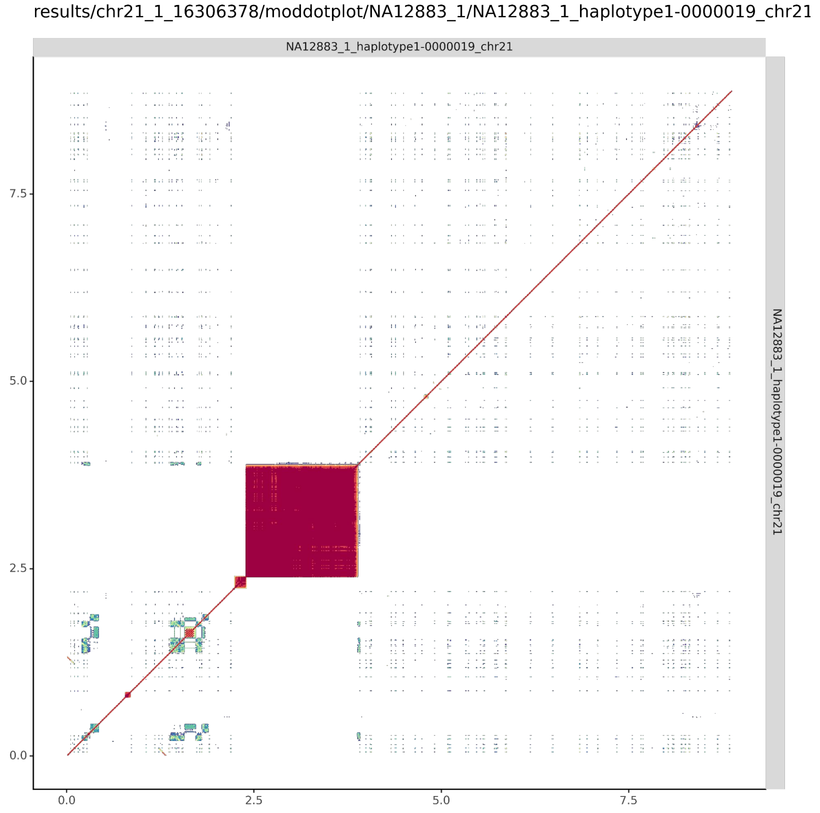

NA12883_2_haplotype2-0000080_chr21

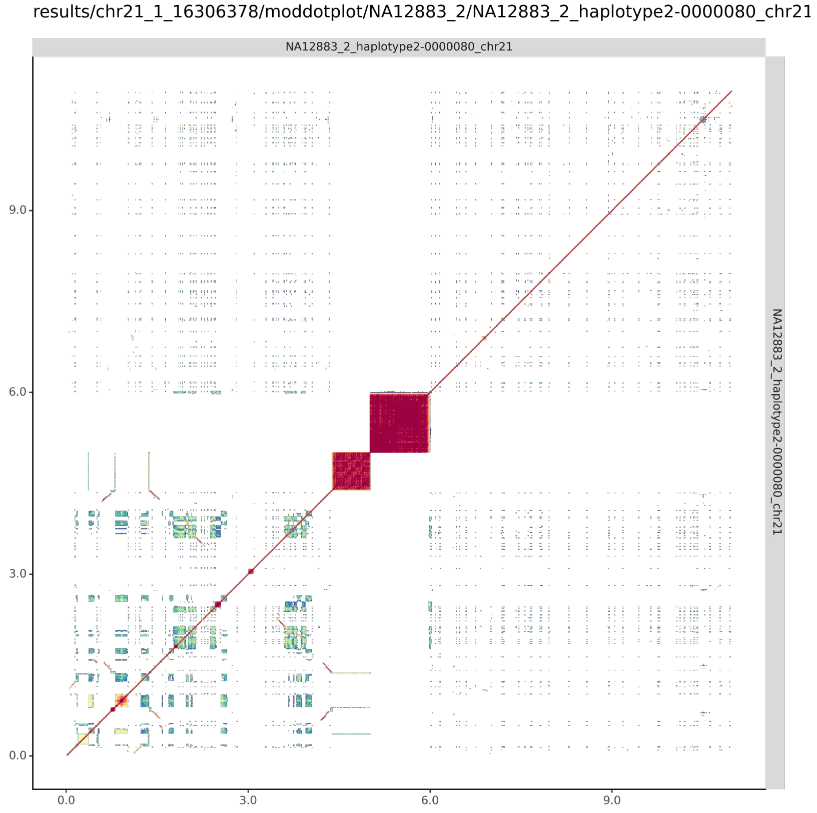

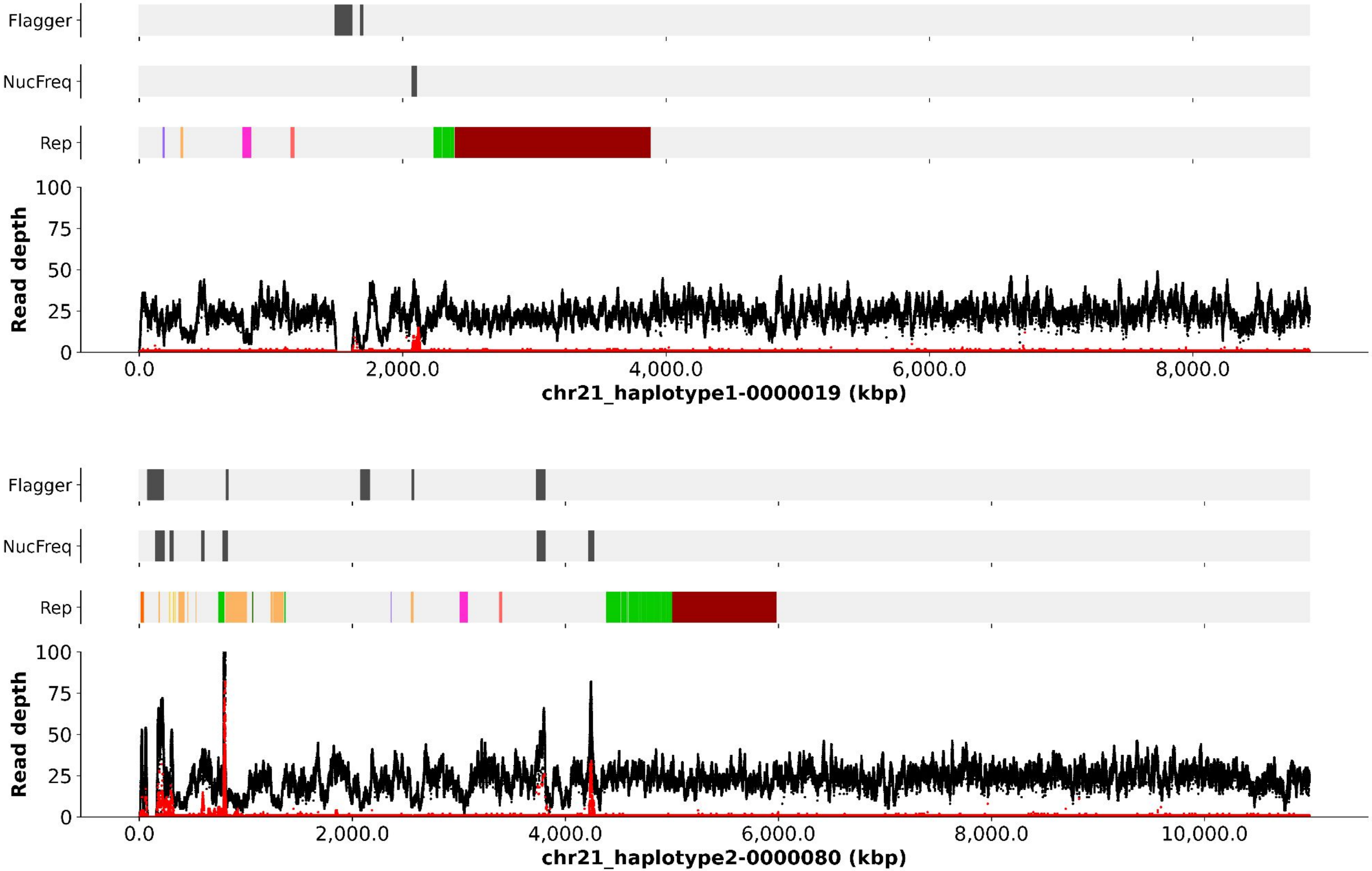

Flagger
NucFreq
Rep
Read depth
100
75
50
25
0
0.0
2,000.0
4,000.0
6,000.0
8,000.0
chr21_haplotype1-0000019 (kbp)
Flagger
NucFreq
Rep
Read depth
100
75
50
25
0
0.0
2,000.0
4,000.0
6,000.0
8,000.0
10,000.0
chr21_haplotype2-0000080 (kbp)

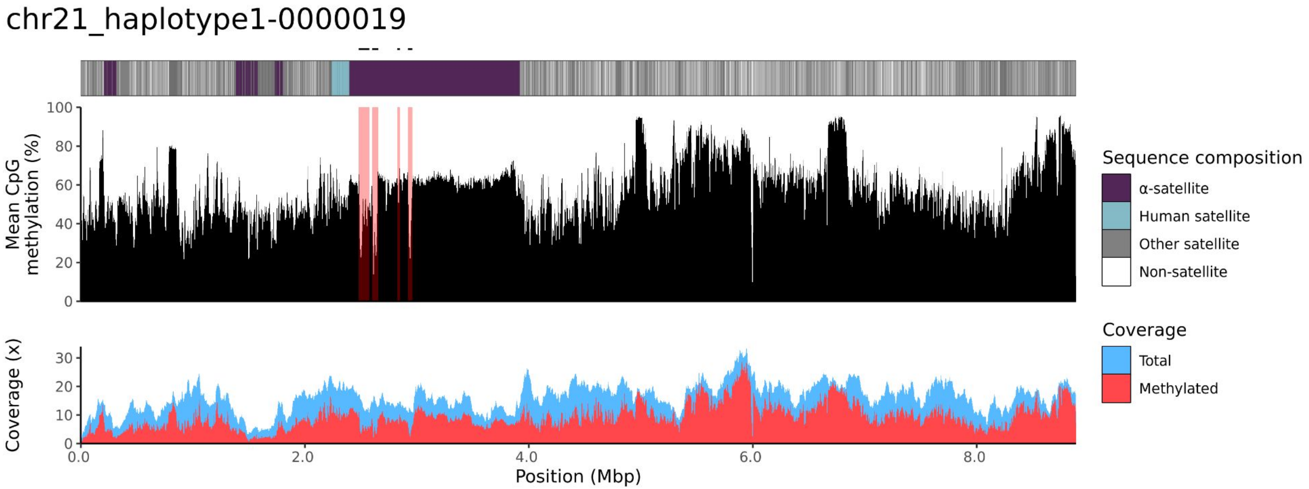

chr21_haplotype1-0000019
Mean CpG methylation (%)
Coverage (x)
Position (Mbp)
Sequence composition
α-satellite
Human satellite
Other satellite
Non-satellite
Coverage
Total
Methylated

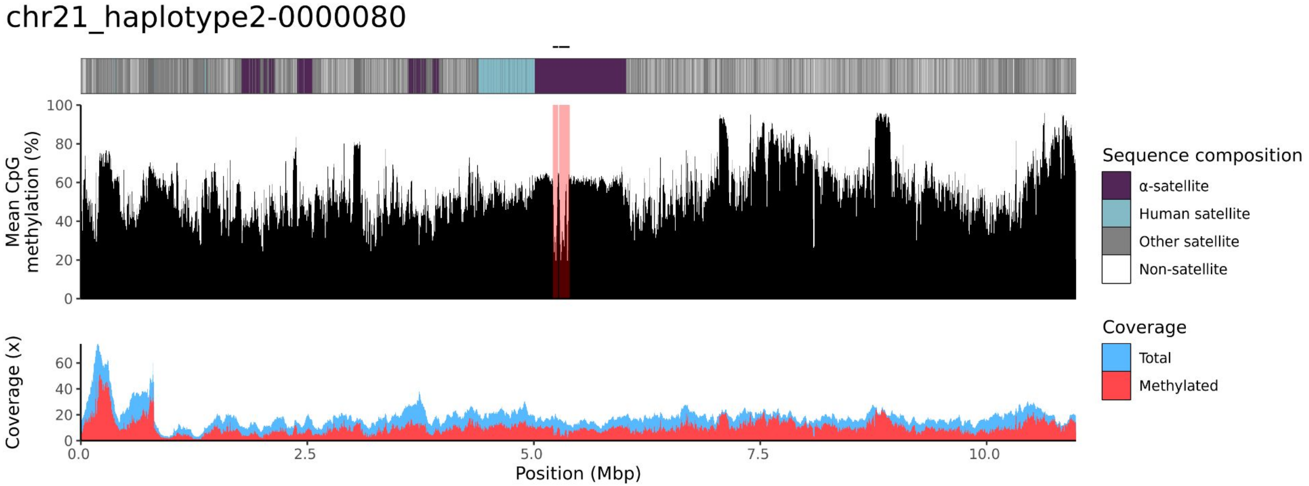

chr21_haplotype2-0000080
Mean CpG methylation (%)
Coverage (x)
Position (Mbp)
Sequence composition
α-satellite
Human satellite
Other satellite
Non-satellite
Coverage
Total
Methylated

chr22

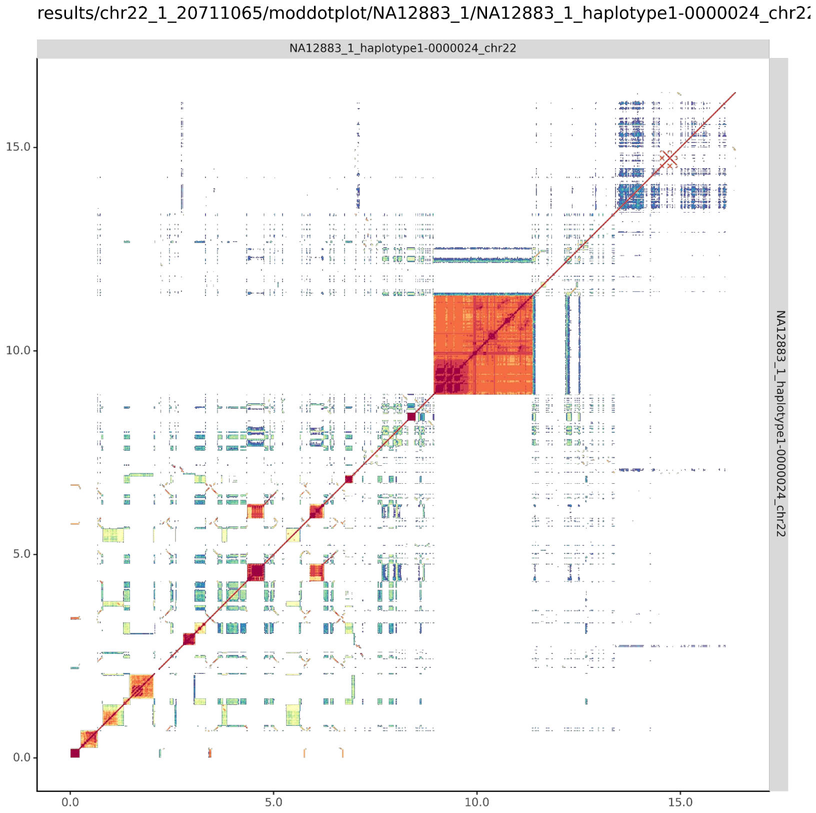

NA12883_1_haplotype1-0000024_chr22
results/chr22_1_20711065/moddotplot/NA12883_1/NA12883_1_haplotype1-0000024_chr22
NA12883_1_haplotype1-0000024_chr22
0.0
5.0
10.0
15.0
NA12883_1_haplotype1-0000024_chr22

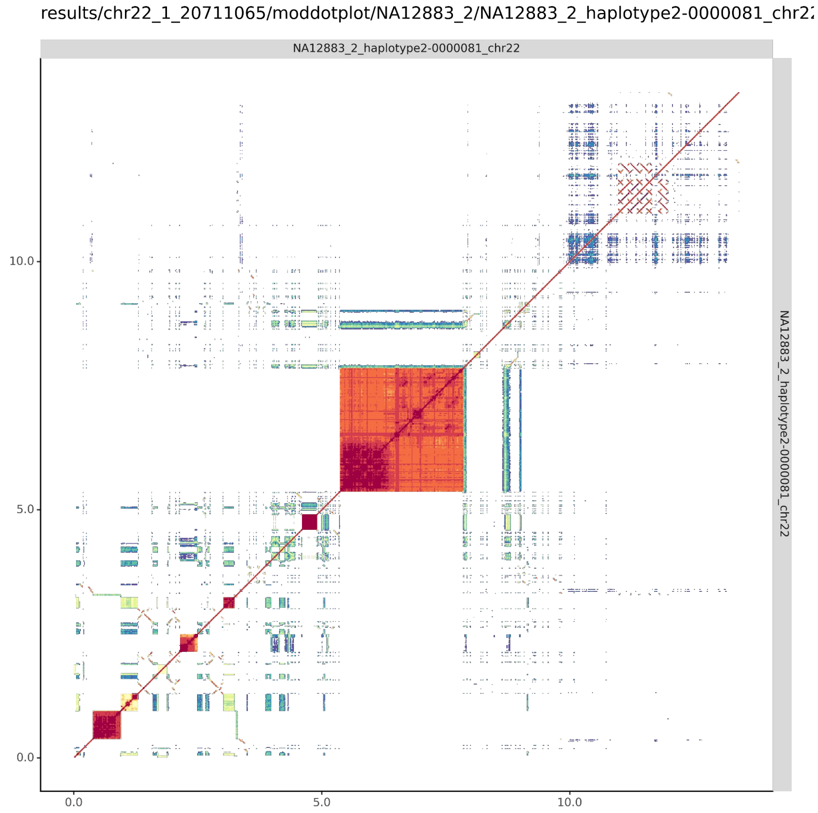

NA12883_2_haplotype2-0000081_chr22
results/chr22_1_20711065/moddotplot/NA12883_2/NA12883_2_haplotype2-0000081_chr22
NA12883_2_haplotype2-0000081_chr22
0.0
5.0
10.0
NA12883_2_haplotype2-0000081_chr22

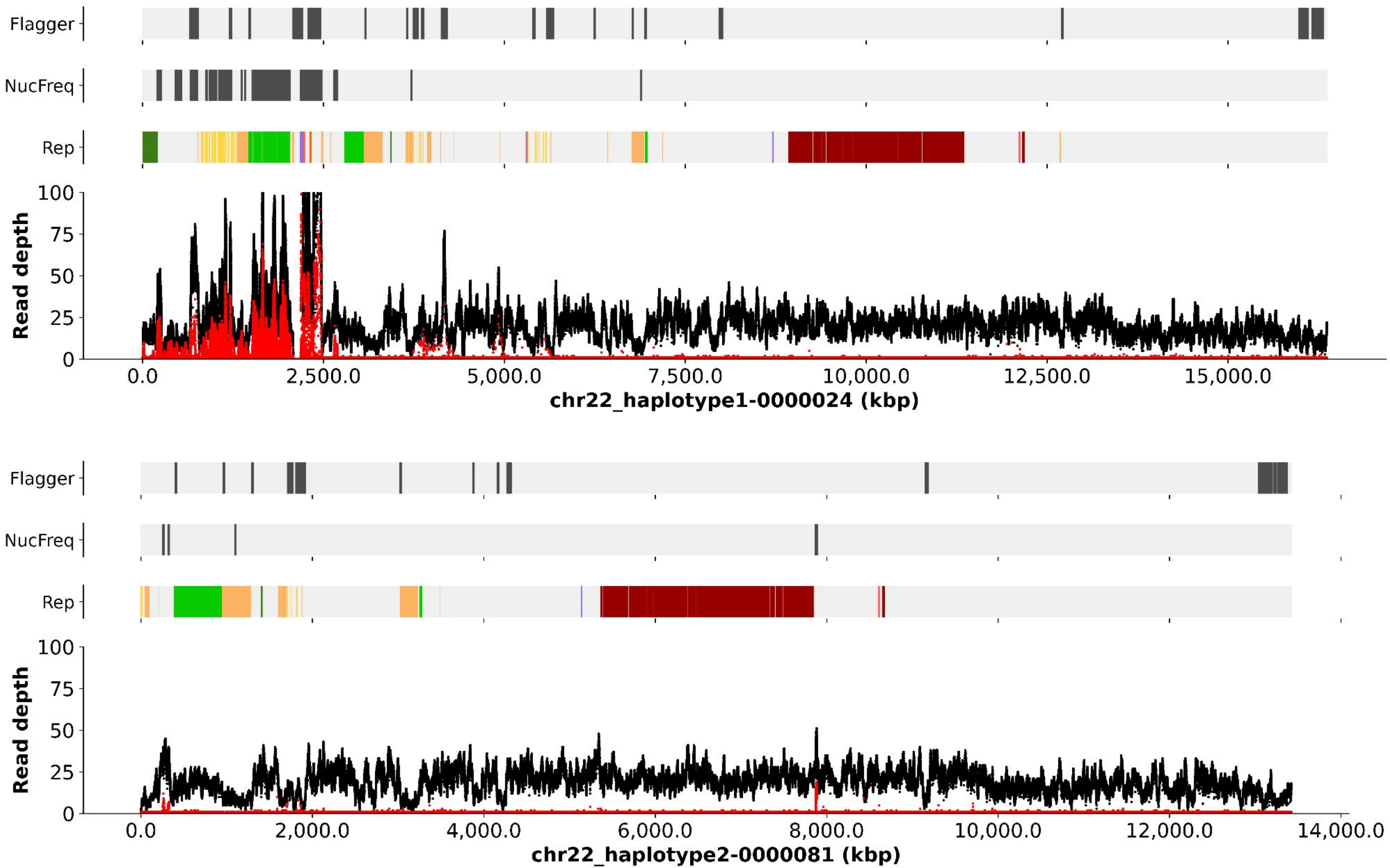
Flagger
NucFreq
Rep
Read depth
100
75
50
25
0
0.0
2,500.0
5,000.0
7,500.0
10,000.0
12,500.0
15,000.0
chr22_haplotype1-0000024 (kbp)
Flagger
NucFreq
Rep
Read depth
100
75
50
25
0
0.0
2,000.0
4,000.0
6,000.0
8,000.0
10,000.0
12,000.0
14,000.0
chr22_haplotype2-0000081 (kbp)

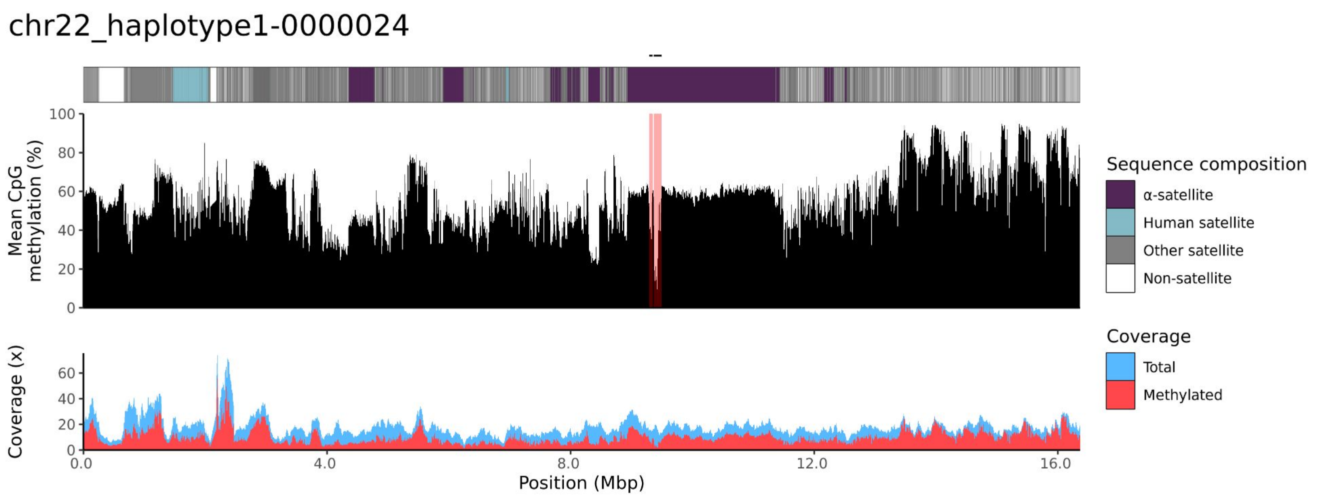
chr22_haplotype1-0000024
Mean CpG methylation (%)
Coverage (x)
Position (Mbp)
Sequence composition
α-satellite
Human satellite
Other satellite
Non-satellite
Coverage
Total
Methylated

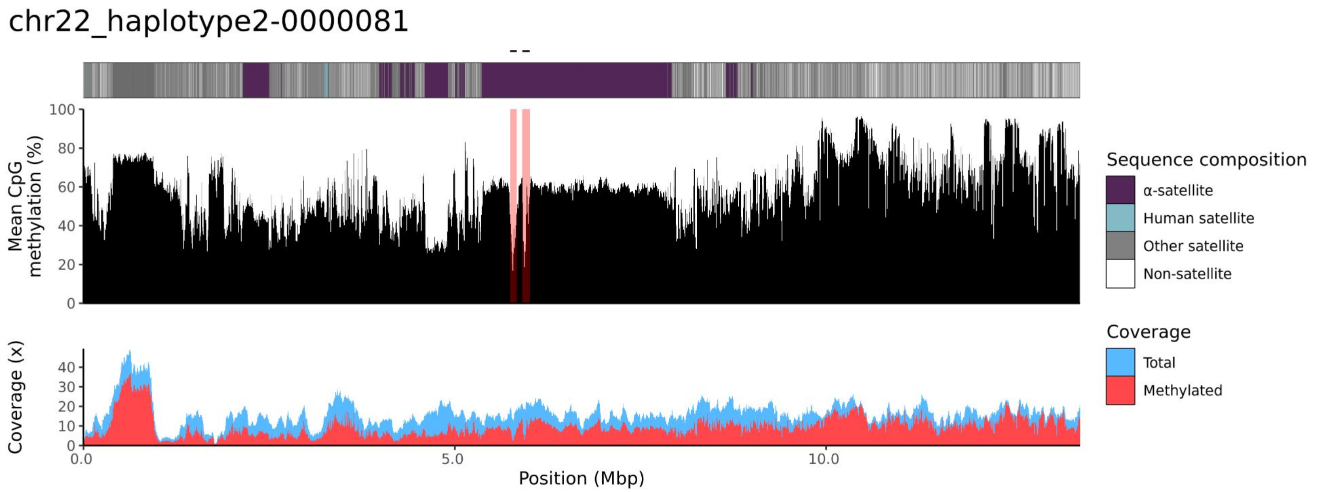
chr22_haplotype2-0000081
Mean CpG methylation (%)
Coverage (x)
Position (Mbp)
Sequence composition
α-satellite
Human satellite
Other satellite
Non-satellite
Coverage
Total
Methylated

NA12884

chr13

NA12884_1_haplotype1-0000016_chr13

results/chr13_1_22508596/moddotplot/NA12884_1/NA12884_1_haplotype1-0000016_chr1:

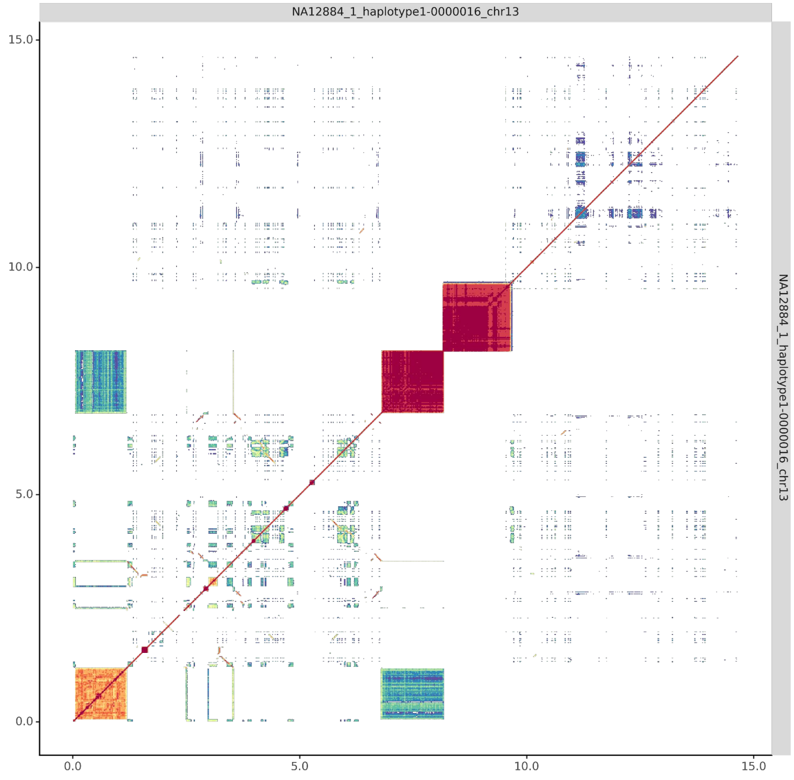

NA12884_2_haplotype2-0000068_chr13

results/chr13_1_22508596/moddotplot/NA12884_2/NA12884_2_haplotype2-0000068_chr1:

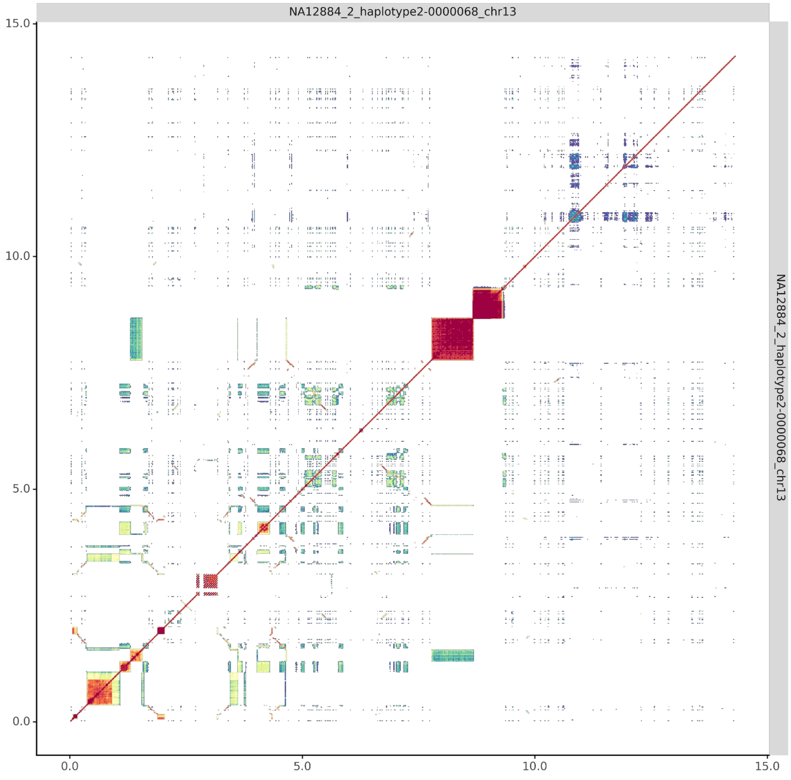

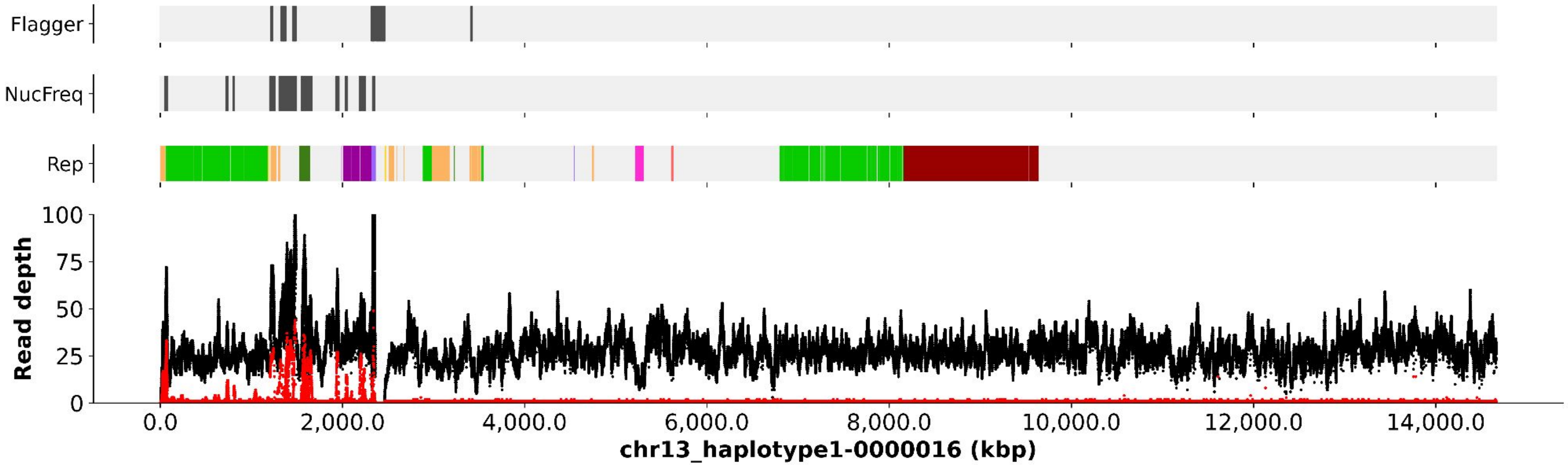
Flagger
NucFreq
Rep
Read depth
100
75
50
25
0
0.0
2,000.0
4,000.0
6,000.0
8,000.0
10,000.0
12,000.0
14,000.0
chr13_haplotype1-0000016 (kbp)

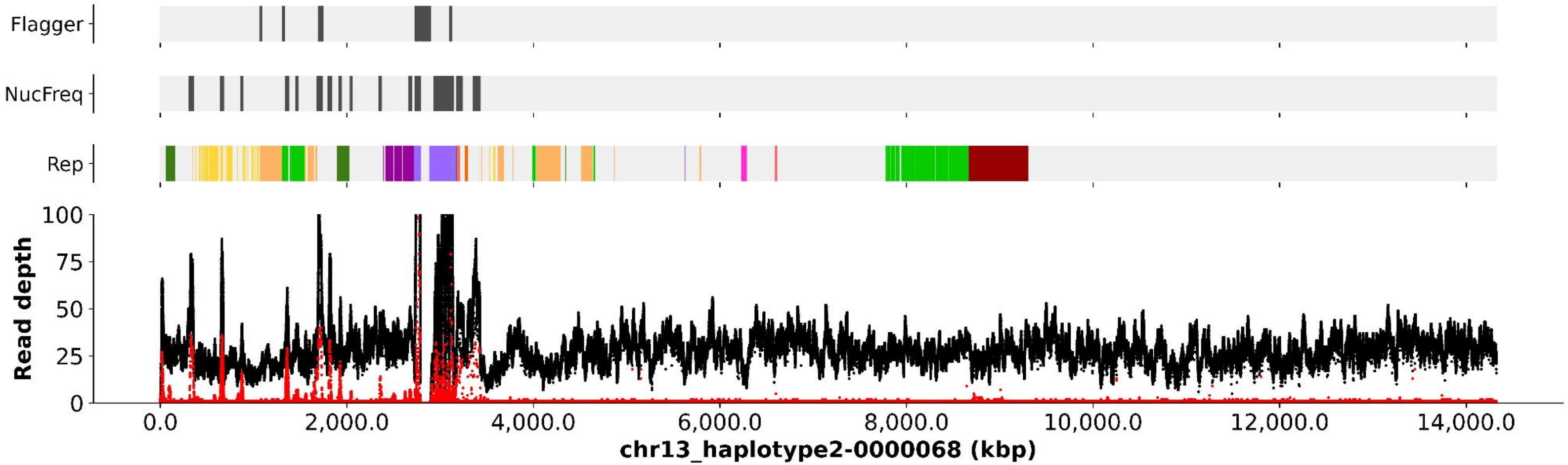
Flagger
NucFreq
Rep
Read depth
100
75
50
25
0
0.0
2,000.0
4,000.0
6,000.0
8,000.0
10,000.0
12,000.0
14,000.0
chr13_haplotype2-0000068 (kbp)

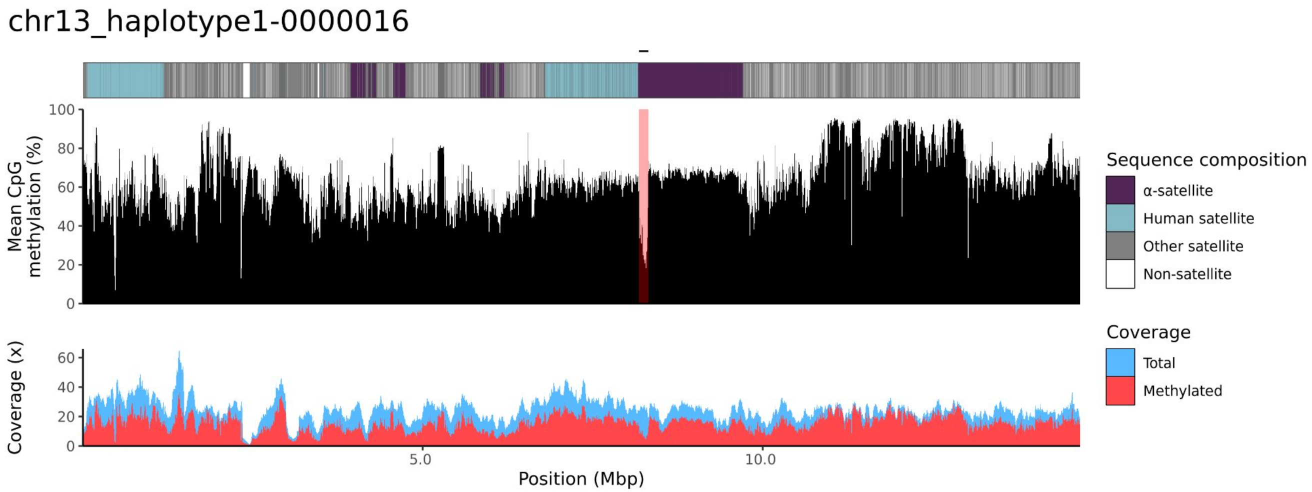
chr13_haplotype1-0000016
Mean CpG methylation (%)
100
80
60
40
20
0
Coverage (x)
60
40
20
0
5.0
10.0
Position (Mbp)
Sequence composition
α-satellite
Human satellite
Other satellite
Non-satellite
Coverage
Total
Methylated

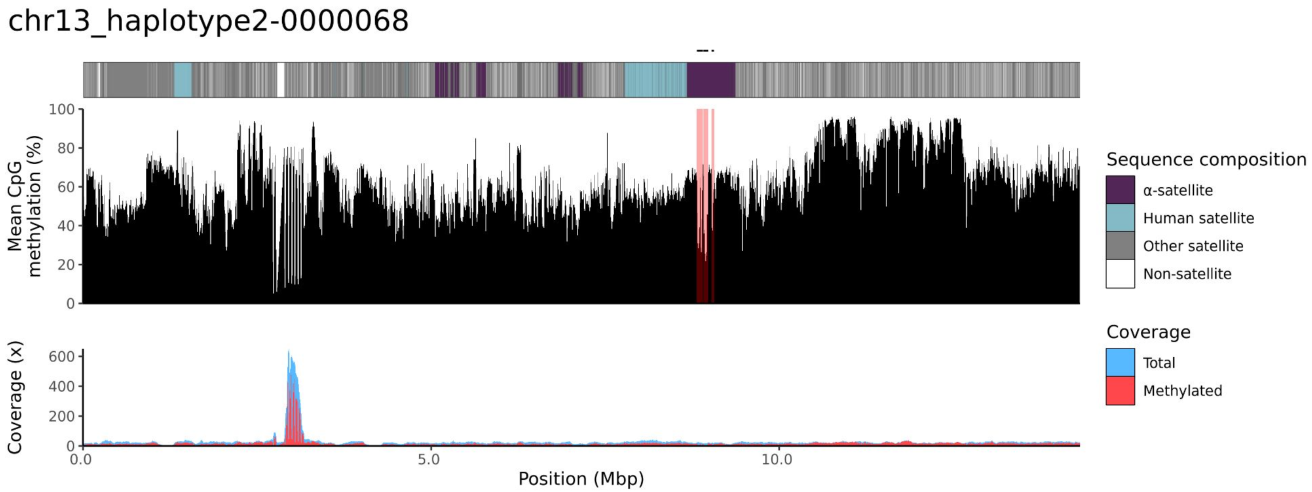
chr13_haplotype2-0000068
Mean CpG methylation (%)
100
80
60
40
20
0
Coverage (x)
600
400
200
0
0.0
5.0
10.0
Position (Mbp)
Sequence composition
α-satellite
Human satellite
Other satellite
Non-satellite
Coverage
Total
Methylated

chr14

NA12884_1_haplotype1-0000003_chr14

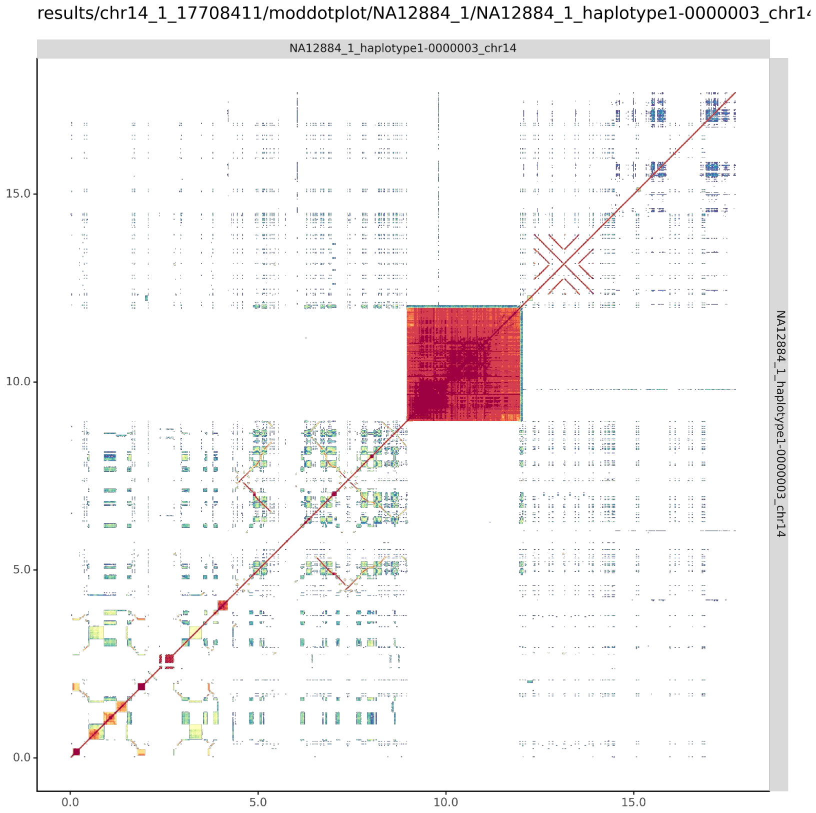

NA12884_2_haplotype2-0000058_chr14

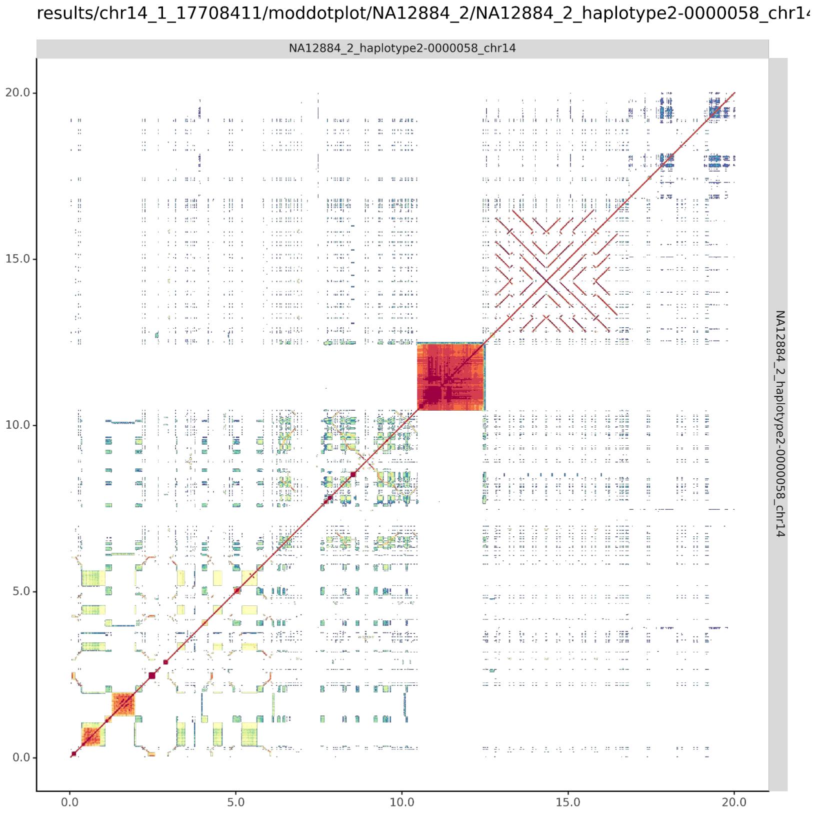

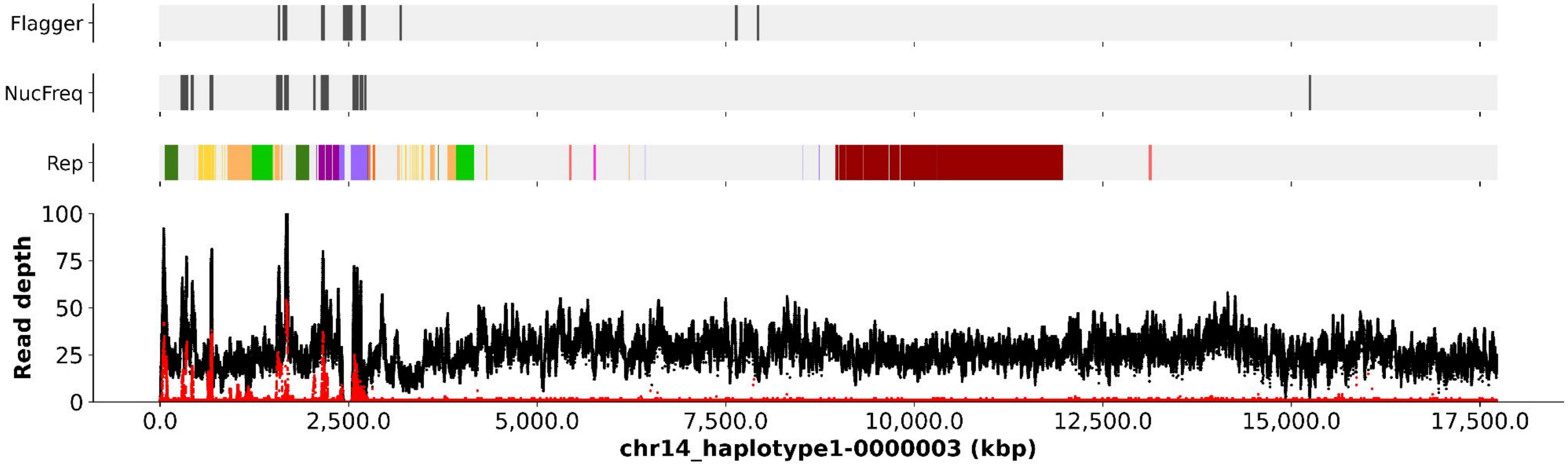
Flagger
NucFreq
Rep
Read depth
100
75
50
25
0
0.0
2,500.0
5,000.0
7,500.0
10,000.0
12,500.0
15,000.0
17,500.0
chr14_haplotype1-0000003 (kbp)

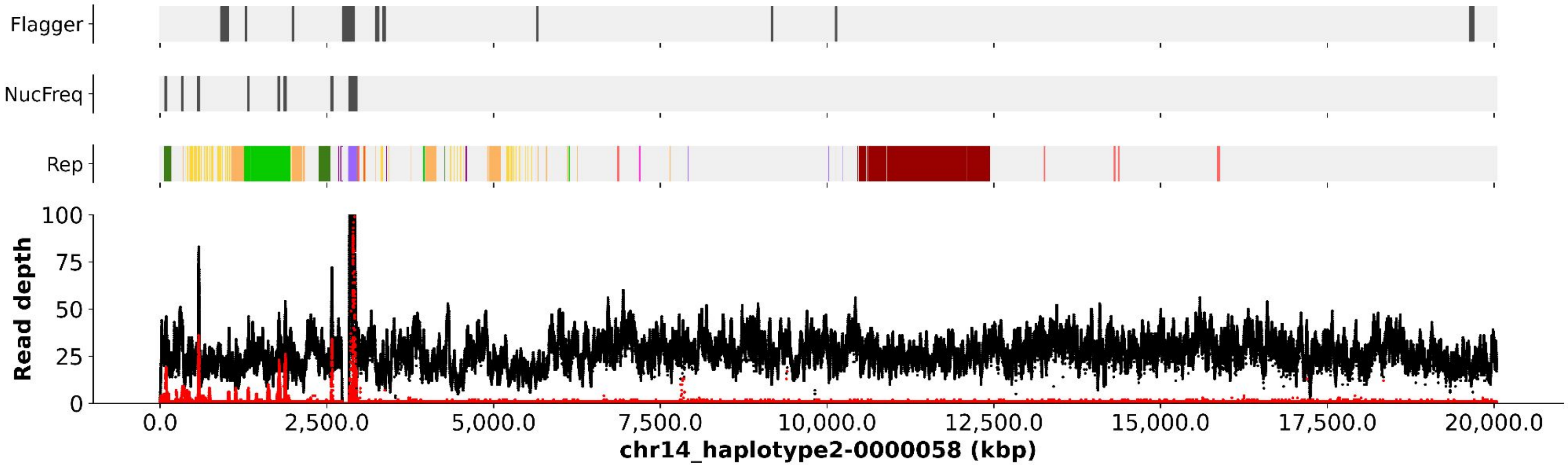
Flagger
NucFreq
Rep
Read depth
100
75
50
25
0
0.0
2,500.0
5,000.0
7,500.0
10,000.0
12,500.0
15,000.0
17,500.0
20,000.0
chr14_haplotype2-0000058 (kbp)

chr14_haplotype1-0000003

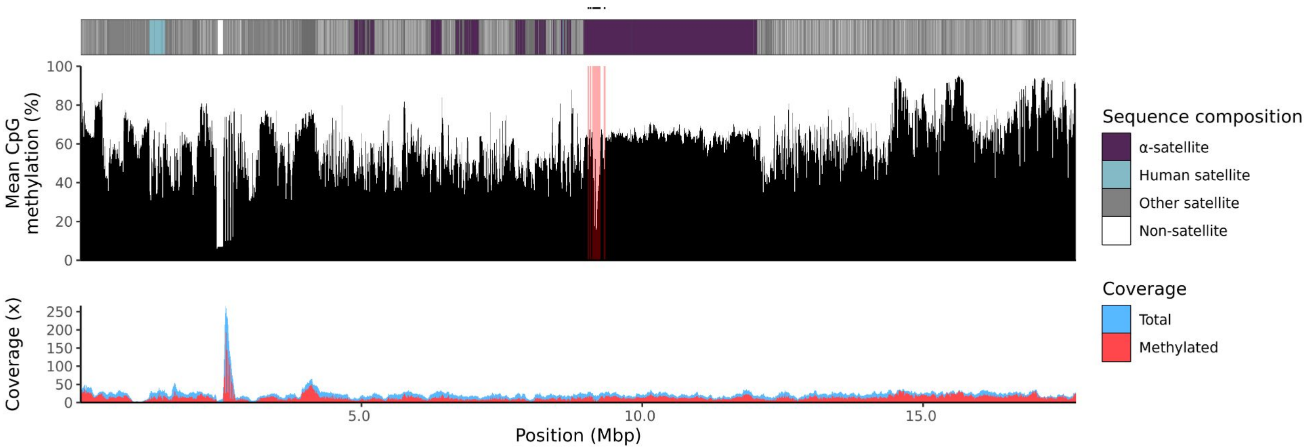

chr14_haplotype2-0000058

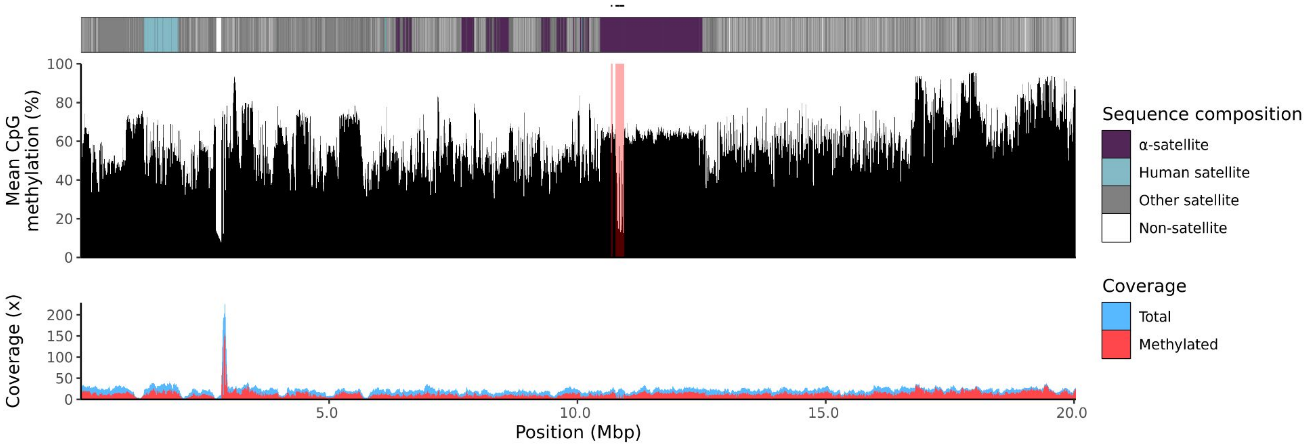

chr15

NA12884_1_haplotype1-0000015_chr15

results/chr15_1_22694466/moddotplot/NA12884_1/NA12884_1_haplotype1-0000015_chr1!

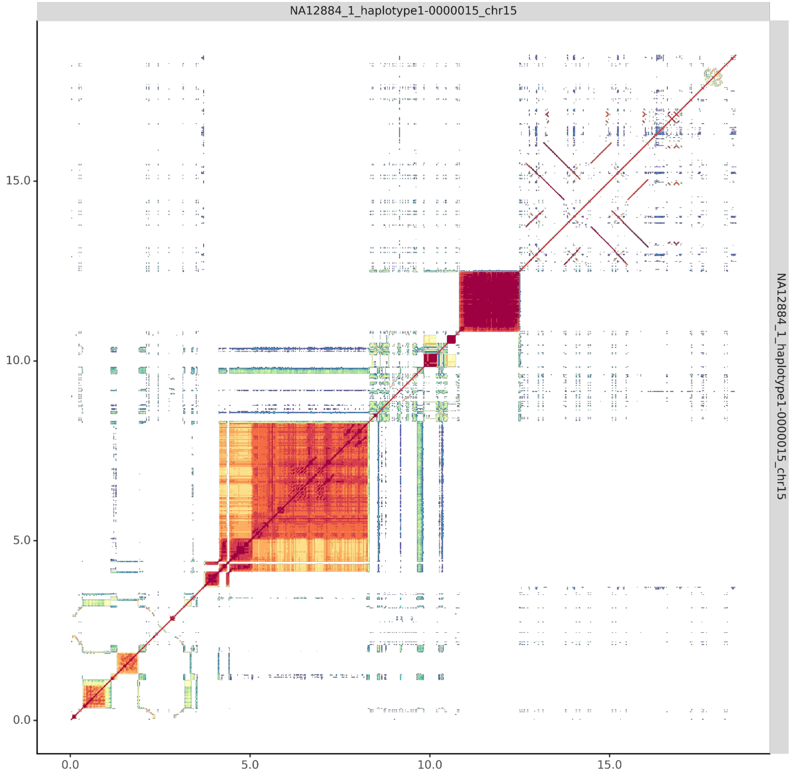

NA12884_2_haplotype2-0000067_chr15

results/chr15_1_22694466/moddotplot/NA12884_2/NA12884_2_haplotype2-0000067_chr1!

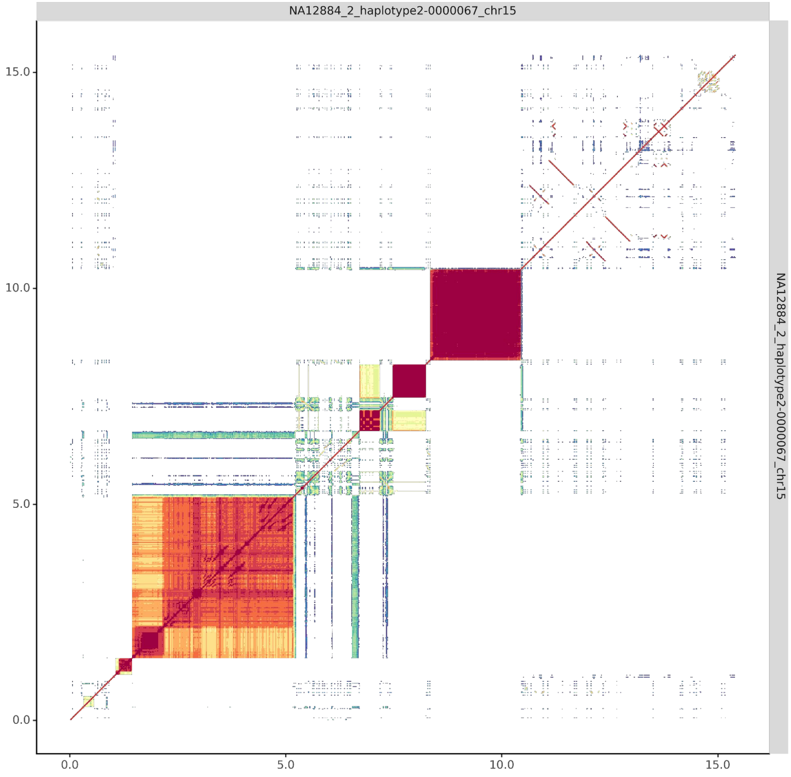

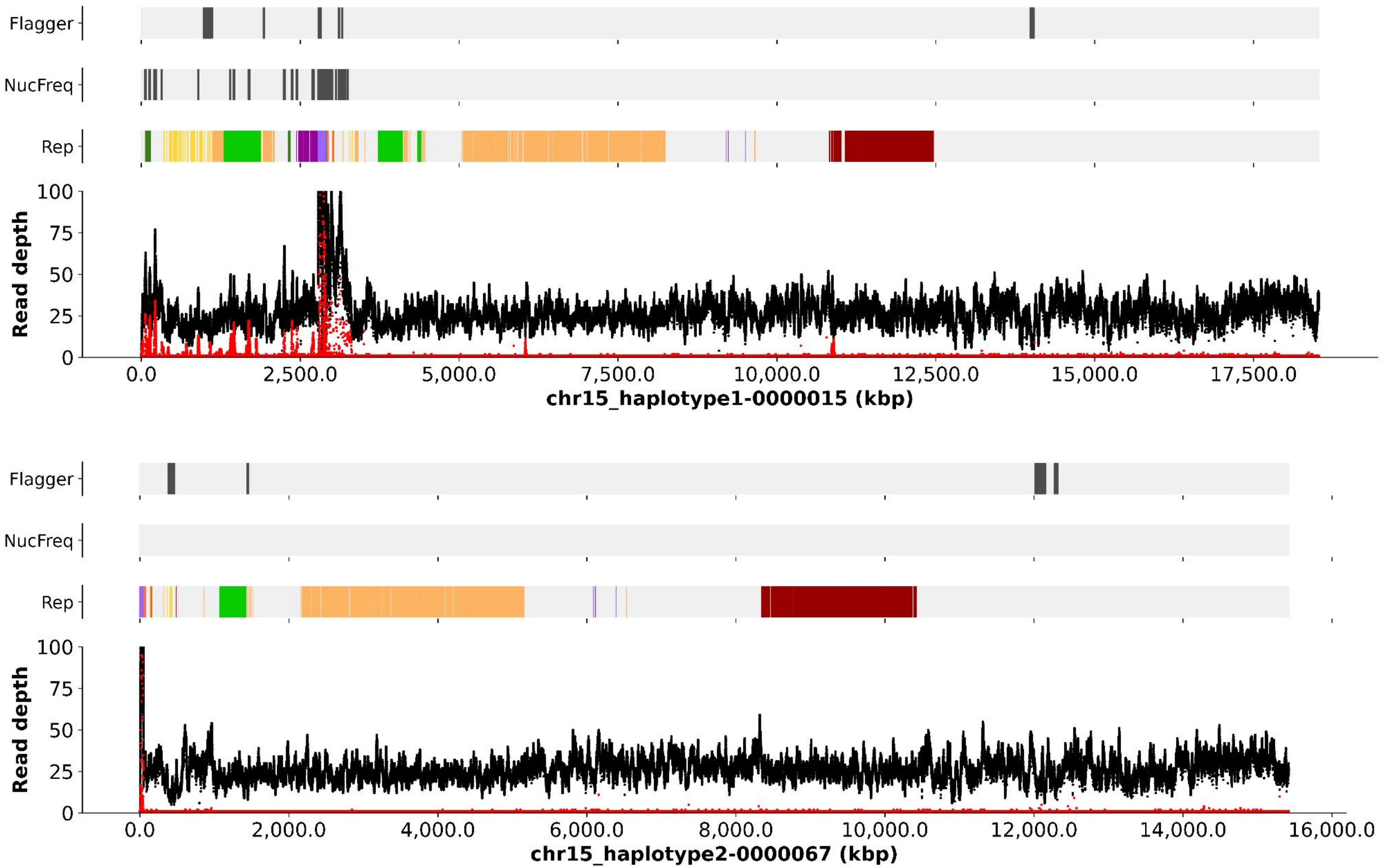

Flagger
NucFreq
Rep
Read depth
100
75
50
25
0
0.0
2,500.0
5,000.0
7,500.0
10,000.0
12,500.0
15,000.0
17,500.0
chr15_haplotype1-0000015 (kbp)
Flagger
NucFreq
Rep
Read depth
100
75
50
25
0
0.0
2,000.0
4,000.0
6,000.0
8,000.0
10,000.0
12,000.0
14,000.0
16,000.0
chr15_haplotype2-0000067 (kbp)

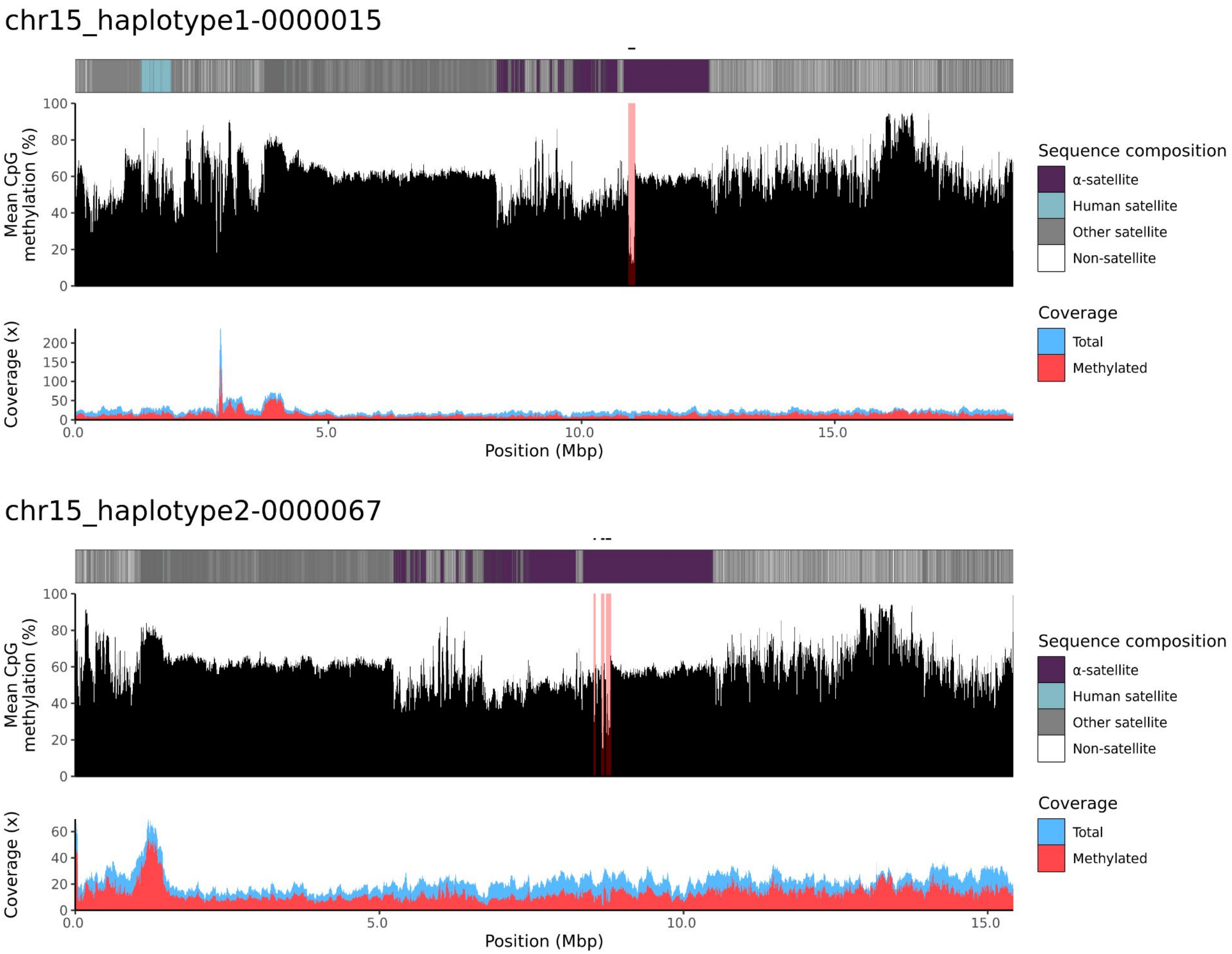
chr15_haplotype1-0000015
Mean CpG methylation (%)
Coverage (x)
Position (Mbp)
Sequence composition
α-satellite
Human satellite
Other satellite
Non-satellite
Coverage
Total
Methylated
chr15_haplotype2-0000067
Mean CpG methylation (%)
Coverage (x)
Position (Mbp)
Sequence composition
α-satellite
Human satellite
Other satellite
Non-satellite
Coverage
Total
Methylated

chr21

NA12884_1_haplotype1-0000010_chr21

results/chr21_1_16306378/moddotplot/NA12884_1/NA12884_1_haplotype1-0000010_chr2:

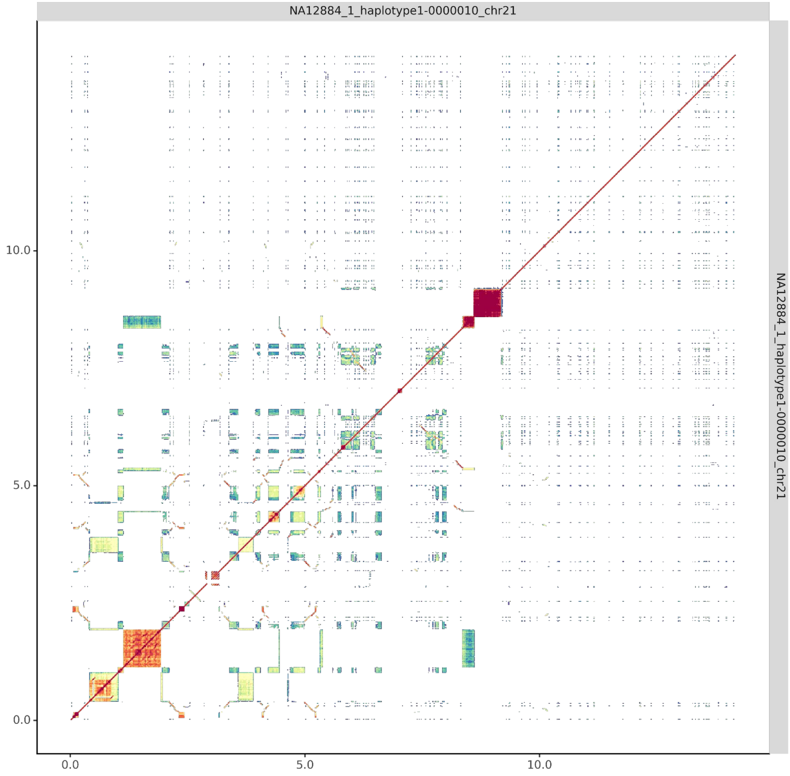

NA12884_2_haplotype2-0000075_chr21

results/chr21_1_16306378/moddotplot/NA12884_2/NA12884_2_haplotype2-0000075_chr2:

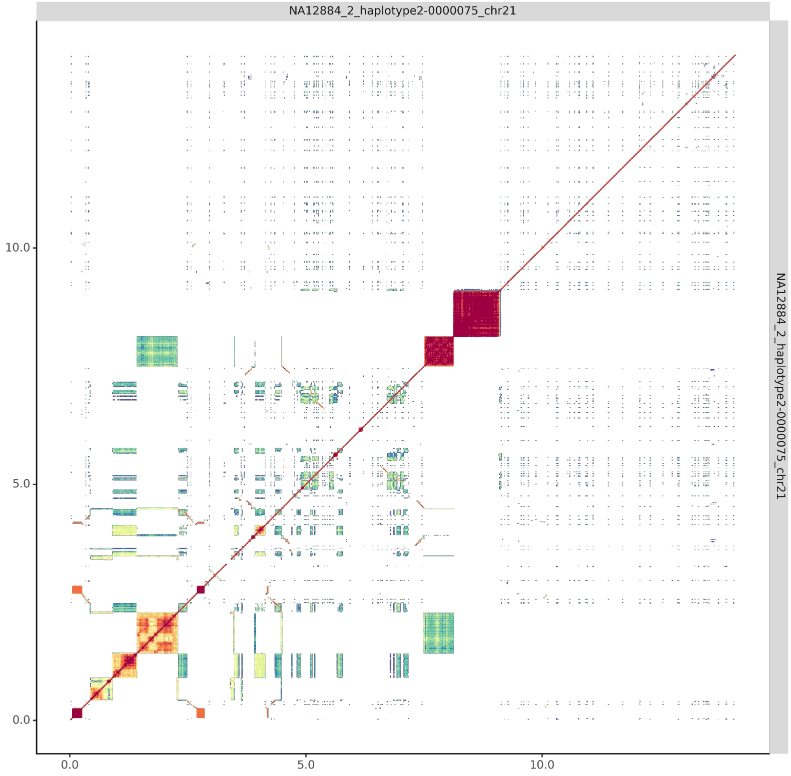

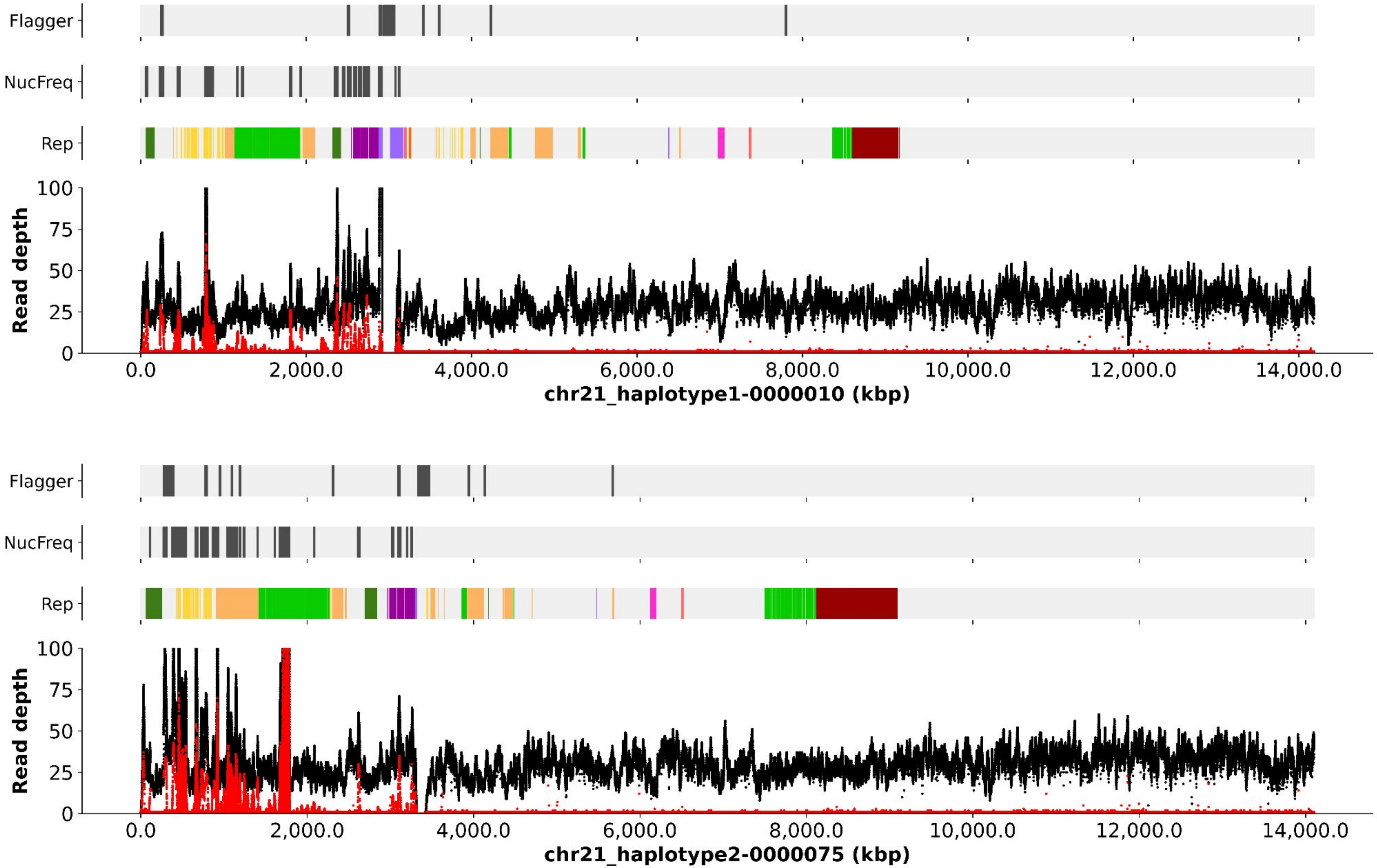
Flagger
NucFreq
Rep
Read depth
100
75
50
25
0
0.0
2,000.0
4,000.0
6,000.0
8,000.0
10,000.0
12,000.0
14,000.0
chr21_haplotype1-0000010 (kbp)
Flagger
NucFreq
Rep
Read depth
100
75
50
25
0
0.0
2,000.0
4,000.0
6,000.0
8,000.0
10,000.0
12,000.0
14,000.0
chr21_haplotype2-0000075 (kbp)

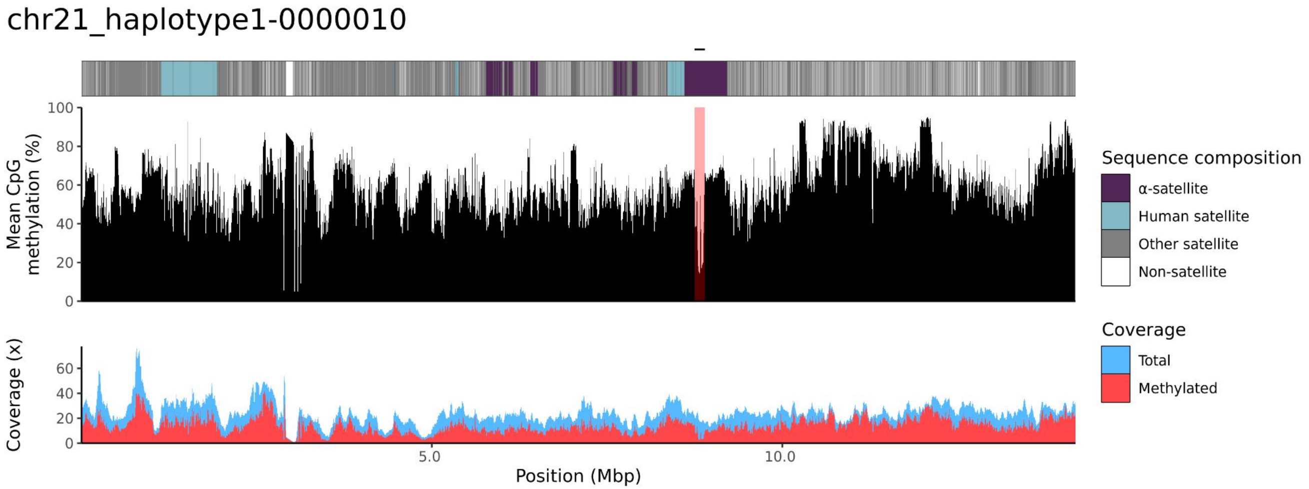
chr21_haplotype1-0000010
Mean CpG methylation (%)
Coverage (x)
Position (Mbp)
Sequence composition
α-satellite
Human satellite
Other satellite
Non-satellite
Coverage
Total
Methylated

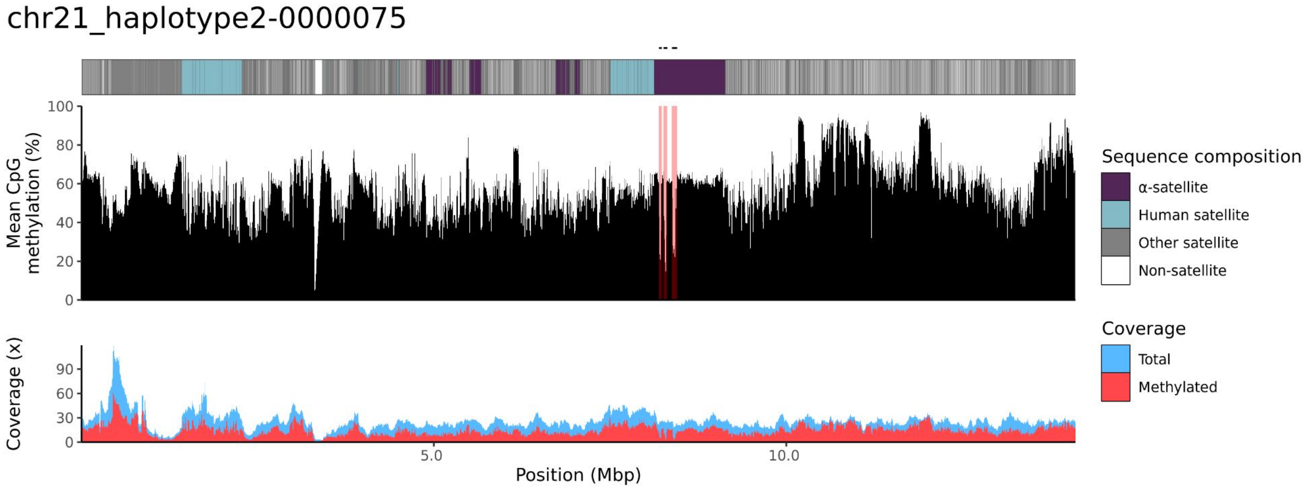
chr21_haplotype2-0000075
Mean CpG methylation (%)
Coverage (x)
Position (Mbp)
Sequence composition
α-satellite
Human satellite
Other satellite
Non-satellite
Coverage
Total
Methylated

chr22

NA12884_1_haplotype1-0000009_chr22

results/chr22_1_20711065/moddotplot/NA12884_1/NA12884_1_haplotype1-0000009_chr2

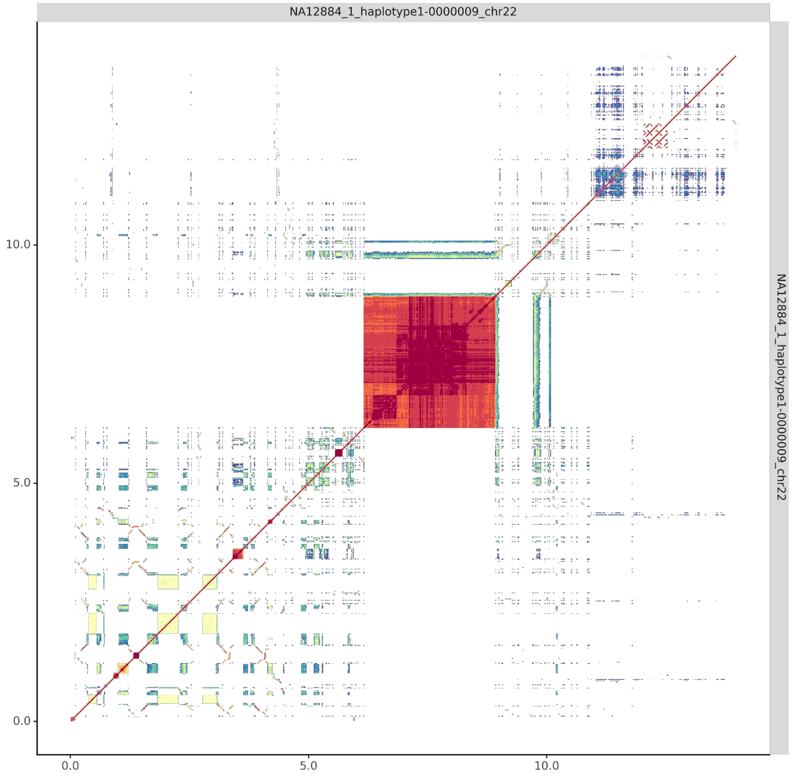

NA12884_2_haplotype2-0000071_chr22

results/chr22_1_20711065/moddotplot/NA12884_2/NA12884_2_haplotype2-0000071_chr2

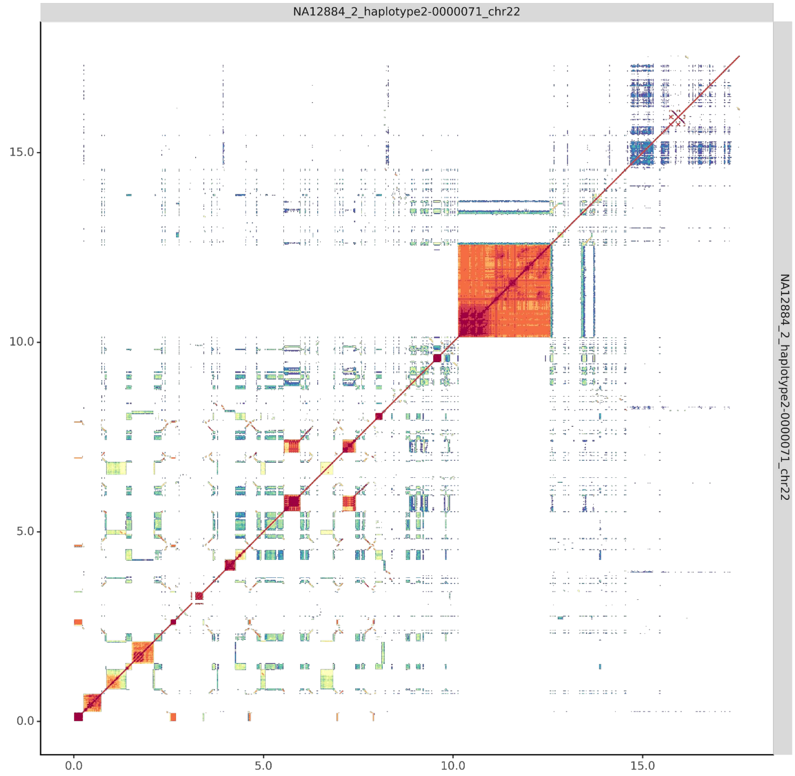

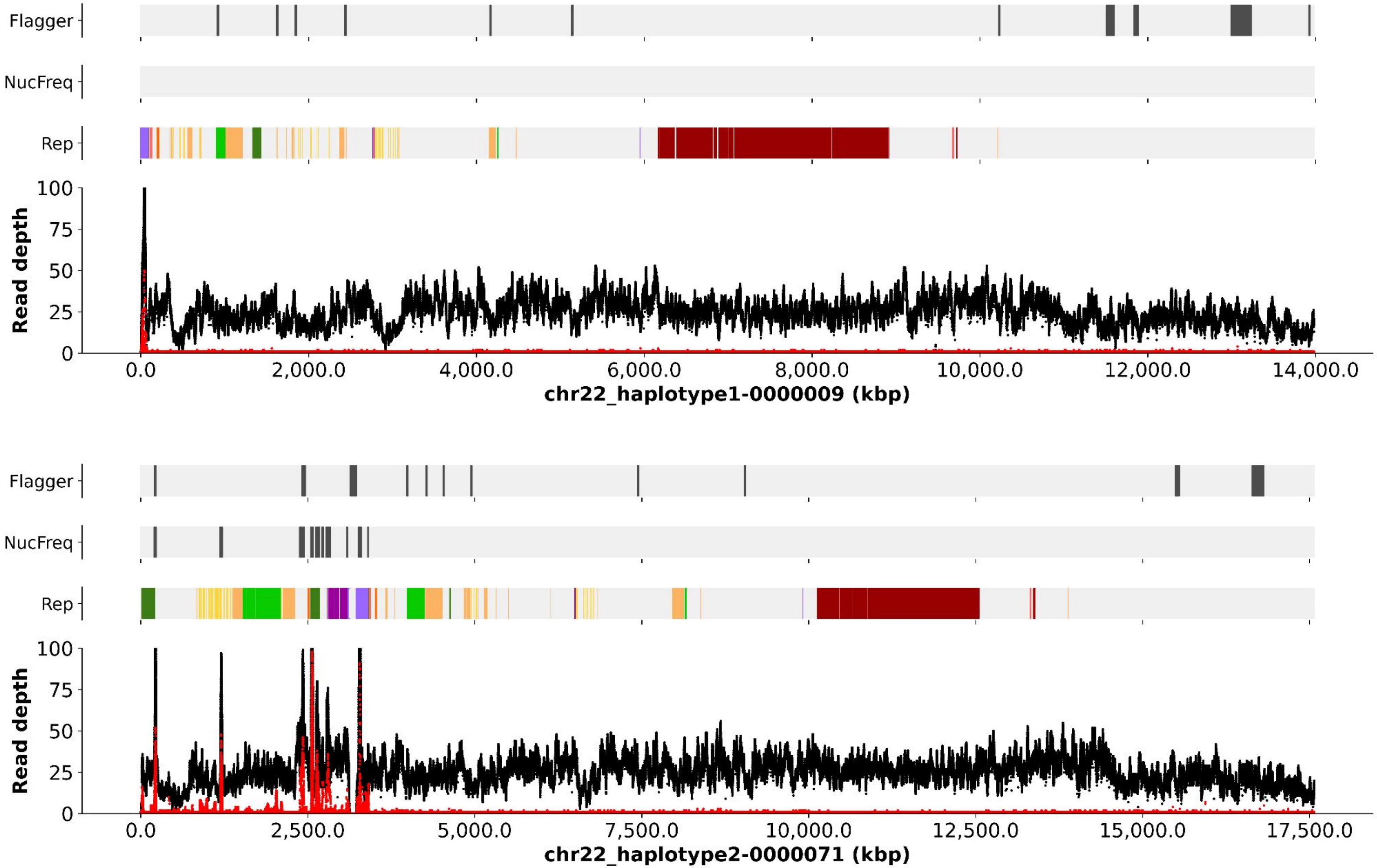

Flagger
NucFreq
Rep
Read depth
100
75
50
25
0
0.0
2,000.0
4,000.0
6,000.0
8,000.0
10,000.0
12,000.0
14,000.0
chr22_haplotype1-0000009 (kbp)
Flagger
NucFreq
Rep
Read depth
100
75
50
25
0
0.0
2,500.0
5,000.0
7,500.0
10,000.0
12,500.0
15,000.0
17,500.0
chr22_haplotype2-0000071 (kbp)

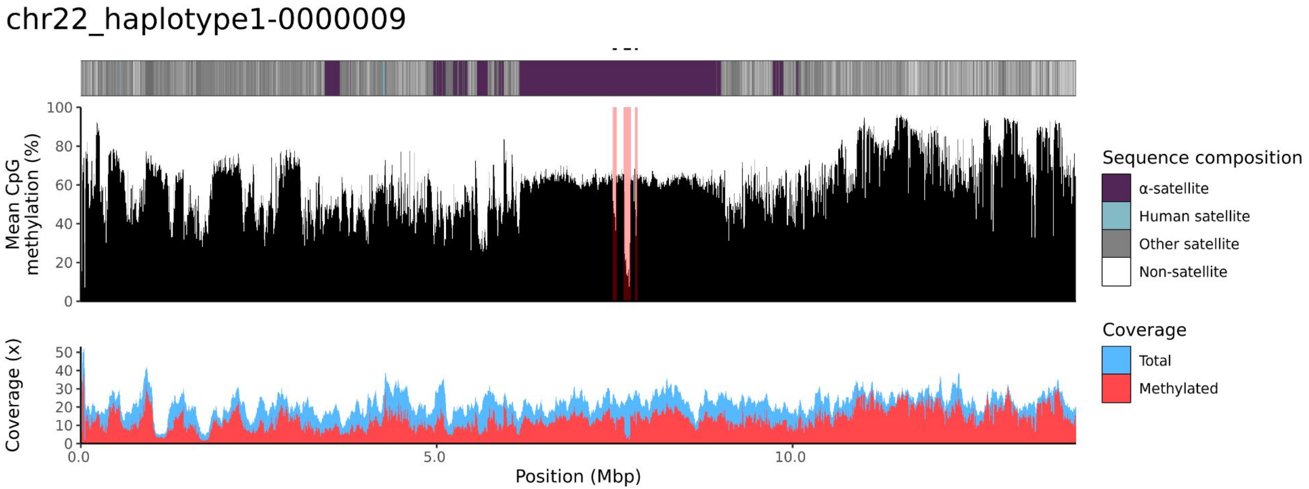
chr22_haplotype1-0000009
Mean CpG methylation (%)
Coverage (x)
Position (Mbp)
Sequence composition
α-satellite
Human satellite
Other satellite
Non-satellite
Coverage
Total
Methylated

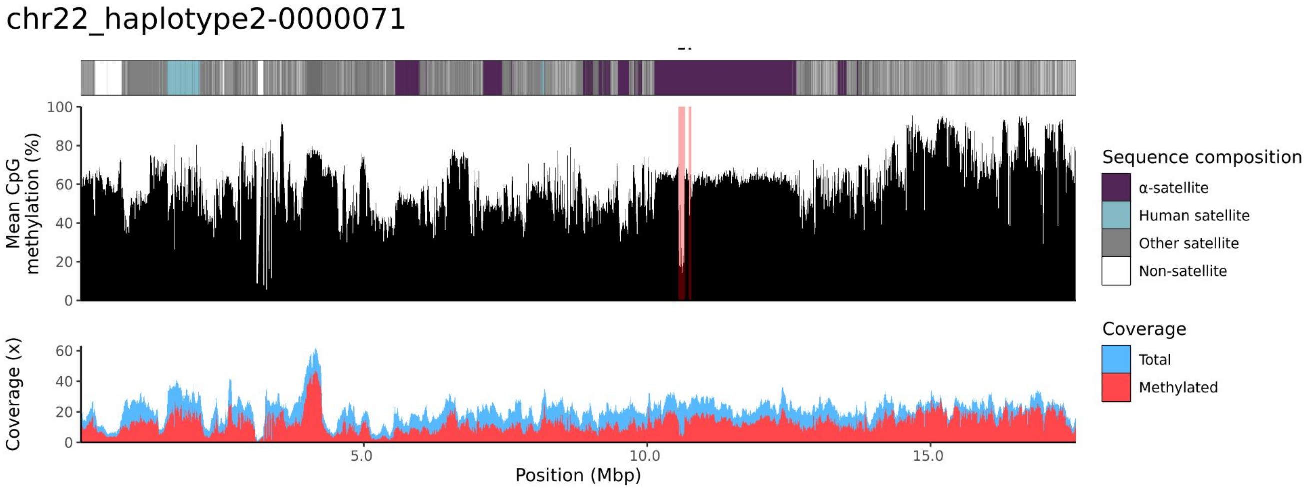
chr22_haplotype2-0000071
Mean CpG methylation (%)
Coverage (x)
Position (Mbp)
Sequence composition
α-satellite
Human satellite
Other satellite
Non-satellite
Coverage
Total
Methylated

NA12885

chr13

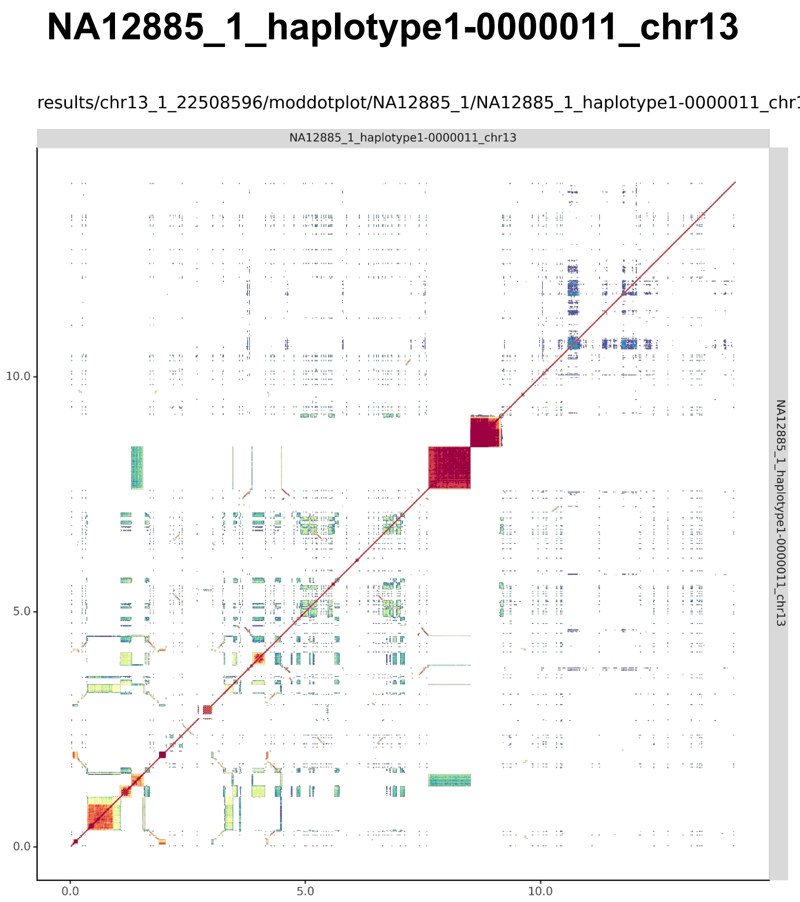

NA12885_1_haplotype1-0000011_chr13
results/chr13_1_22508596/moddotplot/NA12885_1/NA12885_1_haplotype1-0000011_chr13
NA12885_1_haplotype1-0000011_chr13
10.0
5.0
0.0
0.0
5.0
10.0
NA12885_1_haplotype1-0000011_chr13

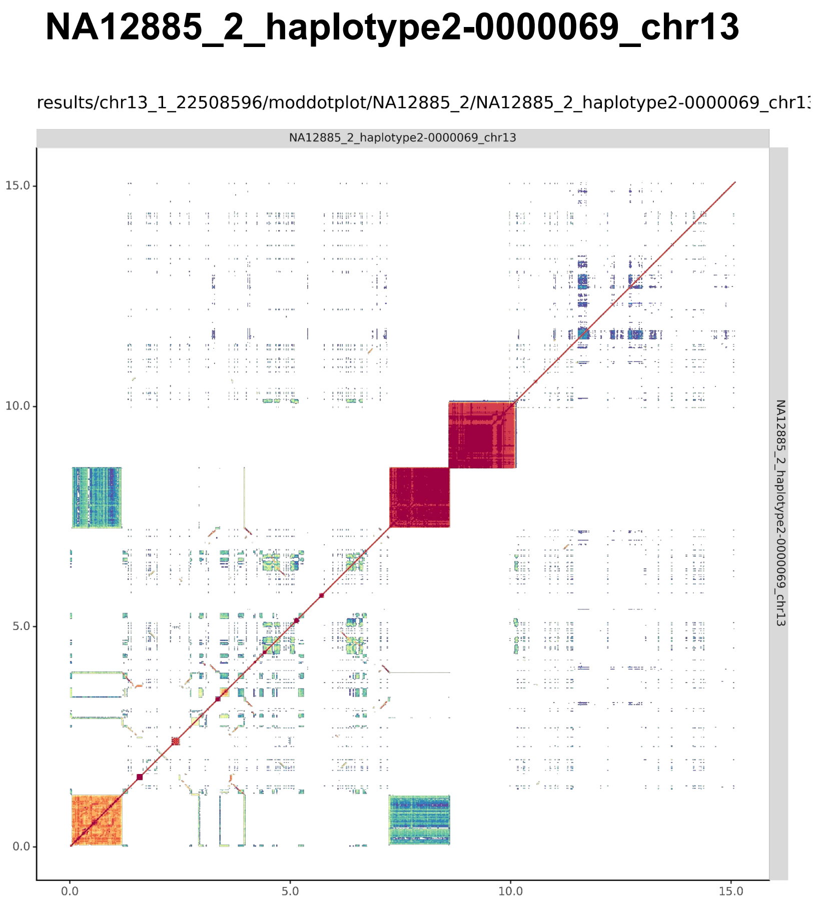

NA12885_2_haplotype2-0000069_chr13
results/chr13_1_22508596/moddotplot/NA12885_2/NA12885_2_haplotype2-0000069_chr13
NA12885_2_haplotype2-0000069_chr13
15.0
10.0
5.0
0.0
0.0
5.0
10.0
15.0
NA12885_2_haplotype2-0000069_chr13

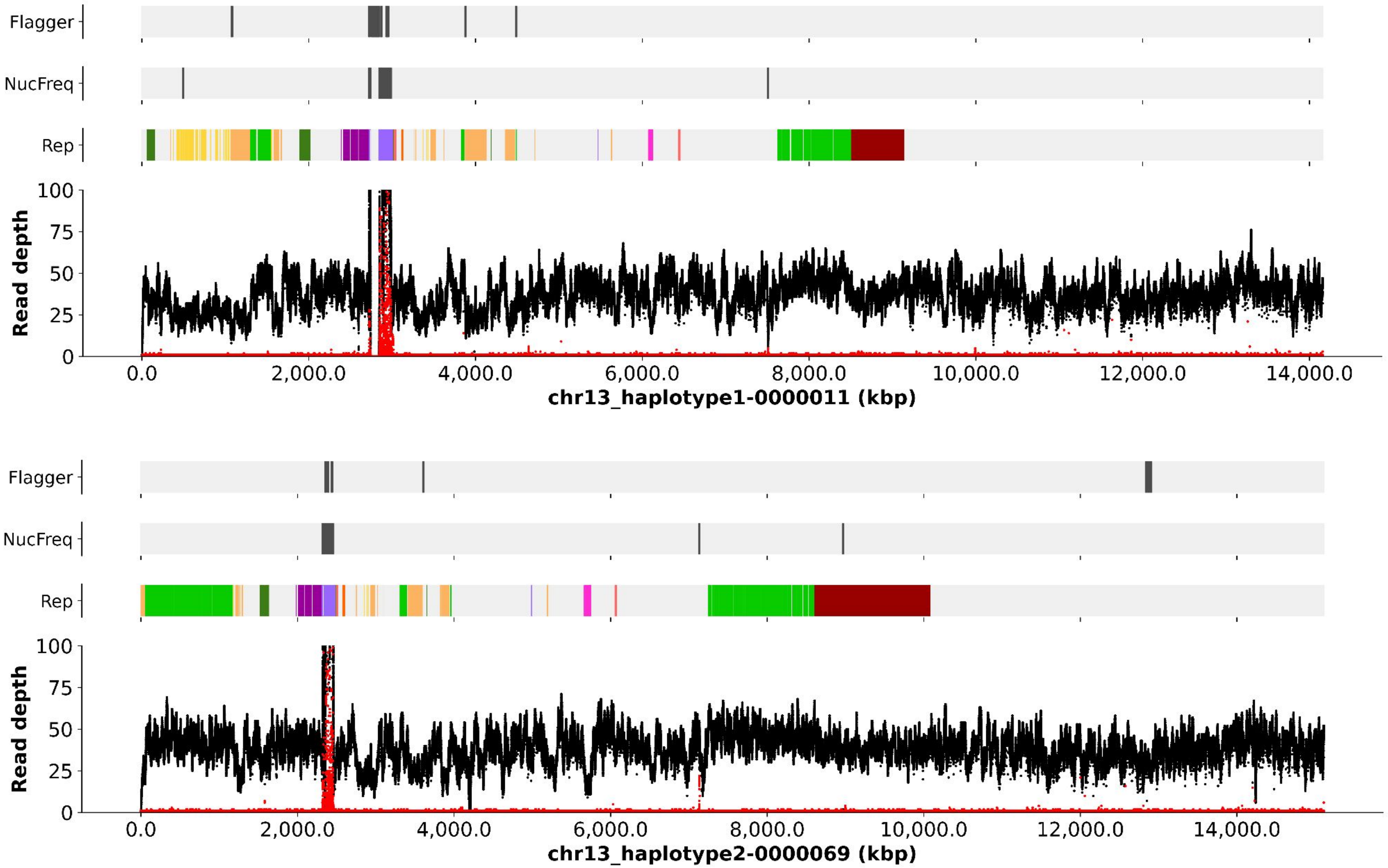

Flagger
NucFreq
Rep
Read depth
100
75
50
25
0
0.0
2,000.0
4,000.0
6,000.0
8,000.0
10,000.0
12,000.0
14,000.0
chr13_haplotype1-0000011 (kbp)
Flagger
NucFreq
Rep
Read depth
100
75
50
25
0
0.0
2,000.0
4,000.0
6,000.0
8,000.0
10,000.0
12,000.0
14,000.0
chr13_haplotype2-0000069 (kbp)

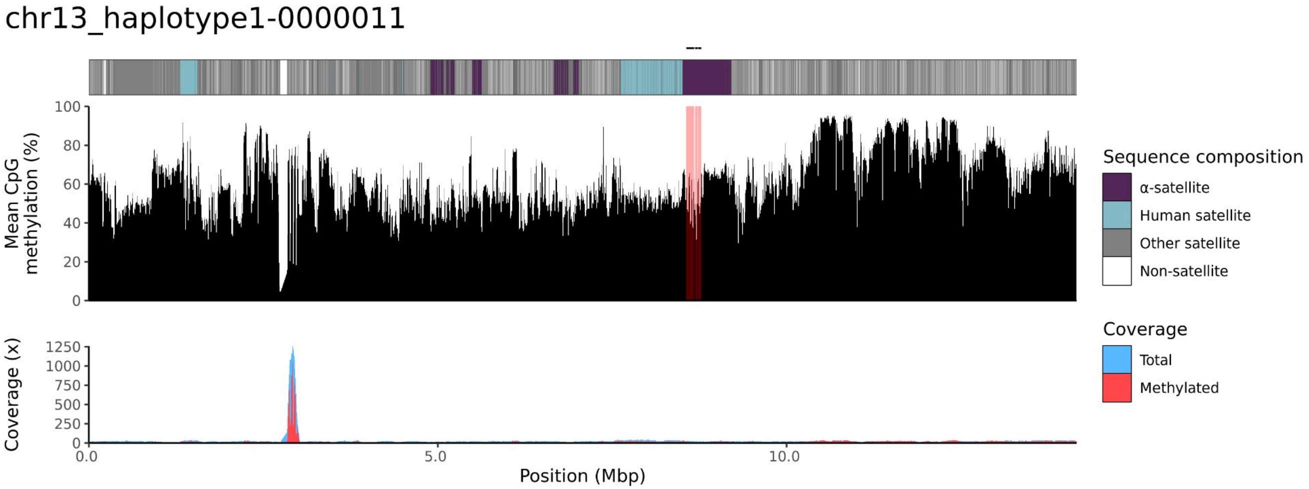
chr13_haplotype1-0000011
Mean CpG methylation (%)
100
80
60
40
20
0
Coverage (x)
1250
1000
750
500
250
0
0.0
5.0
10.0
Position (Mbp)
Sequence composition
α-satellite
Human satellite
Other satellite
Non-satellite
Coverage
Total
Methylated

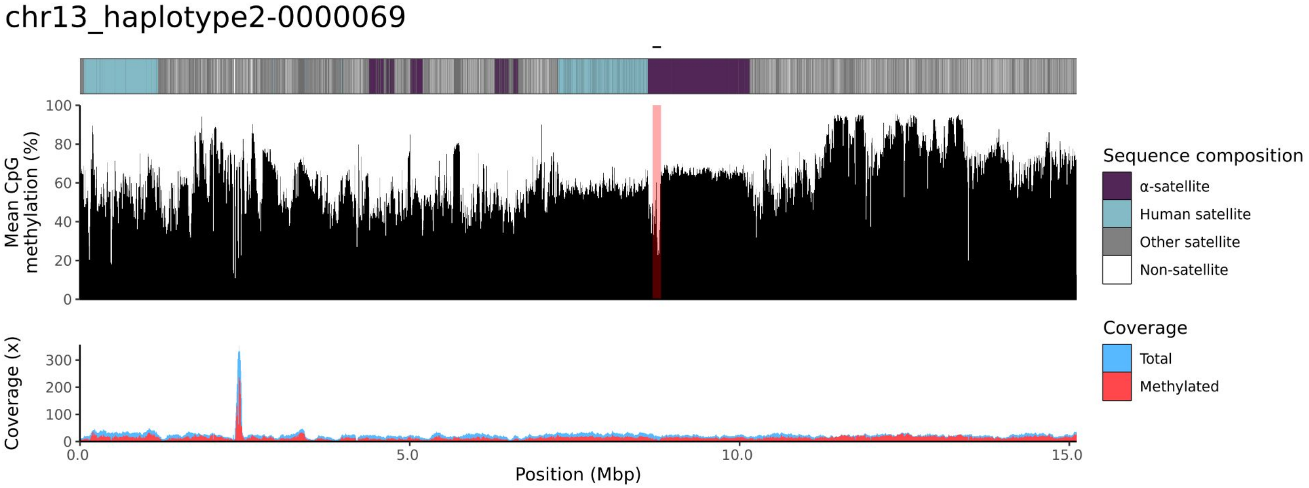
chr13_haplotype2-0000069
Mean CpG methylation (%)
100
80
60
40
20
0
Coverage (x)
300
200
100
0
0.0
5.0
10.0
15.0
Position (Mbp)
Sequence composition
α-satellite
Human satellite
Other satellite
Non-satellite
Coverage
Total
Methylated

chr14

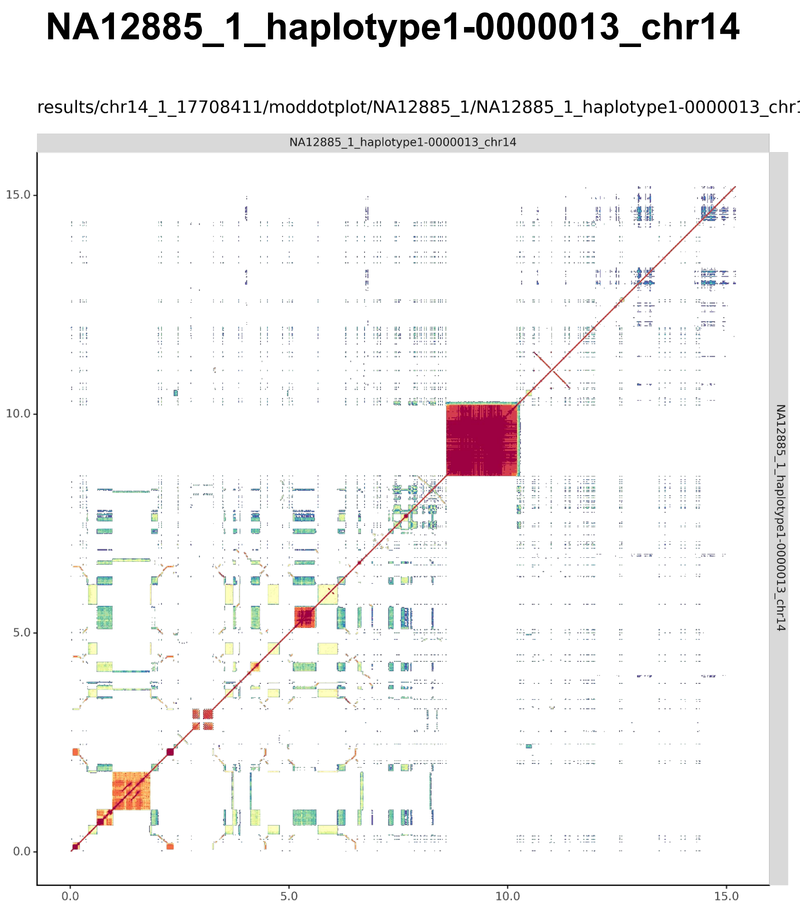

NA12885_1_haplotype1-0000013_chr14
results/chr14_1_17708411/moddotplot/NA12885_1/NA12885_1_haplotype1-0000013_chr1
NA12885_1_haplotype1-0000013_chr14
0.0
5.0
10.0
15.0
NA12885_1_haplotype1-0000013_chr14

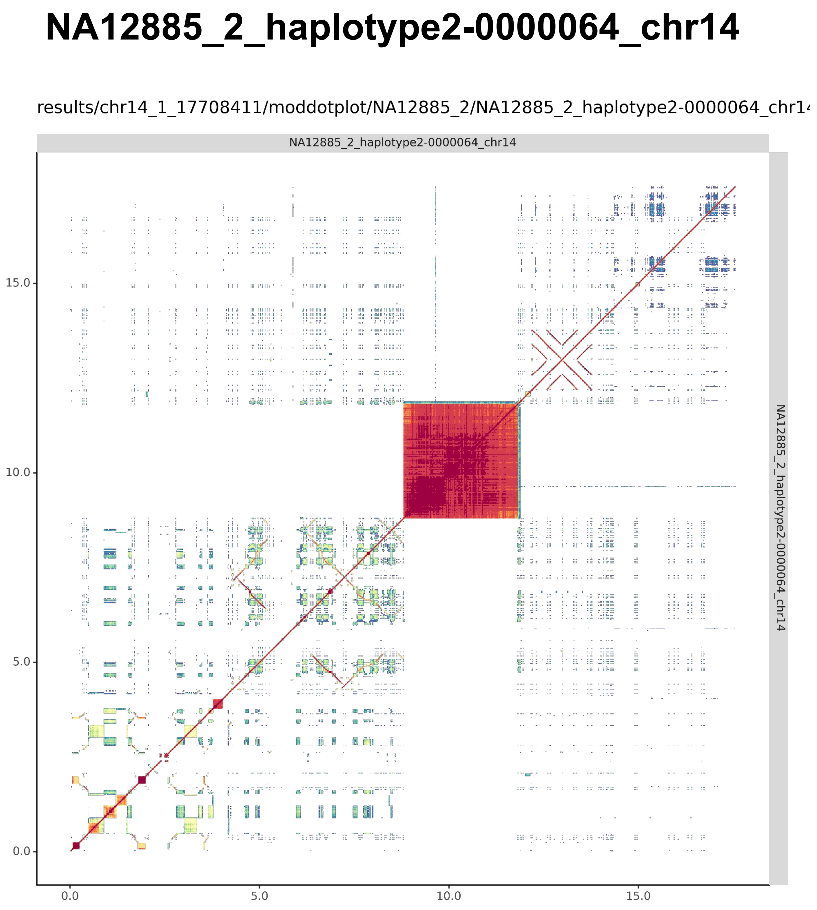

NA12885_2_haplotype2-0000064_chr14
results/chr14_1_17708411/moddotplot/NA12885_2/NA12885_2_haplotype2-0000064_chr1
NA12885_2_haplotype2-0000064_chr14
0.0
5.0
10.0
15.0
NA12885_2_haplotype2-0000064_chr14

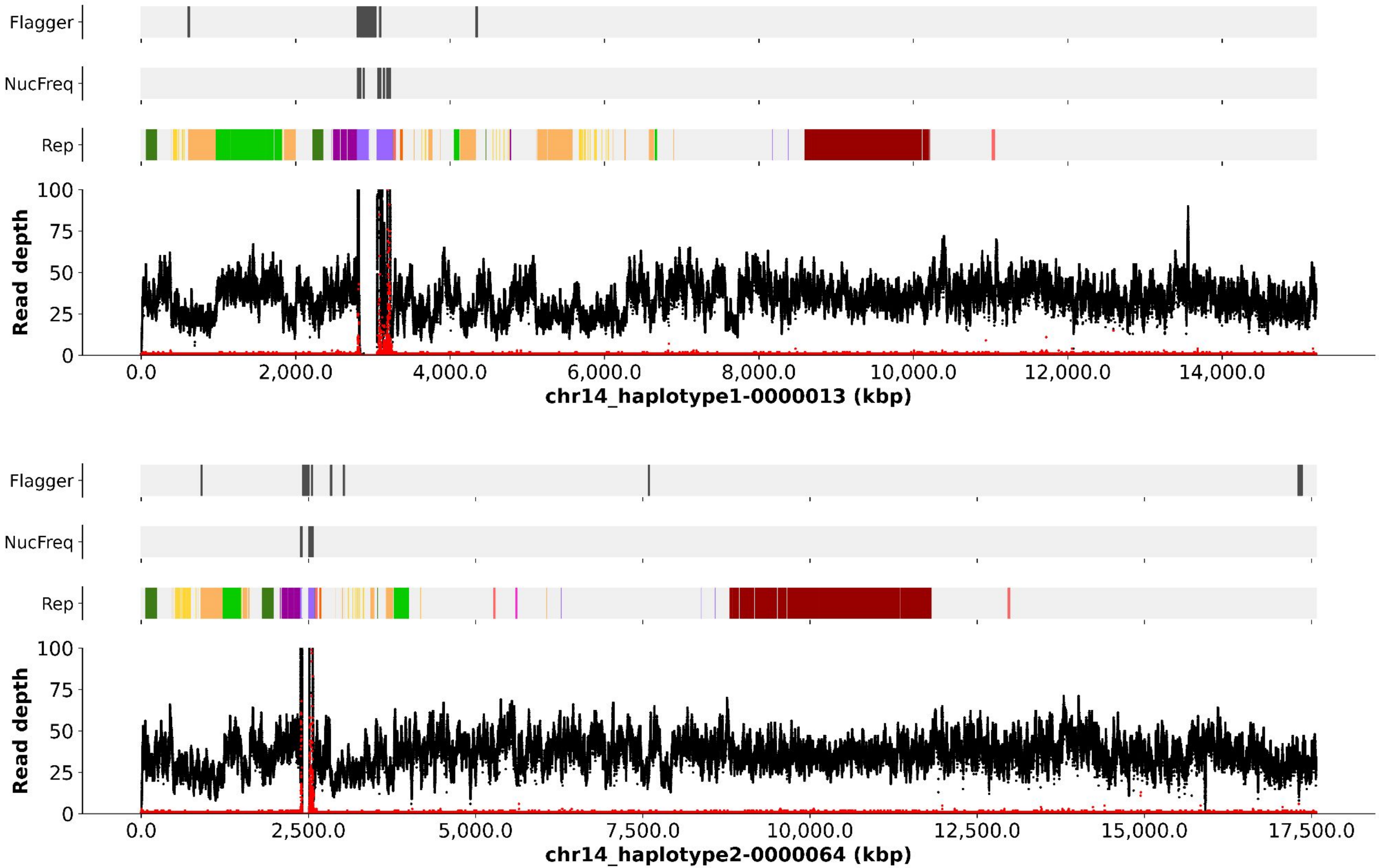
Flagger
NucFreq
Rep
Read depth
100
75
50
25
0
0.0
2,000.0
4,000.0
6,000.0
8,000.0
10,000.0
12,000.0
14,000.0
chr14_haplotype1-0000013 (kbp)
Flagger
NucFreq
Rep
Read depth
100
75
50
25
0
0.0
2,500.0
5,000.0
7,500.0
10,000.0
12,500.0
15,000.0
17,500.0
chr14_haplotype2-0000064 (kbp)

chr14_haplotype1-0000013

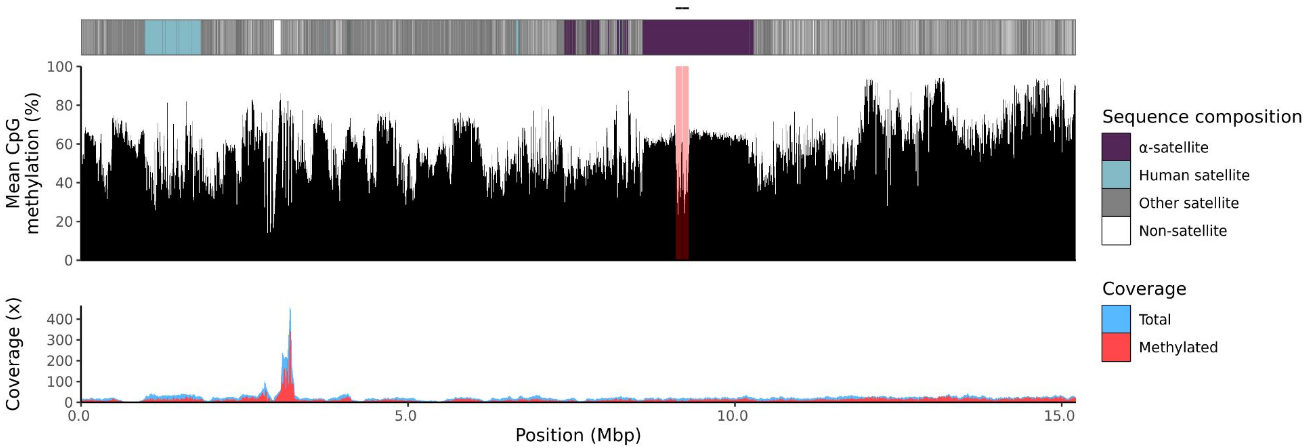

chr14_haplotype2-0000064

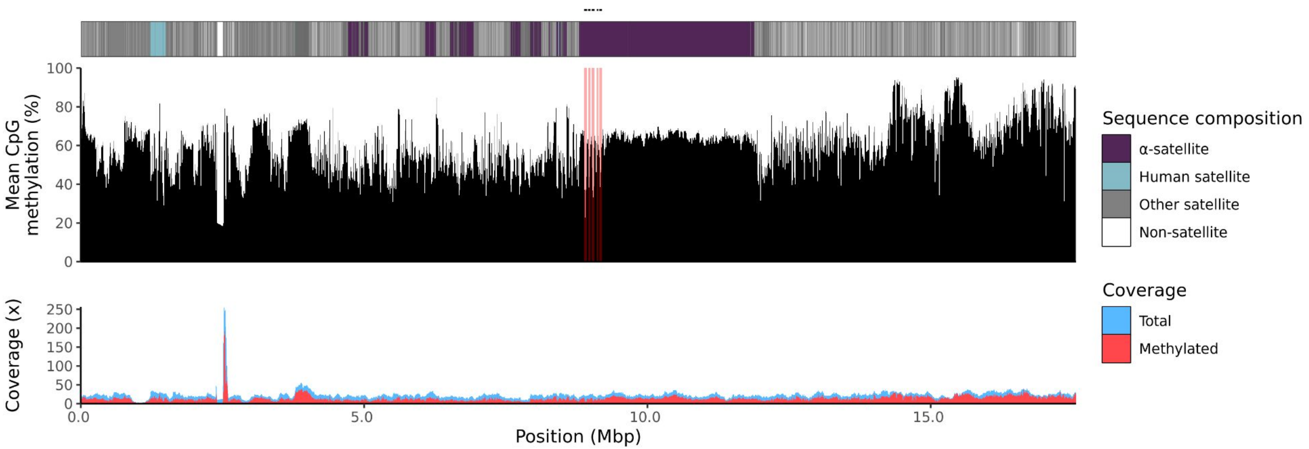

chr15

NA12885_1_haplotype1-0000016_chr15

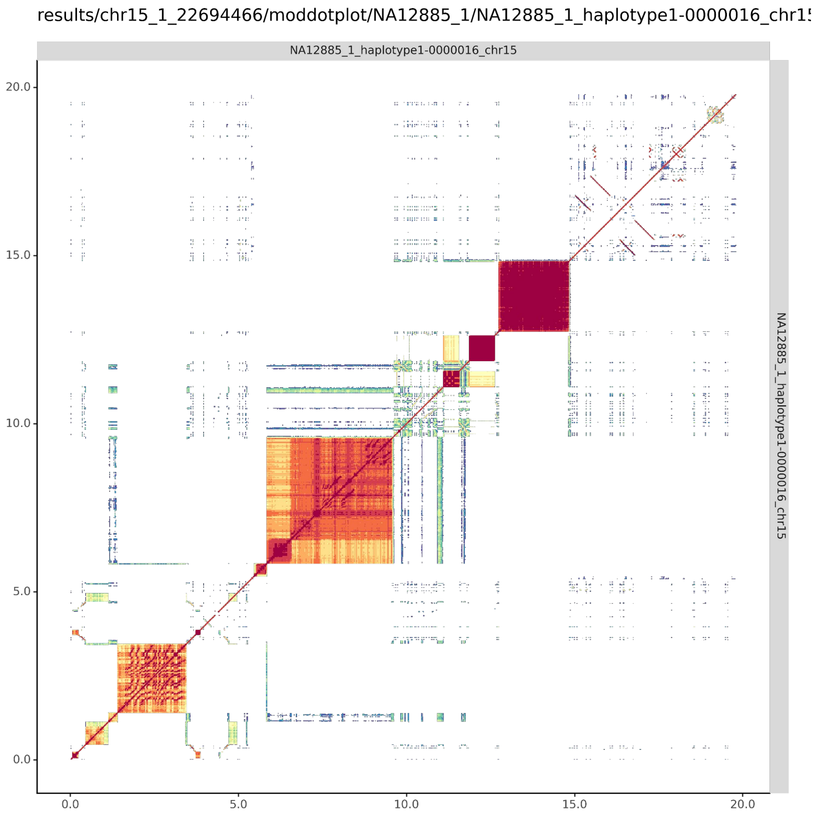

NA12885_2_haplotype2-0000072_chr15

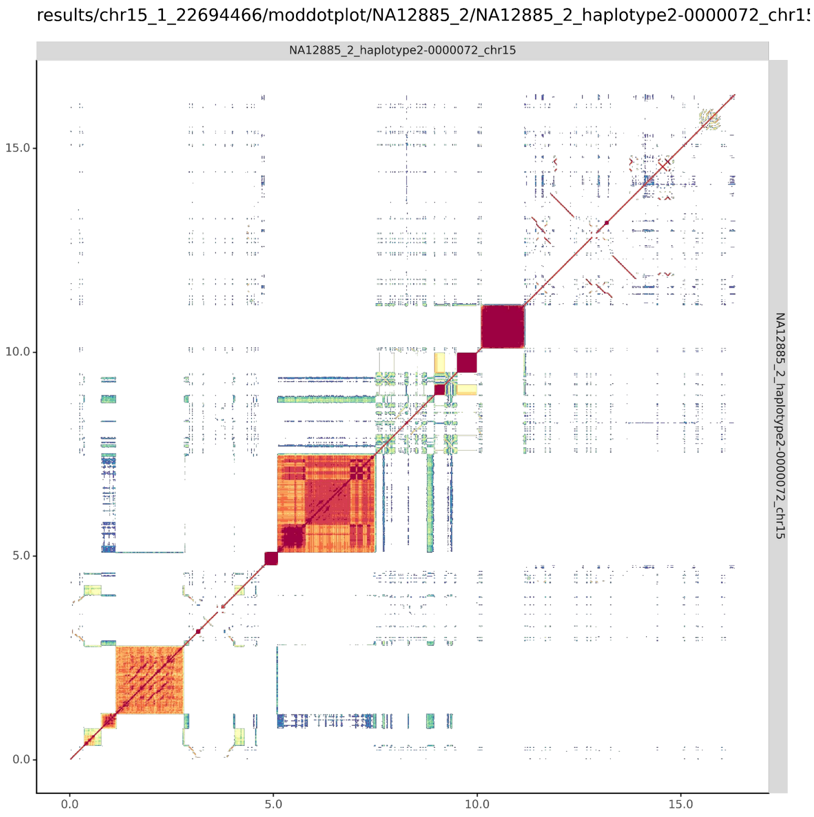

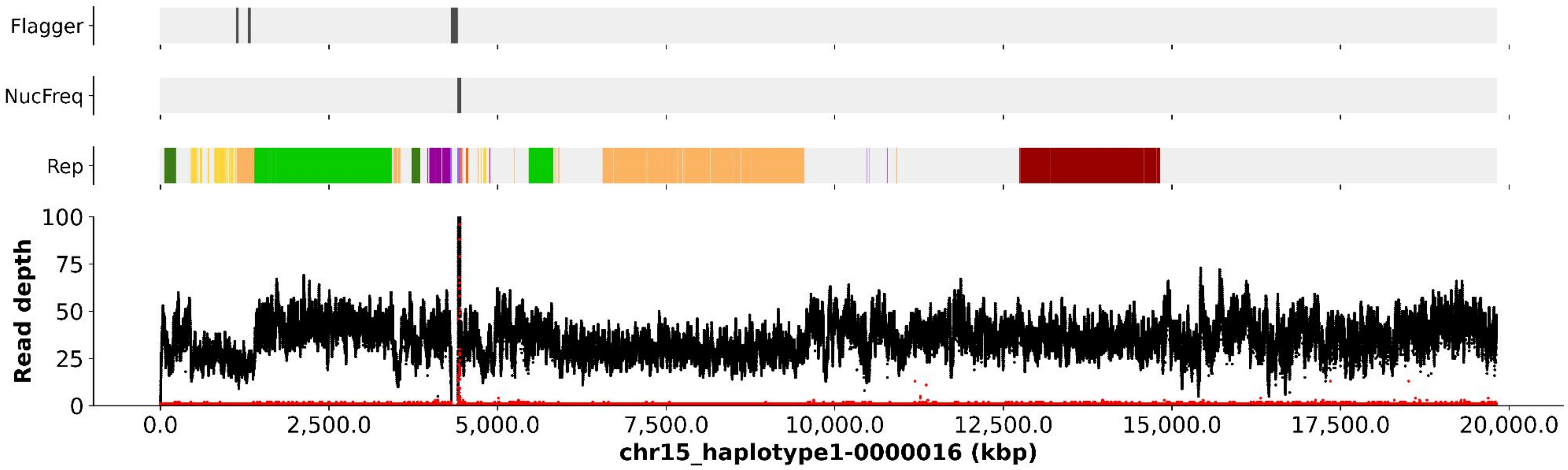

Flagger
NucFreq
Rep
Read depth
100
75
50
25
0
0.0
2,500.0
5,000.0
7,500.0
10,000.0
12,500.0
15,000.0
17,500.0
20,000.0
chr15_haplotype1-0000016 (kbp)

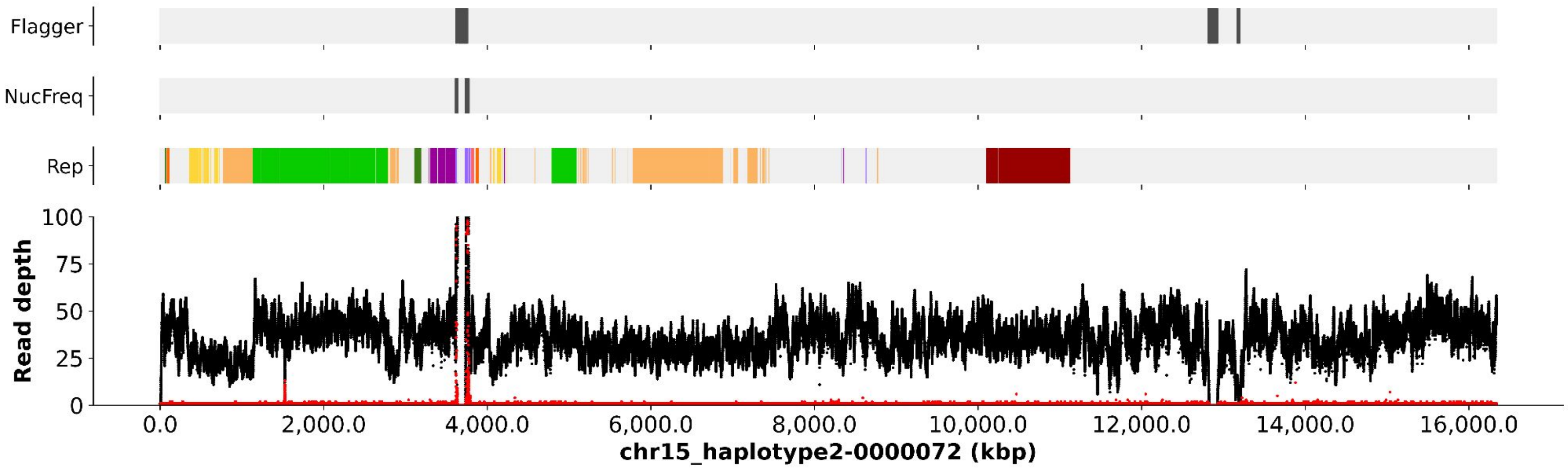

Flagger
NucFreq
Rep
Read depth
100
75
50
25
0
0.0
2,000.0
4,000.0
6,000.0
8,000.0
10,000.0
12,000.0
14,000.0
16,000.0
chr15_haplotype2-0000072 (kbp)

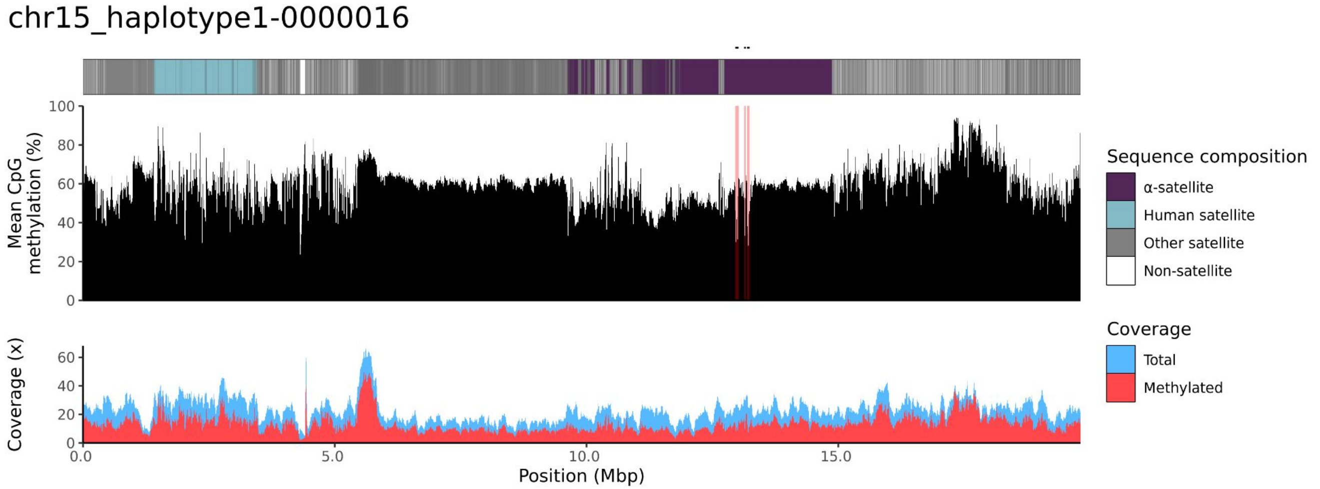

chr15_haplotype1-0000016
100
80
60
40
20
0
Mean CpG methylation (%)
Sequence composition
α-satellite
Human satellite
Other satellite
Non-satellite
Coverage (x)
60
40
20
0
Coverage
Total
Methylated
0.0
5.0
10.0
15.0
Position (Mbp)

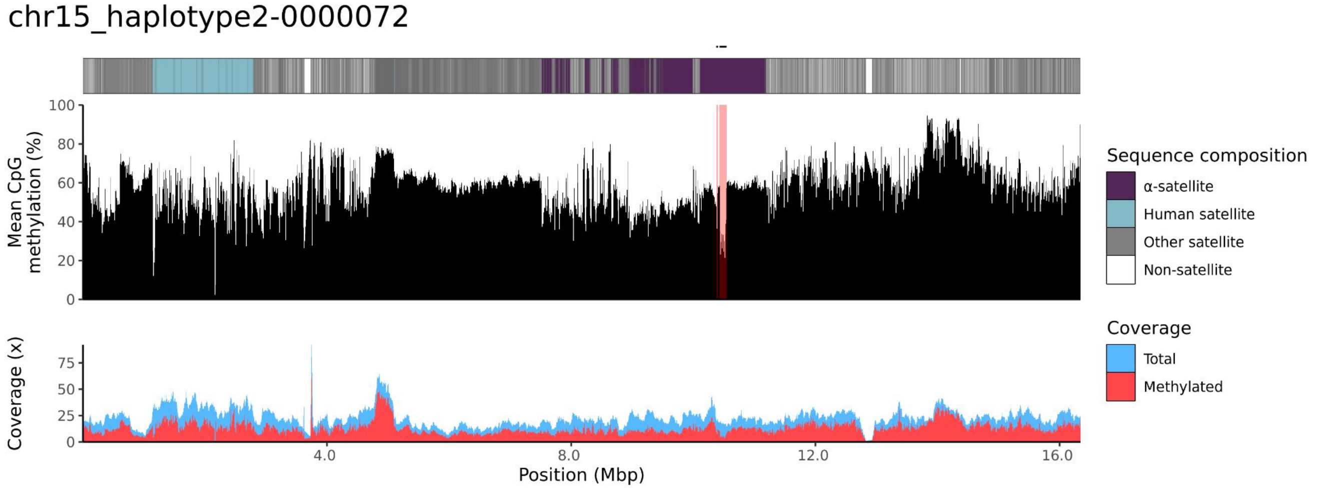

chr15_haplotype2-0000072
100
80
60
40
20
0
Mean CpG methylation (%)
Sequence composition
α-satellite
Human satellite
Other satellite
Non-satellite
Coverage (x)
75
50
25
0
Coverage
Total
Methylated
4.0
8.0
12.0
16.0
Position (Mbp)

chr21

NA12885_1_haplotype1-0000025_chr21

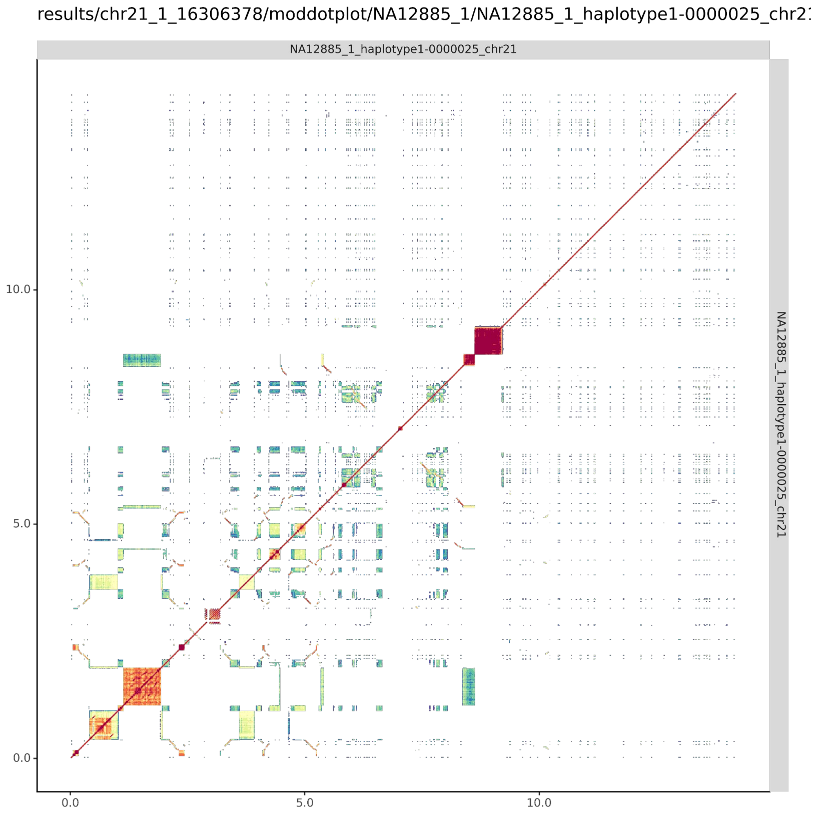

NA12885_2_haplotype2-0000078_chr21

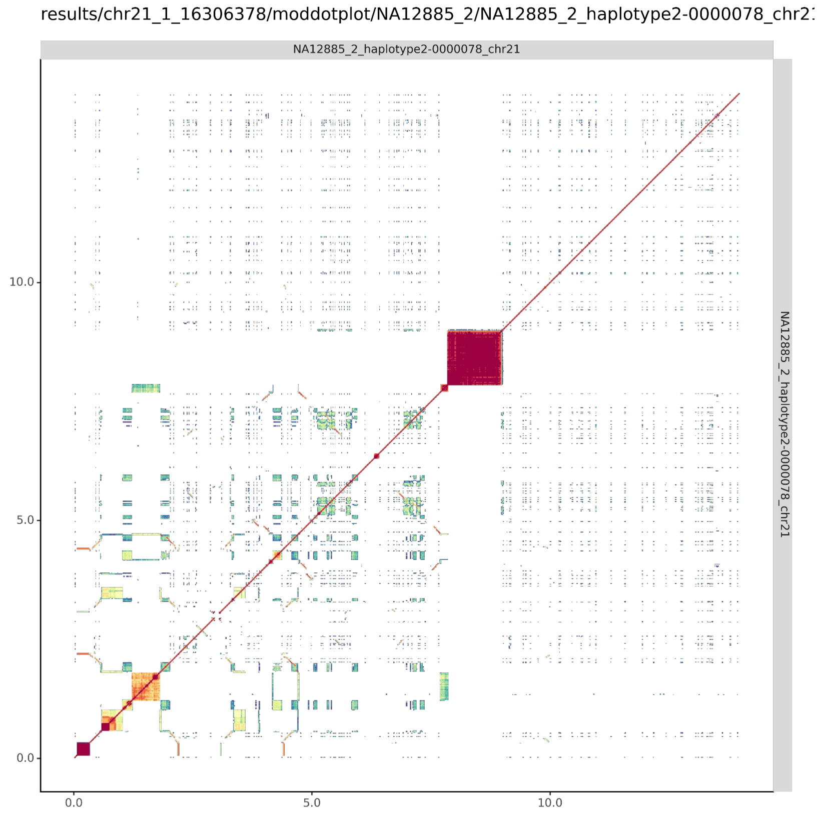

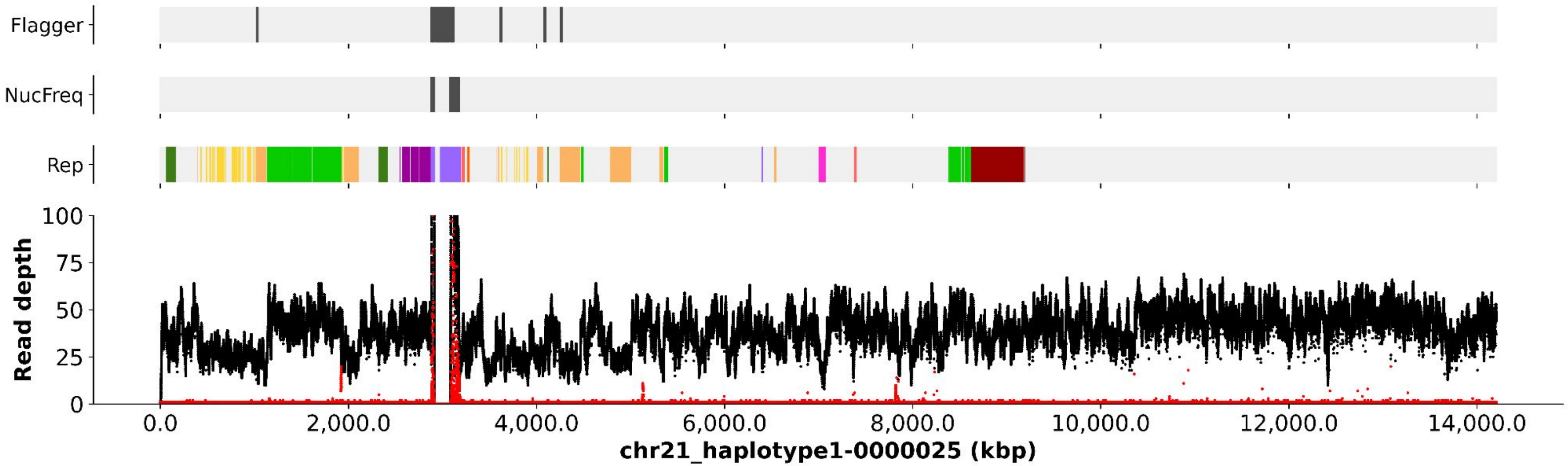
Flagger
NucFreq
Rep
Read depth
100
75
50
25
0
0.0
2,000.0
4,000.0
6,000.0
8,000.0
10,000.0
12,000.0
14,000.0
chr21_haplotype1-0000025 (kbp)

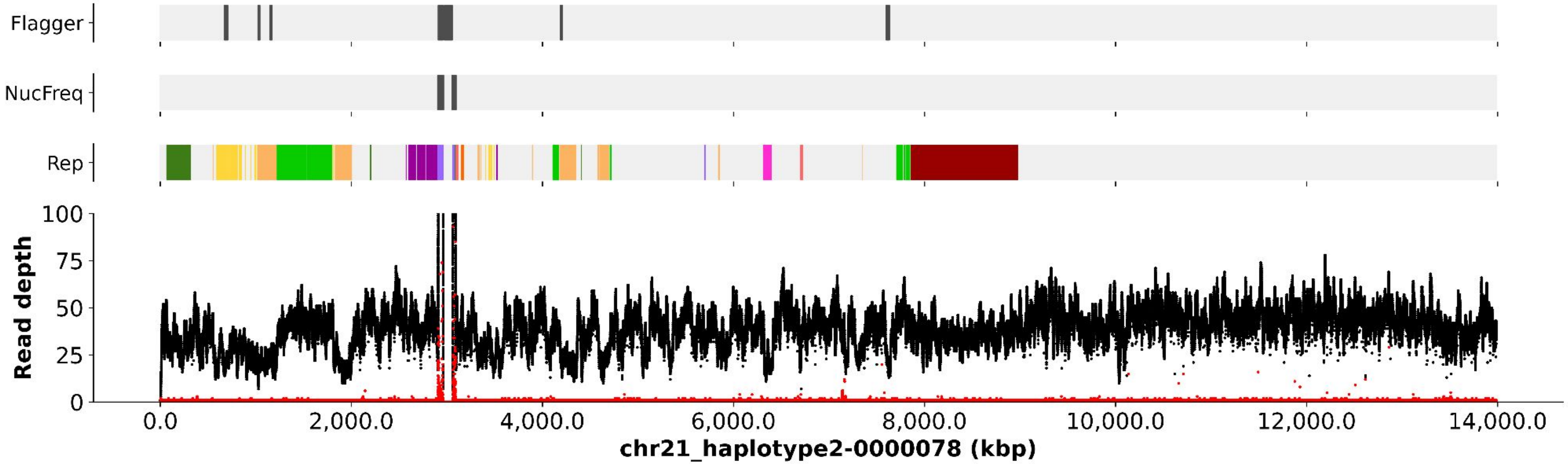
Flagger
NucFreq
Rep
Read depth
100
75
50
25
0
0.0
2,000.0
4,000.0
6,000.0
8,000.0
10,000.0
12,000.0
14,000.0
chr21_haplotype2-0000078 (kbp)

chr21_haplotype1-0000025

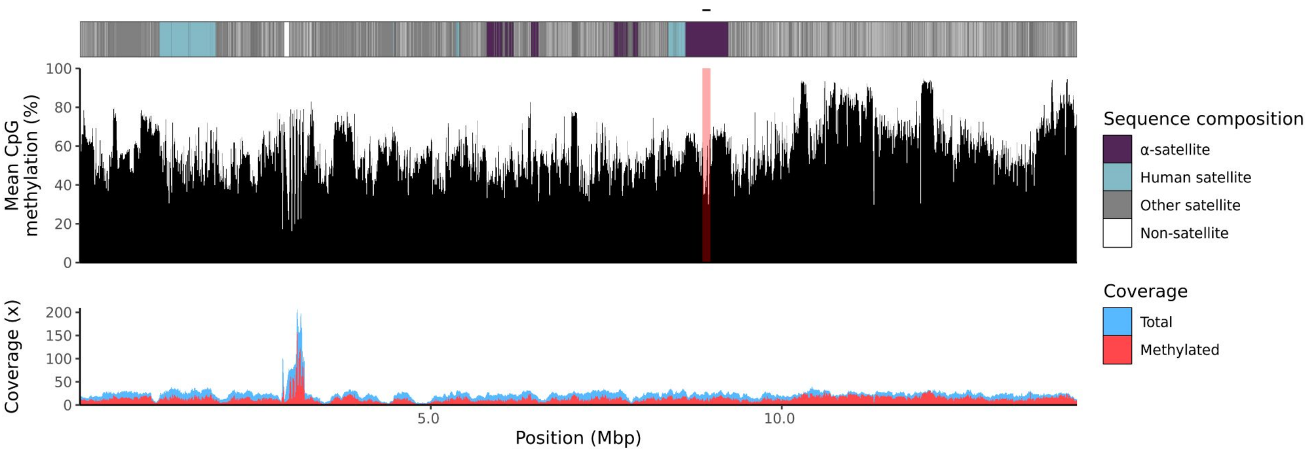

chr21_haplotype2-0000078

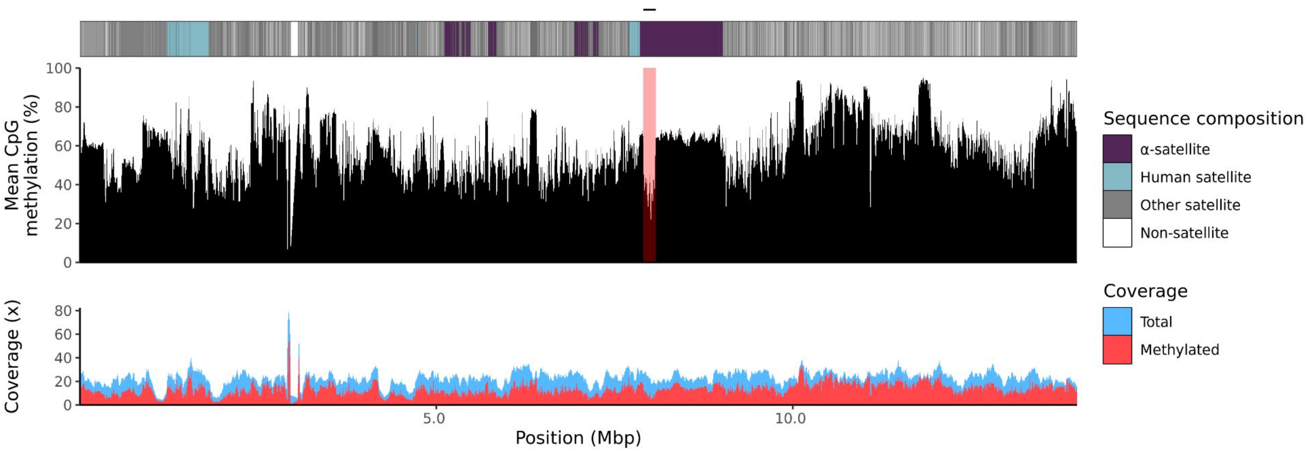

chr22

NA12885_1_haplotype1-0000009_chr22

results/chr22_1_20711065/moddotplot/NA12885_1/NA12885_1_haplotype1-0000009_chr22

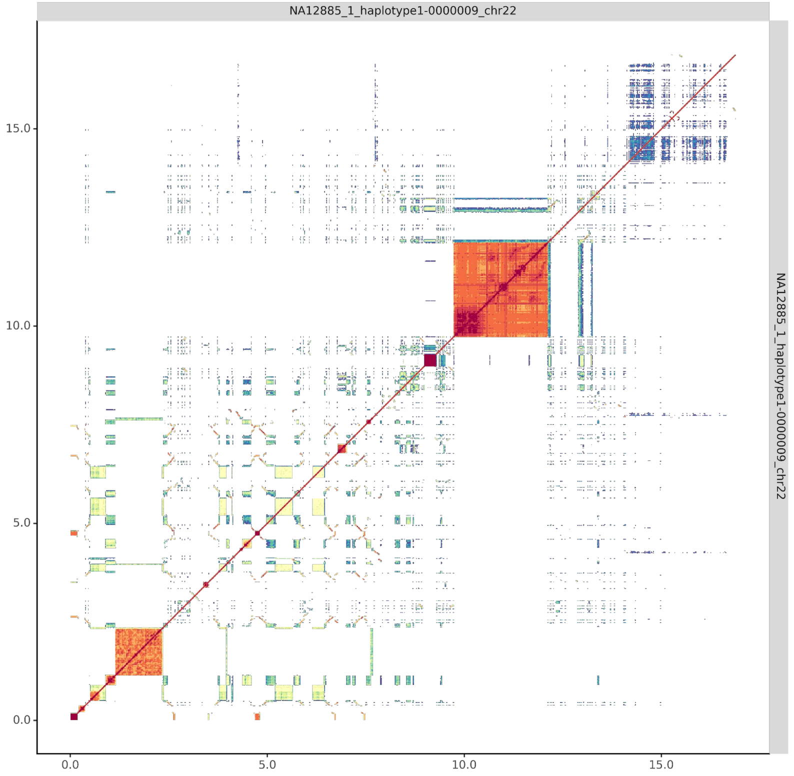

NA12885_2_haplotype2-0000067_chr22

results/chr22_1_20711065/moddotplot/NA12885_2/NA12885_2_haplotype2-0000067_chr22

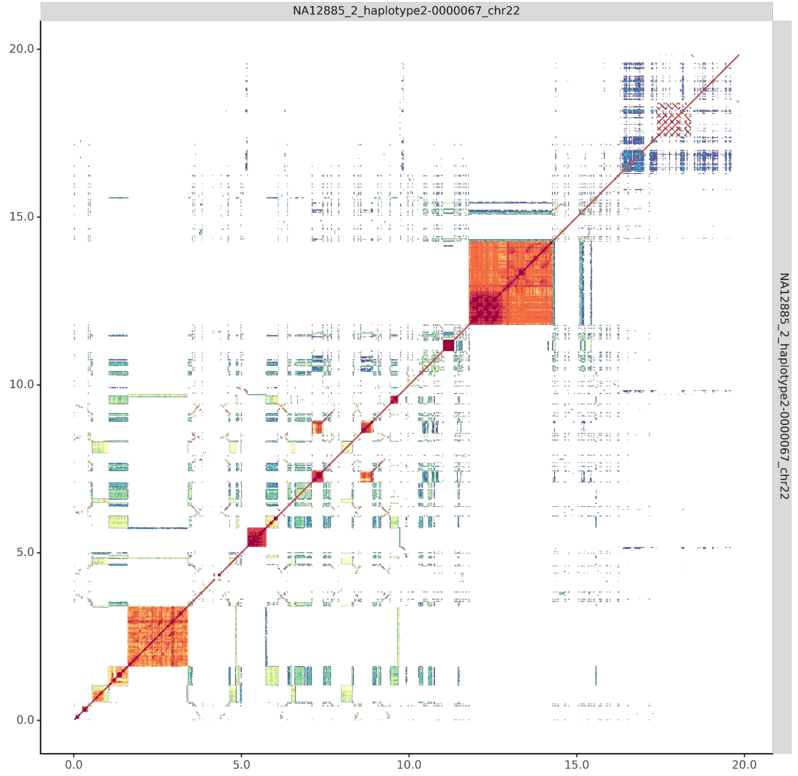

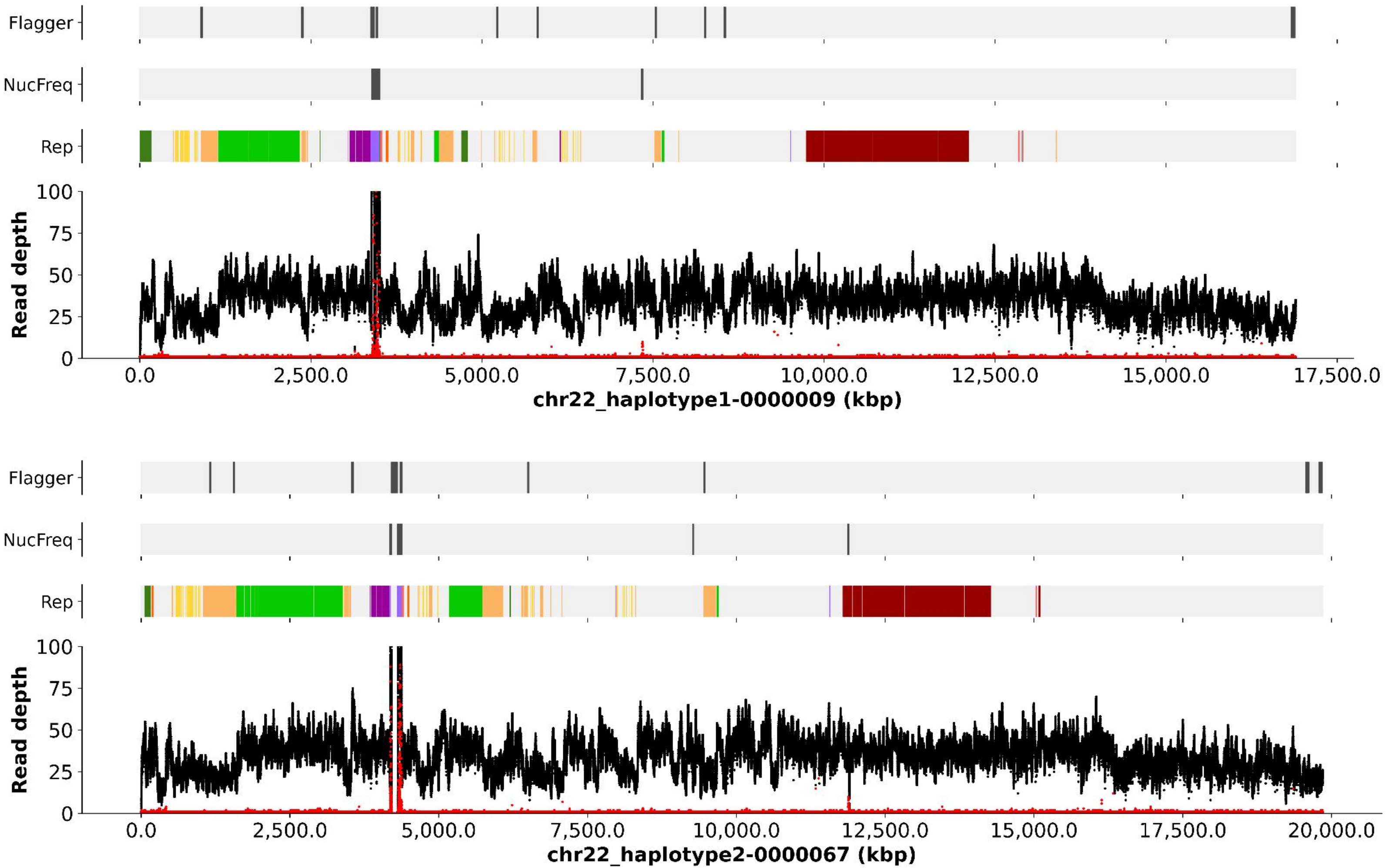
Flagger
NucFreq
Rep
Read depth
100
75
50
25
0
0.0
2,500.0
5,000.0
7,500.0
10,000.0
12,500.0
15,000.0
17,500.0
chr22_haplotype1-0000009 (kbp)
Flagger
NucFreq
Rep
Read depth
100
75
50
25
0
0.0
2,500.0
5,000.0
7,500.0
10,000.0
12,500.0
15,000.0
17,500.0
20,000.0
chr22_haplotype2-0000067 (kbp)

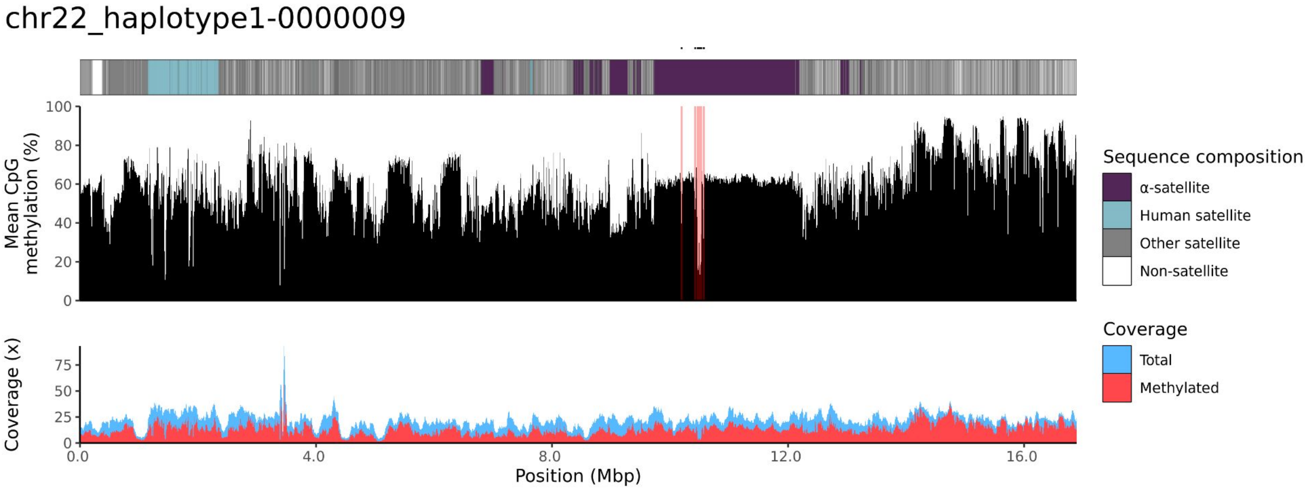
chr22_haplotype1-0000009
Mean CpG methylation (%)
100
80
60
40
20
0
Coverage (x)
75
50
25
0
0.0
4.0
8.0
12.0
16.0
Position (Mbp)
Sequence composition
α-satellite
Human satellite
Other satellite
Non-satellite
Coverage
Total
Methylated

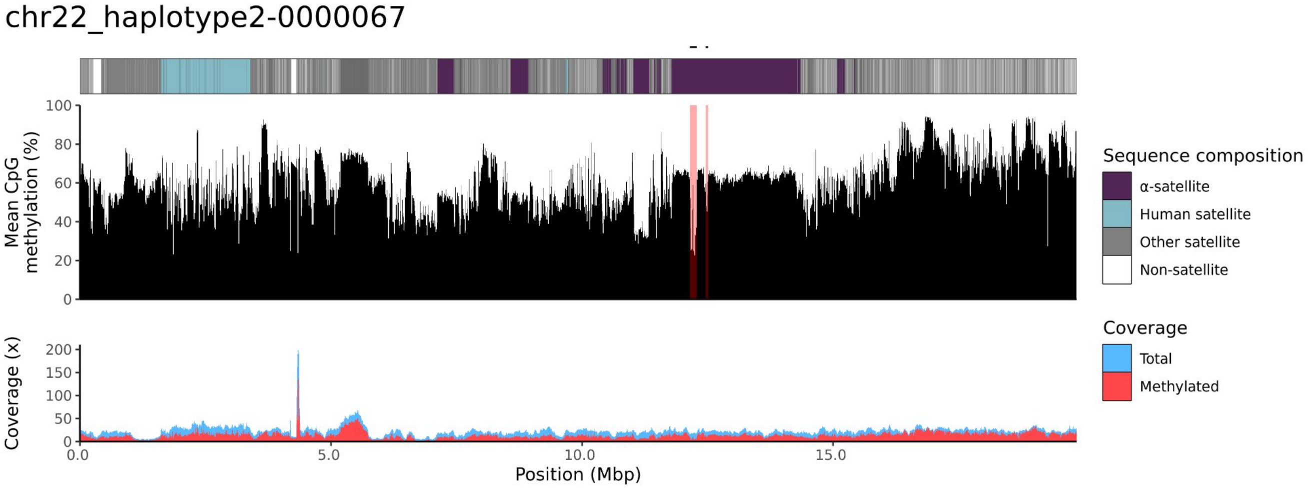
chr22_haplotype2-0000067
Mean CpG methylation (%)
100
80
60
40
20
0
Coverage (x)
200
150
100
50
0
0.0
5.0
10.0
15.0
Position (Mbp)
Sequence composition
α-satellite
Human satellite
Other satellite
Non-satellite
Coverage
Total
Methylated

NA12886

chr13

NA12886_1_haplotype1-0000017_chr13

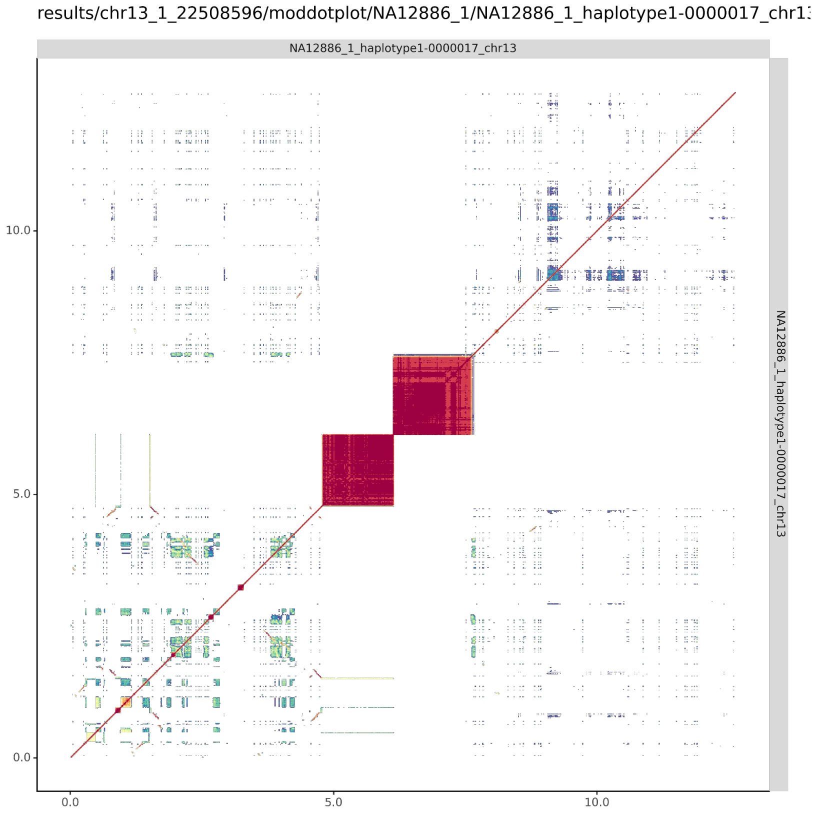

NA12886_2_haplotype2-0000086_chr13

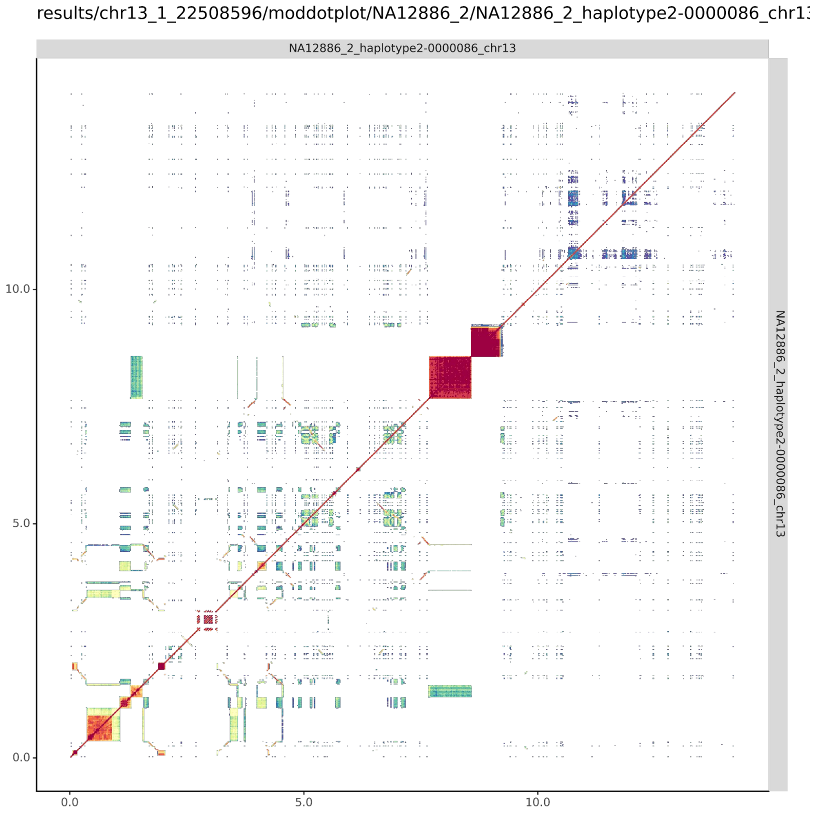

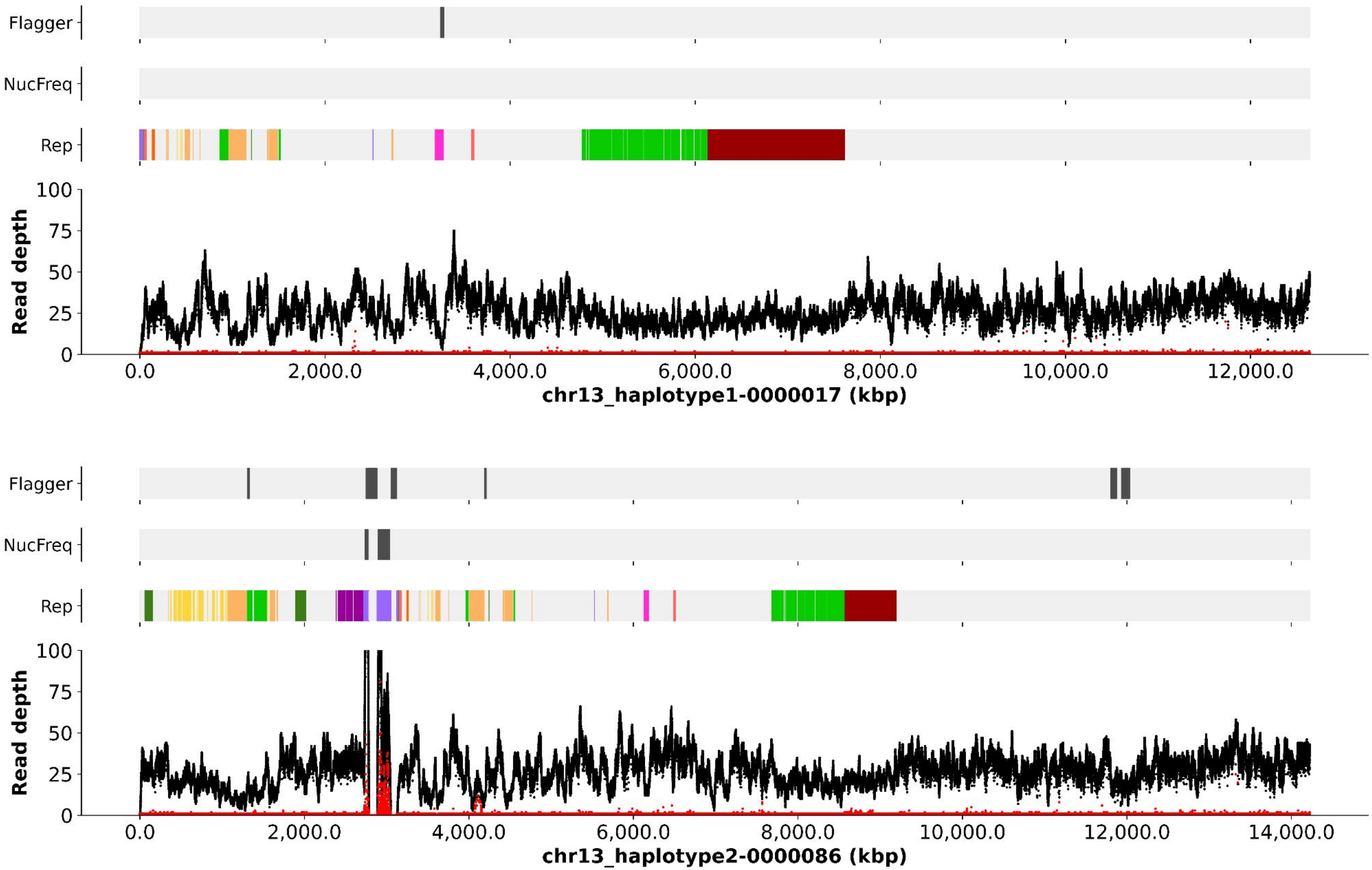

Flagger
NucFreq
Rep
Read depth
100
75
50
25
0
0.0
2,000.0
4,000.0
6,000.0
8,000.0
10,000.0
12,000.0
chr13_haplotype1-0000017 (kbp)
Flagger
NucFreq
Rep
Read depth
100
75
50
25
0
0.0
2,000.0
4,000.0
6,000.0
8,000.0
10,000.0
12,000.0
14,000.0
chr13_haplotype2-0000086 (kbp)

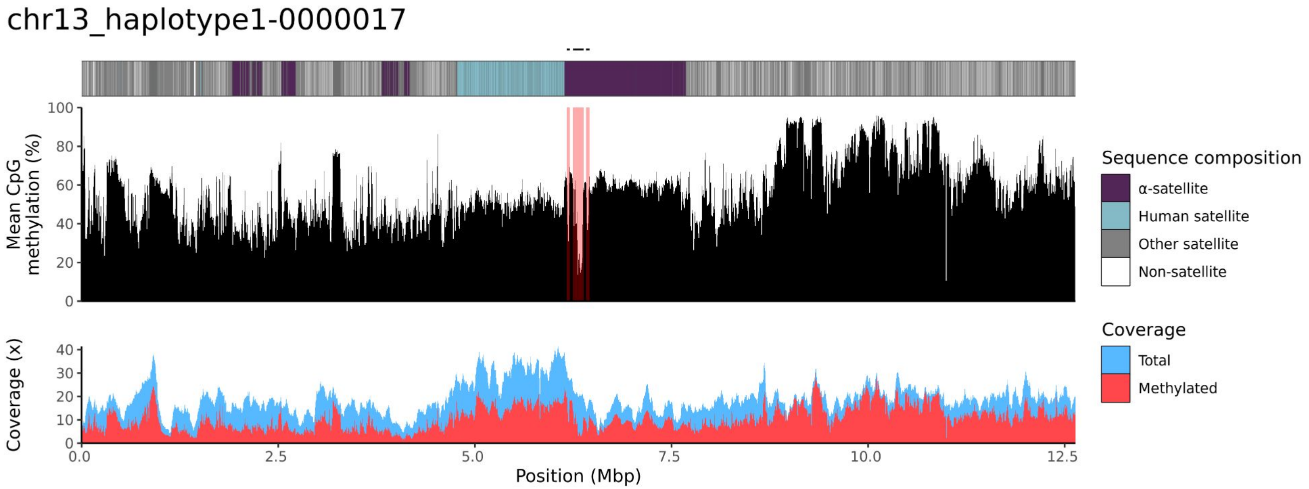
chr13_haplotype1-0000017
Mean CpG methylation (%)
Coverage (x)
Position (Mbp)
Sequence composition
α-satellite
Human satellite
Other satellite
Non-satellite
Coverage
Total
Methylated

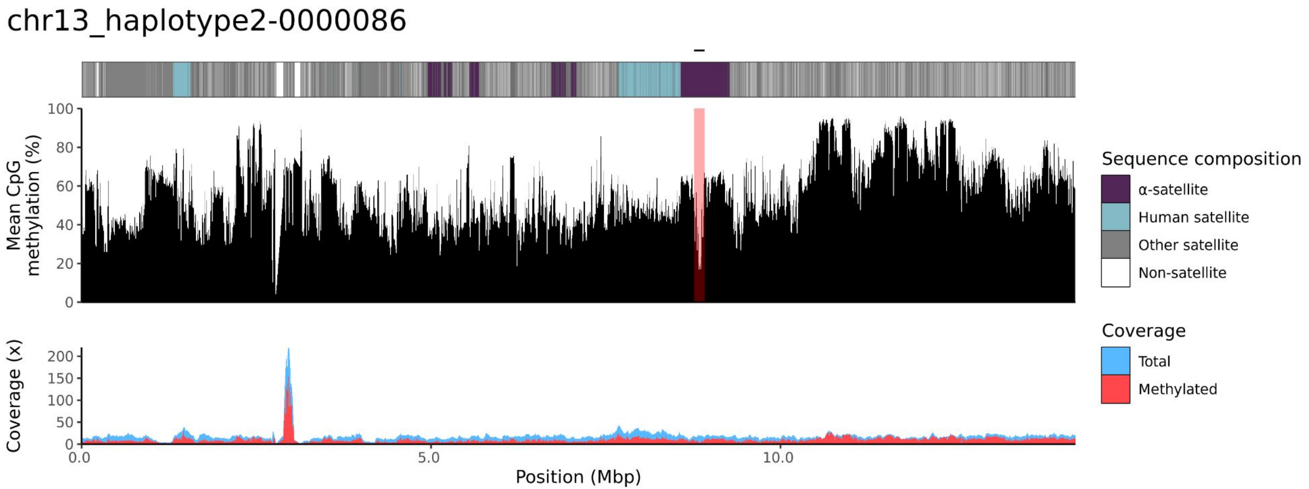
chr13_haplotype2-0000086
Mean CpG methylation (%)
Coverage (x)
Position (Mbp)
Sequence composition
α-satellite
Human satellite
Other satellite
Non-satellite
Coverage
Total
Methylated

chr14

NA12886_1_haplotype1-0000009_chr14

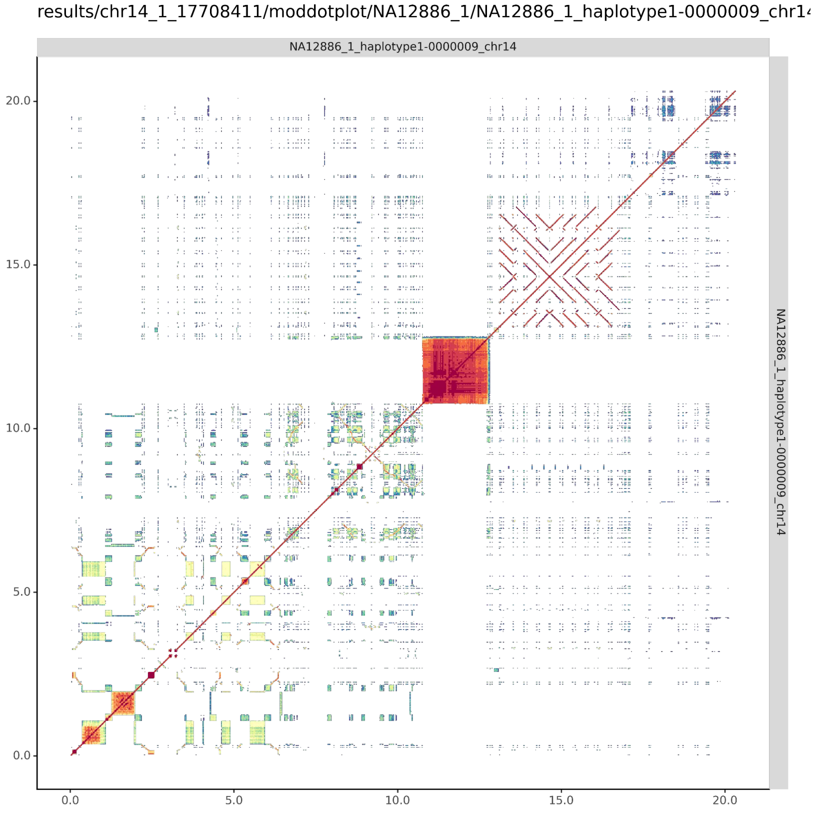

NA12886_2_haplotype2-0000085_chr14

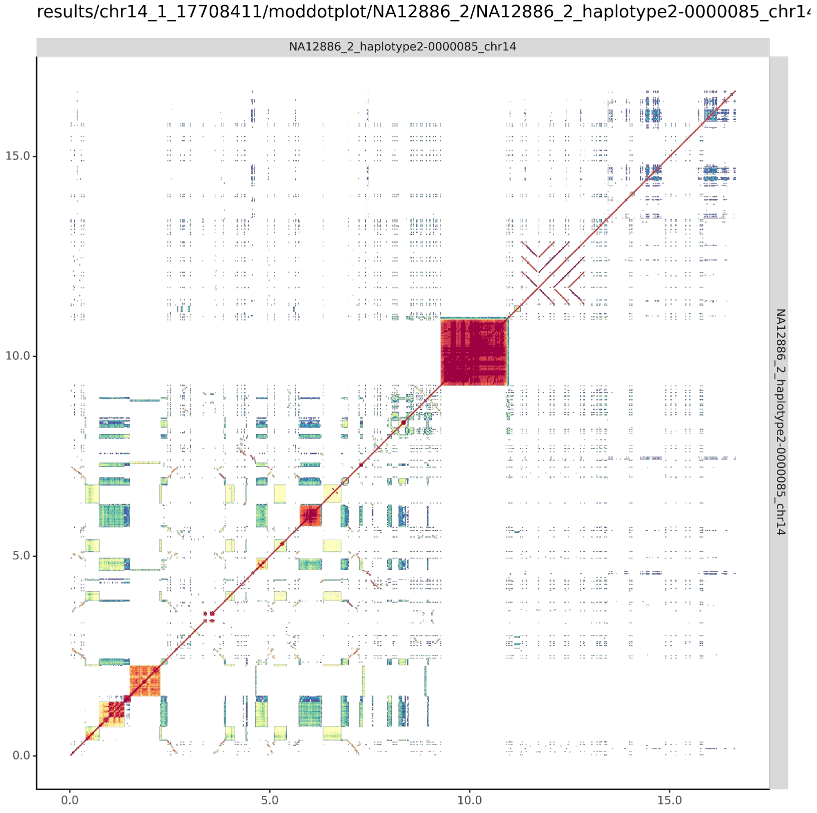

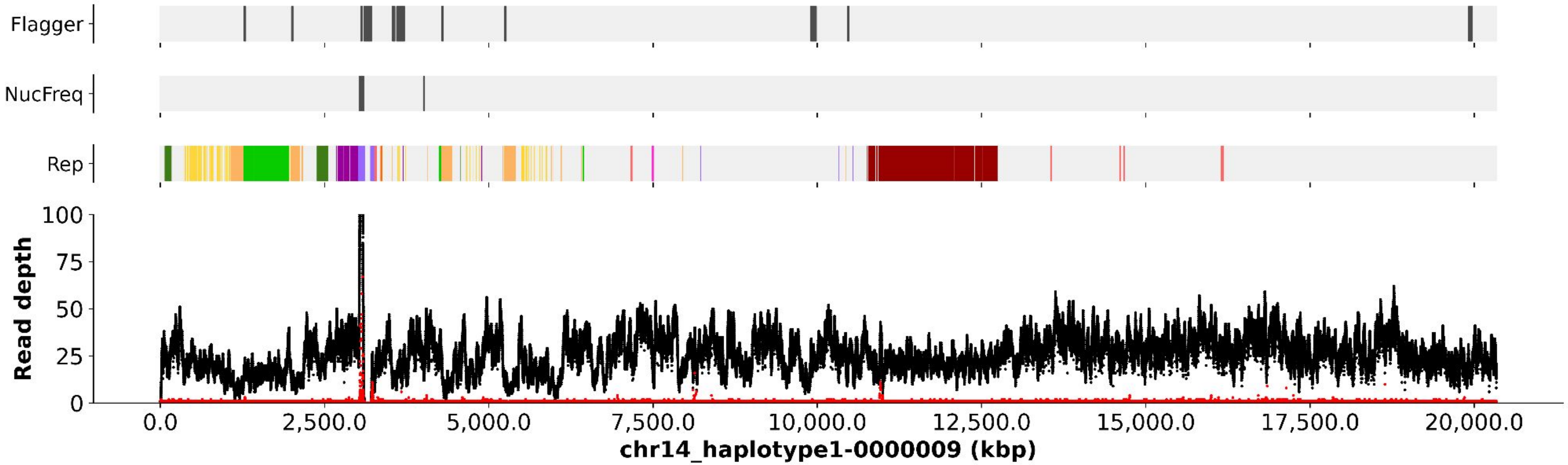
Flagger
NucFreq
Rep
Read depth
100
75
50
25
0
0.0
2,500.0
5,000.0
7,500.0
10,000.0
12,500.0
15,000.0
17,500.0
20,000.0
chr14_haplotype1-0000009 (kbp)

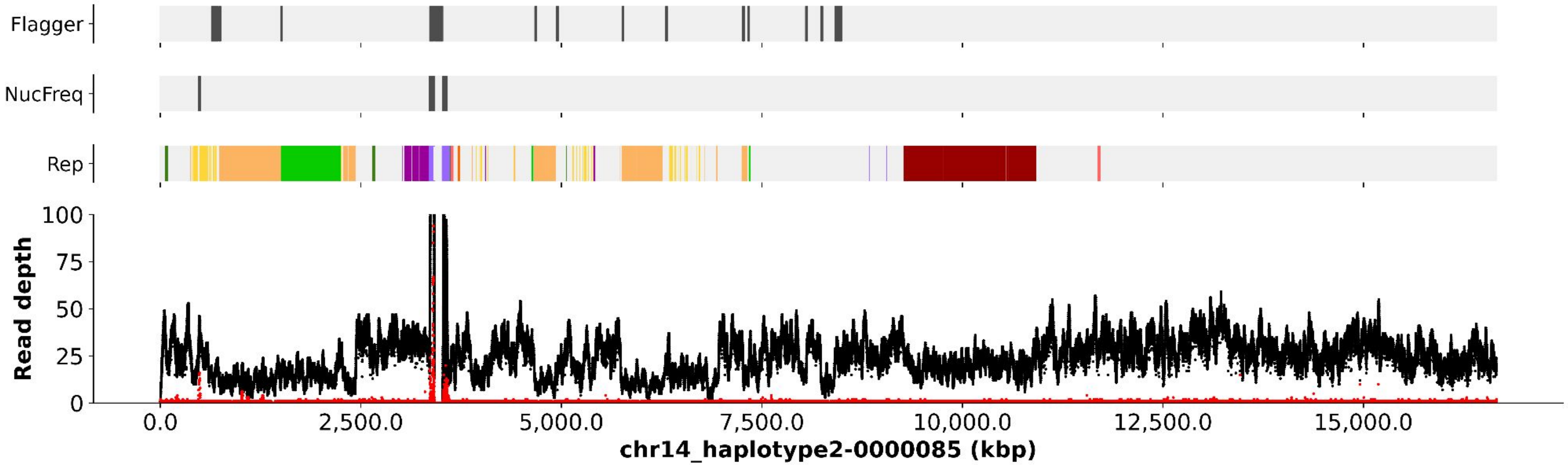
Flagger
NucFreq
Rep
Read depth
100
75
50
25
0
0.0
2,500.0
5,000.0
7,500.0
10,000.0
12,500.0
15,000.0
chr14_haplotype2-0000085 (kbp)

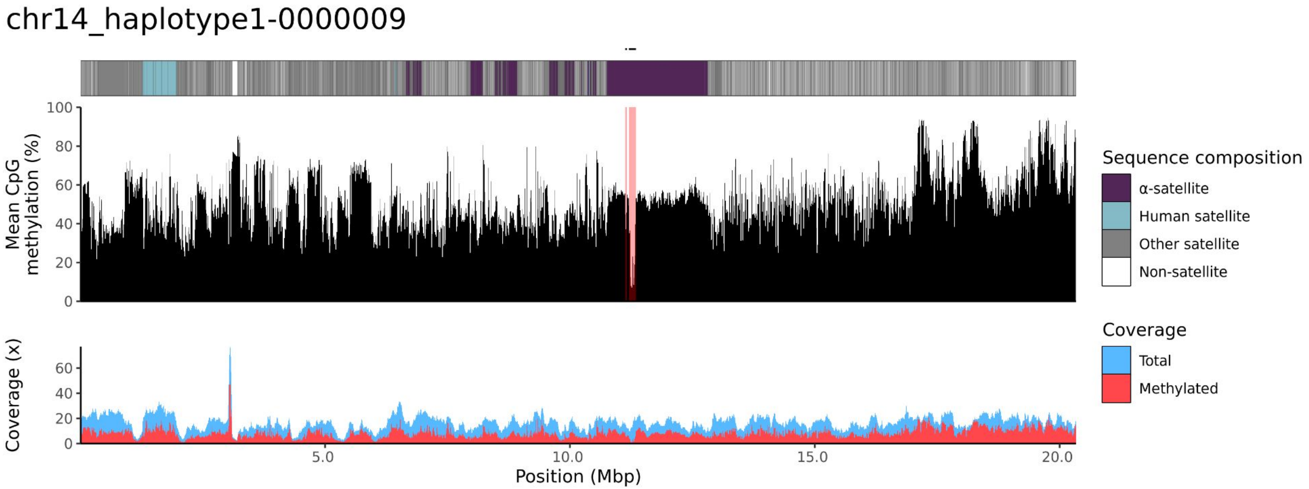
chr14_haplotype1-0000009
Mean CpG methylation (%)
100
80
60
40
20
0
Coverage (x)
60
40
20
0
5.0
10.0
15.0
20.0
Position (Mbp)
Sequence composition
α-satellite
Human satellite
Other satellite
Non-satellite
Coverage
Total
Methylated

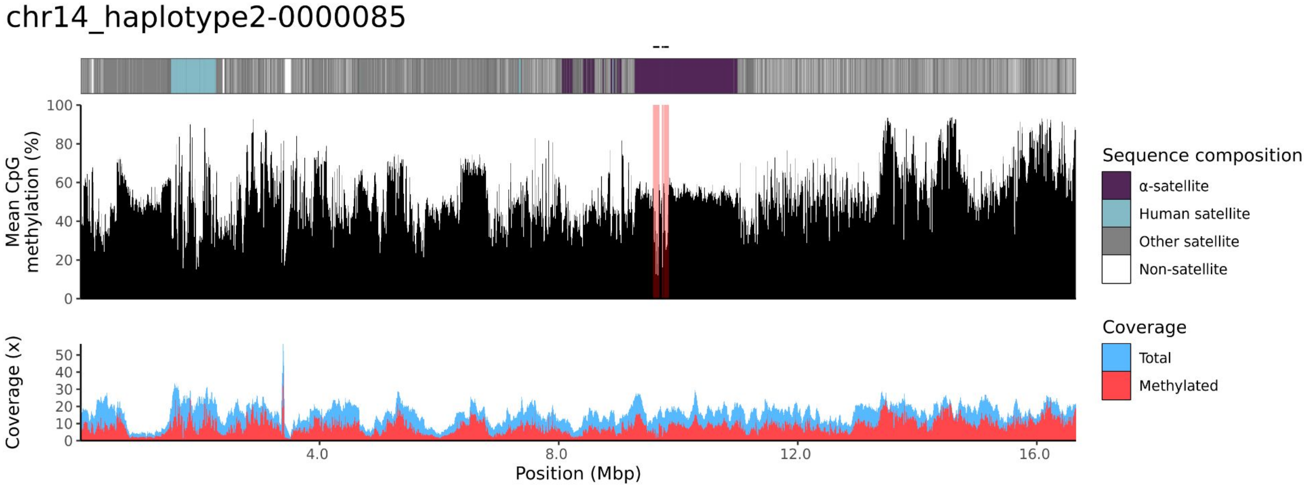
chr14_haplotype2-0000085
Mean CpG methylation (%)
100
80
60
40
20
0
Coverage (x)
50
40
30
20
10
0
4.0
8.0
12.0
16.0
Position (Mbp)
Sequence composition
α-satellite
Human satellite
Other satellite
Non-satellite
Coverage
Total
Methylated

chr15

NA12886_1_haplotype1-0000027_chr15

NA12886_2_haplotype2-0000095_chr15

results/chr15_1_22694466/moddotplot/NA12886_1/NA12886_1_haplotype1-0000027_chr1

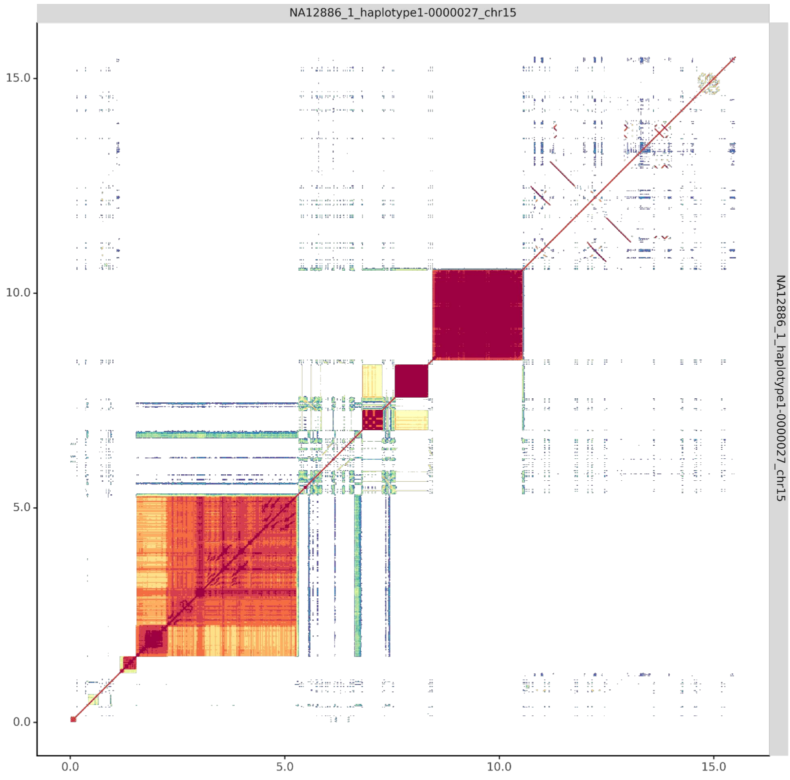

results/chr15_1_22694466/moddotplot/NA12886_2/NA12886_2_haplotype2-0000095_chr1

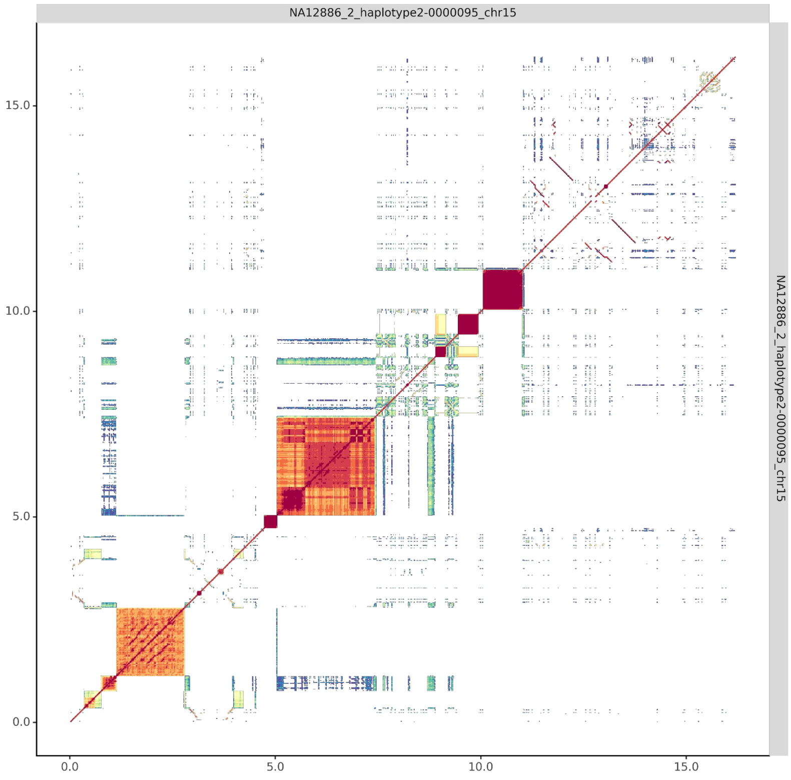

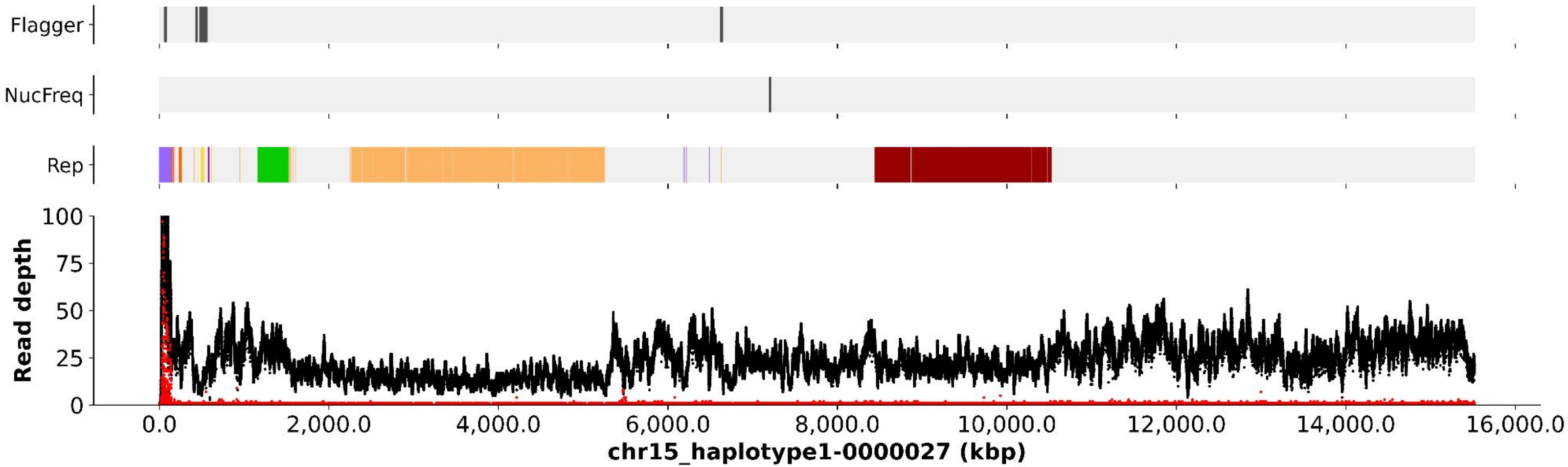

Flagger
NucFreq
Rep
Read depth
100
75
50
25
0
0.0
2,000.0
4,000.0
6,000.0
8,000.0
10,000.0
12,000.0
14,000.0
16,000.0
chr15_haplotype1-0000027 (kbp)

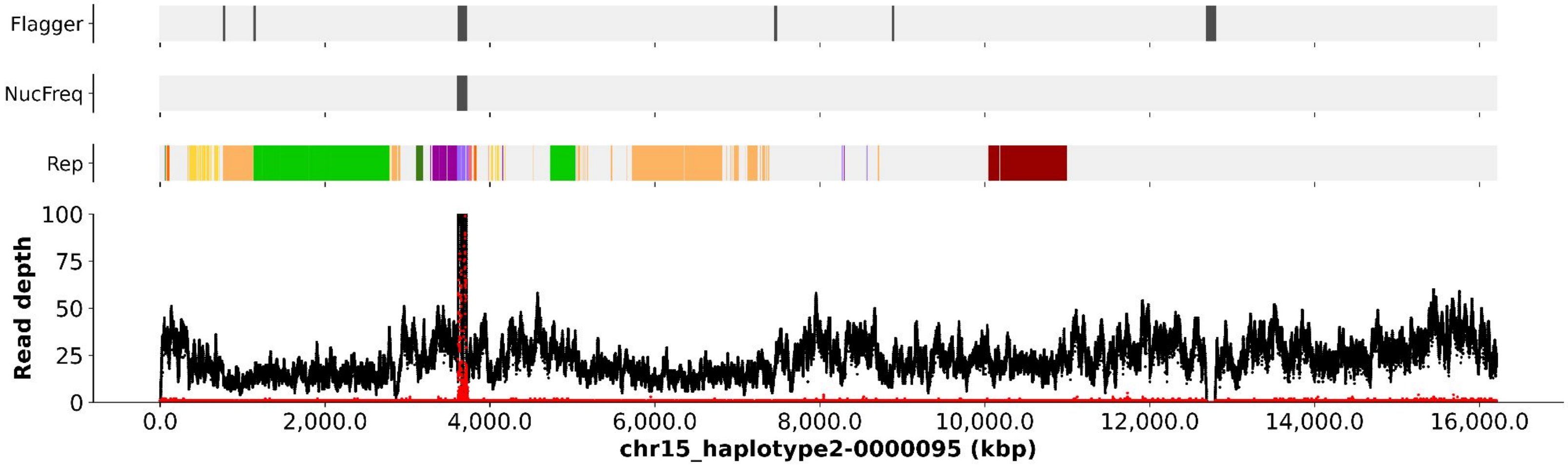

Flagger
NucFreq
Rep
Read depth
100
75
50
25
0
0.0
2,000.0
4,000.0
6,000.0
8,000.0
10,000.0
12,000.0
14,000.0
16,000.0
chr15_haplotype2-0000095 (kbp)

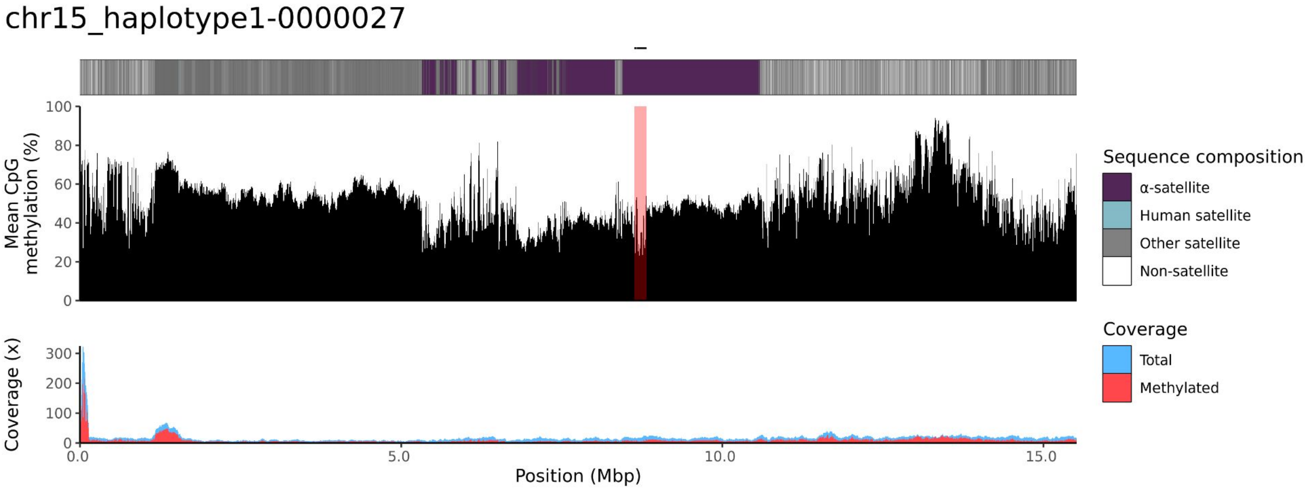
chr15_haplotype1-0000027
Mean CpG methylation (%)
100
80
60
40
20
0
Coverage (x)
300
200
100
0
0.0
5.0
10.0
15.0
Position (Mbp)
Sequence composition
α-satellite
Human satellite
Other satellite
Non-satellite
Coverage
Total
Methylated

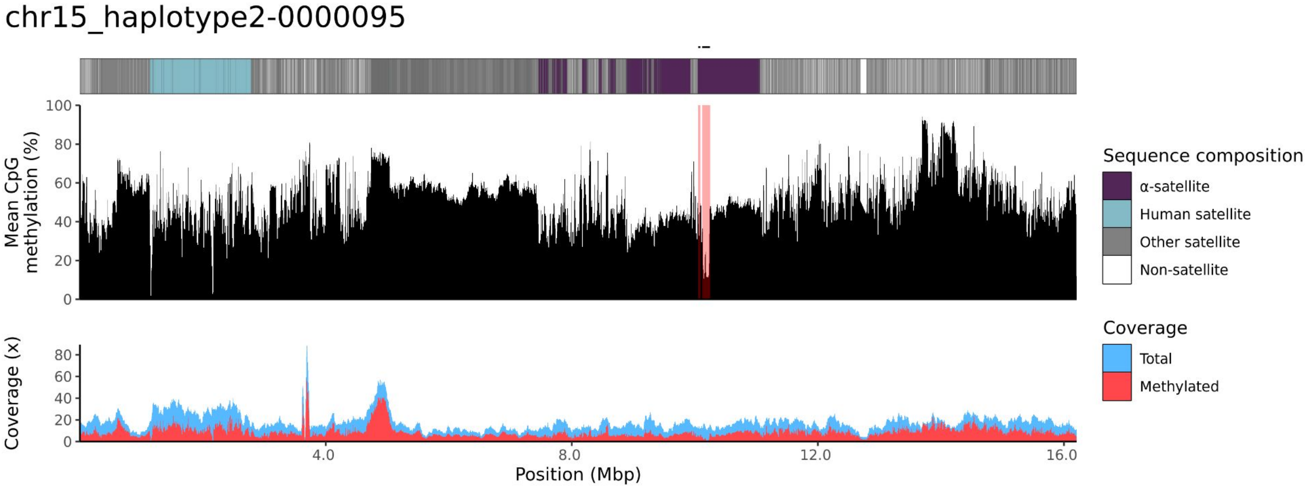
chr15_haplotype2-0000095
Mean CpG methylation (%)
100
80
60
40
20
0
Coverage (x)
80
60
40
20
0
4.0
8.0
12.0
16.0
Position (Mbp)
Sequence composition
α-satellite
Human satellite
Other satellite
Non-satellite
Coverage
Total
Methylated

chr21

NA12886_1_haplotype1-0000005_chr21

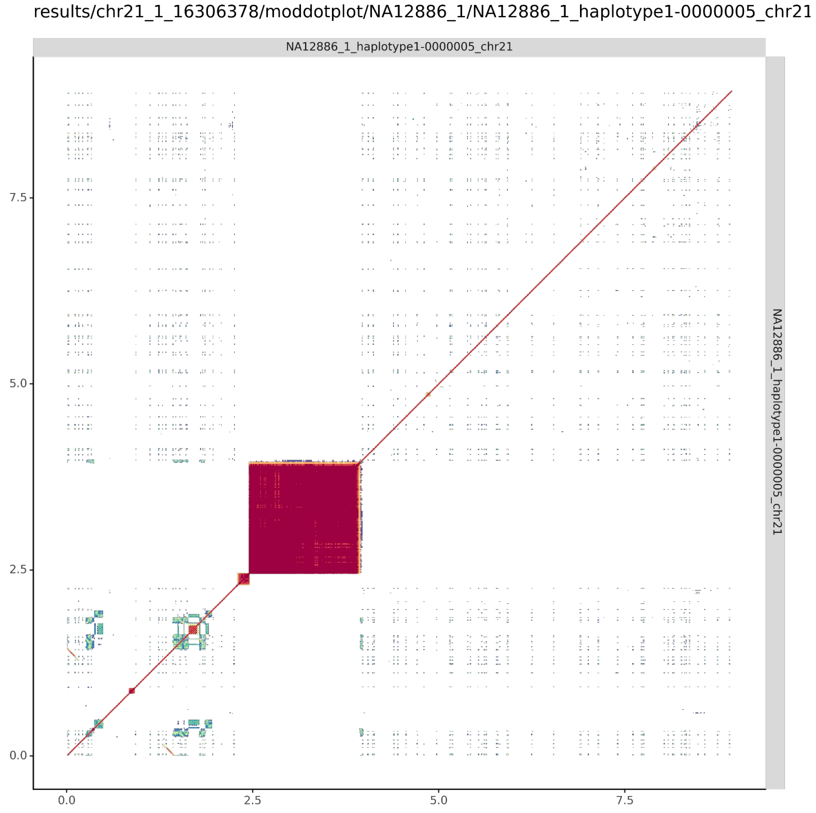

NA12886_2_haplotype2-0000078_chr21

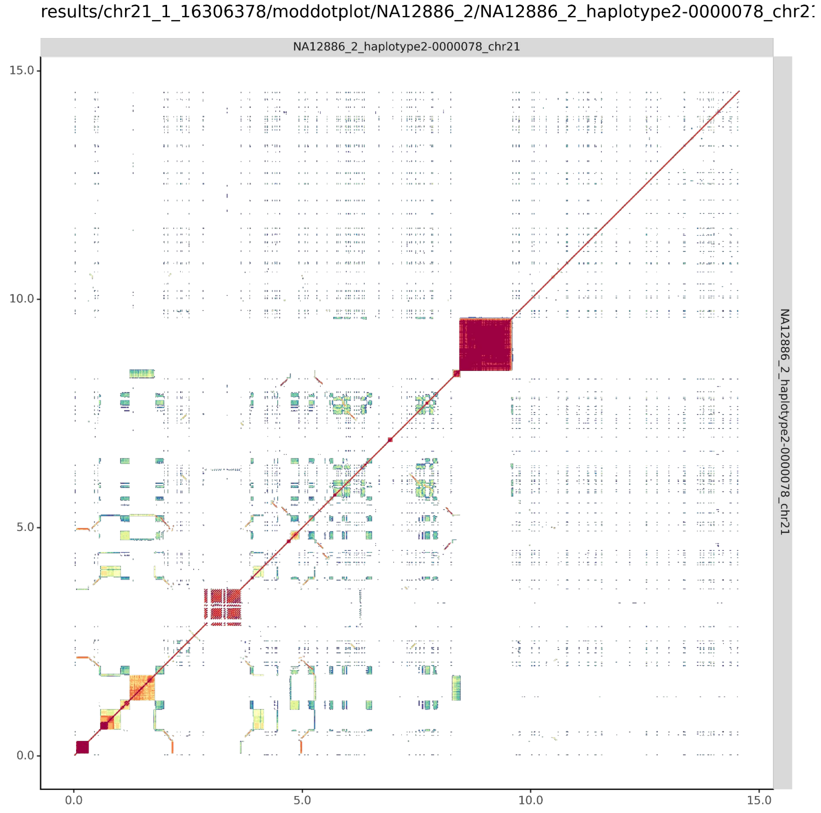

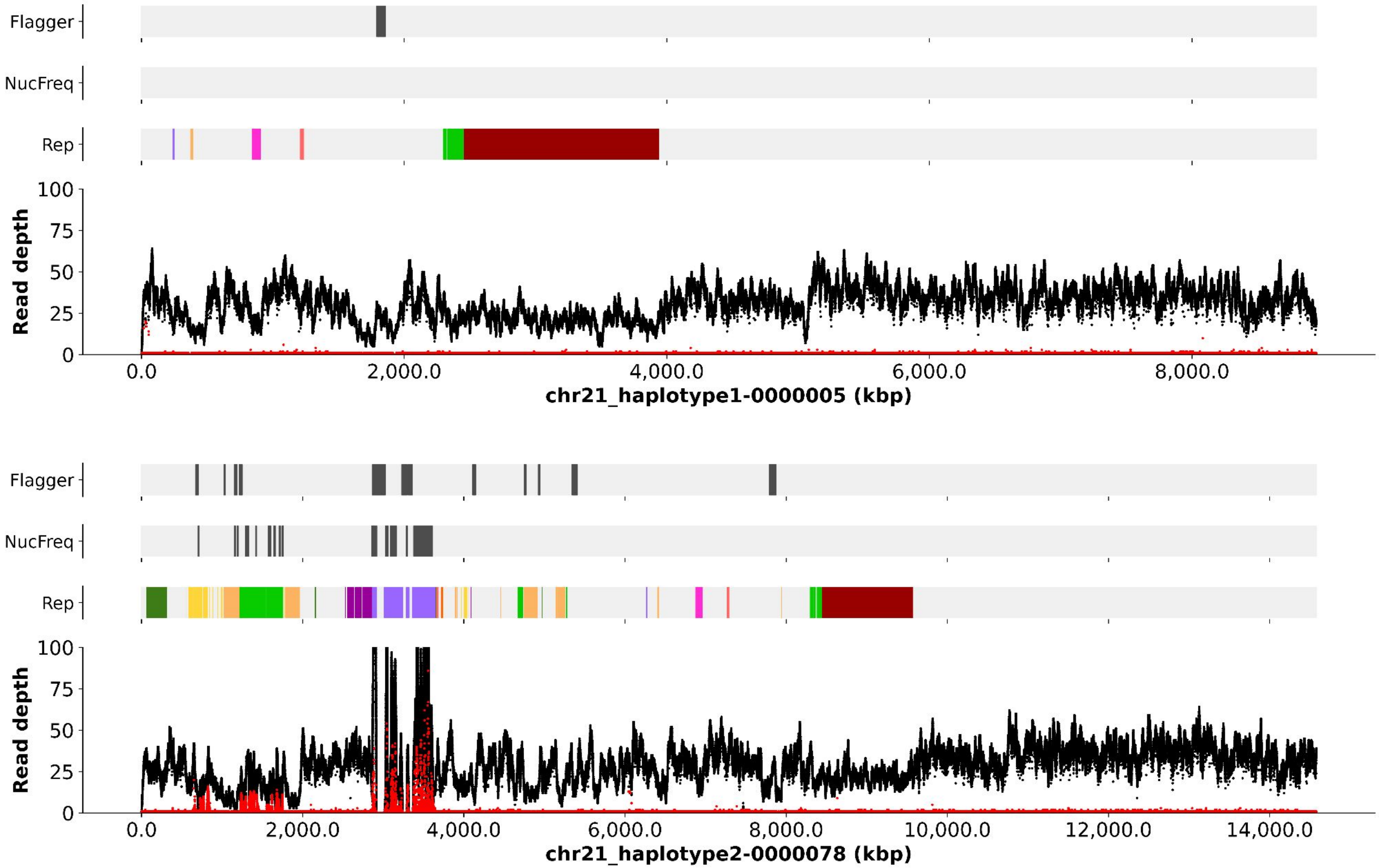
Flagger
NucFreq
Rep
Read depth
100
75
50
25
0
0.0
2,000.0
4,000.0
6,000.0
8,000.0
chr21_haplotype1-0000005 (kbp)
Flagger
NucFreq
Rep
Read depth
100
75
50
25
0
0.0
2,000.0
4,000.0
6,000.0
8,000.0
10,000.0
12,000.0
14,000.0
chr21_haplotype2-0000078 (kbp)

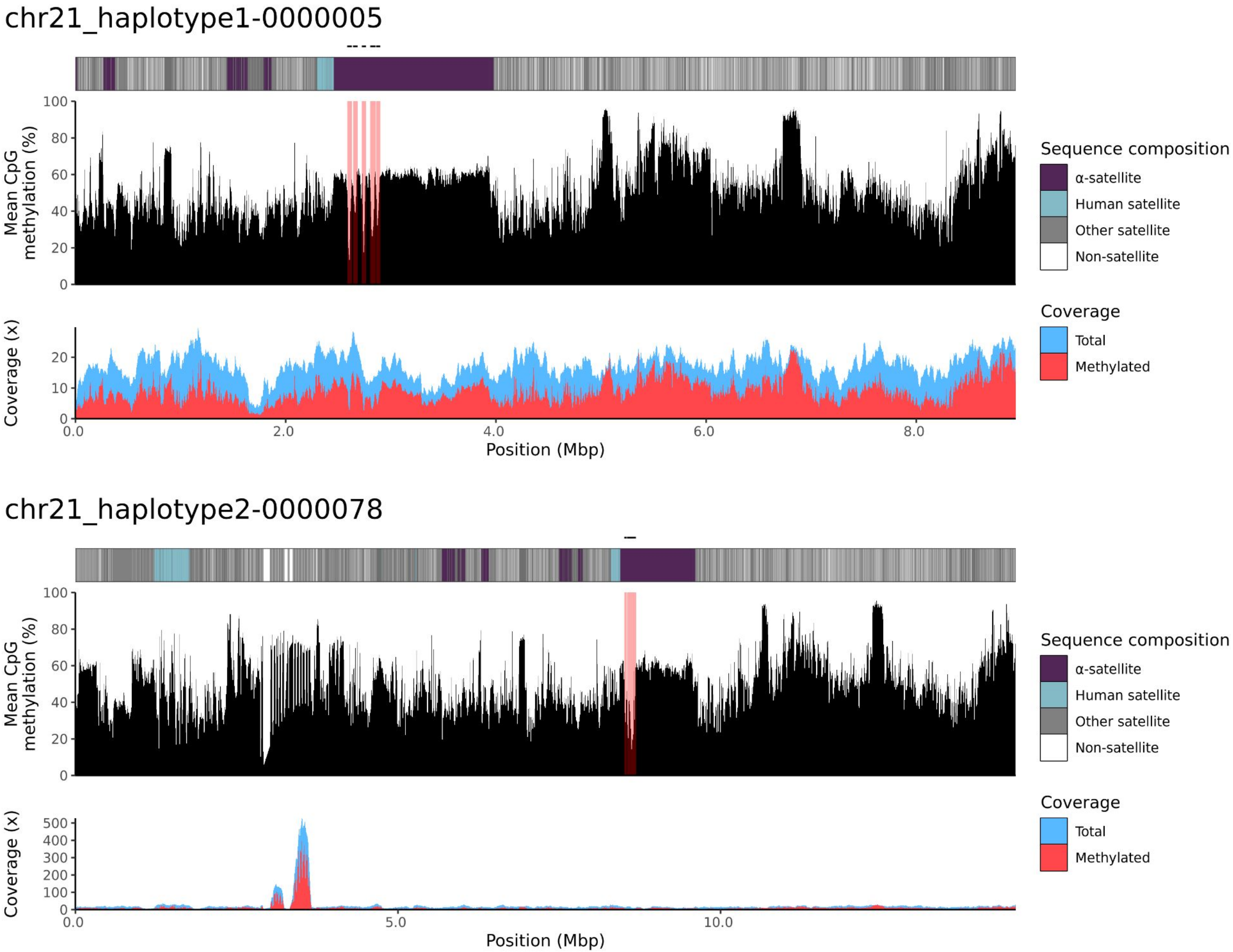
chr21_haplotype1-0000005
Mean CpG methylation (%)
Coverage (x)
Position (Mbp)
Sequence composition
α-satellite
Human satellite
Other satellite
Non-satellite
Coverage
Total
Methylated
chr21_haplotype2-0000078
Mean CpG methylation (%)
Coverage (x)
Position (Mbp)
Sequence composition
α-satellite
Human satellite
Other satellite
Non-satellite
Coverage
Total
Methylated

chr22

NA12886_1_haplotype1-0000015_chr22

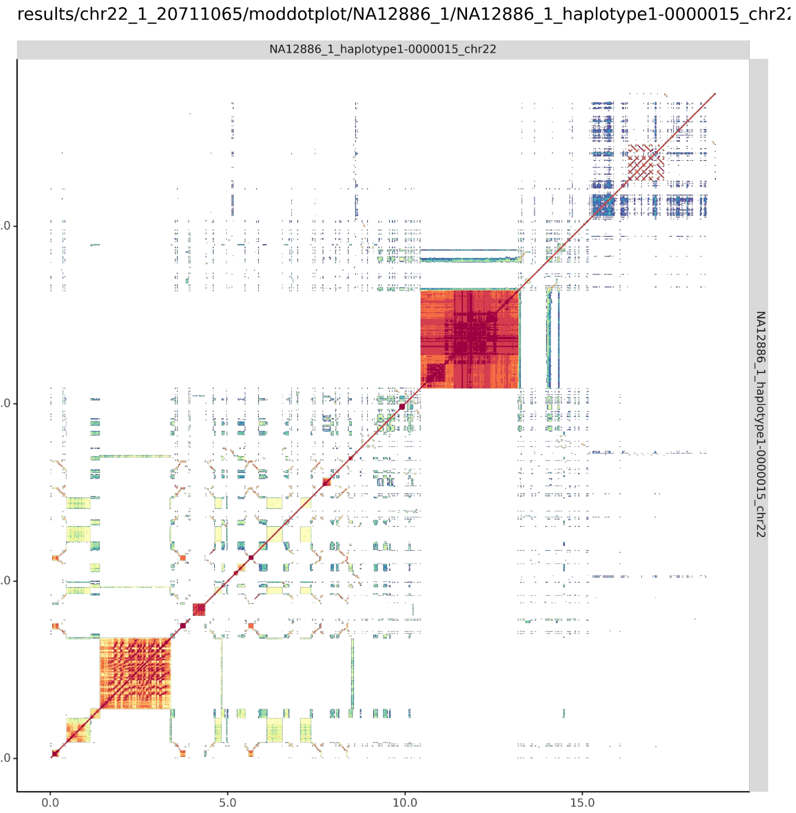

NA12886_2_haplotype2-0000091_chr22

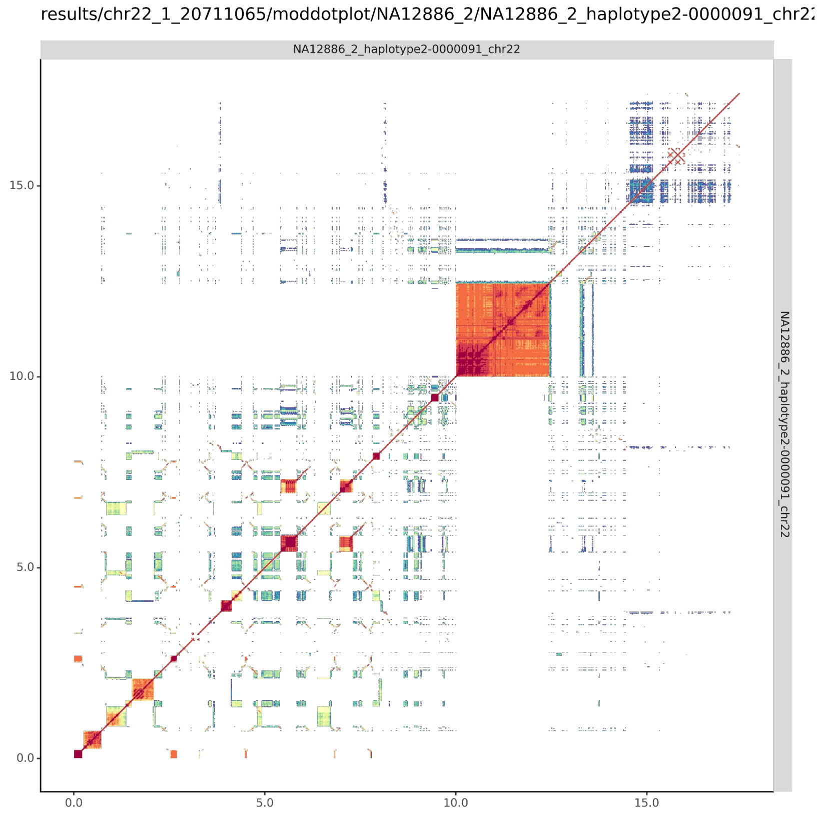

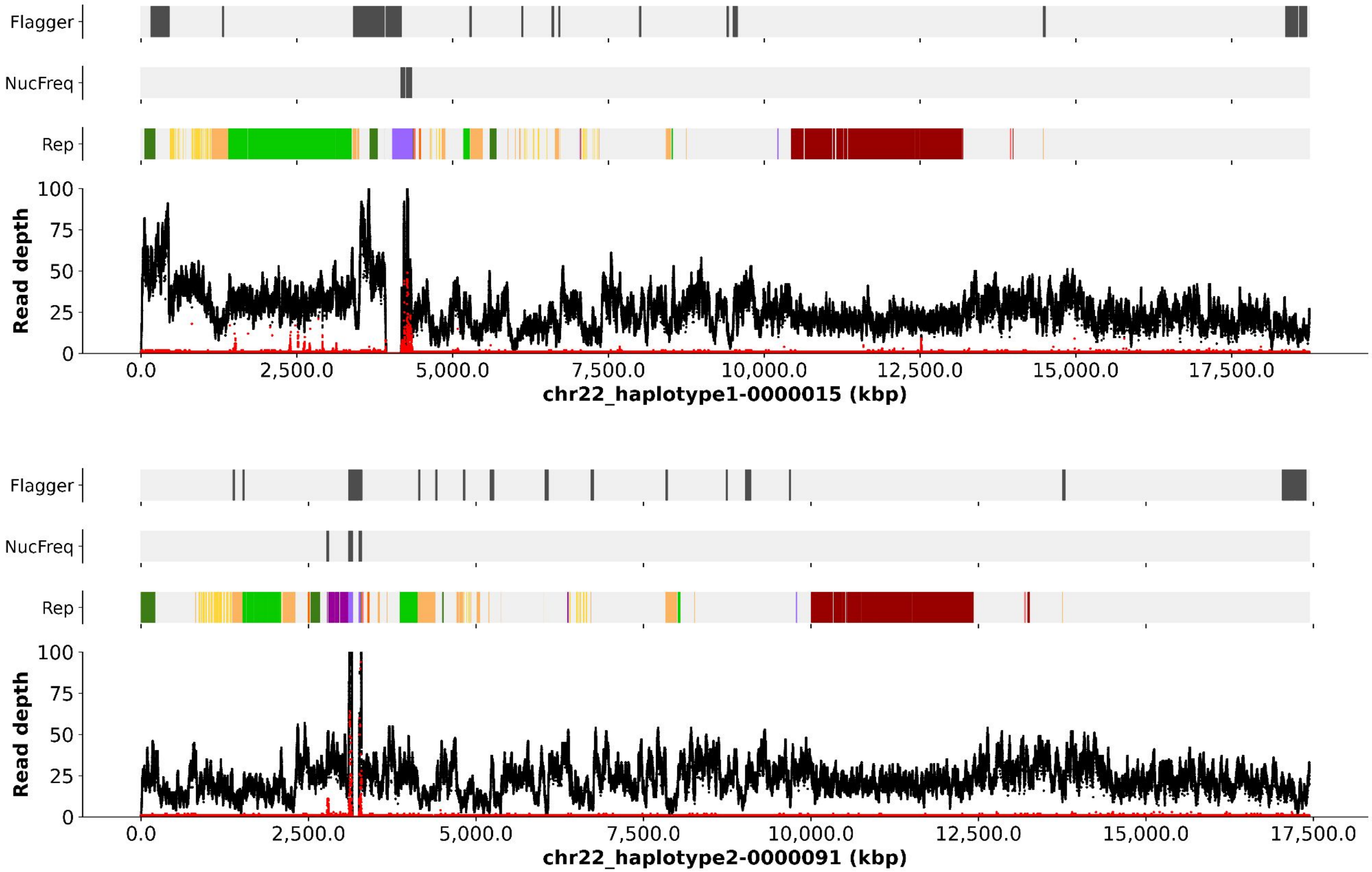

Flagger
NucFreq
Rep
Read depth
100
75
50
25
0
0.0
2,500.0
5,000.0
7,500.0
10,000.0
12,500.0
15,000.0
17,500.0
chr22_haplotype1-0000015 (kbp)
Flagger
NucFreq
Rep
Read depth
chr22_haplotype2-0000091 (kbp)

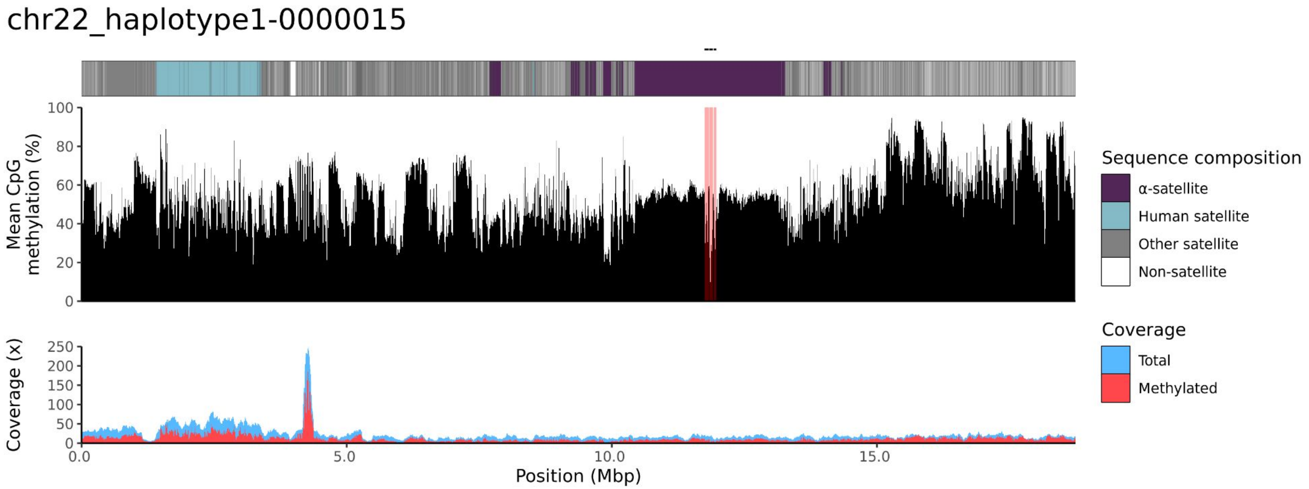
chr22_haplotype1-0000015
Mean CpG methylation (%)
Coverage (x)
Position (Mbp)
Sequence composition
α-satellite
Human satellite
Other satellite
Non-satellite
Coverage
Total
Methylated

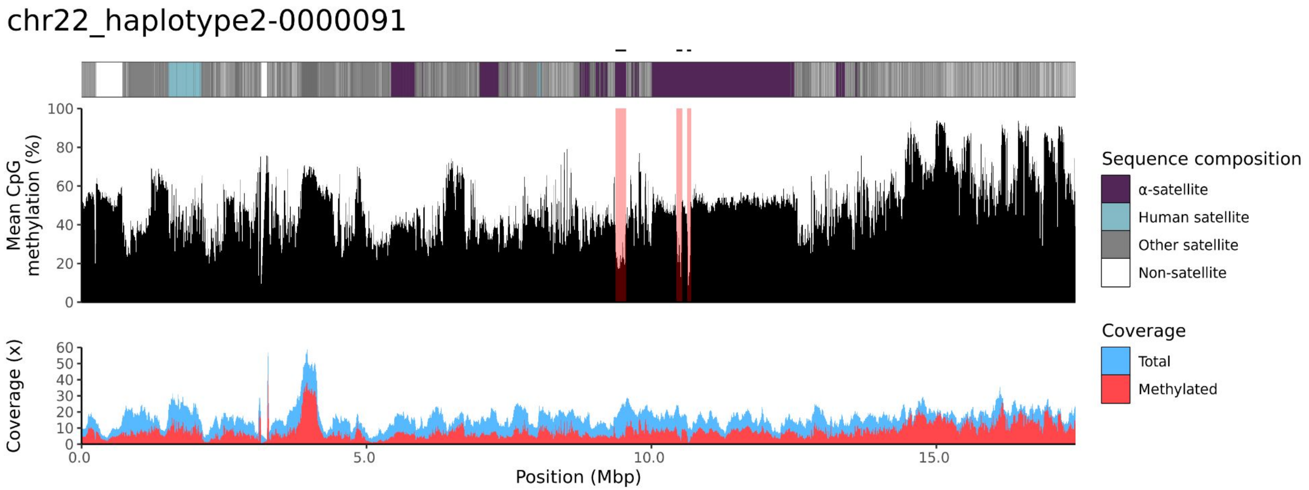
chr22_haplotype2-0000091
Mean CpG methylation (%)
Coverage (x)
Position (Mbp)
Sequence composition
α-satellite
Human satellite
Other satellite
Non-satellite
Coverage
Total
Methylated

NA12887

chr13

NA12887_1_haplotype1-0000013_chr13

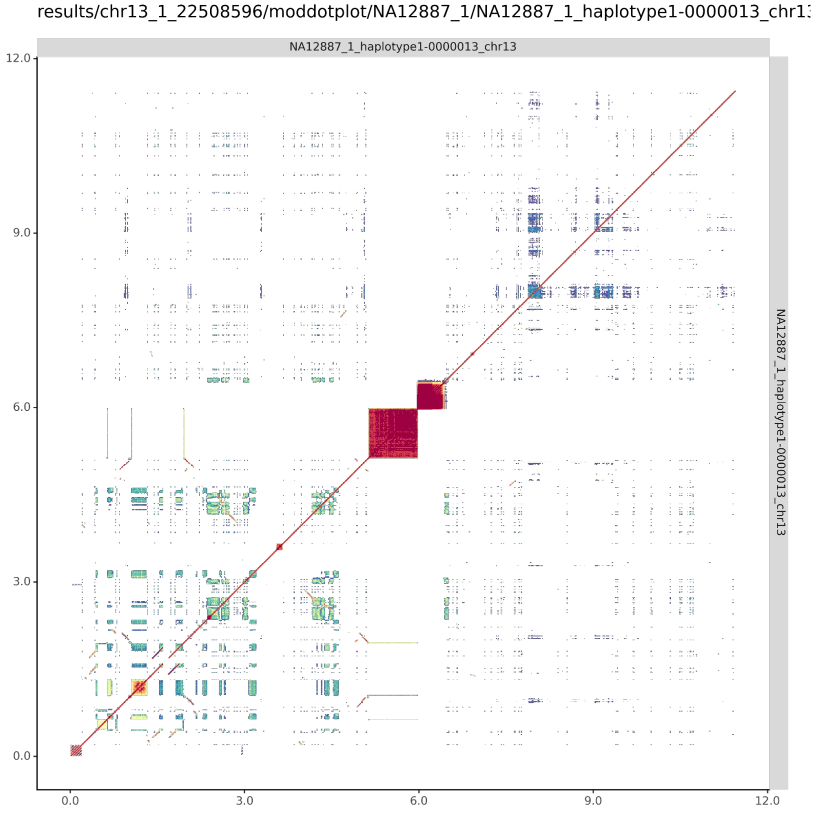

NA12887_2_haplotype2-0000076_chr13

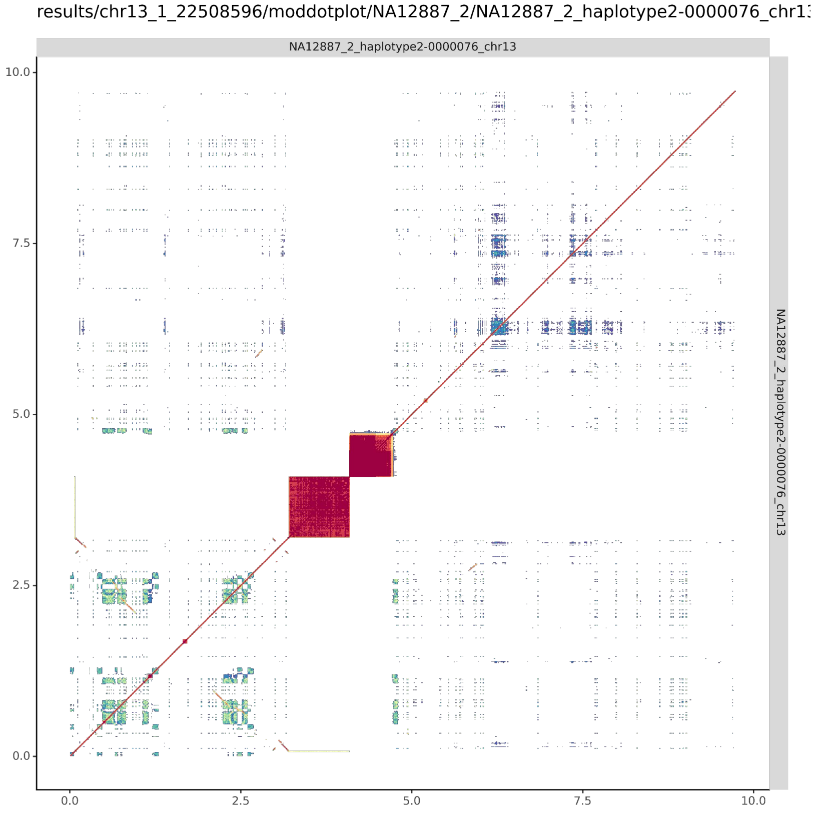

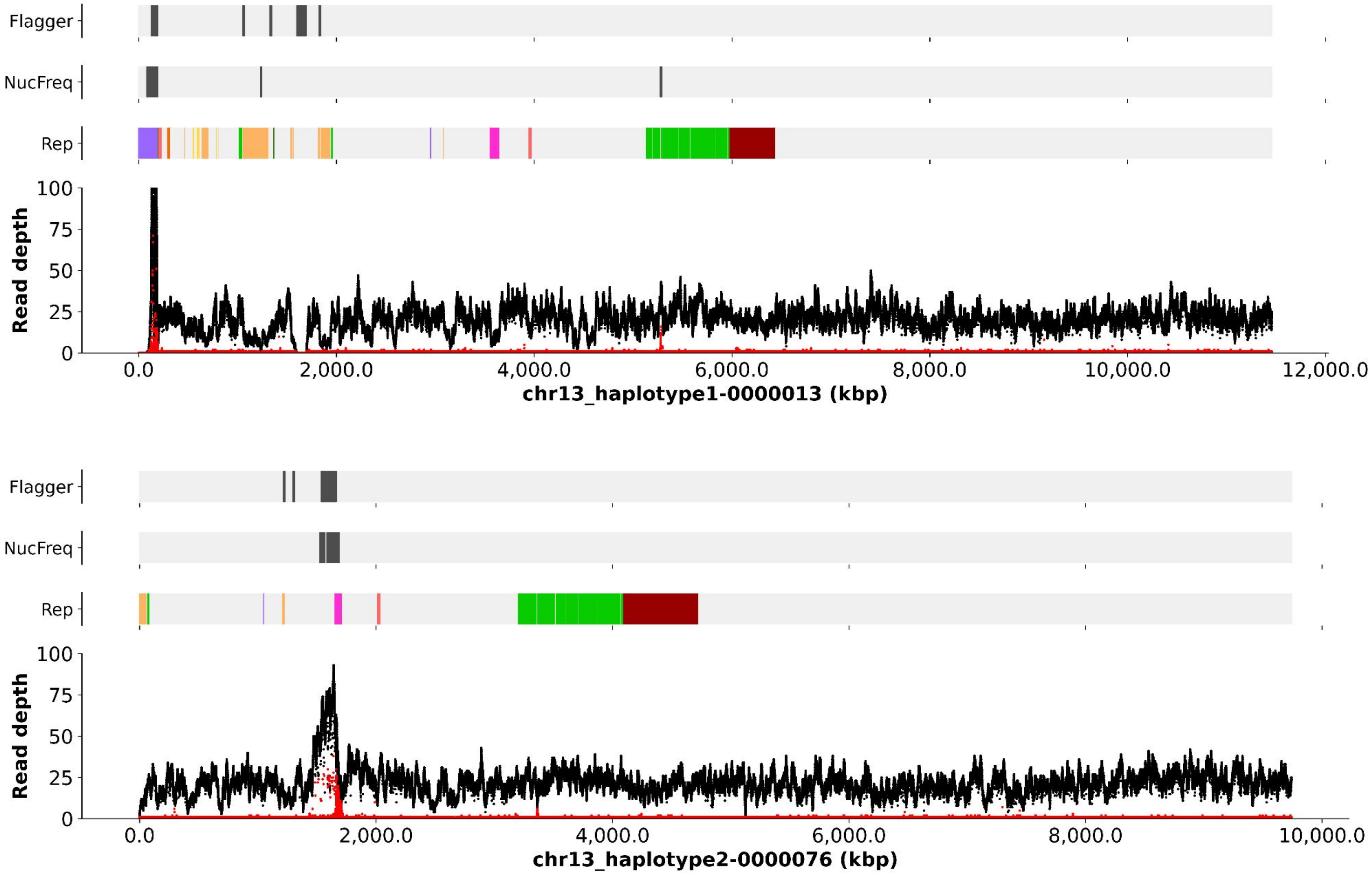

Flagger
NucFreq
Rep
Read depth
100
75
50
25
0
0.0
2,000.0
4,000.0
6,000.0
8,000.0
10,000.0
12,000.0
chr13_haplotype1-0000013 (kbp)
Flagger
NucFreq
Rep
Read depth
100
75
50
25
0
0.0
2,000.0
4,000.0
6,000.0
8,000.0
10,000.0
chr13_haplotype2-0000076 (kbp)

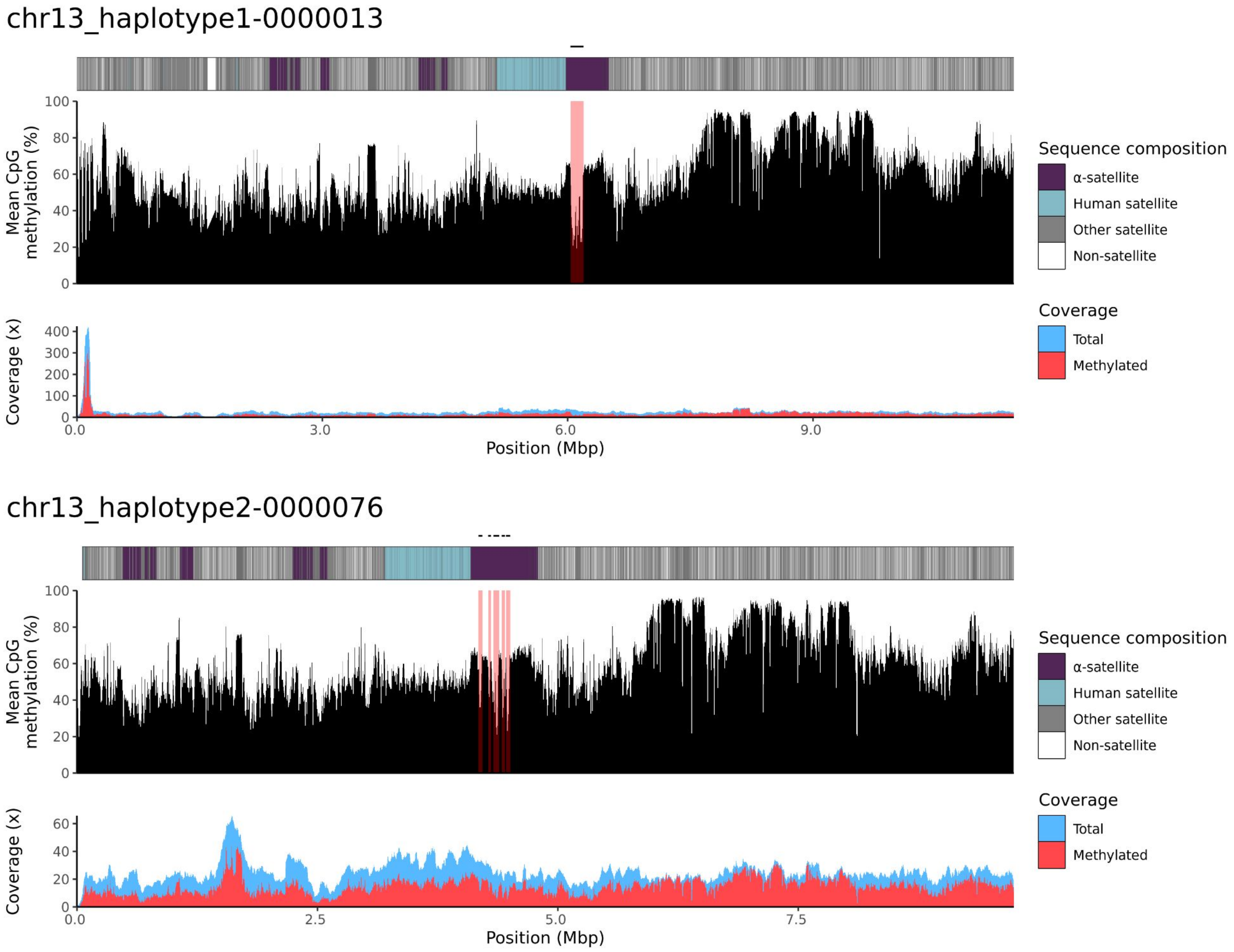
chr13_haplotype1-0000013
Mean CpG methylation (%)
0
20
40
60
80
100
Coverage (x)
0
100
200
300
400
0.0
3.0
6.0
9.0
Position (Mbp)
Sequence composition
α-satellite
Human satellite
Other satellite
Non-satellite
Coverage
Total
Methylated
chr13_haplotype2-0000076
Mean CpG methylation (%)
0
20
40
60
80
100
Coverage (x)
0
20
40
60
0.0
2.5
5.0
7.5
Position (Mbp)
Sequence composition
α-satellite
Human satellite
Other satellite
Non-satellite
Coverage
Total
Methylated

chr14

NA12887_1_haplotype1-0000019_chr14

results/chr14_1_17708411/moddotplot/NA12887_1/NA12887_1_haplotype1-0000019_chr14

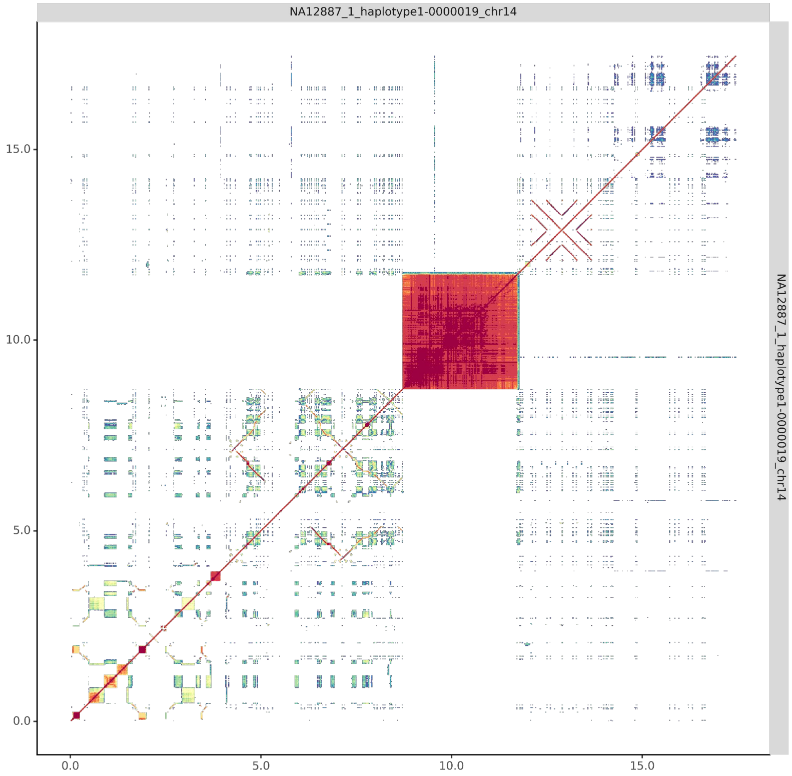

NA12887_2_haplotype2-0000093_chr14

results/chr14_1_17708411/moddotplot/NA12887_2/NA12887_2_haplotype2-0000093_chr14

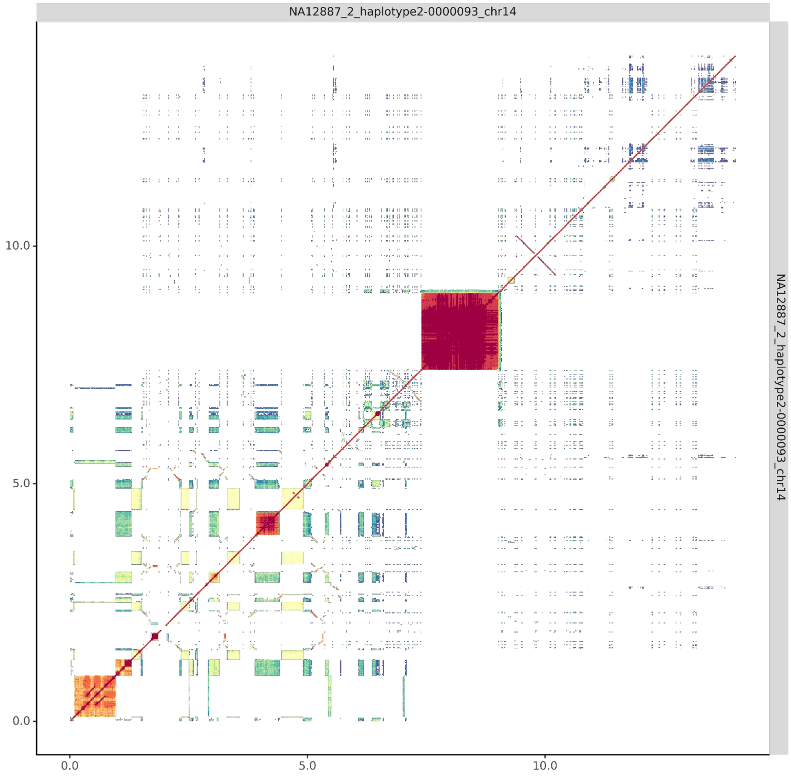

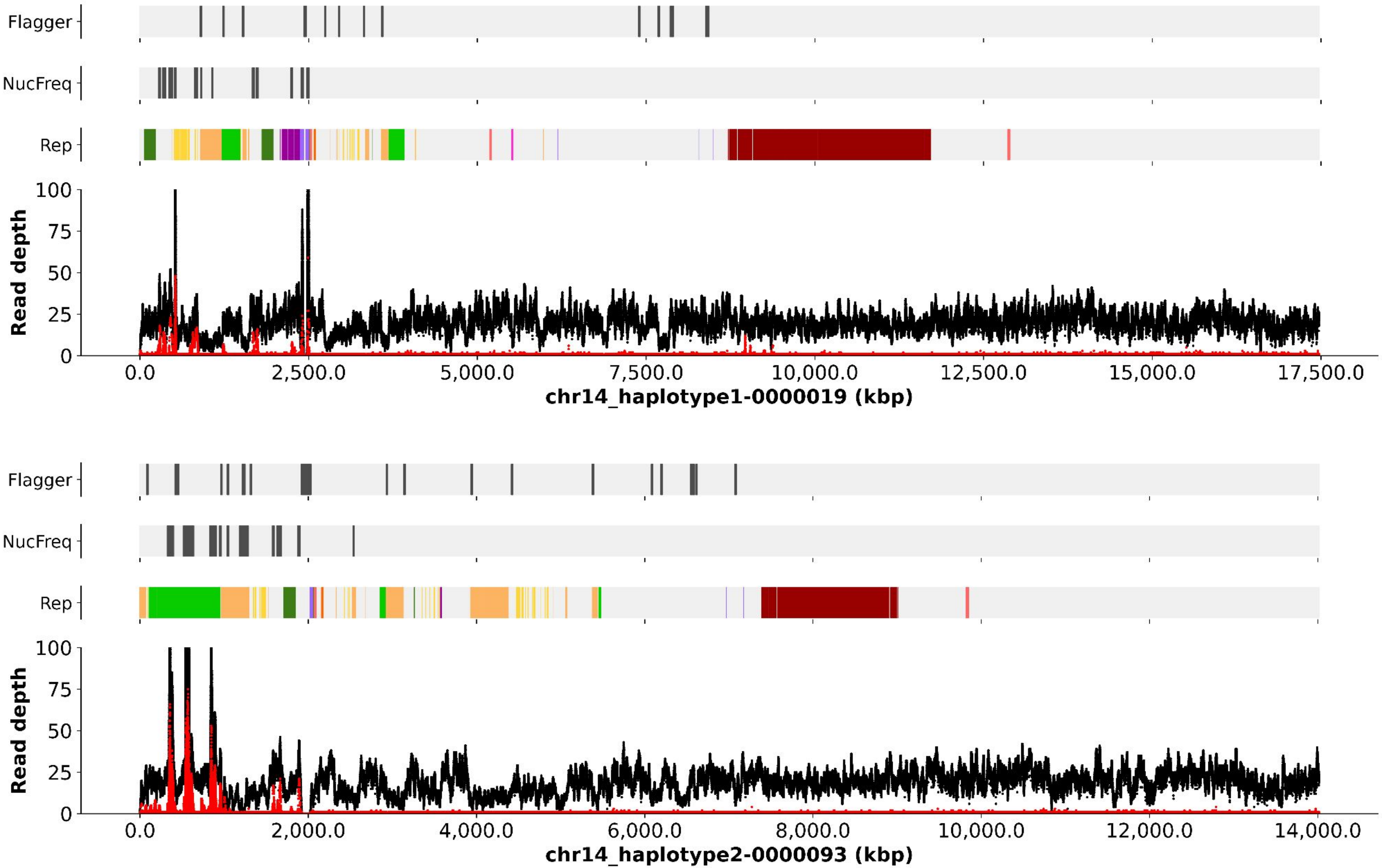

Flagger
NucFreq
Rep
Read depth
100
75
50
25
0
0.0
2,500.0
5,000.0
7,500.0
10,000.0
12,500.0
15,000.0
17,500.0
chr14_haplotype1-0000019 (kbp)
Flagger
NucFreq
Rep
Read depth
100
75
50
25
0
0.0
2,000.0
4,000.0
6,000.0
8,000.0
10,000.0
12,000.0
14,000.0
chr14_haplotype2-0000093 (kbp)

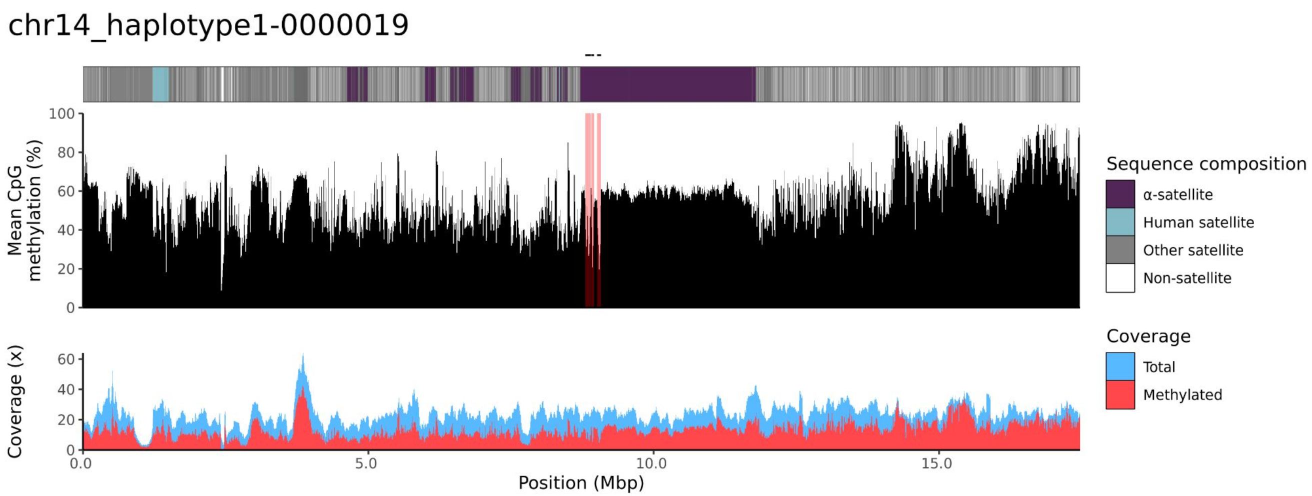
chr14_haplotype1-0000019
Mean CpG methylation (%)
Coverage (x)
Position (Mbp)
Sequence composition
α-satellite
Human satellite
Other satellite
Non-satellite
Coverage
Total
Methylated

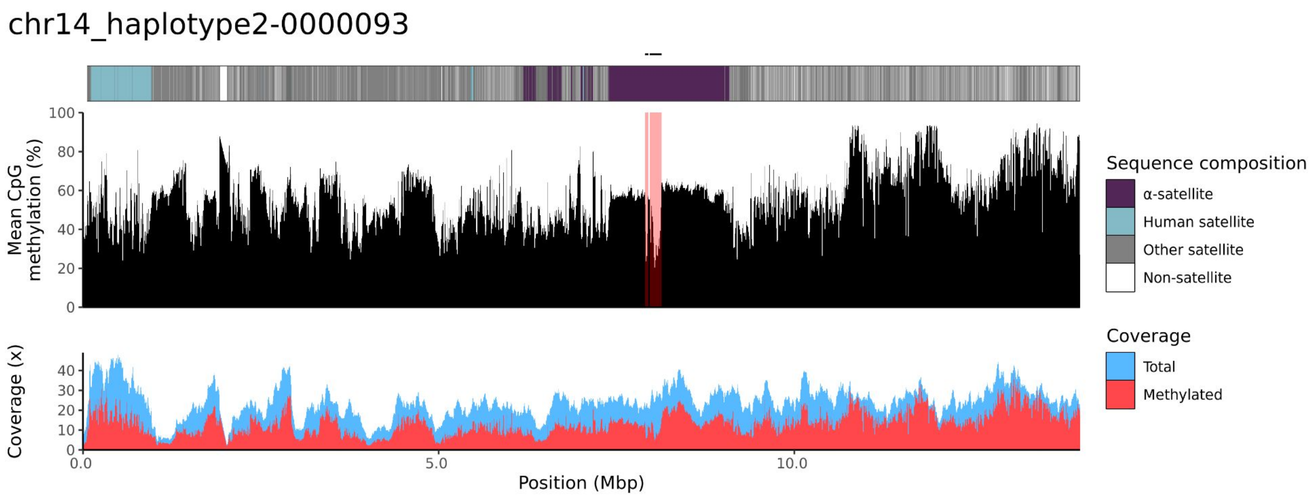
chr14_haplotype2-0000093
Mean CpG methylation (%)
Coverage (x)
Position (Mbp)
Sequence composition
α-satellite
Human satellite
Other satellite
Non-satellite
Coverage
Total
Methylated

chr15

NA12887_1_haplotype1-0000012_chr15

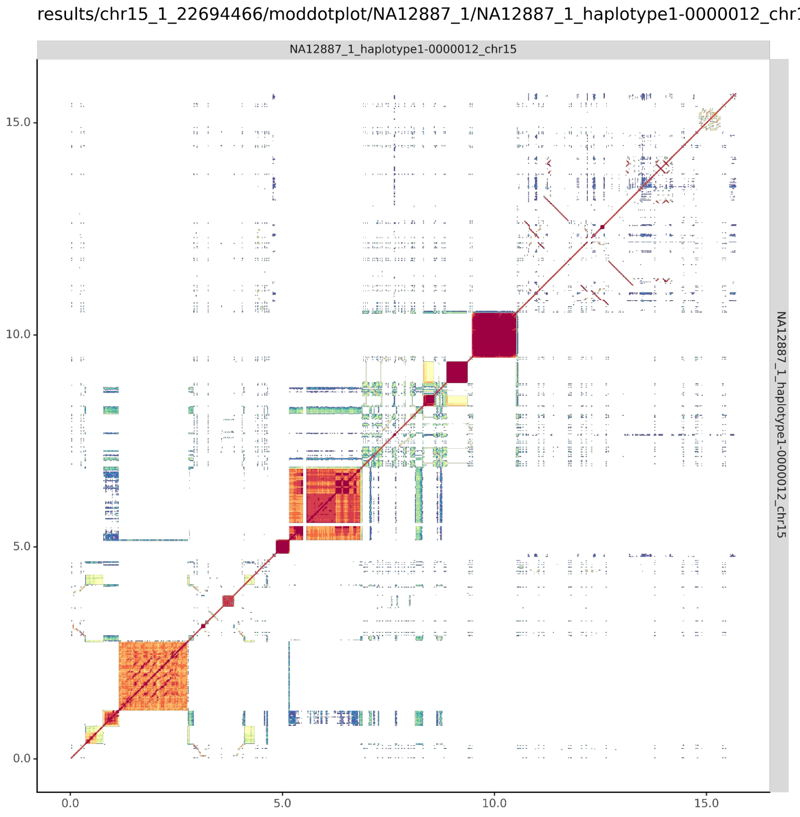

NA12887_2_haplotype2-0000070_chr15

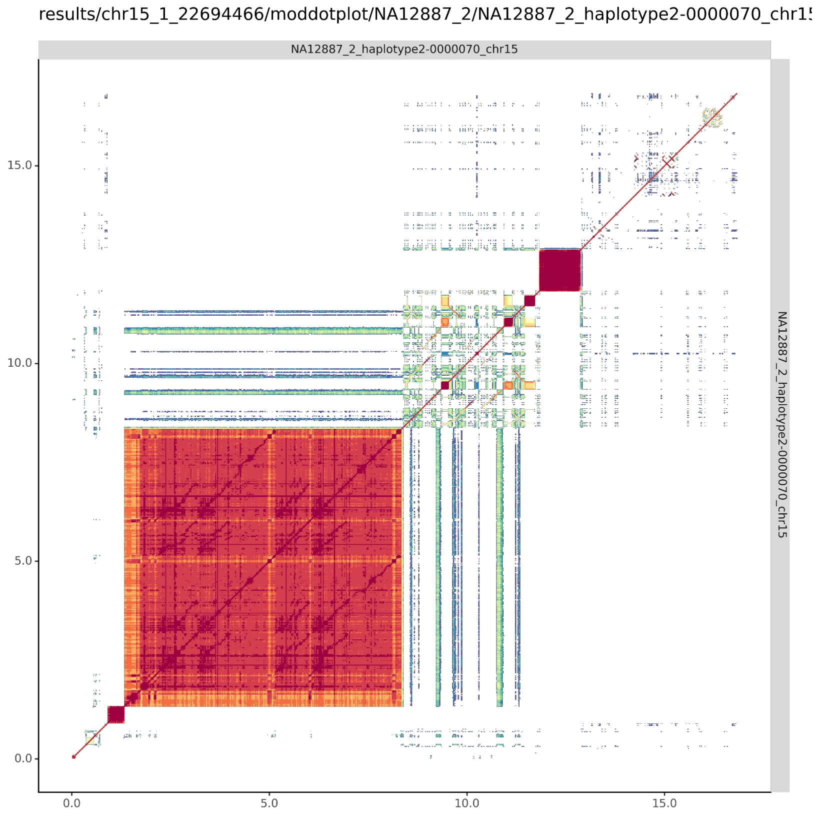

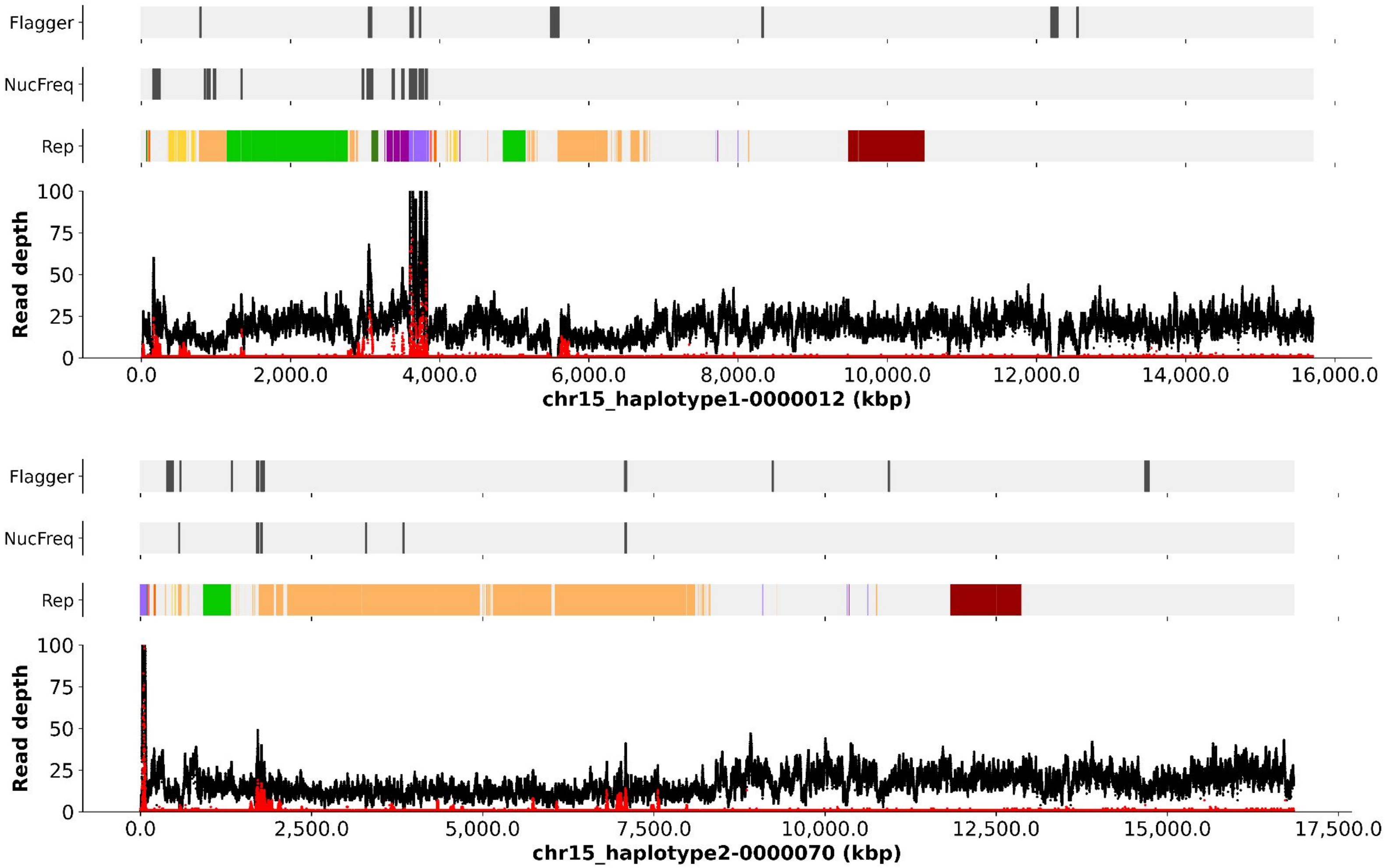

Flagger
NucFreq
Rep
Read depth
0
25
50
75
100
0.0
2,000.0
4,000.0
6,000.0
8,000.0
10,000.0
12,000.0
14,000.0
16,000.0
chr15_haplotype1-0000012 (kbp)
Flagger
NucFreq
Rep
Read depth
0
25
50
75
100
0.0
2,500.0
5,000.0
7,500.0
10,000.0
12,500.0
15,000.0
17,500.0
chr15_haplotype2-0000070 (kbp)

chr15_haplotype1-0000012

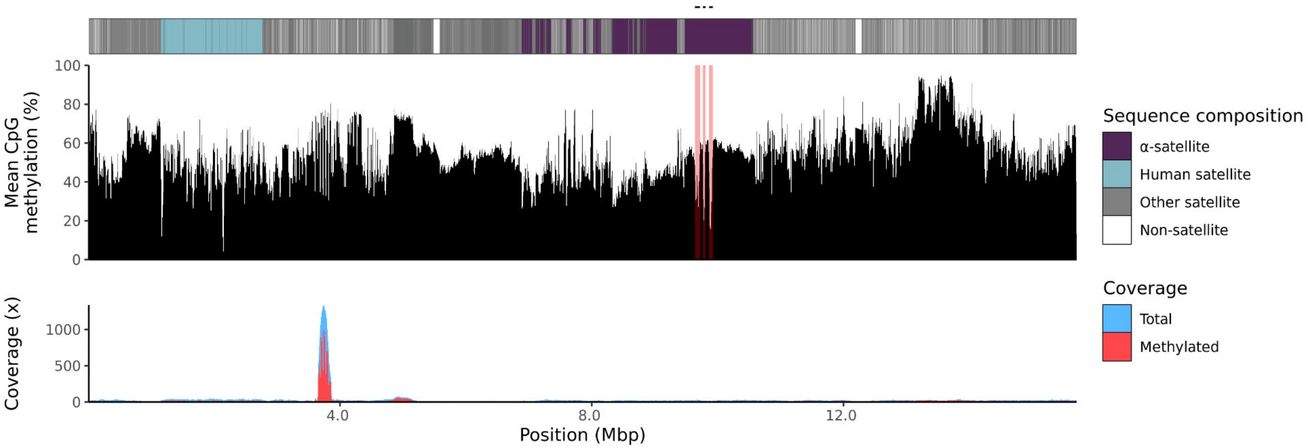

chr15_haplotype2-0000070

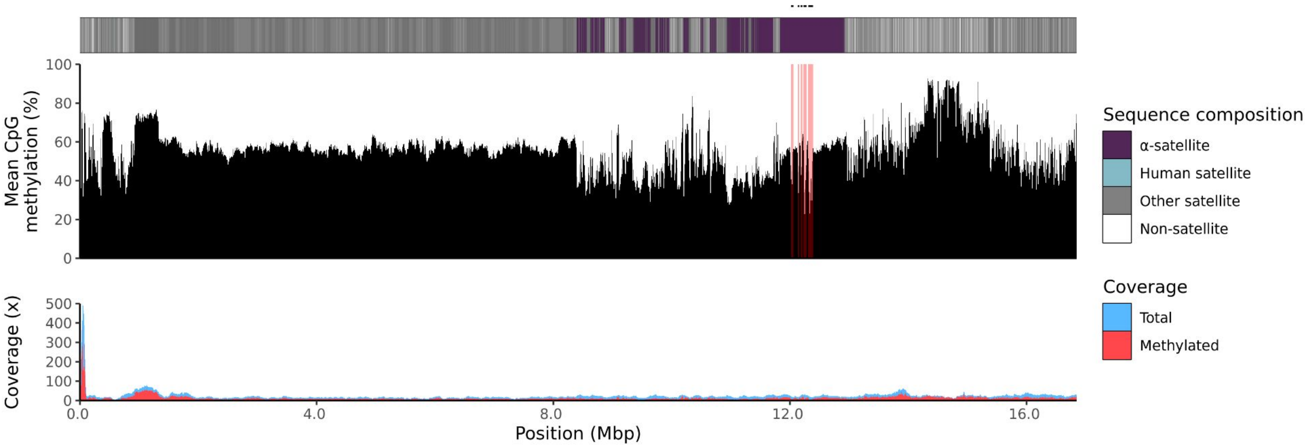

chr21

NA12887_1_haplotype1-0000006_chr21

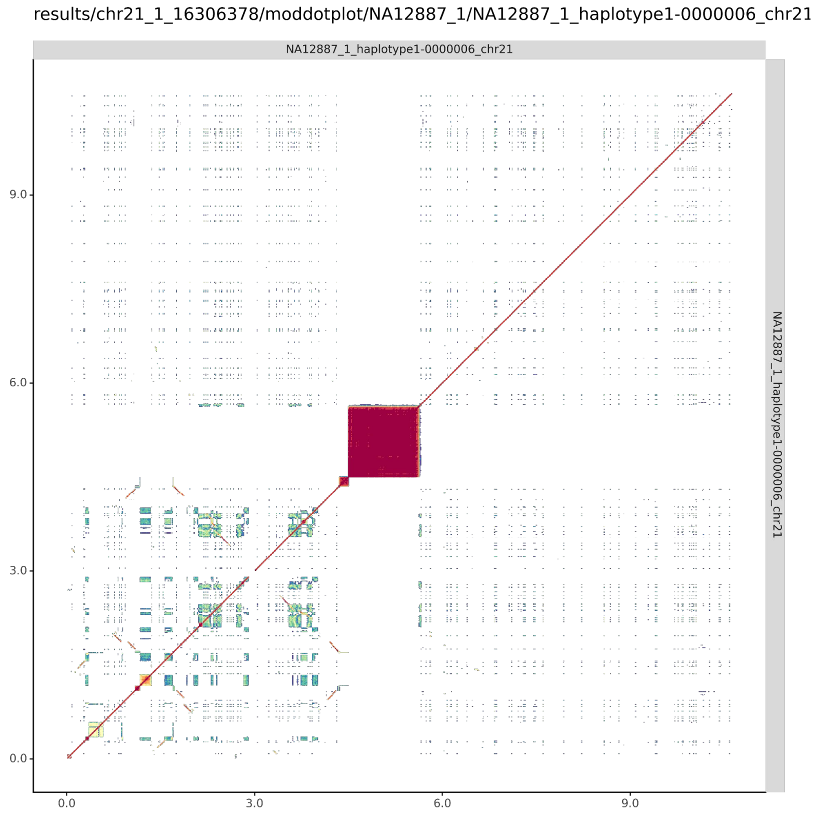

NA12887_2_haplotype2-0000065_chr21

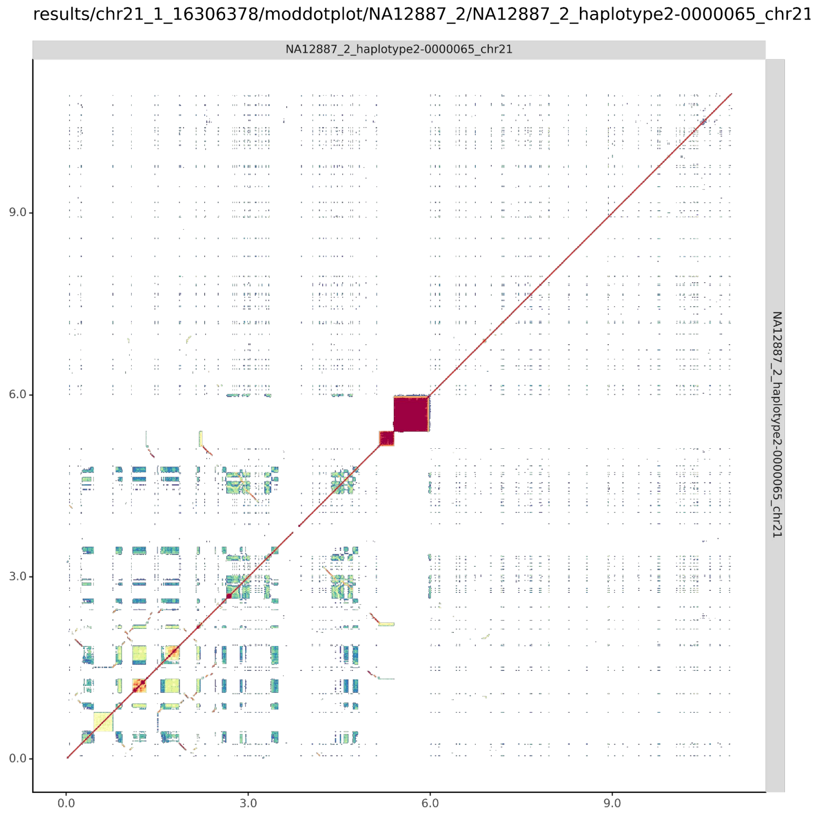

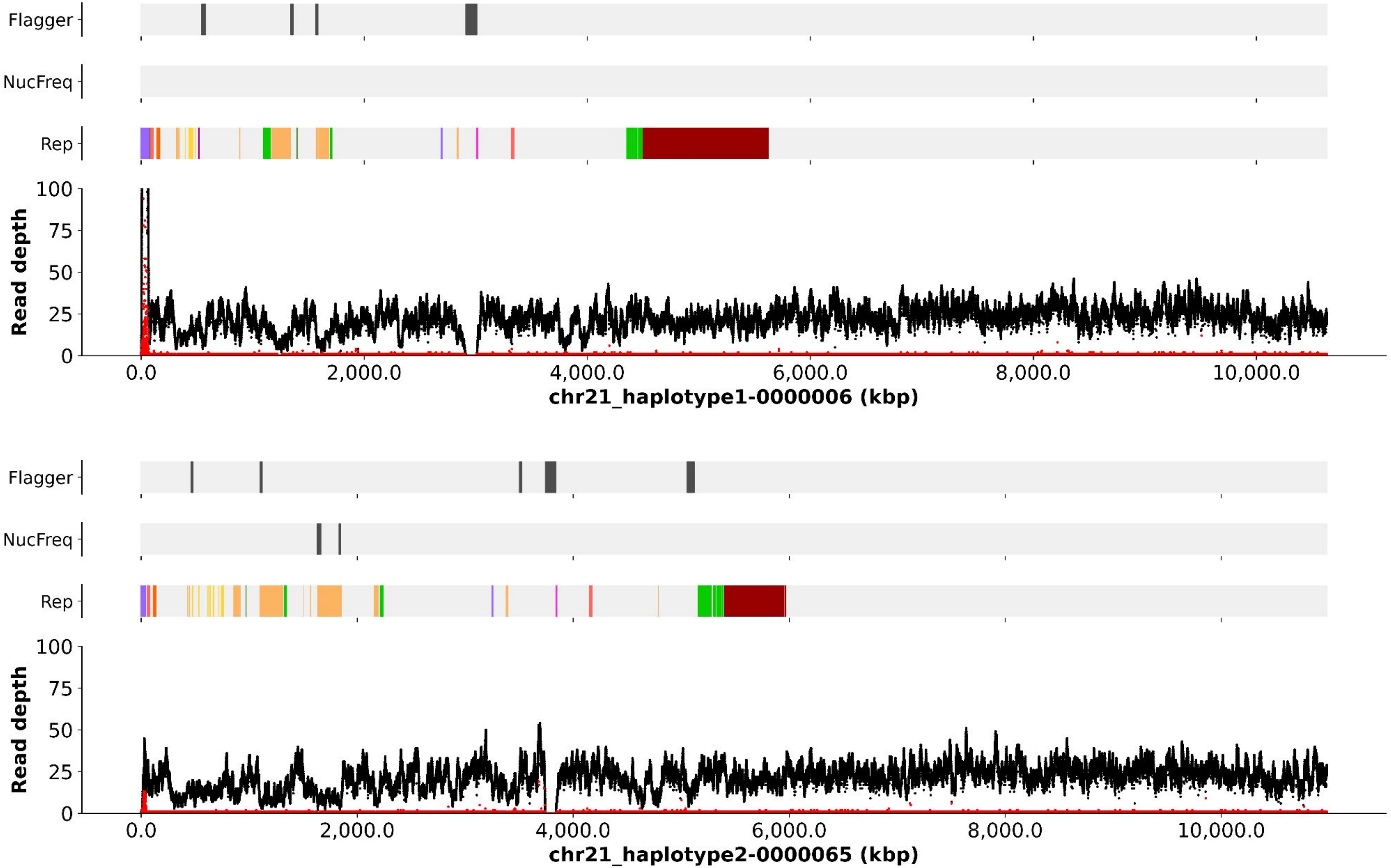
Flagger
NucFreq
Rep
Read depth
100
75
50
25
0
0.0
2,000.0
4,000.0
6,000.0
8,000.0
10,000.0
chr21_haplotype1-0000006 (kbp)
Flagger
NucFreq
Rep
Read depth
100
75
50
25
0
0.0
2,000.0
4,000.0
6,000.0
8,000.0
10,000.0
chr21_haplotype2-0000065 (kbp)

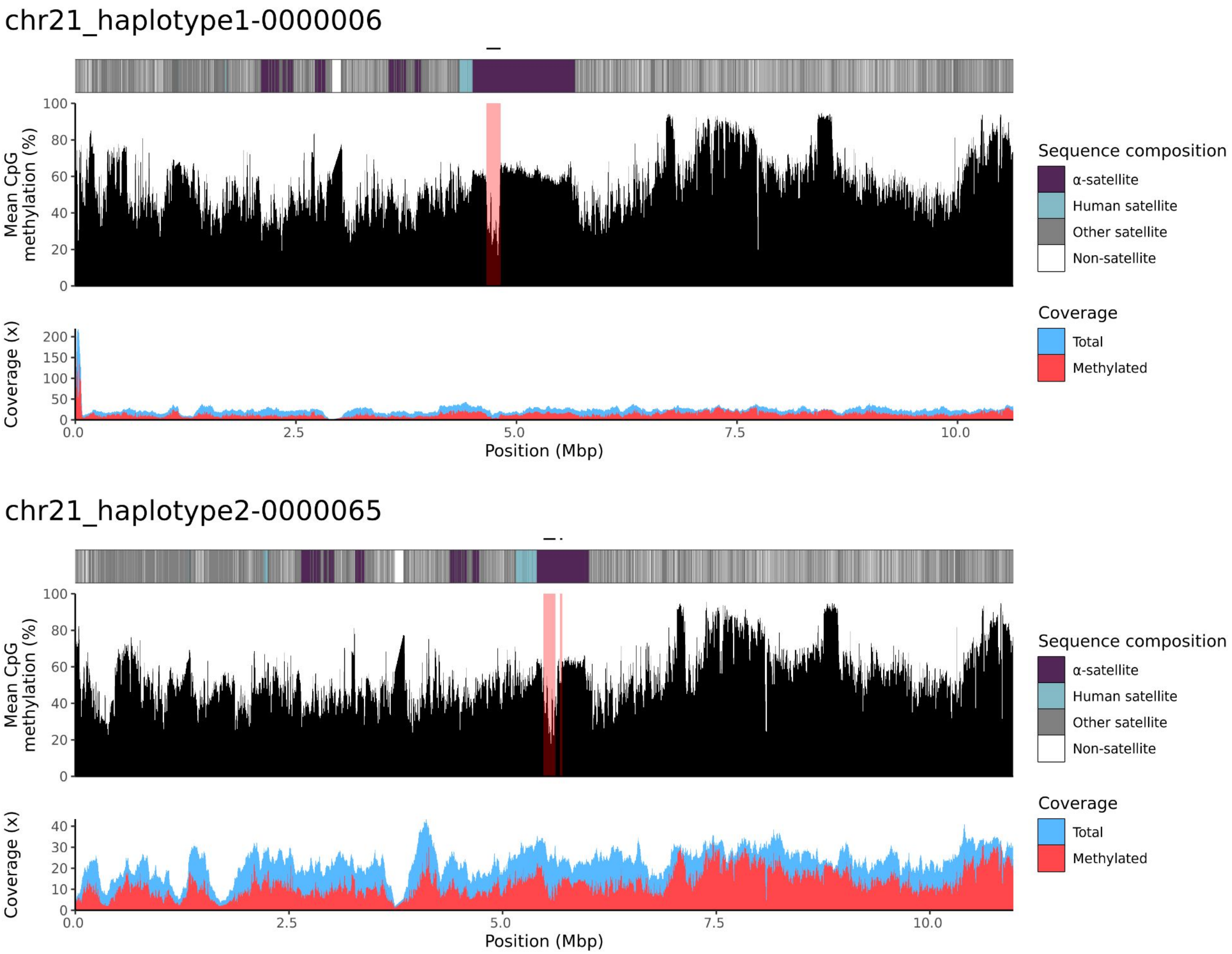
chr21_haplotype1-0000006
Mean CpG methylation (%)
Sequence composition
α-satellite
Human satellite
Other satellite
Non-satellite
Coverage (x)
Coverage
Total
Methylated
Position (Mbp)
chr21_haplotype2-0000065
Mean CpG methylation (%)
Sequence composition
α-satellite
Human satellite
Other satellite
Non-satellite
Coverage (x)
Coverage
Total
Methylated
Position (Mbp)

chr22

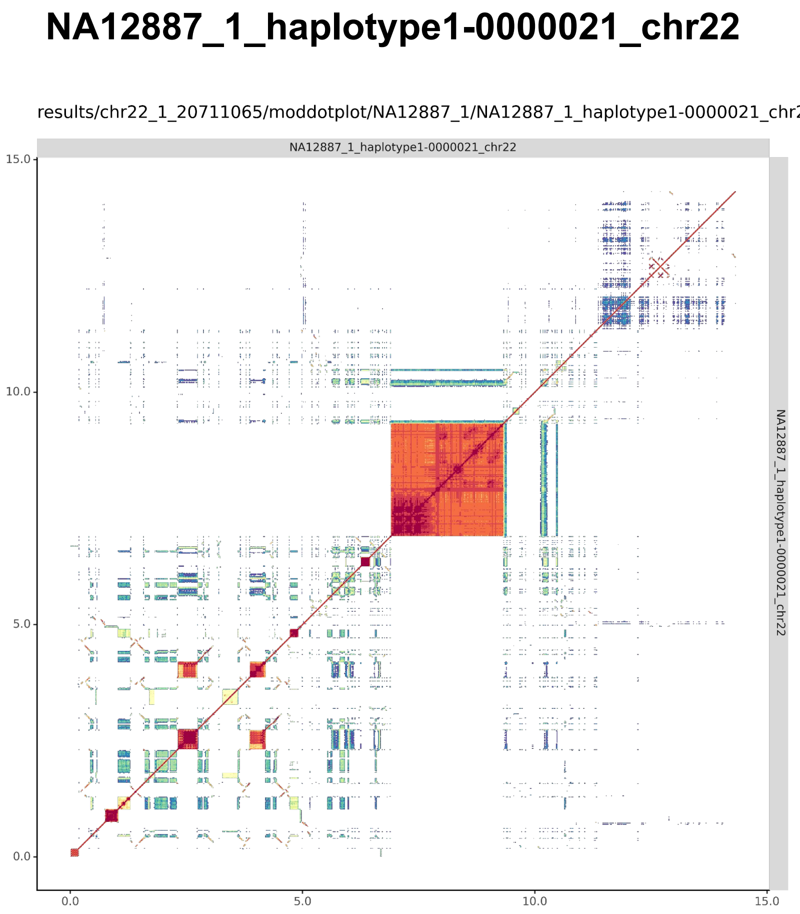
NA12887_1_haplotype1-0000021_chr22
results/chr22_1_20711065/moddotplot/NA12887_1/NA12887_1_haplotype1-0000021_chr2
NA12887_1_haplotype1-0000021_chr22
15.0
10.0
5.0
0.0
0.0
5.0
10.0
15.0
NA12887_1_haplotype1-0000021_chr22

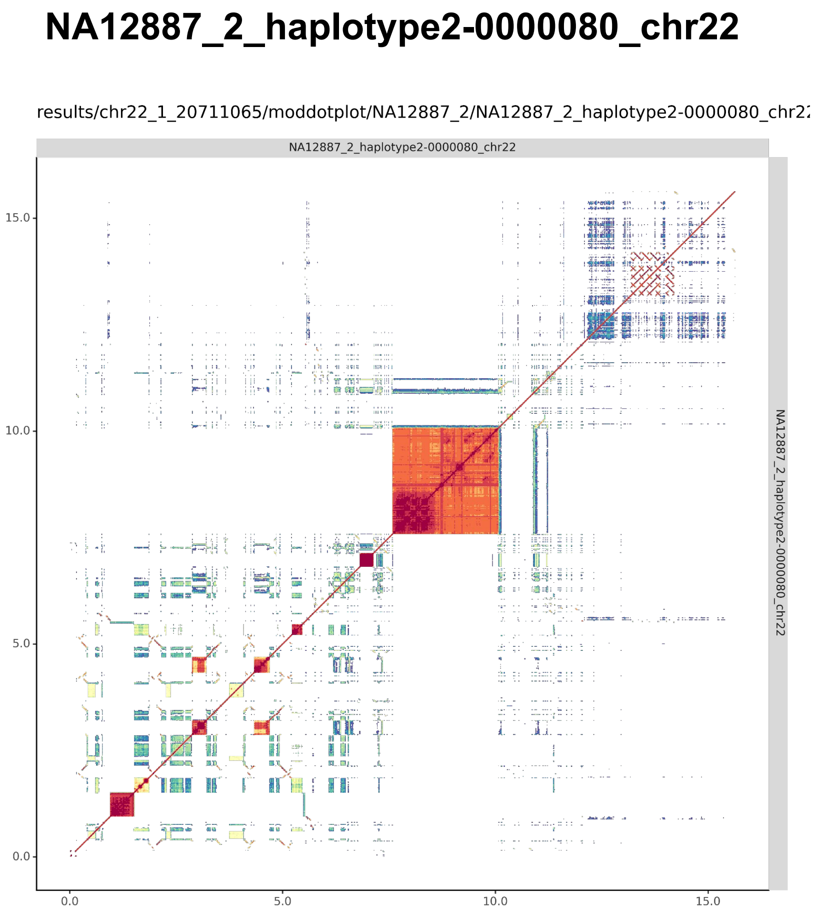
NA12887_2_haplotype2-0000080_chr22
results/chr22_1_20711065/moddotplot/NA12887_2/NA12887_2_haplotype2-0000080_chr2
NA12887_2_haplotype2-0000080_chr22
15.0
10.0
5.0
0.0
0.0
5.0
10.0
15.0
NA12887_2_haplotype2-0000080_chr22

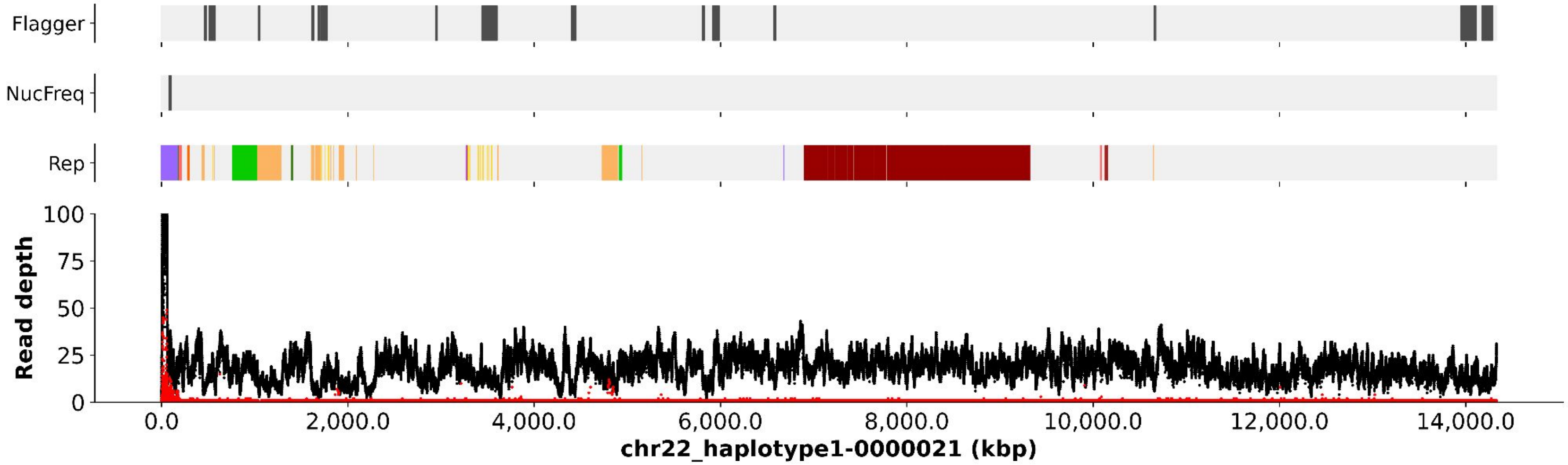

Flagger
NucFreq
Rep
Read depth
100
75
50
25
0
0.0
2,000.0
4,000.0
6,000.0
8,000.0
10,000.0
12,000.0
14,000.0
chr22_haplotype1-0000021 (kbp)

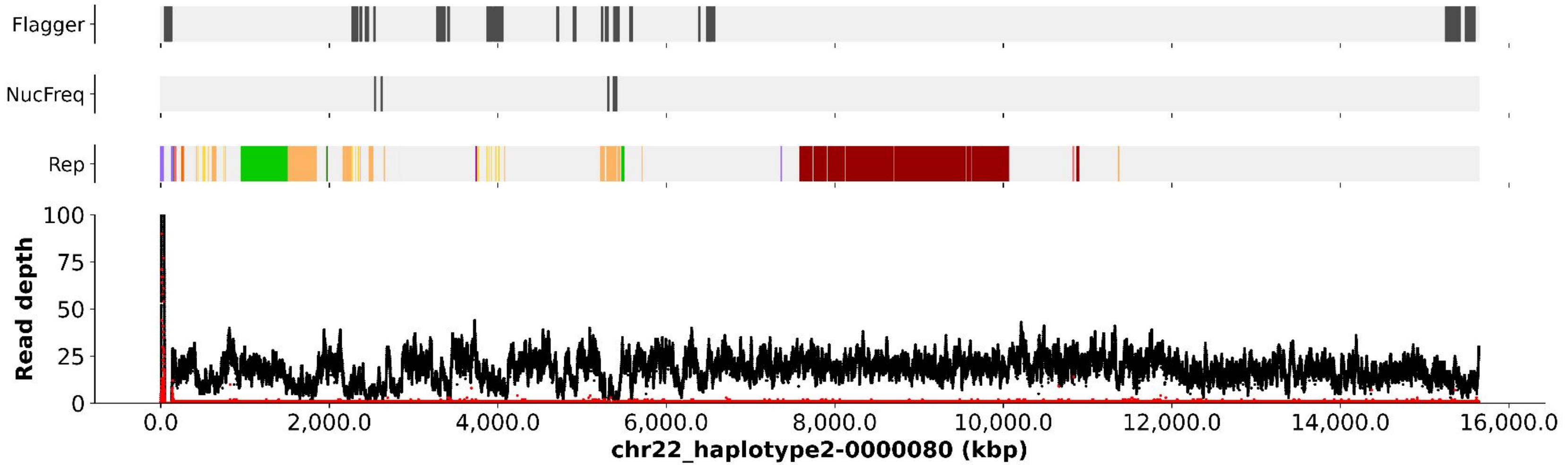

Flagger
NucFreq
Rep
Read depth
100
75
50
25
0
0.0
2,000.0
4,000.0
6,000.0
8,000.0
10,000.0
12,000.0
14,000.0
16,000.0
chr22_haplotype2-0000080 (kbp)

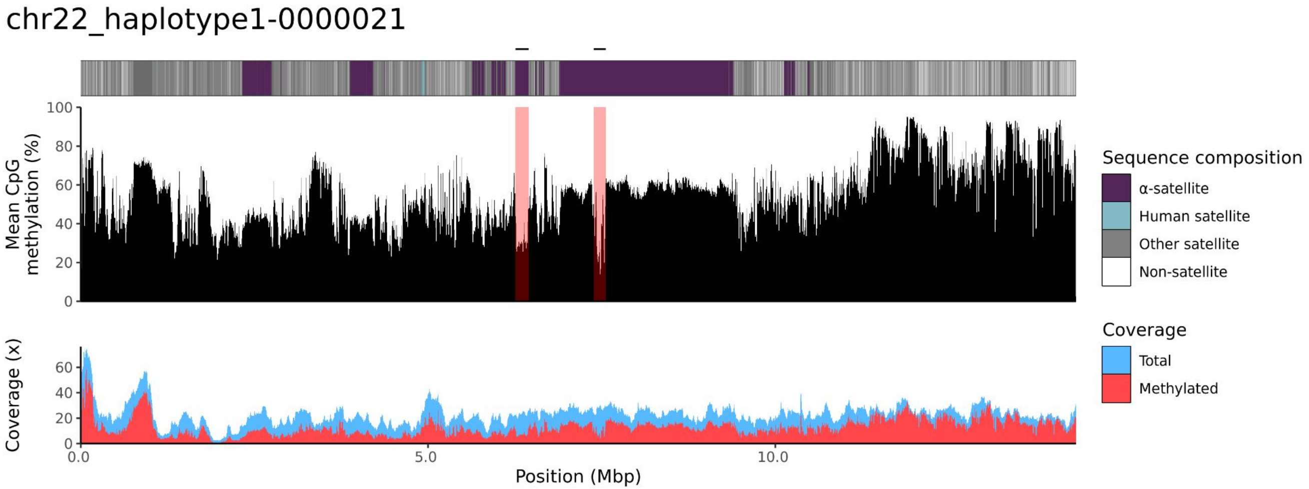

chr22_haplotype1-0000021
Mean CpG methylation (%)
Coverage (x)
Position (Mbp)
Sequence composition
α-satellite
Human satellite
Other satellite
Non-satellite
Coverage
Total
Methylated

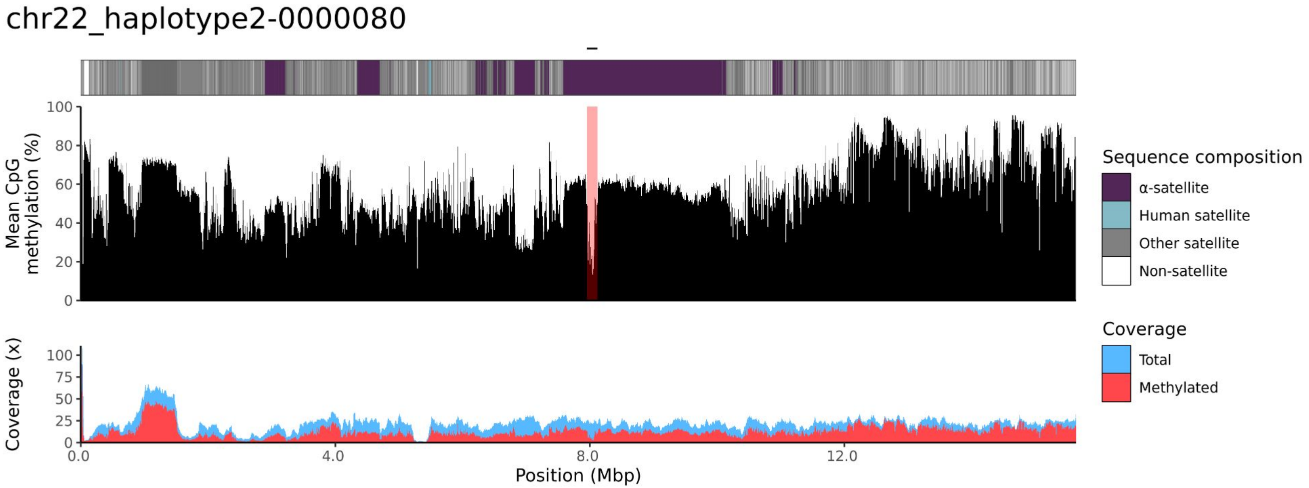

chr22_haplotype2-0000080
Mean CpG methylation (%)
Coverage (x)
Position (Mbp)
Sequence composition
α-satellite
Human satellite
Other satellite
Non-satellite
Coverage
Total
Methylated

200080

chr13

K200080_1_haplotype1-0000024_chr13

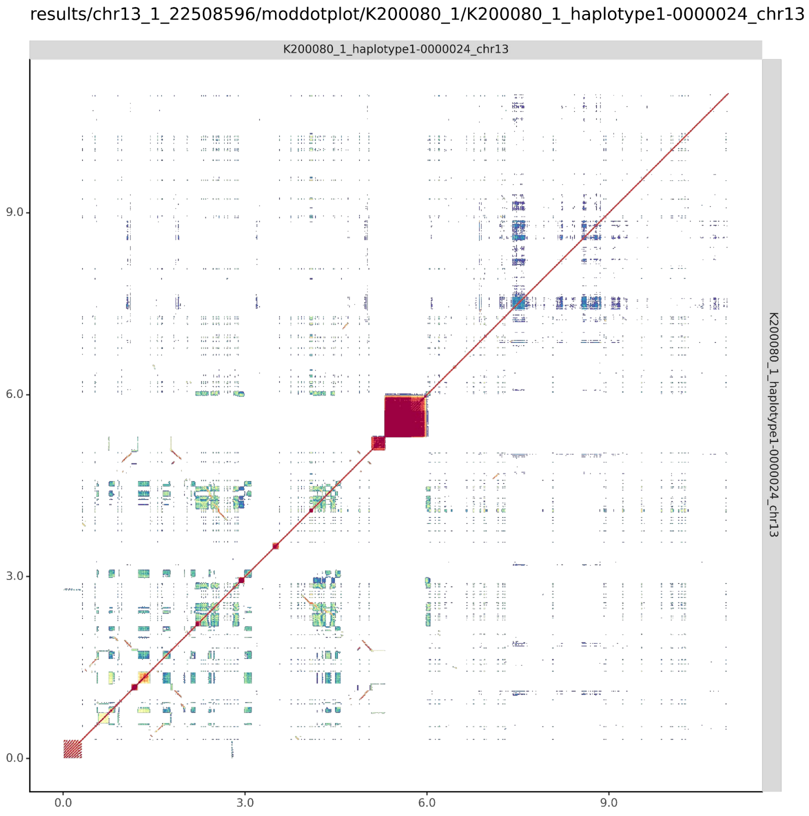

K200080_2_haplotype2-0000075_chr13

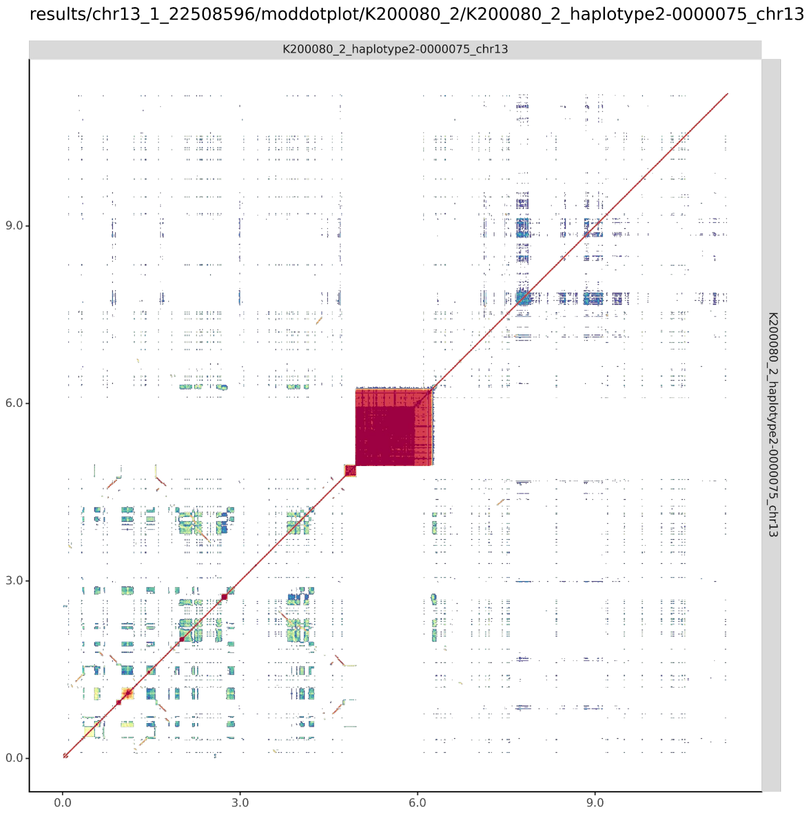

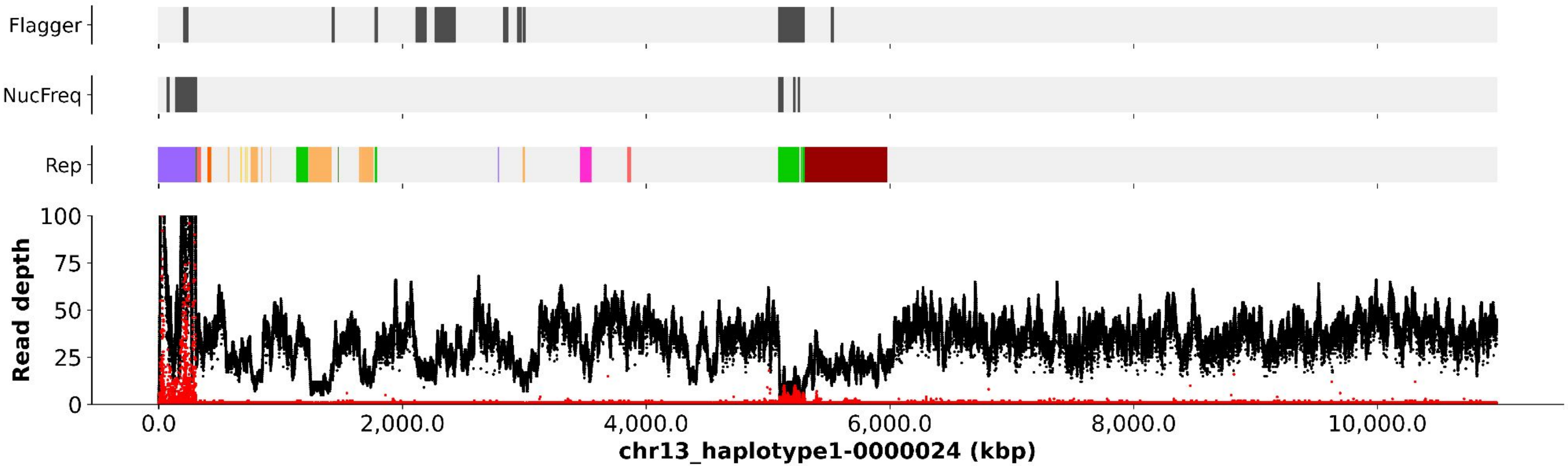
Flagger
NucFreq
Rep
Read depth
100
75
50
25
0
0.0
2,000.0
4,000.0
6,000.0
8,000.0
10,000.0
chr13_haplotype1-0000024 (kbp)

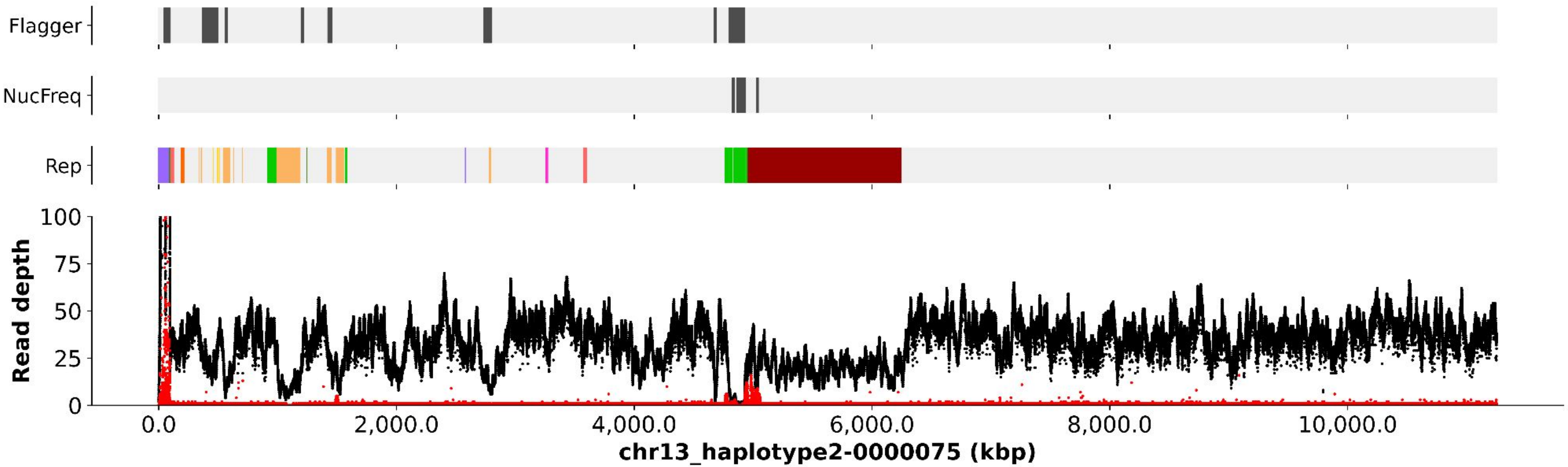
Flagger
NucFreq
Rep
Read depth
100
75
50
25
0
0.0
2,000.0
4,000.0
6,000.0
8,000.0
10,000.0
chr13_haplotype2-0000075 (kbp)

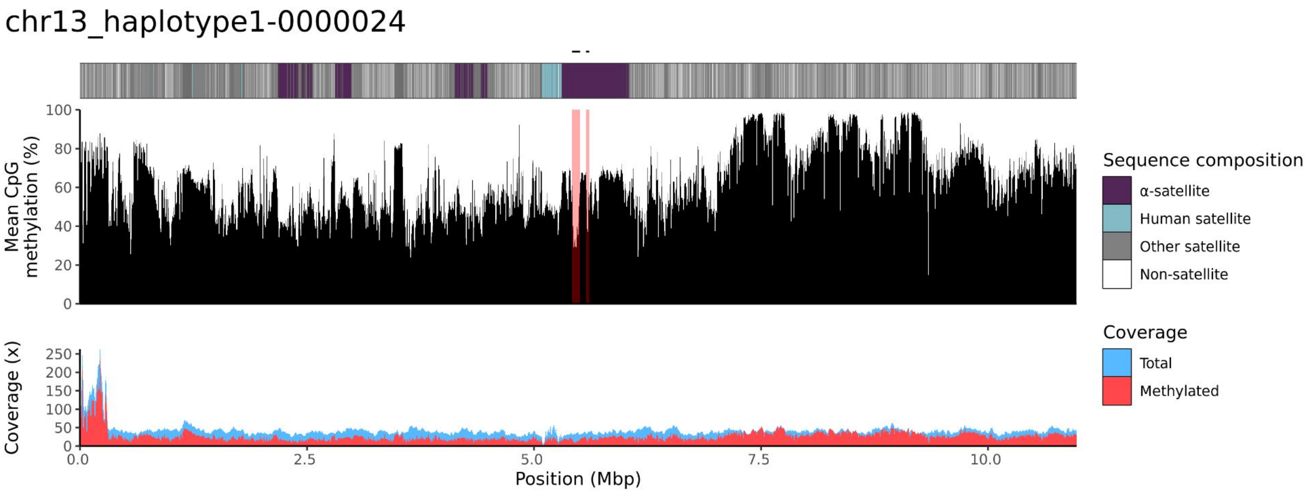
chr13_haplotype1-0000024
Mean CpG methylation (%)
100
80
60
40
20
0
Coverage (x)
250
200
150
100
50
0
0.0
2.5
5.0
7.5
10.0
Position (Mbp)
Sequence composition
α-satellite
Human satellite
Other satellite
Non-satellite
Coverage
Total
Methylated

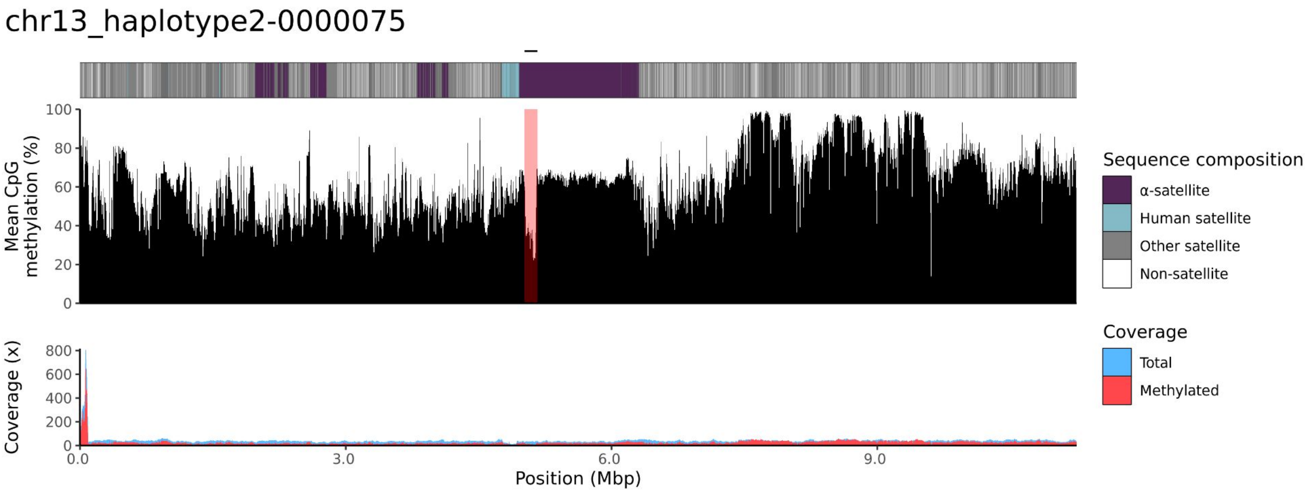
chr13_haplotype2-0000075
Mean CpG methylation (%)
100
80
60
40
20
0
Coverage (x)
800
600
400
200
0
0.0
3.0
6.0
9.0
Position (Mbp)
Sequence composition
α-satellite
Human satellite
Other satellite
Non-satellite
Coverage
Total
Methylated

chr14

K200080_1_haplotype1-0000006_chr14

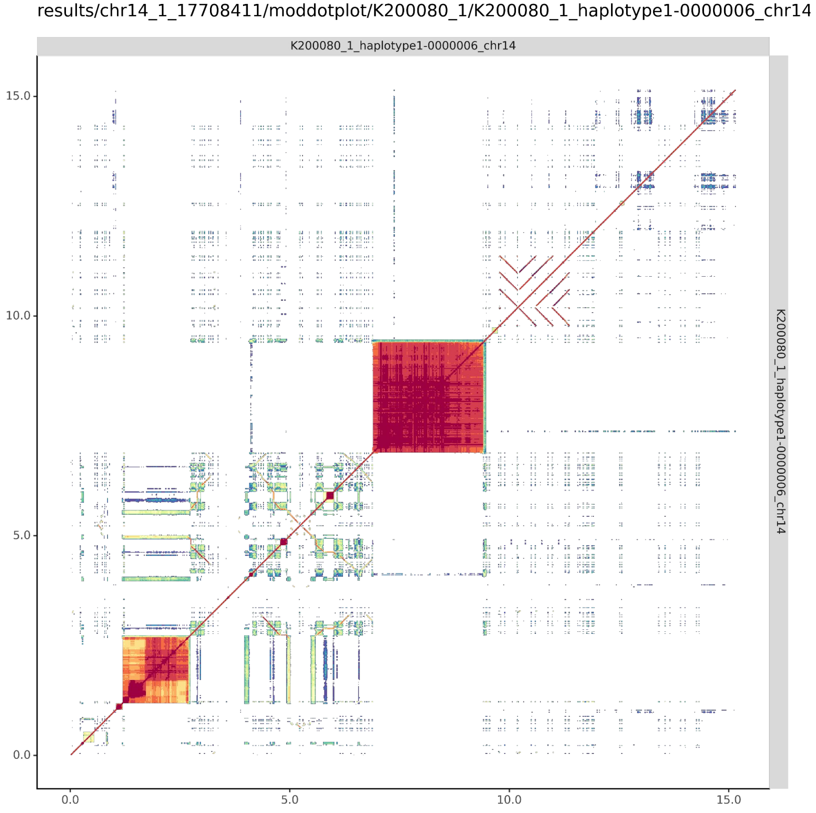

K200080_2_haplotype2-0000055_chr14

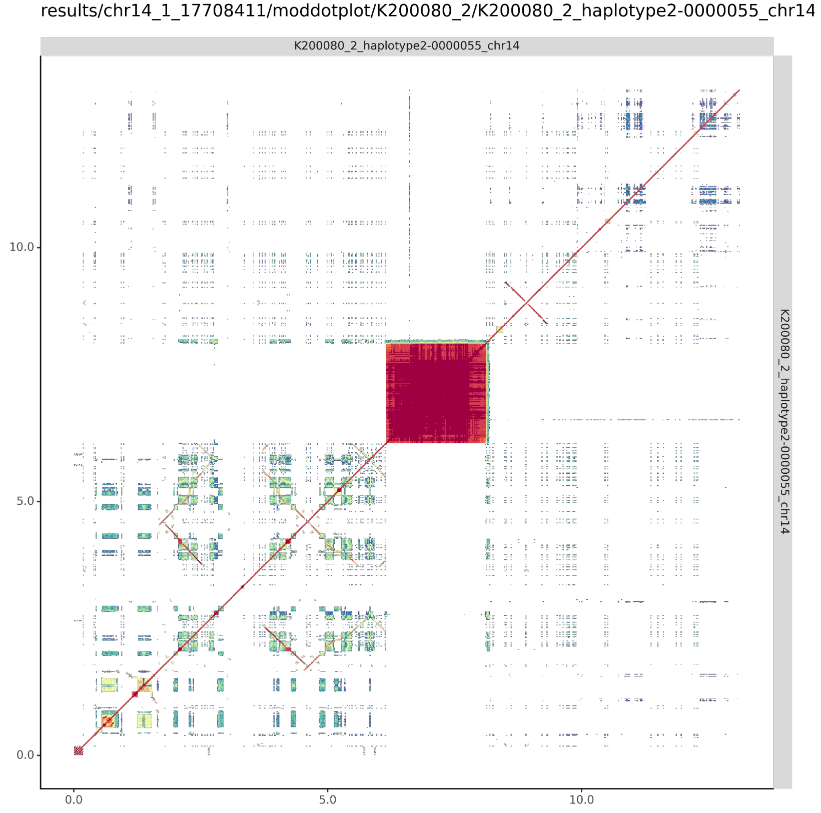

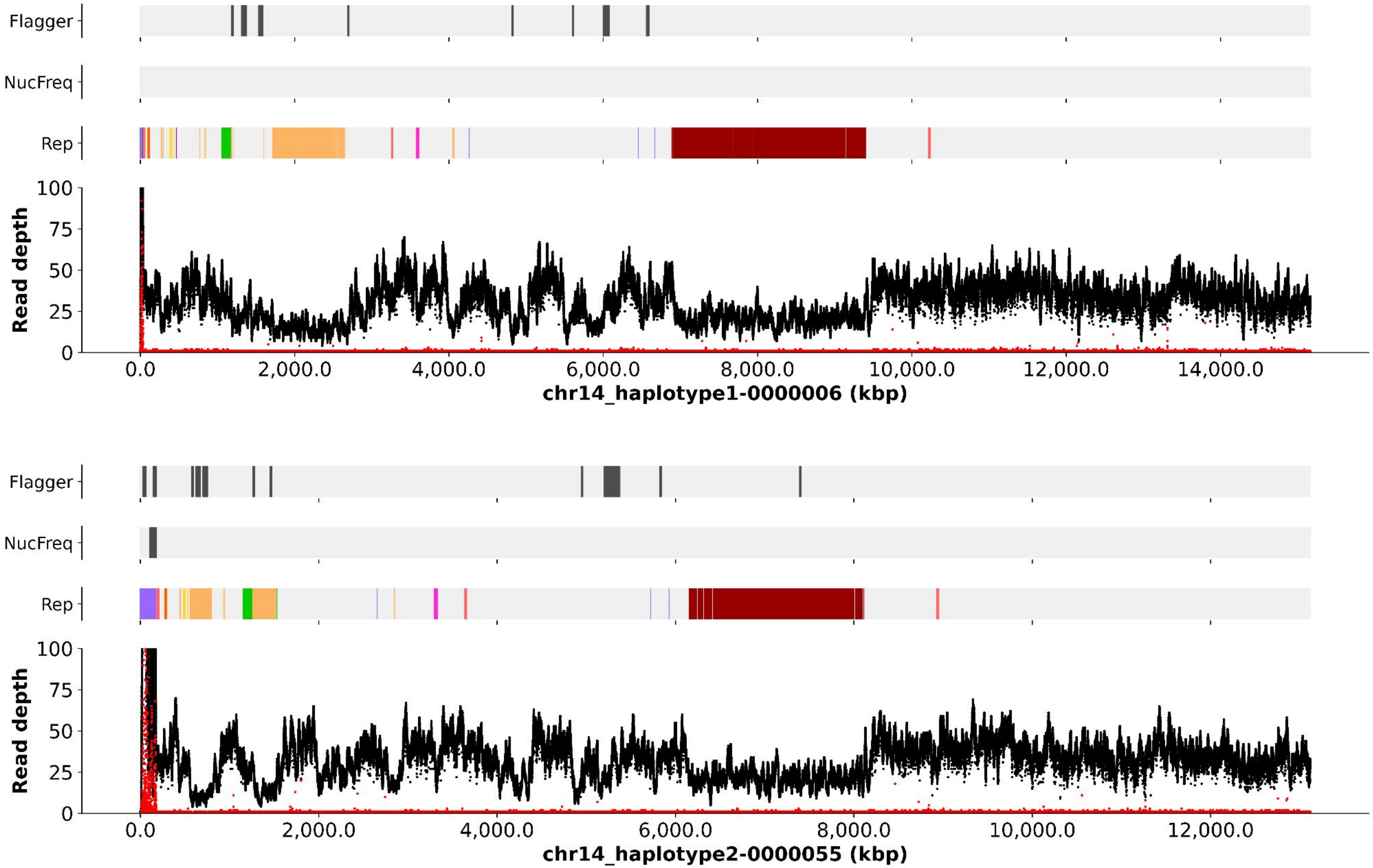
Flagger
NucFreq
Rep
Read depth
100
75
50
25
0
0.0
2,000.0
4,000.0
6,000.0
8,000.0
10,000.0
12,000.0
14,000.0
chr14_haplotype1-0000006 (kbp)
Flagger
NucFreq
Rep
Read depth
100
75
50
25
0
0.0
2,000.0
4,000.0
6,000.0
8,000.0
10,000.0
12,000.0
chr14_haplotype2-0000055 (kbp)

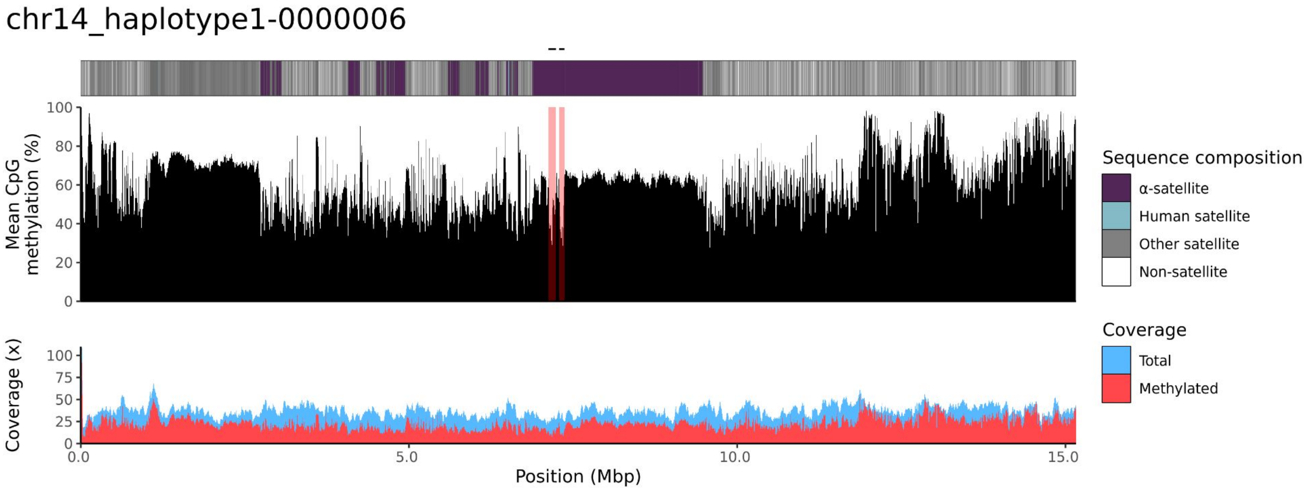

chr14_haplotype1-0000006
Mean CpG methylation (%)
Coverage (x)
Position (Mbp)
Sequence composition
α-satellite
Human satellite
Other satellite
Non-satellite
Coverage
Total
Methylated

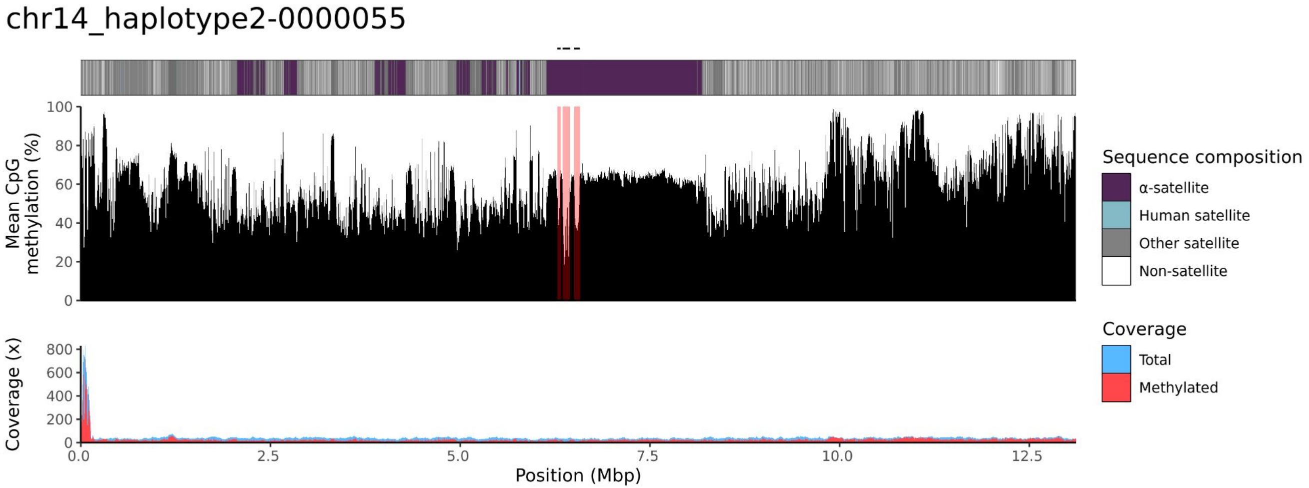

chr14_haplotype2-0000055
Mean CpG methylation (%)
Coverage (x)
Position (Mbp)
Sequence composition
α-satellite
Human satellite
Other satellite
Non-satellite
Coverage
Total
Methylated

chr15

K200080_1_haplotype1-0000026_chr15

results/chr15_1_22694466/moddotplot/K200080_1/K200080_1_haplotype1-0000026_chr15

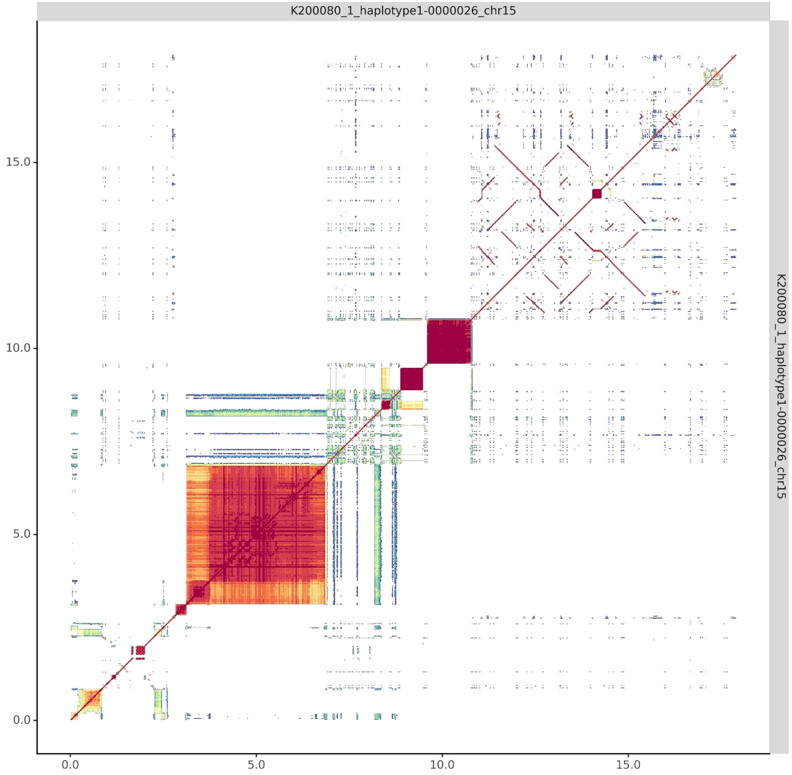

K200080_2_haplotype2-0000071_chr15

results/chr15_1_22694466/moddotplot/K200080_2/K200080_2_haplotype2-0000071_chr15

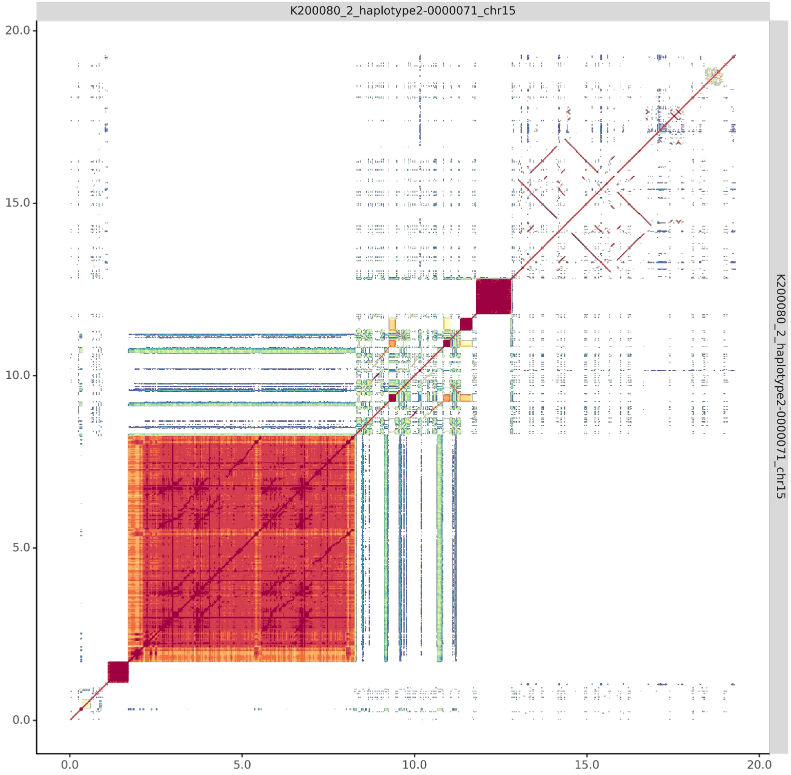

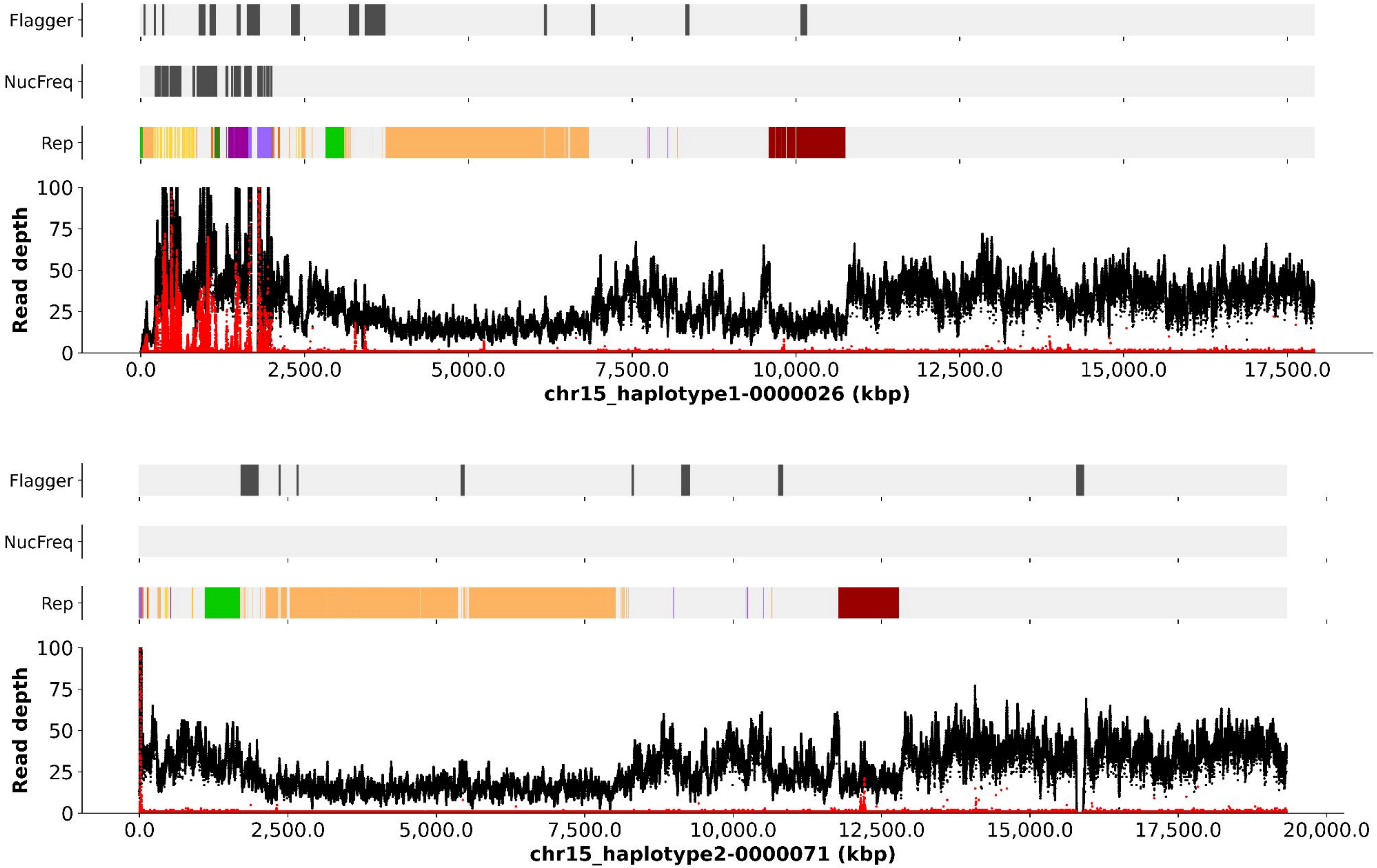
Flagger
NucFreq
Rep
Read depth
100
75
50
25
0
0.0
2,500.0
5,000.0
7,500.0
10,000.0
12,500.0
15,000.0
17,500.0
chr15_haplotype1-0000026 (kbp)
Flagger
NucFreq
Rep
Read depth
100
75
50
25
0
0.0
2,500.0
5,000.0
7,500.0
10,000.0
12,500.0
15,000.0
17,500.0
20,000.0
chr15_haplotype2-0000071 (kbp)

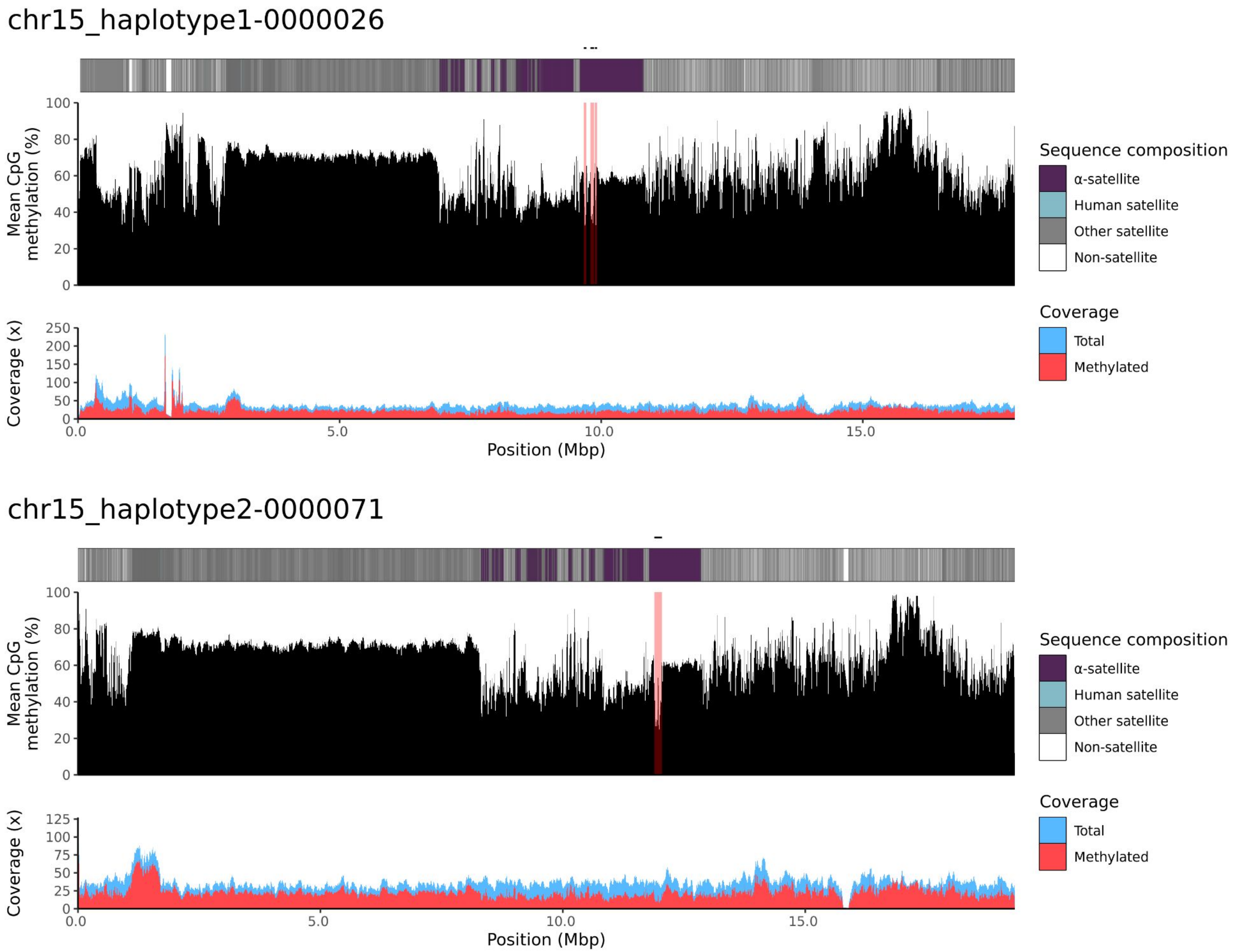
chr15_haplotype1-0000026
Mean CpG methylation (%)
100
80
60
40
20
0
Coverage (x)
250
200
150
100
50
0
0.0
5.0
10.0
15.0
Position (Mbp)
Sequence composition
α-satellite
Human satellite
Other satellite
Non-satellite
Coverage
Total
Methylated
chr15_haplotype2-0000071
Mean CpG methylation (%)
100
80
60
40
20
0
Coverage (x)
125
100
75
50
25
0
0.0
5.0
10.0
15.0
Position (Mbp)
Sequence composition
α-satellite
Human satellite
Other satellite
Non-satellite
Coverage
Total
Methylated

chr21

K200080_1_haplotype1-0000030_chr21

results/chr21_1_16306378/moddotplot/K200080_1/K200080_1_haplotype1-0000030_chr21

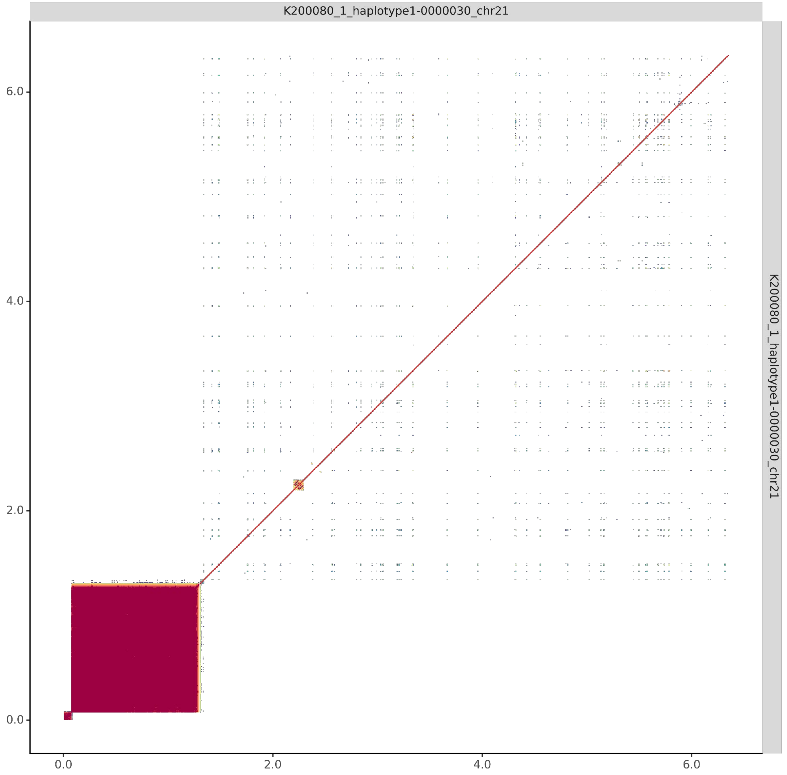

K200080_2_haplotype2-0000081_chr21

results/chr21_1_16306378/moddotplot/K200080_2/K200080_2_haplotype2-0000081_chr21

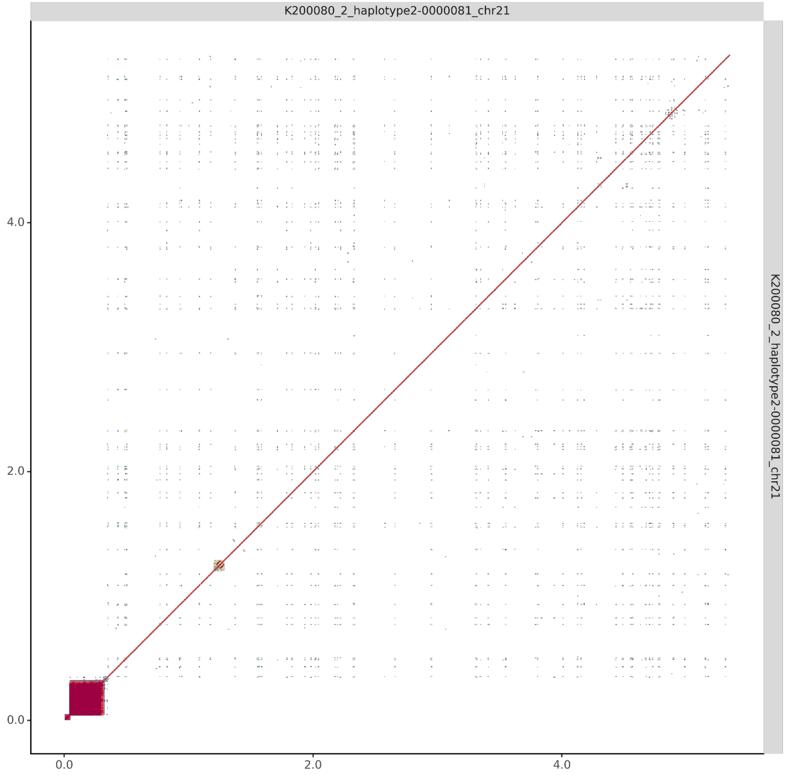

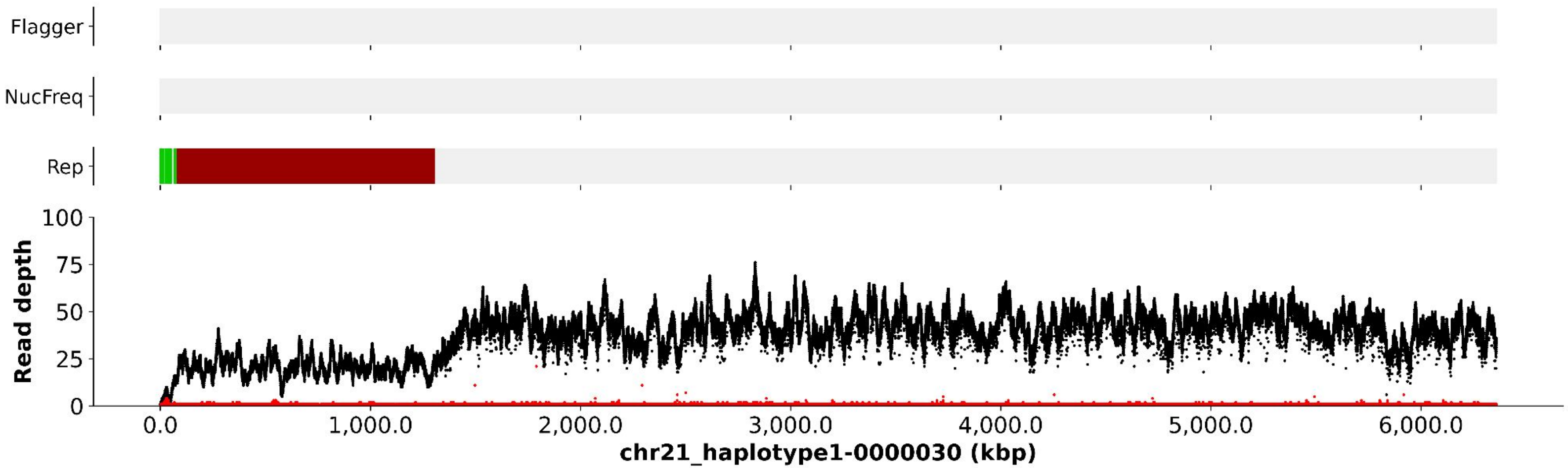
Flagger
NucFreq
Rep
Read depth
100
75
50
25
0
0.0
1,000.0
2,000.0
3,000.0
4,000.0
5,000.0
6,000.0
chr21_haplotype1-0000030 (kbp)

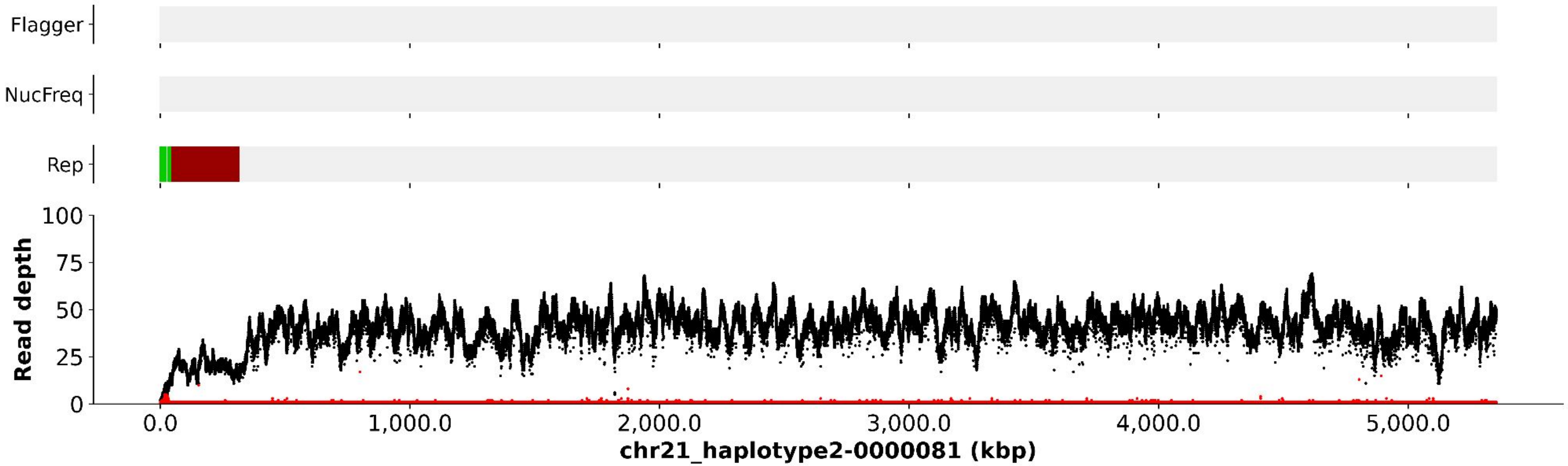
Flagger
NucFreq
Rep
Read depth
100
75
50
25
0
0.0
1,000.0
2,000.0
3,000.0
4,000.0
5,000.0
chr21_haplotype2-0000081 (kbp)

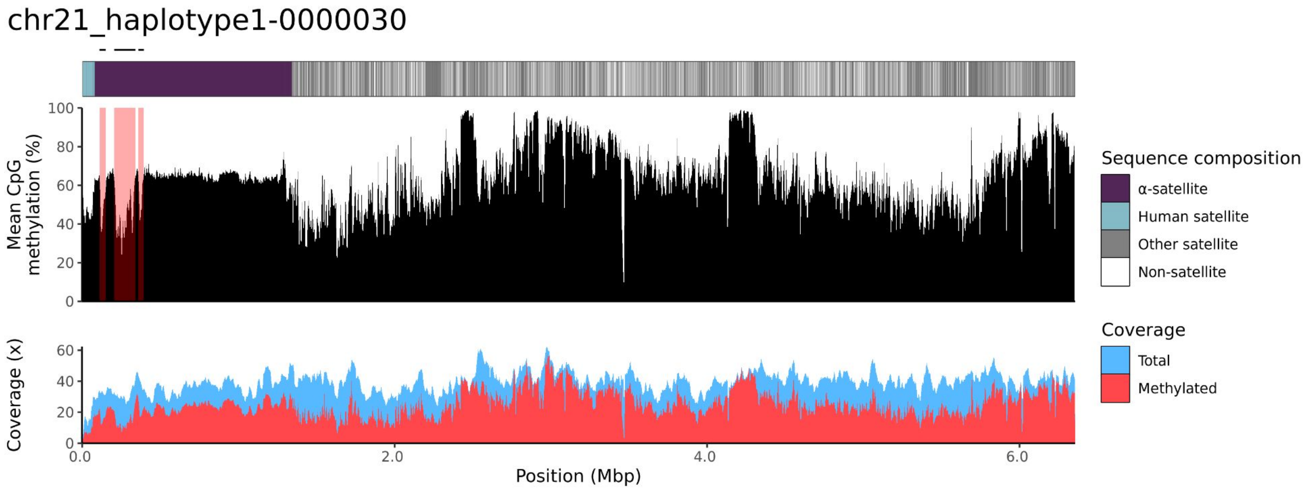
chr21_haplotype1-0000030
Mean CpG methylation (%)
Coverage (x)
Position (Mbp)
Sequence composition
α-satellite
Human satellite
Other satellite
Non-satellite
Coverage
Total
Methylated

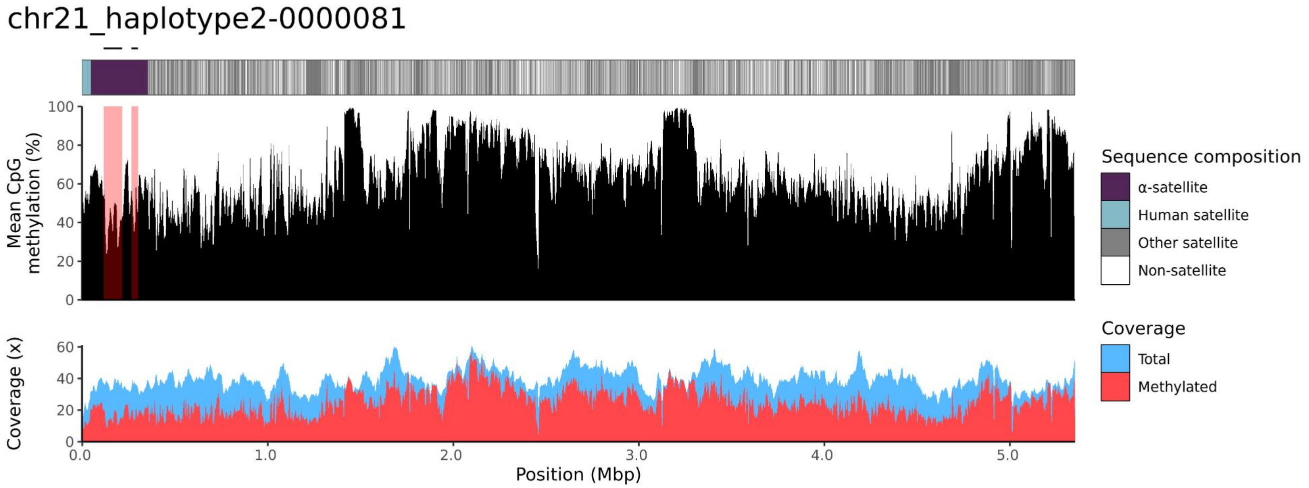
chr21_haplotype2-0000081
Mean CpG methylation (%)
Coverage (x)
Position (Mbp)
Sequence composition
α-satellite
Human satellite
Other satellite
Non-satellite
Coverage
Total
Methylated

chr22

K200080_1_haplotype1-0000016_chr22

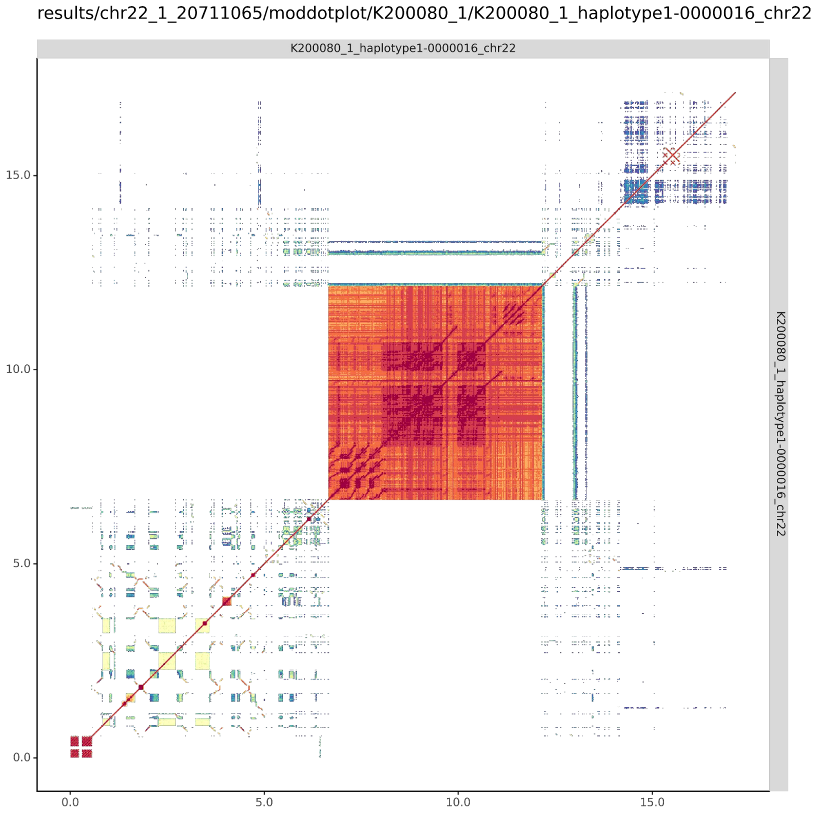

K200080_2_haplotype2-0000063_chr22

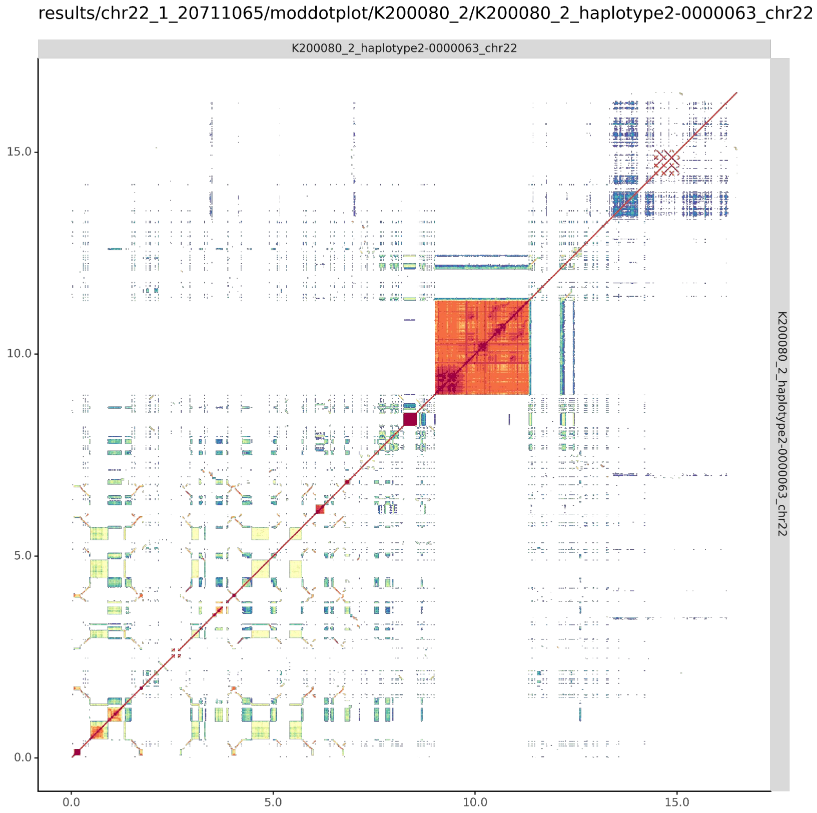

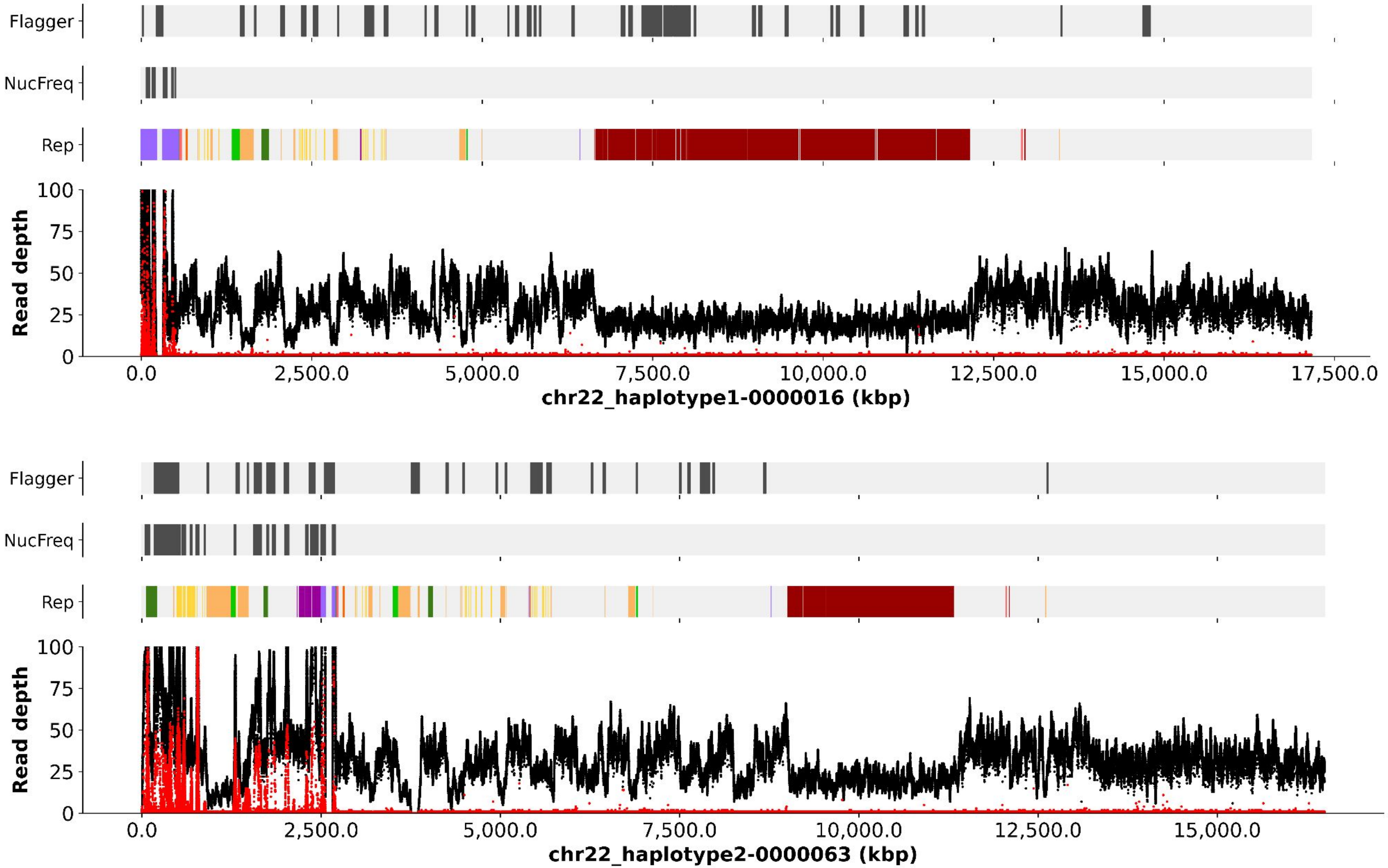
Flagger
NucFreq
Rep
Read depth
100
75
50
25
0
0.0
2,500.0
5,000.0
7,500.0
10,000.0
12,500.0
15,000.0
17,500.0
chr22_haplotype1-0000016 (kbp)
Flagger
NucFreq
Rep
Read depth
100
75
50
25
0
0.0
2,500.0
5,000.0
7,500.0
10,000.0
12,500.0
15,000.0
chr22_haplotype2-0000063 (kbp)

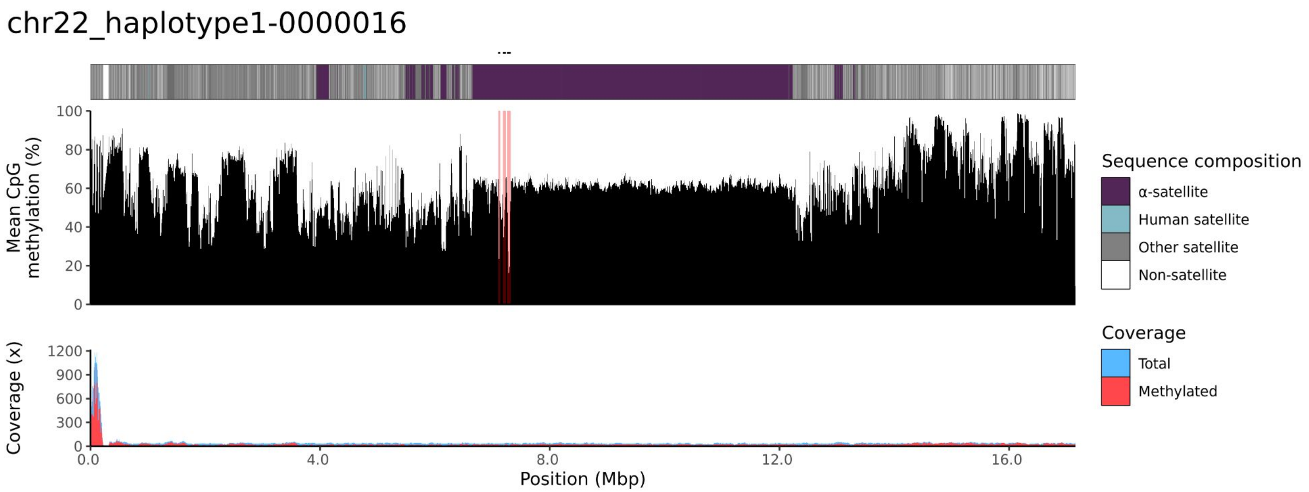

chr22_haplotype1-0000016
Mean CpG methylation (%)
100
80
60
40
20
0
Coverage (x)
1200
900
600
300
0
0.0
4.0
8.0
12.0
16.0
Position (Mbp)
Sequence composition
α-satellite
Human satellite
Other satellite
Non-satellite
Coverage
Total
Methylated

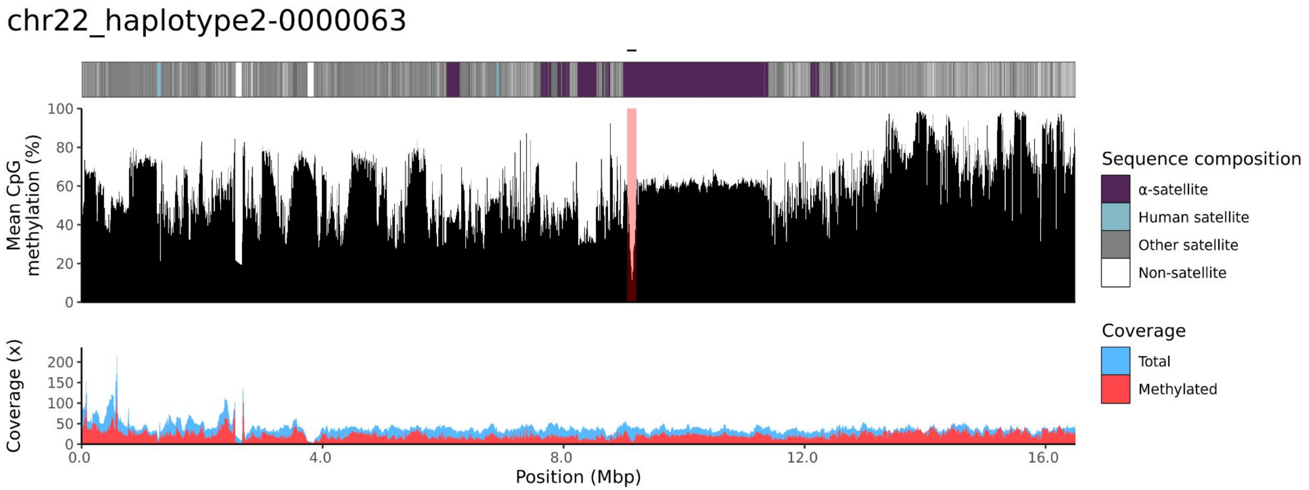

chr22_haplotype2-0000063
Mean CpG methylation (%)
100
80
60
40
20
0
Coverage (x)
200
150
100
50
0
0.0
4.0
8.0
12.0
16.0
Position (Mbp)
Sequence composition
α-satellite
Human satellite
Other satellite
Non-satellite
Coverage
Total
Methylated

200081

chr13

K200081_1_haplotype1-0000023_chr13

results/chr13_1_22508596/moddotplot/K200081_1/K200081_1_haplotype1-0000023_chr13

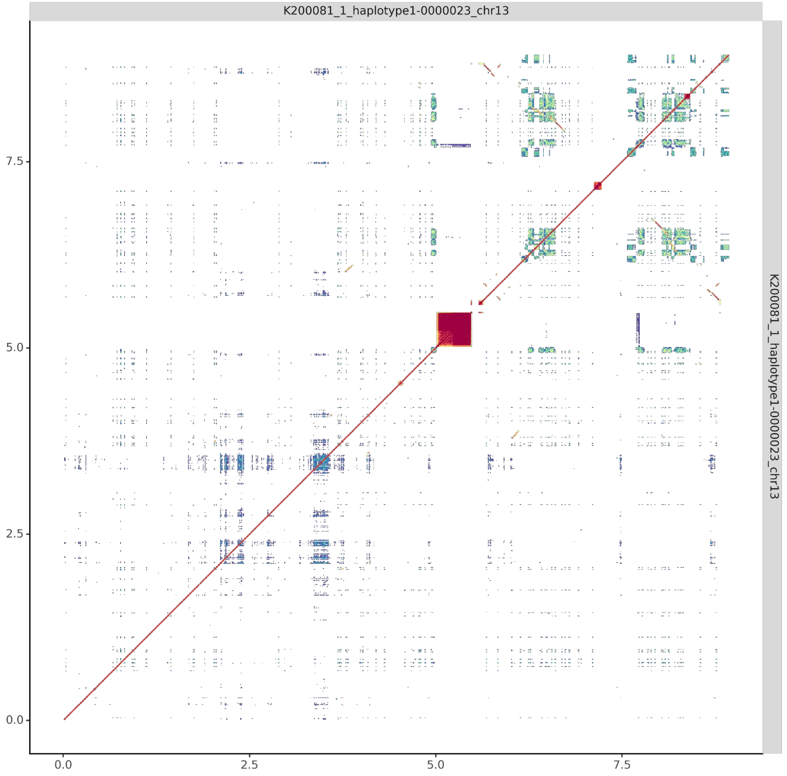

K200081_2_haplotype2-0000065_chr13

results/chr13_1_22508596/moddotplot/K200081_2/K200081_2_haplotype2-0000065_chr13

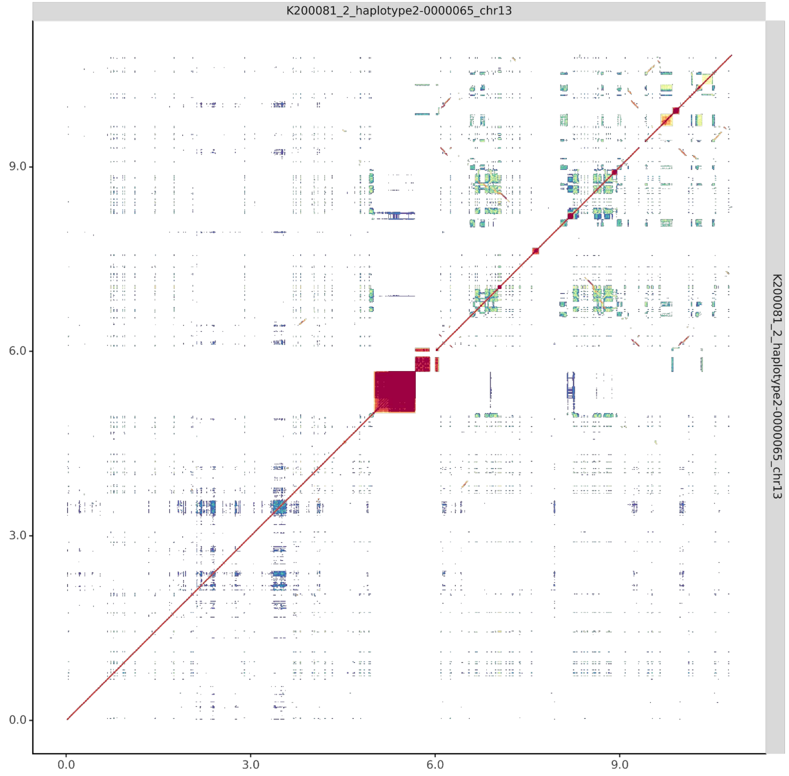

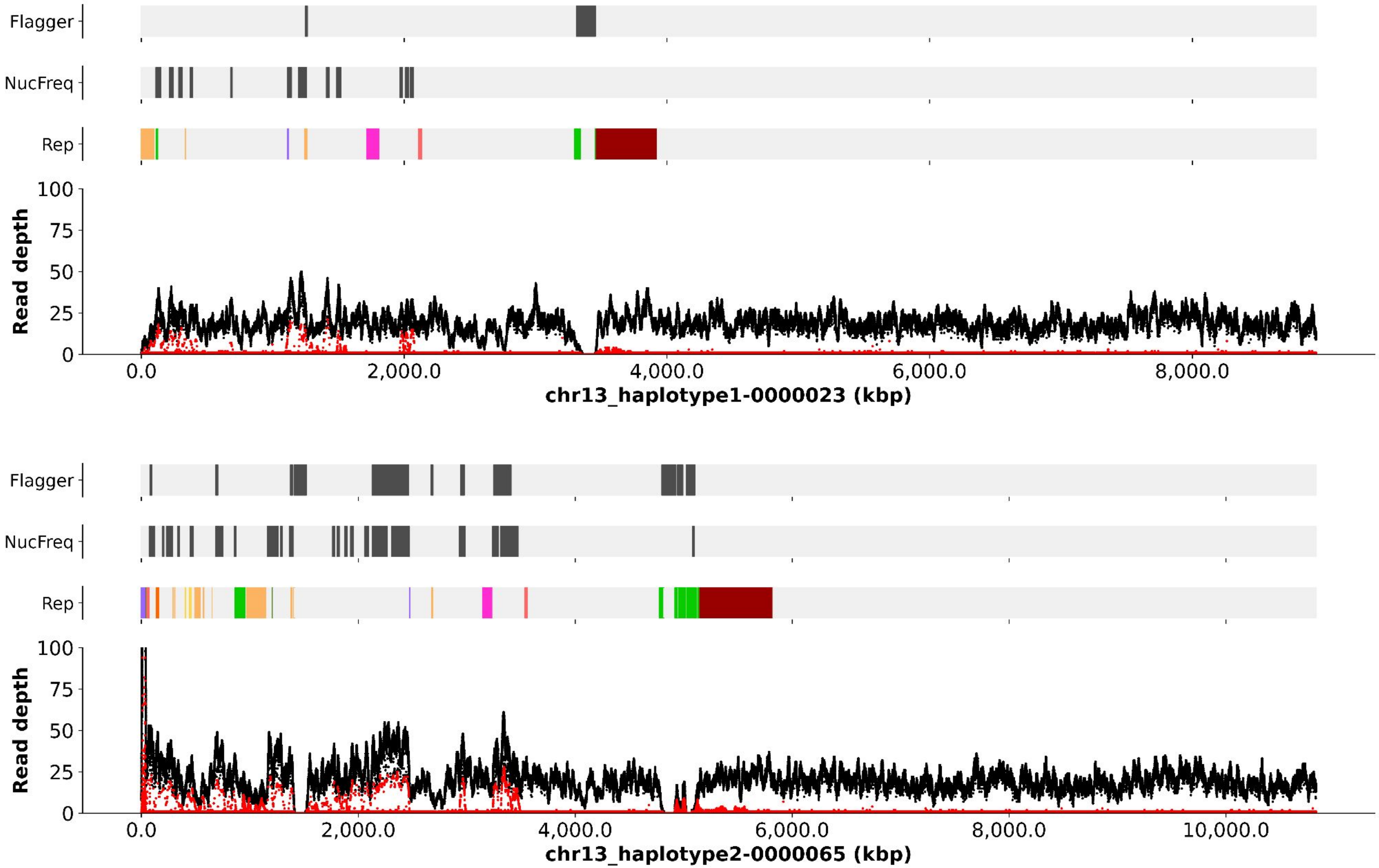

Flagger
NucFreq
Rep
Read depth
100
75
50
25
0
0.0
2,000.0
4,000.0
6,000.0
8,000.0
chr13_haplotype1-0000023 (kbp)
Flagger
NucFreq
Rep
Read depth
100
75
50
25
0
0.0
2,000.0
4,000.0
6,000.0
8,000.0
10,000.0
chr13_haplotype2-0000065 (kbp)

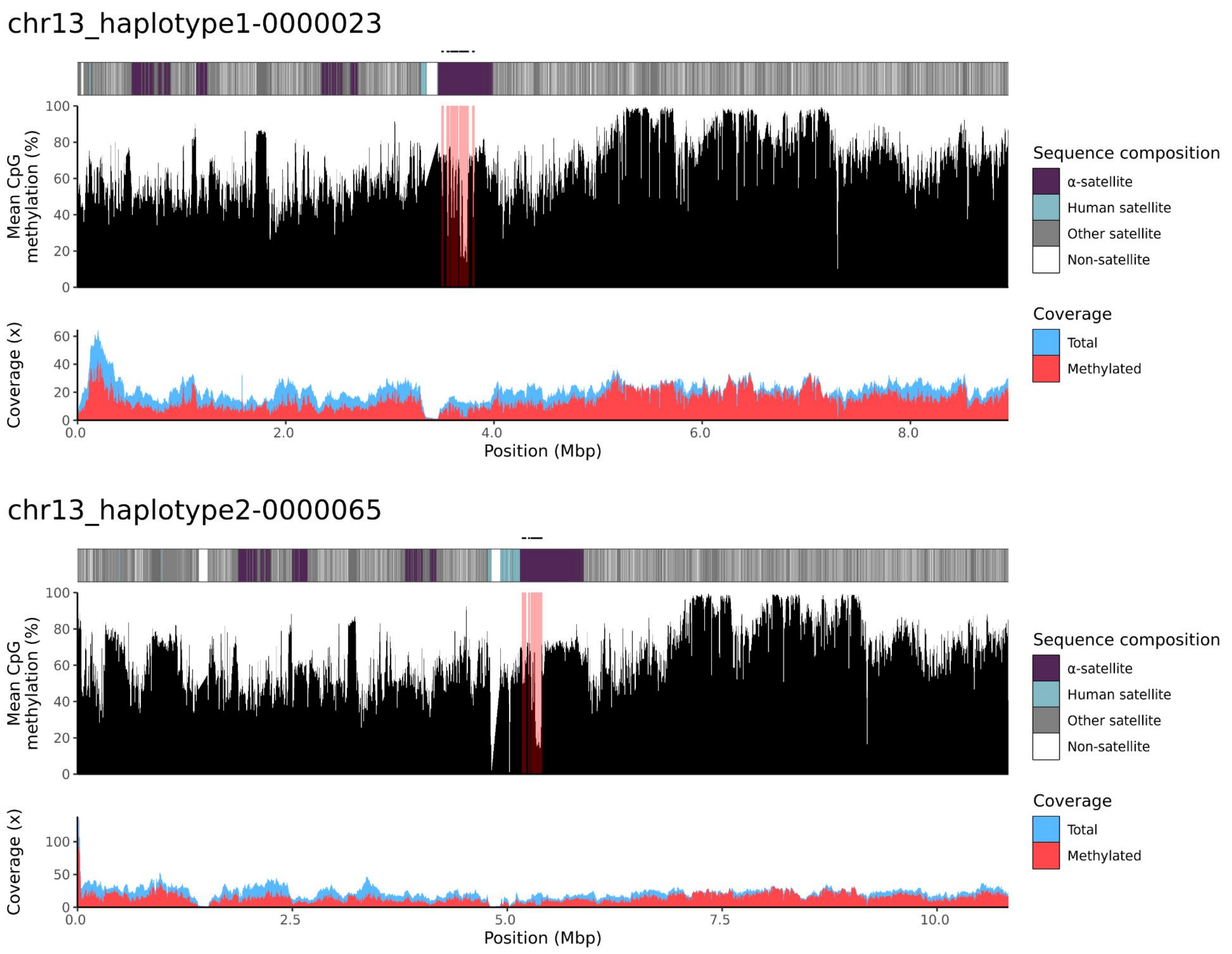

chr13_haplotype1-0000023
Mean CpG methylation (%)
Coverage (x)
Position (Mbp)
Sequence composition
α-satellite
Human satellite
Other satellite
Non-satellite
Coverage
Total
Methylated
chr13_haplotype2-0000065
Mean CpG methylation (%)
Coverage (x)
Position (Mbp)
Sequence composition
α-satellite
Human satellite
Other satellite
Non-satellite
Coverage
Total
Methylated

chr14

K200081_1_haplotype1-0000027_chr14

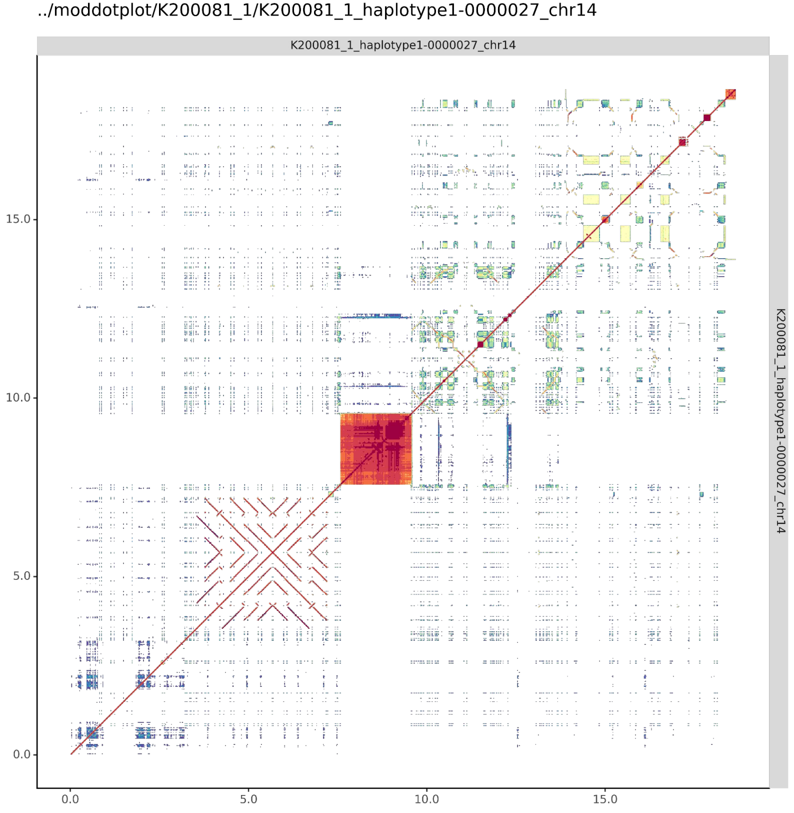

K200081_2_haplotype2-0000075_chr14

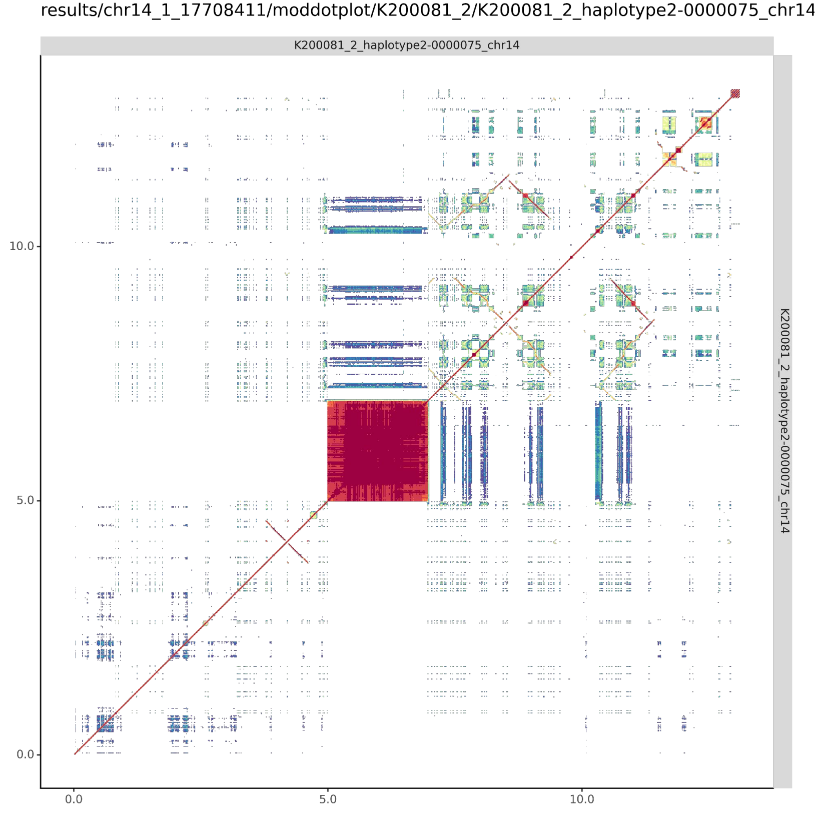

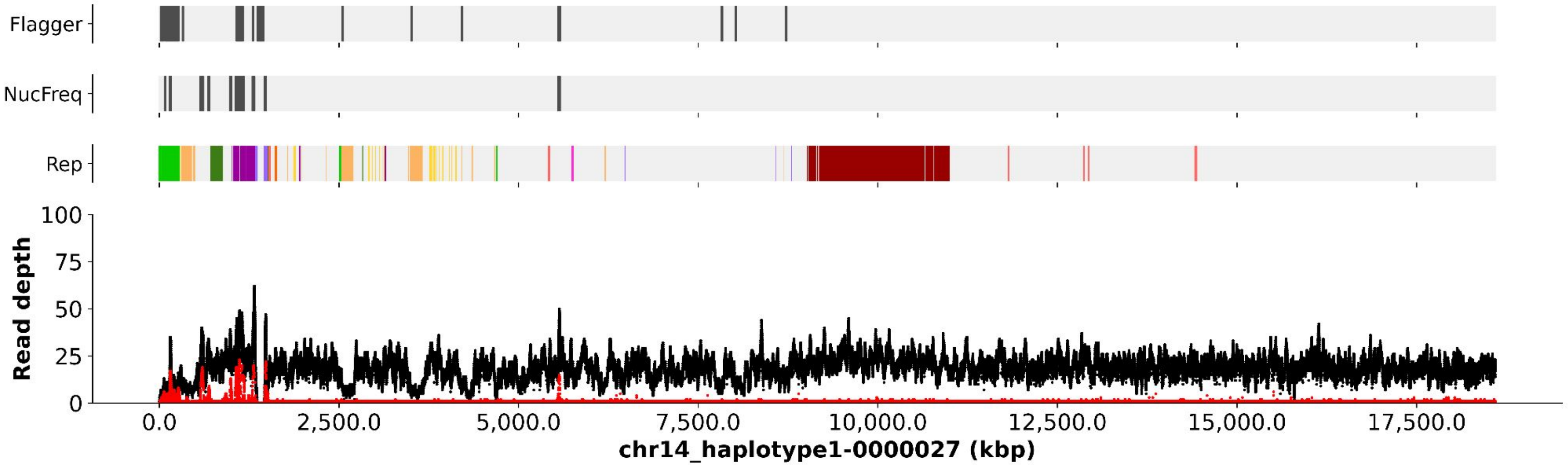

Flagger
NucFreq
Rep
Read depth
100
75
50
25
0
0.0
2,500.0
5,000.0
7,500.0
10,000.0
12,500.0
15,000.0
17,500.0
chr14_haplotype1-0000027 (kbp)

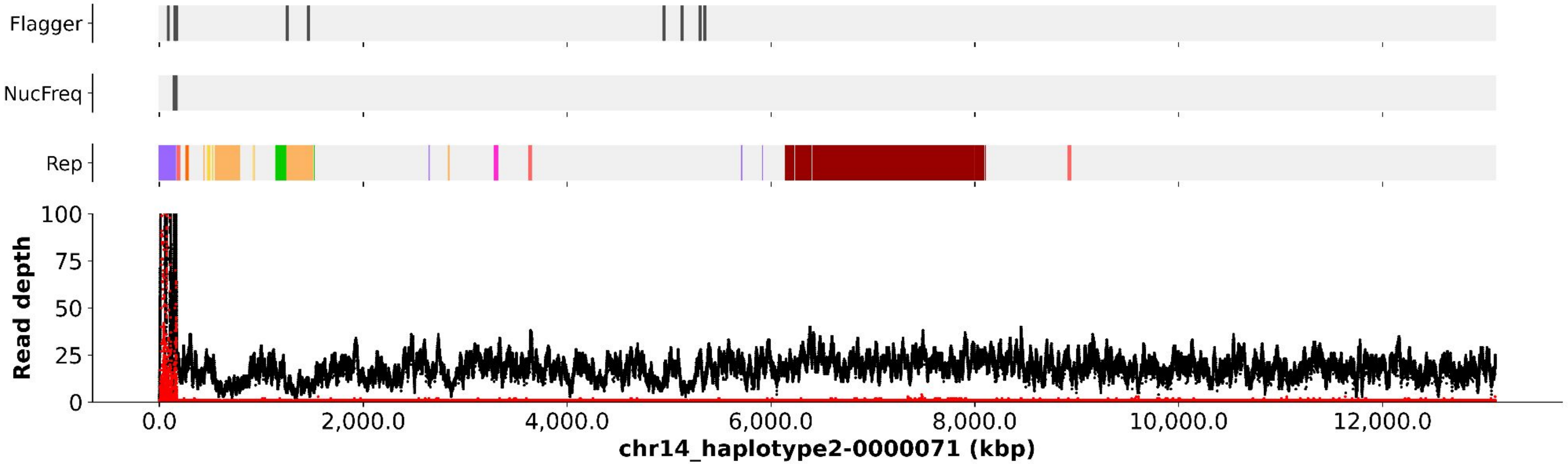

Flagger
NucFreq
Rep
Read depth
100
75
50
25
0
0.0
2,000.0
4,000.0
6,000.0
8,000.0
10,000.0
12,000.0
chr14_haplotype2-0000071 (kbp)

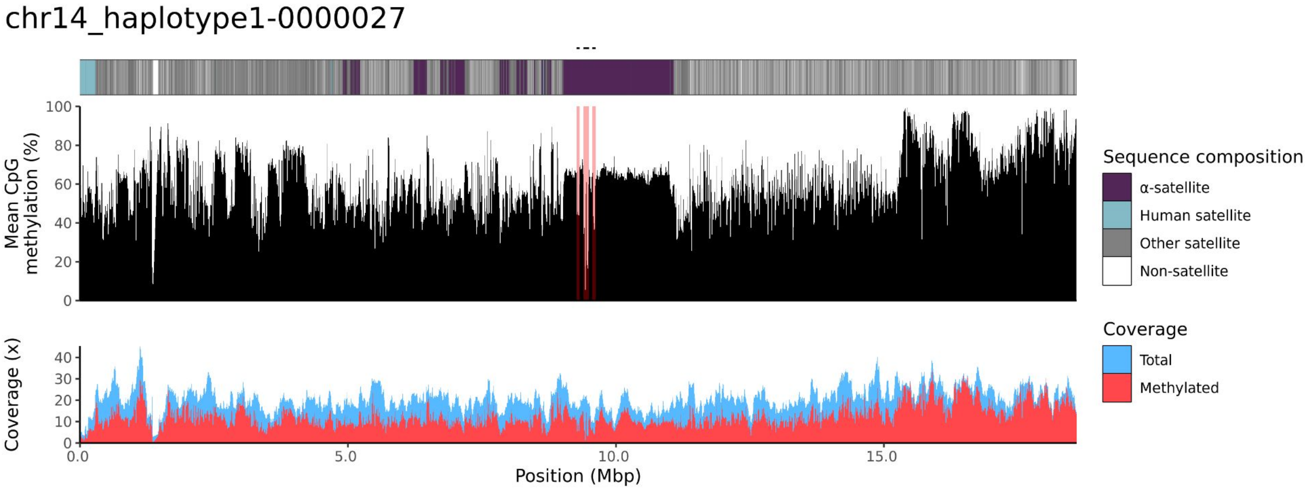
chr14_haplotype1-0000027
Mean CpG methylation (%)
Coverage (x)
Position (Mbp)
0.0
5.0
10.0
15.0
Sequence composition
α-satellite
Human satellite
Other satellite
Non-satellite
Coverage
Total
Methylated

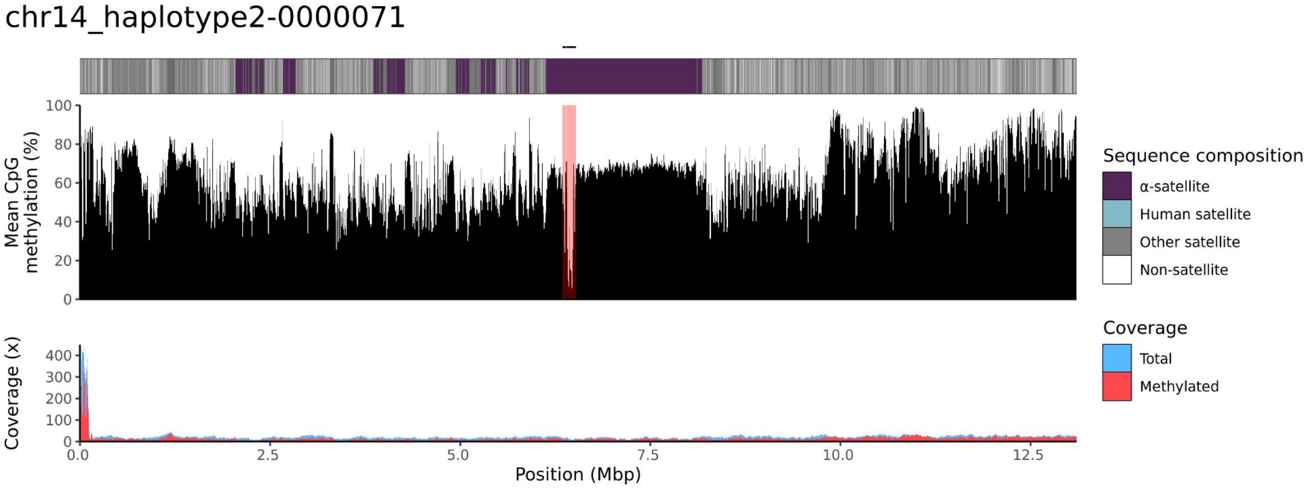
chr14_haplotype2-0000071
Mean CpG methylation (%)
Coverage (x)
Position (Mbp)
0.0
2.5
5.0
7.5
10.0
12.5
Sequence composition
α-satellite
Human satellite
Other satellite
Non-satellite
Coverage
Total
Methylated

chr15

K200081_1_haplotype1-0000009_chr15

results/chr15_1_22694466/moddotplot/K200081_1/K200081_1_haplotype1-0000009_chr15

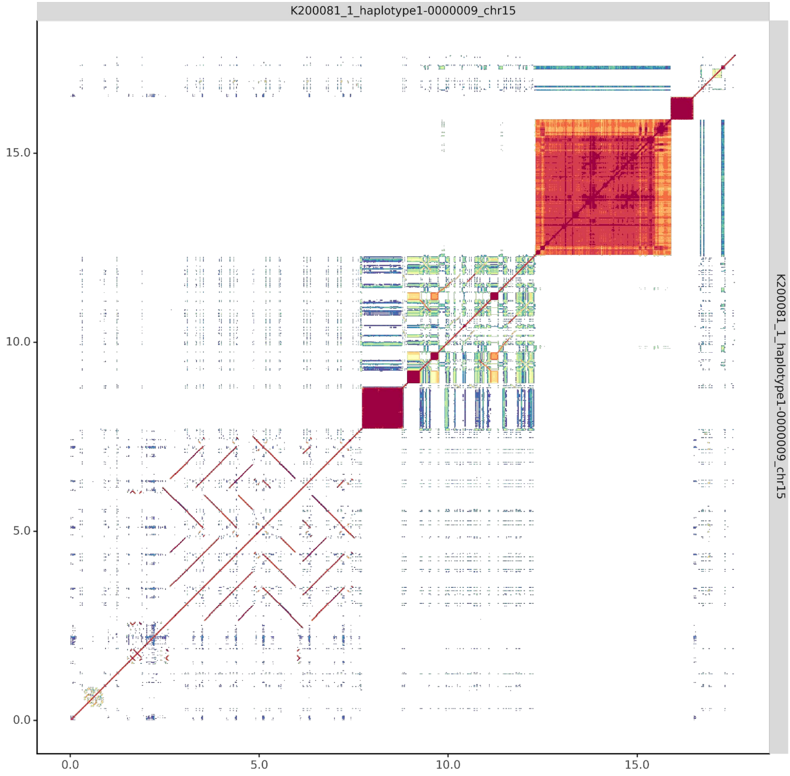

K200081_2_haplotype2-0000056_chr15

results/chr15_1_22694466/moddotplot/K200081_2/K200081_2_haplotype2-0000056_chr15

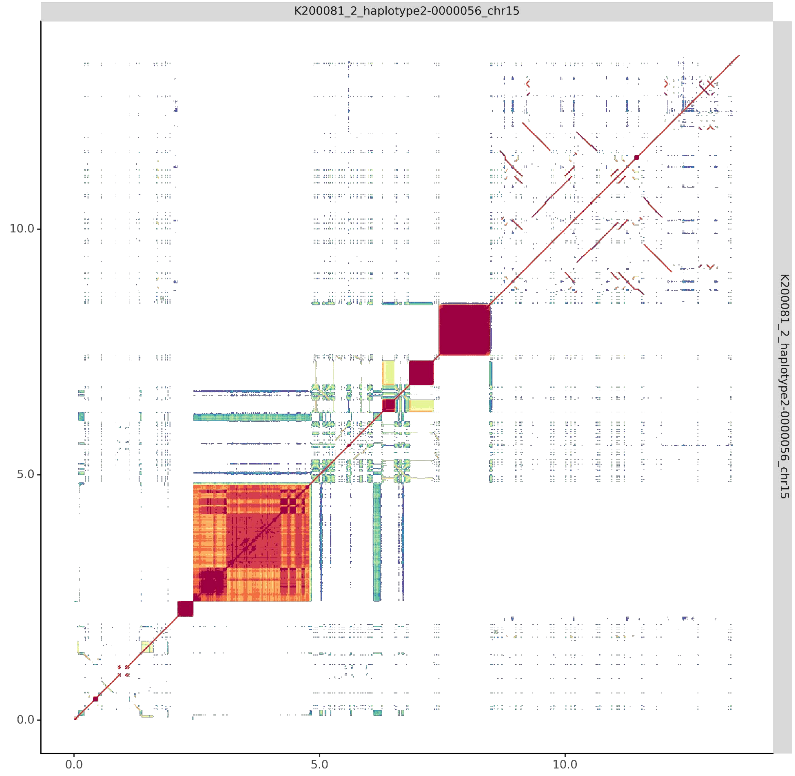

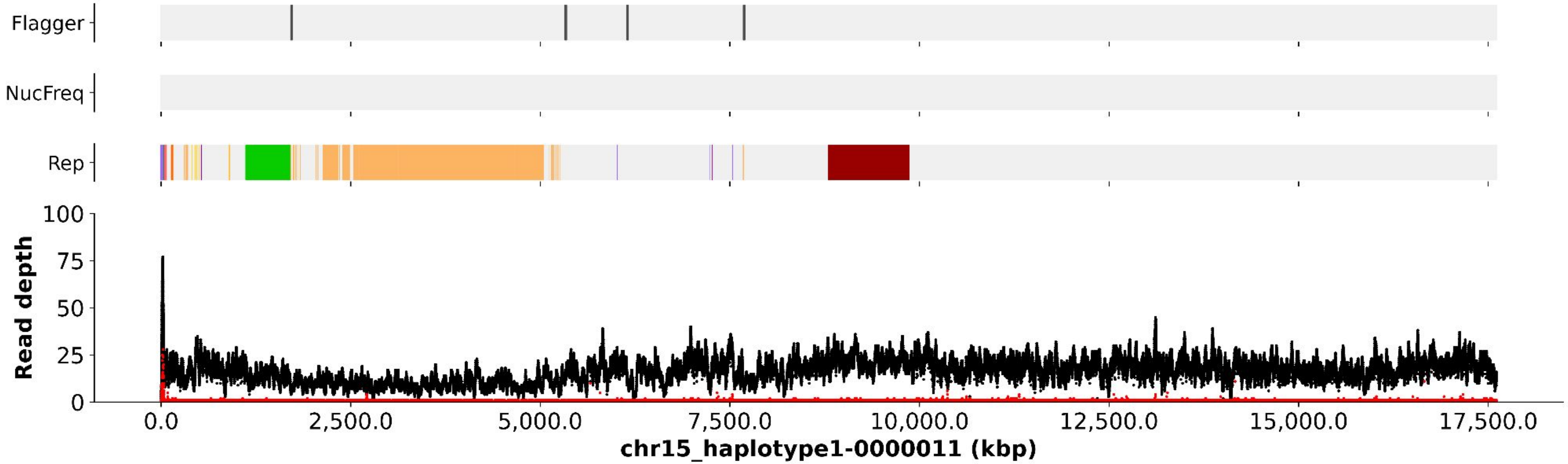
Flagger
NucFreq
Rep
Read depth
100
75
50
25
0
0.0
2,500.0
5,000.0
7,500.0
10,000.0
12,500.0
15,000.0
17,500.0
chr15_haplotype1-0000011 (kbp)

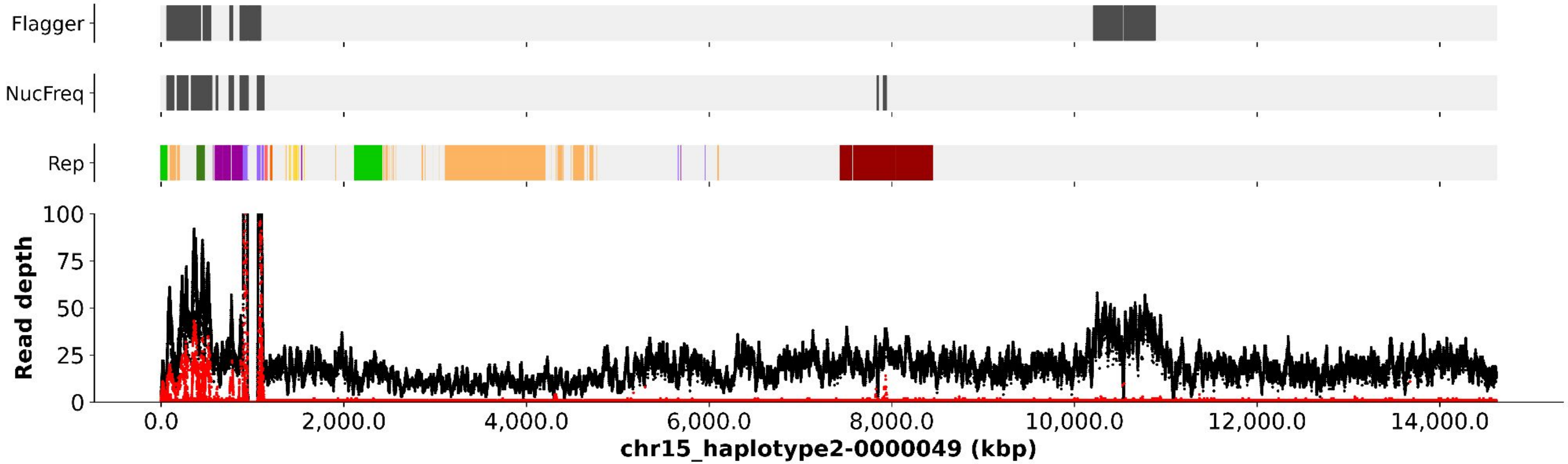
Flagger
NucFreq
Rep
Read depth
100
75
50
25
0
0.0
2,000.0
4,000.0
6,000.0
8,000.0
10,000.0
12,000.0
14,000.0
chr15_haplotype2-0000049 (kbp)

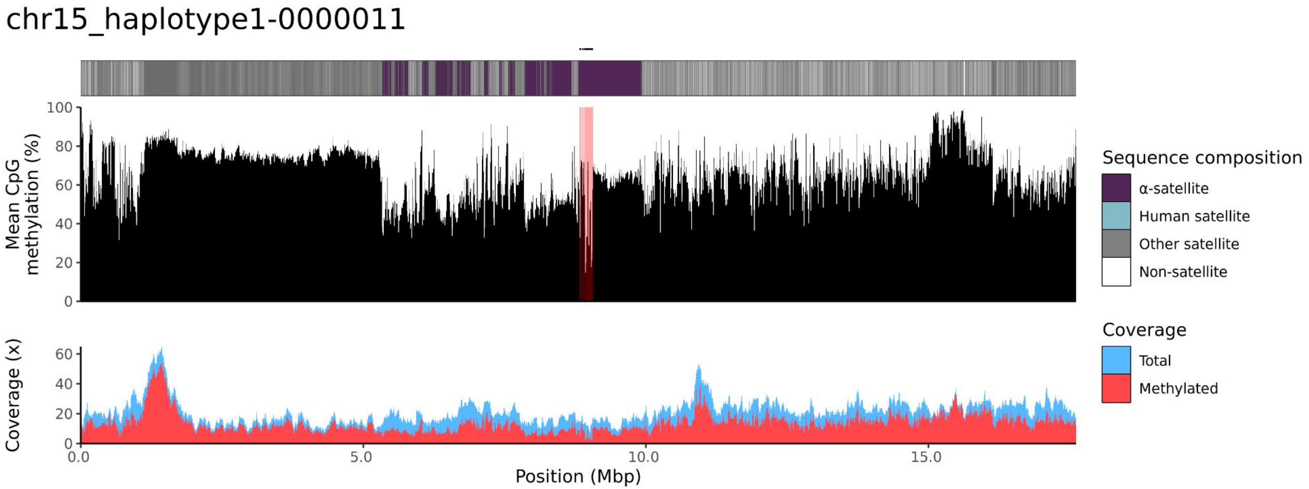
chr15_haplotype1-0000011
Mean CpG methylation (%)
Coverage (x)
Position (Mbp)
Sequence composition
α-satellite
Human satellite
Other satellite
Non-satellite
Coverage
Total
Methylated

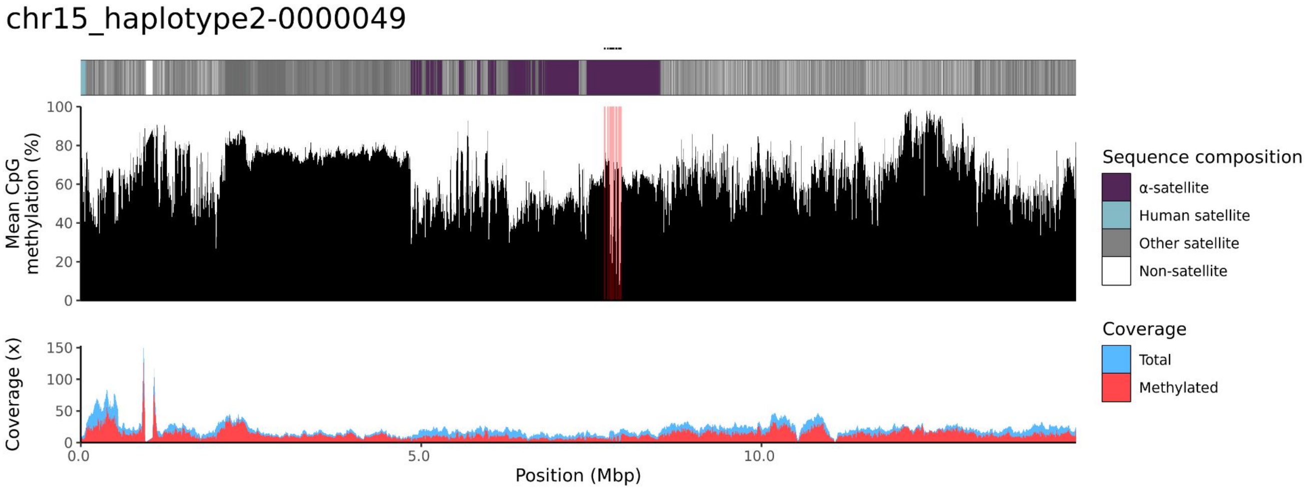
chr15_haplotype2-0000049
Mean CpG methylation (%)
Coverage (x)
Position (Mbp)
Sequence composition
α-satellite
Human satellite
Other satellite
Non-satellite
Coverage
Total
Methylated

chr21

K200081_1_haplotype1-0000015_chr21

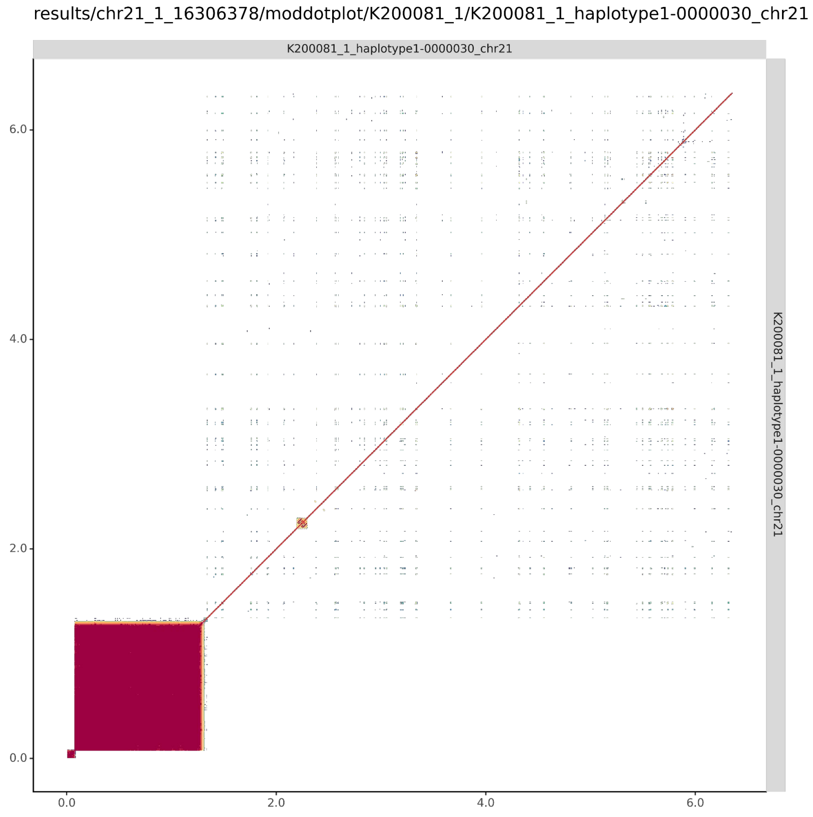

K200081_2_haplotype2-0000059_chr21

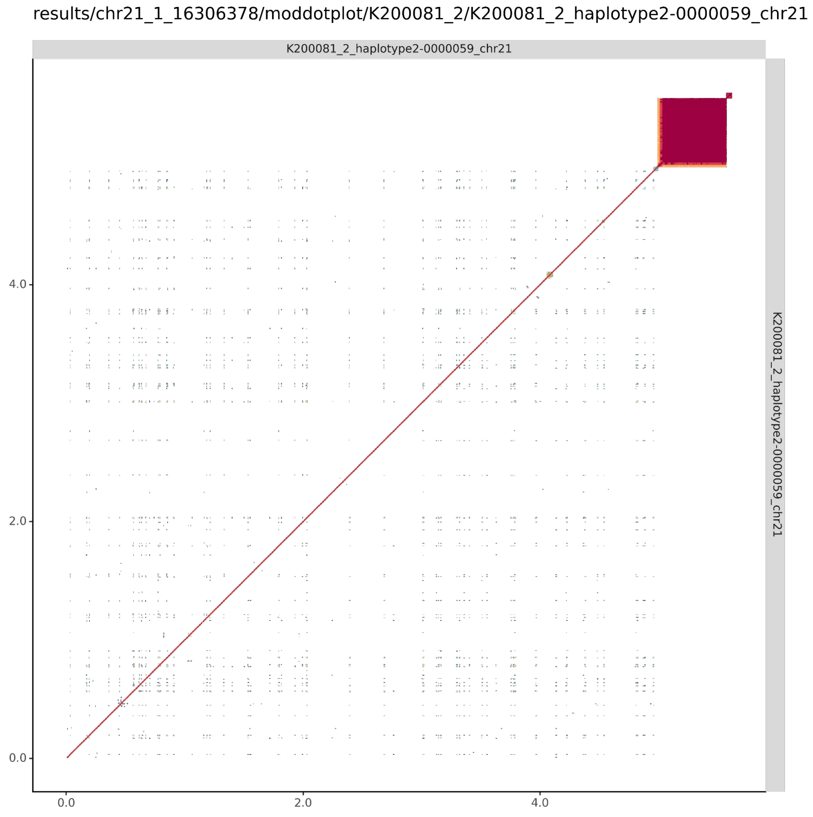

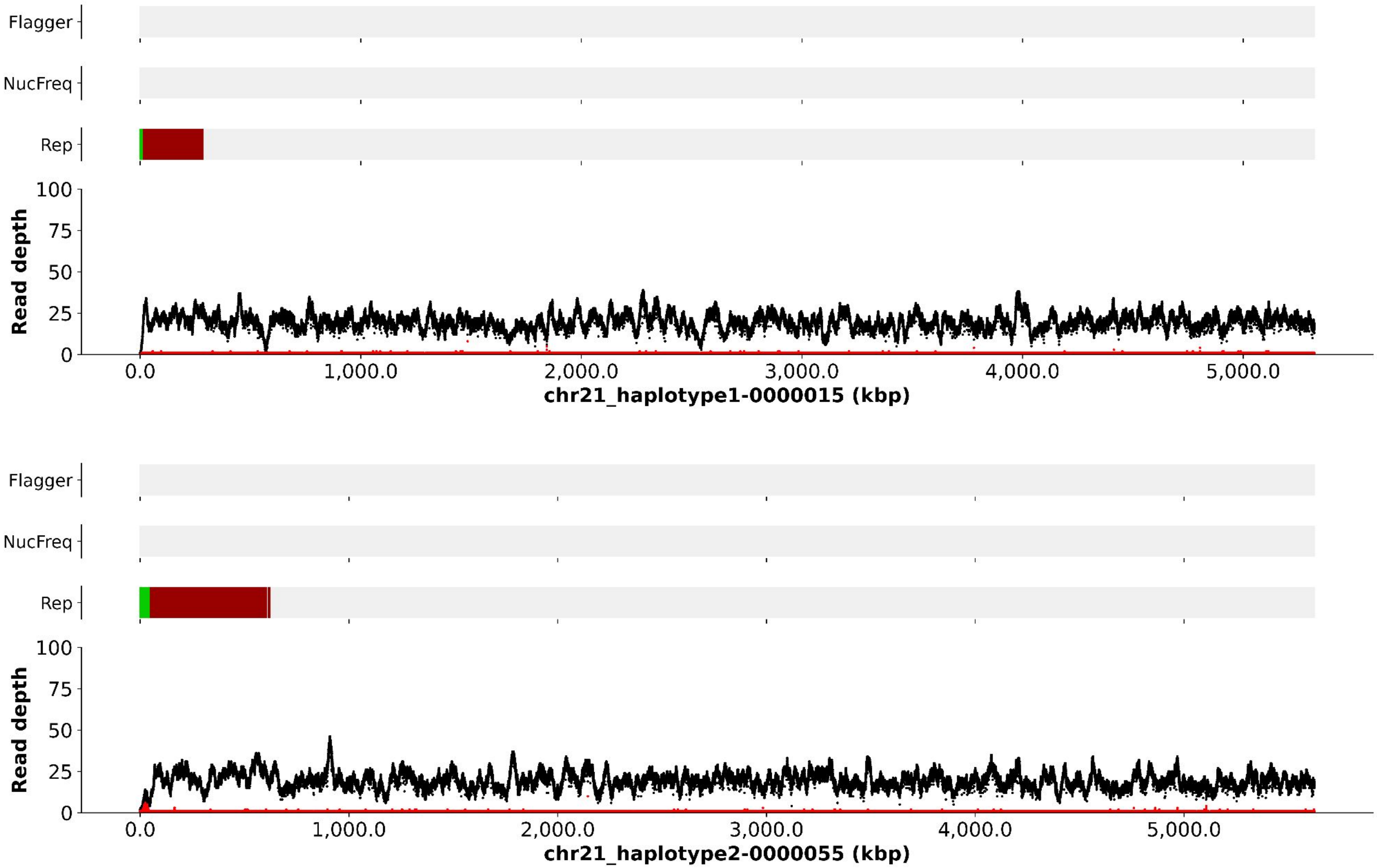

Flagger
NucFreq
Rep
Read depth
100
75
50
25
0
0.0
1,000.0
2,000.0
3,000.0
4,000.0
5,000.0
chr21_haplotype1-0000015 (kbp)
Flagger
NucFreq
Rep
Read depth
100
75
50
25
0
0.0
1,000.0
2,000.0
3,000.0
4,000.0
5,000.0
chr21_haplotype2-0000055 (kbp)

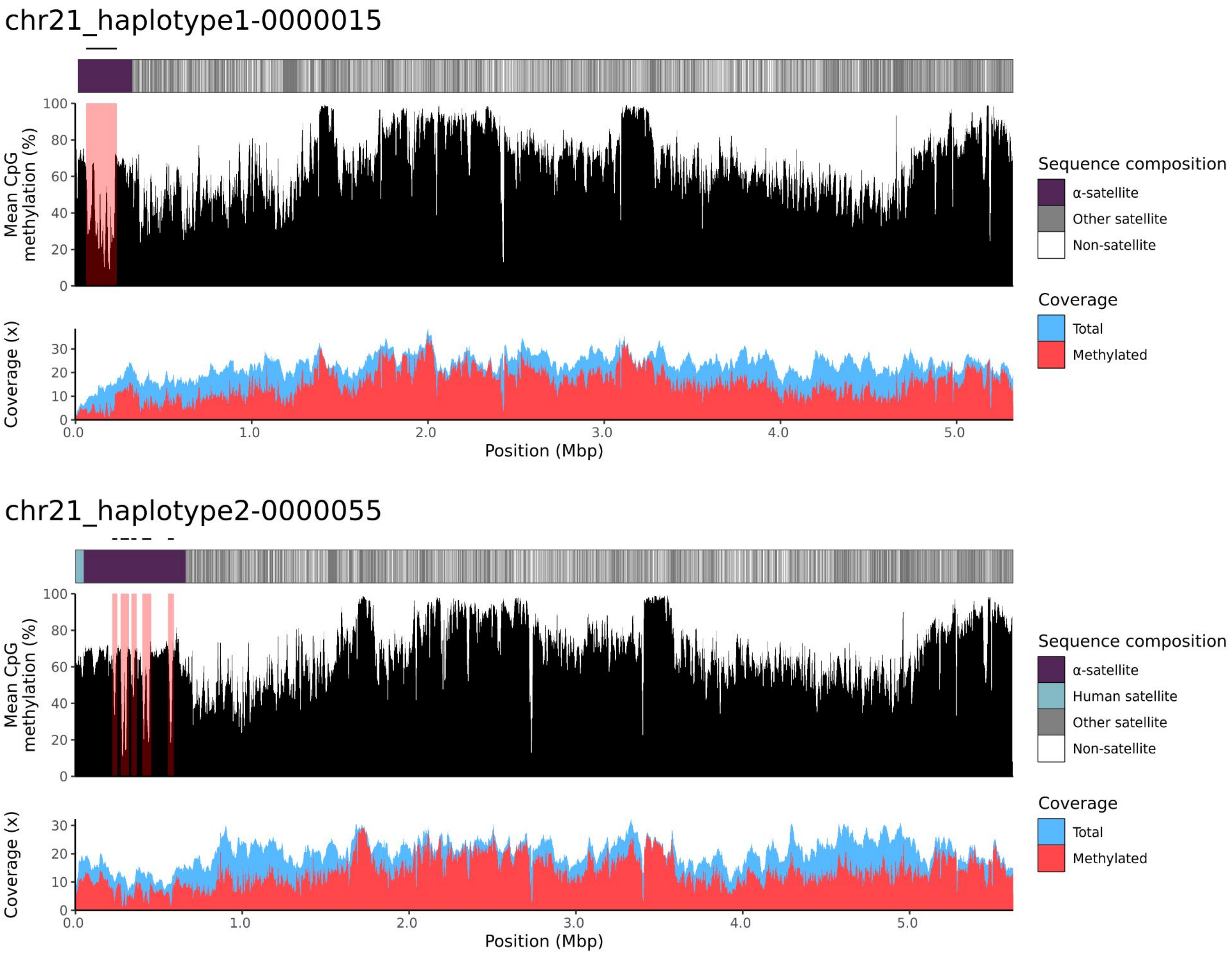
chr21_haplotype1-0000015
Mean CpG methylation (%)
Coverage (x)
Position (Mbp)
Sequence composition
α-satellite
Other satellite
Non-satellite
Coverage
Total
Methylated
chr21_haplotype2-0000055
Mean CpG methylation (%)
Coverage (x)
Position (Mbp)
Sequence composition
α-satellite
Human satellite
Other satellite
Non-satellite
Coverage
Total
Methylated

chr22

K200081_1_haplotype1-0000024_chr22

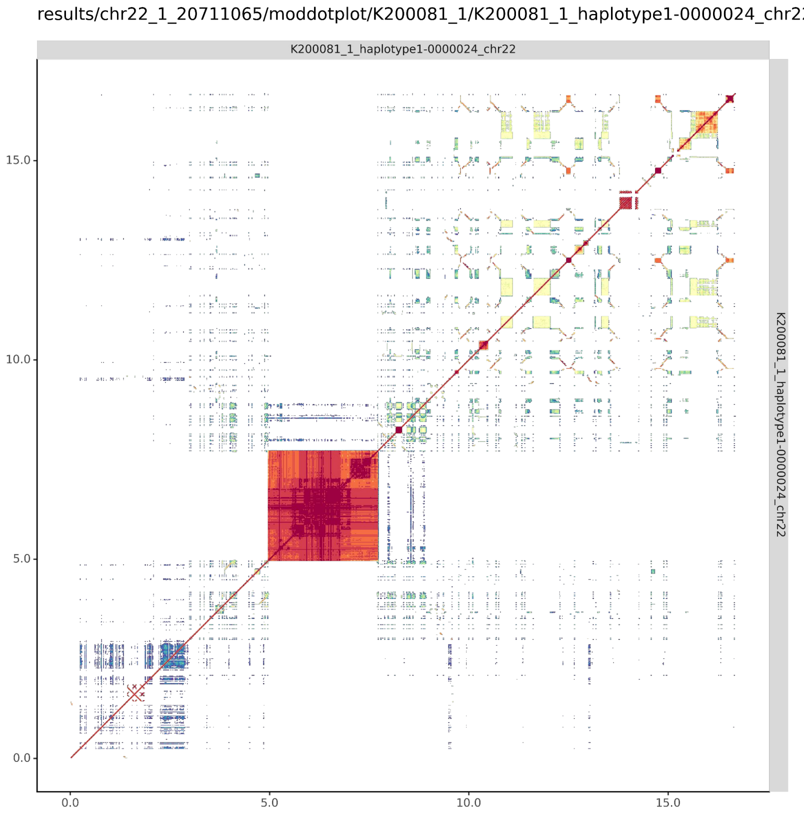

K200081_2_haplotype2-0000072_chr22

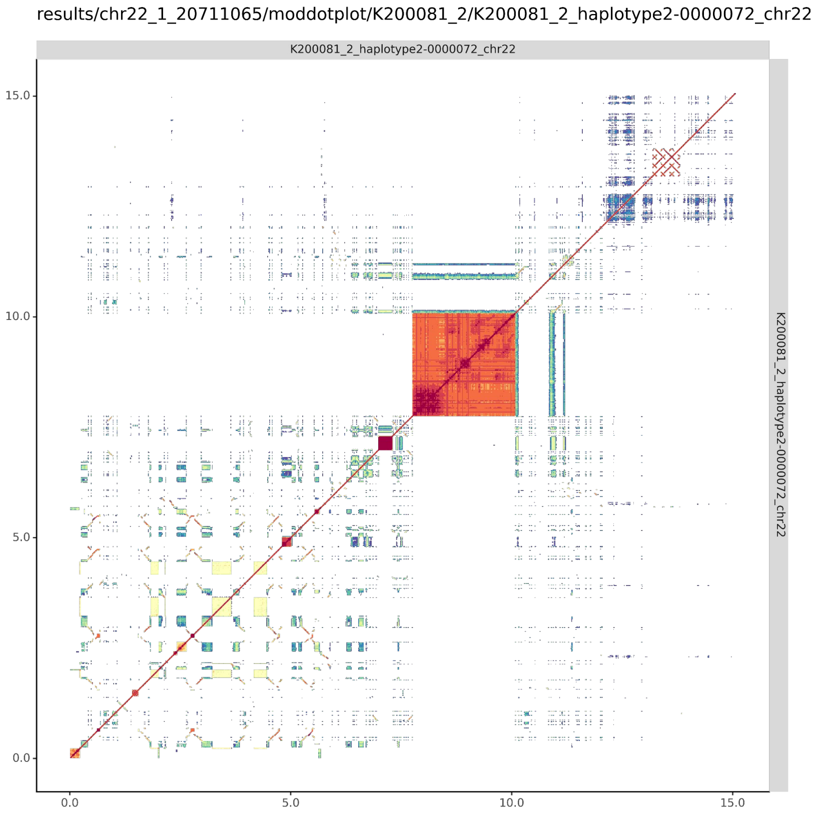

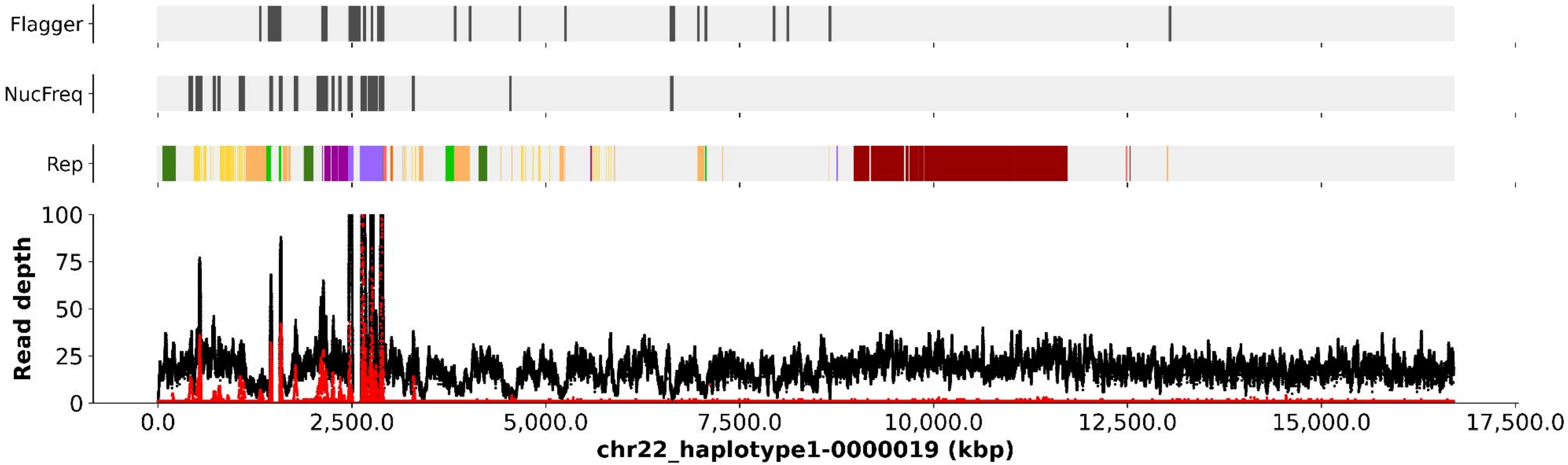

Flagger
NucFreq
Rep
Read depth
100
75
50
25
0
0.0
2,500.0
5,000.0
7,500.0
10,000.0
12,500.0
15,000.0
17,500.0
chr22_haplotype1-0000019 (kbp)

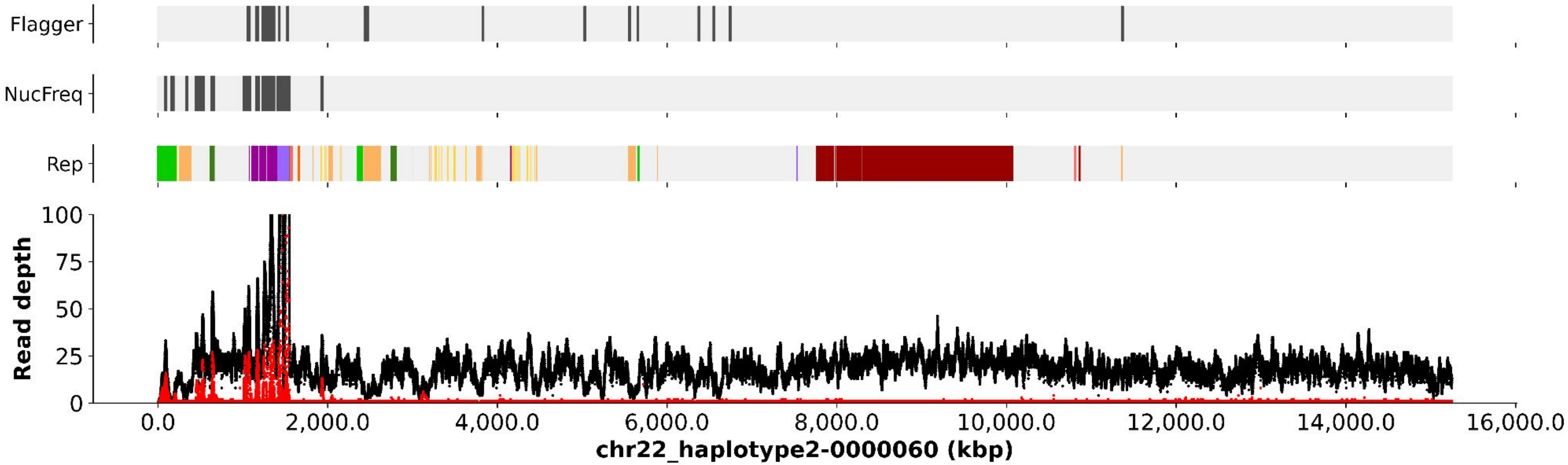

Flagger
NucFreq
Rep
Read depth
100
75
50
25
0
0.0
2,000.0
4,000.0
6,000.0
8,000.0
10,000.0
12,000.0
14,000.0
16,000.0
chr22_haplotype2-0000060 (kbp)

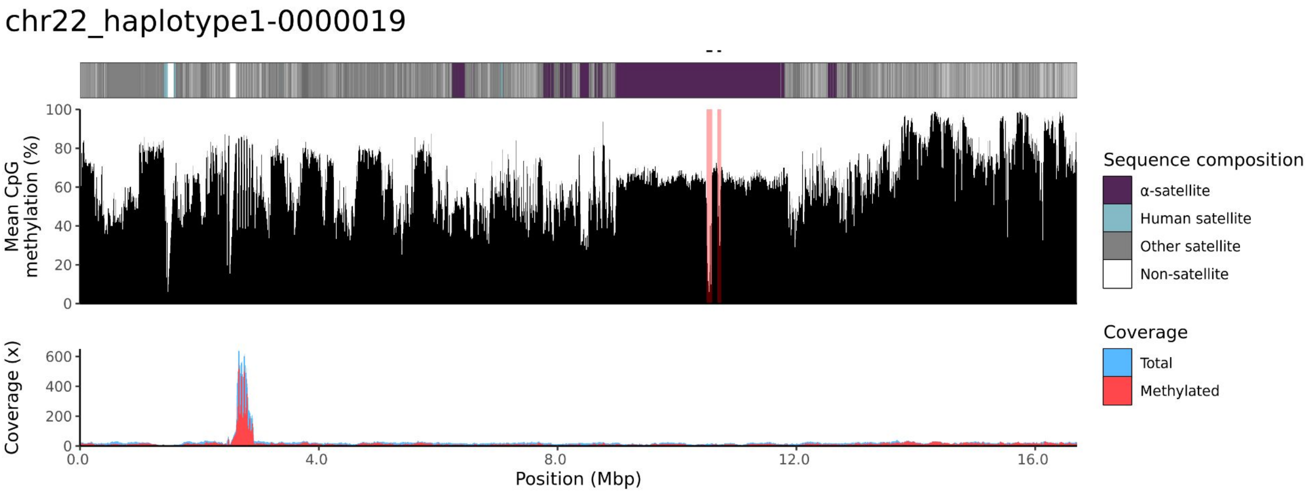
chr22_haplotype1-0000019
Mean CpG methylation (%)
100
80
60
40
20
0
Coverage (x)
600
400
200
0
0.0
4.0
8.0
12.0
16.0
Position (Mbp)
Sequence composition
α-satellite
Human satellite
Other satellite
Non-satellite
Coverage
Total
Methylated

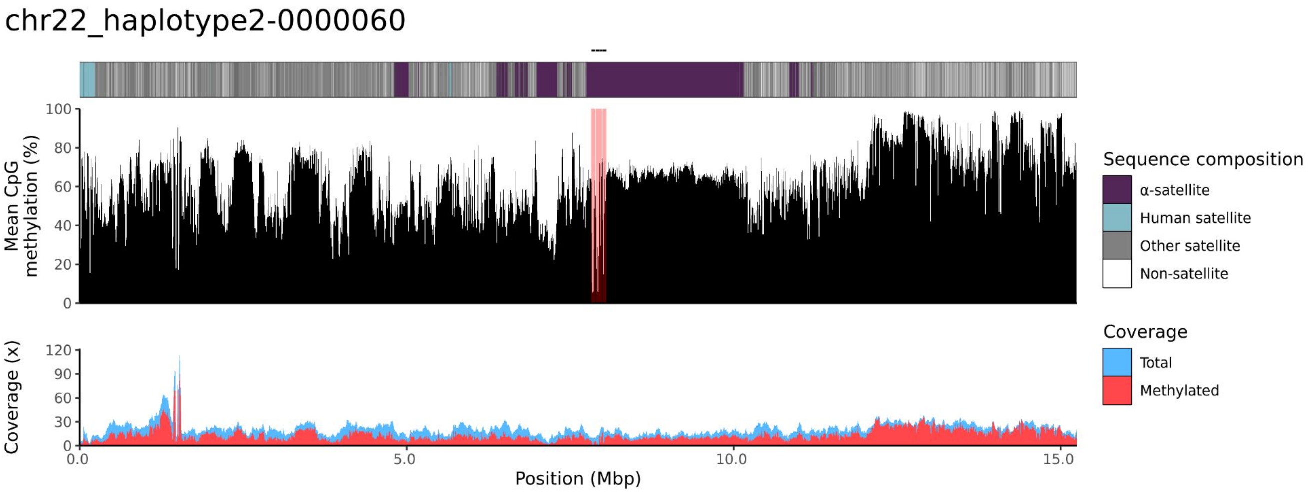
chr22_haplotype2-0000060
Mean CpG methylation (%)
100
80
60
40
20
0
Coverage (x)
120
90
60
30
0
0.0
5.0
10.0
15.0
Position (Mbp)
Sequence composition
α-satellite
Human satellite
Other satellite
Non-satellite
Coverage
Total
Methylated

200082

chr14

K200082_1_haplotype1-0000010_chr14

results/chr14_1_17708411/moddotplot/K200082_1/K200082_1_haplotype1-0000010_chr14

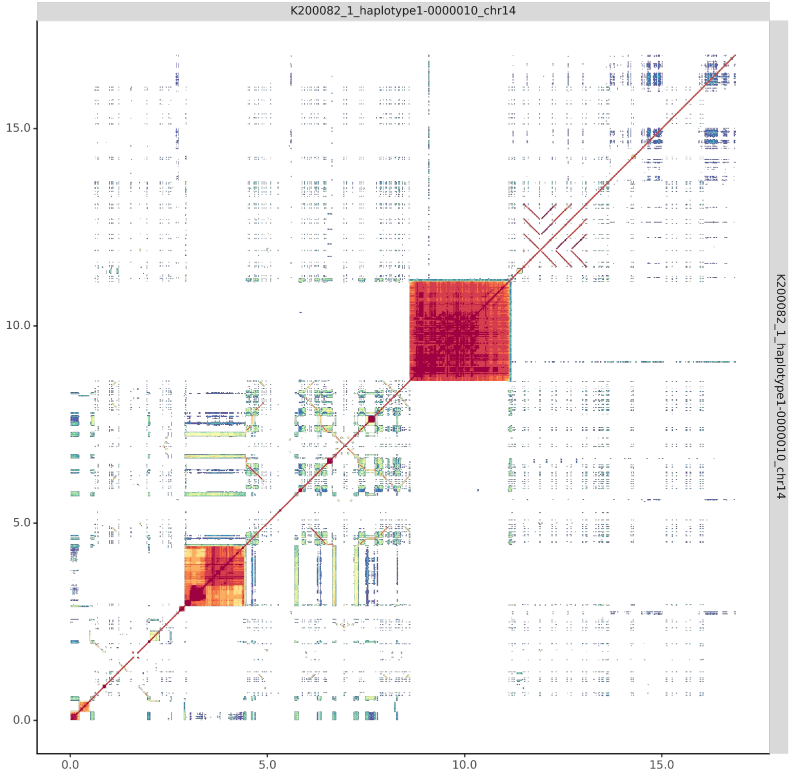

K200082_2_haplotype2-0000128_chr14

results/chr14_1_17708411/moddotplot/K200082_2/K200082_2_haplotype2-0000128_chr14

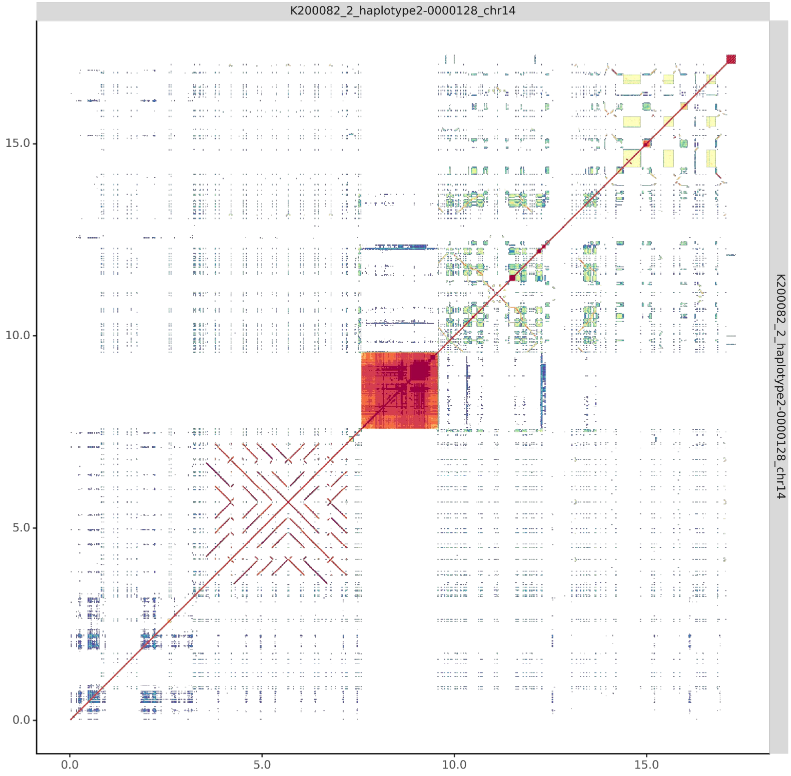

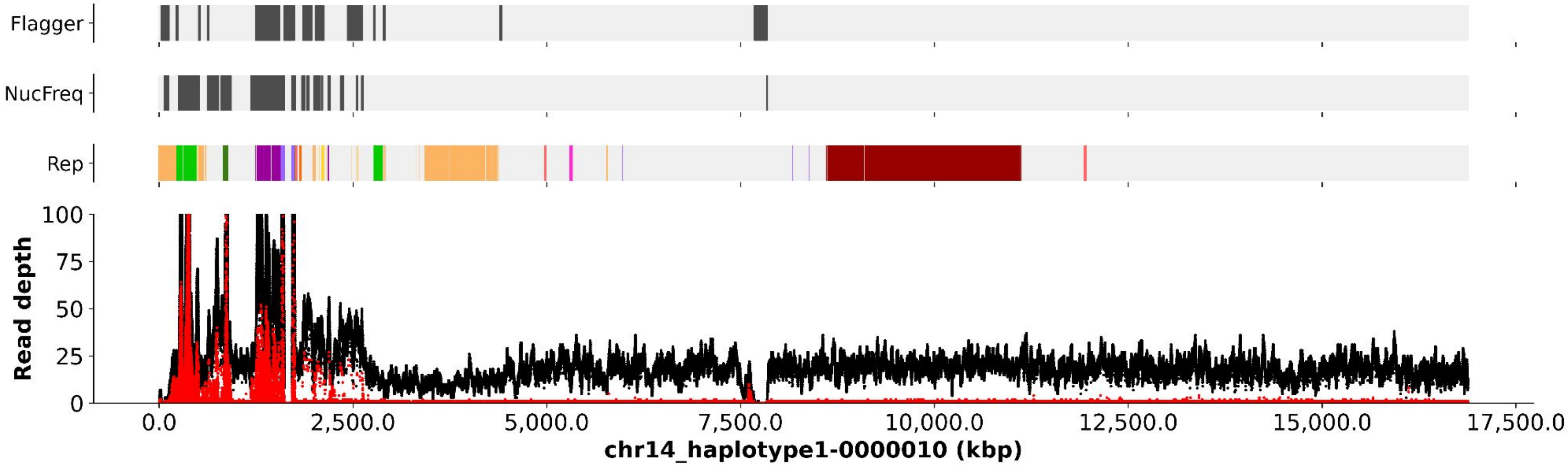

Flagger
NucFreq
Rep
Read depth
100
75
50
25
0
0.0
2,500.0
5,000.0
7,500.0
10,000.0
12,500.0
15,000.0
17,500.0
chr14_haplotype1-0000010 (kbp)

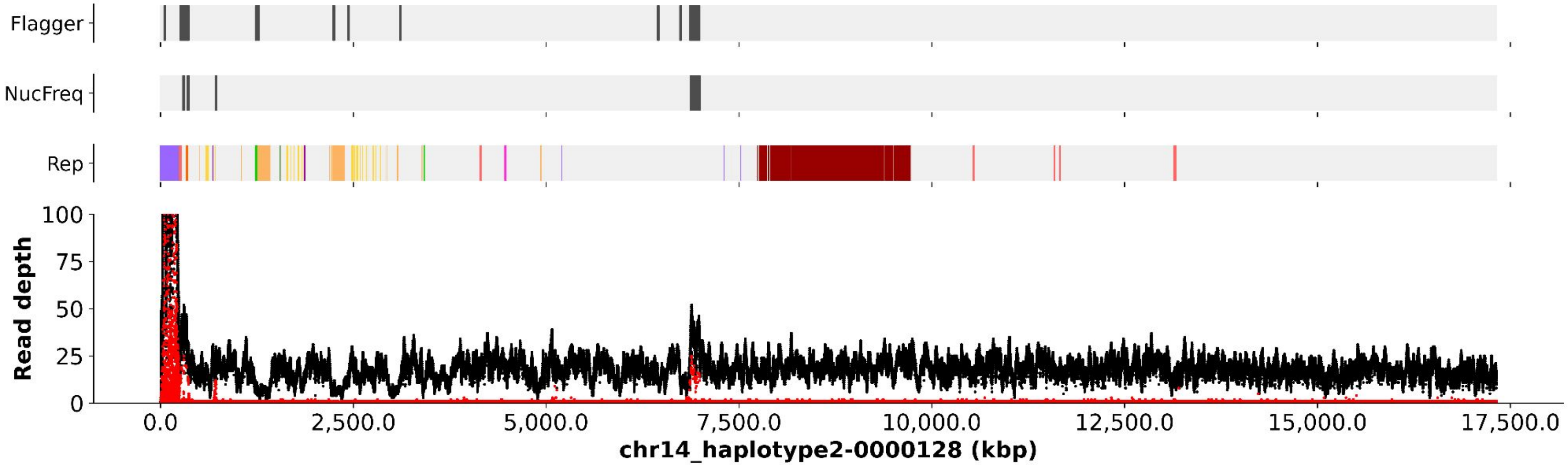

Flagger
NucFreq
Rep
Read depth
100
75
50
25
0
0.0
2,500.0
5,000.0
7,500.0
10,000.0
12,500.0
15,000.0
17,500.0
chr14_haplotype2-0000128 (kbp)

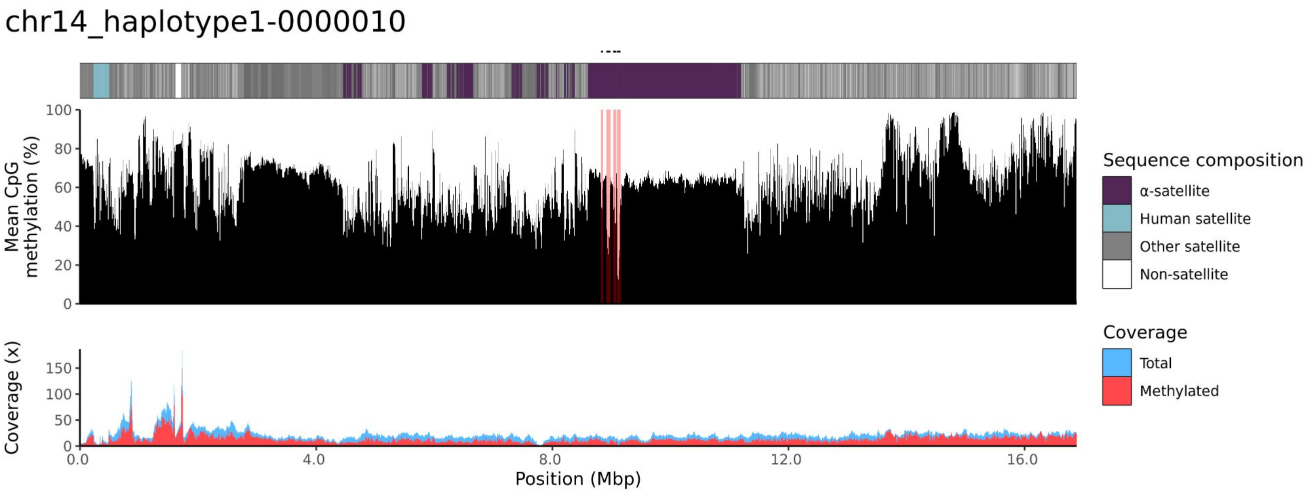
chr14_haplotype1-0000010
Mean CpG methylation (%)
100
80
60
40
20
0
Coverage (x)
150
100
50
0
0.0
4.0
8.0
12.0
16.0
Position (Mbp)
Sequence composition
α-satellite
Human satellite
Other satellite
Non-satellite
Coverage
Total
Methylated

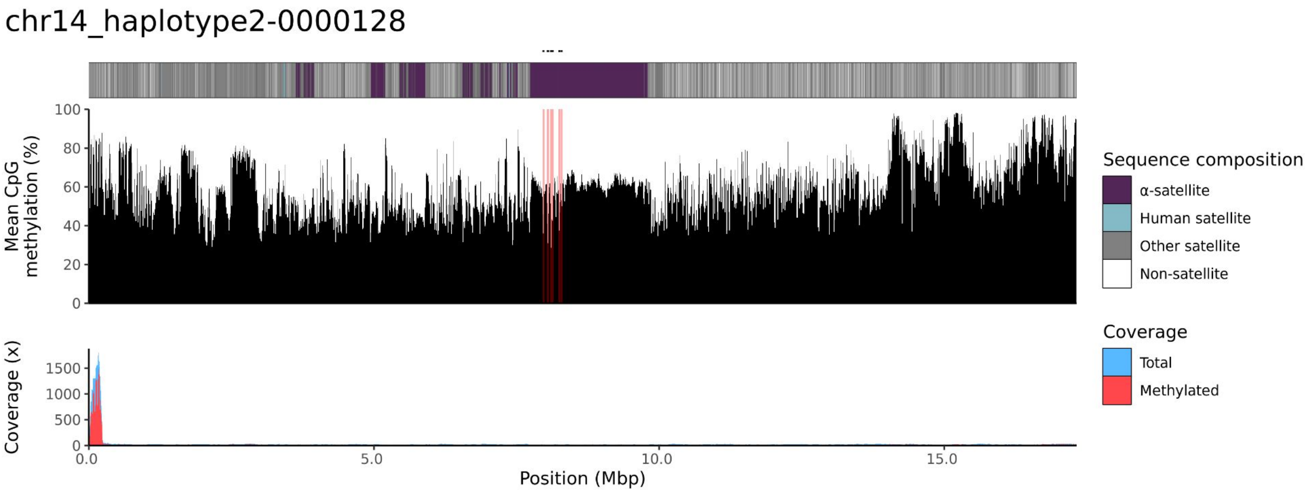
chr14_haplotype2-0000128
Mean CpG methylation (%)
100
80
60
40
20
0
Coverage (x)
1500
1000
500
0
0.0
5.0
10.0
15.0
Position (Mbp)
Sequence composition
α-satellite
Human satellite
Other satellite
Non-satellite
Coverage
Total
Methylated

chr15

K200082_1_haplotype1-0000028_chr15

results/chr15_1_22694466/moddotplot/K200082_1/K200082_1_haplotype1-0000028_chr15

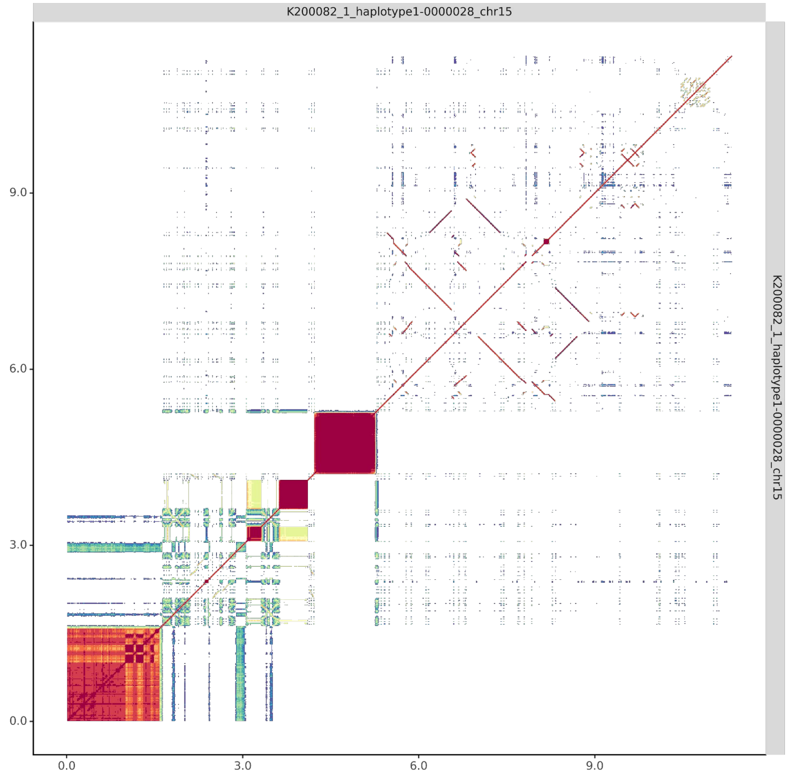

K200082_2_haplotype2-0000146_chr15

results/chr15_1_22694466/moddotplot/K200082_2/K200082_2_haplotype2-0000146_chr15

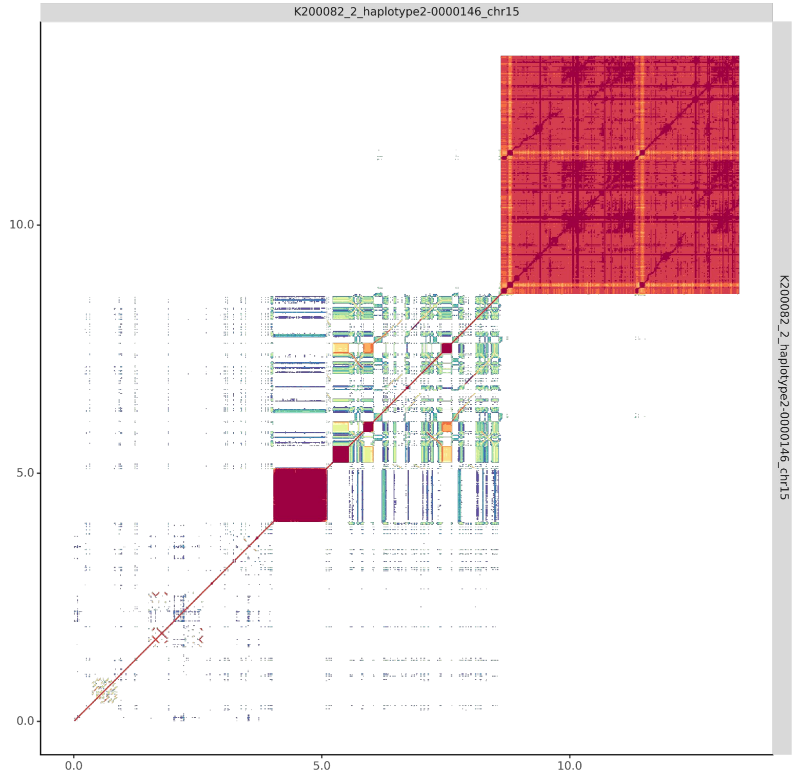

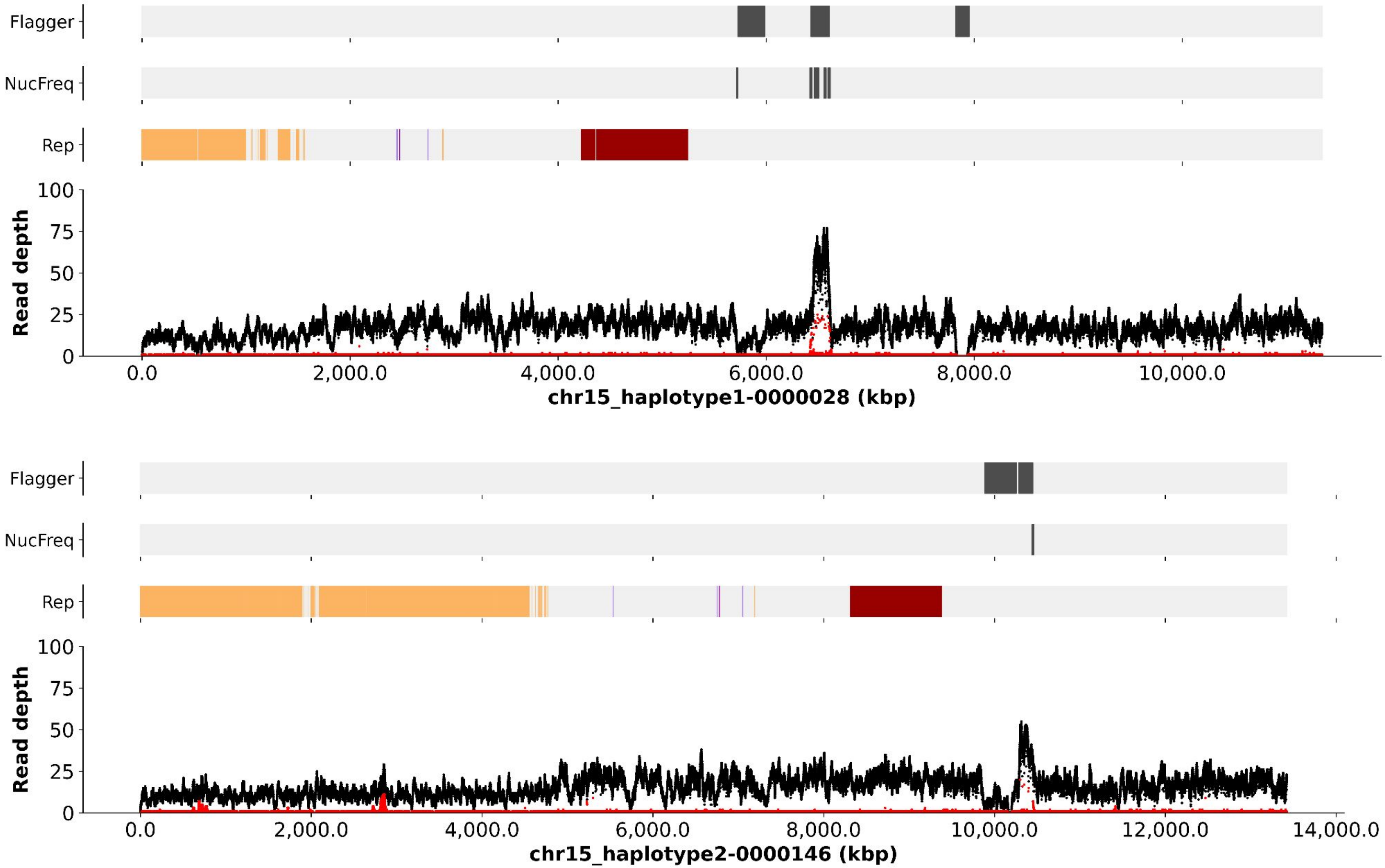
Flagger
NucFreq
Rep
Read depth
100
75
50
25
0
0.0
2,000.0
4,000.0
6,000.0
8,000.0
10,000.0
chr15_haplotype1-0000028 (kbp)
Flagger
NucFreq
Rep
Read depth
100
75
50
25
0
0.0
2,000.0
4,000.0
6,000.0
8,000.0
10,000.0
12,000.0
14,000.0
chr15_haplotype2-0000146 (kbp)

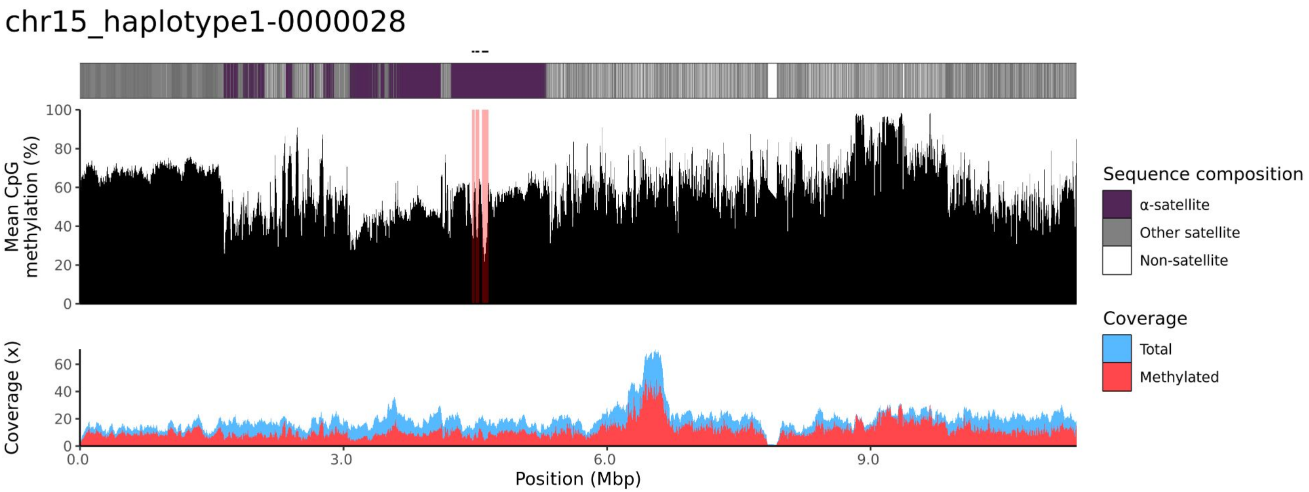

chr15_haplotype1-0000028
Mean CpG methylation (%)
100
80
60
40
20
0
Coverage (x)
60
40
20
0
0.0
3.0
6.0
9.0
Position (Mbp)
Sequence composition
α-satellite
Other satellite
Non-satellite
Coverage
Total
Methylated

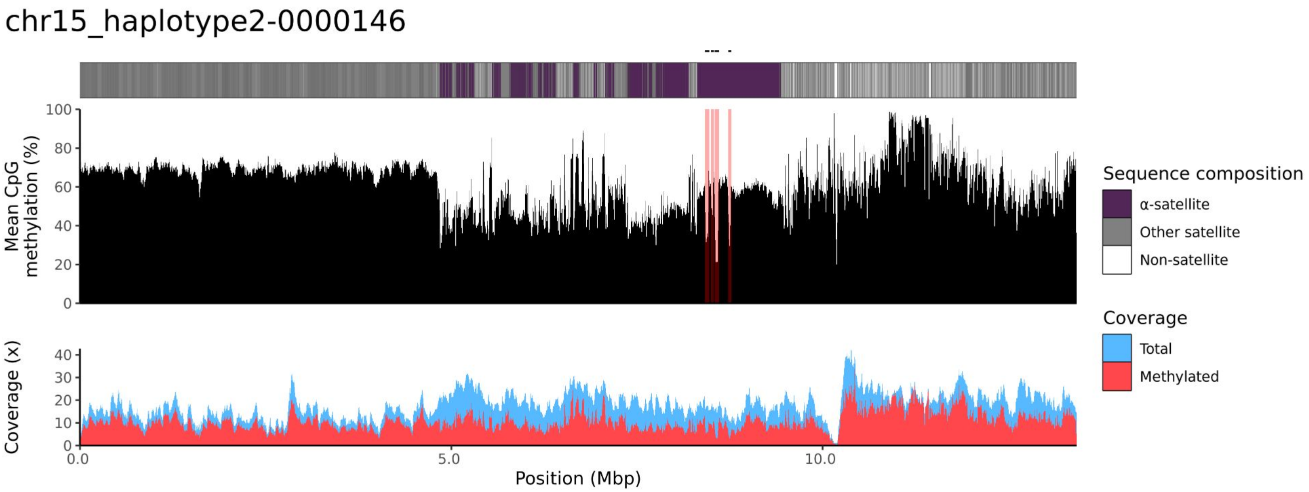

chr15_haplotype2-0000146
Mean CpG methylation (%)
100
80
60
40
20
0
Coverage (x)
40
30
20
10
0
0.0
5.0
10.0
Position (Mbp)
Sequence composition
α-satellite
Other satellite
Non-satellite
Coverage
Total
Methylated

chr21

K200082_1_haplotype1-0000020_chr21

results/chr21_1_16306378/moddotplot/K200082_1/K200082_1_haplotype1-0000020_chr21

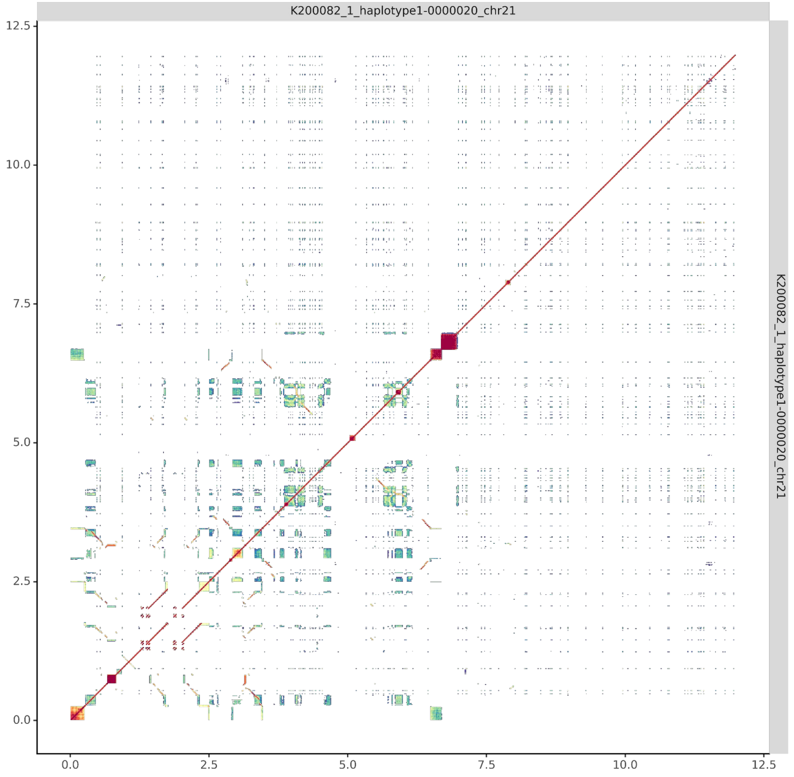

K200082_2_haplotype2-0000136_chr21

results/chr21_1_16306378/moddotplot/K200082_2/K200082_2_haplotype2-0000136_chr21

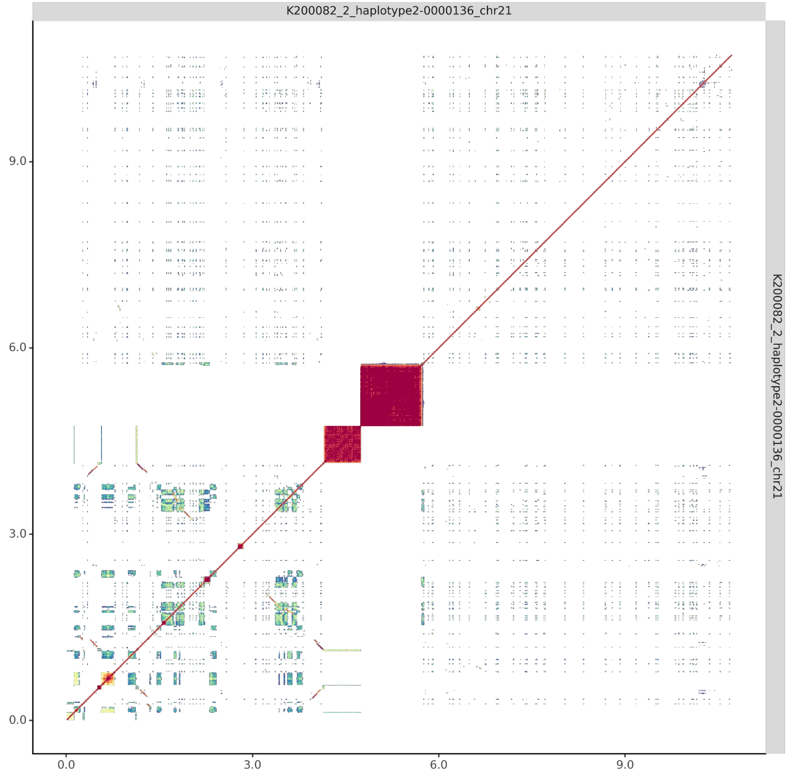

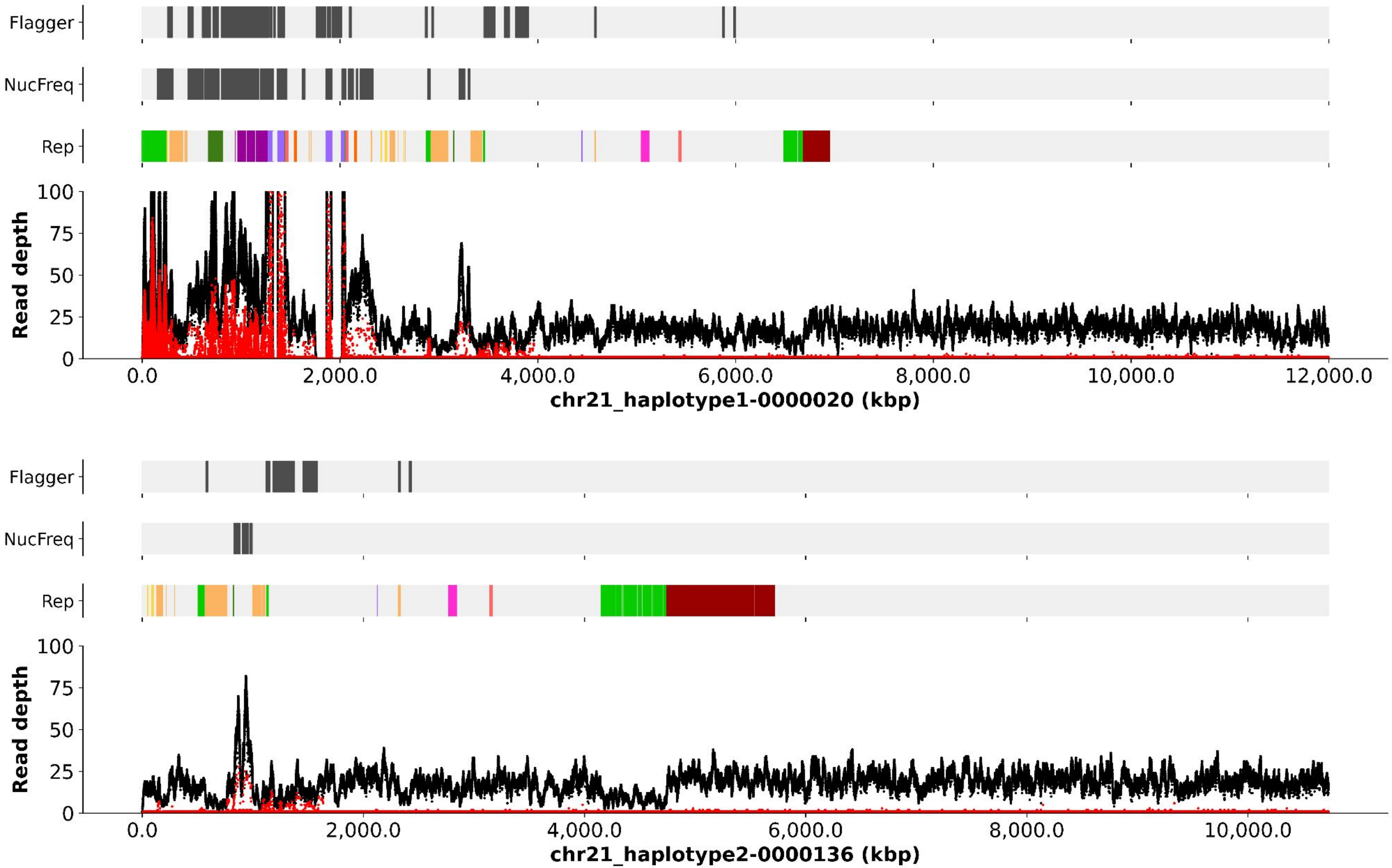

Flagger
NucFreq
Rep
Read depth
100
75
50
25
0
0.0
2,000.0
4,000.0
6,000.0
8,000.0
10,000.0
12,000.0
chr21_haplotype1-0000020 (kbp)
Flagger
NucFreq
Rep
Read depth
100
75
50
25
0
0.0
2,000.0
4,000.0
6,000.0
8,000.0
10,000.0
chr21_haplotype2-0000136 (kbp)

chr21_haplotype1-0000020

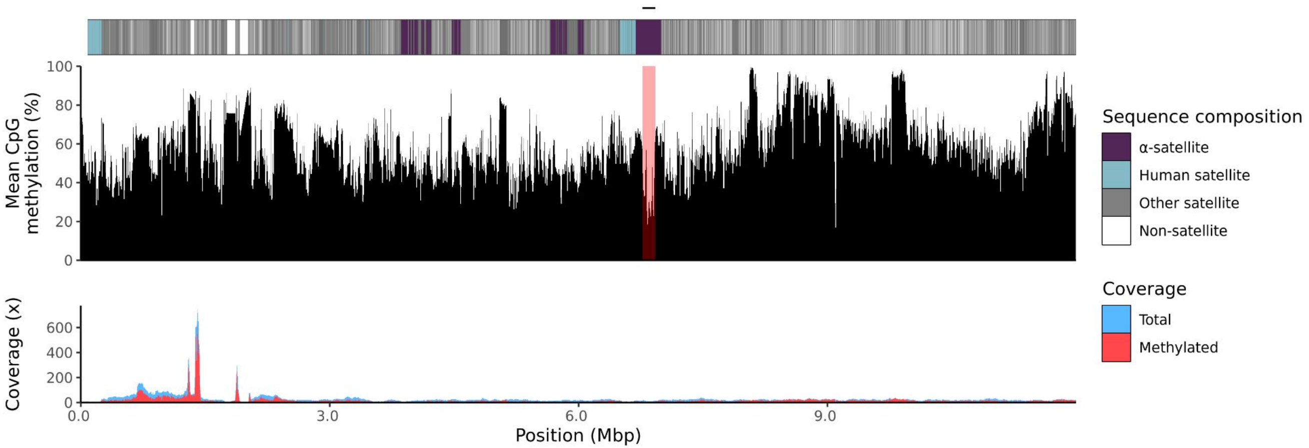

chr21_haplotype2-0000136

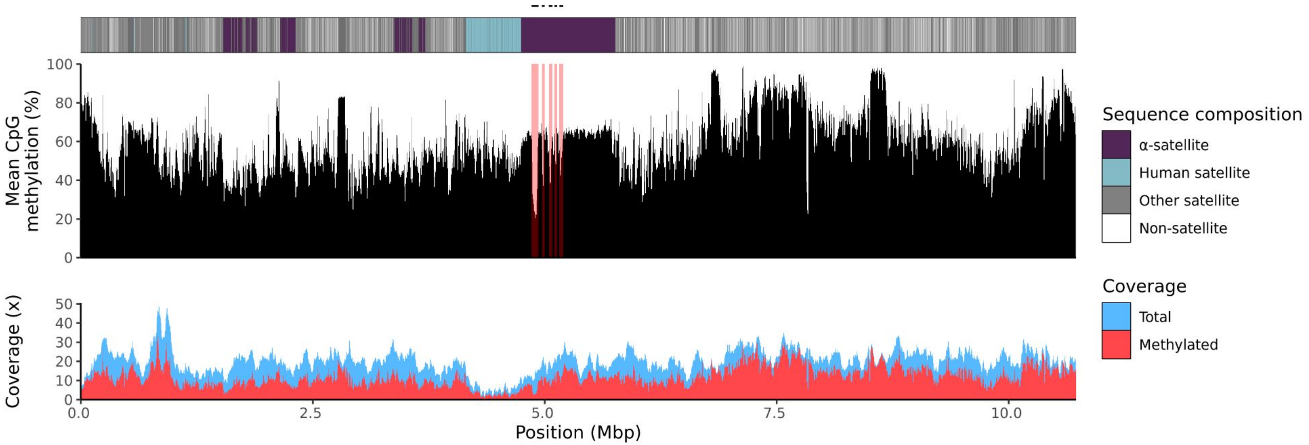

chr22

K200082_1_haplotype1-0000003_chr22

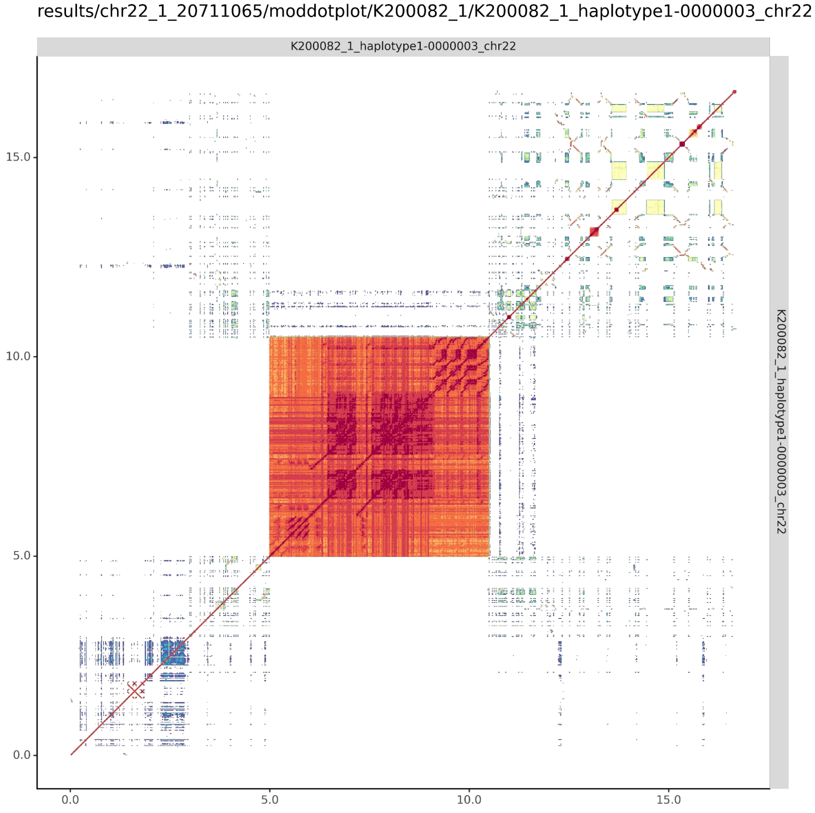

K200082_2_haplotype2-0000121_chr22

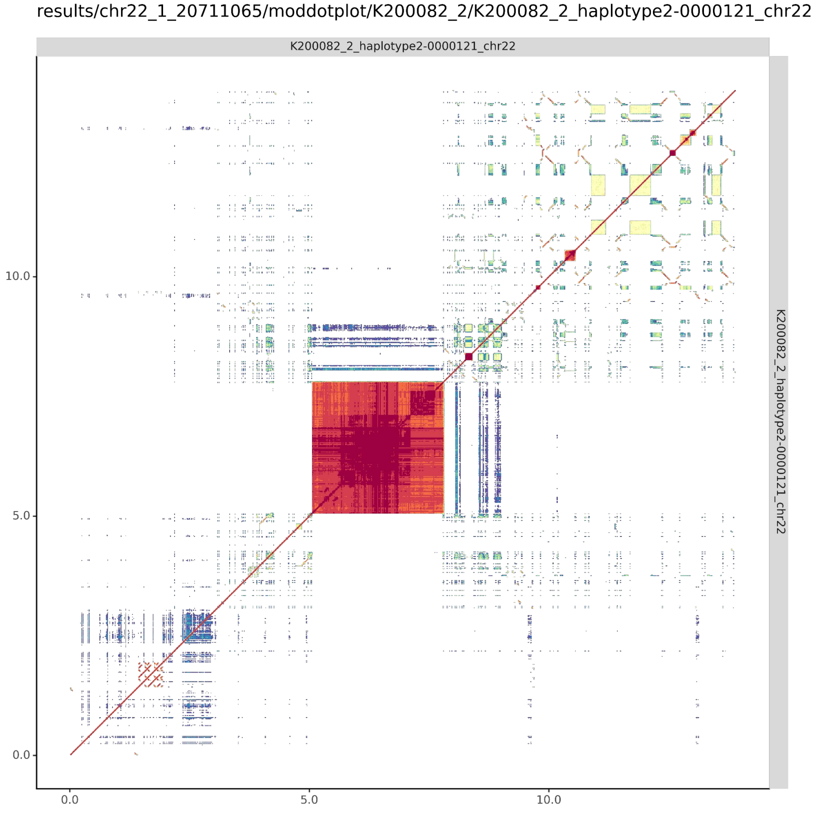

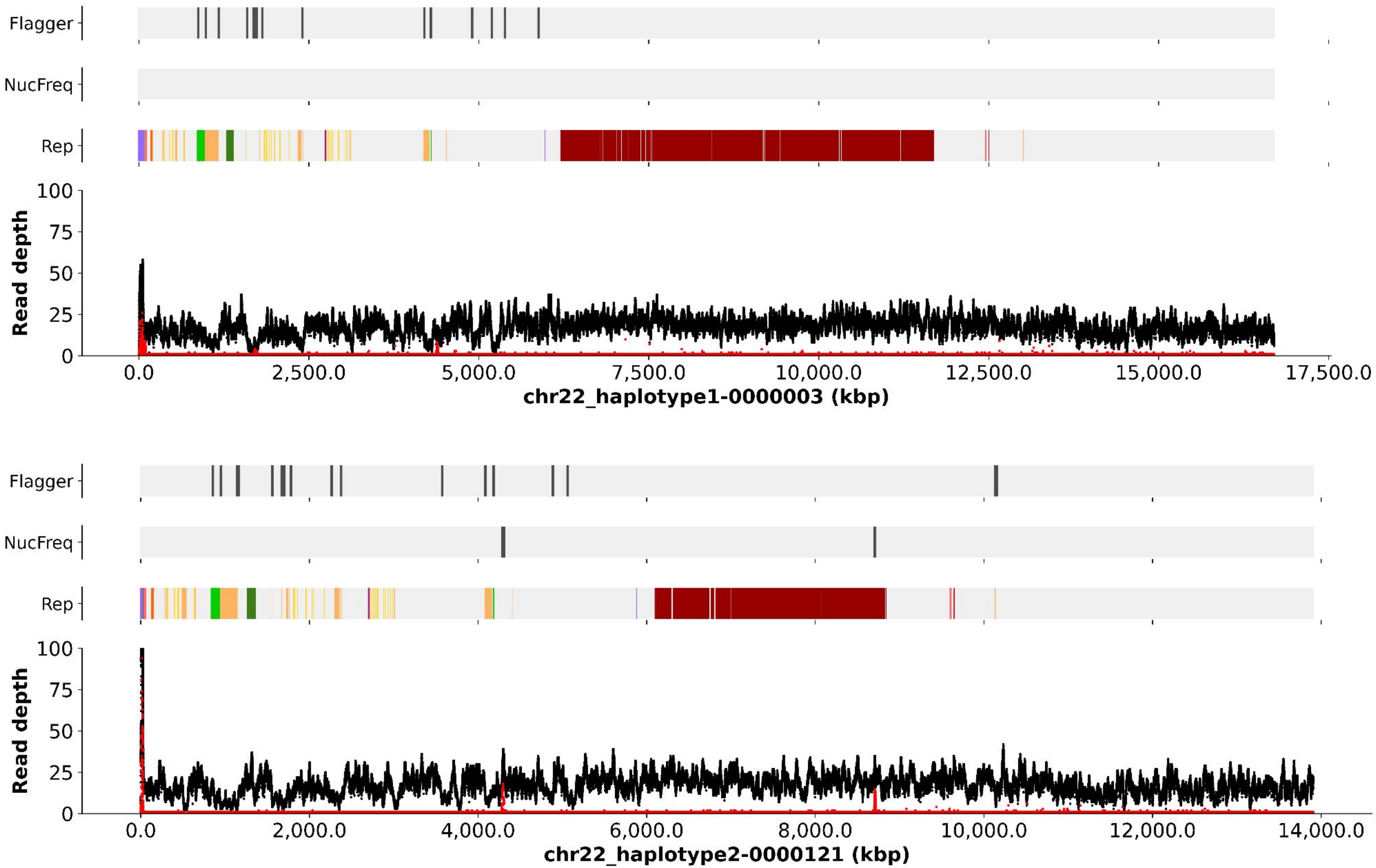
Flagger
NucFreq
Rep
Read depth
100
75
50
25
0
0.0
2,500.0
5,000.0
7,500.0
10,000.0
12,500.0
15,000.0
17,500.0
chr22_haplotype1-0000003 (kbp)
Flagger
NucFreq
Rep
Read depth
100
75
50
25
0
0.0
2,000.0
4,000.0
6,000.0
8,000.0
10,000.0
12,000.0
14,000.0
chr22_haplotype2-0000121 (kbp)

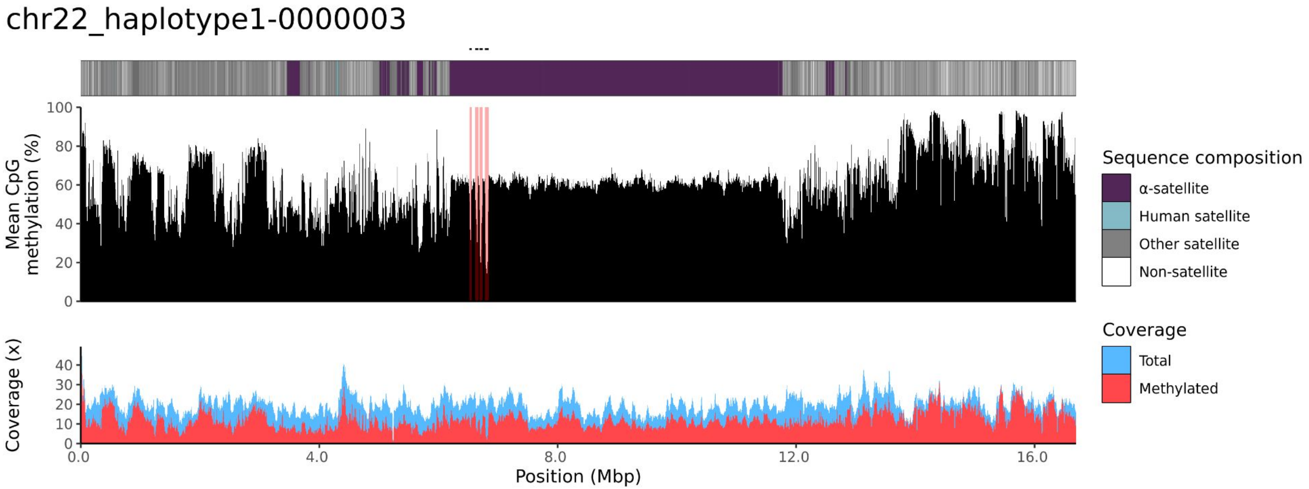
chr22_haplotype1-0000003
Mean CpG methylation (%)
100
80
60
40
20
0
Coverage (x)
40
30
20
10
0
0.0
4.0
8.0
12.0
16.0
Position (Mbp)
Sequence composition
α-satellite
Human satellite
Other satellite
Non-satellite
Coverage
Total
Methylated

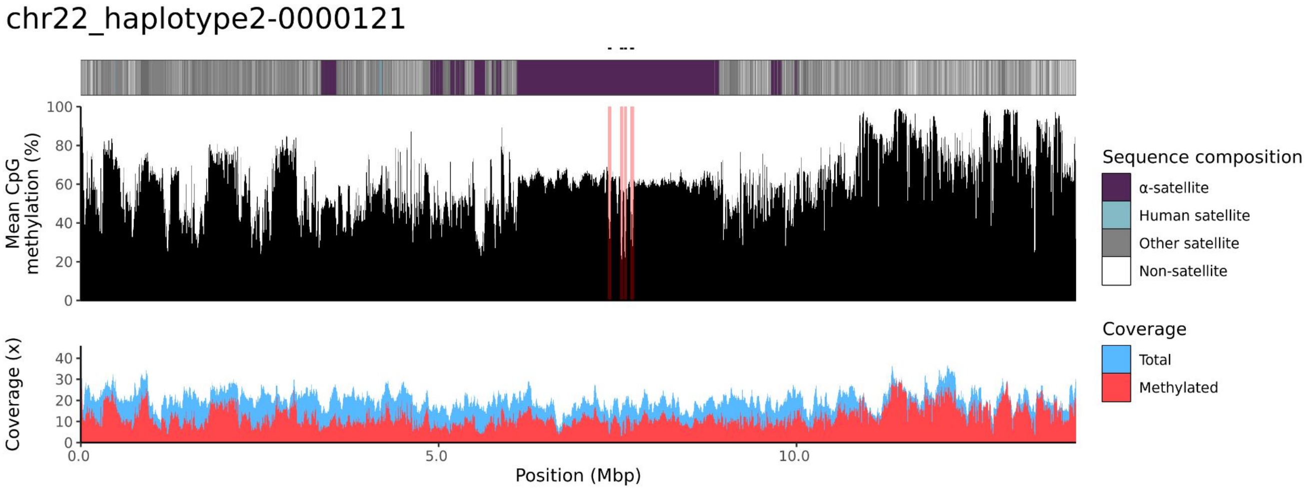
chr22_haplotype2-0000121
Mean CpG methylation (%)
100
80
60
40
20
0
Coverage (x)
40
30
20
10
0
0.0
5.0
10.0
Position (Mbp)
Sequence composition
α-satellite
Human satellite
Other satellite
Non-satellite
Coverage
Total
Methylated

200084

chr13

K200084_1_haplotype1-0000019_chr13

K200084_2_haplotype2-0000083_chr13

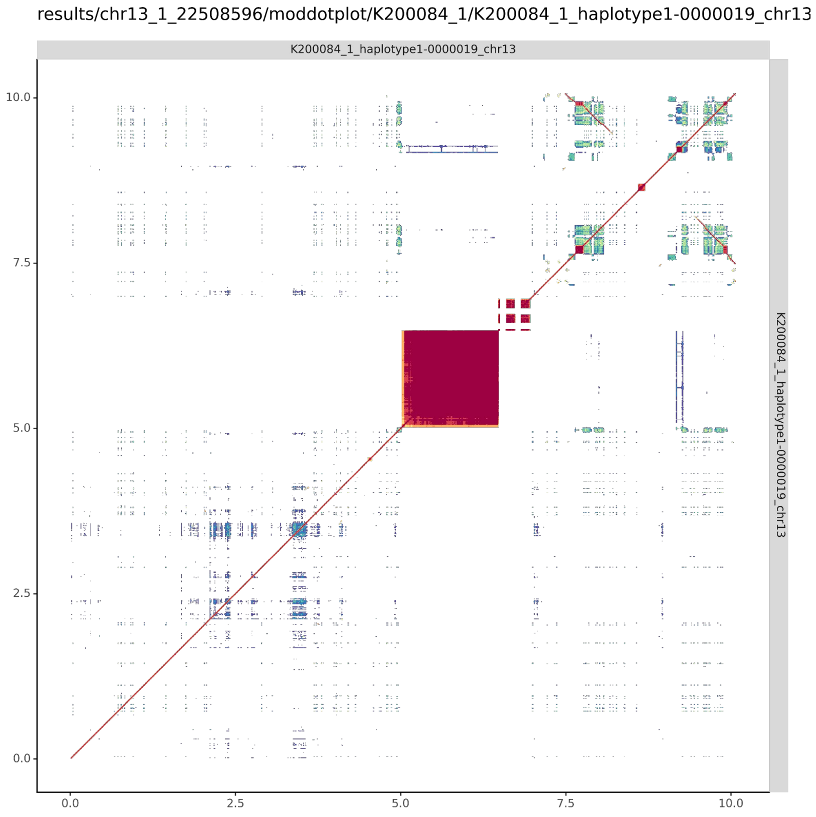

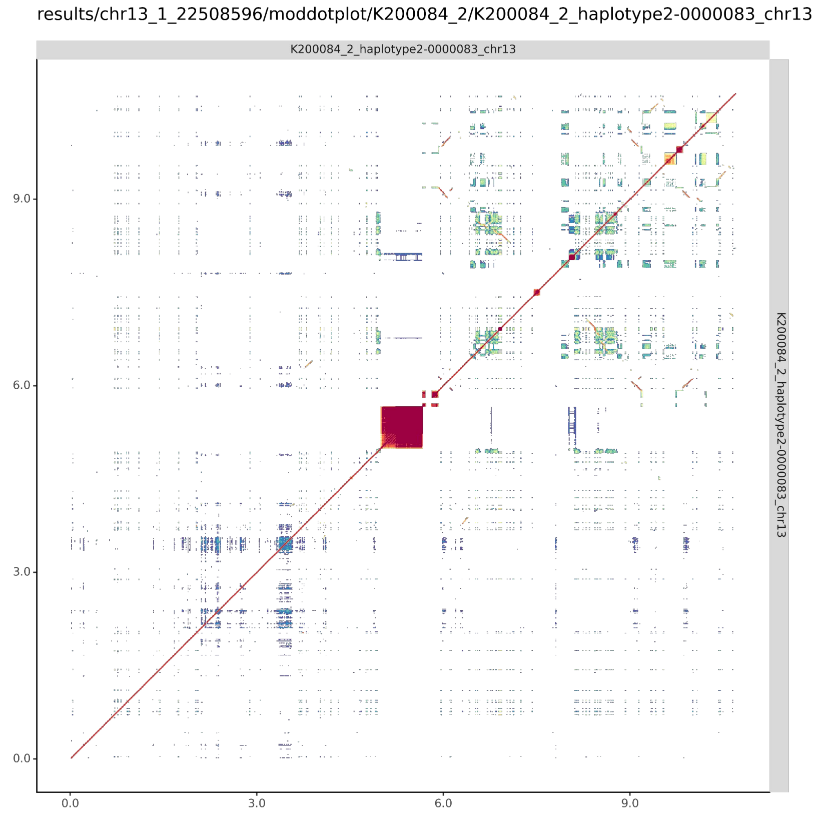

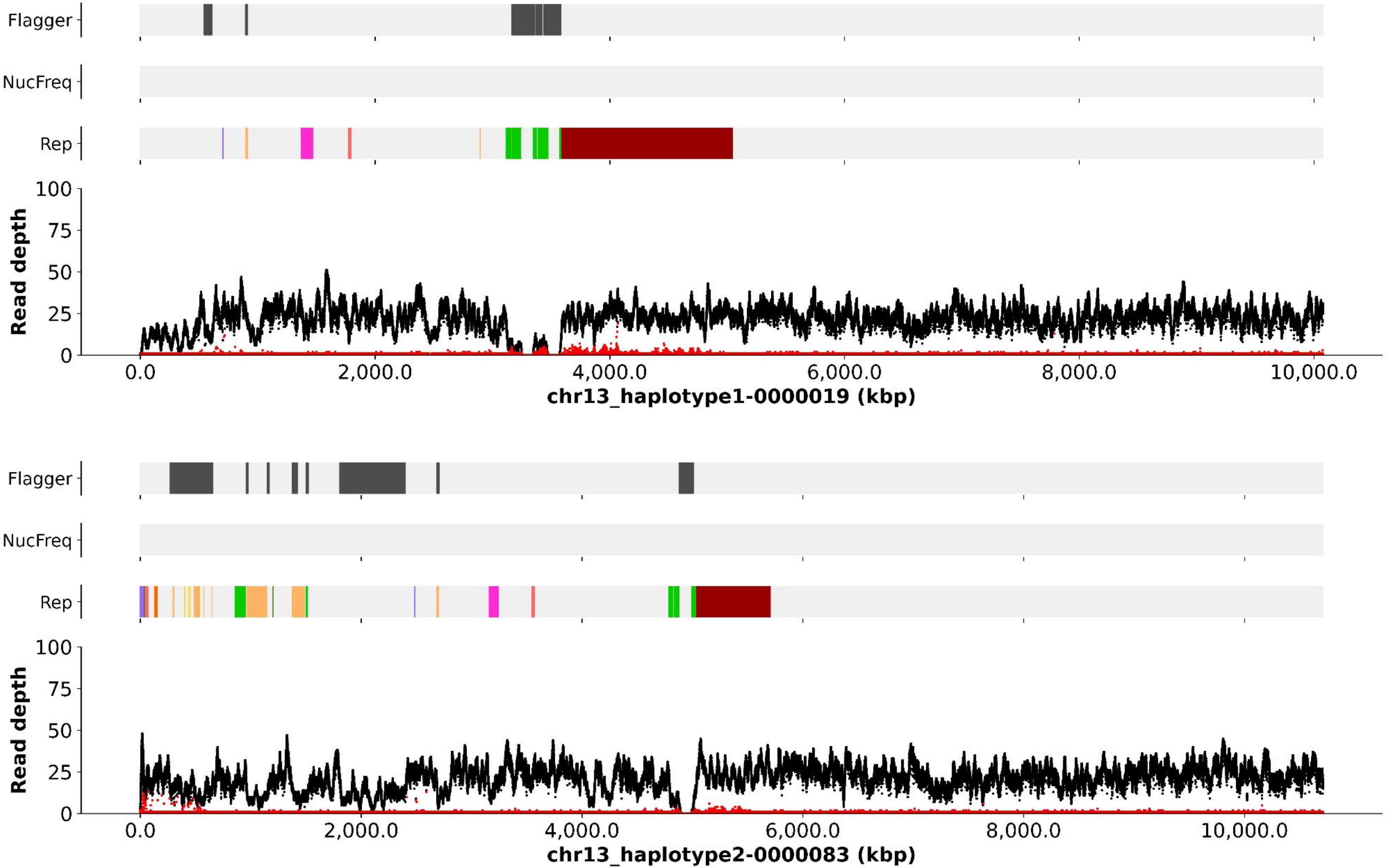
Flagger
NucFreq
Rep
Read depth
100
75
50
25
0
0.0
2,000.0
4,000.0
6,000.0
8,000.0
10,000.0
chr13_haplotype1-0000019 (kbp)
Flagger
NucFreq
Rep
Read depth
100
75
50
25
0
0.0
2,000.0
4,000.0
6,000.0
8,000.0
10,000.0
chr13_haplotype2-0000083 (kbp)

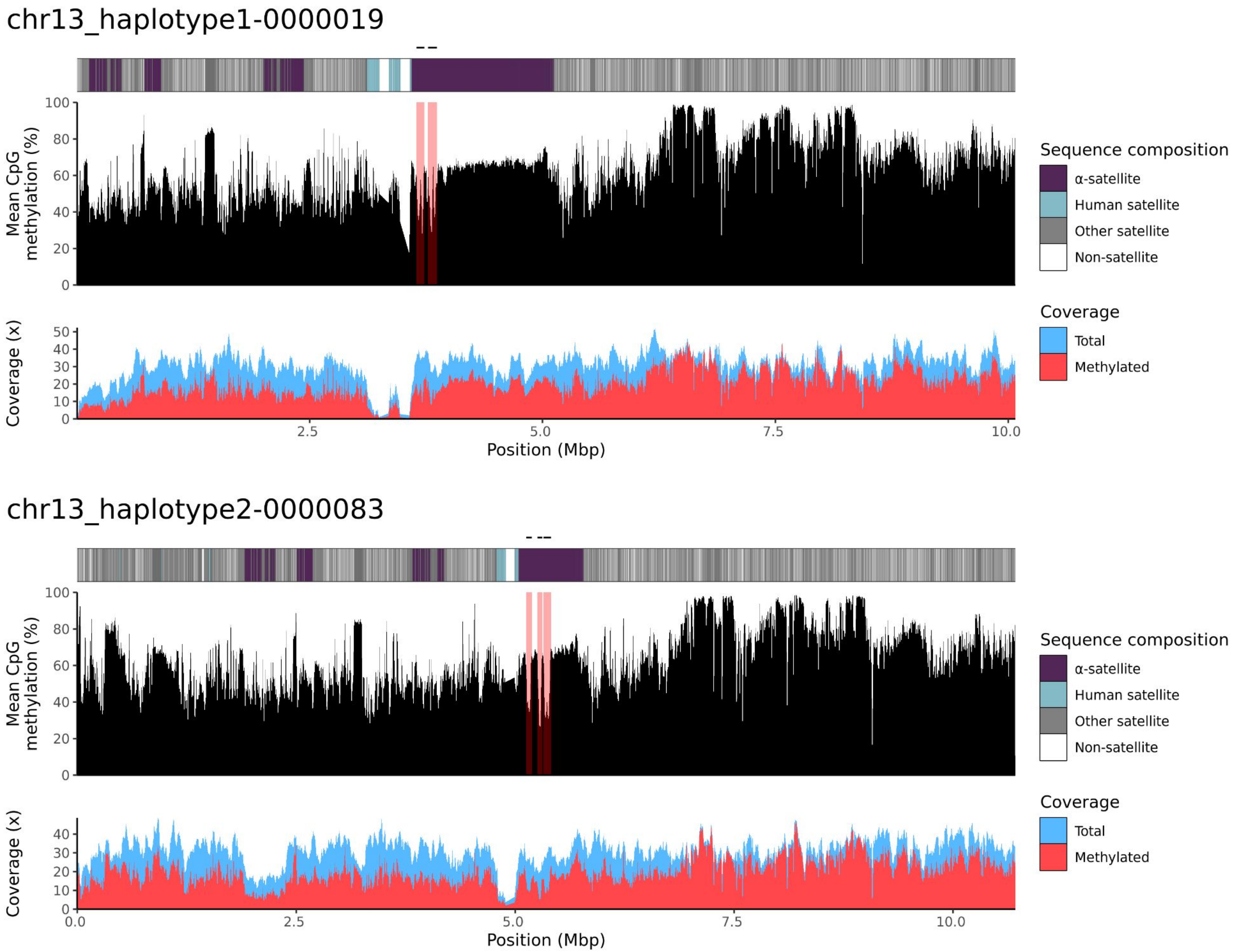
chr13_haplotype1-0000019
Mean CpG methylation (%)
Coverage (x)
Position (Mbp)
Sequence composition
α-satellite
Human satellite
Other satellite
Non-satellite
Coverage
Total
Methylated
chr13_haplotype2-0000083
Mean CpG methylation (%)
Coverage (x)
Position (Mbp)
Sequence composition
α-satellite
Human satellite
Other satellite
Non-satellite
Coverage
Total
Methylated

chr14

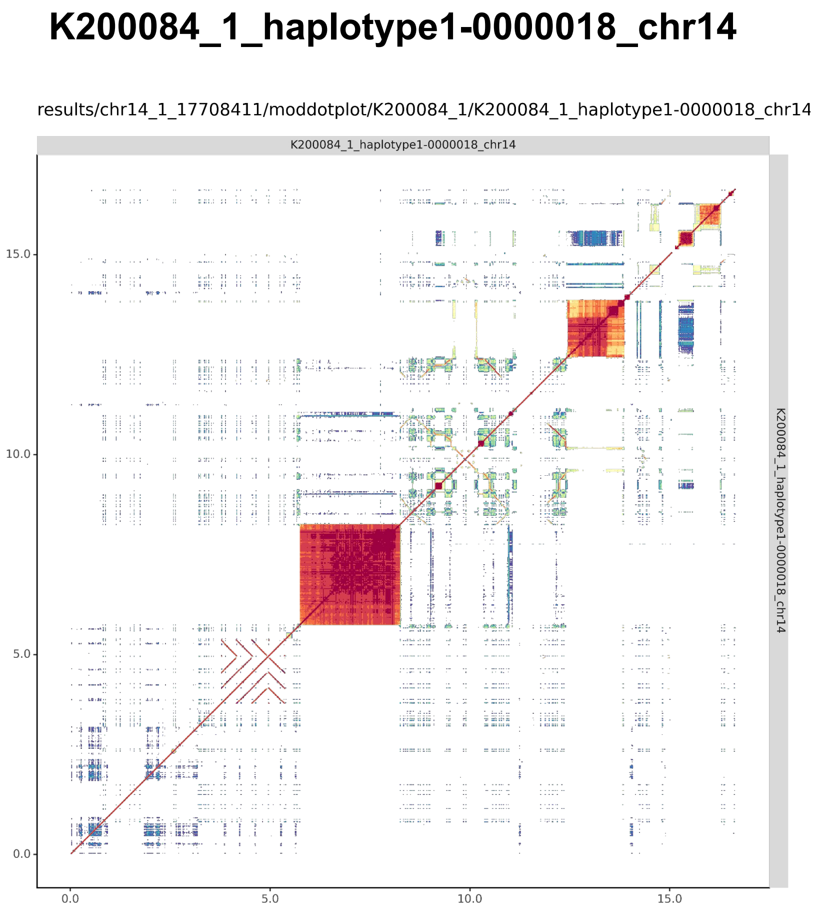
K200084_1_haplotype1-0000018_chr14
results/chr14_1_17708411/moddotplot/K200084_1/K200084_1_haplotype1-0000018_chr14
K200084_1_haplotype1-0000018_chr14
15.0
10.0
5.0
0.0
0.0
5.0
10.0
15.0
K200084_1_haplotype1-0000018_chr14

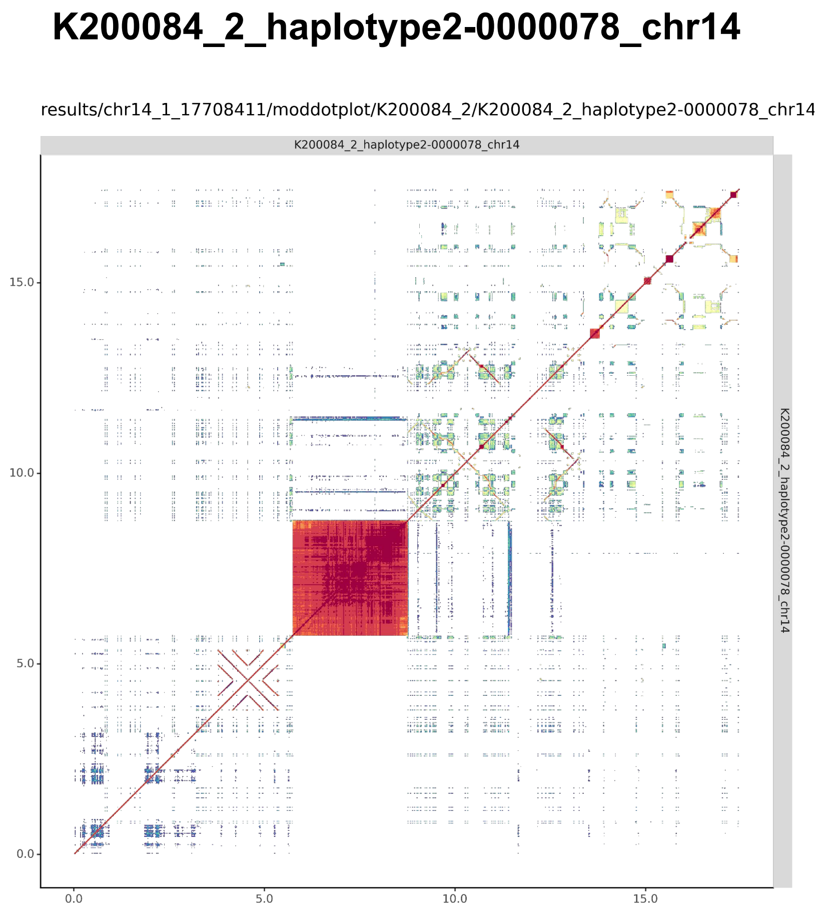
K200084_2_haplotype2-0000078_chr14
results/chr14_1_17708411/moddotplot/K200084_2/K200084_2_haplotype2-0000078_chr14
K200084_2_haplotype2-0000078_chr14
15.0
10.0
5.0
0.0
0.0
5.0
10.0
15.0
K200084_2_haplotype2-0000078_chr14

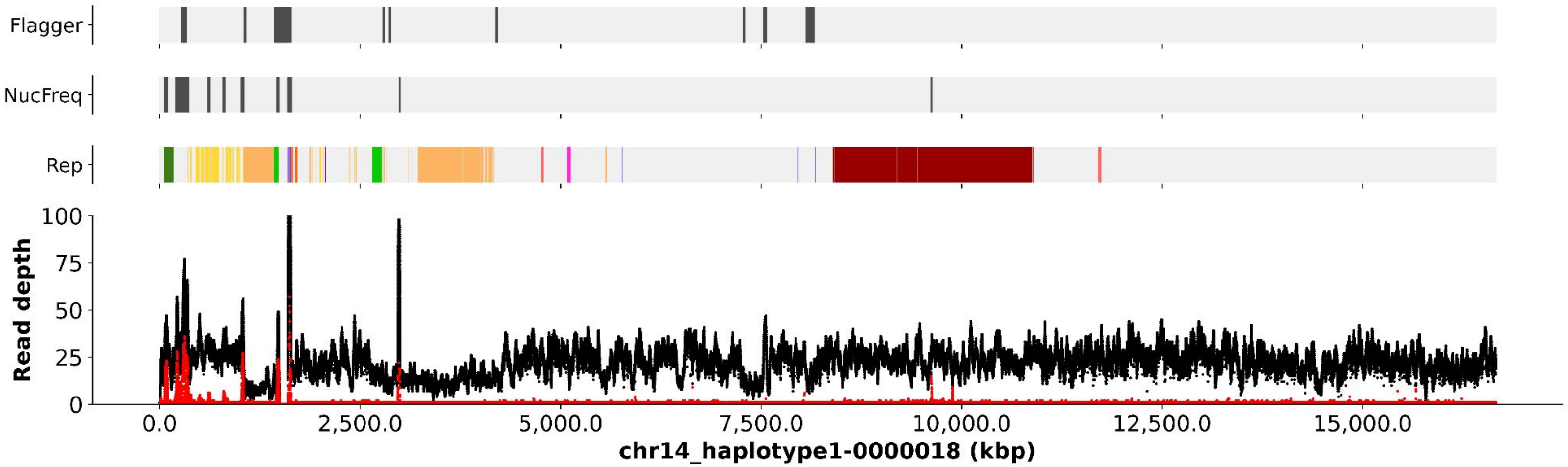
Flagger
NucFreq
Rep
100
75
50
25
0
Read depth
0.0
2,500.0
5,000.0
7,500.0
10,000.0
12,500.0
15,000.0
chr14_haplotype1-0000018 (kbp)

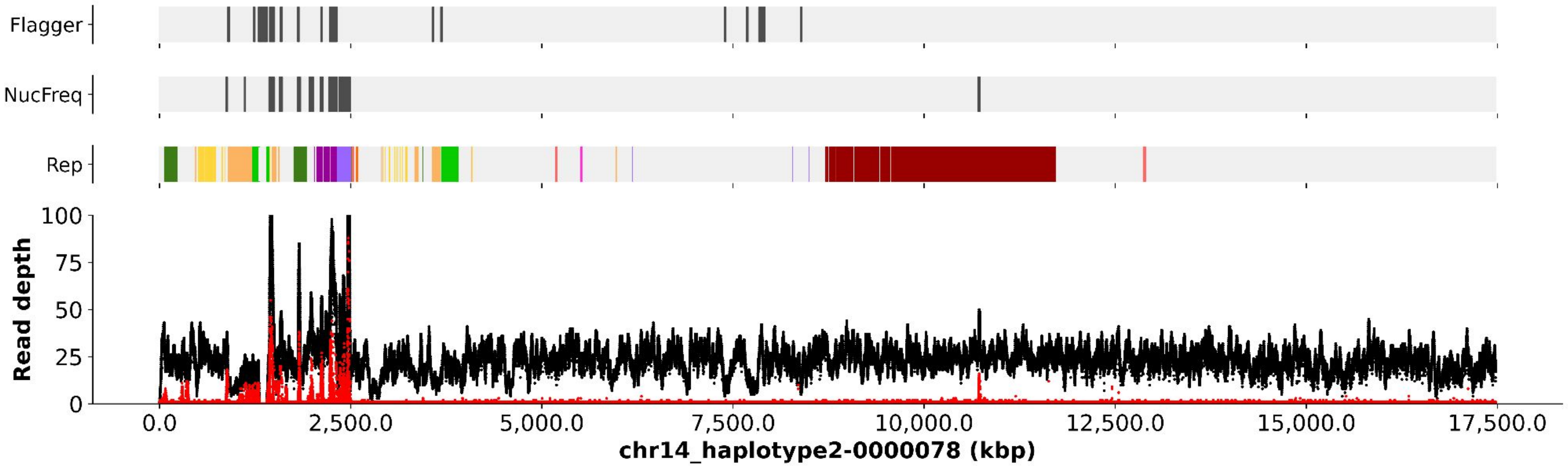
Flagger
NucFreq
Rep
100
75
50
25
0
Read depth
0.0
2,500.0
5,000.0
7,500.0
10,000.0
12,500.0
15,000.0
17,500.0
chr14_haplotype2-0000078 (kbp)

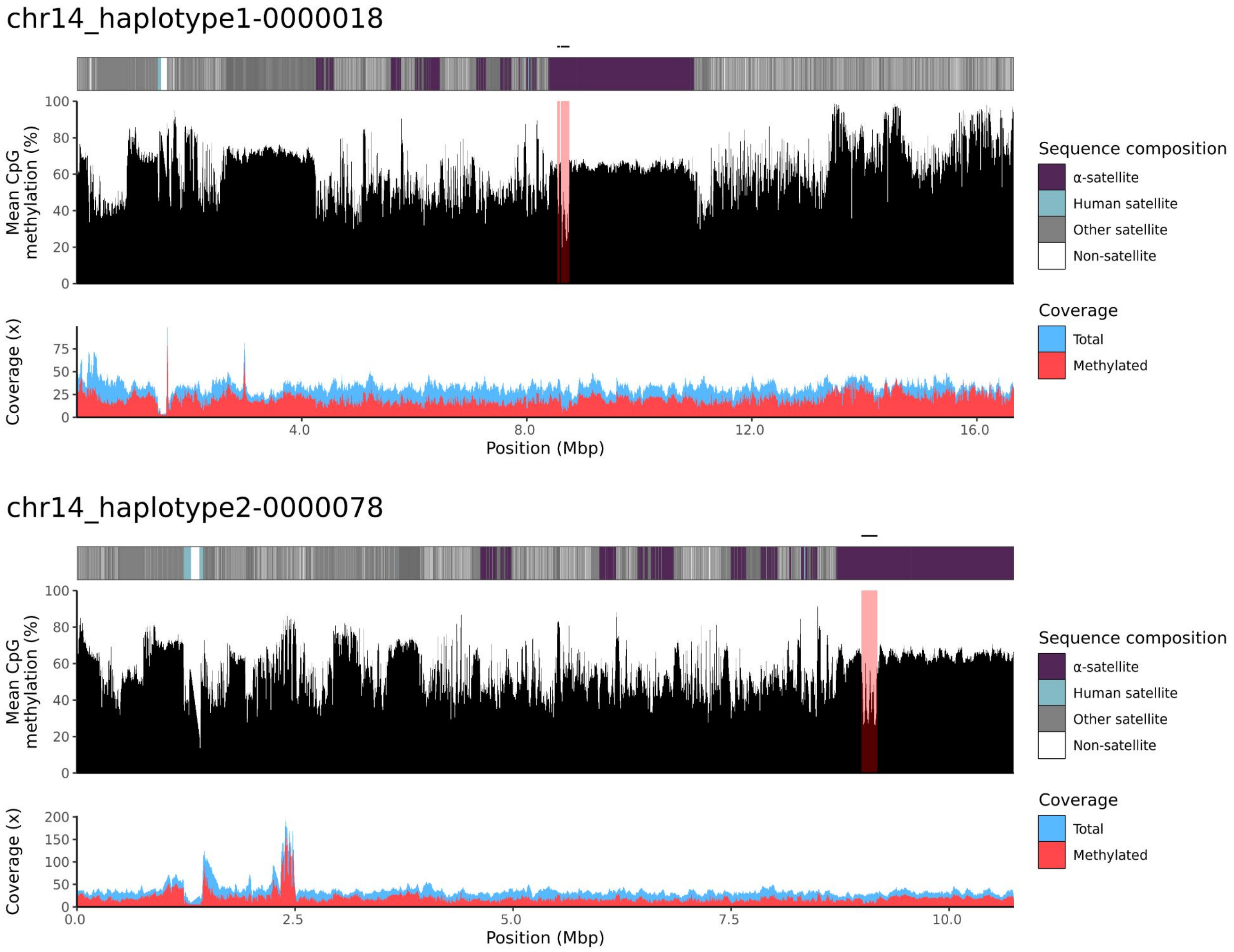

chr14_haplotype1-0000018
Mean CpG methylation (%)
100
80
60
40
20
0
Coverage (x)
75
50
25
0
4.0
8.0
12.0
16.0
Position (Mbp)
Sequence composition
α-satellite
Human satellite
Other satellite
Non-satellite
Coverage
Total
Methylated
chr14_haplotype2-0000078
Mean CpG methylation (%)
100
80
60
40
20
0
Coverage (x)
200
150
100
50
0
0.0
2.5
5.0
7.5
10.0
Position (Mbp)
Sequence composition
α-satellite
Human satellite
Other satellite
Non-satellite
Coverage
Total
Methylated

chr15

K200084_1_haplotype1-0000007_chr15

results/chr15_1_22694466/moddotplot/K200084_1/K200084_1_haplotype1-0000007_chr15

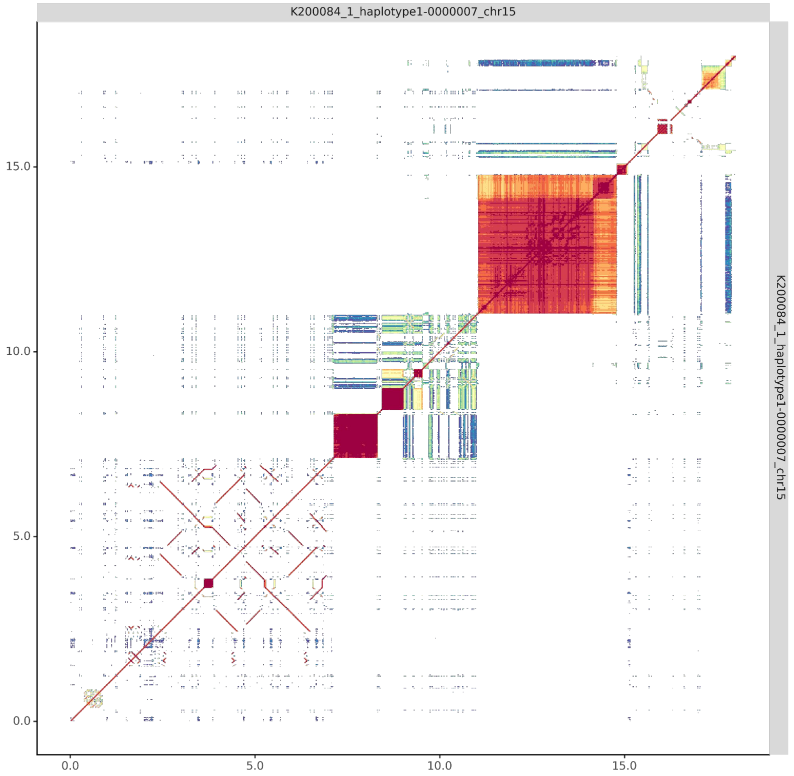

K200084_2_haplotype2-0000070_chr15

results/chr15_1_22694466/moddotplot/K200084_2/K200084_2_haplotype2-0000070_chr15

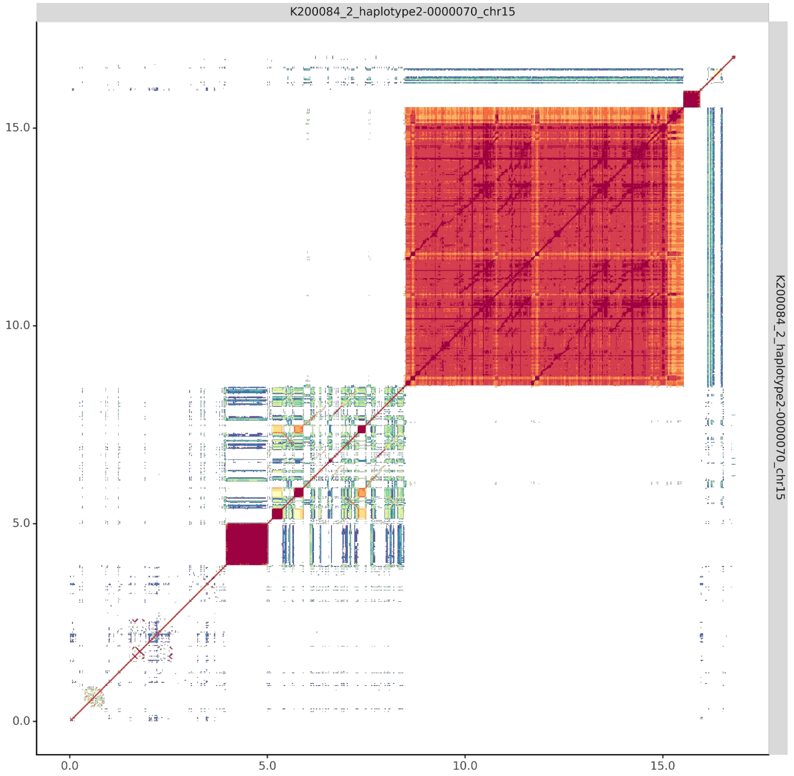

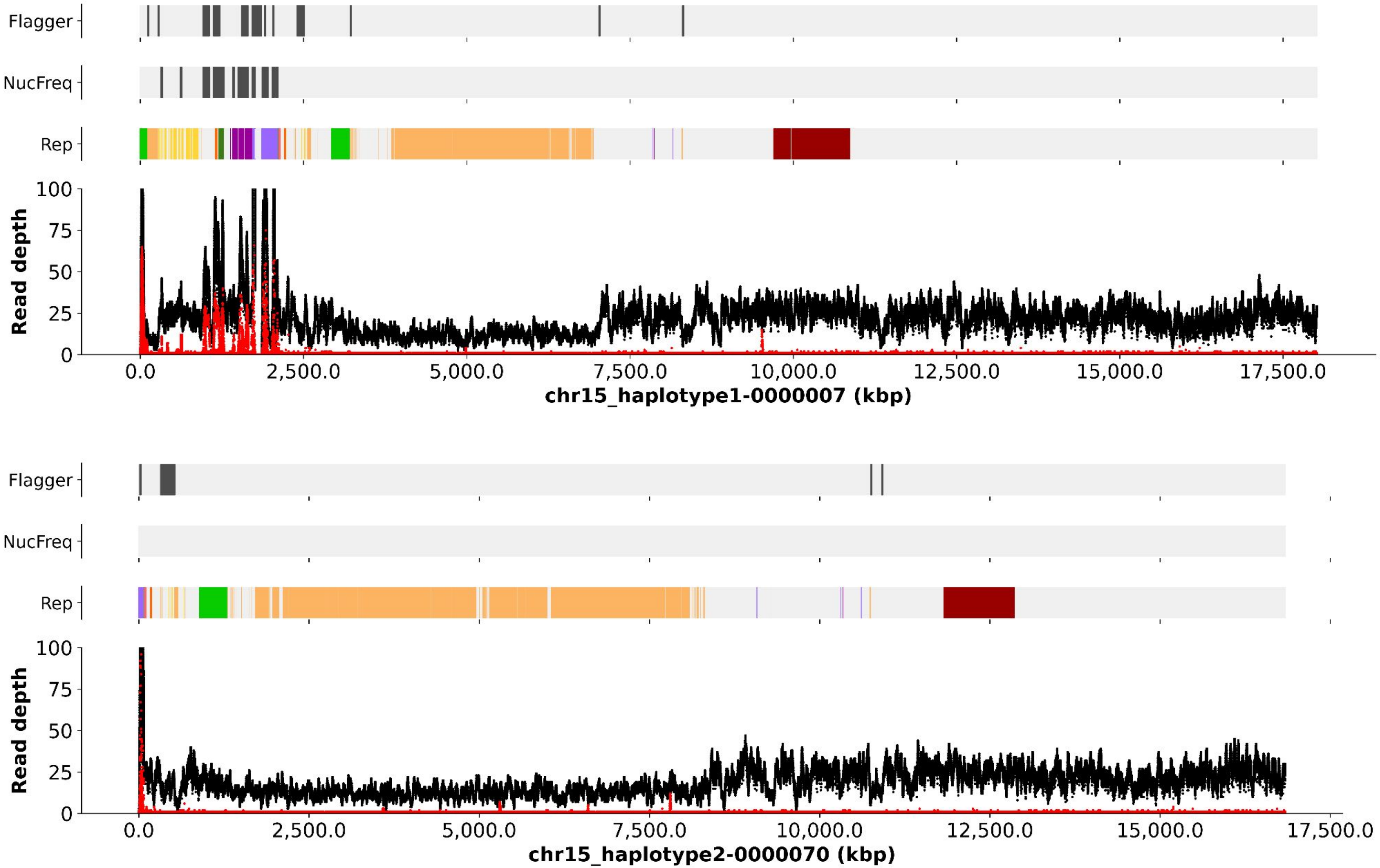

Flagger
NucFreq
Rep
Read depth
100
75
50
25
0
0.0
2,500.0
5,000.0
7,500.0
10,000.0
12,500.0
15,000.0
17,500.0
chr15_haplotype1-0000007 (kbp)
Flagger
NucFreq
Rep
Read depth
100
75
50
25
0
0.0
2,500.0
5,000.0
7,500.0
10,000.0
12,500.0
15,000.0
17,500.0
chr15_haplotype2-0000070 (kbp)

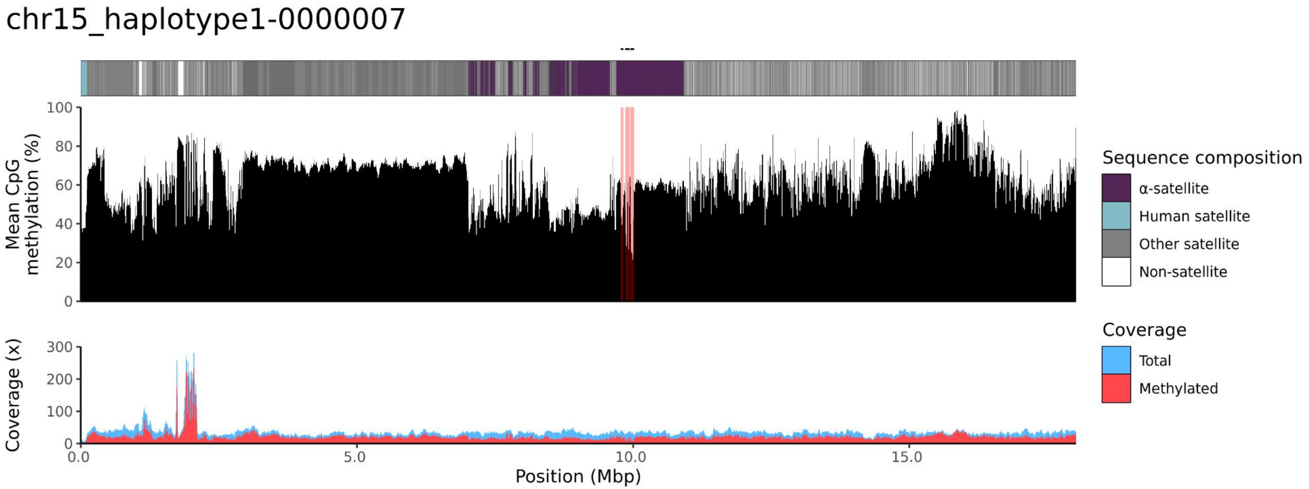
chr15_haplotype1-0000007
Mean CpG methylation (%)
Coverage (x)
Position (Mbp)
Sequence composition
α-satellite
Human satellite
Other satellite
Non-satellite
Coverage
Total
Methylated

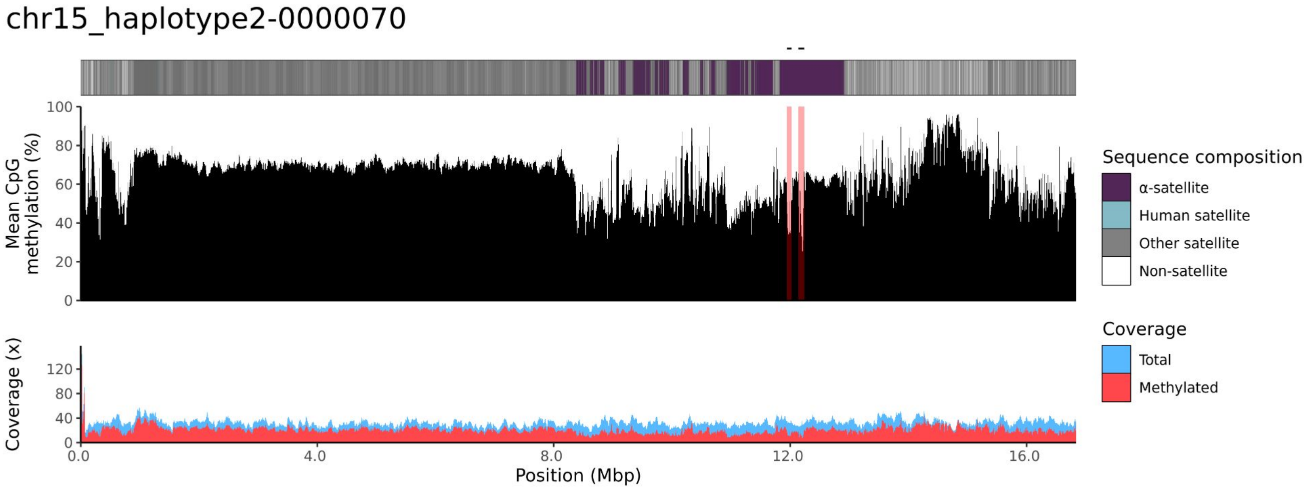
chr15_haplotype2-0000070
Mean CpG methylation (%)
Coverage (x)
Position (Mbp)
Sequence composition
α-satellite
Human satellite
Other satellite
Non-satellite
Coverage
Total
Methylated

chr21

K200084_1_haplotype1-0000012_chr21

results/chr21_1_16306378/moddotplot/K200084_1/K200084_1_haplotype1-0000012_chr21

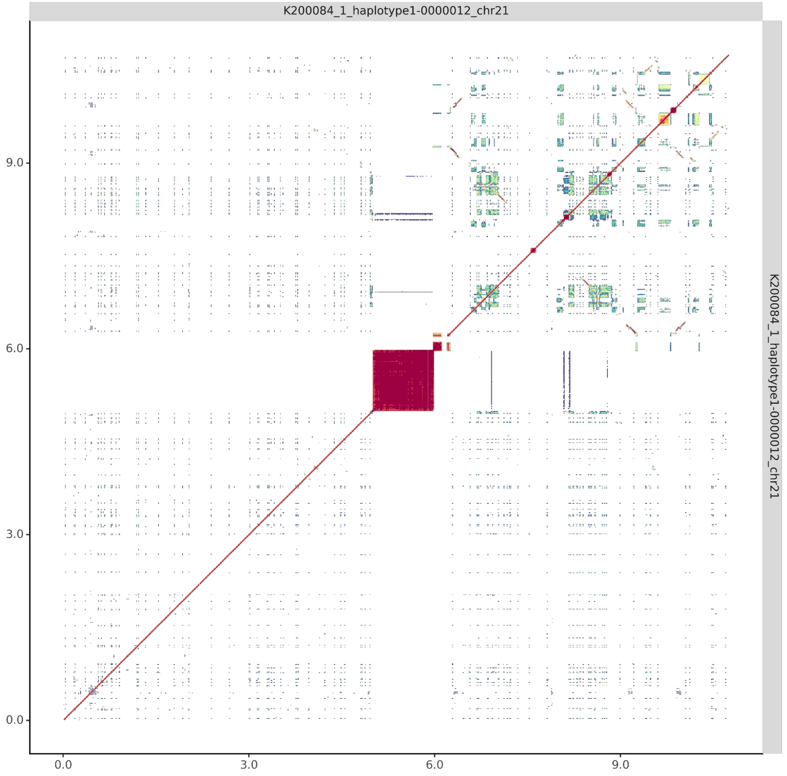

K200084_2_haplotype2-0000074_chr21

results/chr21_1_16306378/moddotplot/K200084_2/K200084_2_haplotype2-0000074_chr21

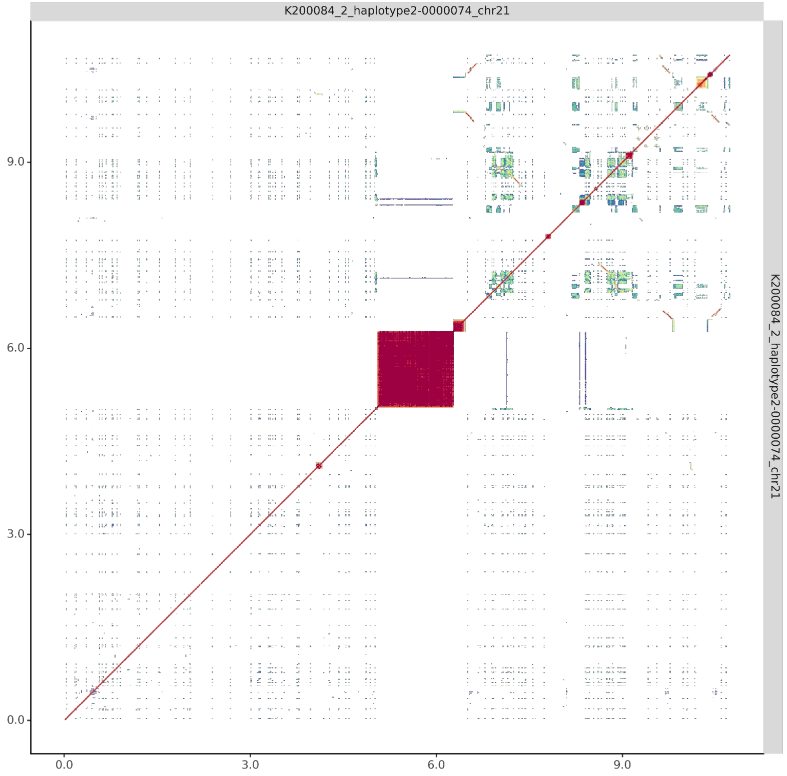

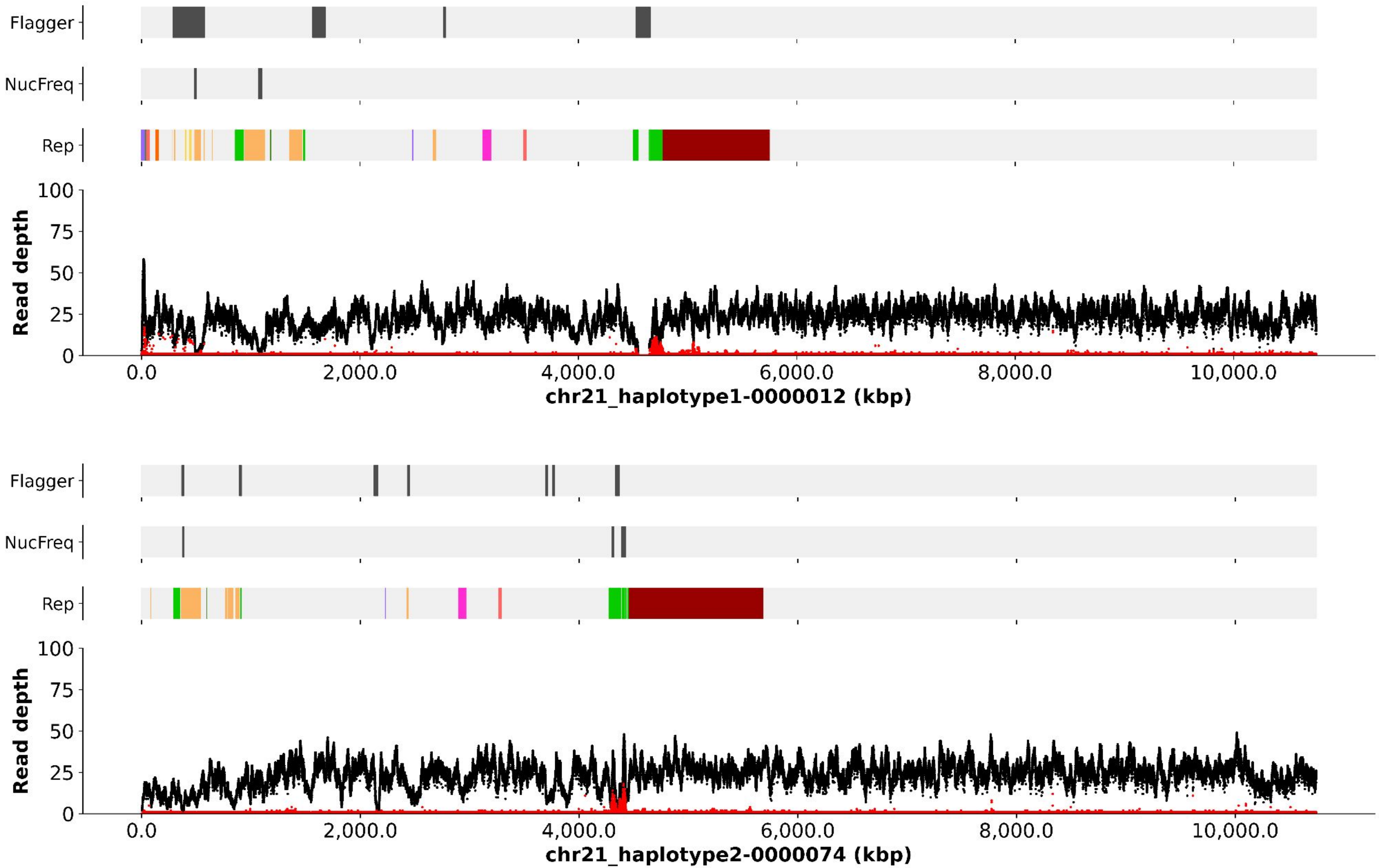

Flagger
NucFreq
Rep
Read depth
100
75
50
25
0
0.0
2,000.0
4,000.0
6,000.0
8,000.0
10,000.0
chr21_haplotype1-0000012 (kbp)
Flagger
NucFreq
Rep
Read depth
100
75
50
25
0
0.0
2,000.0
4,000.0
6,000.0
8,000.0
10,000.0
chr21_haplotype2-0000074 (kbp)

chr21_haplotype1-0000012

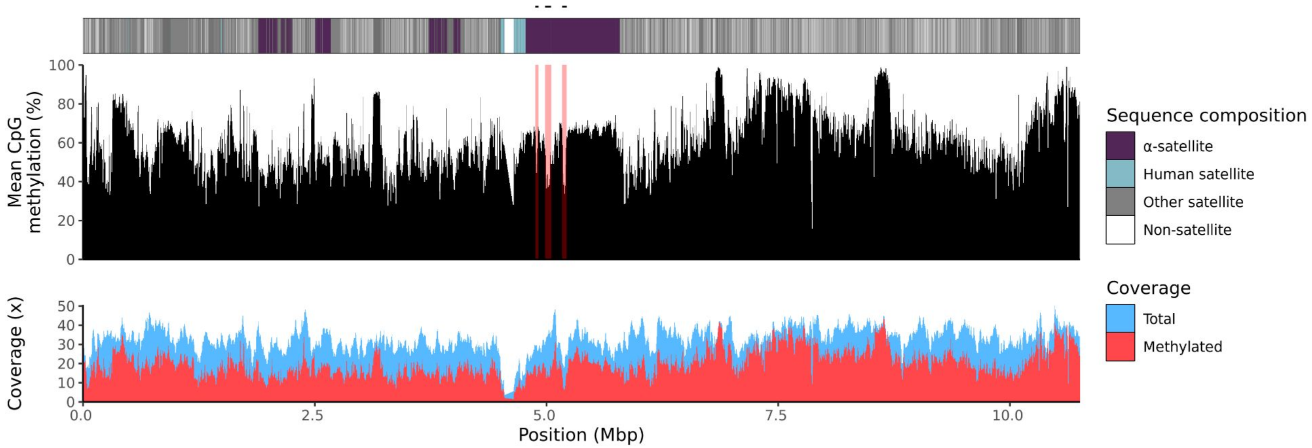

chr21_haplotype2-0000074

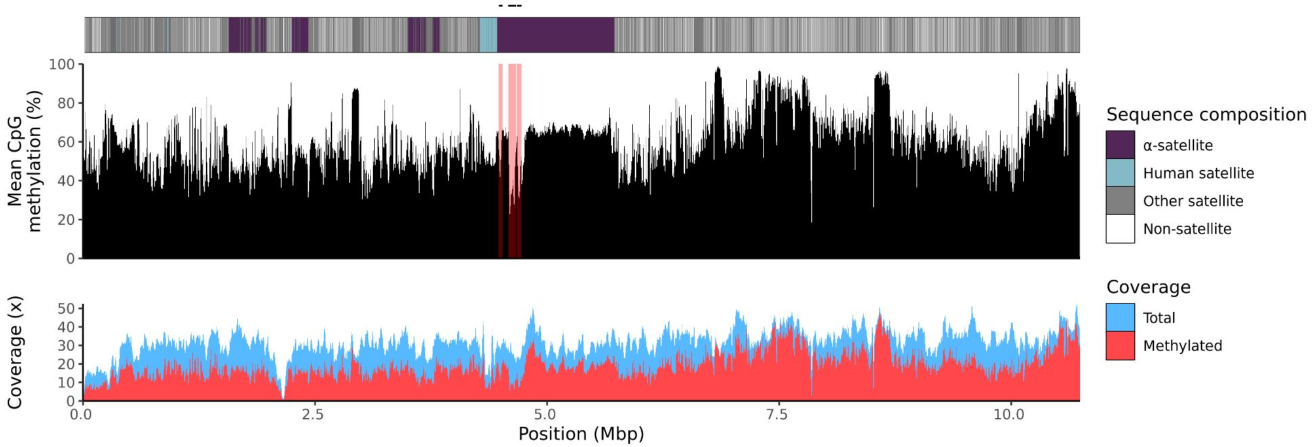

chr22

K200084_1_haplotype1-0000026_chr22

results/chr22_1_20711065/moddotplot/K200084_1/K200084_1_haplotype1-0000026_chr22

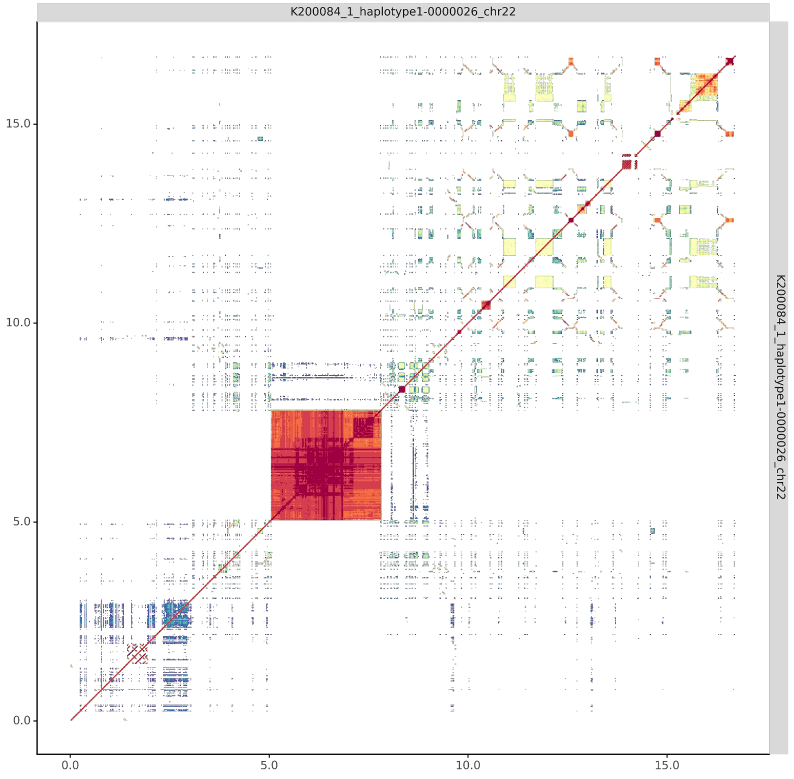

K200084_2_haplotype2-0000088_chr22

results/chr22_1_20711065/moddotplot/K200084_2/K200084_2_haplotype2-0000088_chr22

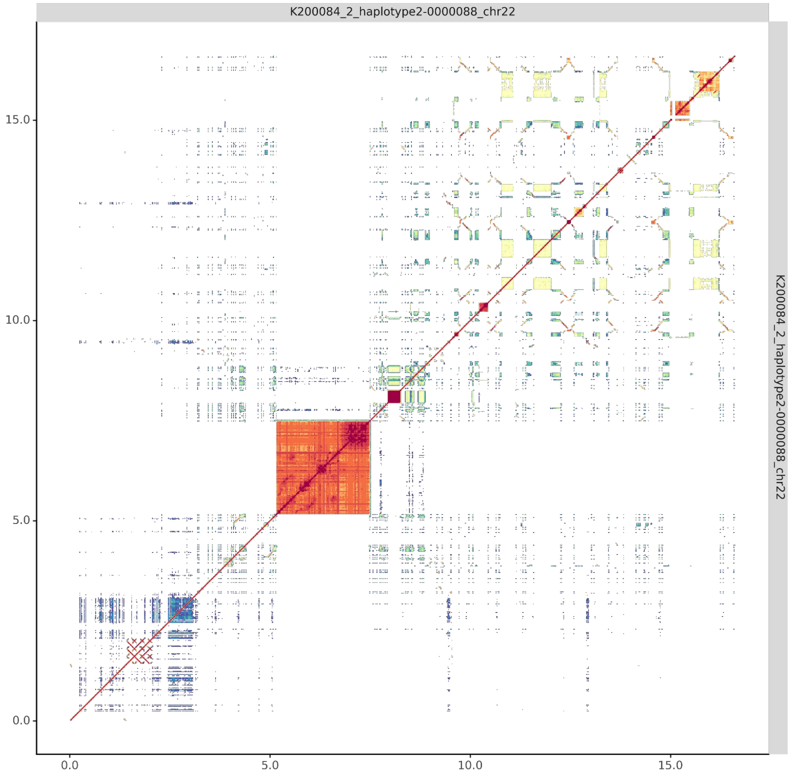

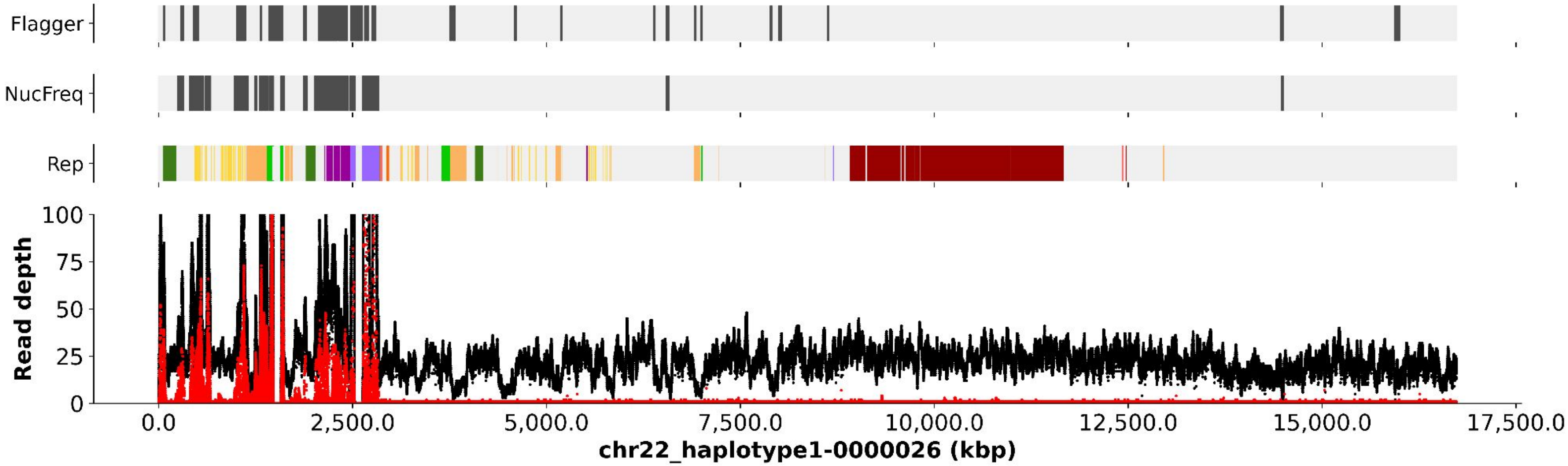
Flagger
NucFreq
Rep
Read depth
100
75
50
25
0
0.0
2,500.0
5,000.0
7,500.0
10,000.0
12,500.0
15,000.0
17,500.0
chr22_haplotype1-0000026 (kbp)

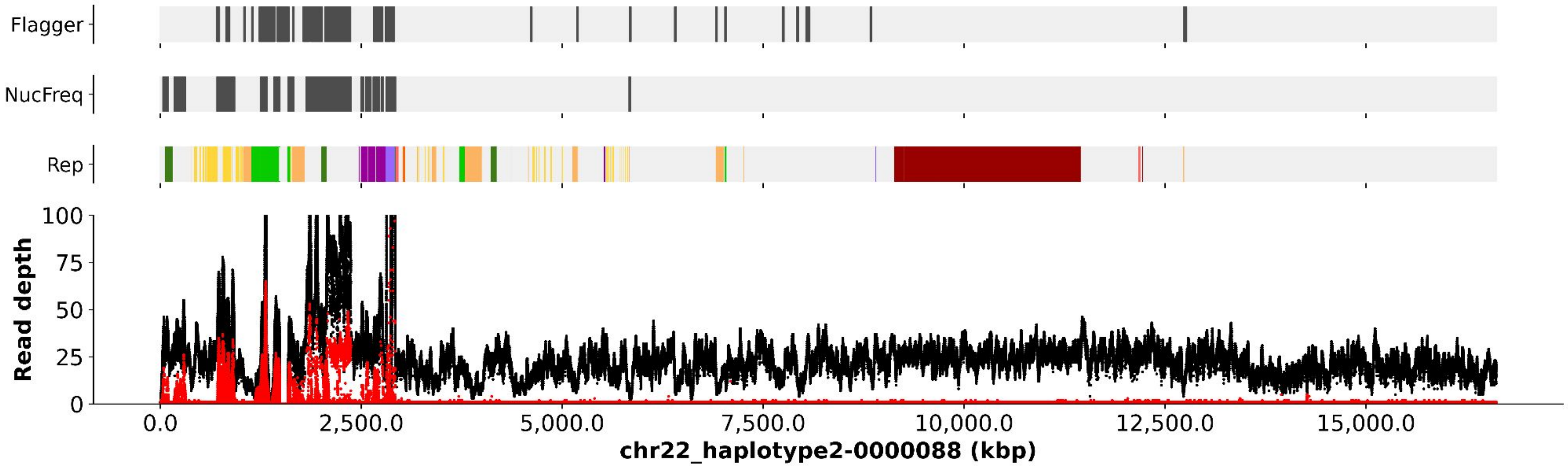
Flagger
NucFreq
Rep
Read depth
100
75
50
25
0
0.0
2,500.0
5,000.0
7,500.0
10,000.0
12,500.0
15,000.0
chr22_haplotype2-0000088 (kbp)

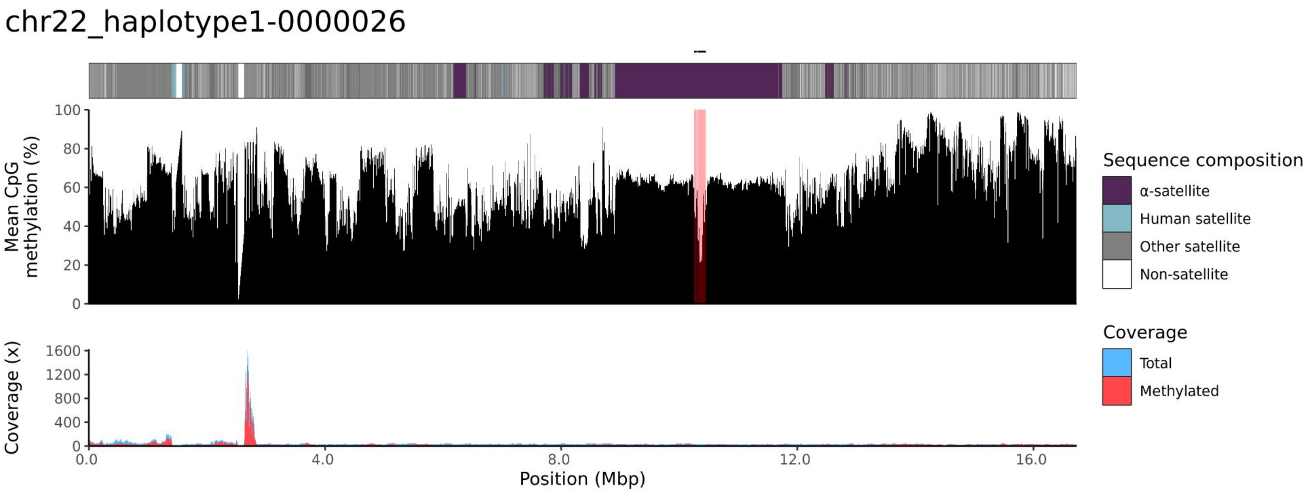

chr22_haplotype1-0000026
Mean CpG methylation (%)
Coverage (x)
Position (Mbp)
Sequence composition
α-satellite
Human satellite
Other satellite
Non-satellite
Coverage
Total
Methylated

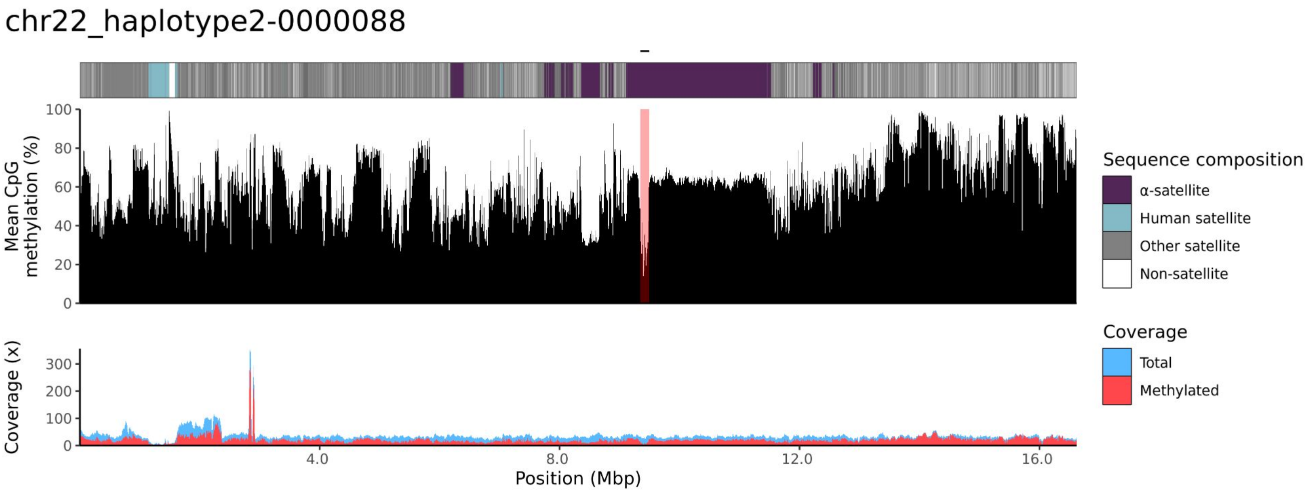

chr22_haplotype2-0000088
Mean CpG methylation (%)
Coverage (x)
Position (Mbp)
Sequence composition
α-satellite
Human satellite
Other satellite
Non-satellite
Coverage
Total
Methylated

200085

chr13

K200085_1_haplotype1-0000015_chr13

results/chr13_1_22508596/moddotplot/K200085_1/K200085_1_haplotype1-0000015_chr13

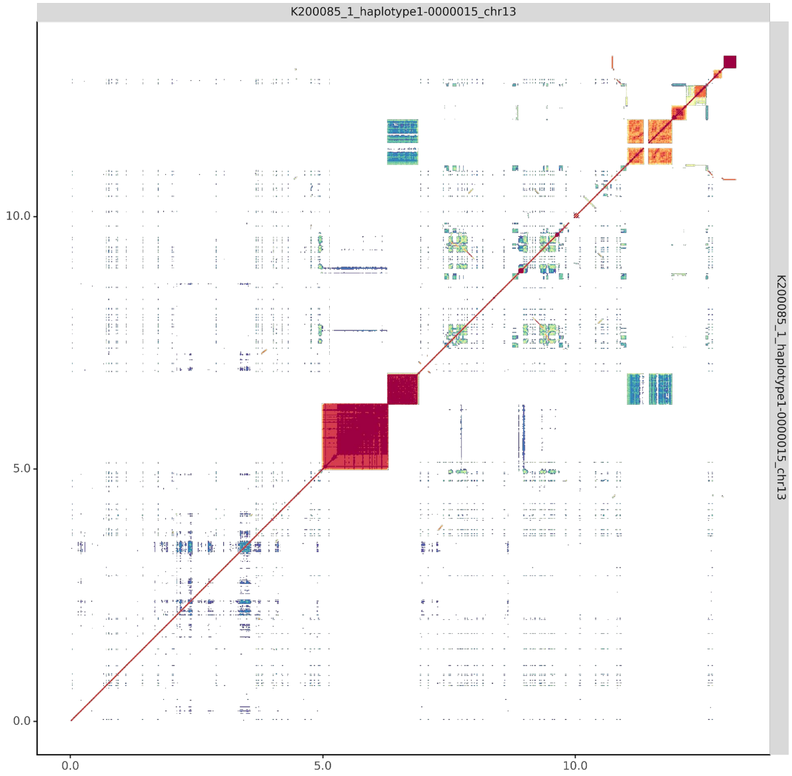

K200085_2_haplotype2-0000056_chr13

results/chr13_1_22508596/moddotplot/K200085_2/K200085_2_haplotype2-0000056_chr13

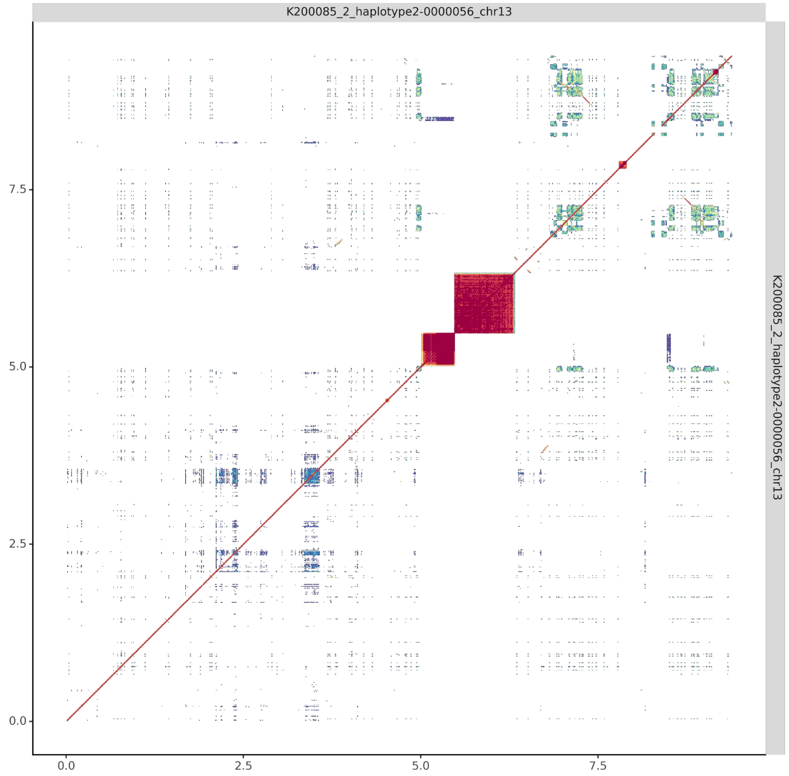

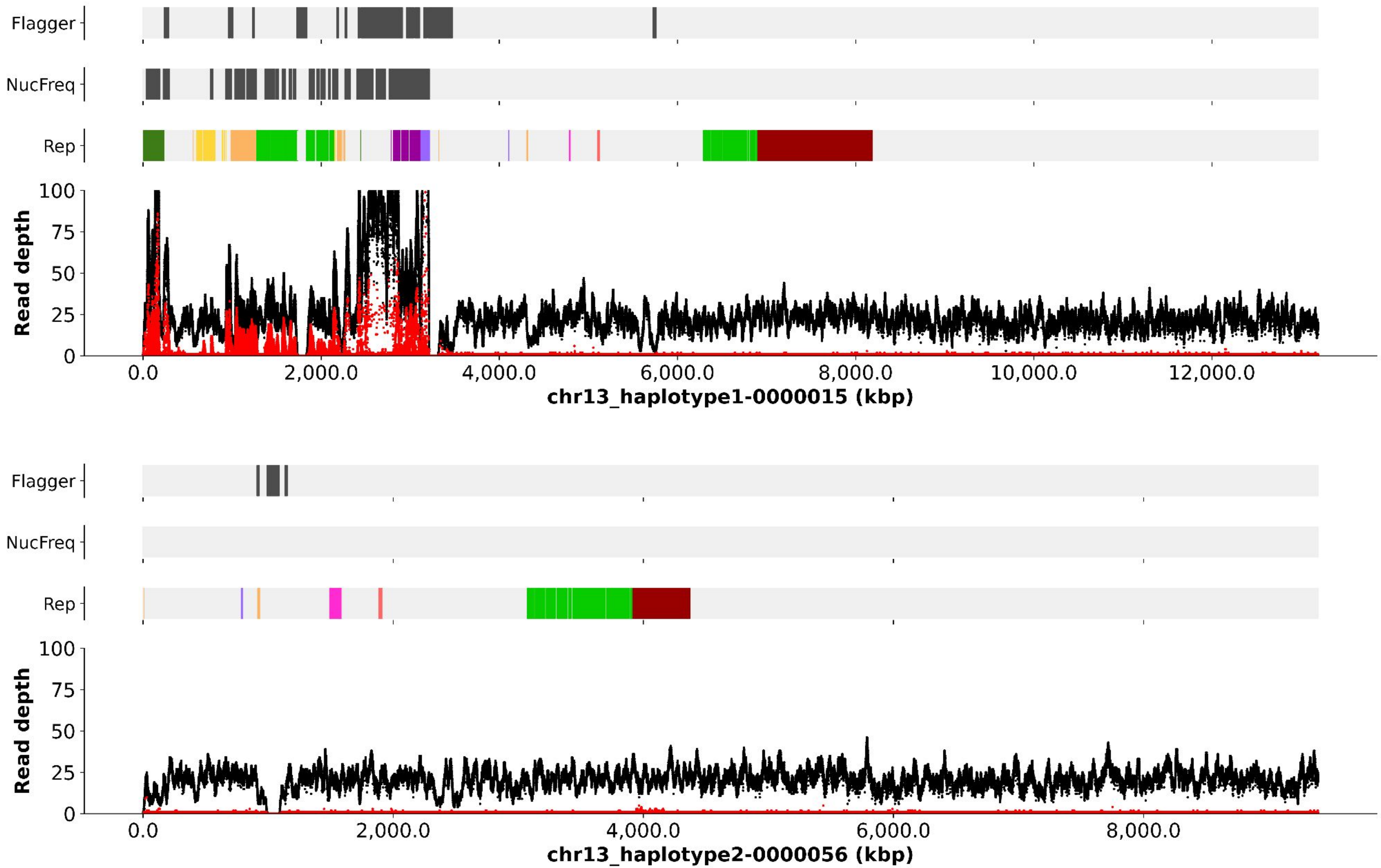
Flagger
NucFreq
Rep
Read depth
100
75
50
25
0
0.0
2,000.0
4,000.0
6,000.0
8,000.0
10,000.0
12,000.0
chr13_haplotype1-0000015 (kbp)
Flagger
NucFreq
Rep
Read depth
100
75
50
25
0
0.0
2,000.0
4,000.0
6,000.0
8,000.0
chr13_haplotype2-0000056 (kbp)

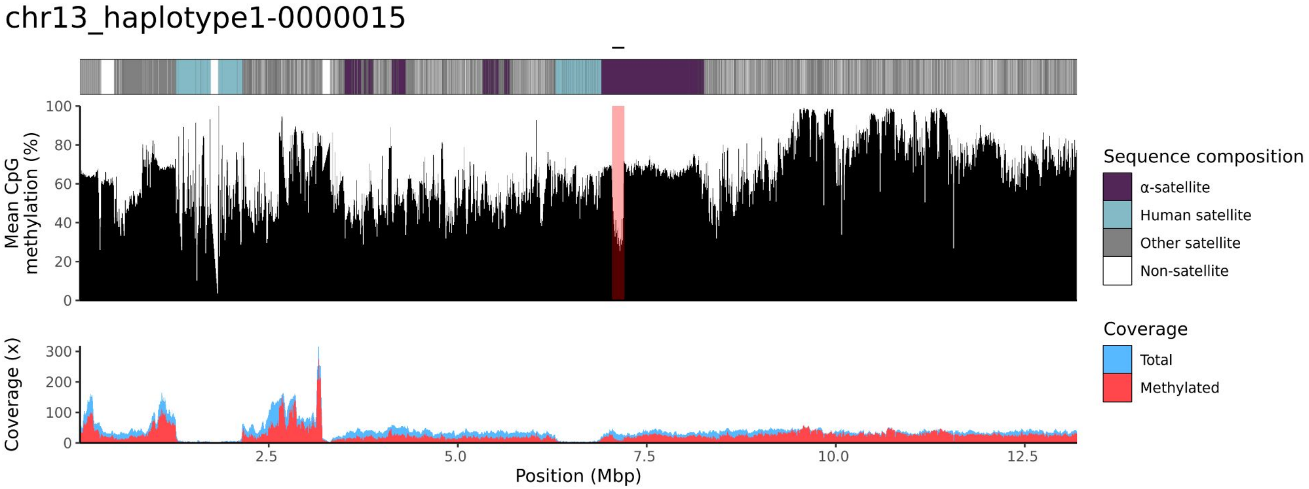
chr13_haplotype1-0000015
Mean CpG methylation (%)
Coverage (x)
Position (Mbp)
Sequence composition
α-satellite
Human satellite
Other satellite
Non-satellite
Coverage
Total
Methylated

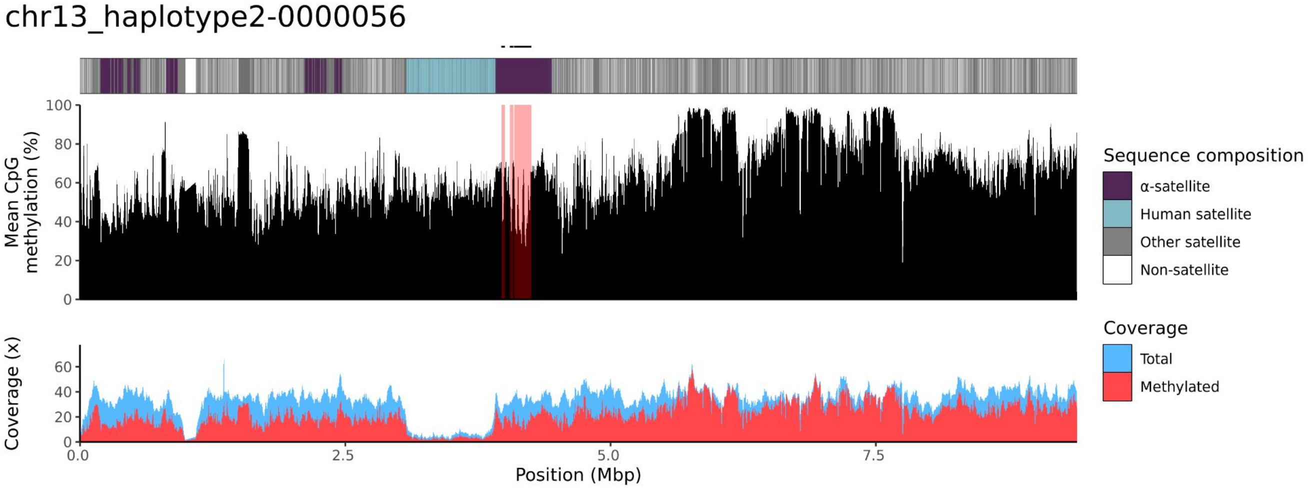
chr13_haplotype2-0000056
Mean CpG methylation (%)
Coverage (x)
Position (Mbp)
Sequence composition
α-satellite
Human satellite
Other satellite
Non-satellite
Coverage
Total
Methylated

chr14

K200085_1_haplotype1-0000026_chr14

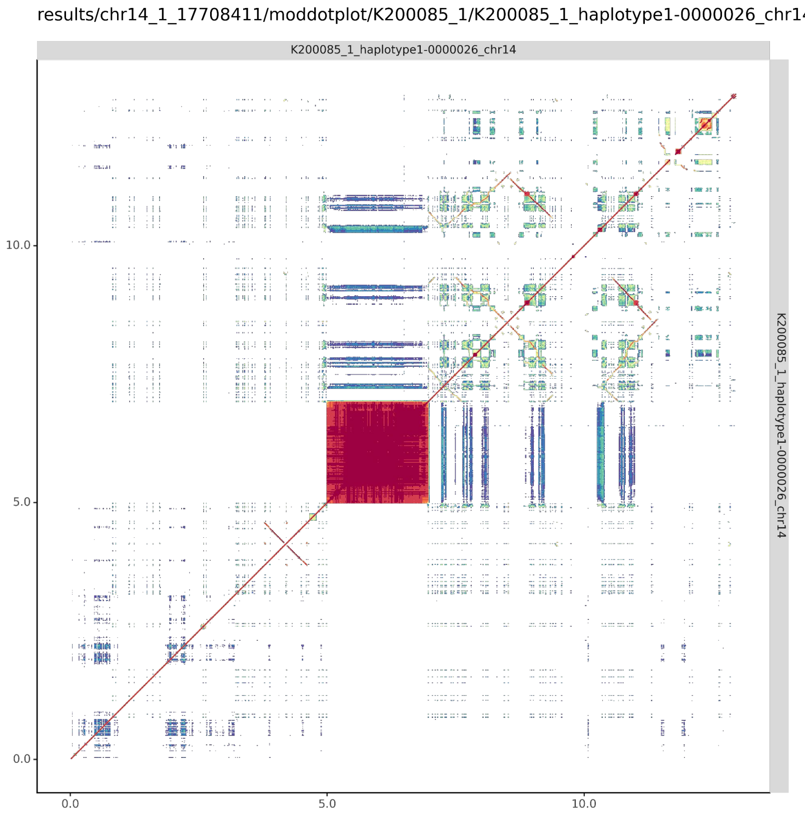

K200085_2_haplotype2-0000064_chr14

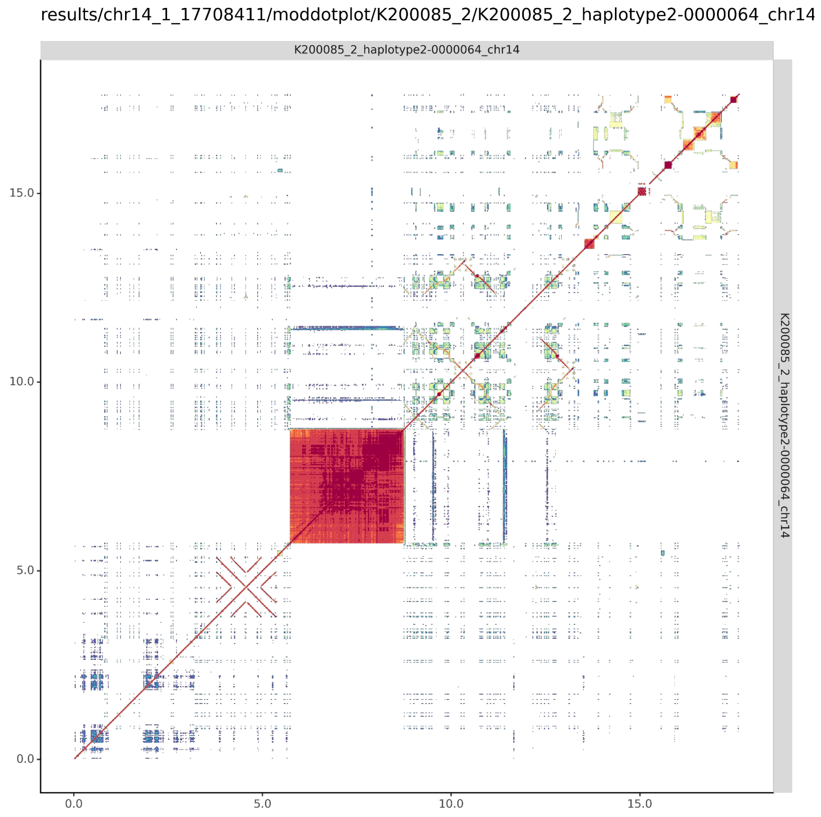

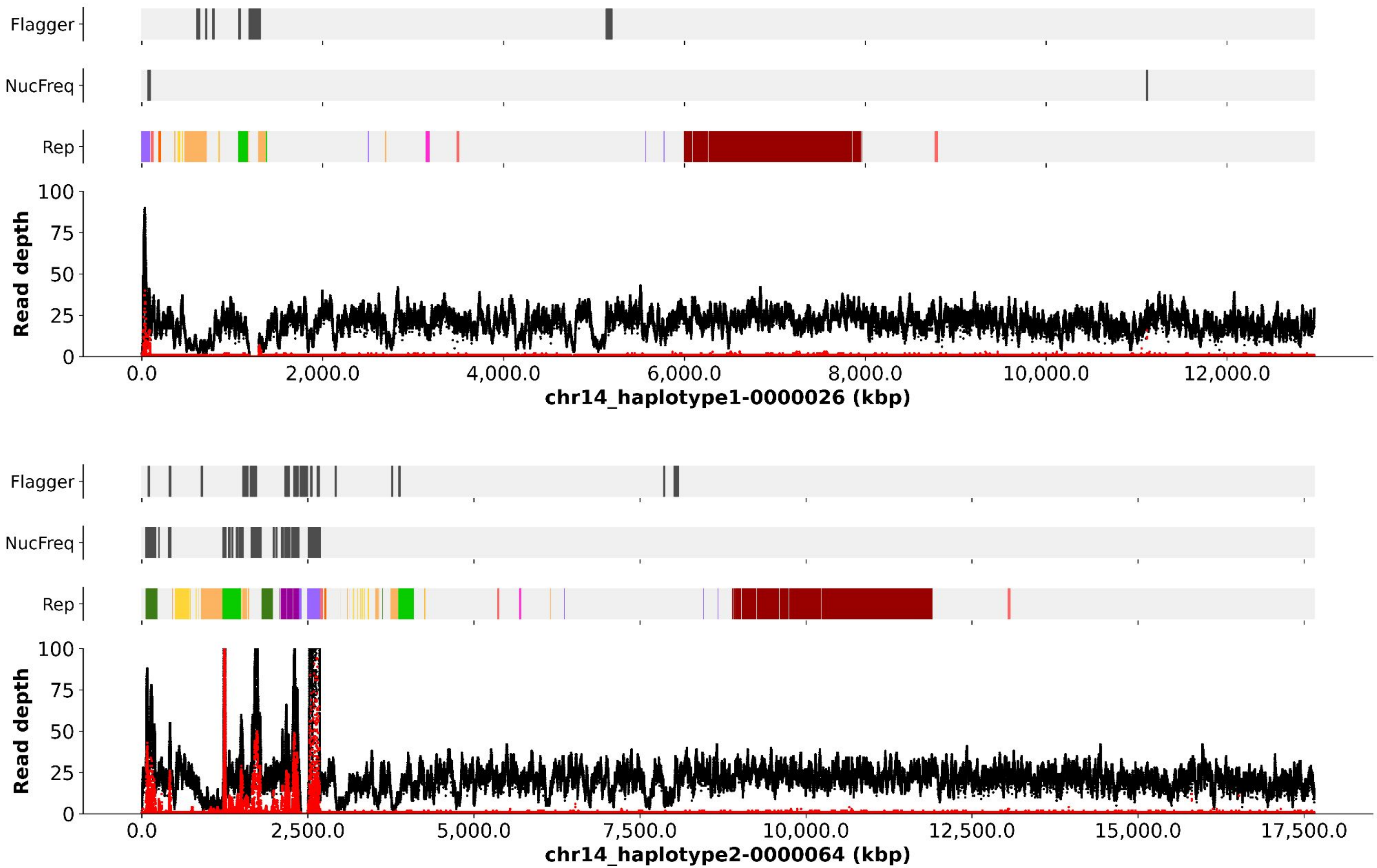
Flagger
NucFreq
Rep
Read depth
100
75
50
25
0
0.0
2,000.0
4,000.0
6,000.0
8,000.0
10,000.0
12,000.0
chr14_haplotype1-0000026 (kbp)
Flagger
NucFreq
Rep
Read depth
100
75
50
25
0
0.0
2,500.0
5,000.0
7,500.0
10,000.0
12,500.0
15,000.0
17,500.0
chr14_haplotype2-0000064 (kbp)

chr14_haplotype1-0000026

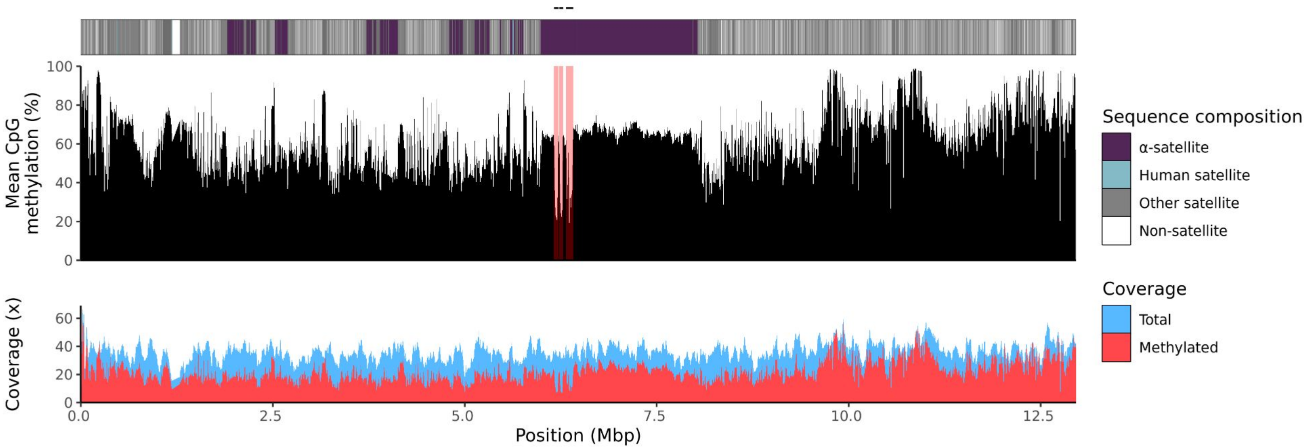

chr14_haplotype2-0000064

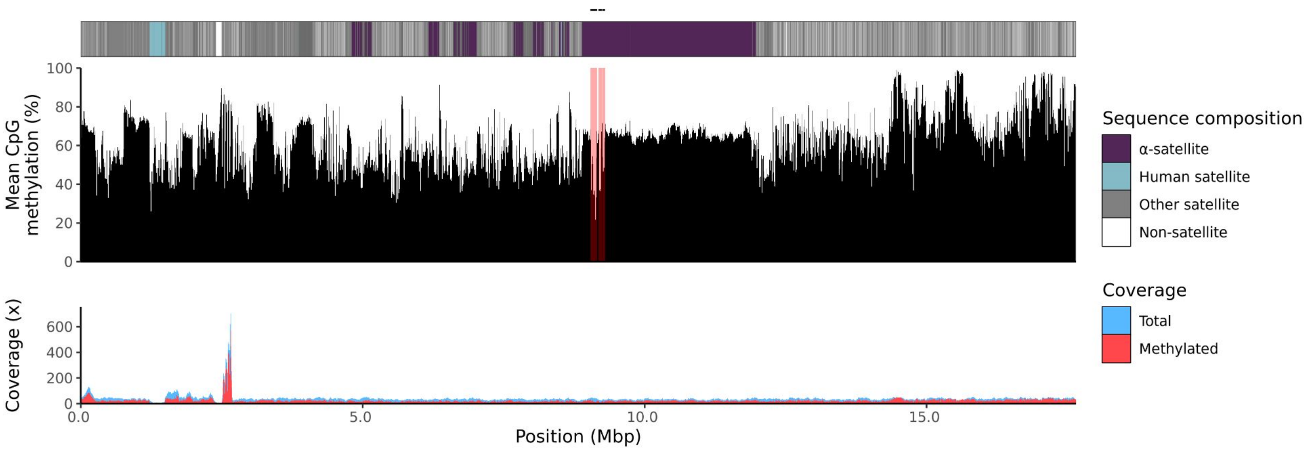

chr15

K200085_1_haplotype1-0000025_chr15

K200085_2_haplotype2-0000063_chr15

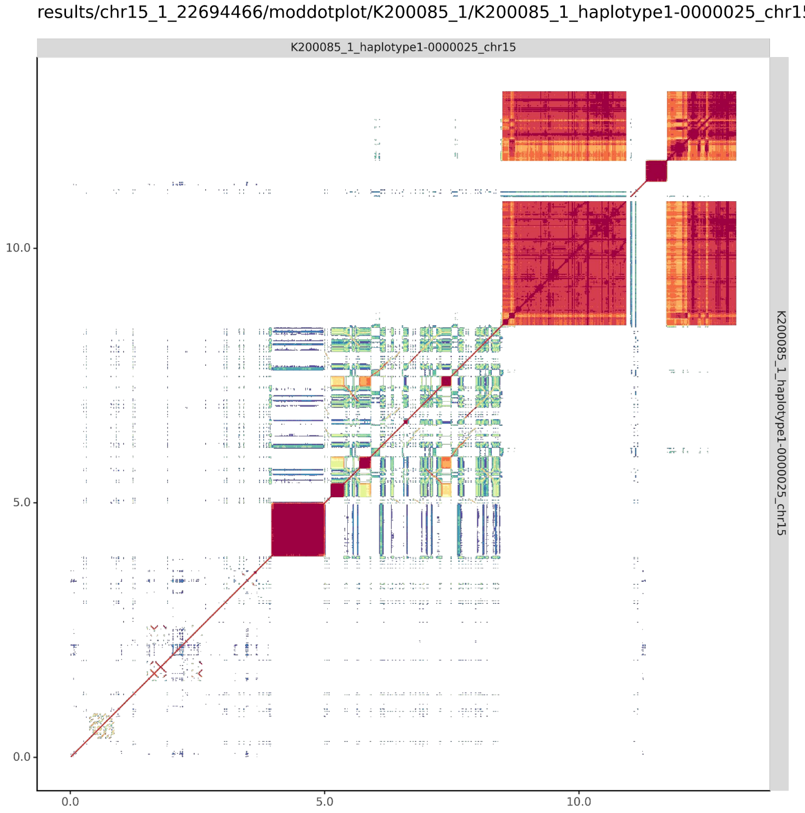

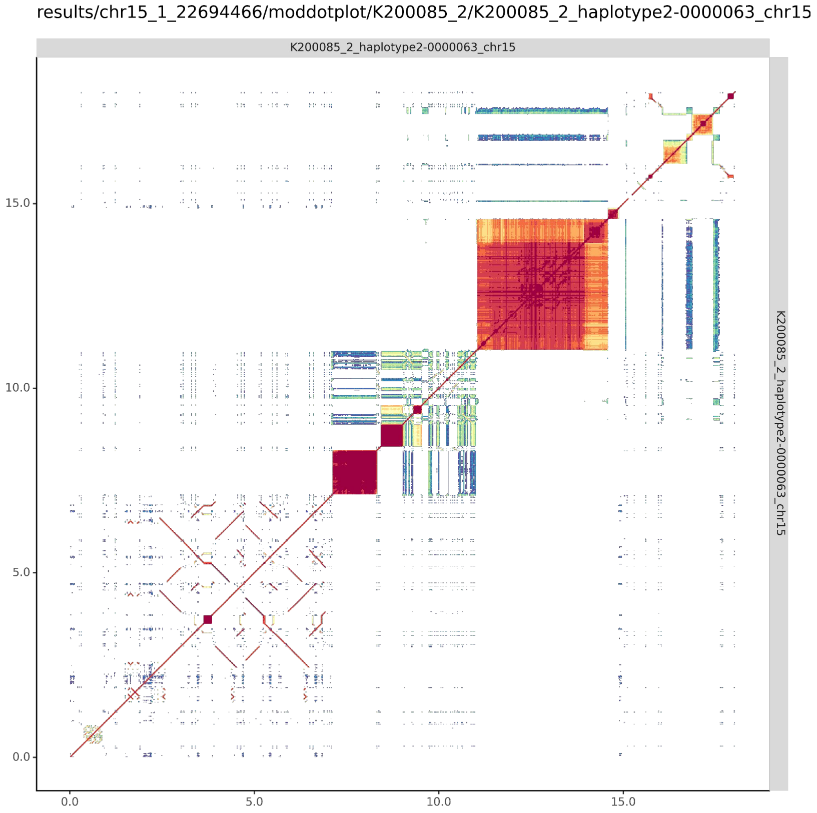

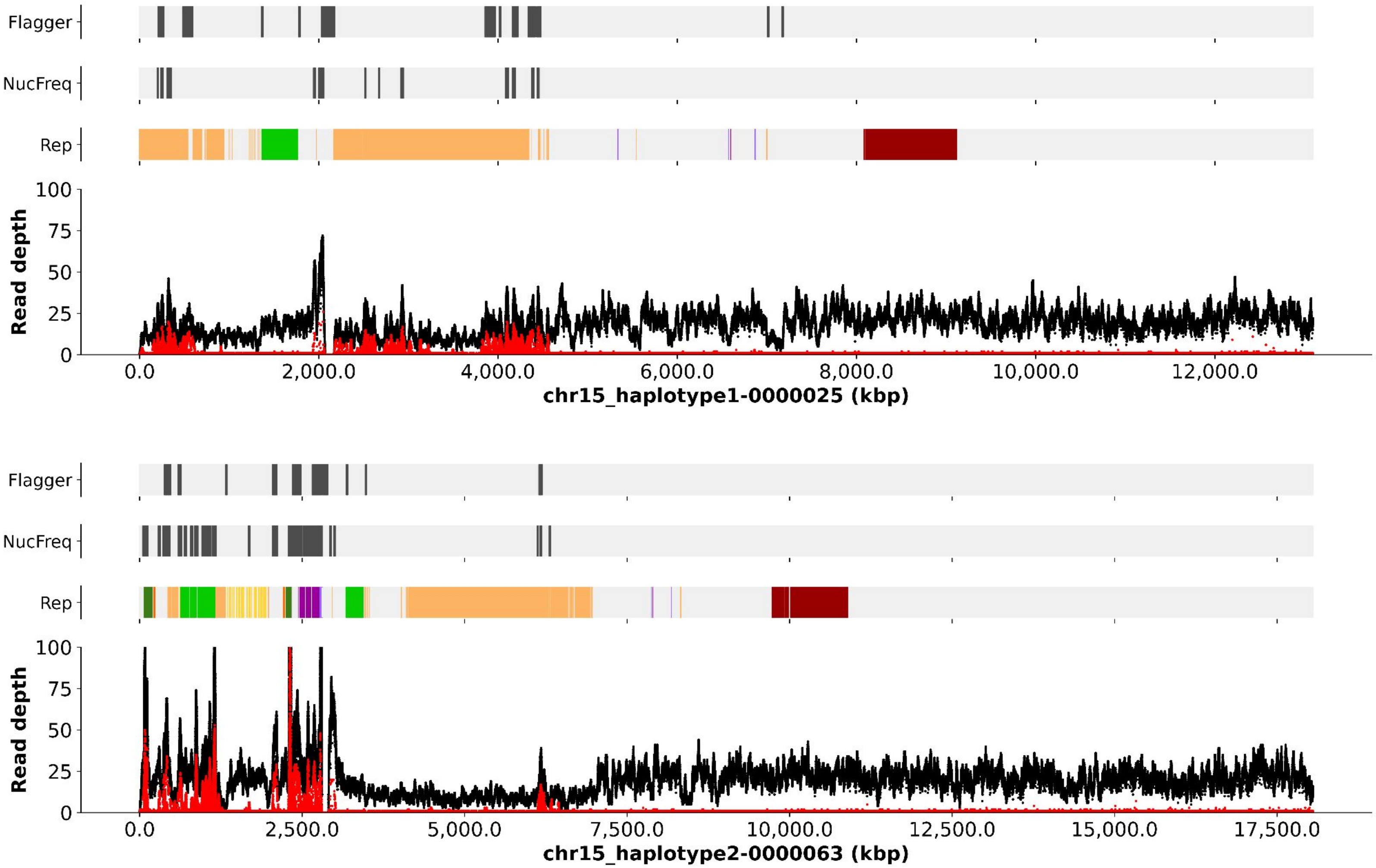
Flagger
NucFreq
Rep
Read depth
100
75
50
25
0
0.0
2,000.0
4,000.0
6,000.0
8,000.0
10,000.0
12,000.0
chr15_haplotype1-0000025 (kbp)
Flagger
NucFreq
Rep
Read depth
100
75
50
25
0
0.0
2,500.0
5,000.0
7,500.0
10,000.0
12,500.0
15,000.0
17,500.0
chr15_haplotype2-0000063 (kbp)

chr15_haplotype1-0000025

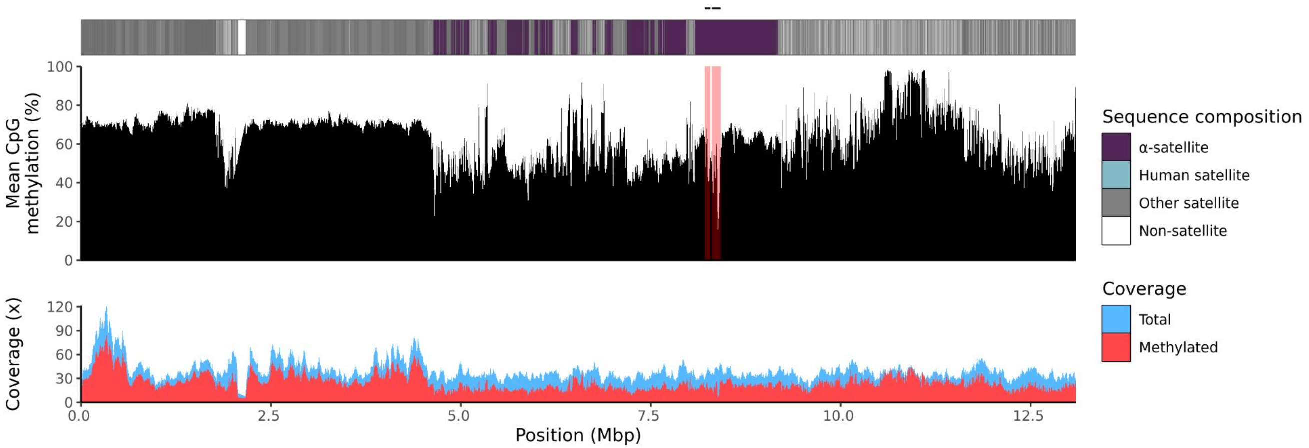

chr15_haplotype2-0000063

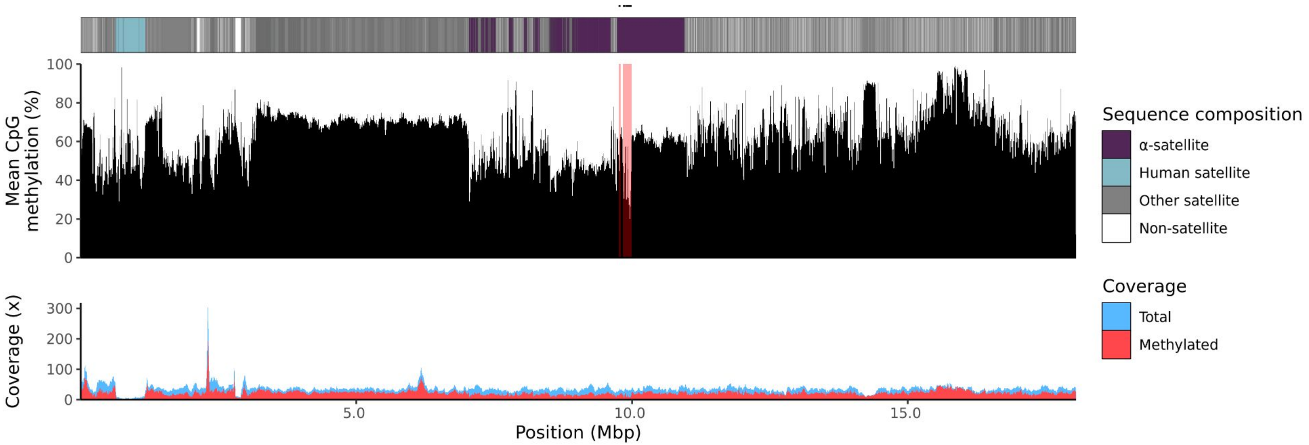

chr21

K200085_1_haplotype1-0000012_chr21

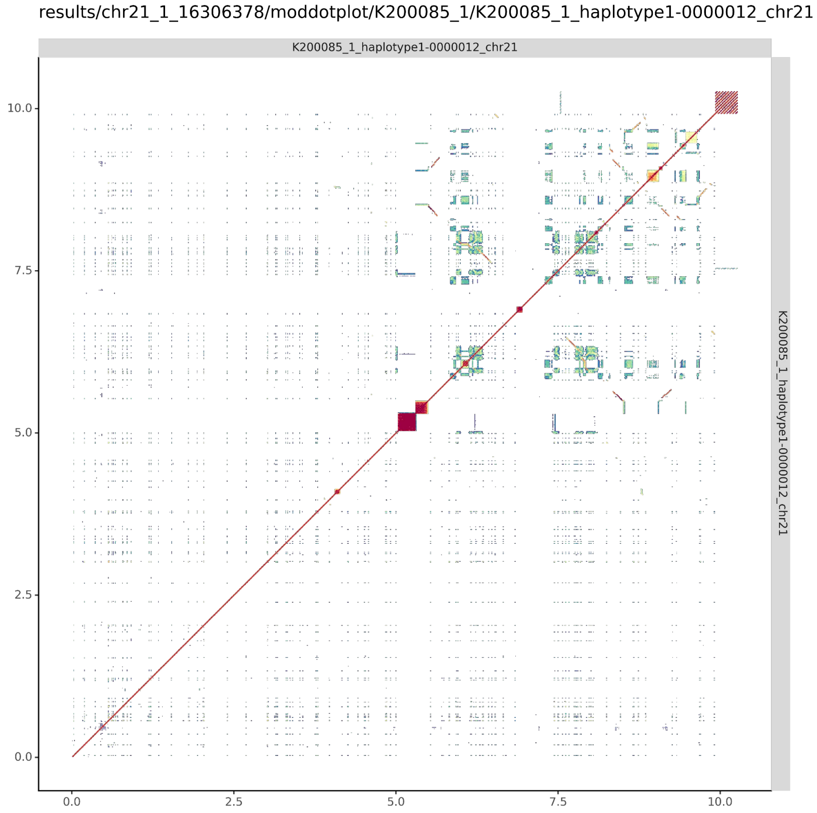

K200085_2_haplotype2-0000053_chr21

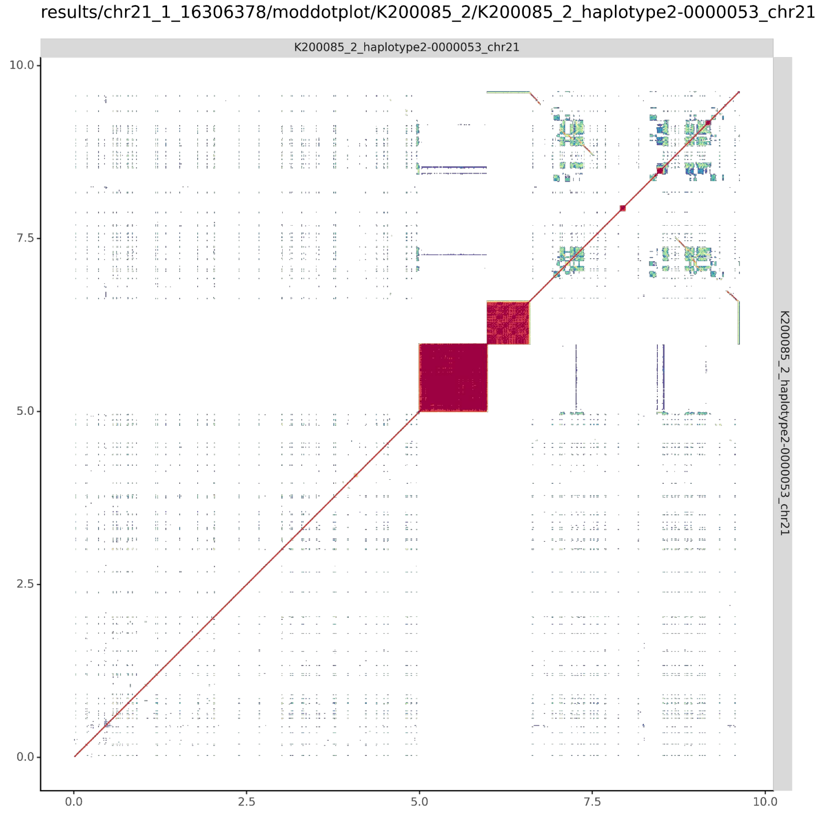

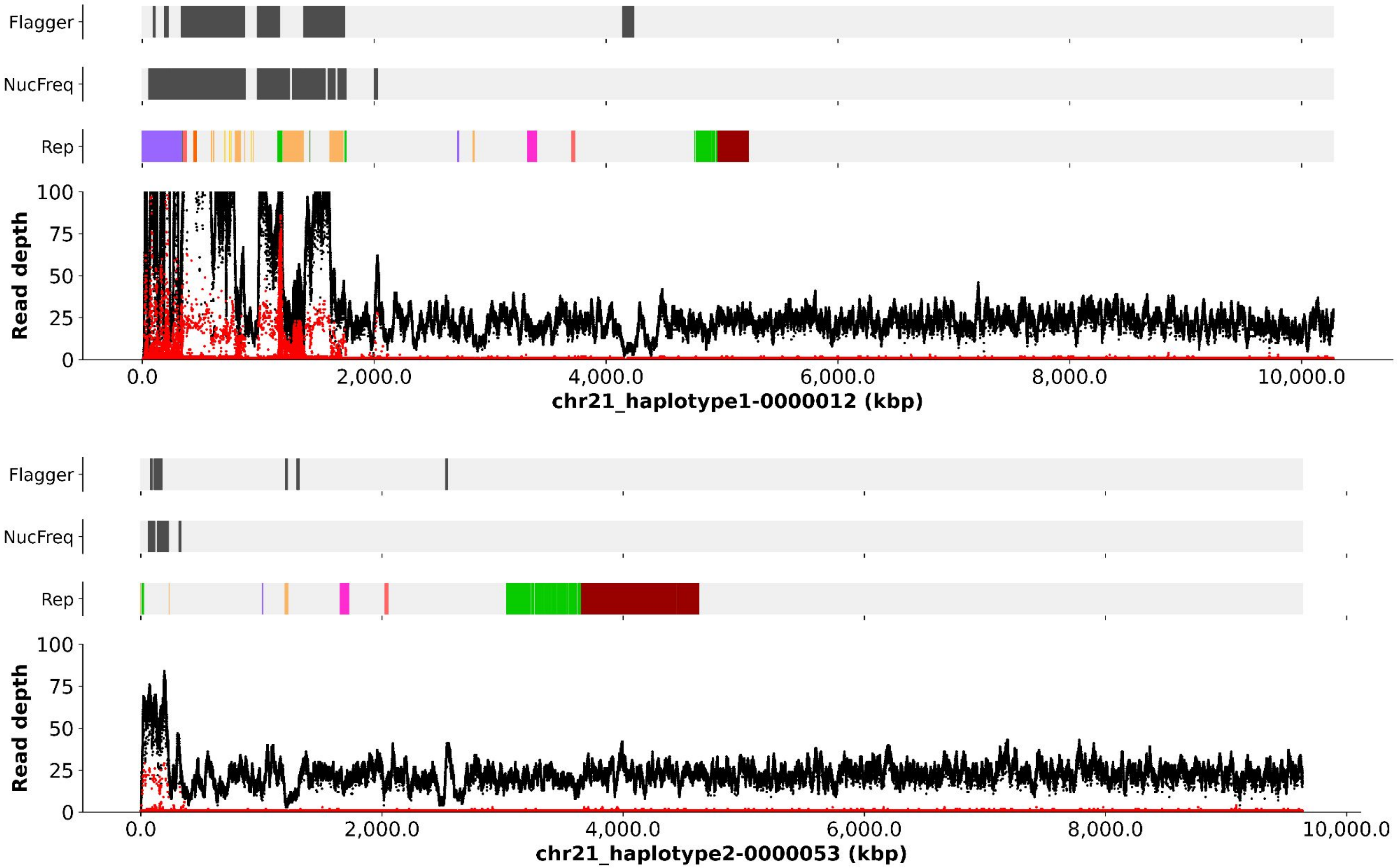

Flagger
NucFreq
Rep
Read depth
100
75
50
25
0
0.0
2,000.0
4,000.0
6,000.0
8,000.0
10,000.0
chr21_haplotype1-0000012 (kbp)
Flagger
NucFreq
Rep
Read depth
100
75
50
25
0
0.0
2,000.0
4,000.0
6,000.0
8,000.0
10,000.0
chr21_haplotype2-0000053 (kbp)

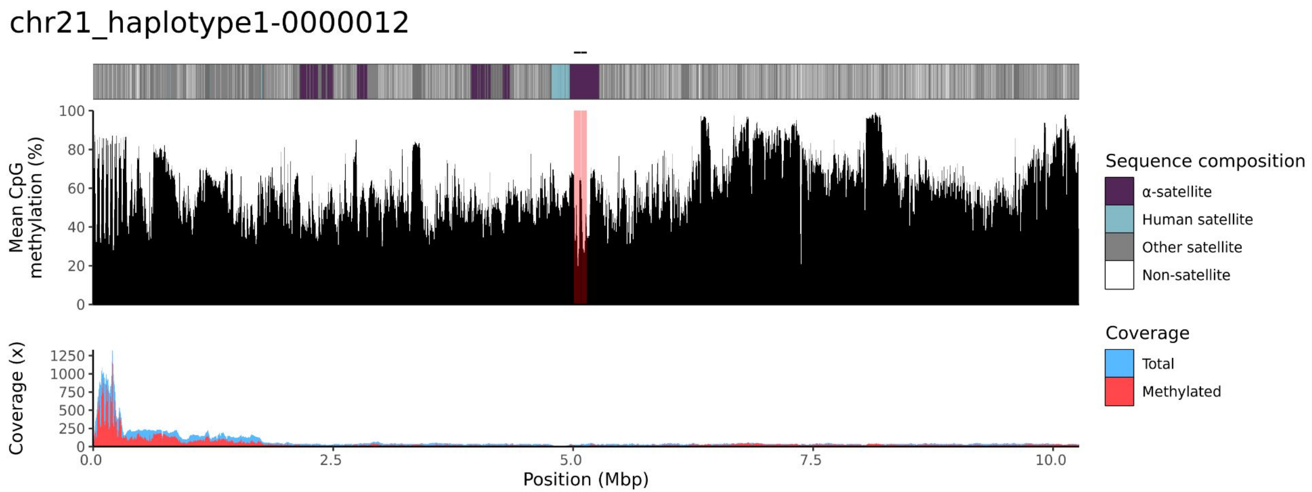
chr21_haplotype1-0000012
Mean CpG methylation (%)
100
80
60
40
20
0
Coverage (x)
1250
1000
750
500
250
0
0.0
2.5
5.0
7.5
10.0
Position (Mbp)
Sequence composition
α-satellite
Human satellite
Other satellite
Non-satellite
Coverage
Total
Methylated

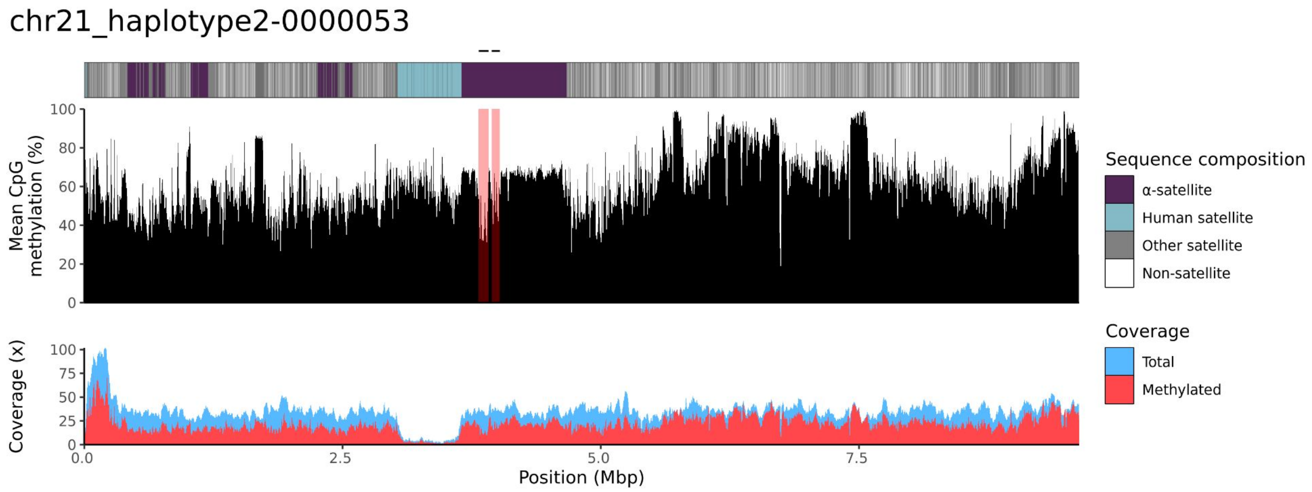
chr21_haplotype2-0000053
Mean CpG methylation (%)
100
80
60
40
20
0
Coverage (x)
100
75
50
25
0
0.0
2.5
5.0
7.5
Position (Mbp)
Sequence composition
α-satellite
Human satellite
Other satellite
Non-satellite
Coverage
Total
Methylated

chr22

K200085_1_haplotype1-0000001_chr22

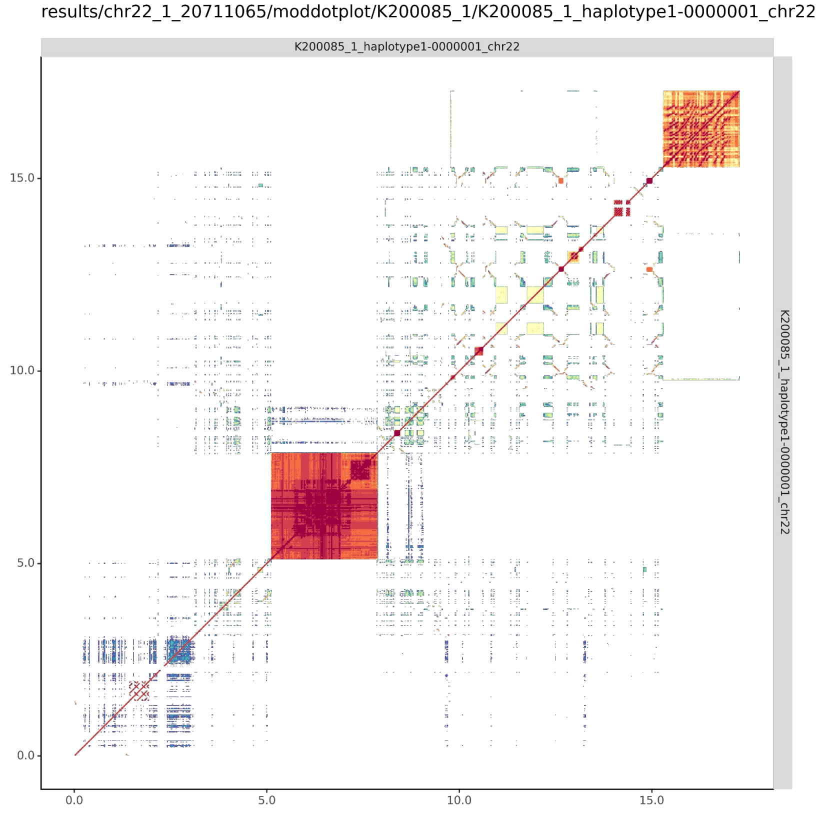

K200085_2_haplotype2-0000042_chr22

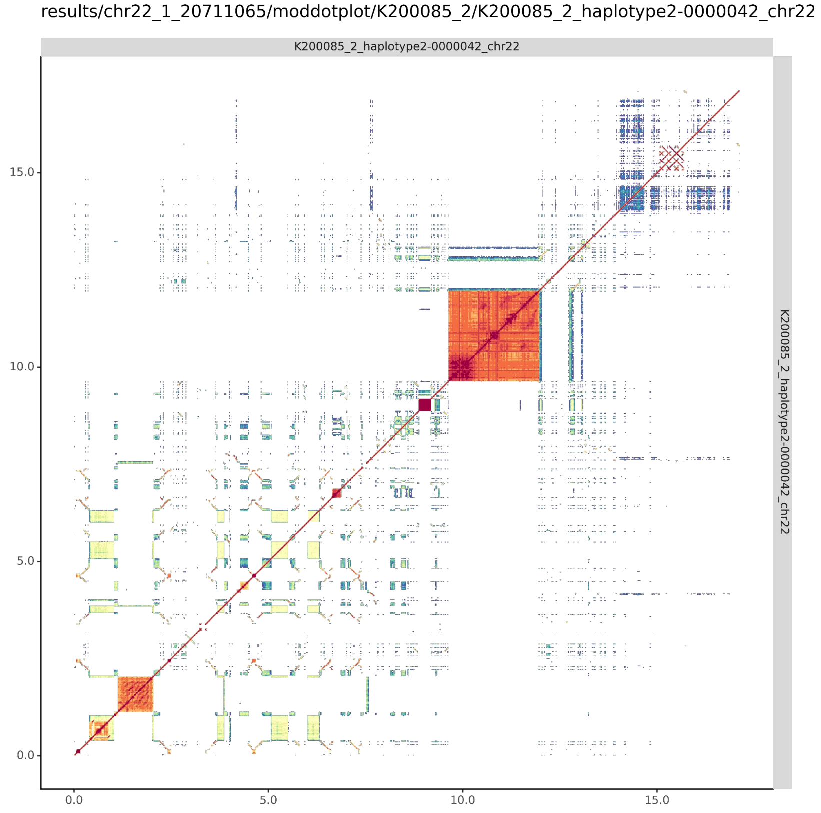

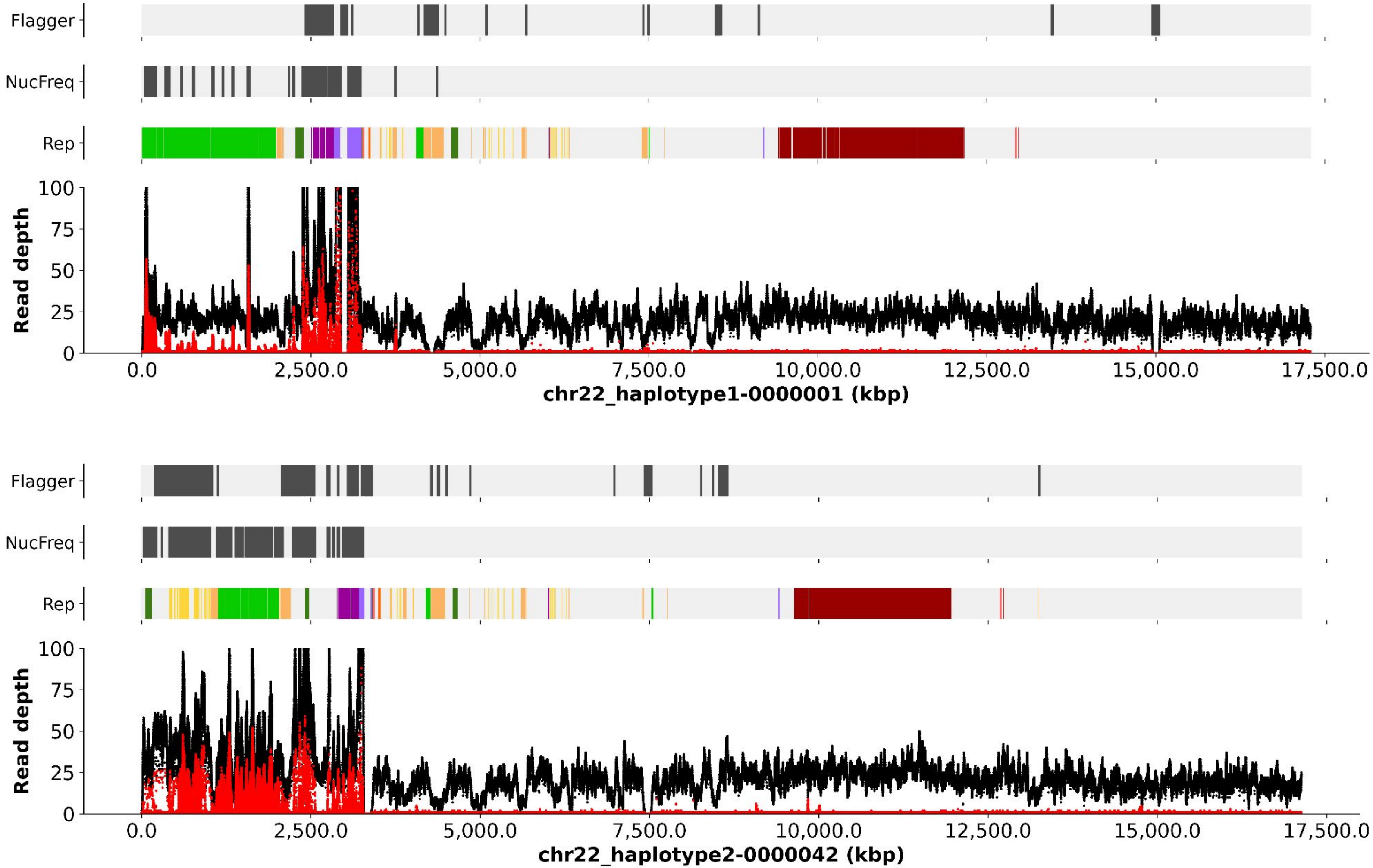
Flagger
NucFreq
Rep
Read depth
100
75
50
25
0
0.0
2,500.0
5,000.0
7,500.0
10,000.0
12,500.0
15,000.0
17,500.0
chr22_haplotype1-0000001 (kbp)
Flagger
NucFreq
Rep
Read depth
100
75
50
25
0
0.0
2,500.0
5,000.0
7,500.0
10,000.0
12,500.0
15,000.0
17,500.0
chr22_haplotype2-0000042 (kbp)

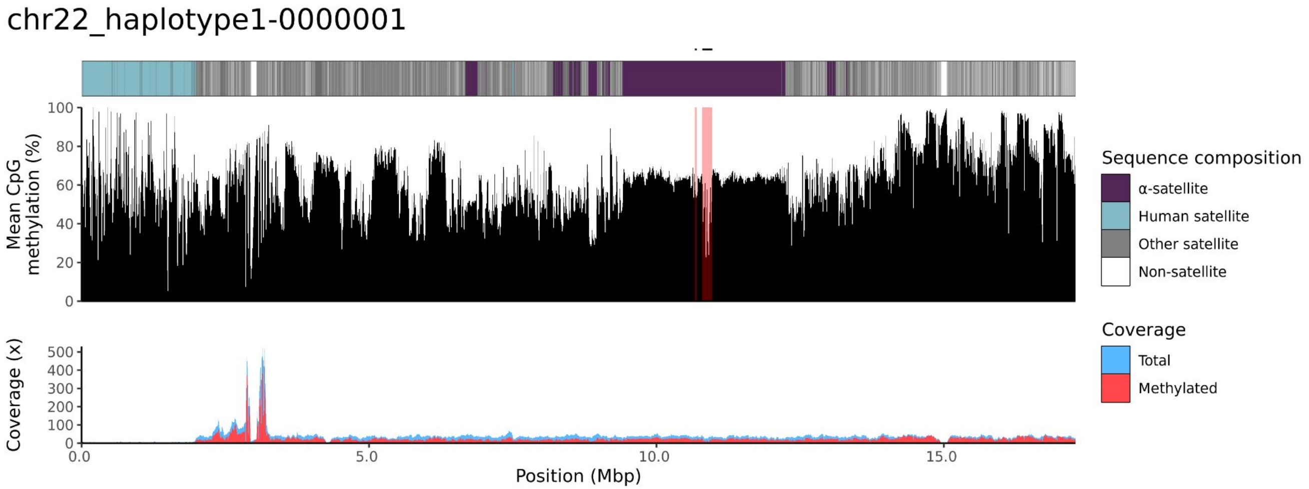
chr22_haplotype1-0000001
Mean CpG methylation (%)
100
80
60
40
20
0
Coverage (x)
500
400
300
200
100
0
0.0
5.0
10.0
15.0
Position (Mbp)
Sequence composition
α-satellite
Human satellite
Other satellite
Non-satellite
Coverage
Total
Methylated

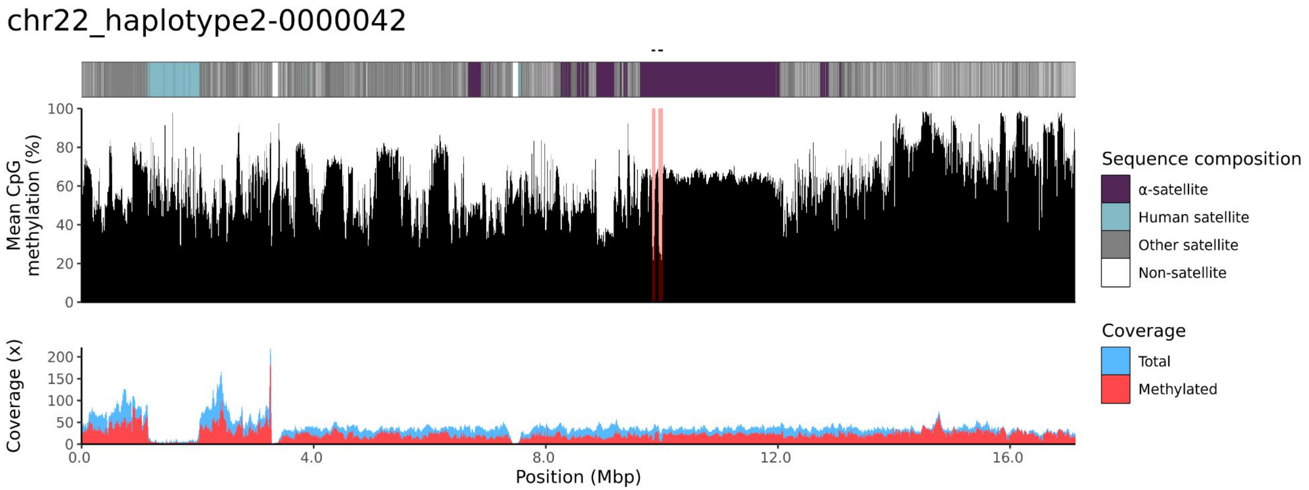
chr22_haplotype2-0000042
Mean CpG methylation (%)
100
80
60
40
20
0
Coverage (x)
200
150
100
50
0
0.0
4.0
8.0
12.0
16.0
Position (Mbp)
Sequence composition
α-satellite
Human satellite
Other satellite
Non-satellite
Coverage
Total
Methylated

200087

chr13

K200087_2_haplotype2-0000205_chr13

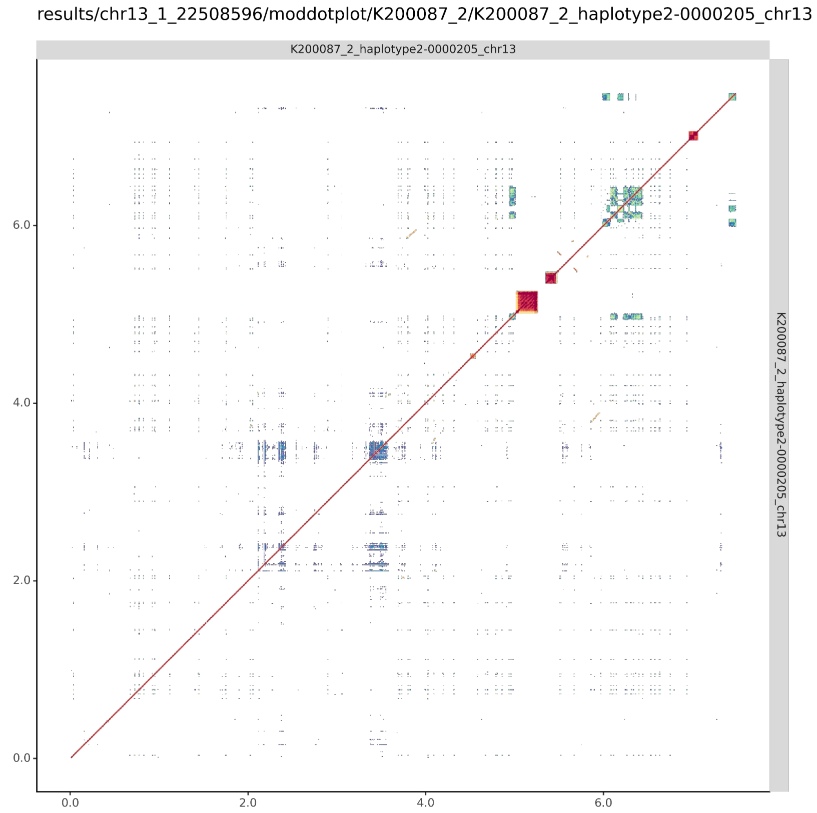

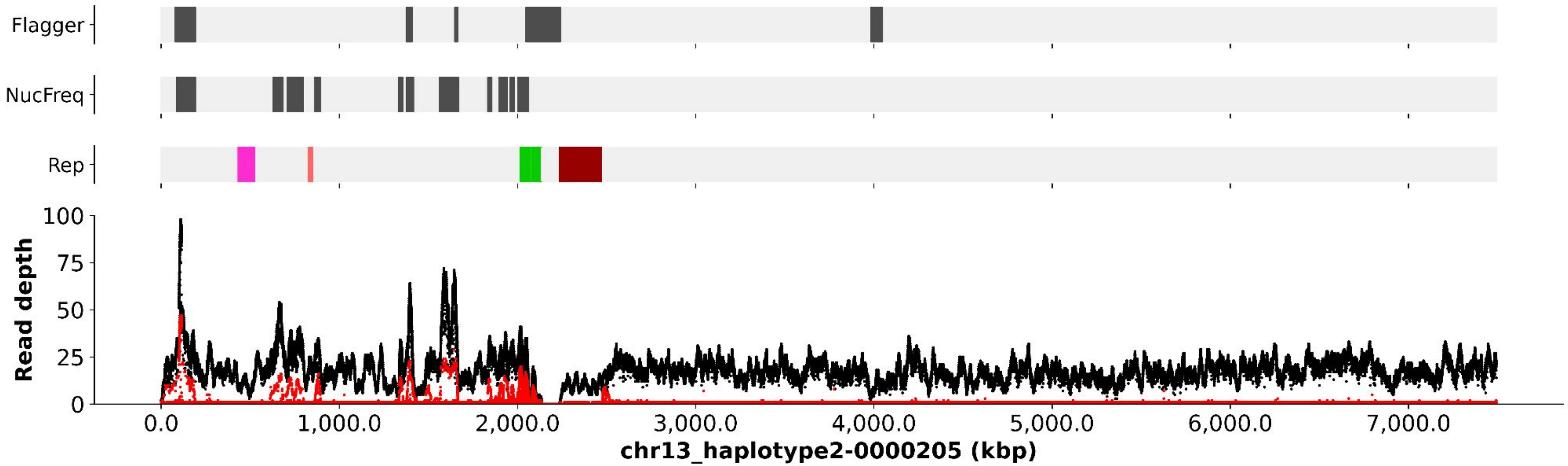

Flagger
NucFreq
Rep
Read depth
100
75
50
25
0
0.0
1,000.0
2,000.0
3,000.0
4,000.0
5,000.0
6,000.0
7,000.0
chr13_haplotype2-0000205 (kbp)

chr13_haplotype2-0000205

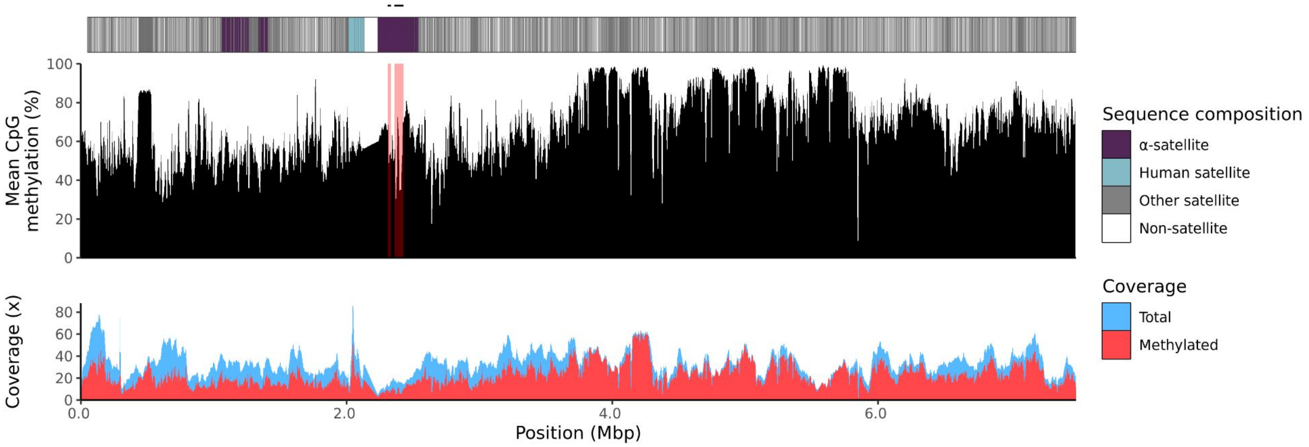

chr14

K200087_1_haplotype1-0000002_chr14

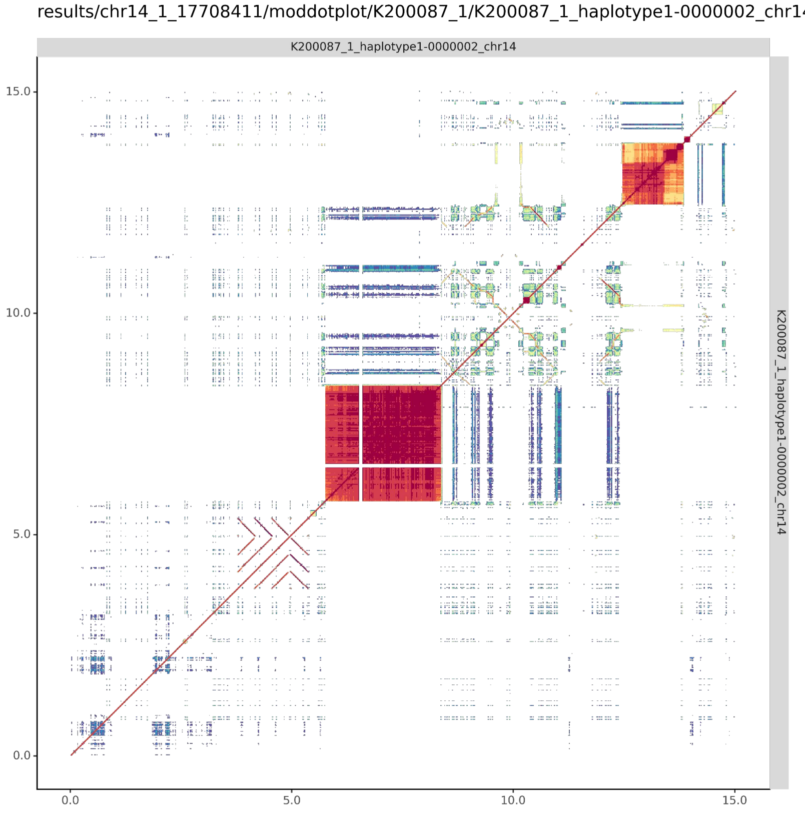

K200087_2_haplotype2-0000194_chr14

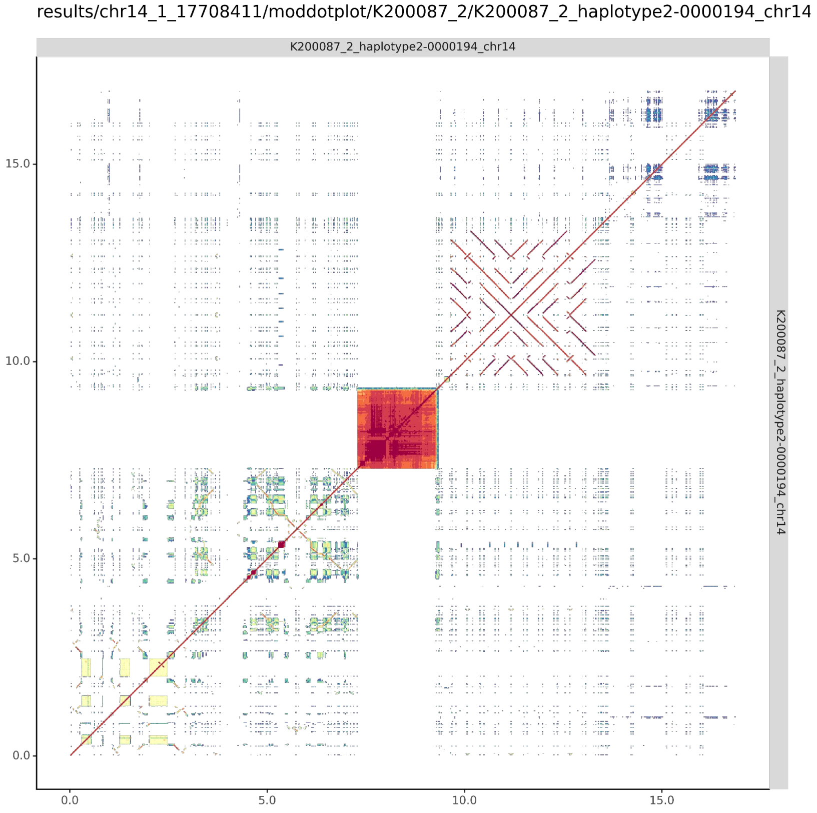

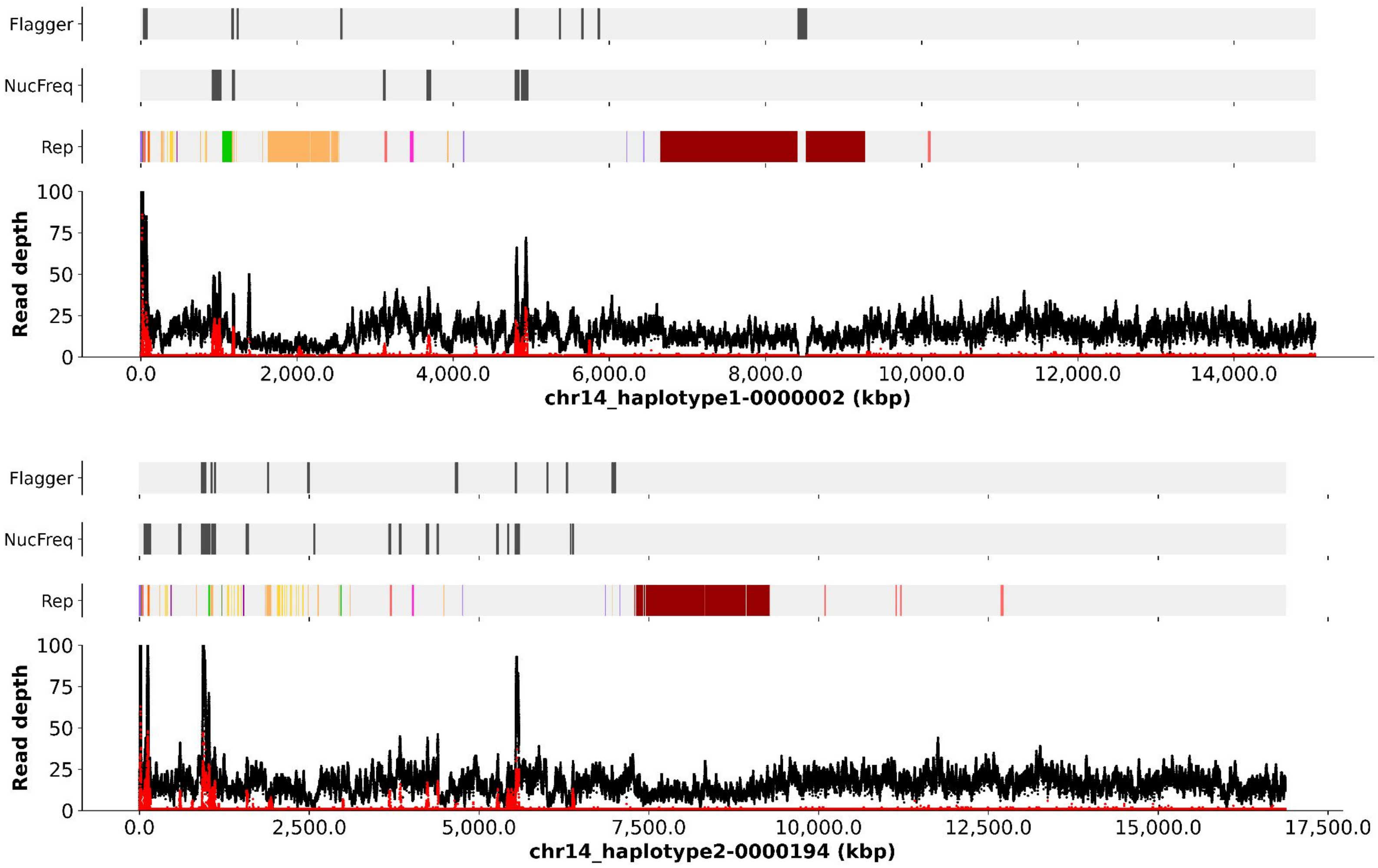

Flagger
NucFreq
Rep
Read depth
100
75
50
25
0
0.0
2,000.0
4,000.0
6,000.0
8,000.0
10,000.0
12,000.0
14,000.0
chr14_haplotype1-0000002 (kbp)
Flagger
NucFreq
Rep
Read depth
100
75
50
25
0
0.0
2,500.0
5,000.0
7,500.0
10,000.0
12,500.0
15,000.0
17,500.0
chr14_haplotype2-0000194 (kbp)

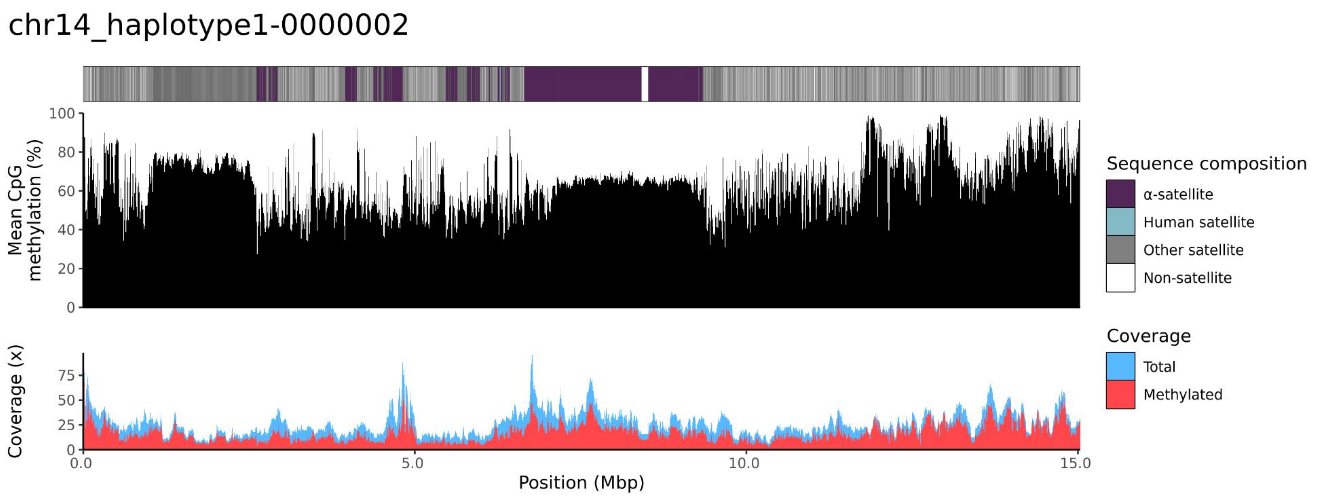
chr14_haplotype1-0000002
Mean CpG methylation (%)
Coverage (x)
Position (Mbp)
Sequence composition
α-satellite
Human satellite
Other satellite
Non-satellite
Coverage
Total
Methylated

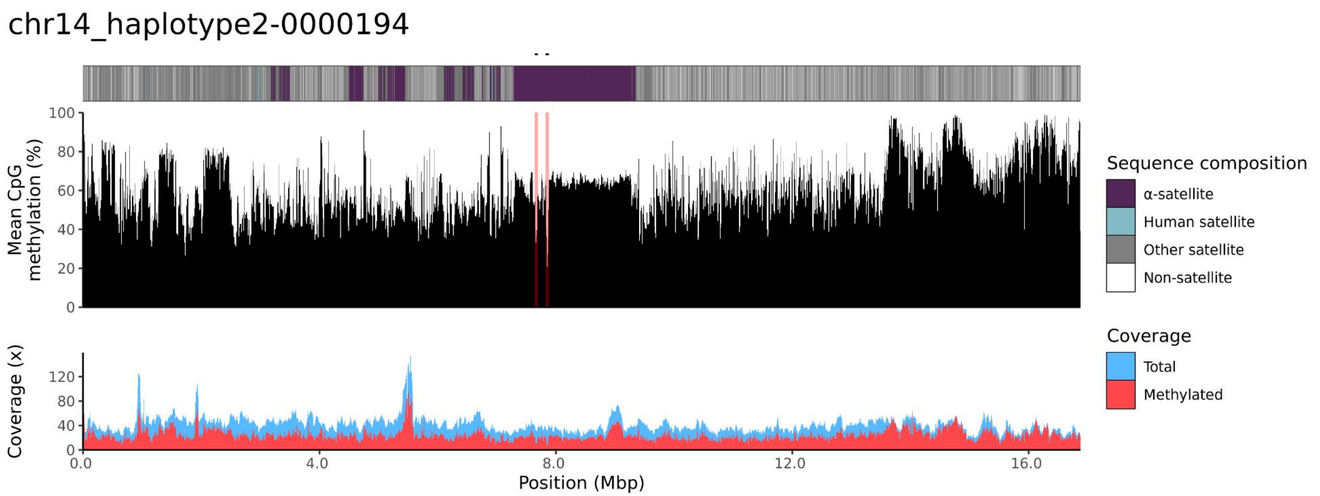
chr14_haplotype2-0000194
Mean CpG methylation (%)
Coverage (x)
Position (Mbp)
Sequence composition
α-satellite
Human satellite
Other satellite
Non-satellite
Coverage
Total
Methylated

chr15

K200087_1_haplotype1-0000014_chr15

K200087_2_haplotype2-0000206_chr15

results/chr15_1_22694466/moddotplot/K200087_1/K200087_1_haplotype1-0000014_chr15

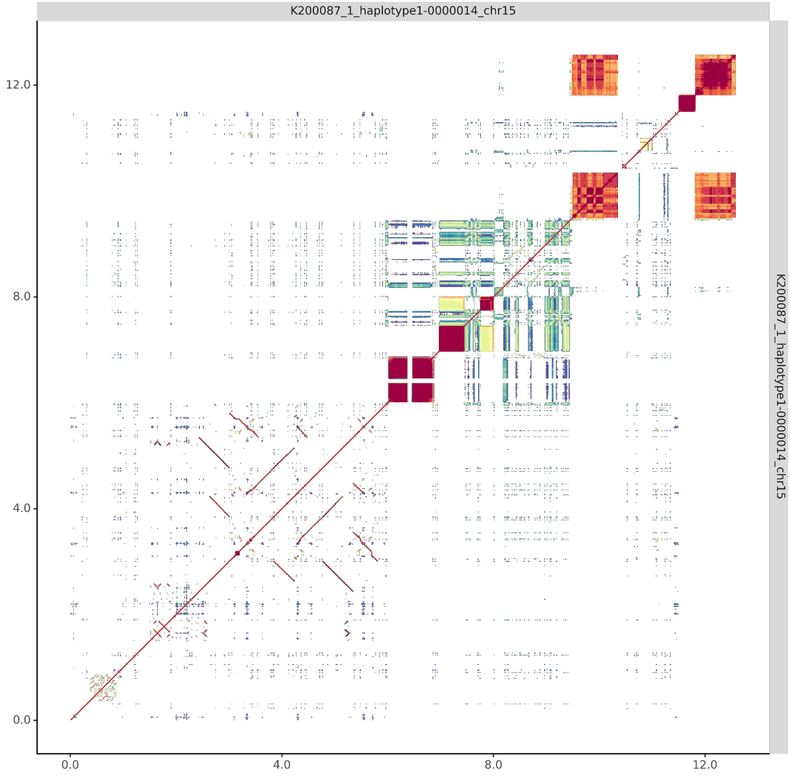

results/chr15_1_22694466/moddotplot/K200087_2/K200087_2_haplotype2-0000206_chr15

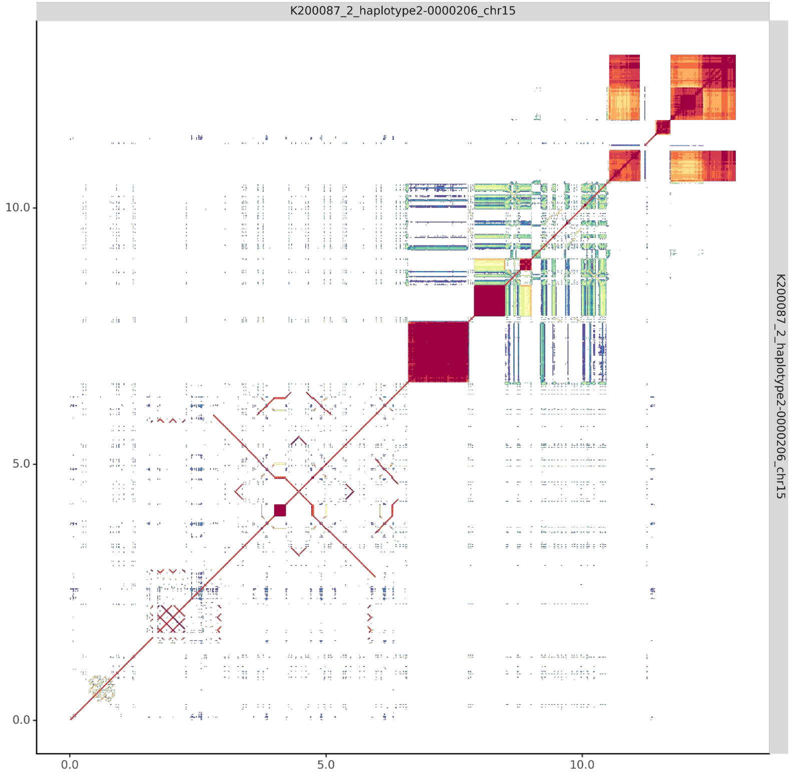

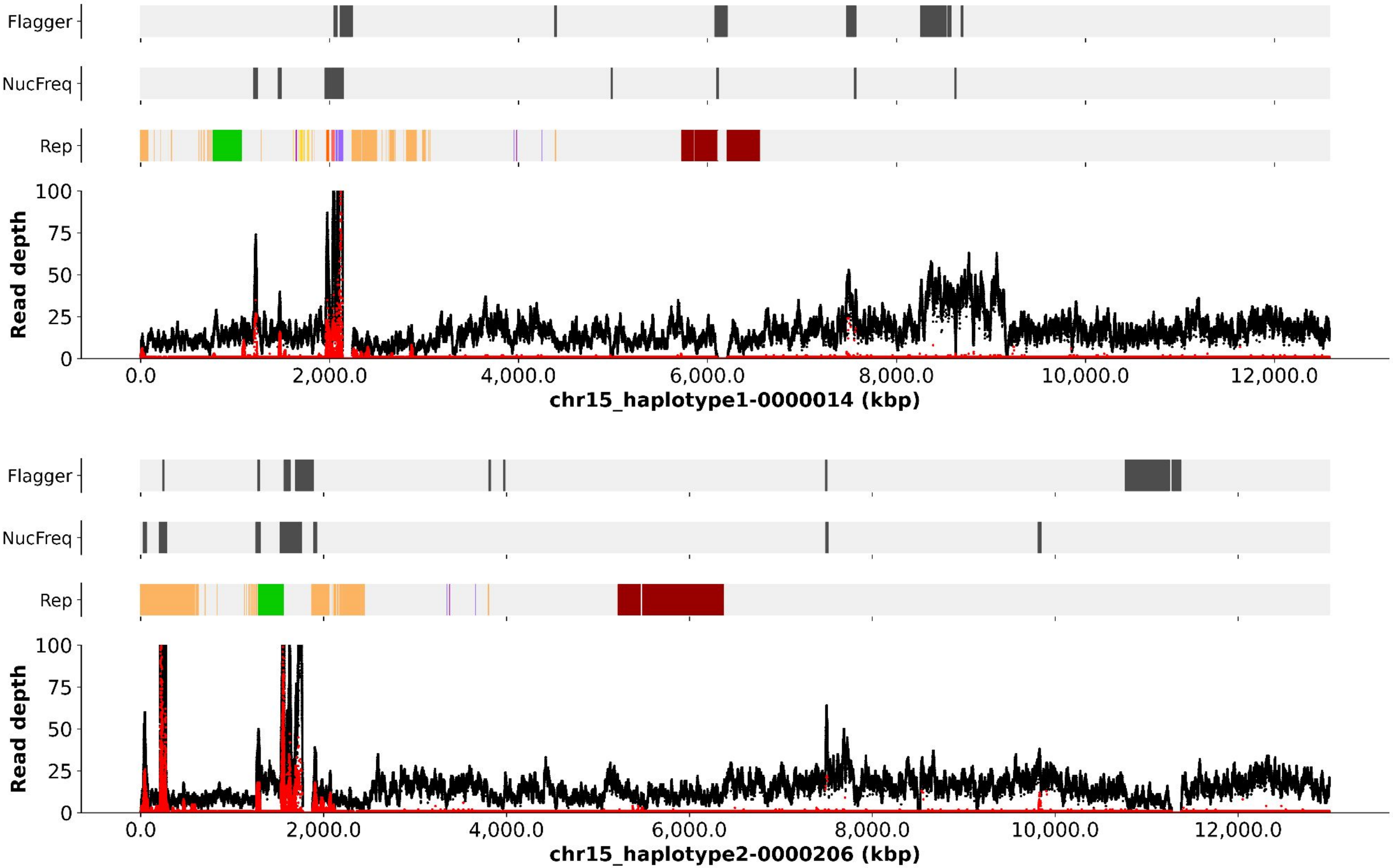

Flagger
NucFreq
Rep
Read depth
0
25
50
75
100
0.0
2,000.0
4,000.0
6,000.0
8,000.0
10,000.0
12,000.0
chr15_haplotype1-0000014 (kbp)
Flagger
NucFreq
Rep
Read depth
0
25
50
75
100
0.0
2,000.0
4,000.0
6,000.0
8,000.0
10,000.0
12,000.0
chr15_haplotype2-0000206 (kbp)

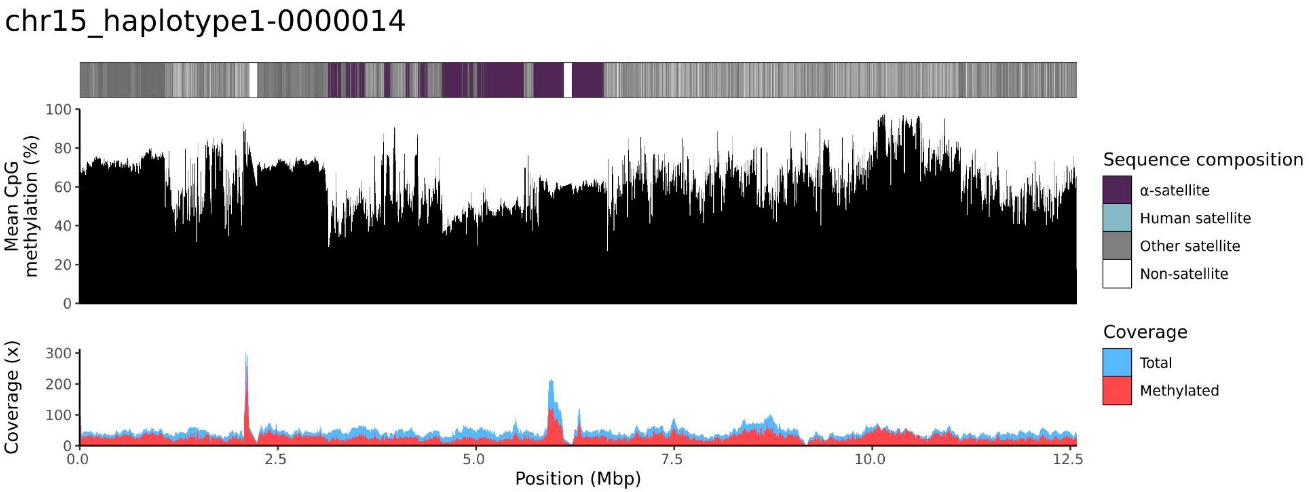
chr15_haplotype1-0000014
Mean CpG methylation (%)
Coverage (x)
Position (Mbp)
Sequence composition
α-satellite
Human satellite
Other satellite
Non-satellite
Coverage
Total
Methylated

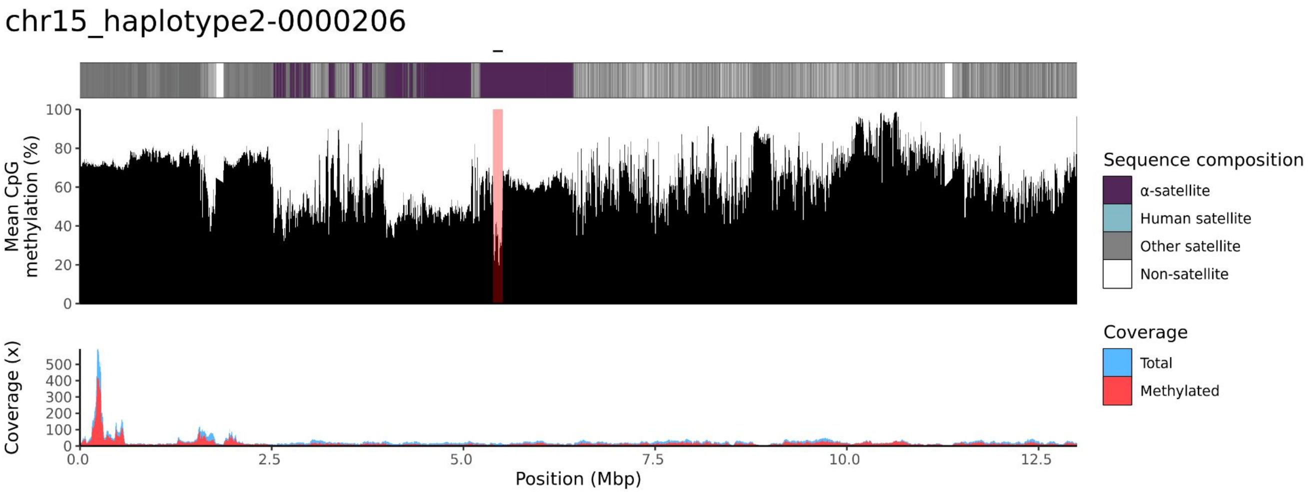
chr15_haplotype2-0000206
Mean CpG methylation (%)
Coverage (x)
Position (Mbp)
Sequence composition
α-satellite
Human satellite
Other satellite
Non-satellite
Coverage
Total
Methylated

chr21

K200087_1_haplotype1-0000020_chr21

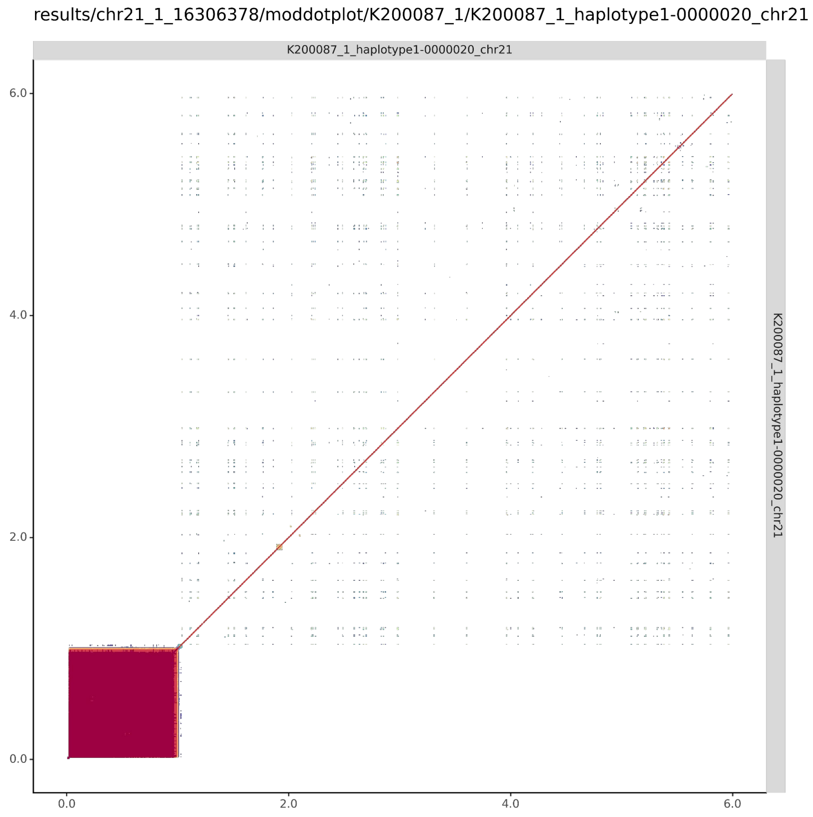

K200087_2_haplotype2-0000214_chr21

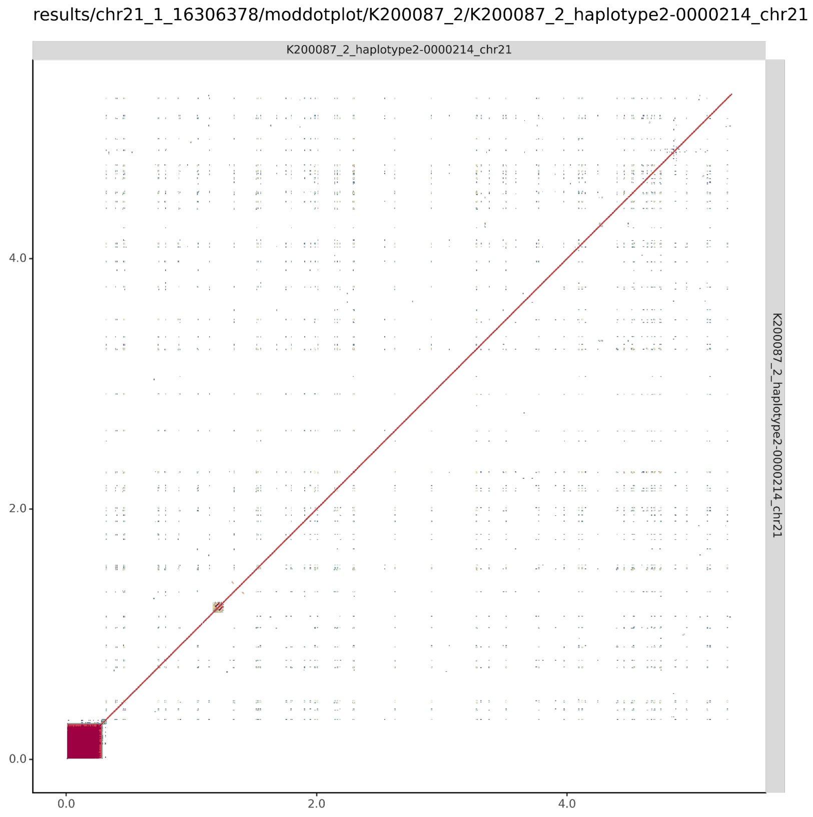

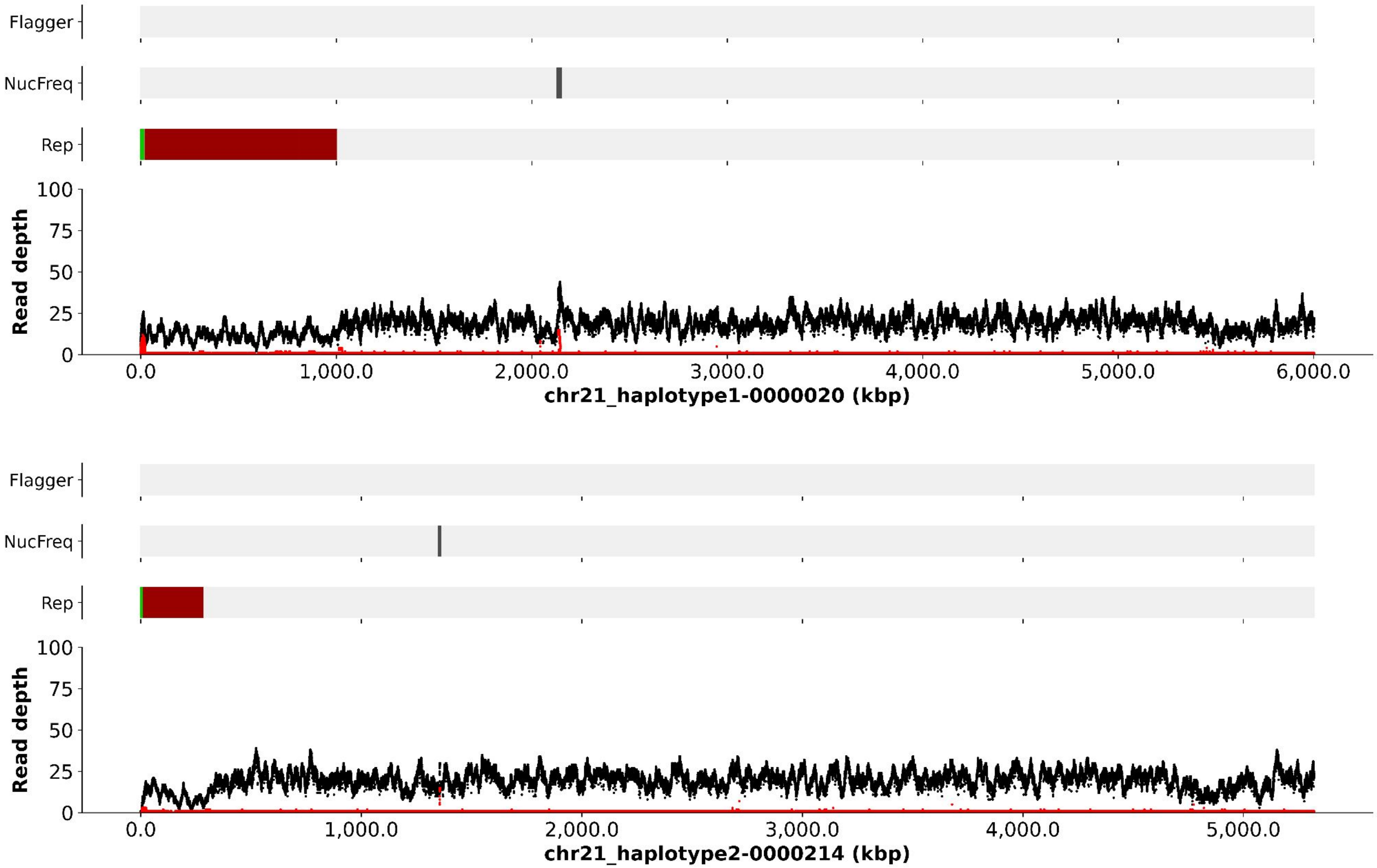

Flagger
NucFreq
Rep
Read depth
100
75
50
25
0
0.0
1,000.0
2,000.0
3,000.0
4,000.0
5,000.0
6,000.0
chr21_haplotype1-0000020 (kbp)
Flagger
NucFreq
Rep
Read depth
100
75
50
25
0
0.0
1,000.0
2,000.0
3,000.0
4,000.0
5,000.0
chr21_haplotype2-0000214 (kbp)

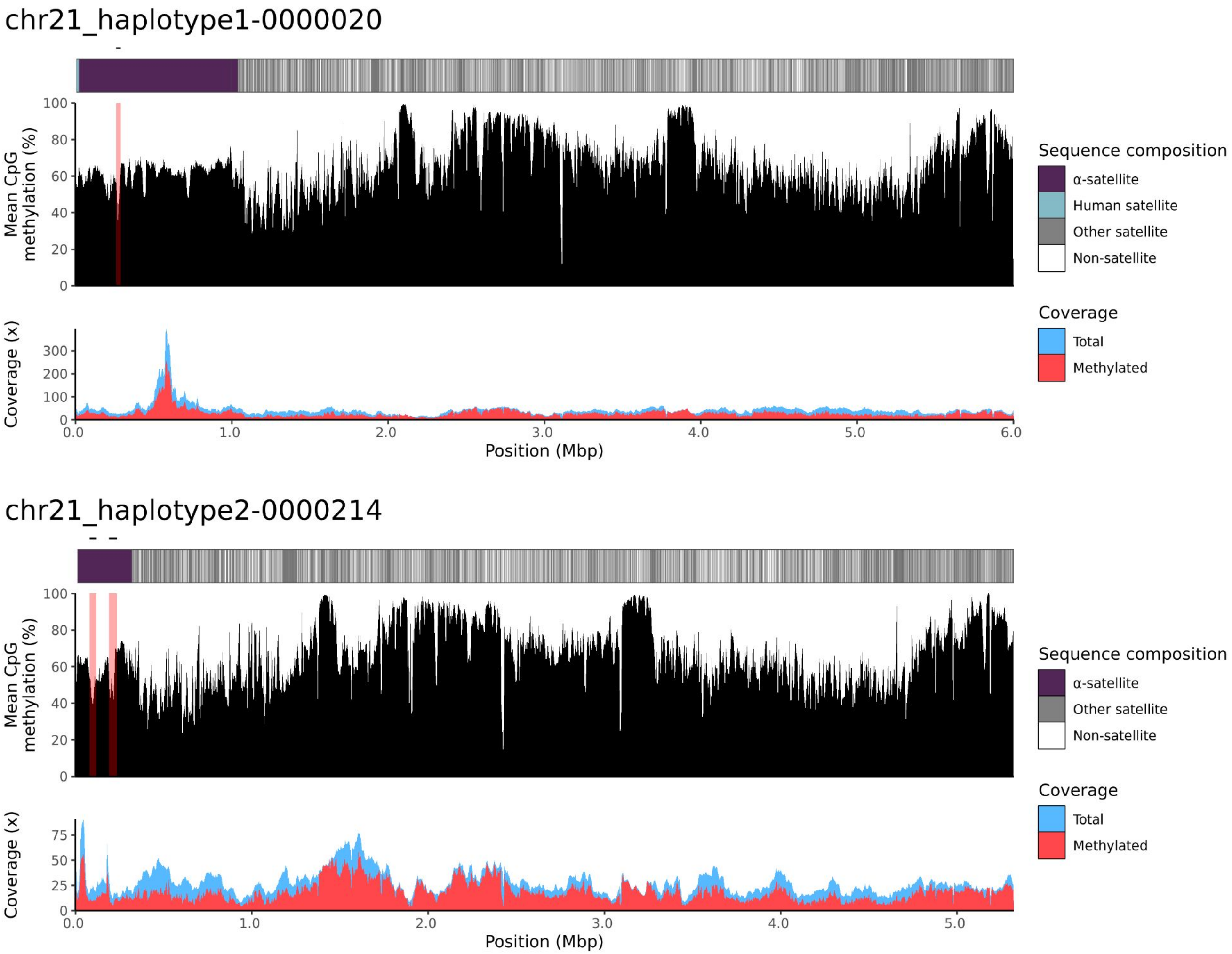
chr21_haplotype1-0000020
Mean CpG methylation (%)
100
80
60
40
20
0
Coverage (x)
300
200
100
0
0.0
1.0
2.0
3.0
4.0
5.0
6.0
Position (Mbp)
Sequence composition
α-satellite
Human satellite
Other satellite
Non-satellite
Coverage
Total
Methylated
chr21_haplotype2-0000214
Mean CpG methylation (%)
100
80
60
40
20
0
Coverage (x)
75
50
25
0
0.0
1.0
2.0
3.0
4.0
5.0
Position (Mbp)
Sequence composition
α-satellite
Other satellite
Non-satellite
Coverage
Total
Methylated

chr22

K200087_1_haplotype1-0000031_chr22

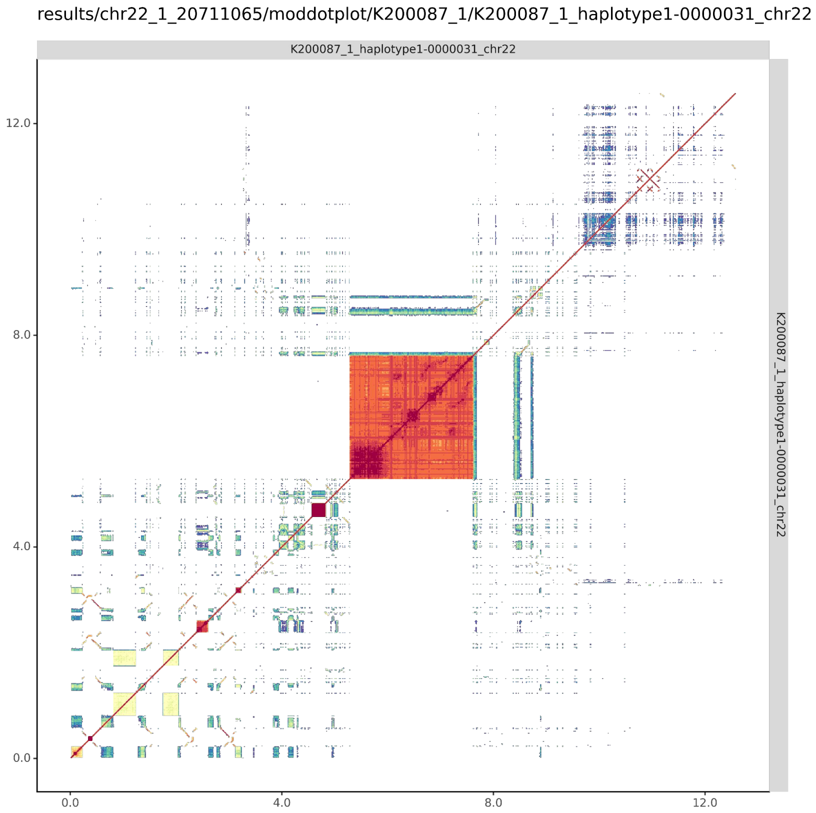

K200087_2_haplotype2-0000227_chr22

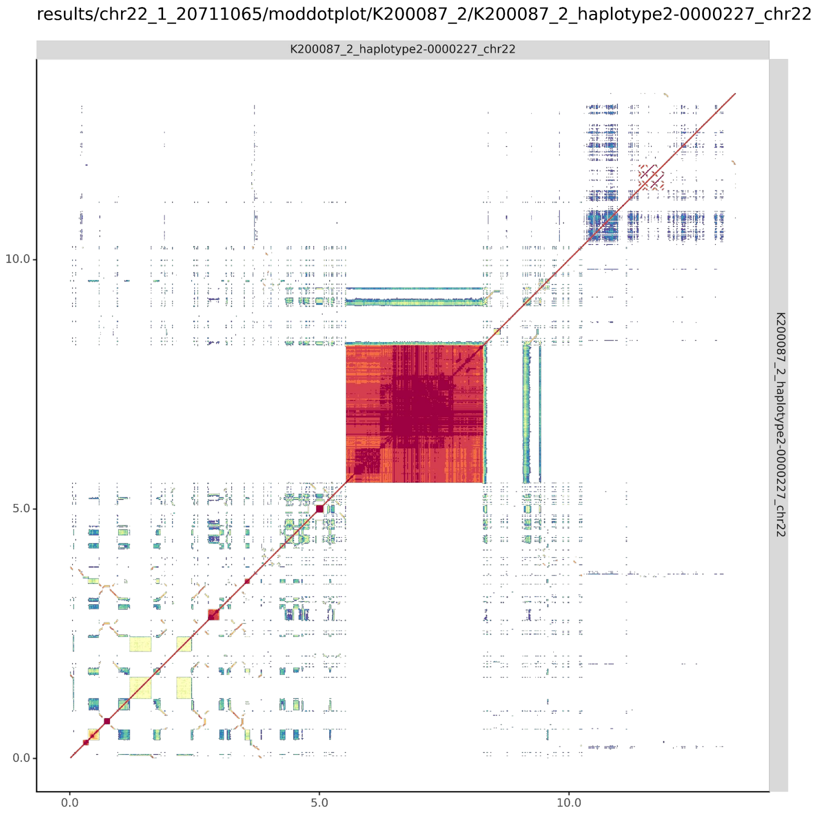

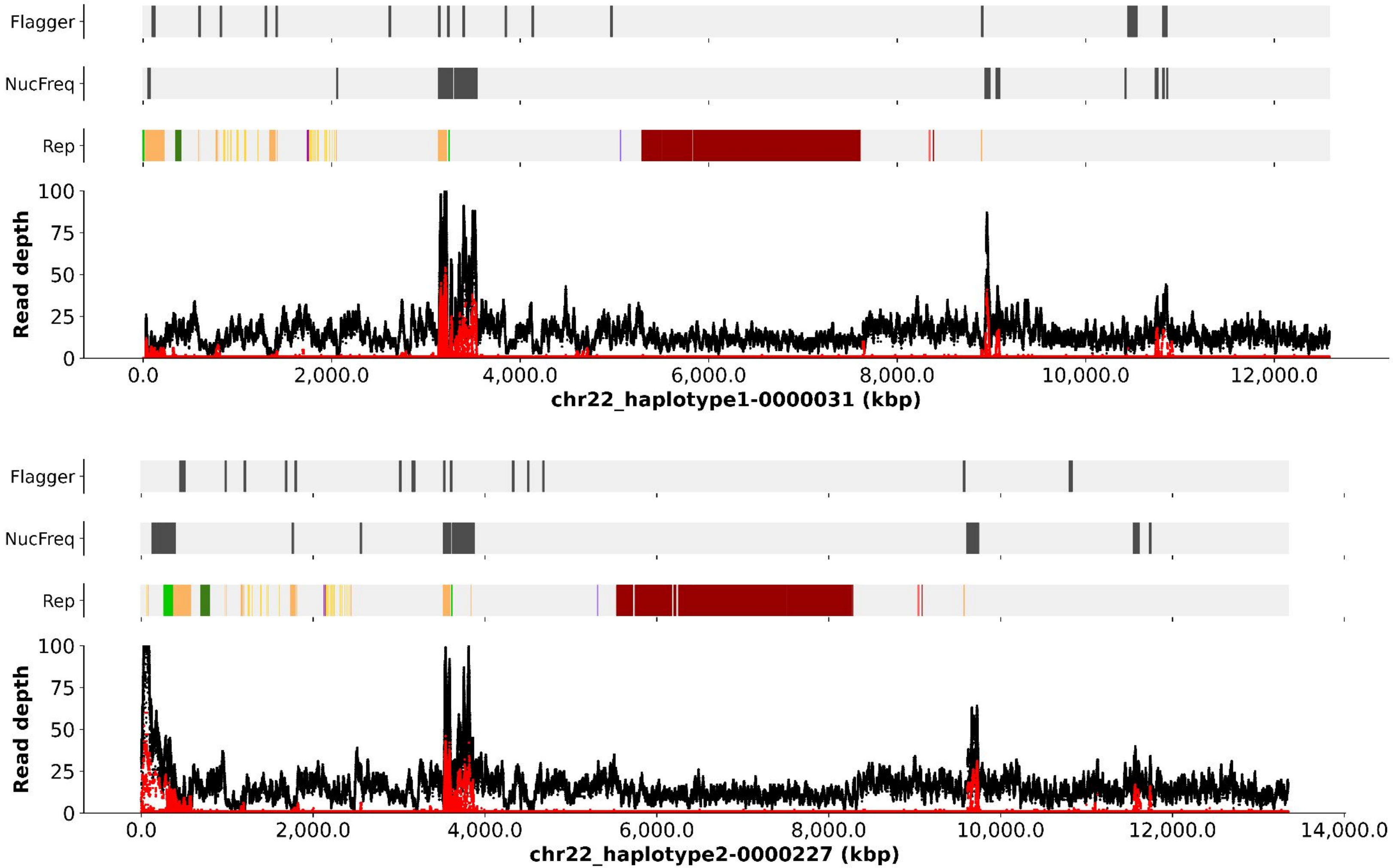

Flagger
NucFreq
Rep
Read depth
100
75
50
25
0
0.0
2,000.0
4,000.0
6,000.0
8,000.0
10,000.0
12,000.0
chr22_haplotype1-0000031 (kbp)
Flagger
NucFreq
Rep
Read depth
100
75
50
25
0
0.0
2,000.0
4,000.0
6,000.0
8,000.0
10,000.0
12,000.0
14,000.0
chr22_haplotype2-0000227 (kbp)

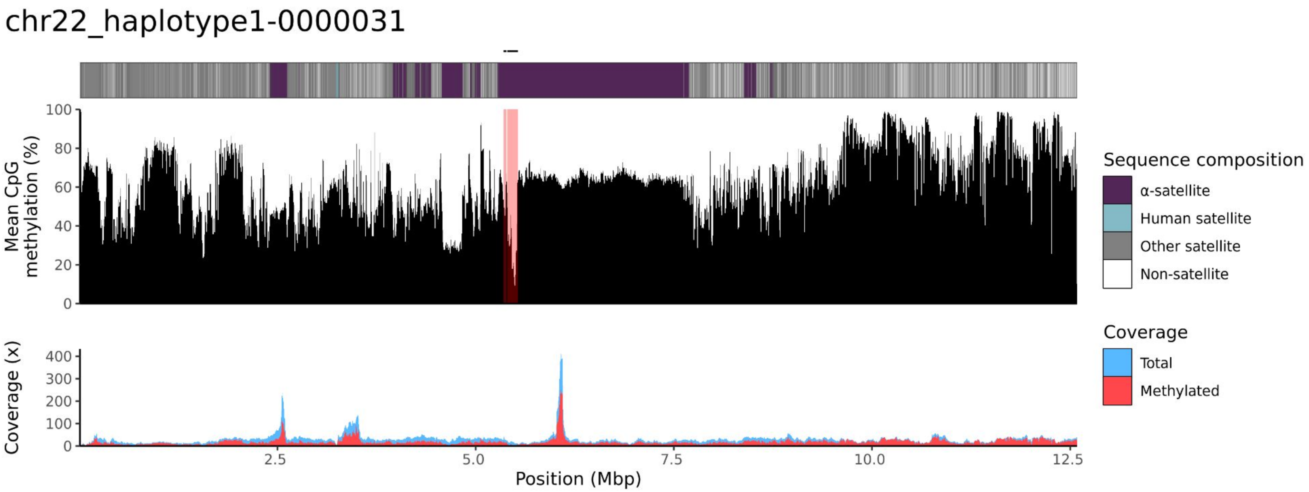
chr22_haplotype1-0000031
Mean CpG methylation (%)
0
20
40
60
80
100
Coverage (x)
0
100
200
300
400
2.5
5.0
7.5
10.0
12.5
Position (Mbp)
Sequence composition
α-satellite
Human satellite
Other satellite
Non-satellite
Coverage
Total
Methylated

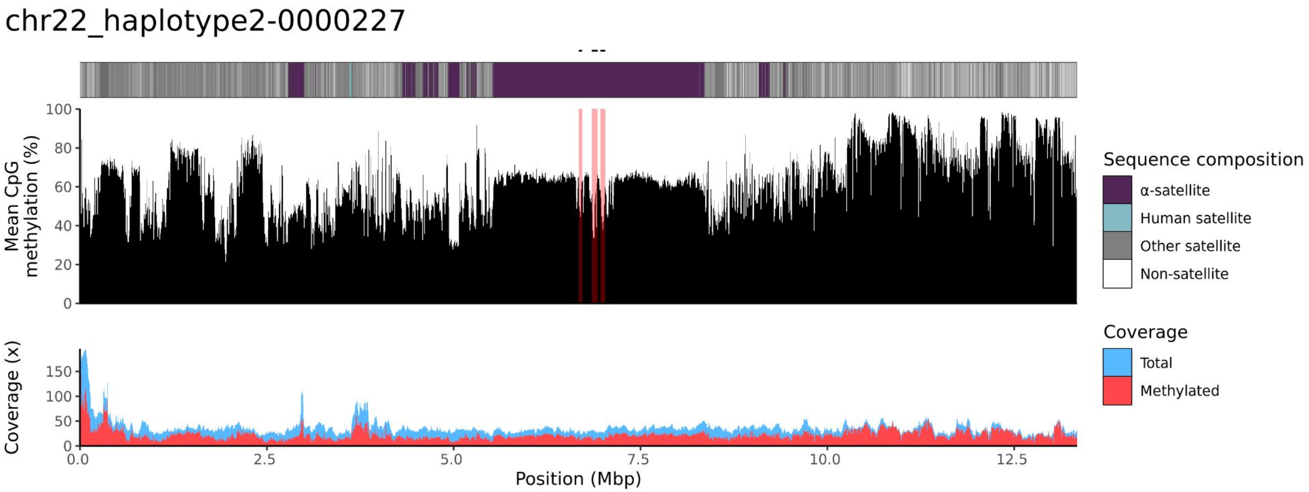
chr22_haplotype2-0000227
Mean CpG methylation (%)
0
20
40
60
80
100
Coverage (x)
0
50
100
150
0.0
2.5
5.0
7.5
10.0
12.5
Position (Mbp)
Sequence composition
α-satellite
Human satellite
Other satellite
Non-satellite
Coverage
Total
Methylated
