# Supplementary material for: Human acrocentric chromosome short arm de novo mutation and recombination
Source: bioRxiv. 2025 Dec 17:2025.12.16.694519. Preprint. [Version 1] doi: 10.64898/2025.12.16.694519 (PMC12724521; doi:10.64898/2025.12.16.694519)
Supplement: Supplement 8 [file NIHPP2025.12.16.694519v1-supplement-8.pdf]

bioRxiv preprint doi: https://doi.org/10.64898/2025.12.16.694519; this version posted December 17, 2025. The copyright holder for this preprint (which was not certified by peer review) is the author/funder, who has granted bioRxiv a license to display the preprint in perpetuity. It is made available under aCC-BY 4.0 International license.

Supplementary Figures for:
Human acrocentric chromosome short arm *de novo* mutation and recombination

Figures S1-S10

Jiadong Lin[1], F. Kumara Mastrorosa[1], Michelle D. Noyes[1], DongAhn Yoo[1], Arang Rhie[2], David Porubsky[1,3], Kendra Hoekzema[1], Katherine M. Munson[1], Nidhi Koundinya[1], W. Scott Watkins[4], Lynn B. Jorde[4], Aaron R. Quinlan[4], Deborah W. Neklason[5], Adam M. Phillippy[2], Evan E. Eichler[1,6]

Affiliations
[1]Department of Genome Sciences, University of Washington School of Medicine, Seattle, WA, USA
[2]Genome Informatics Section, Center for Genomics and Data Science Research, National Human Genome Research Institute, National Institutes of Health, Bethesda, MD, USA
[3]European Molecular Biology Laboratory (EMBL), Genome Biology Unit, Heidelberg, Germany
[4]Department of Human Genetics, University of Utah, Salt Lake City, UT, USA
[5]Department of Internal Medicine, University of Utah, Salt Lake City, UT, USA
[6]Howard Hughes Medical Institute, University of Washington, Seattle, WA, USA

Corresponding author
Evan E. Eichler, Ph.D.
Department of Genome Sciences
University of Washington School of Medicine
3720 15th Ave NE, S413C
Box 355065
Seattle, WA 98195-5065
Email: ee3@uw.edu
Tel: 1-206-543-9526

bioRxiv preprint doi: https://doi.org/10.64898/2025.12.16.694519; this version posted December 17, 2025. The copyright holder for this preprint (which was not certified by peer review) is the author/funder, who has granted bioRxiv a license to display the preprint in perpetuity. It is made available under aCC-BY 4.0 International license.

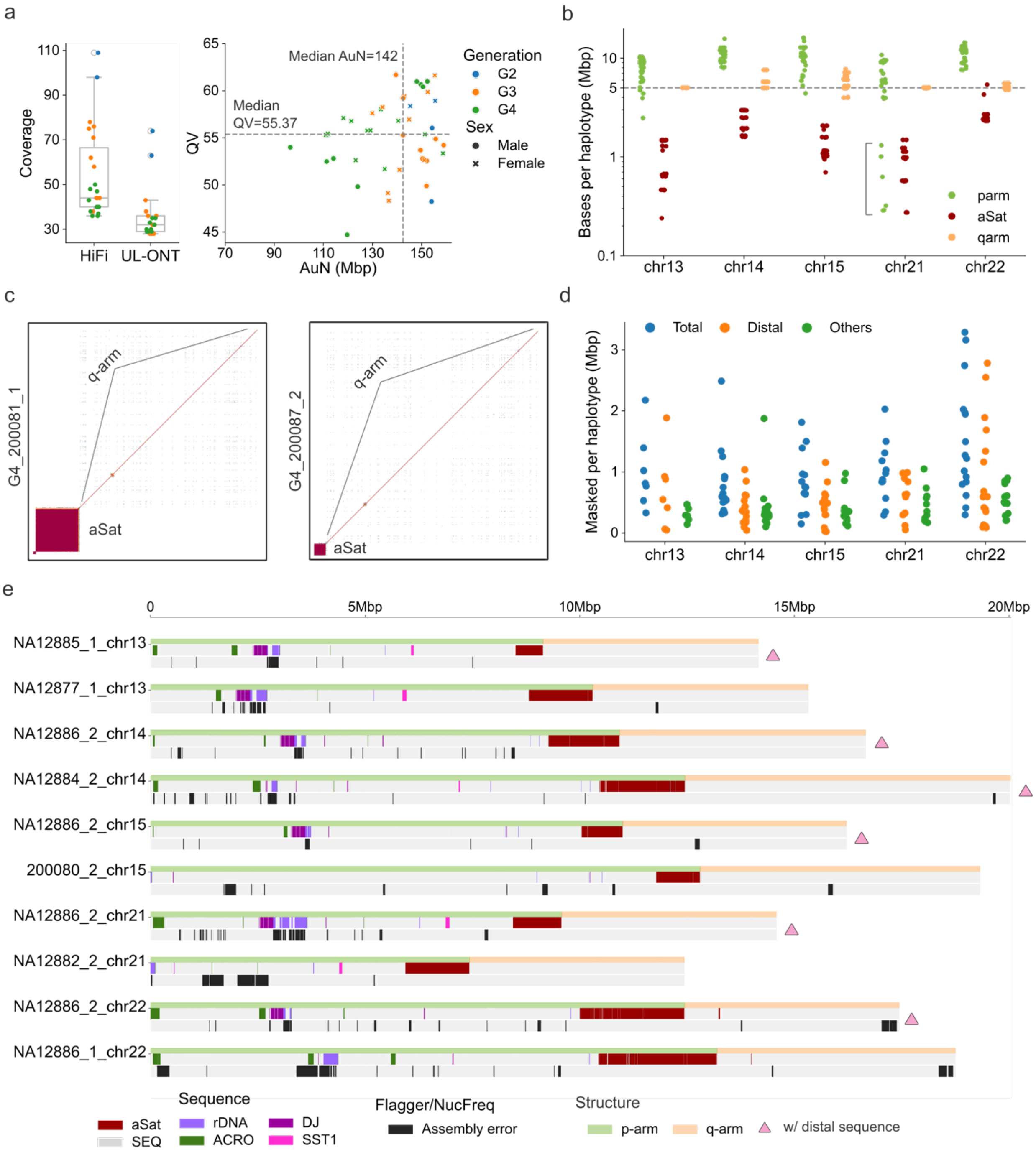

Figure S1. Short arm assembly evaluation. a) Data coverage and the assembly quality (base quality QV and contiguity AuN). The QV is calculated with Merqury (v1.1) based on Illumina short-read data. b) Number of bases per haplotype for p-arm, aSat (α-satellite), and q-arm sequences. The six pq-scatigs with shorter p-arms are indicated by the bracket. c) Two examples of the six short p-arms that only contain a limited proportion of pericentromeric sequence on the short arm side. d) NucFreq and Flagger identified incorrect bases per haplotype for all sequences (Total), distal sequence (Distal), and sequence from non-distal regions (Others). e) Examples of structure diagrams comparing pq-scatigs with distal sequence and without distal sequence. The distal sequence is completed if it contains the subtelomeric ACRO repeat (dark green) and the distal junction (DJ; dark purple) flank rDNA array.

bioRxiv preprint doi: https://doi.org/10.64898/2025.12.16.694519; this version posted December 17, 2025. The copyright holder for this preprint (which was not certified by peer review) is the author/funder, who has granted bioRxiv a license to display the preprint in perpetuity. It is made available under aCC-BY 4.0 International license.

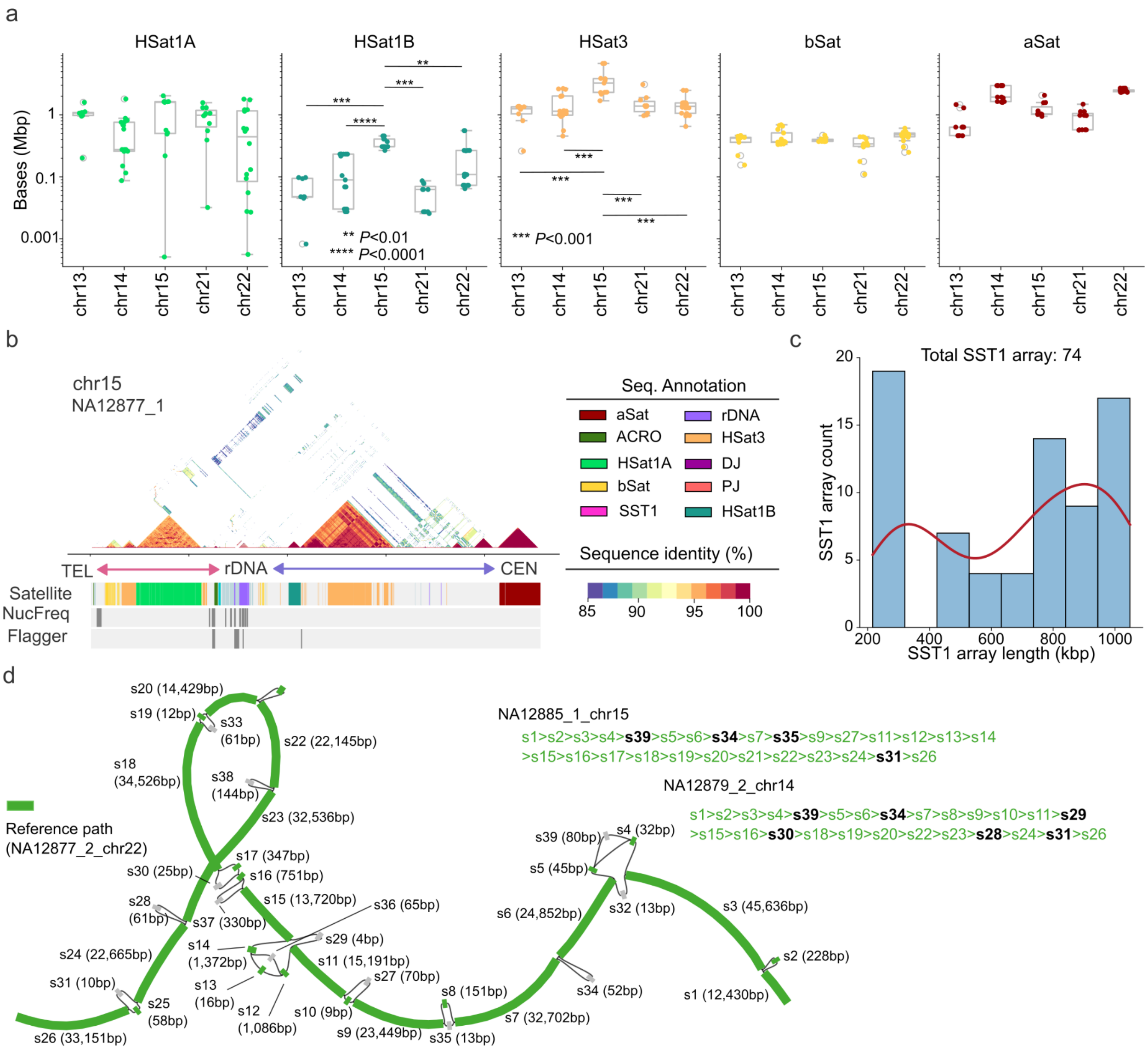

Figure S2. Genetic variations of short arm sequence. a) Comparison of satellite sequence among different chromosomes. A significant difference is determined using Mann-Whitney-Wilcoxon two-sided test; $**P<0.01$, $***P<0.001$, $****P<0.0001$. b) Satellite sequence organization on chr15 with a long HSat3 expansion on the proximal portion of p-arm. The diverged HSat3 sequence between HSat1B and HSat3 shares ~95% identity with HSat3 repeats but is different from HSat1B. c) Length distribution of all SST1 repeat arrays within this family without assembly error. d) Minigraph (v0.21) using 12 correctly assembled distal junction (DJ) sequences within this family. The two example paths are the chr14 and chr15 DJ sequences from NA12885 and NA12879, respectively, when using chr22 from NA12877 as the reference path. The nodes in green indicate the reference sequence and bolded black indicates variation in the sequence.

bioRxiv preprint doi: https://doi.org/10.64898/2025.12.16.694519; this version posted December 17, 2025. The copyright holder for this preprint (which was not certified by peer review) is the author/funder, who has granted bioRxiv a license to display the preprint in perpetuity. It is made available under aCC-BY 4.0 International license.

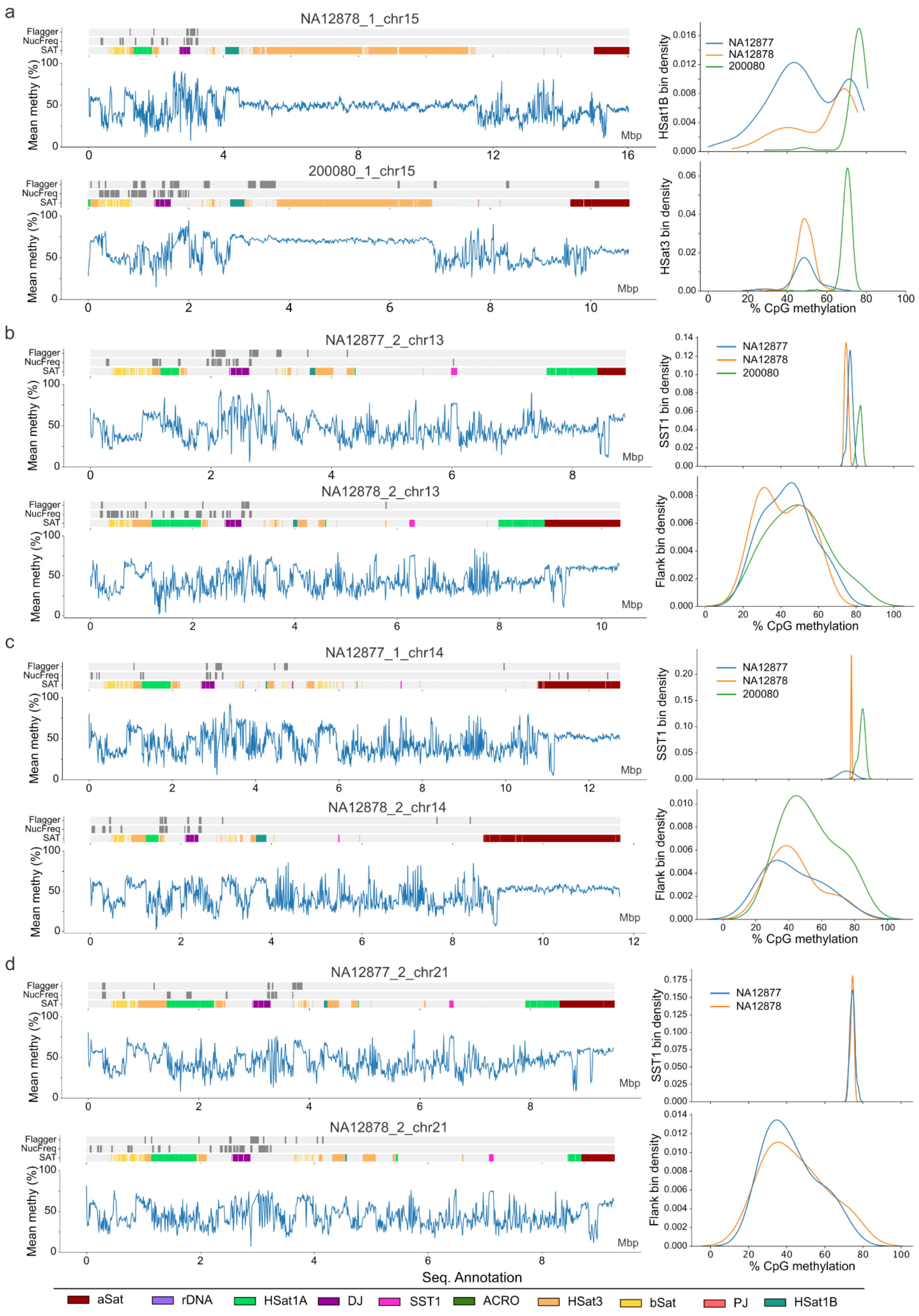

bioRxiv preprint doi: https://doi.org/10.64898/2025.12.16.694519; this version posted December 17, 2025. The copyright holder for this preprint (which was not certified by peer review) is the author/funder, who has granted bioRxiv a license to display the preprint in perpetuity. It is made available under aCC-BY 4.0 International license.

Figure S3. Epigenetic variation of satellite sequence on short arms. The assembly quality, satellite sequence annotation, and methylation of unrelated haplotypes of HSat3 from a) chr15 and SST1 from b) chr13, c) chr14 and d) chr21. a) The right panel shows the density of binned CpG methylation of HSat1B and HSat3 for each haplotype. b-d) The right panels show the density of binned CpG methylation of SST1 and the 50 kbp flanking region for each haplotype.

bioRxiv preprint doi: https://doi.org/10.64898/2025.12.16.694519; this version posted December 17, 2025. The copyright holder for this preprint (which was not certified by peer review) is the author/funder, who has granted bioRxiv a license to display the preprint in perpetuity. It is made available under aCC-BY 4.0 International license.

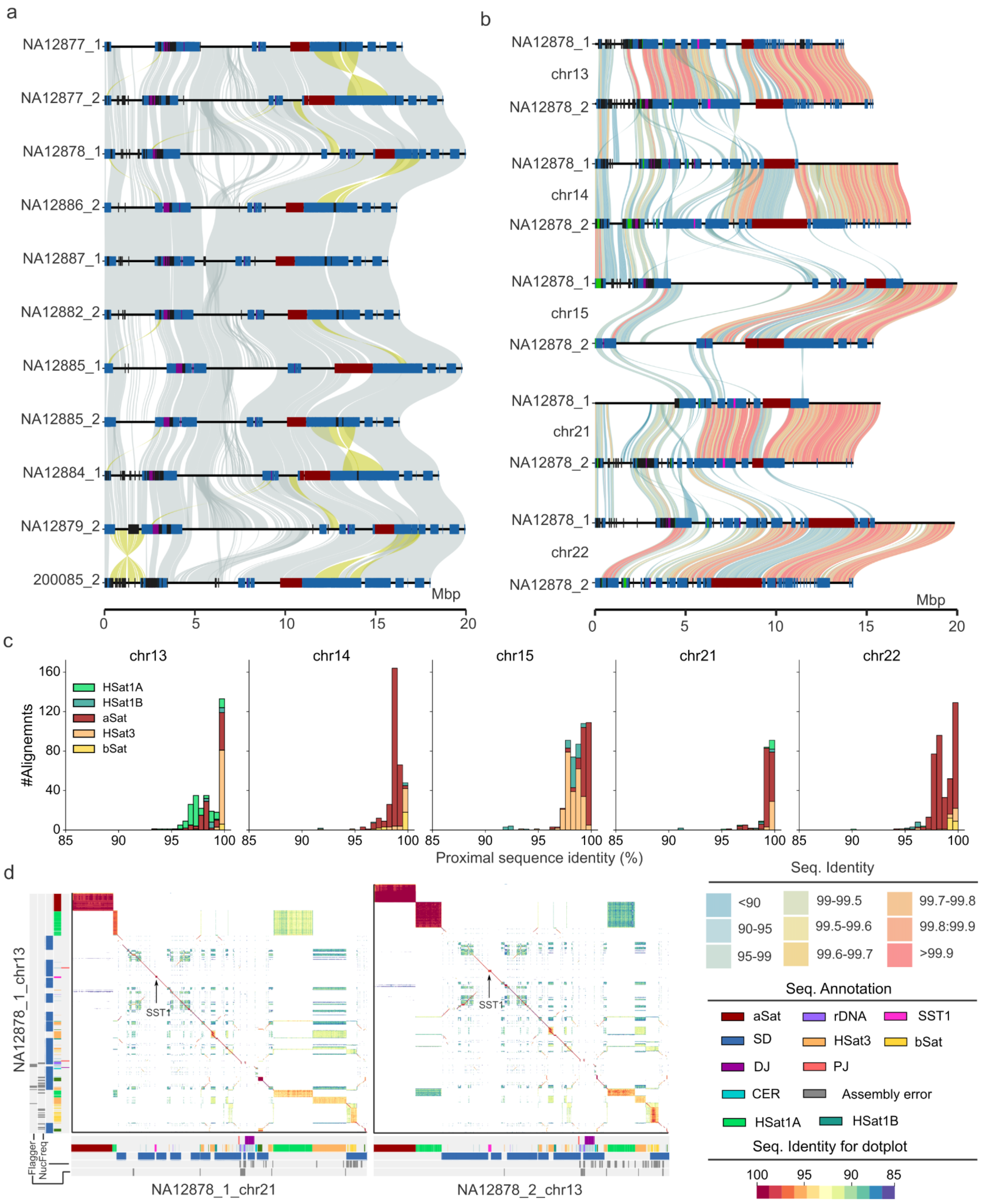

Figure S4. Allelic and nonallelic alignment of acrocentric short arms. a) Comparison of chr15 pq-scatigs containing both distal and proximal sequence assembled. The alignment reveals chr15 q-arm inversion polymorphism (reversed alignment in yellow) within this family. b) SVbyEye plot shows the all-vs-all alignment of all pq-scatigs from NA12878, revealing extensive allelic and nonallelic variations on short arms. c) Allelic sequence identity of 10 kbp binned alignment on short arms. This plot only shows the alignment in HSat1, aSat (α-satellite),

bioRxiv preprint doi: https://doi.org/10.64898/2025.12.16.694519; this version posted December 17, 2025. The copyright holder for this preprint (which was not certified by peer review) is the author/funder, who has granted bioRxiv a license to display the preprint in perpetuity. It is made available under aCC-BY 4.0 International license.

HSat3, and bSat (β-satellite). All alignments inside incorrectly assembled regions are excluded in the evaluation. d) ModDotPlot (v0.9.8) shows the variation between chr13 homologous chromosomes as well as chr13 and chr21 heterologous chromosomes.

bioRxiv preprint doi: https://doi.org/10.64898/2025.12.16.694519; this version posted December 17, 2025. The copyright holder for this preprint (which was not certified by peer review) is the author/funder, who has granted bioRxiv a license to display the preprint in perpetuity. It is made available under aCC-BY 4.0 International license.

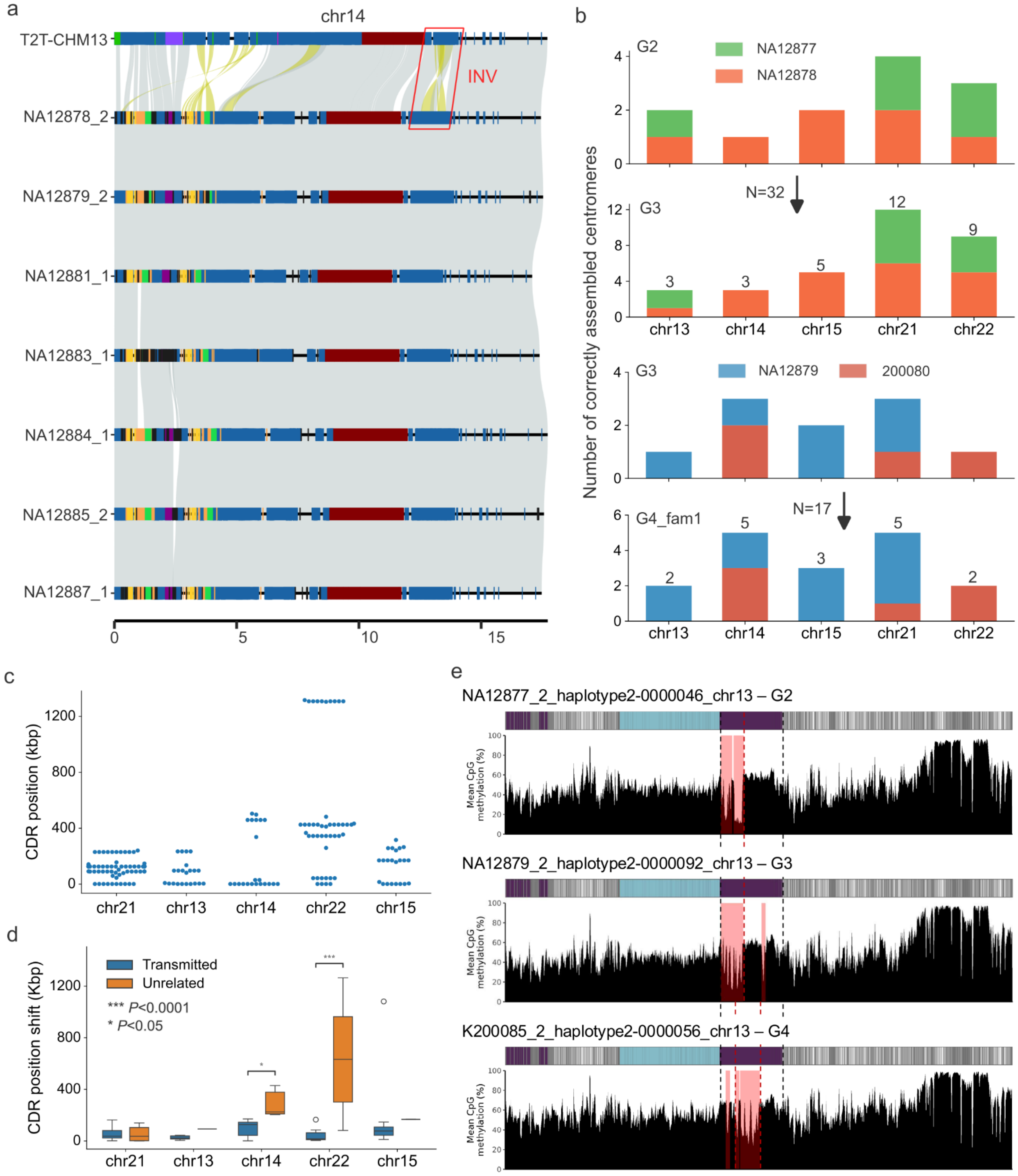

Figure S5. Intergenerational transmission of the short arms. a) Inheritance of an inversion detected against the T2T-CHM13 reference genome. b) Number of accessed intergenerational centromere transmissions from G2 to G3 and G3 to G4_fam1. c) Position of the centromere dip region (CDR), which is measured with respect to the start coordinate of the aSat (α-satellite) array. d) Comparison of CDR position shift between transmitted and unrelated individuals. A significant difference is determined using Mann-Whitney-Wilcoxon two-sided test; *P<0.05. ***P<0.001. Note there are not enough correctly assembled chr13 and chr15 centromeres from unrelated individuals (NA12877, NA12878 and 200080) to make a comparison. e) Example of

bioRxiv preprint doi: https://doi.org/10.64898/2025.12.16.694519; this version posted December 17, 2025. The copyright holder for this preprint (which was not certified by peer review) is the author/funder, who has granted bioRxiv a license to display the preprint in perpetuity. It is made available under aCC-BY 4.0 International license.

centromere transmitted from G2 to G4. The CDR positions in G2 and G3 are 3.6 kbp and 8 kbp to the start of the aSat, respectively, while the position becomes 53 kbp when transmitted to G4. The other three centromeres transmitted across two generations are shown in **Data S1**.

bioRxiv preprint doi: https://doi.org/10.64898/2025.12.16.694519; this version posted December 17, 2025. The copyright holder for this preprint (which was not certified by peer review) is the author/funder, who has granted bioRxiv a license to display the preprint in perpetuity. It is made available under aCC-BY 4.0 International license.

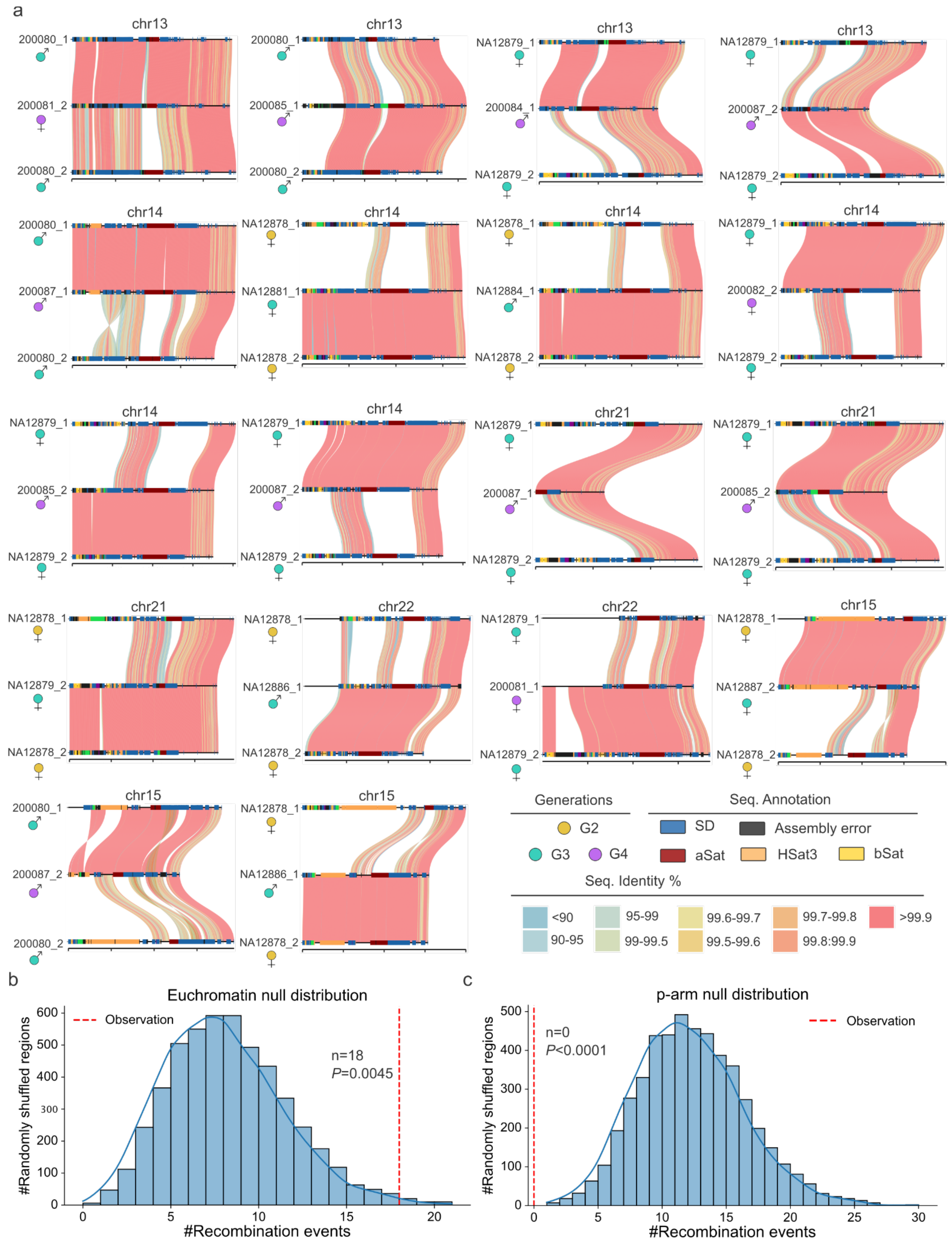

Figure S6. Summary of the 18 allelic recombinations and their comparison to the null distribution. a) The all-vs-all alignment of 18 q-arm pericentromeric recombinations visualized by SVbyEye. In each plot, the haplotype in the middle is the combination product and the

bioRxiv preprint doi: https://doi.org/10.64898/2025.12.16.694519; this version posted December 17, 2025. The copyright holder for this preprint (which was not certified by peer review) is the author/funder, who has granted bioRxiv a license to display the preprint in perpetuity. It is made available under aCC-BY 4.0 International license.

parental haplotypes are on top and bottom, respectively. The read-depth profile and breakpoint alignments of each recombination are shown in **Data S1**. b) Euchromatin recombination distribution created by randomly shuffling segments across the genome exclude SAACs, metacentric pericentromeric regions on T2T-CHM13. The red dashed line is the number of observed recombinations on 25 Mbp acrocentric q-arm pericentromeric regions. c) Metacentric chromosome p-arm recombination distribution created by randomly shuffling a 36 Mbp segment on T2T-CHM13 SAAC proximal sequences. The red dashed line is the number of observed recombinations on SAAC regions of 38 Mbp.

bioRxiv preprint doi: https://doi.org/10.64898/2025.12.16.694519; this version posted December 17, 2025. The copyright holder for this preprint (which was not certified by peer review) is the author/funder, who has granted bioRxiv a license to display the preprint in perpetuity. It is made available under aCC-BY 4.0 International license.

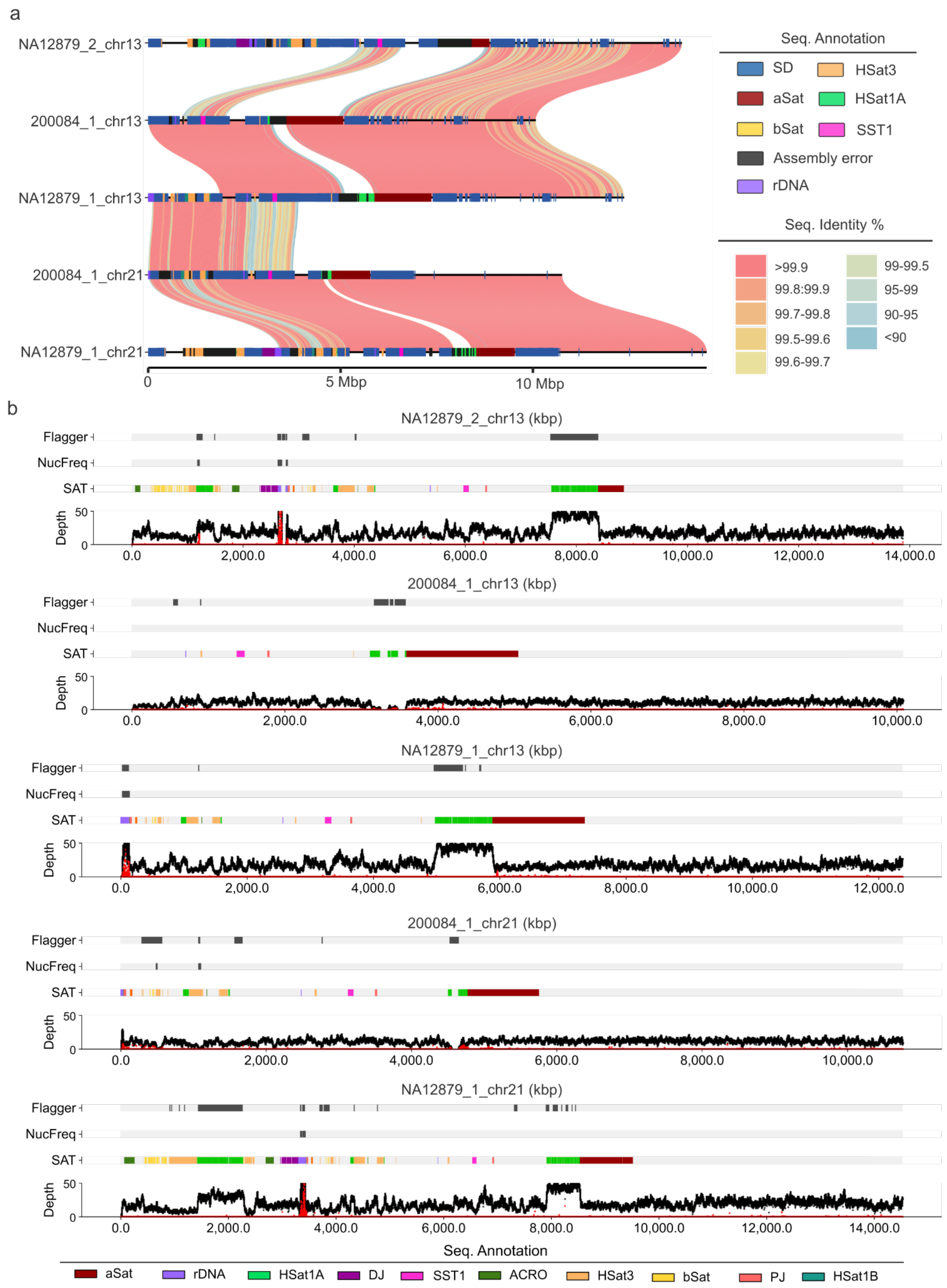

Figure S7. Chr13 and chr21 recombinations in sample 200084. a) SVbyEye plot shows the all-vs-all alignment of the two recombinations in 200084: one chr13-chr21 ectopic recombination

bioRxiv preprint doi: https://doi.org/10.64898/2025.12.16.694519; this version posted December 17, 2025. The copyright holder for this preprint (which was not certified by peer review) is the author/funder, who has granted bioRxiv a license to display the preprint in perpetuity. It is made available under aCC-BY 4.0 International license.

and the other homologous recombination on chr13 q-arm. b) Assembly quality assessed by Flagger and NucFreq for the five contigs involved in the two recombinations.

bioRxiv preprint doi: https://doi.org/10.64898/2025.12.16.694519; this version posted December 17, 2025. The copyright holder for this preprint (which was not certified by peer review) is the author/funder, who has granted bioRxiv a license to display the preprint in perpetuity. It is made available under aCC-BY 4.0 International license.

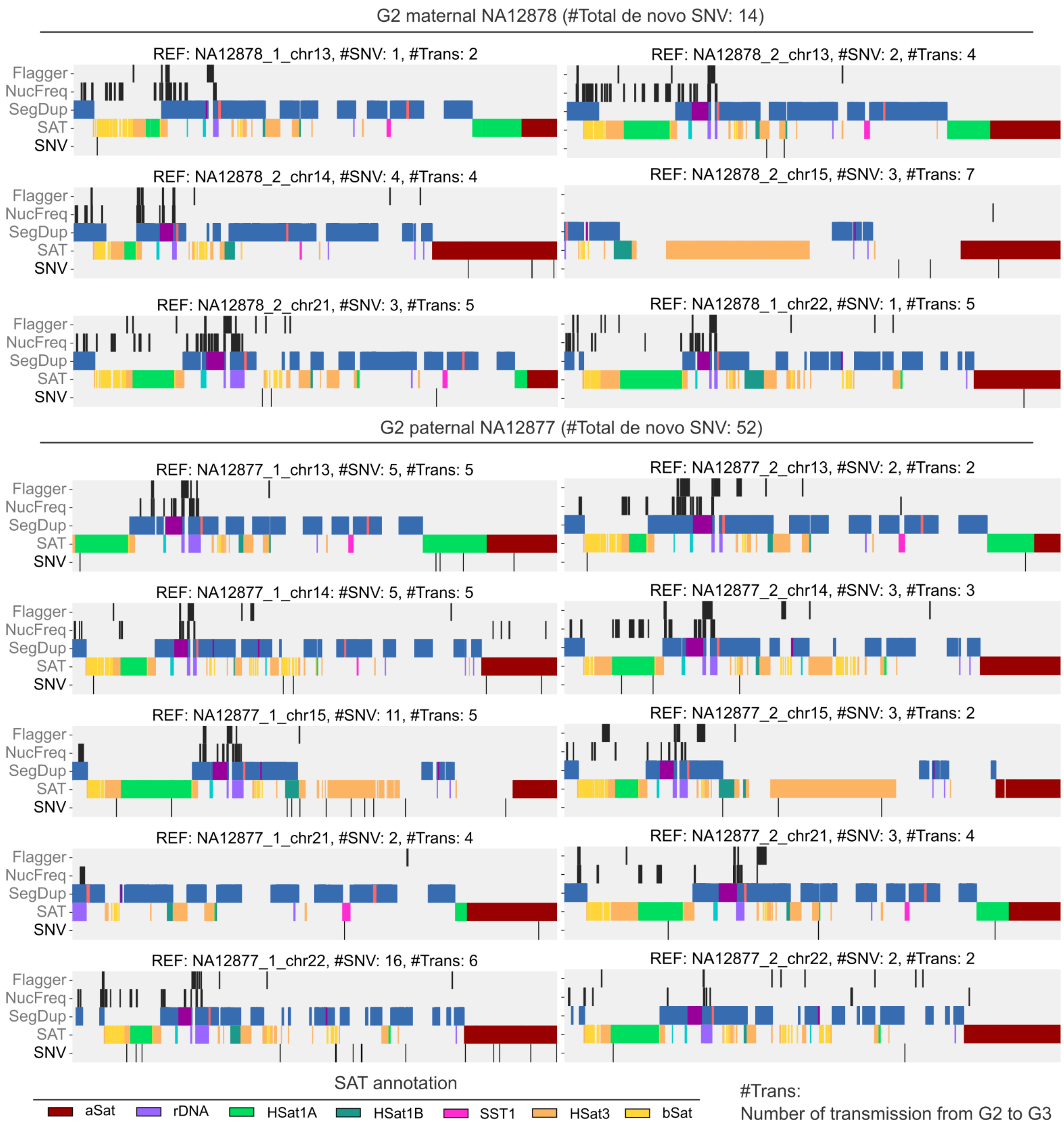

Figure S8. ***De novo*** **SNVs detected in G3 against G2 parents NA12877 and NA12878.** Each plot shows the detected variants against one parental haplotype. The reference haplotype name (REF), number of single-nucleotide variants (#SNV), and transmissions (#Trans) are indicated on the top of each plot. The number of transmissions is counted from G2 to eight G3 children (NA12879, NA12881, NA12882, NA12883, NA12884, NA12885, NA12886 and NA12887).

bioRxiv preprint doi: https://doi.org/10.64898/2025.12.16.694519; this version posted December 17, 2025. The copyright holder for this preprint (which was not certified by peer review) is the author/funder, who has granted bioRxiv a license to display the preprint in perpetuity. It is made available under aCC-BY 4.0 International license.

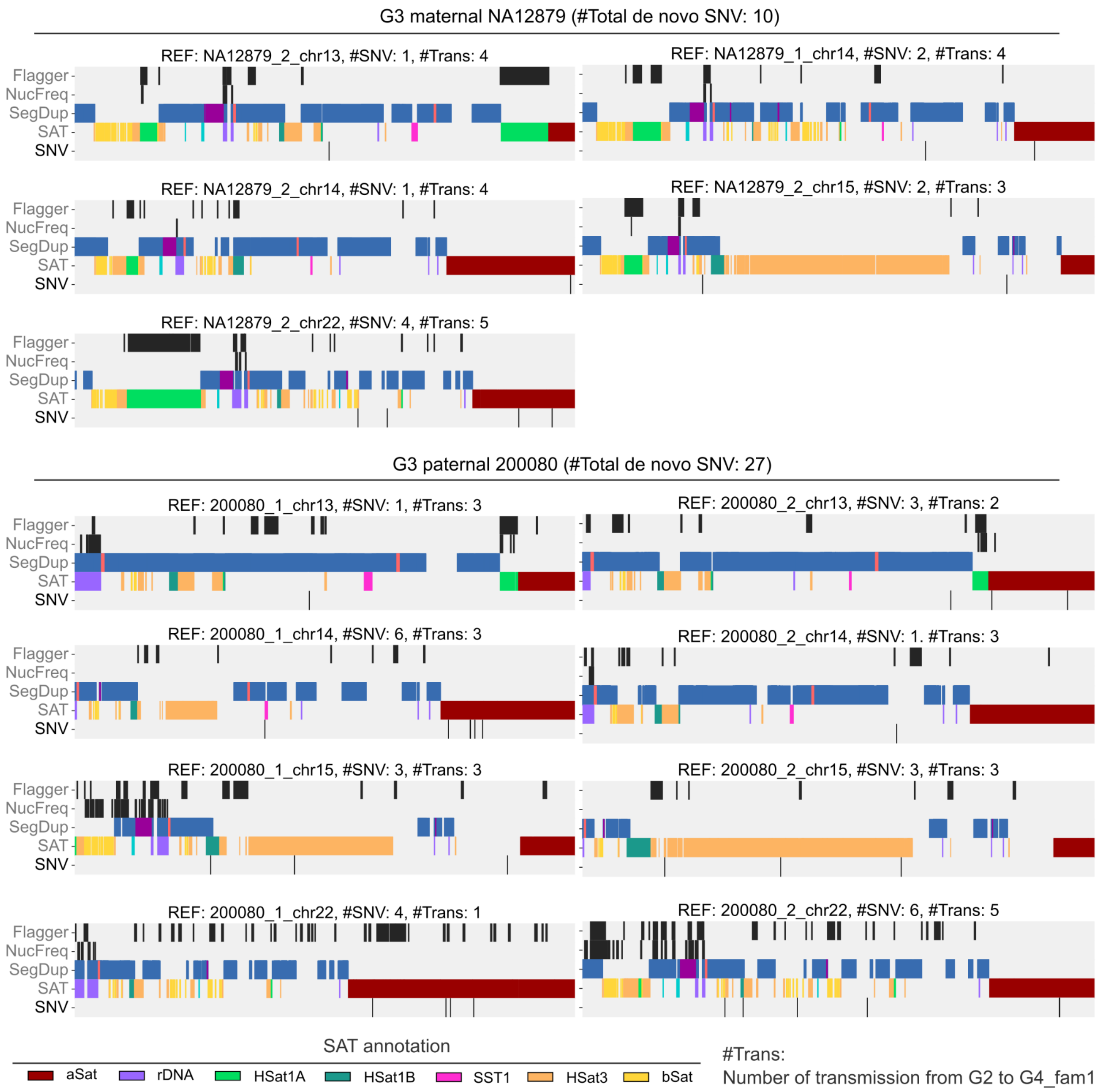

Figure S9. *De novo* SNVs detected in G4_fam1 against G3 parents NA12879 and 200080. Each plot shows the detected variants against one parental haplotype. The reference haplotype name (REF), number of single-nucleotide variants (#SNV), and transmissions (#Trans) are indicated correspondingly. The number of transmissions is counted from G3 (NA12879 and 200080) to five G4_fam1 children (200081, 200083, 200084, 200085 and 200087).

bioRxiv preprint doi: https://doi.org/10.64898/2025.12.16.694519; this version posted December 17, 2025. The copyright holder for this preprint (which was not certified by peer review) is the author/funder, who has granted bioRxiv a license to display the preprint in perpetuity. It is made available under aCC-BY 4.0 International license.

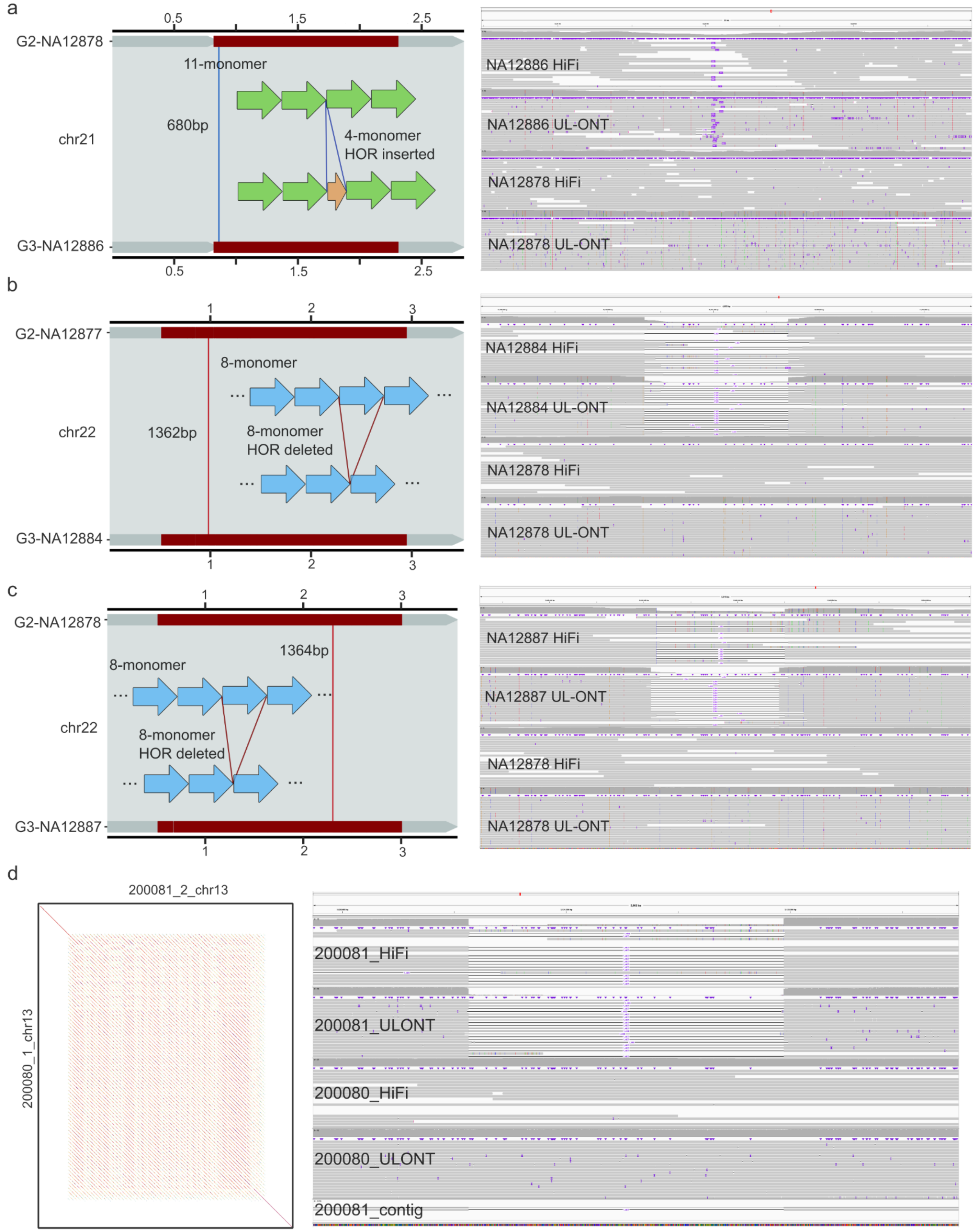

Figure S10. *De novo* SVs detected in HOR and SST1 arrays. a-c) Three *de novo* structural variants (SVs) detected in centromere higher-order repeat (HOR) arrays. The left alignment shows the transmission and location of the SV. The right panel is the IGV screenshot of HiFi and UL-ONT reads aligned to the paternal reference haplotype. d) The left panel shows the

bioRxiv preprint doi: https://doi.org/10.64898/2025.12.16.694519; this version posted December 17, 2025. The copyright holder for this preprint (which was not certified by peer review) is the author/funder, who has granted bioRxiv a license to display the preprint in perpetuity. It is made available under aCC-BY 4.0 International license.

difference of the SST1 repeat array with the reads supported (right IGV screenshot) one 1407 bp deletion inside the array.
